# Hydrogen-Bonded Matched Ion Pair Gold(I) Catalysis

**DOI:** 10.1021/acscatal.3c02638

**Published:** 2023-07-20

**Authors:** Àlex Martí, Gala Ogalla, Antonio M. Echavarren

**Affiliations:** Institute of Chemical Research of Catalonia (ICIQ), Barcelona Institute of Science and Technology (BIST), Av. Països Catalans 16, 43007 Tarragona, Spain; Departament de Química Orgànica i Analítica, Universitat Rovira i Virgili (URV), C/Marcel·lí Domingo s/n, 43007 Tarragona, Spain

**Keywords:** hydrogen bond interaction, chiral counterion, gold(I) catalysis, urea, phosphoramide, match−mismatch

## Abstract

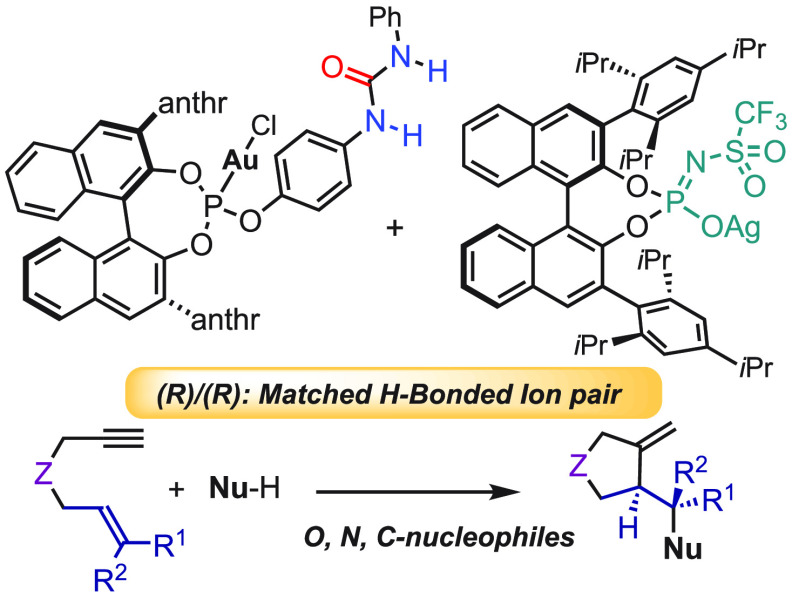

The enantioselective reaction of 1,6-enynes with *O*-, *N*-, and *C*-nucleophiles has been developed by matched ion pair gold(I) catalysis in which the chiral gold(I) cation and anion are H-bonded through a urea group. Very high levels of enantiocontrol are achieved (up to >99:1 er) for a broad scope of substrates. DFT studies demonstrate the importance of the H-bond donor group in anchoring the matched chiral cation- and anion-favoring additional noncovalent interactions.

Although the field of gold(I) catalysis has experienced an exponential growth in past decades,^1−9^ the development of enantioselective transformations with broader scope has been more difficult.^10−13^ After the pioneering work by Toste et al. on gold asymmetric counterion-directed catalysis (ACDC) for the enantioselective cyclization of allenes using chiral phosphate salts in combination with achiral digold complexes (Figure 1),^14−17^ the use of that concept has been used for the activation of alkyne^18−22^ or allene-containing substrates^23−27^ to circumvent some of the limitations in enantioselective gold(I) catalysis.^28−30^

**Figure 1:**
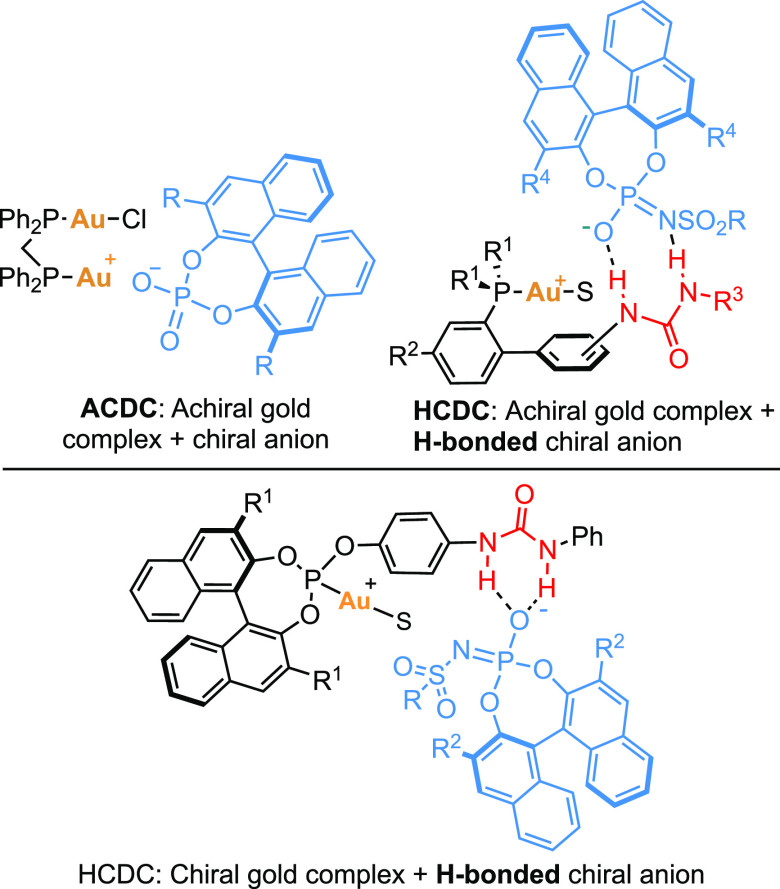
Gold(I) in the ACDC and HCDC strategies.

Our group recently introduced H-bonded counterion-directed catalysis (HCDC) (Figure 1) using achiral gold catalysts containing urea or squaramide H-bond donor motifs.^31,32^ In this approach, the H-bond donor facilitates the ligand-substrate exchange step and fixes the chiral information close to the reaction center, thereby allowing for an efficient transfer of the stereochemical information in cyclization reactions.^33−36^

Herein, we present the application of the HCDC approach with chiral binol-based phosphite gold(I) complexes^37^ equipped with urea groups together with matched chiral counterions for the enantioselective gold(I)-catalyzed nucleophilic addition to 1,6-enynes, which takes place with excellent enantioselectivities.

A library of phosphite gold(I) complexes carrying a urea was easily prepared in a one-pot, two-step procedure starting from chiral (*R*)-binaphthols with the desired 3,3′-substitution pattern or from achiral resorcinol [4]arenes (Figure 2). Additionally, chiral silver salts were prepared from commercially available chiral BINOLs and related biphenols (Figure 2). Urea groups in the *para*-, *meta*-, or *ortho*-position with respect to the phosphite were introduced to test the directing effect of the H-bond donor. We envisioned that chiral phosphoramidate silver salts would form more reactive catalysts since they are less basic than their corresponding phosphoric acid counterparts and, therefore, more easily substituted by the unsaturated substrates from the gold(I) coordination sphere.^31^

**Figure 2:**
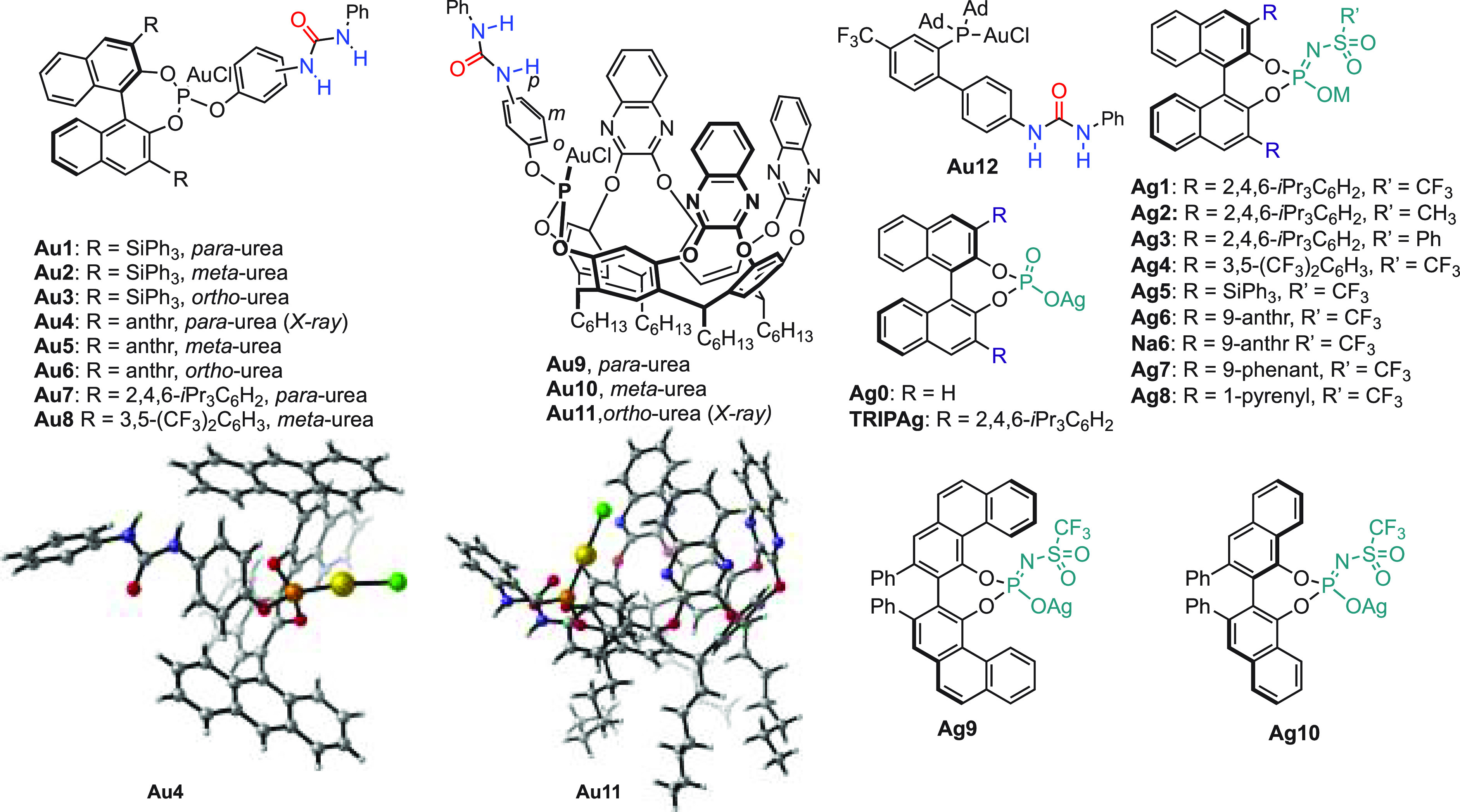
Library of chiral gold(I) complexes and Ag(I) salts. ^a^CYLview representations are for X-ray crystallography structures of **Au4** and **Au11**. Solvent molecules are omitted for clarity. **Au4** forms dimers in the solid state. Color code: P, orange; Au, yellow; Cl, green; O, red; N, blue; C, gray; and H, white. anthr = anthracenyl, phenant = phenantracenyl.

Our system proved to be particularly efficient in the addition of nucleophiles to 1,6-enynes.^38−52^ We first examined, using high-throughput experimentation (HTE), the addition of indole to **1a**(40,41) with different chiral gold(I) complexes and silver(I) salts.^53^ The best combinations were then scaled up to 0.05 mmol (Table 1). Gold(I) complexes with the urea in the *para*-position showed much higher reactivity than those with ureas at *ortho* or *meta*. Substituents in the 3,3′-position of the BINOL scaffolds in both the Au(I) catalyst and Ag salt also had an important impact. Through the use of toluene as solvent, a clear match-missmatch scenario was observed using (*R*)-**Au4** with the (*R*)- or (*S*)-enantiomers of **Ag6**, which led to **1a** with 95:5 *er* and 61.5:38.5 *er*, respectively (Table 1, entries 4 and 7). This observation was also present in other examples. For example, EtOH was used as a nucleophile with (*R*)-**Au1** or (*R*)-**Au4** in combination with the (*R*)- or (*S*)-enantiomers of the same Ag salt (Table 1, entries 8–11). Better yields and enantioselectivities were obtained in 1,4-dioxane. Thus, the combination of (*R*)-**Au4** with the (*R*)-enantiomers of **Ag1**, **Ag6**, or **Ag8** provided **1a** in >99:1, 97.5:2.5, and 95:5 *er*, respectively (Table 1, entries 13–15). Control experiments showed that neither (*R*)-**Au4** nor (*R*)-**Ag1** was active on its own (Table 1, entries 17 and 18). Whereas cavitand **Au9**,^52^ equipped with *para*-urea groups, gives satisfactory results with (*R*)-**Ag6** (Table 1, entry 16), achiral Au(I) complex **Au12**, which was found to be the optimal for the intramolecular formal [4 + 2] cycloaddition of arylalkynes with alkenes,^31^ together with (*R*)-**Ag1**, showed poor reactivity and low enantioinduction in the formation of **2a** (Table 1, entry 19). Silver salt AgSbF_6_ in combination with (*R*)-**Au4** (Table 1, entry 20) gave lower yields and enantioselectivities than those observed with the dual-matched chiral system.^53^

**Table 1:**
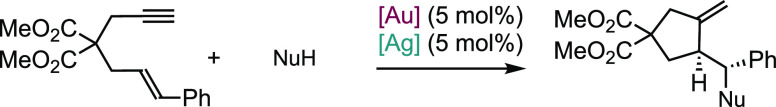
Enantioselective Gold(I)-Catalyzed Addition of Indole to 1,6-Enyne **1a**^a^

entry	[Au]	[Ag]	NuH	time (h)	yield (%)^b^	er^c^
1	**Au1**	(*R*)-**Ag6**	Ind^d^	44	61	78:22
2	**Au2**	(*R*)-**Ag6**	Ind^d^	44	69	54:46
3	**Au3**	(*R*)-**Ag6**	Ind^d^	44	6	50:50
4	**Au4**	(*R*)-**Ag6**	Ind^d^	44	82	95:5
5	**Au5**	(*R*)-**Ag6**	Ind^d^	44	74	60:40
6	**Au6**	(*R*)-**Ag6**	Ind^d^	44	39	54:46
7	**Au4**	(*S*)-**Ag6**	Ind^d^	44	61	61.5:38.5
8	**Au4**	(*R*)-**Ag6**	EtOH^d^	14	87	98:2
9	**Au4**	(*S*)-**Ag6**	EtOH^d^	14	85	57:43
10	**Au1**	(*R*)-**Ag6**	EtOH^d^	14	67	91:9
11	**Au1**	(*S*)-**Ag6**	EtOH^d^	14	53	47:53
12	**Au1**	(*R*)-**Ag1**	Ind^e^	14	82	91:9
13	**Au4**	(*R*)-**Ag1**	Ind^e^	14	93	>99:1
14	**Au4**	(*R*)-**Ag6**	Ind^e^	14	98	97.5:2.5
15	**Au4**	(*R*)-**Ag8**	Ind^e^	14	90	95:5
16	**Au9**	(*R*)-**Ag6**	Ind^e^	18	78	94:6
17	**Au4**		Ind^e^	24	0	
18		(*R*)-**Ag1**	Ind^e^	24	0	
19	**Au12**	(*R*)-**Ag1**	Ind^e^	24	18	52:48
20	**Au4**	AgSbF_6_	Ind^e^	24	76	92.5:7.5

a Reactions carried out under Ar or N_2_ at a 0.05 mmol scale, at 27 °C with (*R*)-configured Au(I) complexes.

b Yields determined by ^1^H NMR using dodecane as internal standard.

c *er* determined by supercritical fluid chromatography (SFC) using a chiral stationary phase.

d Toluene (0.1 M) was used as solvent.

e 1,4-dioxane (0.1 M) was used as solvent. NuH = nucleophile. Ind = Indole.

It is interesting that, whereas in our system the matched pair is achieved with (*R*)-Au cation and (*R*)-phosphoramidate anion, in systems based on two BINOL-based cation/anion ion pairs, the matched catalyst resulted from the *(R*)/(S) ion combination.^17,24,26^

We examined the scope of the reaction using the optimal combination (*R*)-**Au4** and (*R*)-**Ag1** (Scheme 1). The reactions of 1,6-enyne **1a** were performed at 2 mol% catalyst loading in a 0.100 mmol scale, and two conditions were used depending on the nature of the nucleophile and the reactivity of the enyne: conditions *A* using 1,4-dioxane at 27 °C or conditions *B* using toluene at 27, 0, or −10 °C. Substituents in the 1, 2, and 5-positions of indole were well tolerated to give adducts **2b**–**e**. Electron-rich arenes, such as 1,3,5-trimethoxybenzne, 1,3-dimethoxybenzene, and *N,N*-dimethylaniline, led to **2f**–**h** in excellent yields and enantioselectivities. Similarly, excellent results were also obtained with heteroatom-centered nucleophiles, such as anilines; a carbamate; alcohols; and water to give **2i**–**r** in excellent yields and enantioselectivities.

**Scheme 1:**
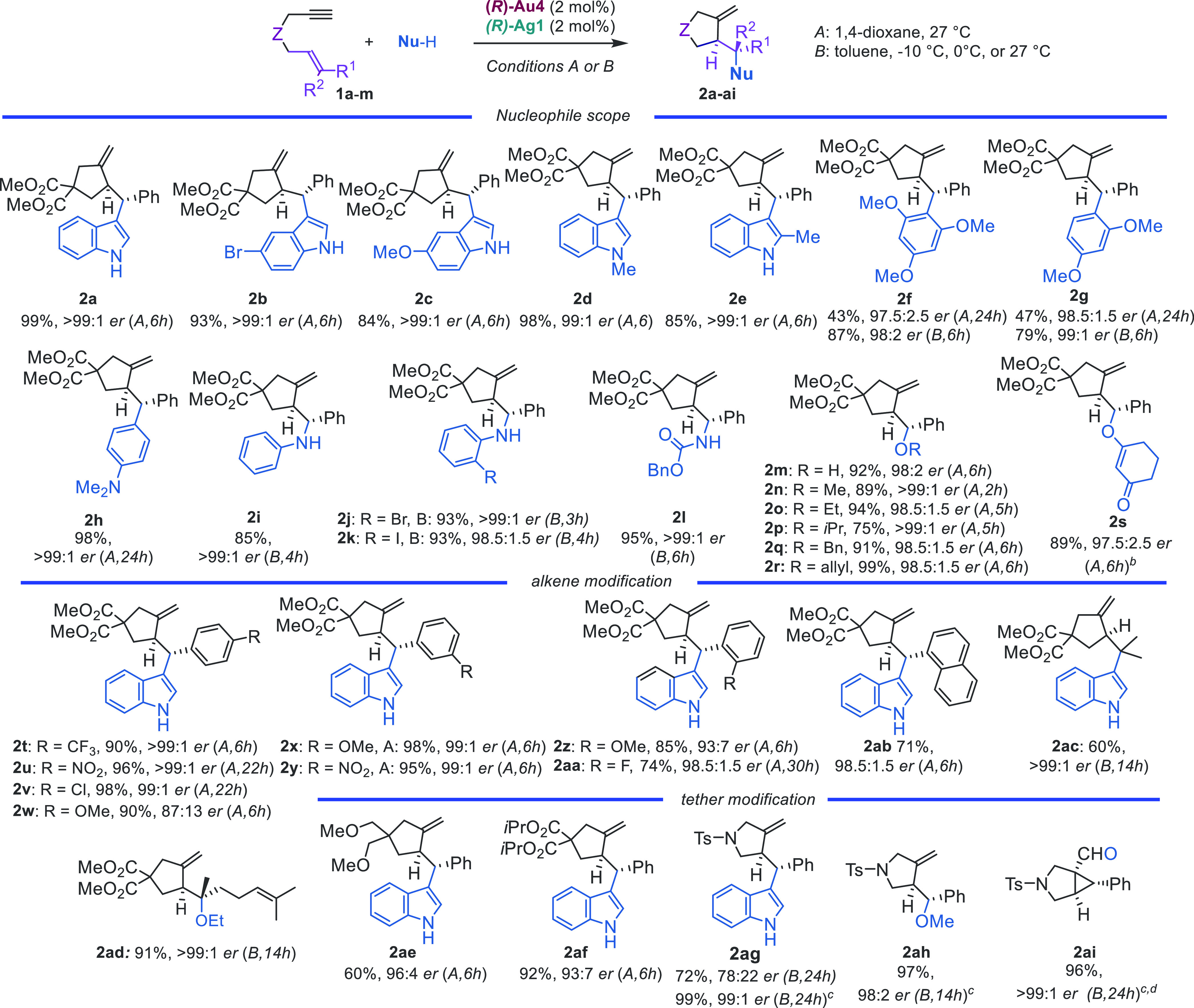
Enantioselective Addition of *O*-, *N*-, and *C*-Nucleophiles to 1,6-Enynes Reaction performed under Ar or N_2_ in anhydrous solvent (0.1 M). Yields given for isolated material after purification; *er* values were determined by HPLC or SFC on chiral stationary phase. Products were obtained as single diastereomers. Reaction times are in parentheses. 1,3-Cyclohexandione (2.0 equiv) was used as nucleophile. Reaction carried out using (*R*)-**Au7** and (*R*)-**Ag5** at a 3 mol% catalyst loading. Diphenylsulfoxide (1.5 equiv) was used as nucleophile.

1,6-Enynes with different substitutions at the alkene also gave the corresponding adducts **2t**–**ad** in good to excellent yields and high enantioselectivities, except for those with a *para*- or *ortho*-anisyl group, which gave **2w** and **2z** in 87:13 and 93:7 *er* (Scheme 1). The absolute configurations of **2v** and **2ai** were determined to be the (*S*,*S*) and (*R*,*S*) by X-ray diffraction.^54^ Changing the malonate to a dimethyl ether tether favored the formation of the cycloisomerization product, thereby leading to **2ae** in moderate yield. However, increasing the size of the ester from methyl to isopropyl reduced the enantioselectivity to a 93:7 *er* in **2af**, which suggests that the tether plays an important role in the folding of the 1,6-enyne in the chiral pocket of the catalyst.

Changing the malonate tether in the 1,6-enyne to a *N*-tosyl led to a decrease in the enantioselectivity, which provided **2ag** in 78:22 *er*. (Scheme 1) However, using (*R*)-**Au7** together with (*R*)-**Ag5** gave adducts **2ag** and **2ah**, as well as aldehyde **2ai**, which resulted from the oxidation of the gold(I) carbene intermediate with diphenylsulfoxide,^55^ in 98:2 to >99:1 *er*.

To further demonstrate the utility of this enantioselective addition of nucleophiles to 1,6-enynes, selected transformations into more complex products were performed (Scheme 2). Thus, the ring-closing metathesis of **2r** with the second generation Grubbs catalyst^56^ gave bicyclic derivative **3a** with no erosion on the enantioselectivity. The *ortho*-iodoaniline addition product **2k** underwent an intramolecular Heck reaction with the concomitant formation of a new stereocenter to afford 2,3,3a,9b-tetrahydro-1*H*-cyclopenta[*c*]quinoline **3b**.^50^ Finally, the allylic position in pyrrolidine **2ah** was oxidized with NaClO_2_ and *N*-hydroxyphthalimide to give lactam **3c** with 98.5:1.5 *er*.

**Scheme 2:**
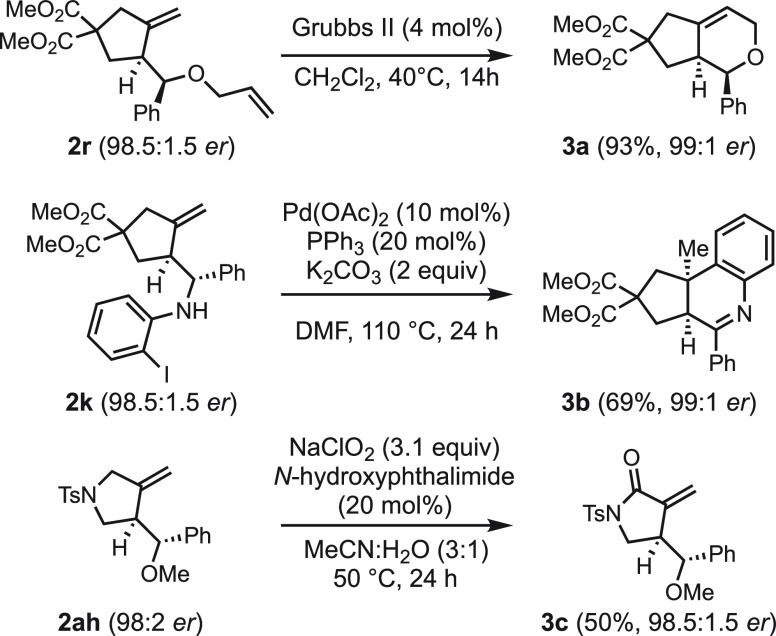
Product Derivatization

DFT studies were conducted [B3LYP-D3/6-31G(d) (C, H, P, O, F, N, S)//B3LYP-D3/6-311G(d,p) and SDD (Au), PCM = toluene]^57^ to gain insight into the role of the urea and the possible secondary interactions involved in the enantioselective cyclization step using chiral (*R*)-**Au4** and chiral counterion from (*R*)-**Ag6**, which provided **2a** in 95:5 *er*.

Our computations predicted a Curtin–Hammett scenario (Scheme 3a), where the two orientations of the alkenes (**int1a** and **int3a**) are in equilibrium. Although **int3a** is 4.9 kcal/mol more stable than **int1a**, the major product arises from the latter via **TS**_**int1a-2a**_, which is lower in energy than **TS**_**int3a-4a**_, thereby giving rise to product **2a** with an *S* configuration by reaction through the *Re* face of the alkene, which agrees with the experimental results.

**Scheme 3:**
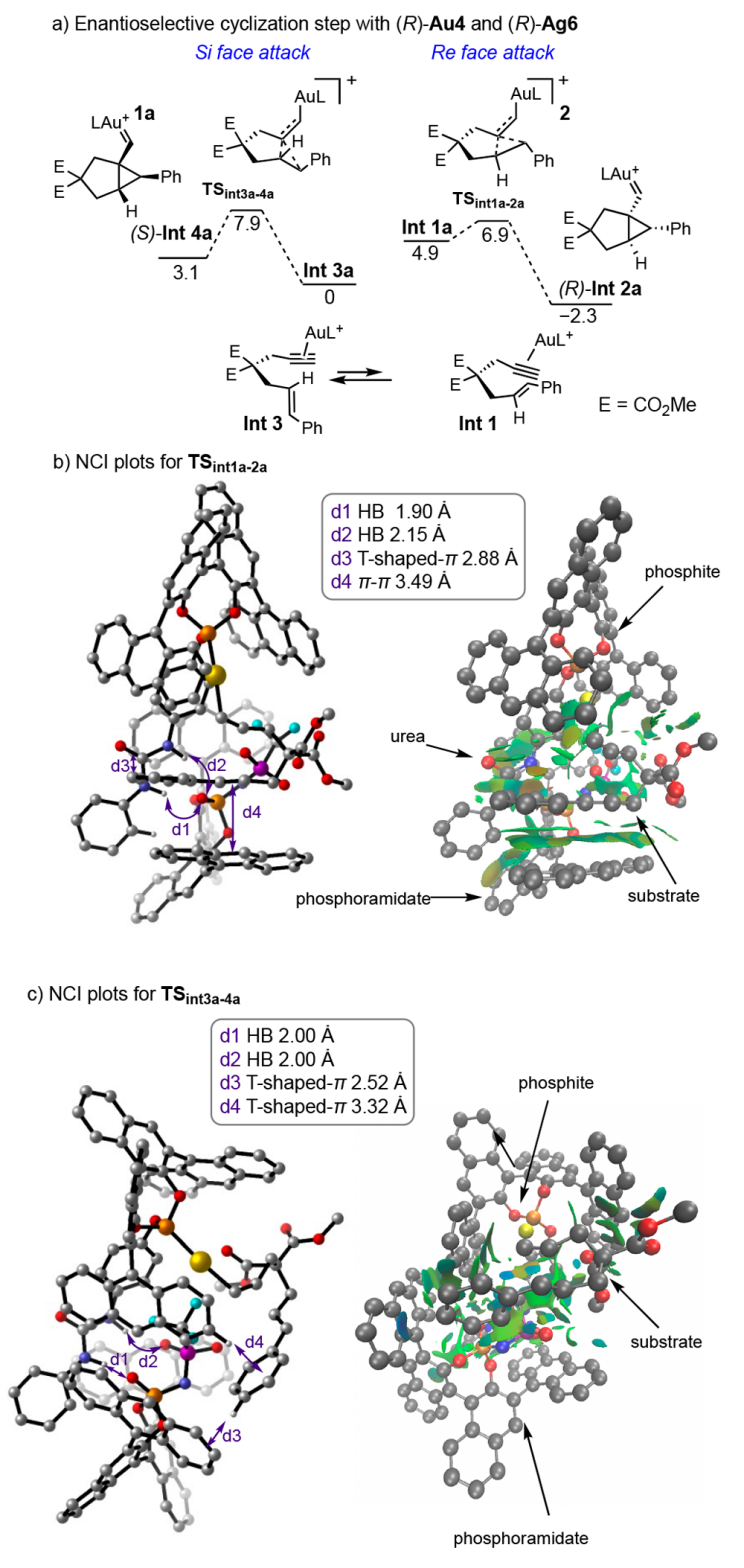
DFT Calculations for the Enantiodetermining Step Two most relevant pathways for the enantiodetermining cyclization step of **1a** with (*R*)-**Au4** and (*R*)-**Ag6**. NCI plots and CYLview representations for **TS_int1a-2a_** and **TS_int3a-4a_**. Hydrogens are omitted for clarity, except relevant ones. Strong attractive interactions are blue, weak attractive interactions are green, and strong repulsive interactions are red. Color code: P, orange; Au, yellow; F, cyan; O, red; N, blue; S, purple; C, gray; and H, white. Energy values are in kcal/mol. HB = hydrogen bond.

Noncovalent interaction (NCI) plots were performed to visualize the noncovalent interactions in the two possible transition states (Scheme 3b,c) which revealed that the H-bond interactions from the urea and phosphoramidate group act as anchors of the two parts and favor the additional interactions that stabilize the transition state **TS**_**int1a-2a**_. Apart from the strong H-bonding interactions, T-shaped-π interactions between the *ortho*-C–H of the *N*-phenyl urea and the π-system of the BINOL counterion were observed. A strong extended attractive sandwich π–π interaction between the π-system of the cinnamyl alkene of the substrate and one anthracenyl group of the chiral counterion were observed in **TS**_**int1a-2a**_. However, for **TS**_**int3a-4a**_, a T-shaped π-attractive interaction was found between the C–H in *para* position to the alkene in the aryl ring of the substrate and the anthracene of the counterion, which helped to stabilize the transition state.

Finally, other reactions were tested by combining BINOL-derived gold(I) catalysts and different silver(I) salts as chloride scavengers, but the optimal ion pair combination could not be found. We discovered that 1,6-enynes bearing internal aryl-substituted alkynes led to formal products of [4 + 2] cycloaddition^31^ with poor yields and moderate enantioselectivities, most likely because of the small pocket generated by the ion pair. However, when using chiral gold(I) complexes and small achiral counterions, such as AgSbF_6_, promising enantioselectivities were found (95:5 *er*). We also tested the [2 + 2] cycloaddition of phenylacetylene with alkenes,^58^ but no promising combination was observed.

In summary, we have developed the enantioselective nucleophilic addition of hetero- and carbonucleophiles that proceeds with the broadest scope and highest enantioselectivity using a chiral catalyst with a chirally matched gold(I) phosphitourea and phosphoramidate, which can be readily prepared from commercially available BINOLs. A model for the enantioinduction has been proposed on the basis of DFT calculations and NCI plots where the urea in the chiral gold(I) cation anchors the chiral counterion in close proximity creating a chiral pocket to fold the unsaturated substrate.

## Supporting Information Available

Optimization tables, procedures, characterization, NMR spectra, SFC and HPLC traces, DFT computations, and crystallographic data (PDF)

## Accession Codes

CCDC 2266405–2266408 contain the supplementary crystallographic data for this paper. These data can be obtained free of charge via www.ccdc.cam.ac.uk/data_request/cif, or by emailing data_request@ccdc.cam.ac.uk, or by contacting The Cambridge Crystallographic Data Centre, 12 Union Road, Cambridge CB2 1EZ, UK; fax: + 44 1223 336033.
