# Supplementary material for: Hydrogen-Bonded Matched Ion Pair Gold(I) Catalysis
Source: ACS Catal. 2023 Jul 20;13(15):10217–23. doi: 10.1021/acscatal.3c02638 (PMC10407850; doi:10.1021/acscatal.3c02638)
Supplement: Supplementary file 1 — cs3c02638_si_001.pdf [file cs3c02638_si_001.pdf]

Hydrogen-Bonded Matched Ion Pair Gold(I) Catalysis

Àlex Martí, Gala Ogalla and Antonio M. Echavarren*

Institute of Chemical Research of Catalonia (ICIQ), Barcelona Institute of Science and Technology, Av. Països Catalans 16, 43007 Tarragona, Spain

Departament de Química i Analítica, Universitat Rovira i Virgili, C/ Marcel·lí Domingo s/n, 43007 Tarragona, Spain

Corresponding Author

Prof. A. M. Echavarren

*E-mail: aechavarren@iciq.es

Table of Contents

1. General remarks 3

2. Synthesis and characterization of gold and silver catalysts 3

2.1 General remarks 4

2.2 Procedure and characterization 5

3. Synthesis of substrates 19

4. Nucleophile addition to 1,6-enynes 28

4.1 Optimization 28

4.2 Procedures and characterization 35

4.3 Assigment of Absolute Configuration 56

5. DFT details 57

5.1 5.1 Discussion 58

5.2 5.2 NCI plots 59

6. X-Ray structures 61

7. NMR spectra 69

8. HPLC and SFC traces 173

9. References 212

1. General remarks

Unless otherwise stated, reactions were performed with magnetic stirring under nitrogen or argon atmosphere using standard Schlenk techniques, and room temperature (rt) indicates a temperature of approximately 23 ºC. Compound names were generated using ChemDraw. Chemicals were obtained from commercial suppliers and used as received unless stated otherwise. Anhydrous solvents were dried by passing through an activated alumina column on a PureSolv solvent purification system, taken from commercial bottles equipped with septa and molecular sieves or by drying them on activated 3Å molecular sieves for at least 48h. Solutions were evaporated using a Büchi rotary evaporator under reduced pressure.

Analytical thin-layer chromatography (TLC) was carried out using aluminum sheets coated with 0.2 mm of silica gel (fluorescent-treated Merck Kieselgel 60 F254). Visualization was accomplished under UV light at 254 nm and by staining with an alkaline aqueous potassium permanganate solution, ninhydrin or vanillin staining dips. Flash column chromatography (FCC) was carried out using PanReac Silica Gel 60 (40–63 μm) or employing the automated flash column chromatographer CombiFlash Companion.

NMR spectra were recorded at 298 K (unless otherwise stated) on BrukerAvance Ultrashield NMR spectrometers operating at ^{1}H resonances of 300, 400 or 500 MHz (in the latter case, with cryoprobe for enhanced sensitivity). Proton and carbon chemical shifts (δ) are given in parts per million (ppm) relative to tetramethylsilane, with the solvent resonance as internal standard.[1] ^{1}H NMR spectra are reported as follows: chemical shift (multiplicity, coupling constant, number of protons). The following abbreviations are used: s = singlet, d = doublet, t = triplet, q = quartet, quint = quintet, hept = heptet, m = multiplet, br = broad signal, app = apparent. Coupling constants (*J*) are given in Hertz. ^{13}C, ^{31}P and ^{19}F NMR spectra were always acquired with proton decoupling.

High-resolution mass spectra (HRMS) were recorded by ICIQ mass spectrometry staff on Bruker MicroTOF Focus or Maxis impact spectrometers equipped with an ESI or APCI source. Melting points were measured using a Mettler Toledo MP70 Melting Point apparatus.

2. Synthesis and characterization of gold and silver catalysts

Chiral gold complexes (**Au1–Au11**) and were prepared in a two-step sequence starting from commercially available chiral binaphtols precursors with the desired 3,3’ substitution pattern (**A1–A4**) or from biphenol cavitand (**B**).

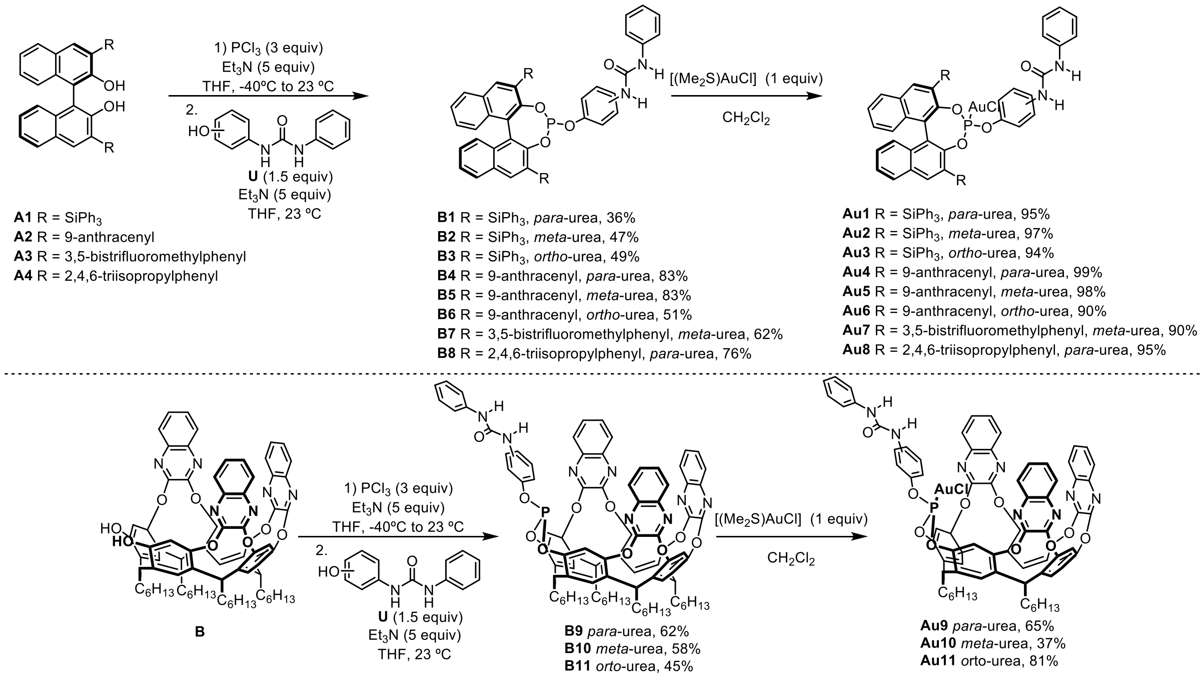

Figure S1 Synthetic route for the preparation of phsophitourea gold(I) complexes

Chiral silver salts (**Ag0**–**Ag10**) were prepared starting from the corresponding diols following literature procedures.[2]

2.1 General remarks

(*R*)-3,3′-Bis(triphenylsilyl)-1,1′-bi-2-naphthol [(*R*)-**A1**, CAS 111822-69-6] and (*R*)-3,3′-Bis(3,5-bis(trifluoromethyl)phenyl)-1,1′-bi-2-naphthol [(*R*)-**A3**, CAS 756491-54-0] were bought from commercial suppliers and used as received.

(*R*)-3,3′-Di-9-anthracenyl-1,1′-bi-2-naphthol [(*R*)-**A2**, CAS 361342-49-6] and (*R*)-3,3′-Bis(2,4,6-triisopropylphenyl)-1,1′-bi-2-naphthol [(*R*)-**A4**, CAS 247123-09-7] are also commercially available, but were instead synthesized on larger scale following literature procedures.[2]

2.2 Procedure and characterization

General procedure GP1: phosphite formation

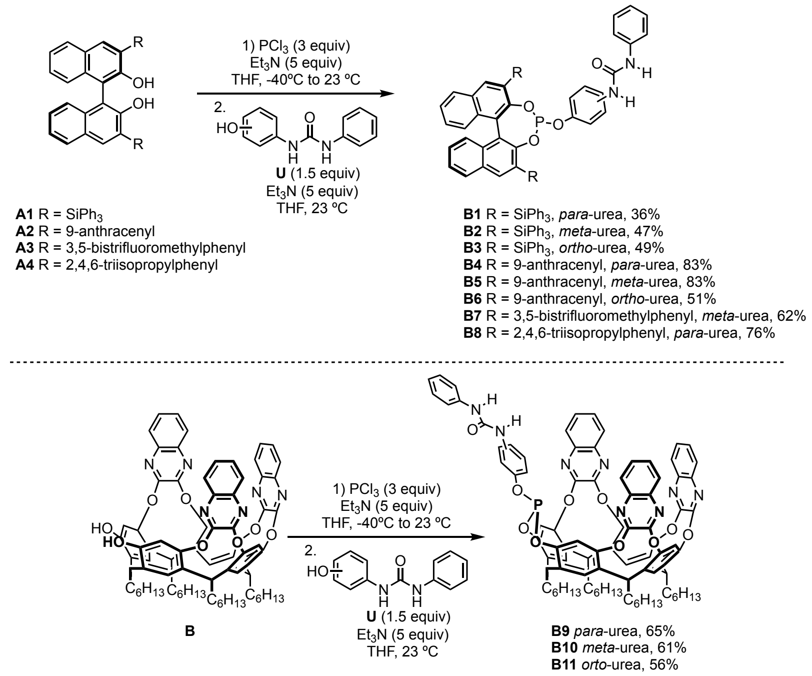

Following a modified literature procedure,[3] under Ar, a flame-dried Schlenk tube equipped with a stir bar was charged with the desired binaphthol A or B. Anhydrous THF was added and the solid was dissolved before cooling it to -40 °C. A solution of PCl_3 (3 equiv) in THF (0.12 M final concentration) was then added dropwise. After stirring for 10 min, anhydrous Et_3N (5 equiv) was added at the same temperature. The mixture was allowed to warm up to room temperature and stirred for 2 h. The reaction was moved inside the glovebox where it was filtered through a celite pad (rinsing with THF). The filtrate was concentrated under reduced pressure, treated with toluene (1 mL) and evaporated. The obtained crude was dissolved in anhydrous THF (0.04 M) and Et_3N (5 equiv) was added followed by the corresponding hydroxyurea U (2 equiv). The resulting mixture was left stirring 18 h at room temperature. Next day, the volatiles were removed under vacuum and the crude was purified by FCC (CyH:AcOEt or pent:AcOEt solvent mixtures) providing the desired phosphite ligand (**B1**–**B11**).
Note: phosphites were found to readily oxidize under air. FCC were carried out using N_2. Compounds had to be stored in the glovebox or quickly coordinated to gold.

General procedure GP2: Gold complexation

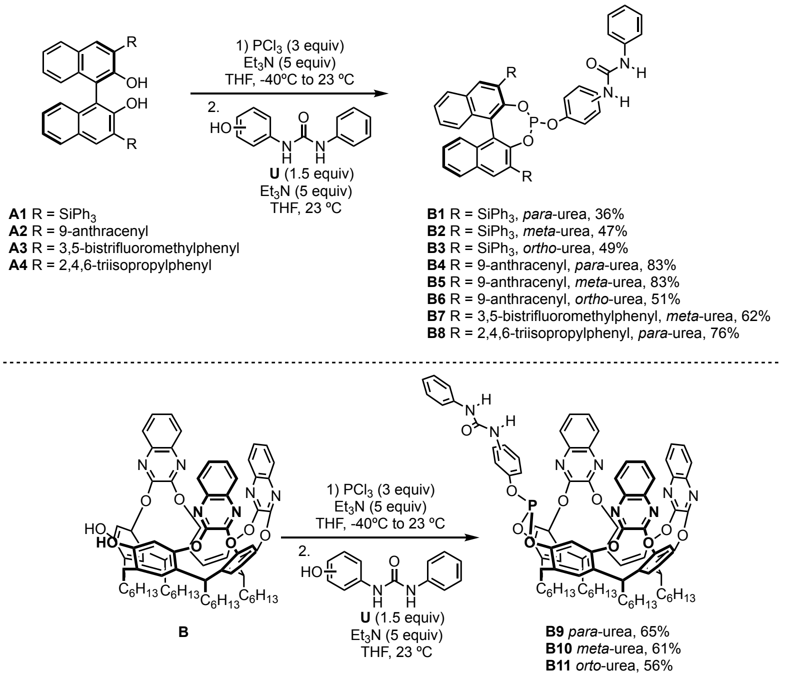

Under air, to a stirred solution or suspension of the corresponding phosphite **B** in CH_2Cl_2, [$(Me_2S)AuCl$] (1.0 equiv) was added, followed by additional CH_2Cl_2 (final concentration 0.05 M). The resulting solution was stirred in the dark at 23 ºC for 0.5–6 h. The solvent was evaporated under reduced pressure and the crude product was purified by FCC (CyH:AcOEt or pent:AcOEt solvent mixtures) yielding complex **Au**.

1-(4-Hydroxyphenyl)-3-phenylurea (U1)

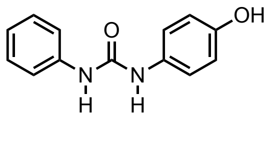

To an oven-dried 50 mL flask was added 4-aminophenol (0.88 mL, 9.16 mmol) and dissolved in anhydrous MeCN (18 mL). The system was cooled to 0 ºC with an ice/water bath and isocyanatobenzene (1.0 mL, 9.16 mmol, 1.0 equiv) was added dropwise and the mixture was left stirring at the same temperature for 2h. The solvent was evaporated obtaining hidroxyurea **U1** as a white solid (2.08g, 9.11 mmol, 99% yield) which was used as it is without further purification.

Spectroscopic data matched those reported in the literature.[4]

^{1}H NMR (500 MHz, $(CD_3)_2SO$) δ 9.06 (s, 1H), 8.52 (s, 1H), 8.31 (s, 1H), 7.43 (dt, J = 8.7, 1.6 Hz, 2H), 7.31 – 7.24 (m, 2H), 7.24 – 7.19 (m, 2H), 6.94 (tt, J = 7.5, 1.1 Hz, 1H), 6.75 – 6.63 (m, 2H).

1-(3-Hydroxyphenyl)-3-phenylurea (U2)

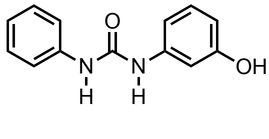

To an oven-dried 50 mL flask was added 3-aminophenol (0.500 g, 4.58 mmol) and dissolved in anhydrous MeCN (9 mL). The system was cooled to 0 ºC with an ice/water bath and isocyanatobenzene (0.50 mL, 4.58 mmol, 1.0 equiv)

was added dropwise. The mixture was left stirring at the same temperature for 1h. The solvent was evaporated and the crude was purified by FCC (CyH:AcOEt 70:30 to 50:50) obtaining hidroxyurea **U2** as a white solid (0.982 g, 4.30 mmol, 94% yield).
Spectroscopic data matched those reported in the literature.[4]
^{1}H NMR (500 MHz, $(CD_3)_2SO$) δ 9.31 (s, 1H), 8.56 (d, J = 18.3 Hz, 2H), 7.44 (dd, J = 8.6, 1.1 Hz, 2H), 7.30 – 7.24 (m, 2H), 7.07 – 7.00 (m, 2H), 6.99 – 6.91 (m, 1H), 6.79 (s, 1H), 6.37 (ddd, J = 8.1, 2.3, 0.8 Hz, 1H).

1-(2-Hydroxyphenyl)-3-phenylurea (U3)

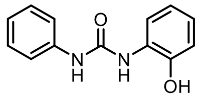

To an oven-dried 50 mL flask was added 2-aminophenol (0.500 g, 4.58 mmol) and dissolved in anhydrous MeCN (9 mL). The system was cooled to 0 ºC with an ice/water bath and isocyanatobenzene (0.50 mL, 4.58 mmol, 1.0 equiv) was added dropwise. The mixture was left stirring at the same temperature for 1h. The solvent was evaporated and the crude was purified by FCC (CyH:AcOEt 70:30 to 50:50) obtaining hidroxyurea **U3** as a white solid (0.963 g, 4.22 mmol, 92% yield).
Spectroscopic data matched those reported in the literature.[5]
^{1}H NMR (500 MHz, $(CD_3)_2SO$) δ 9.93 (s, 1H), 9.29 (s, 1H), 8.16 (s, 1H), 8.03 (dd, *J* = 7.9, 1.7 Hz, 1H), 7.44 (dt, *J* = 8.7, 1.6 Hz, 2H), 7.31 – 7.23 (m, 2H), 6.95 (tt, *J* = 7.5, 1.1 Hz, 1H), 6.84 (dd, *J* = 7.8, 1.6 Hz, 1H), 6.76 (dtd, *J* = 23.8, 7.4, 1.7 Hz, 2H).
^{13}C NMR (126 MHz, $(CD_3)_2SO$) δ 153.0, 146.1, 140.4, 129.3, 128.3, 122.2, 122.1, 119.6, 119.0, 118.3, 114.8.

(*R*)-B1

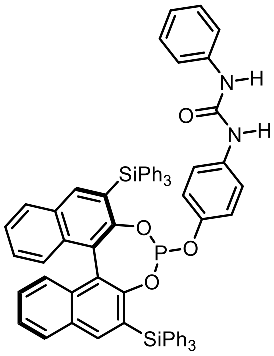

Prepared following general procedure GP1, using binaphthol **(*R*)-A1** (200 mg, 0.250 mmol) and hidroxyurea **U1** (114 mg, 0.500 mmol, 2.0 equiv). **(*R*)-B1** was obtained as a white solid) (94 mg, 0.089 mmol, 36% yield) after FCC (CyH:AcOEt dry load silica, 90:10 to 80:20).
M.p. 212 °C.
^{1}H NMR (500 MHz, CD_2Cl_2) δ 8.10 (s, 1H), 7.96 (s, 1H), 7.83 (d, *J* = 8.2 Hz, 1H), 7.78 (d, *J* = 8.2 Hz, 1H), 7.57 (ddd, *J* = 20.0, 8.0, 1.5 Hz, 12H), 7.46 – 7.19 (m, 28H), 7.09 (tt, *J* = 6.8, 1.7 Hz, 1H), 6.79 – 6.69 (m, 2H), 6.46 (brs, 1H), 6.35 (brs, 1H), 5.79 – 5.68 (m, 2H).
^{13}C NMR (126 MHz, CD_2Cl_2) δ 152.8, 152.5 (d, *J* = 4.4 Hz) 151.9 (d, *J* = 4.4 Hz), 147.3 (d, *J* = 7.4 Hz), 141.1, 141.0, 138.4, 136.7, 136.5, 134.7 (d, *J* = 38.5 Hz) 134.6, 133.7, 133.6, 130.9, 130.4, 129.7, 129.5, 129.2, 128.7 (d, *J* = 6.4 Hz)128.0, 127.8, 127.5, 127.2 (d, *J* = 7.7 Hz)126.8, 126.7, 126.6, 125.1 (d, *J* = 5.7 Hz), 123.8, 123.4 (d, *J* = 4.4 Hz), 122.9 (d, *J* = 4.4 Hz), 121.8, 121.2 (d, *J* = 6.8 Hz), 120.5.
^{31}P NMR (203 MHz, CD_2Cl_2) δ 149.9.

HRMS (ESI +) calculated for $[C_{69}H_{51}N_2NaO_4PSi_2]^{+\cdot}$ $[M+Na]^{+\cdot}$ 1081.3017 m/z; found 1081.3003 m/z.
[α]^{26}D −140.5 (c 0.38, acetone).

(*R*)-B2

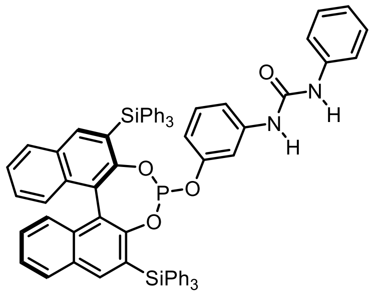

Prepared following general procedure GP1, using binaphthol **(*R*)-A1** (200 mg, 0.250 mmol) and hydroxyurea **U2** (114 mg, 0.500 mmol, 2.0 equiv). **(*R*)-B2** was obtained as a white solid (123 mg, 0.116 mmol, 47% yield) after FCC (CyH:AcOEt dry load silica, 90:10 to 80:20).

M.p. 215 °C.

^{1}H NMR (500 MHz, CD_2Cl_2) δ 8.14 (s, 1H), 7.99 (s, 1H), 7.88 (d, *J* = 8.2 Hz, 1H), 7.81 (d, *J* = 8.1 Hz, 1H), 7.67 – 7.55 (m, 12H), 7.53 – 7.22 (m, 29H),7.16 (t, *J* = 7.1 Hz, 1H), 6.78 (t, *J* = 8.2 Hz, 1H), 6.34 (brs, 1H), 5.57 (brs, 1H), 5.56 – 5.53 (m, 1H), 5.50 – 5.47 (m, 1H).

^{13}C NMR (126 MHz, CD_2Cl_2) δ 152.2 (d, J = 5.0 Hz), 151.7, 151.5 (d, J = 4.1 Hz), 150.7 (d, J = 6.9 Hz), 140.9 (d, J = 21.9 Hz), 138.1 (d, J = 29.0 Hz), 136.4, 136.4, 134.5, 134.2, 134.2, 133.5, 130.7, 130.1, 129.4, 129.2, 129.0, 129.0, 128.5 (d, J = 5.6 Hz), 127.7, 127.5, 126.9 (d, J = 3.6 Hz), 126.6, 126.4, 126.3, 124.8 (d, J = 1.7 Hz), 123.7, 123.1 (d, J = 4.9 Hz), 122.6 (d, J = 3.0 Hz), 120.2, 115.6 (d, J = 7.3 Hz), 115.5, 112.1 (d, J = 6.7 Hz).

^{31}P NMR (203 MHz, CD_2Cl_2) δ 149.9.

HRMS (ESI +) calculated for $[C_{69}H_{52}N_2O_4PSi_2]^{+\cdot}$ $[M+H]^{+\cdot}$ 1059.3198 m/z; found 1059.3184 m/z.

[α]^{26}D −159.2 (c 0.43, acetone).

(*R*)-B3

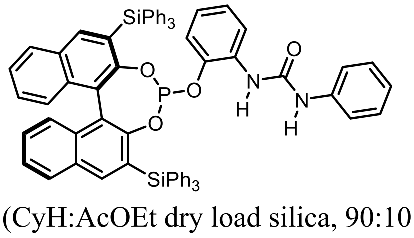

Prepared following general procedure GP1, using binaphthol **(*R*)-A1** (200 mg, 0.250 mmol) and hydroxyurea **U3** (114 mg, 0.500 mmol, 2.0 equiv). (*R*)-**B3** was obtained as a white solid (130 mg, 0.129 mmol, 49% yield, 97% pure according to ^{31}P NMR) after FCC (CyH:AcOEt dry load silica, 90:10 to 80:20).

M.p. 177 °C.

^{1}H NMR (500 MHz, CD_2Cl_2) δ 8.06 (d, *J* = 4.6 Hz, 2H), 7.89 – 7.77 (m, 3H), 7.56 – 7.45 (m, 12H), 7.45 – 7.40 (m, 2H), 7.37 – 7.15 (m, 25H), 7.02 (d, *J* = 7.6 Hz, 2H), 6.97 – 6.85 (m, 2H), 6.52 (td, *J* = 7.9, 1.5 Hz, 1H), 5.92 (s, 1H), 5.82 (d, *J* = 8.0 Hz, 1H).

^{13}C NMR (126 MHz, CD_2Cl_2) δ 152.5 (d, *J* = 5.3 Hz), 151.3, 141.2 (d, *J* = 9.9 Hz), 139.2 (d, *J* = 6.6 Hz), 138.1, 136.4, 136.3, 134.5 (d, *J* = 41.7 Hz), 134.0, 133.4, 131.1, 130.4, 130.3, 129.6 (d, *J* = 16.5 Hz), 128.9 (d, *J* = 2.1 Hz), 128.0, 127.7, 127.4, 127.3, 126.6 (d, *J* = 5.1 Hz), 125.4 (d, *J* = 11.4 Hz), 123.8, 123.4, 122.7, 121.6, 120.6, 119.6, 119.5.

^{31}P NMR (162 MHz, $CDCl_3$) δ 147.5.

HRMS (ESI +) calculated for $[C_{69}H_{52}N_2O_4PSi_2]^{+\cdot}$ $[M+H]^{+\cdot}$ 1059.3198 m/z; found 1059.3178 m/z.

$[\alpha]^{26}_{D}$ −66.5 (c 1.01, acetone).

(*R*)-B4

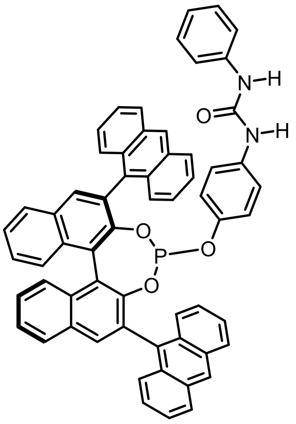

Prepared following general procedure GP1, using binaphthol **(*R*)-A2** (300 mg, 0.470 mmol) and hydroxyurea **U1** (241 mg, 0.940 mmol, 2.0 equiv). **(*R*)-B4** was obtained as a beige solid (348 mg, 0.349 mmol, 83% yield) after FCC (CyH:AcOEt dry load silica, 90:10).

M.p. 258 °C.

^{1}H NMR (500 MHz, CD_2Cl_2) δ 8.54 (d, *J* = 13.9 Hz, 2H), 8.21 (s, 1H), 8.16 – 8.03 (m, 5H), 7.97 (dd, *J* = 15.4, 8.5 Hz, 2H), 7.88 – 7.78 (m, 4H), 7.77 – 7.66 (m, 3H), 7.66 – 7.50 (m, 3H), 7.50 – 7.19 (m, 11H), 7.15 – 6.99 (m, 2H), 6.44 (d, *J* = 8.5 Hz, 3H), 6.27 (brs, 1H), 4.75 (d, *J* = 8.4 Hz, 2H.

^{13}C NMR (126 MHz, CD_2Cl_2) δ 153.1, 147.8, 147.2 (d, *J* = 10.9 Hz), 138.6, 133.8, 133.6 (d, *J* = 9.6 Hz), 133.2, 132.7, 132.1 (d, *J* = 4.9 Hz), 131.9 (d, *J* = 9.1 Hz), 131.8, 131.6, 131.5, 131.4, 131.3, 130.9 (d, *J* = 27.8 Hz), 129.4, 129.1, 128.9 (d, *J* = 5.6 Hz), 128.7 (d, *J* = 6.8 Hz), 128.4, 127.75, 127.68, 127.6, 127.2, 127.1, 127.04 126.9, 126.4 (d, *J* = 10.2 Hz), 126.1, 125.9, 125.7 (d, *J* = 7.3 Hz), 125.5 (d, *J* = 11.2 Hz), 125.4, 124.1, 124.0 (d, *J* = 2.8 Hz), 122.3, 120.8, 119.3 (d, *J* = 9.1 Hz).

^{31}P NMR (203 MHz, CD_2Cl_2) δ 143.7.

HRMS (ESI +) calculated for $[C_{61}H_{39}N_2NaO_4P]^{+\cdot}$ $[M+Na]^{+\cdot}$ 917.2540 m/z; found 917.2559 m/z.

$[\alpha]^{26}_{D}$ −56.21 (c 0.49, acetone).

(*R*)-B5

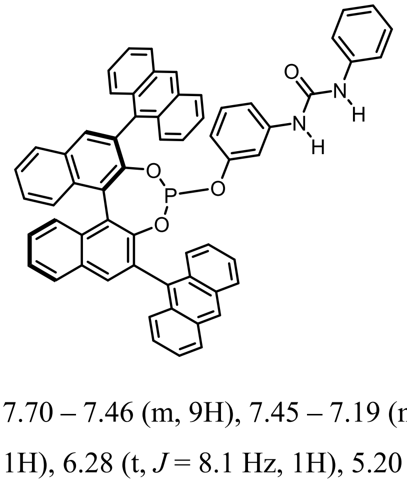

Prepared following general procedure GP1, using binaphthol **(*R*)-A2** (150 mg, 0.235 mmol) and hydroxyurea **U2** (107 mg, 0.470 mmol, 2.0 equiv). **(*R*)-B5** was obtained as a beige solid (175 mg, 0.196 mmol, 83% yield) after FCC (CyH:AcOEt dry load silica, 90:10).

M.p. 254–256 °C.

^{1}H NMR (500 MHz, $(CD_3)_2CO$) δ 8.61 (s, 1H), 8.49 (s, 1H), 8.24 – 8.09 (m, 5H), 8.06 – 7.92 (m, 3H), 7.78 (dt, *J* = 32.2, 10.5 Hz, 5H), 7.70 – 7.46 (m, 9H), 7.45 – 7.19 (m, 8H), 7.14 (t, *J* = 7.5 Hz, 1H), 7.05 – 6.96 (m, 2H), 6.84 – 6.77 (m, 1H), 6.28 (t, *J* = 8.1 Hz, 1H), 5.20 (s, 1H), 4.60 (dd, *J* = 8.1, 2.0 Hz, 1H).

^{13}C NMR (126 MHz, $(CD_3)_2CO$) δ 152.6, 151.2 (d, *J* = 11.6 Hz), 148.3 (d, *J* = 5.3 Hz), 147.1, 140.9 (d, *J* = 14.7 Hz), 134.1 (d, *J* = 7.7 Hz), 133.9, 133.6, 132.7, 132.6, 132.42, 132.4, 132.3, 132.1, 132.1 (d, *J* = 12.5 Hz), 131.8, 131.7, 131.5, 131.1, 129.7, 129.7, 129.53, 129.48, 129.4, 129.2, 128.9, 128.3 (d, *J* =

8.9 Hz), 127.9 (d, *J* = 1.9 Hz), 127.8 (d, *J* = 11.4 Hz), 127.4, 127.3 (d, *J* = 3.8 Hz), 127.2, 127.0, 126.9, 124.5, 123.0, 119.5, 114.7, 112.3 (d, *J* = 11.4 Hz), 109.9 (d, *J* = 8.9 Hz).

^{31}P NMR (203 MHz, $(CD_3)_2CO$) δ 145.2.

HRMS (ESI +) calculated for $[C_{61}H_{39}N_2NaO_4P]^{+\cdot}$ $[M+Na]^{+\cdot}$ 917.2540 m/z; found 917.2545 m/z.

$[\alpha]^{24.6}_D$ −12.6 (c 1.18, acetone).

(*R*)-B6

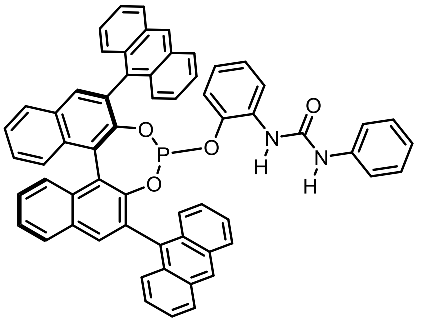

Prepared following general procedure GP1, using binaphthol **(*R*)-A2** (100 mg, 0.157 mmol) and hydroxyurea **U3** (71 mg, 0.313 mmol, 2.0 equiv). **(*R*)-B6** was obtained as a beige solid (71 mg, 0.079 mmol, 51% yield) after FCC (CyH:AcOEt dry load silica, 90:10).

M.p. 190–194 °C.

^{1}H NMR (500 MHz, $(CD_3)_2CO$) δ 8.46 (d, *J* = 6.7 Hz, 2H), 8.25 – 8.18 (m, 3H), 8.16 (s, 1H), 8.13 (d, *J* = 8.5 Hz, 1H), 7.92 (dd, *J* = 8.4, 3.2 Hz, 2H), 7.83 (dd, *J* = 21.7, 8.7 Hz, 5H), 7.71 (td, *J* = 9.5, 7.7, 2.3 Hz, 2H), 7.68 – 7.55 (m, 5H), 7.55 – 7.47 (m, 2H), 7.46 – 7.37 (m, 2H), 7.35 – 7.20 (m, 7H), 7.02 – 6.94 (m, 3H), 6.56 – 6.49 (m, 1H), 6.34 (s, 1H), 5.84 (td, *J* = 7.8, 1.4 Hz, 1H), 4.58 (d, *J* = 8.0 Hz, 1H).

^{13}C NMR (126 MHz, $(CD_3)_2CO$) δ 152.1, 148.1 (d, *J* = 4.9 Hz), 146.7 (d, *J* = 1.8 Hz), 140.7, 139.1 (d, *J* = 11.3 Hz), 134.5 (d, *J* = 6.0 Hz), 133.8, 133.6, 132.8, 132.7, 132.5, 132.4, 132.3, 132.26, 132.10, 132.04, 131.99, 131.84 (d, *J* = 3.4 Hz), 131.60, 131.42, 131.14, 130.95 (d, *J* = 2.9 Hz), 129.70 (d, *J* = 11.8 Hz), 129.5, 129.4, 129.3 (d, *J* = 3.6 Hz), 128.9, 128.5, 128.2, 128.0 (d, *J* = 7.0 Hz), 127.9 (d, *J* = 7.1 Hz), 127.5, 127.3, 127.24, 127.18, 126.9, 126.8 (d, *J* = 10.3 Hz), 126.53 (d, *J* = 5.8 Hz), 126.48, 126.1, 126.02, 125.98, 125.6, 124.5 (d, *J* = 2.3 Hz), 124.2, 122.9, 121.2, 119.5, 119.4, 117.6 (d, *J* = 16.7 Hz).

^{31}P NMR (203 MHz, $(CD_3)_2CO$) δ 144.6.

HRMS (ESI +) calculated for $[C_{61}H_{40}N_2O_4P]^{+\cdot}$ $[M+H]^{+\cdot}$ 895.2720 m/z; found 895.2708 m/z.

$[\alpha]^{26}_D$ +150.7 (c 0.45, acetone).

(*R*)-B7

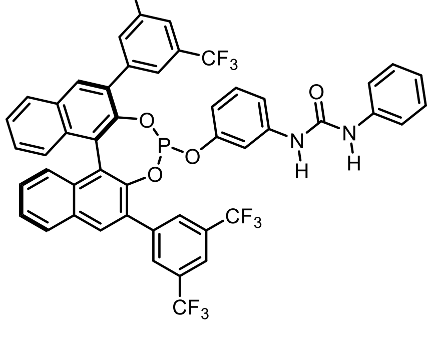

Prepared following general procedure GP1, using binaphthol **(*R*)-A3** (100 mg, 0.141 mmol) and hydroxyurea **U2** (64 mg, 0.248 mmol, 2.0 equiv). **(*R*)-B6** was obtained as a white solid (84 mg, 0.087 mmol, 62% yield) after FCC (CyH:AcOEt dry load silica, 90:10).

M.p. 250 °C.

^{1}**H NMR** (500 MHz CD_2Cl_2) δ 8.24 (d, *J* = 1.7 Hz, 2H), 8.17 (s, 1H), 8.12 (d, *J* = 1.7 Hz, 2H), 8.11 – 8.08 (m, 2H), 8.06 (d, *J* = 8.2 Hz, 1H), 7.92 (s, 1H), 7.83 (s, 1H), 7.62 – 7.54 (m, 2H), 7.51 – 7.47 (m, 1H), 7.46 – 7.38 (m, 3H), 7.35 – 7.28 (m, 4H), 7.13 – 7.06 (m, 1H), 7.00 (dd, *J* = 7.8, 2.4 Hz, 1H), 6.91 (td, *J* = 8.2, 1.8 Hz, 1H), 6.56 – 6.44 (m, 1H), 6.41 (d, *J* = 2.4 Hz, 1H), 6.37 (d, *J* = 25.7 Hz, 1H), 5.98 (dd, *J* = 8.1, 2.3 Hz, 1H).

^{13}**C NMR** (126 MHz, CD_2Cl_2) δ 152.0, 151.6 (d, *J* = 9.7 Hz), 144.4 (d, *J* = 3.8 Hz), 143.7 (d, *J* = 2.8 Hz), 139.9, 139.7, 139.3, 138.0, 133.0, 132.6, 131.9, 131.8, 131.7, 131.6, 131.5, 131.44, 131.39, 131.33, 131.30, 131.2, 131.13, 131.07, 130.91, 130.87, 130.4 – 129.9 (m), 129.7, 129.1, 128.8 (d, *J* = 4.7 Hz), 127.4 (d, *J* = 13.5 Hz), 126.6 (d, *J* = 12.0 Hz), 126.3, 126.2, 125.6 (d, *J* = 5.5 Hz), 124.3 (d, *J* = 2.4 Hz), 124.0, 123.43 (q, *J* = 272.8), 123.39 (q, *J* = 272.8), 121.6 (hept, *J* = 7.7 Hz), 121.2 (hept, *J* = 7.7 Hz), 120.8.

^{31}**P NMR** (203 MHz, CD_2Cl_2) δ 147.0.

^{19}**F NMR** (471 MHz, CD_2Cl_2) δ -62.98 (6F), -63.03 (6F).

HRMS (ESI +) calculated for $[C_{49}H_{27}F_{12}N_2NaO_4P]^{+\cdot}$ $[M+Na]^{+\cdot}$ 989.1409 m/z; found 989.1408 m/z.

[α]^{26}$_{D}$ −100.5 (c 0.485, acetone).

(*R*)-B8

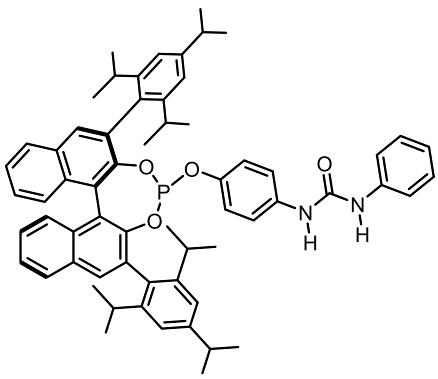

Prepared following general procedure GP1, using binaphthol **(*R*)-A3** (250 mg, 0.361 mmol) and hidroxyurea **U1** (165 mg, 0.723 mmol, 2.0 equiv). **(*R*)-B6** was obtained as a white solid (260 mg, 0.274 mmol, 76% yield) after FCC (CyH:AcOEt dry load silica, 90:10).

M.p. 271 °C.

^{1}**H NMR** (500 MHz, CD_2Cl_2) δ 7.96 (dd, *J* = 14.3, 8.2 Hz, 2H), 7.91 (s, 2H), 7.50 (dt, *J* = 14.1, 7.3 Hz, 2H), 7.42 (t, *J* = 8.8 Hz, 2H), 7.36 – 7.23 (m, 6H), 7.17 – 7.11 (m, 3H), 7.10 – 7.04 (m, 2H), 6.98 (d, *J* = 8.8 Hz, 2H), 6.65 (t, *J* = 28.9 Hz, 2H), 6.04 (d, *J* = 8.7 Hz, 2H), 3.04 – 2.92 (m, 2H), 2.92 – 2.83 (m, 1H), 2.84 – 2.74 (m, 1H), 2.73 – 2.64 (m, 1H), 2.59 (hept, *J* = 6.2 Hz, 1H), 1.34 (d, *J* = 6.9 Hz, 6H), 1.31 (d, *J* = 3.1 Hz, 3H), 1.29 (d, *J* = 3.1 Hz, 3H), 1.24 (d, *J* = 6.8 Hz, 3H), 1.21 (d, *J* = 6.8 Hz, 3H), 1.15 (d, *J* = 6.8 Hz, 6H), 1.07 (d, *J* = 6.8 Hz, 3H), 1.02 (d, *J* = 6.8 Hz, 3H), 0.95 (d, *J* = 6.8 Hz, 3H), 0.90 (d, *J* = 6.8 Hz, 3H).

^{13}**C NMR** (126 MHz, CD_2Cl_2) δ 153.4, 149.2, 148.9, 148.2, 148.1, 148.0, 147.9 (d, *J* = 8.2 Hz), 147.8 (d, *J* = 11.5 Hz), 147.7, 145.6, 138.6, 134.8, 134.0, 133.0, 132.9, 132.7, 132.4, 132.1, 132.0, 131.7, 130.9, 129.5, 128.7 (d, *J* = 26.4 Hz), 127.7, 126.3 (d, *J* = 23.0 Hz), 126.0, 125.71 (d, *J* = 44.7 Hz), 124.3, 122.9 (d, *J* = 2.3 Hz), 122.3, 121.9 (d, *J* = 7.6 Hz), 121.5 (d, *J* = 19.7 Hz), 121.0, 120.8 (d, *J* = 14.5 Hz), 34.8 (d, *J* = 4.9 Hz), 31.7, 31.6, 31.2 (d, *J* = 4.6 Hz), 31.1, 27.3, 27.0, 26.3, 25.5, 25.1, 24.6, 24.4, 24.4, 23.5, 23.2, 23.0, 22.9.

^{31}**P NMR** (203 MHz, CD_2Cl_2) δ 140.4.

HRMS (ESI +) calculated for $[C_{63}H_{68}N_2O_4P]^{+\cdot}$ $[M+H]^{+\cdot}$ 947.4911 m/z; found 947.4913 m/z.

[α]$^{26}_{D}$ +16.1 (c 0.86, acetone).

Phosphite B9

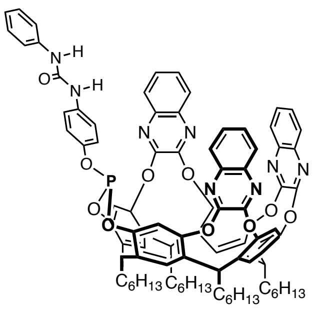

Prepared following general procedure GP1, using binaphthol **B** (200 mg, 0.166 mmol) and hydroxyurea **U1** (76 mg, 0.331 mmol, 2.0 equiv). **B9** was obtained as a beige solid (158 mg, 0.108 mmol, 65% yield) after FCC (CyH:AcOEt dry load silica, 90:10).

M.p. 247–250 °C.

^{1}H NMR (500 MHz, $CDCl_3$) δ 8.32 (s, 2H), 8.00 – 7.96 (m, 2H), 7.78 (dd, *J* = 6.3, 3.5 Hz, 2H), 7.72 (dd, *J* = 8.4, 1.4 Hz, 2H), 7.60 – 7.55 (m, 2H), 7.50 – 7.48 (m, 2H), 7.47 – 7.44 (m, 2H), 7.37 – 7.30 (m, 8H), 7.23 (d, J = 3.9 Hz, 6H), 7.13 – 7.09 (m, 1H), 6.78 (s, 1H), 5.71 (t, J = 8.2 Hz, 3H), 4.47 (td, *J* = 9.3, 2.0 Hz, 1H), 2.34 – 2.23 (m, 6H), 2.18 (q, *J* = 8.0 Hz, 2H), 1.52 – 1.23 (m, 34H), 1.04 – 0.85 (m, 12H).

^{13}C NMR (126 MHz, CDCl3) δ 153.6 (d, J = 4.7 Hz), 153.0 (d, J = 6.0 Hz), 152.8 (d, J = 4.9 Hz), 152.6 (d, J = 7.0 Hz), 147.9, 146.5 (d, J = 5.8 Hz), 139.9, 139.9, 138.0, 137.3 (d, J = 2.6 Hz), 136.1, 135.7, 134.6, 129.5, 129.3, 129.1, 128.1, 128.0 (d, J = 4.0 Hz), 124.6, 123.5, 122.9, 122.7, 122.0 (d, J = 5.2 Hz), 121.5, 119.1, 117.7, 36.0, 34.4, 34.2, 32.8, 32.2, 32.1, 32.0, 32.0, 31.7, 22.8, 14.2.

^{31}P NMR (203 MHz, $CDCl_3$) δ 131.5.

HRMS (ESI +) calculated for $[C_{89}H_{88}N_8O_{10}P]^{+\cdot}$ $[M+H]^{+\cdot}$ 1459.6356 m/z; found 1459.6358 m/z.

Phosphite B10

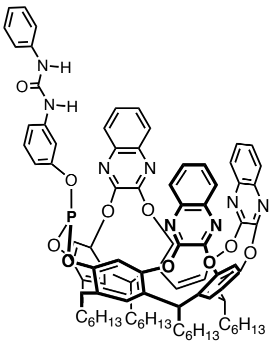

Prepared following general procedure GP1, using binaphthol **B** (400 mg, 0.332 mmol) and hydroxyurea **U1** (152 mg, 0.664 mmol, 2.0 equiv). **B10** was obtained as a beige solid (296 mg, 0.203 mmol, 61% yield) after FCC (CyH:AcOEt dry load silica, 90:10).

M.p. 270–273 °C.

^{1}H NMR (500 MHz, $CDCl_3$) δ 8.32 (d, *J* = 1.6 Hz, 2H), 7.96 (dd, *J* = 8.3, 1.4 Hz, 2H), 7.78 (dd, *J* = 6.3, 3.5 Hz, 2H), 7.71 (d, *J* = 8.3 Hz, 2H), 7.56 (td, *J* = 9.0, 1.2 Hz, 2H), 7.44 (td, *J* = 6.3, 2.6 Hz, 5H), 7.37 (s, 3H), 7.36 – 7.32 (m, 3H), 7.25 – 7.22 (m, 6H), 7.08 – 7.02 (m, 1H), 7.00 – 6.94 (m, 1H), 5.71 (t, *J* = 8.2 Hz, 3H), 4.48 (t, *J* = 7.9 Hz, 1H), 2.32 – 2.25 (m, 7H), 2.17 (q, *J* = 7.9 Hz, 2H), 1.54 – 1.29 (m, 36H), 0.97 – 0.84 (m, 18H).

^{13}C NMR (126 MHz, $CDCl_3$) δ 153.0, 153.0, 152.8, 152.7, 152.6, 152.5, 151.9, 146.5 (d, *J* = 5.9 Hz), 139.9, 139.8, 138.0, 137.4 (d, *J* = 2.6 Hz), 136.1 (d, *J* = 1.9 Hz), 135.7, 130.3, 129.6, 129.5, 129.4, 129.3, 129.1, 128.0, 124.4, 123.5, 123.0, 121.1, 119.1, 117.7 (d, *J* = 3.0 Hz), 116.5, 116.2, 112.9 (d, *J* = 4.7 Hz), 36.0, 34.4, 34.2, 32.8, 32.2, 32.1, 32.0, 31.7, 29.9, 29.6, 29.5, 28.2, 28.1, 27.9, 22.8 (d, *J* = 2.9 Hz), 14.2, 14.2.

^{31}**P NMR** (203 MHz, $CDCl_3$) δ 132.0.

HRMS (ESI +) calculated for $[C_{89}H_{87}N_8O_{10}P]^{+\cdot}$ $[M+H]^{+\cdot}$ 1459.6356 m/z; found 1459.6341 m/z.

Phosphite B11

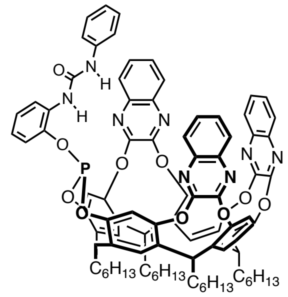

Prepared following general procedure GP1, using binaphthol **B** (150 mg, 0.125 mmol) and hydroxyurea **U1** (57 mg, 0.249 mmol, 2.0 equiv). **B11** was obtained as a beige solid (101 mg, 0.069 mmol, 56% yield) after FCC (CyH /AcOEt dry load silica, 90:10).

M.p. 259–261 °C.

^{1}**H NMR** (500 MHz, $CDCl_3$) δ 8.31 (d, *J*= 2.5 Hz, 2H), 8.25 (t, *J* = 6.9 Hz, 1H), 7.96 (d, *J* = 8.3 Hz, 2H), 7.81 – 7.77 (m, 2H), 7.68 – 7.64 (m, 2H), 7.56 (ddd, *J* = 8.4, 7.0, 1.4 Hz, 2H), 7.50 – 7.42 (m, 6H), 7.41 – 7.37 (m, 2H), 7.32 (d, *J* = 3.7 Hz, 2H), 7.30 – 7.26 (m, 2H), 7.25 – 7.22 (m, 5H), 7.22 – 7.17 (m, 1H), 7.08 – 7.01 (m, 2H), 5.72 (td, *J* = 6.8, 4.2 Hz, 3H), 4.44 (t, *J* = 7.9 Hz, 1H), 2.36 – 2.24 (m, 6H), 2.18 (q, *J* = 8.0 Hz, 2H), 1.61 – 1.30 (m, 36H), 0.99 – 0.88 (m, .19H).

^{13}**C NMR** (126 MHz, $CDCl_3$) δ 153.0, 152.9, 152.7 (d, *J* = 3.6 Hz), 152.6, 146.0 (d, *J* = 5.5 Hz), 141.1 (d, *J* = 2.9 Hz), 139.9, 139.8, 138.0 (d, *J* = 5.2 Hz), 137.2 (d, *J* = 2.6 Hz), 136.1, 136.1, 136.0, 130.7, 129.6 (d, *J* = 2.8 Hz), 129.3, 129.1, 128.0, 128.0 (d, *J* = 3.1 Hz), 125.4, 124.7 (d, *J* = 5.8 Hz), 123.5, 123.3, 123.0, 121.9, 120.2 (d, *J* = 8.2 Hz), 119.2, 117.7, 36.0, 34.3, 34.2, 32.8, 32.3, 32.1, 32.0, 31.7, 29.9, 29.6, 29.5, 28.2 (d, *J* = 2.7 Hz), 28.0, 22.8, 14.2.

HRMS (ESI +) calculated for $[C_{89}H_{87}N_8NaO_{10}P]^{+\cdot}$ $[M+H]^{+\cdot}$ 1481.6175 m/z; found 1481.6167 m/z.

^{31}**P NMR** (203 MHz, $CDCl_3$) δ 134.8.

(*R*)-Au1

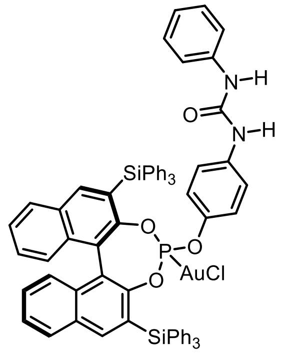

Prepared following general procedure GP2, using phosphite **(*R*)-B1** (77.5 mg, 0.073 mmol). **(*R*)-Au1** was obtained as a white solid (89 mg, 0.069 mmol, 94% yield) after FCC (CyH:AcOEt dry load silica, 100:0 to 80:20).

M.p. 200–203 ºC.

^{1}**H NMR** (500 MHz, CD_2Cl_2) δ 8.21 (s, 1H), 8.13 (s, 1H), 7.87 (d, *J* = 8.2 Hz, 1H), 7.84 (d, *J* = 8.2 Hz, 1H), 7.62 – 7.57 (m, 6H), 7.56 – 7.46 (m, 8H), 7.35 (t, *J* = 7.5 Hz, 13H), 7.33 – 7.20 (m, 17H), 7.04 (t, *J* = 7.3 Hz, 1H), 6.94 (d, *J* = 8.9 Hz, 4H), 5.90 (d, *J* = 8.7 Hz, 2H).

^{13}**C NMR** (126 MHz, CD_2Cl_2) δ 152.7, 151.0 (d, *J* = 13.3 Hz), 149.8 (d, *J* = 7.6 Hz), 144.2, 142.3 (d, *J* = 40.1 Hz), 138.3, 136.6, 136.4, 136.1, 134.2 (d, *J* = 29.9 Hz), 133.5, 133.3, 131.4, 131.1, 130.0 (d, *J* = 2.0 Hz), 129.1, 128.8 (d, *J* = 10.4 Hz), 128.3, 128.1, 127.9 (d, *J* = 8.2 Hz), 126.7 (d, *J* = 30.5 Hz), 126.1 (d, *J* = 11.7 Hz), 125.7, 123.8, 122.5, 122.0, 121.3 (d, *J* = 5.3 Hz), 120.6, 120.5.

^{31}**P NMR** (202 MHz, CD_2Cl_2) δ 123.7.

HRMS (ESI +) calculated for $[C_{69}H_{51}AuClN_2NaO_4PSi_2]^{+\cdot}$ $[M+Na]^{+\cdot}$ 1313.2371 m/z; found 1313.2377 m/z.

$[\alpha]^{24.6}_{D}$ −55.7 (c 1.22, $CHCl_3$).

(*R*)-Au2

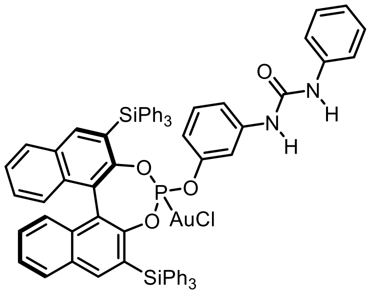

Prepared following general procedure GP2, using phosphite **(*R*)-B2** (112 mg, 0.106 mmol). **(*R*)-Au2** was obtained as a white solid (132 mg, 0.102 mmol, 97% yield) after FCC (CyH:AcOEt dry load silica, 100:0 to 80:20).

M.p. 208–211 °C.

^{1}H NMR (500 MHz, CD_2Cl_2) δ 8.20 (s, 1H), 8.13 (s, 1H), 7.90 – 7.80 (m, 2H), 7.61 – 7.55 (m, 6H), 7.55 – 7.48 (m, 8H), 7.42 – 7.22 (m, 28H), 7.09 (tt, *J* = 7.3, 1.2 Hz, 1H), 6.83 (t, *J* = 8.2 Hz, 1H), 6.69 (s, 1H), 6.31 (s, 1H), 5.99 – 5.90 (m, 1H), 5.81 – 5.70 (m, 1H).

^{13}C NMR (126 MHz, CD_2Cl_2) δ 152.4, 151.5 (d, *J* = 13.0 Hz), 150.1 (d, *J* = 7.3 Hz), 149.3 (d, *J* = 5.0 Hz), 142.7 (d, *J* = 38.9 Hz), 139.9, 138.6, 137.0 (d, *J* = 17.0 Hz), 134.6 (d, *J* = 30.2 Hz), 133.8 (d, *J* = 14.1 Hz), 131.8, 131.47, 130.3, 130.2, 129.6, 129.3 (d, *J* = 8.4 Hz), 128.6 (d, *J* = 22.1 Hz), 128.4, 127.1 (d, *J* = 17.6 Hz), 126.5 (d, *J* = 18.0 Hz), 126.3, 126.1 (d, *J* = 3.5 Hz), 124.3, 123.0, 122.4, 120.8, 117.2, 115.7 (d, *J* = 5.7 Hz), 112.2 (d, *J* = 5.6 Hz).

^{31}P NMR (202 MHz, CD_2Cl_2) δ 123.0.

HRMS (ESI +) calculated for $[C_{69}H_{51}AuClN_2NaO_4PSi_2]^{+\cdot}$ $[M+Na]^{+\cdot}$ 1313.2371 m/z; found 1313.2371 m/z.

$[\alpha]^{24.6}_{D}$ −65.7 (c 1.0, $CHCl_3$).

(*R*)-Au3

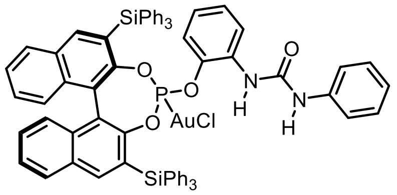

Prepared following general procedure GP2, using phosphite **(*R*)-B3** (115 mg, 0.109 mmol). **(*R*)-Au3** was obtained as a white solid (139 mg, 0.103 mmol, 95% yield) after FCC (CyH:AcOEt dry load silica, 100:0 to 90:10).

M.p. 189–192 °C.

^{1}H NMR (500 MHz, $CDCl_3$) δ 8.23 (d, *J* = 8.2 Hz, 1H), 8.18 (d, *J* = 14.6 Hz, 2H), 7.90 (dd, *J* = 11.1, 8.5 Hz, 2H), 7.55 (dd, *J* = 12.5, 4.9 Hz, 8H), 7.47 (d, *J* = 7.0 Hz, 7H), 7.39 (dq, *J* = 13.5, 7.8, 7.4 Hz, 7H), 7.30 (t, *J* = 7.3 Hz, 10H), 7.22 (q, *J* = 7.6 Hz, 8H), 7.03 (t, *J* = 7.9 Hz, 1H), 6.97 (t, *J* = 7.3 Hz, 1H), 6.42 – 6.34 (m, 2H), 5.82 (d, *J* = 8.2 Hz, 1H), 5.78 (s, 1H).

^{13}C NMR (126 MHz, $CDCl_3$) δ 151.6, 150.6 (d, *J* = 13.2 Hz), 149.6 (d, *J* = 7.3 Hz), 142.6, 142.3, 138.0, 137.5 (d, *J* = 10.4 Hz), 136.7, 136.5, 134.0 (d, *J* = 55.2 Hz), 133.2 (d, *J* = 26.3 Hz), 131.4 (d, *J* = 49.6 Hz), 130.3, 130.1 (d, *J* = 2.2 Hz), 129.3, 129.2, 128.4, 128.3, 128.2, 126.8 (d, *J* = 28.0 Hz), 126.6, 126.5,

126.2 (d, J = 3.1 Hz), 126.1, 124.1, 122.7 (d, J = 2.9 Hz), 122.3 (d, J = 3.2 Hz), 122.0, 120.9, 120.3 (d, J = 3.9 Hz), 120.2.

^{31}P NMR (203 MHz, $CDCl_3$) δ 120.8.

HRMS (ESI +) calculated for $[C_{69}H_{51}AuClN_2NaO_4PSi_2]^{+\cdot}$ $[M+Na]^{+\cdot}$ 1313.2371 m/z; found 1313.2346 m/z.

$[\alpha]^{24.6}_D$ −108 (c 2.09, $CHCl_3$).

(*R*)-Au4

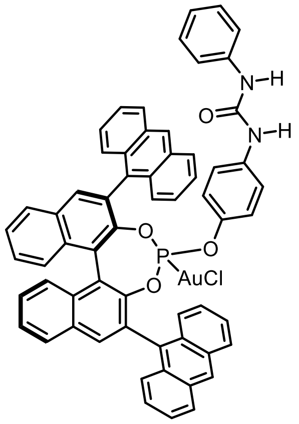

Prepared following general procedure GP2, using phosphite **(*R*)-B4** (348 mg, 0.389 mmol). **(*R*)-Au4** was obtained as a military green solid (395 mg, 0.350 mmol, 90% yield) after FCC (CyH:AcOEt dry load silica, 100:0 to 80:20).

M.p. 253–255 °C.

^{1}H NMR (500 MHz, CD_2Cl_2) δ 8.58 (s, 1H), 8.45 (s, 1H), 8.34 (s, 1H), 8.20 – 8.13 (m, 2H), 8.08 (d, J = 8.3 Hz, 2H), 8.06 – 7.97 (m, 2H), 7.97 – 7.88 (m, 2H), 7.80 (t, J = 9.4 Hz, 2H), 7.75 – 7.70 (m, 3H), 7.71 – 7.64 (m, 2H), 7.64 – 7.52 (m, 2H), 7.49 – 7.34 (m, 6H), 7.30 – 7.18 (m, 5H), 7.04 – 6.97 (m, 2H), 6.64 – 6.55 (m, 1H), 6.53 – 6.41 (m, 3H), 4.81 (d, J = 8.7 Hz, 2H).

^{13}C NMR (126 MHz, CD_2Cl_2) δ 152.4, 146.2 (d, J = 13.4 Hz), 144.0 (d, J = 7.0 Hz), 143.7, 138.2, 135.8, 135.0, 134.5, 132.9, 132.75(d, J = 31.5 Hz), 132.6, 132.2, 131.9, 131.7, 131.5, 131.2 (d, J = 8.6 Hz), 130.9, 130.8, 130.5 (d, J = 5.9 Hz), 130.3, 130.0, 129.8 (d, J = 2.8 Hz), 129.6, 129.0, 128.9, 128.7, 128.6 (d, J = 7.4 Hz), 128.3, 128.1 (d, J = 8.8 Hz), 127.5, 127.4, 127.4, 126.9, 126.8, 126.7, 126.3, 126.2, 126.0, 125.9, 125.6, 125.3, 125.2, 125.1, 124.1, 123.7, 122.8, 120.6, 120.4, 119.6 (d, J = 6.9 Hz).

^{31}P NMR (202 MHz, CD_2Cl_2) δ 125.4.

HRMS (ESI +) calculated for $[C_{61}H_{39}AuClN_2NaO_4P]^{+\cdot}$ $[M+Na]^{+\cdot}$ 1149.1894 m/z; found 1149.1885 m/z.

$[\alpha]^{24.6}_D$ −36.5 (c 1.0, $CHCl_3$).

(*R*)-Au5

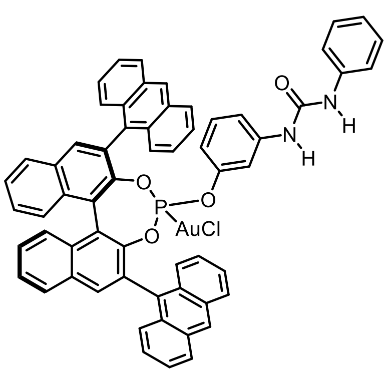

Prepared following general procedure GP2, using phosphite **(*R*)-B5** (155.7 mg, 0.174 mmol). **(*R*)-Au5** was obtained as a military green solid (182 mg, 0.161 mmol, 93% yield) after FCC (CyH:AcOEt dry load silica, 100:0 to 80:20).

M.p. 316 °C.

^{1}H NMR (500 MHz, CD_2Cl_2) δ 8.55 (d, J = 2.6 Hz, 1H), 8.48 (d, J = 3.0 Hz, 1H), 8.31 (s, 1H), 8.19 – 8.13 (m, 2H), 8.12 – 7.97 (m, 4H), 7.92 (d, J = 8.8 Hz, 1H), 7.87 – 7.76 (m, 3H), 7.76 – 7.65 (m, 4H), 7.65 – 7.53 (m, 3H), 7.52 –

7.37 (m, 6H), 7.36 – 7.24 (m, 4H), 7.18 – 7.09 (m, 2H), 7.02 – 6.96 (m, 1H), 6.96 – 6.86 (m, 1H), 6.46 – 6.35 (m, 2H), 5.95 (s, 1H), 4.76 – 4.61 (m, 2H).

^{13}C NMR (126 MHz, CD_2Cl_2) δ 152.4, 148.9, 146.7 (d, J = 13.5 Hz), 144.6 (d, J = 6.8 Hz), 139.6, 138.7, 135.6, 135.2, 133.5 (d, J = 1.3 Hz), 133.2 (d, J = 1.6 Hz), 132.8, 132.6, 132.4, 132.1, 131.9, 131.7, 131.5, 131.5, 131.1, 131.0, 130.8, 130.7, 130.6 (d, J = 2.4 Hz), 130.3, 130.0, 129.81 129.4, 129.3 (d, J = 5.5 Hz), 129.2, 129.1, 129.0, 128.8 (d, J = 9.8 Hz), 128.7, 128.1, 128.04, 128.02, 127.99, 127.5 (d, J = 23.7 Hz), 127.2, 127.0, 126.9, 126.7, 126.6, 126.5, 126.4, 126.0, 125.8, 125.6, 124.8, 124.6, 123.5 (d, J = 2.9 Hz), 121.55 – 121.25 (m), 117.7, 114.2, 111.3 (d, J = 7.8 Hz).

^{31}P NMR (202 MHz, CD_2Cl_2) δ 125.5.

HRMS (ESI +) calculated for $[C_{61}H_{40}AuClN_2O_4P]^{+\cdot}$ $[M+H]^{+\cdot}$ 1127.2074 m/z; found 1127.2029 m/z.

$[\alpha]^{26}_{D}$ −37.4 (c 0.32, acetone).

(*R*)-Au6

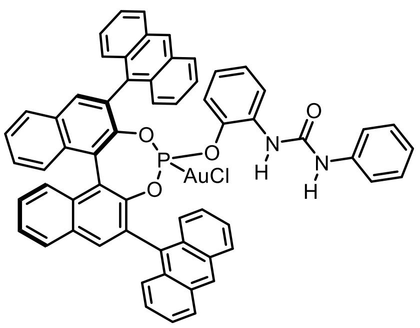

Prepared following general procedure GP2, using phosphite **(*R*)-B6** (53 mg, 0.059 mmol). **(*R*)-Au6** was obtained as a white solid (66 mg, 0.059 mmol, 99% yield) after FCC (CyH:AcOEt dry load silica, 100:0 to 80:20).

M.p. 240–242 °C.

^{1}H NMR (500 MHz, $CDCl_3$) δ 8.50 (s, 1H), 8.38 (s, 1H), 8.33 (s, 1H), 8.14 (d, J = 8.3 Hz, 1H), 8.11 – 8.06 (m, 2H), 8.04 (d, J = 8.2 Hz, 2H), 8.00 – 7.97 (m, 1H), 7.92 (d, J = 8.4 Hz, 1H), 7.84 – 7.58 (m, 10H), 7.57 – 7.52 (m, 1H), 7.49 – 7.27 (m, 11H), 7.12 (t, J = 7.6 Hz, 1H), 7.07 – 7.00 (m, 1H), 6.93 – 6.81 (m, 1H), 6.70 (t, J = 7.9 Hz, 1H), 5.98 (td, J = 7.8, 1.6 Hz, 1H), 5.63 (s, 1H), 4.98 (d, J = 8.2 Hz, 1H), 4.77 (s, 1H).

^{13}C NMR (126 MHz, $CDCl_3$) δ 150.8, 146.4 (d, J = 13.7 Hz), 143.8 (d, J = 6.6 Hz), 138.2, 135.2, 134.9, 132.9, 132.7, 132.4, 132.0, 131.8, 131.5, 131.3, 131.1, 130.9, 130.7, 130.55 – 130.33 (m), 130.2 (d, J = 2.8 Hz), 130.0, 129.6 (d, J = 5.4 Hz), 129.3, 129.2, 129.0 (d, J = 3.2 Hz), 128.9 (d, J = 2.4 Hz), 128.8 (d, J = 4.1 Hz), 128.3, 127.9, 127.8, 127.7, 127.6, 127.3, 127.0, 126.9, 126.3, 126.3, 126.1, 126.05, 125.99, 125.9, 125.7, 125.6, 124.9, 124.3 (d, J = 3.2 Hz), 123.6, 122.9 (d, J = 3.0 Hz), 122.1, 120.2, 120.0, 118.8, 118.7.

^{31}P NMR (202 MHz, $CDCl_3$) δ 125.80.

HRMS (ESI +) calculated for $[C_{61}H_{40}AuClN_2O_4P]^{+\cdot}$ $[M+H]^{+\cdot}$ 1127.2074 m/z; found 1127.2055 m/z.

$[\alpha]^{24.6}_{D}$ 126.9 (c 0.39, $CHCl_3$).

(*R*)-Au7

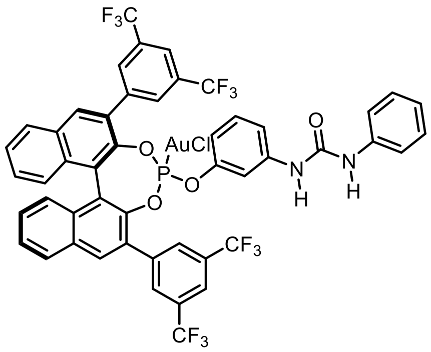

Prepared following general procedure GP2, using phosphite **(*R*)-B7** (73.1 mg, 0.076 mmol). **(*R*)-Au7** was obtained as a white solid (82 mg, 0.068 mmol, 90% yield) after FCC (CyH:AcOEt dry load silica, 100:0 to 80:20).

M.p. 187–189 °C.

^{1}H NMR (300 MHz, $CDCl_3$) δ 8.23 (s, 1H), 8.18 (s, 2H), 8.16 – 8.08 (m, 2H), 8.07 – 8.00 (m, 3H), 7.98 – 7.85 (m, 2H), 7.74 – 7.58 (m, 2H), 7.57 – 7.47 (m, 4H), 7.37 – 7.27 (m, 4H), 7.17 – 7.02 (m, 2H), 6.93 (t, *J* = 8.2 Hz, 1H), 6.66 – 6.43 (m, 3H), 6.05 – 5.96 (m, 1H).

^{13}C NMR (126 MHz, $CDCl_3$) δ 152.6, 149.2, 143.5 (d, *J* = 13.2 Hz), 141.3 (d, *J* = 6.7 Hz), 140.7, 138.8, 138.0, 137.7, 132.7 (d, *J* = 11.3 Hz), 132.5, 132.4, 132.2 (d, *J* = 6.8 Hz), 131.9 (d, *J* = 18.5 Hz), 131.1 (d, *J* = 2.5 Hz), 130.6, 130.3, 130.0, 129.3, 129.1, 128.4 (d, *J* = 13.8 Hz), 127.7, 127.5, 127.1 (d, *J* = 6.3 Hz), 125.3 (d, *J* = 3.3 Hz), 124.4, 123.4 (d, *J* = 2.8 Hz), 122.6 – 122.4 (m), 122.4 – 122.2 (m), 121.2, 117.6, 113.3 (d, *J* = 7.3 Hz), 110.8 (d, *J* = 6.6 Hz).

^{31}P NMR (202 MHz, $CDCl_3$) δ 125.3.

^{19}F NMR (471 MHz, $CDCl_3$) δ -62.5, -62.6.

HRMS (ESI +) calculated for $[C_{49}H_{27}AuClF_{12}N_2NaO_4P]^{+\cdot}$ $[M+Na]^{+\cdot}$ 1221.0763 m/z; found 1221.0756 m/z.

$[\alpha]^{24.6}_D$ −18.7 (c 1.95, acetone).

(*R*)-Au8

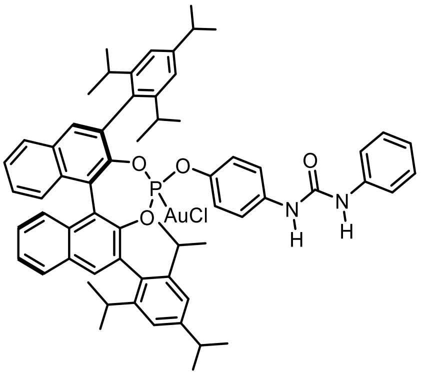

Prepared following general procedure GP2, using phosphite **(*R*)-B8** (240 mg, 0.253 mmol). **(*R*)-Au8** was obtained as a white solid (253 mg, 0.214 mmol, 85% yield) after FCC (CyH:AcOEt dry load silica, 100:0 to 80:20).

M.p. 250–208 °C.

^{1}H NMR (500 MHz, CD_2Cl_2) δ 8.04 (s, 2H), 8.03 (d, *J* = 8.2 Hz, 2H), 8.01 – 7.94 (m, 2H), 7.59 (ddd, *J* = 8.1, 5.6, 2.4 Hz, 1H), 7.57 – 7.51 (m, 1H), 7.41 – 7.34 (m, 2H), 7.36 – 7.26 (m, 5H), 7.20 (dd, *J* = 7.6, 1.8 Hz, 2H), 7.16 – 7.10 (m, 2H), 7.13 – 7.04 (m, 5H), 6.59 (s, 2H), 6.15 – 6.07 (m, 2H), 3.10 (hept, *J* = 6.8 Hz, 1H), 2.99 (dh, *J* = 21.0, 6.9 Hz, 2H), 2.88 (h, *J* = 6.8 Hz, 1H), 2.67 – 2.54 (m, *J* = 6.7 Hz, 2H), 1.38 – 1.30 (m, 18H), 1.30 (d, *J* = 7.0 Hz, 11H), 1.20 (d, *J* = 6.7 Hz, 3H), 1.11 (d, *J* = 6.8 Hz, 3H), 1.06 (d, *J* = 6.8 Hz, 3H), 0.91 (d, *J* = 6.8 Hz, 3H), 0.78 (d, *J* = 6.8 Hz, 3H).

^{13}C NMR (126 MHz, CD_2Cl_2) δ 152.73, 149.73 (d, *J* = 6.1 Hz), 148.27 (d, *J* = 13.7 Hz), 147.91 (d, *J* = 34.8 Hz), 146.37 (d, *J* = 14.2 Hz), 145.46, 138.51, 137.09, 133.65, 133.54, 133.00, 132.61, 131.94, 131.89 (d, *J* = 3.5 Hz), 131.77, 131.15, 130.49, 129.54, 128.82, 127.88, 127.41, 127.14, 126.95, 126.91, 126.55, 124.40, 123.82 (d, *J* = 3.7 Hz), 122.29, 122.16 (d, *J* = 6.6 Hz), 122.01, 121.61, 121.54, 121.19,

121.14, 34.76 (d, J = 2.3 Hz), 31.97, 31.55 (d, J = 5.2 Hz), 31.05, 27.29 (d, J = 9.5 Hz), 26.84, 26.14, 25.54, 25.29, 24.59, 24.33, 24.28, 24.20, 23.75, 23.13, 22.87.

^{31}P NMR (202 MHz, CD_2Cl_2) δ 124.22.

HRMS (ESI +) calculated for $[C_{63}H_{67}AuClN_2NaO_4P]^{+\cdot}$ $[M+Na]^{+\cdot}$ 1201.4085 m/z; found 1201.4091 m/z.

$[\alpha]^{24.6}_D$ +106.2 (c 0.84, $CHCl_3$).

Complex Au9

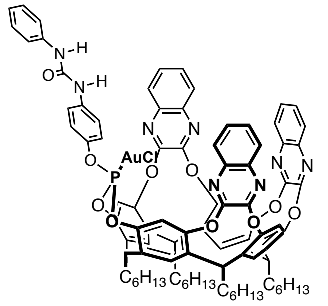

Prepared following general procedure GP2, using phosphite **B9** (480 mg, 0.329 mmol). **Au9** was obtained as a white solid (466 mg, 0.275 mmol, 84% yield) after FCC (CyH:AcOEt dry load silica, 100:0 to 80:20).

M.p. 234–240 °C.

^{1}H NMR (500 MHz, $CDCl_3$) δ 8.18 (s, 2H), 7.85 (dd, J = 8.3, 1.5 Hz, 2H), 7.80 – 7.76 (m, 3H), 7.76 – 7.73 (m, 1H), 7.51 – 7.48 (m, 4H), 7.47 (dd, J = 4.8, 2.1 Hz, 4H), 7.43 (td, J = 8.4, 7.7 Hz, 2H), 7.40 – 7.35 (m, 4H), 7.33 – 7.30 (m, 5H), 7.18 – 7.14 (m, 1H), 6.94 (s, 1H), 6.79 (s, 1H), 5.77 (t, J = 8.2 Hz, 1H), 5.72 (t, J = 8.2 Hz, 2H), 4.52 (td, J = 8.0, 3.0 Hz, 1H), 2.38 – 2.17 (m, 8H), 1.54 – 1.30 (m, 38H), 0.93 (m, 15H).

^{13}C NMR (126 MHz, $CDCl_3$) δ 153.1, 153.0, 152.8, 152.6, 152.3, 152.1, 145.1 (d, J = 4.7 Hz), 143.6 (d, J = 3.0 Hz), 140.1 (d, J = 6.4 Hz), 139.8, 138.3 (d, J = 1.9 Hz), 137.8, 137.1, 136.5, 135.9 (d, J = 2.9 Hz), 135.2, 129.7, 129.6, 129.6, 129.3, 128.6, 127.9, 127.5, 124.9, 123.8, 122.8, 122.2 (d, J = 4.5 Hz), 121.9, 121.8, 35.9, 34.2, 32.8, 32.8, 32.1, 32.0, 30.5, 29.8, 29.6, 29.5, 29.4, 28.2, 28.1, 27.9, 22.8, 22.8, 22.8, 14.2.

^{31}P NMR (202 MHz, $CDCl_3$) 109.7.

HRMS (ESI +) calculated for $[C_{89}H_{87}AuClN_8NaO_{10}P]^{+\cdot}$ $[M+Na]^{+\cdot}$ 1713.5529 m/z; found 1713.5519 m/z.

Complex Au10

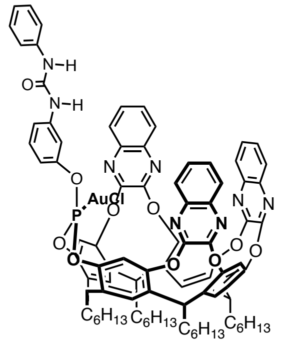

Prepared following general procedure GP2, using phosphite **B10** (296 mg, 0.203 mmol). **Au10** was obtained as a white solid (126 mg, 0.075 mmol, 37% yield) after FCC (CyH:AcOEt dry load silica, 100:0 to 80:20).

M.p. 176–177 ºC.

^{1}H NMR (500 MHz, $CDCl_3$) δ 8.19 (s, 2H), 7.85 (dd, J = 8.3, 1.4 Hz, 2H), 7.79 – 7.75 (m, 2H), 7.73 (dd, J = 8.4, 1.4 Hz, 2H), 7.64 (s, 1H), 7.51 – 7.48 (m, 5H), 7.47 (d, J = 1.4 Hz, 1H), 7.44 – 7.39 (m, 4H), 7.33 – 7.29 (m, 5H), 7.27 (s, 1H), 7.25 – 7.21 (m, 1H), 7.19 (s, 1H), 7.13 – 7.01 (m, 3H), 5.78 (t, J = 8.2 Hz, 1H), 5.73 (t, J = 8.2 Hz, 2H), 4.55 (td, J = 7.9, 3.0 Hz, 1H), 2.39 – 2.18 (m, 8H), 1.59 – 1.28 (m, 34H), 0.98 – 0.89 (m, 12H).

^{31}P NMR (203 MHz, $CDCl_3$) δ 109.4.

^{13}C NMR (126 MHz, $CDCl_3$) δ 153.0, 153.0 (d, J = 1.8 Hz), 152.9, 152.7, 152.6, 152.3, 152.1, 149.8 (d, J = 3.6 Hz), 143.6 (d, J = 2.8 Hz), 140.7, 140.1 (d, J = 5.1 Hz), 139.8, 138.3 (d, J = 2.1 Hz), 137.9, 136.5, 136.0 (d, J = 2.9 Hz), 135.3, 130.8, 129.7, 129.6, 129.5, 129.3, 128.5, 127.9, 127.5, 124.5, 123.8, 122.9, 121.1, 119.0, 117.8 (d, J = 3.9 Hz), 117.5, 115.7 (d, J = 4.7 Hz), 112.4 (d, J = 5.0 Hz), 66.0, 36.0, 34.2, 32.8, 32.1, 32.0, 30.6, 29.6, 29.5, 29.5, 28.2, 28.1, 28.0, 22.8, 22.8, 22.8, 15.4, 14.2, 14.2.

HRMS (ESI +) calculated for $[C_{89}H_{87}AuClN_8NaO_{10}P]^{+\cdot}$ $[M+Na]^{+\cdot}$ 1713.5529 m/z; found 1713.5512 m/z.

Complex Au11

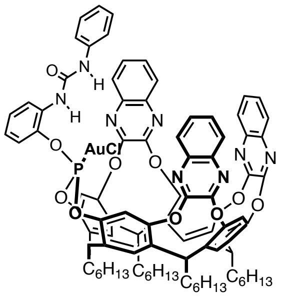

Prepared following general procedure GP2, using phosphite **B11** (76 mg, 0.052 mmol). **Au11** was obtained as a white solid (71 mg, 0.042 mmol, 81% yield) after FCC (CyH:AcOEt dry load silica, 100:0 to 80:20).

M.p. 169–173 ºC.

^{1}H NMR (500 MHz, $CDCl_3$) δ 8.51 (d, J = 8.3 Hz, 1H), 8.21 (s, 2H), 7.95 (s, 2H), 7.85 – 7.79 (m, 2H), 7.75 (d, J = 8.3 Hz, 2H), 7.58 (s, 2H), 7.52 (dd, J = 6.2, 3.6 Hz, 3H), 7.46 – 7.40 (m, 4H), 7.34 (t, J = 8.0 Hz, 1H), 7.30 (s, 2H), 7.22 (s, 2H), 7.15 (s, 6H), 7.09 (td, J = 7.9, 1.6 Hz, 1H), 5.76 (t, J = 8.2 Hz, 1H), 5.70 (t, J = 8.2 Hz, 2H), 4.18 (t, J = 7.1 Hz, 1H), 2.30 (q, J = 8.0 Hz, 2H), 2.27 – 2.16 (m, 6H), 1.56 – 1.31 (m, 33H), 0.98 – 0.90 (m, 14H).

^{13}C NMR (126 MHz, $CDCl_3$) δ 153.0, 153.0, 152.8, 152.6, 152.6, 152.3, 151.9, 143.4, 134.0 (d, J = 6.5 Hz), 139.6, 139.0 (d, J = 12.4 Hz), 138.3, 138.3, 136.4, 135.7, 135.3, 131.6, 129.7, 129.5, 129.4, 129.3, 127.9, 127.6, 127.4, 123.6, 123.0, 122.9, 122.3, 121.9, 118.9, 117.7, (d, J = 3.9 Hz), 35.3, 34.2, 34.2, 32.8, 32.6, 32.1, 32.0, 31.9, 30.0, 29.9, 29.5, 28.8, 28.1, 28.1, 27.4, 22.8, 22.8, 22.7, 14.2, 14.2.

^{31}P NMR (203 MHz, $CDCl_3$) δ 105.6.

HRMS (ESI +) calculated for $[C_{89}H_{87}AuClN_8NaO_{10}P]^{+\cdot}$ $[M+Na]^{+\cdot}$ 1713.5529 m/z; found 1713.5519 m/z.

3. Synthesis of substrates

Enynes **1a**–**1o** were prepared from the corresponding allyl bromides and commercially available propargyl malonate. Allyl bromides were not stable and were prepared from the corresponding allyl alcohols and immediately used or stored in the freezer for a short period of time.

General procedure GP3: 1,6-enyne preparation

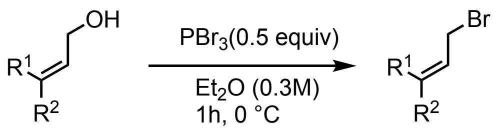

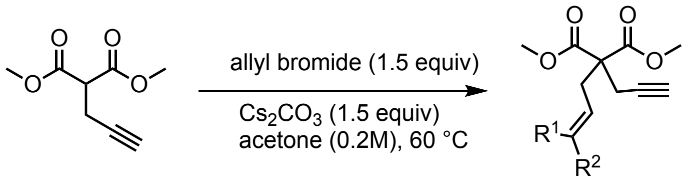

1

Allyl bromide preparation: To a flame-dried flask charged with a stirring bar and the corresponding allyl alcohol was added anhydrous Et_2O (0.3 M). The solution was cooled to 0 °C with an ice/water bath and PBr_3 (0.5 equiv) was added dropwise. The mixture was allowed to warm up to room temperature and was stirred until starting material consumption (monitored by GC-MS and TLC). The reaction was then quenched by dropwise addition of H_2O, the AP is then extracted with Et_2O (2x). The combined organic layers are washed with brine, dried over Na_2SO_4 or $MgSO_4$, filtered and evaporated. The crude was used as such in the next step without further purification.

Alkylation propargyl malonate: To a flask equipped with a stirring bar and charged with Cs_2CO_3 (1.5 equiv) was added HPLC-grade acetone. Then propargyl malonate was added dropwise (1 equiv) and the system was stirred for 5 min. Finally, all bromide (1.5 equiv) was added as a solution in acetone (0.2M final concentration). The system was heated to reflux and left stirring until starting material consumption (monitored by GC-MS and TLC). The reaction was filtered and the solvent was evaporated. The crude was purified by FCC (CyH:AcOEt, solvent mixtures) affording the corresponding 1,6-enyne.

Dimethyl 2-Cinnamyl-2-(prop-2-yn-1-yl)malonate (1a)

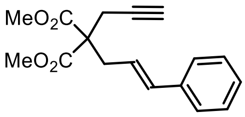

Prepared following general procedure GP3, using (*E*)-cinnamyl alcohol (2.69 g, 20.8 mmol). Cinnamyl bromide was obtained as a colorless oil (3.30 g, 16.8 mmol, 83%) and was used in the following step with propargyl malonate (1.70 mL, 11.2 mmol, 1.0 equiv). Enyne **1a** was obtained as a white solid (2.90 g, 10.1 mmol, 91% yield) after FCC (CyH:AcOEt dry load silica, 100:0 to 90:10).

^{1}H NMR (300 MHz, $CDCl_3$) δ 7.37 – 7.26 (m, 4H), 7.25 – 7.18 (m, 1H), 6.52 (dt, *J* = 15.6, 1.3 Hz, 1H), 6.00 (dt, *J* = 15.5, 7.7 Hz, 1H), 3.76 (s, 6H), 2.97 (dd, *J* = 7.7, 1.3 Hz, 2H), 2.85 (d, *J* = 2.7 Hz, 2H), 2.06 (t, *J* = 5.4 Hz, 1H).

^{13}C NMR (75 MHz, $CDCl_3$) δ 170.3, 137.1, 134.8, 128.7, 127.7, 126.4, 123.2, 79.0, 71.8, 57.4, 53.0, 36.0, 23.1.

Diisopropyl (*E*)-2-Cinnamyl-2-(prop-2-yn-1-yl)malonate (1b)

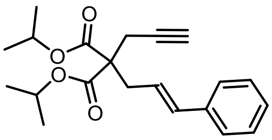

Prepared following a modified literature procedure.[6] Under argon, to a solution of **1a** (400 mg, 1.40 mmol) in *i*-PrOH (2.3 mL) was added, at room temperature, Ti(O*i*-Pr)$_4$ (0.83 mL, 2.80 mmol, 2.0 equiv). The mixture was warmed to 80 °C and stirred overnight. After 14h the reaction was diluted with water (5 mL) and Et_2O (10 mL). The aqueous phase was extracted with Et_2O (3 x 10 mL). The combined organic layers were washed with brine, dried over Na_2SO_4, filtered and evaporated. The crude was purified by FCC (CyH:AcOEt, 100:0 to 98:2) enyne **1b** as a colorless oil (306 mg, 0.894 mmol, 64% yield).

Spectroscopic data matched those reported in the literature. [7]

^{1}H NMR (300 MHz, $CDCl_3$) δ 7.38 – 7.25 (m, 4H), 7.27 – 7.16 (m, 2H), 6.51 (d, *J* = 15.7 Hz, 1H), 6.03 (dt, *J* = 15.5, 7.7 Hz, 1H), 5.09 (hept, *J* = 6.3 Hz, 2H), 2.94 (dd, *J* = 7.6, 1.2 Hz, 2H), 2.82 (d, *J* = 2.7 Hz, 2H), 2.04 (t, *J* = 2.7 Hz, 1H), 1.25 (d, *J* = 2.5 Hz, 6H), 1.23 (d, *J* = 2.5 Hz, 6H).

^{13}C NMR (75 MHz, $CDCl_3$) δ 169.4, 137.2, 134.6, 128.6, 127.6, 126.4, 123.5, 79.2, 71.6, 69.3, 57.0, 35.8, 22.9, 21.8, 21.7.

(*E*)-2-Cinnamyl-2-(prop-2-yn-1-yl)propane-1,3-diol (S1)

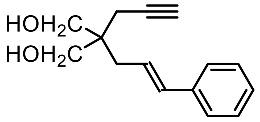

Under argon, to a flame dried 50 mL two-necked flask equipped with a stirring bar and charged with $LiAlH_4$ (159 mg, 4.19 mmol, 4.0 equiv) was added anhydrous Et_2O (15 mL). The suspension was cooled with an ice/water bath and a solution of **1a** (300 mg, 1.05 mmol) in Et_2O (6 mL) was added. The mixture was allowed to warm up to room temperature and was left stirring until starting material consumption. After 4h the reaction was quenched: H_2O (0.15 mL) was added dropwise, then a 15 w% aqueous solution of NaOH (0.15 mL) and H_2O (0.15 mL). The mixture was stirred for 15 min and finally $MgSO_4$ was added allowing it to stir for another 15min. The solution was filtered through a celite pad and washed with Et_2O. The crude was purified by FCC (CyH:AcOEt, 90:10 to 70:30) enyne **S1** as a white solid (190 mg, 0.825 mmol, 79% yield).

Spectroscopic data matched those reported in the literature.

^{1}H NMR (500 MHz, $CDCl_3$) δ 7.38 – 7.33 (m, 2H), 7.33 – 7.29 (m, 2H), 7.25 – 7.18 (m, 1H), 6.56 – 6.44 (m, 1H), 6.23 (dt, *J* = 15.6, 7.7 Hz, 1H), 3.79 – 3.54 (m, 4H), 2.35 – 2.30 (m, 4H), 2.19 (d, *J* = 16.3 Hz, 2H), 2.07 (t, *J* = 2.7 Hz, 1H).

^{13}C NMR (126 MHz, $CDCl_3$) δ 137.4, 133.78 128.7, 127.5, 126.3, 125.0, 81.1, 71.2, 67.7, 42.9, 35.4, 21.8.

(*E*)-(4,4-Bis(methoxymethyl)hept-1-en-6-yn-1-yl)benzene (1c)

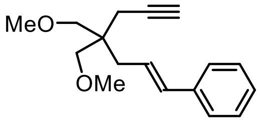

Under argon, to a flame dried 50 mL two-necked flask equipped with a stirring bar and charged with NaH (96.4 mg, 2.41 mmol, 3 equiv) was added anhydrous THF (6 mL). The mixture was cooled to 0 °C and diol **S1** (185 mg, 0.803 mmol, 1 equiv) was added dropwise as a solution in THF (2 mL). After 15 min iodomethane (150 µL, 2.41

mmol, 3 equiv) was slowly added at the same temperature. Reaction was allowed to warm up to room temperature and was left stirring overnight. After 16 h, a saturated NH_4Cl aqueous solution was added (10 mL). The aqueous phase was then extracted with Et_2O (3 x 15 mL). The combined organic layers were washed with brine, dried over Na_2SO_4, filtered and evaporated. The crude was purified by FCC (CyH:AcOEt, 99:1) enyne **1c** as a colorless oil (202 mg, 0.803 mmol, 98% yield).
Spectroscopic data matched those reported in the literature.[8]
^{1}H NMR (500 MHz, $CDCl_3$) δ 7.38 – 7.35 (m, 2H), 7.33 – 7.29 (m, 2H), 7.24 – 7.19 (m, 1H), 6.46 (d, *J* = 15.8 Hz, 1H), 6.22 (dt, *J* = 15.6, 7.7 Hz, 1H), 3.36 (s, 6H), 3.33 – 3.27 (m, 4H), 2.34 (dd, *J* = 7.7, 1.3 Hz, 2H), 2.27 (d, *J* = 2.7 Hz, 2H), 2.02 (t, *J* = 2.7 Hz, 1H).
^{13}C NMR (126 MHz, $CDCl_3$) δ 137.9, 133.3, 128.6, 127.2, 126.2, 125.9, 81.4, 74.5, 70.4, 59.5, 42.6, 35.5, 22.4.
HRMS (ESI +) calculated for $[C_{17}H_{22}NaO_2]^{+\cdot}$ $[M+Na]^{+\cdot}$ 281.1512 m/z; found 281.1523 m/z.

N-(*E*)-Cinnamyl-4-methyl-N-(prop-2-yn-1-yl)benzenesulfonamide (1d)

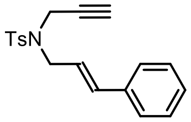

Following a modified literature procedure.[9] To a flame-dried flask equipped with a stirring bar were sequentially added triphenylphosphine (1.95 g, 7.45 mmol, 2.0 equiv), 4-methyl-N- (prop-2-yn-1-yl)benzenesulfonamide (936 mg, 4.47 mmol, 1.2 equiv), anhydrous THF (12 mL) and cinnamyl alcohol (0.48 mL, 3.37 mmol). The mixture was cooled to 0 °C and diethyl azodicarboxylate (2.9 mL, 7.45 mmol, 2.0 equiv) was added dropwise. Then the reaction was stirred at room temperature for 14h. After complete conversion of the starting material, the solvent was removed under reduced pressure and the residue was dissolved in ethyl acetate, washed with brine, dried over MgSO4, filtered and evaporate. The crude was purified by FCC (CyH:AcOEt, 9:1) and the product was obtained as an off-white solid. The columned fraction was then recrystallized from refluxing CyH obtaining enyne **1d** as a white solid (762 mg, 2.34 mmol, 63 % yield).
Spectroscopic data matched those reported in the literature.[9]
^{1}H NMR (300 MHz, $CDCl_3$) δ 7.83 – 7.72 (m, 2H), 7.37 – 7.22 (m, 7H), 6.58 (d, *J* = 15.8 Hz, 1H), 6.08 (dt, *J* = 15.8, 6.9 Hz, 1H), 4.13 (d, *J* = 2.4 Hz, 2H), 4.00 (dd, *J* = 6.9, 1.1 Hz, 2H), 2.44 (s, 3H), 2.05 (t, *J* = 2.5 Hz, 1H).
^{13}C NMR (75 MHz, $CDCl_3$) δ 143.6, 136.1, 134.9, 129.5, 128.6, 128.1, 127.8, 126.6, 122.9, 73.8, 48.6, 35.9, 21.6.

Dimethyl (*E*)-2-(Prop-2-yn-1-yl)-2-(3-(4-(trifluoromethyl)phenyl)allyl)malonate (1e)

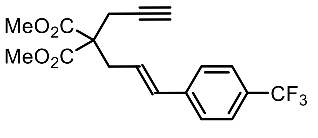

Prepared following general procedure GP3, using (*E*)-3-(4-(trifluoromethyl)phenyl)prop-2-en-1-ol (360 mg, 1.73 mmol). Allyl bromide was obtained as a colorless oil (350 mg, 1.32 mmol, 74%) and was used in the following step with propargyl malonate (134 µL, 0.880 mmol, 1.0 equiv). Enyne **1e** was

obtained as a colorless oil (280 mg, 0.790 mmol, 90% yield) after FCC (CyH:AcOEt dry load silica, 100:0 to 90:10).
Spectroscopic data matched those reported in the literature.[8]
^{1}H NMR (300 MHz, $CDCl_3$) δ 7.54 (d, *J* = 8.2 Hz, 2H), 7.42 (d, *J* = 8.2 Hz, 2H), 6.55 (d, *J* = 15.7 Hz, 1H), 6.13 (dt, *J* = 15.6, 7.6 Hz, 1H), 3.77 (s, 6H), 2.99 (dd, *J* = 7.6, 1.3 Hz, 2H), 2.85 (d, *J* = 2.7 Hz, 2H), 2.07 (t, *J* = 2.7 Hz, 1H).
^{13}C NMR (75 MHz, $CDCl_3$) δ 170.2, 140.5, 133.5, 126.6, 126.3, 125.6 (q, *J* = 3.8 Hz), 78.8, 72.0, 57.3, 53.1, 36.1, 23.3.
^{19}F NMR (282 MHz, $CDCl_3$) δ -62.5.

Dimethyl (*E*)-2-(3-(4-Nitrophenyl)allyl)-2-(prop-2-yn-1-yl)malonate (1f)

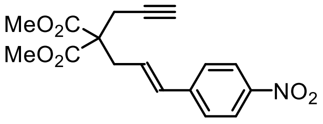

Prepared following general procedure GP3, using (*E*)-3-(4-nitrophenyl)prop-2-en-1-ol (400 mg, 2.23 mmol). Allyl bromide was obtained as a yellow oil (410 mg, 1.69 mmol, 76%) and was used in the following step with propargyl malonate (170 µL, 1.11 mmol, 1.0 equiv). Enyne **1f** was obtained as a yellow solid (230 mg, 0.69 mmol, 62% yield) after FCC (CyH:AcOEt dry load silica, 100:0 to 90:10).
Spectroscopic data matched those reported in the literature. [10]
M.p. 68 ºC
^{1}H NMR (300 MHz, $CDCl_3$) δ 8.21 – 8.09 (m, 2H), 7.51 – 7.37 (m, 2H), 6.59 (d, *J* = 15.8 Hz, 1H), 6.33 – 6.14 (m, 1H), 3.77 (s, 6H), 3.01 (dd, *J* = 7.6, 1.2 Hz, 2H), 2.85 (d, *J* = 2.7 Hz, 2H), 2.08 (t, *J* = 2.7 Hz, 1H).
^{13}C NMR (75 MHz, $CDCl_3$) δ 170.1, 147.1, 143.4, 132.8, 128.8, 127.0, 124.1, 78.6, 72.1, 57.2, 53.1, 36.3, 23.4.
HRMS (ESI +) calculated for $[C_{17}H_{17}NNaO_6]^+$ $[M+Na]^+$ 354.0948 m/z; found 354.0950 m/z.

Dimethyl (*E*)-2-(3-(4-Chlorophenyl)allyl)-2-(prop-2-yn-1-yl) (1g)

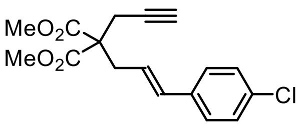

Prepared following general procedure **GP3**, using (*E*)-3-(4-chlorophenyl)prop-2-en-1-ol (200 mg, 1.19 mmol). Allyl bromide was obtained as a colorless oil (200 mg, 0.864 mmol, 76%) and was used in the following step with propargyl malonate (89 µL, 0.588 mmol, 1.0 equiv). Enyne **1g** was obtained as a white solid (150 mg, 0.468 mmol, 80% yield) after FCC (CyH:AcOEt dry load silica, 100:0 to 90:10).
Spectroscopic data matched those reported in the literature.[8]
M.p. 84–86 ºC.
^{1}H NMR (300 MHz, $CDCl_3$) δ 7.26 – 7.24 (m, 4H), 6.47 (dt, *J* = 15.7, 1.3 Hz, 1H), 5.99 (dt, *J* = 15.5, 7.7 Hz, 1H), 3.76 (s, 5H), 2.95 (dd, *J* = 7.7, 1.3 Hz, 2H), 2.84 (d, *J* = 2.7 Hz, 2H), 2.06 (t, *J* = 2.6 Hz, 1H).

^{13}**C NMR** (75 MHz, $CDCl_3$) δ 170.2, 135.6, 133.6, 133.3, 128.8, 127.6, 124.1, 78.9, 71.9, 57.3, 53.0, 36.0, 23.2.

Dimethyl (*E*)-2-(3-(4-Methoxyphenyl)allyl)-2-(prop-2-yn-1-yl)malonate (1h)

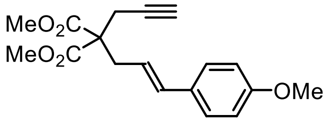

Prepared following general procedure GP3, using (*E*)-3-(4-methoxyphenyl)prop-2-en-1-ol (300 mg, 1.83 mmol). Allyl bromide was obtained as a colorless oil (350 mg, 1.54 mmol, 84%) and was used in the following step with propargyl malonate (152 μL, 0.999 mmol, 1.0 equiv). Enyne **1h** was obtained as a colorless oil (200 mg, 0.632 mmol, 63% yield) after FCC (CyH:AcOEt dry load silica, 100:0 to 90:10). Spectroscopic data matched those reported in the literature.[10]

^{1}**H NMR** (300 MHz, $CDCl_3$) δ 7.31 – 7.26 (m, 1H), 7.25 – 7.23 (m, 1H), 6.87 – 6.79 (m, 2H), 6.46 (d, *J* = 15.7 Hz, 1H), 5.84 (dt, *J* = 15.5, 7.6 Hz, 1H), 3.80 (s, 3H), 3.76 (s, 6H), 2.94 (dd, *J* = 7.7, 1.2 Hz, 2H), 2.84 (d, *J* = 2.7 Hz, 2H), 2.05 (t, *J* = 2.7 Hz, 1H).

^{13}**C NMR** (75 MHz, $CDCl_3$) δ 170.4, 134.2, 127.6, 120.9, 114.1, 79.0, 71.7, 57.44 55.4, 53.0, 36.0, 23.0.

Dimethyl (*E*)-2-(3-(3-Methoxyphenyl)allyl)-2-(prop-2-yn-1-yl)malonate (1i)

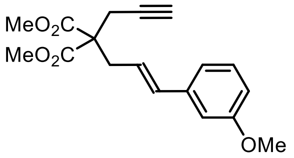

Prepared following general procedure **GP3**, using (*E*)-3-(3-methoxyphenyl)prop-2-en-1-ol (871 mg, 5.30 mmol). Allyl bromide was obtained as a colorless oil (995 mg, 5.30 mmol, 83%) and was used in the following step with propargyl malonate (152 μL, 0.999 mmol, 1.0 equiv). Enyne **1i** was obtained as a colorless oil (239 mg, 0.755 mmol, 44% yield) after FCC (CyH:AcOEt dry load silica, 100:0 to 90:10).

M.p. 58 ºC.

^{1}**H NMR** (500 MHz, $CDCl_3$) δ 7.21 (t, *J* = 7.9 Hz, 1H), 6.93 (dt, *J* = 7.6, 1.3 Hz, 1H), 6.86 (dd, *J* = 2.6, 1.6 Hz, 1H), 6.78 (ddd, *J* = 8.2, 2.6, 0.9 Hz, 1H), 6.49 (dt, *J* = 15.8, 1.4 Hz, 1H), 6.00 (dt, *J* = 15.5, 7.7 Hz, 1H), 3.81 (s, 3H), 3.76 (s, 6H), 2.96 (dd, *J* = 7.7, 1.3 Hz, 2H), 2.85 (d, *J* = 2.7 Hz, 2H), 2.06 (t, *J* = 2.7 Hz, 1H).

^{13}**C NMR** (126 MHz, $CDCl_3$) δ 170.3, 159.9, 138.6, 134.7, 129.6, 123.6, 119.1, 113.2, 112.0, 79.0, 71.8, 57.4, 55.4, 53.0, 36.0, 23.1.

HRMS (ESI +) calculated for $[C_{18}H_{20}NaO]^{+\cdot}$ $[M+Na]^{+\cdot}$ 339.1203 m/z; found 339.1203 m/z.

Dimethyl (*E*)-2-(3-(3-Nitrophenyl)allyl)-2-(prop-2-yn-1-yl)malonate (1j)

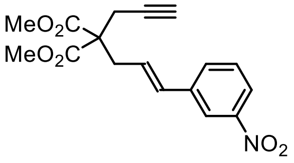

Prepared following general procedure GP3, using (*E*)-3-(3-nitrophenyl)prop-2-en-1-ol (432 mg, 2.41 mmol). Allyl bromide was obtained as a yellow solid (350 mg, 1.54 mmol, 84%) and was used in the following step with propargyl malonate (110 μL, 0.723 mmol, 1.0 equiv).

Enyne **1j** was obtained as pale yellow solid (193 mg, 0.583 mmol, 80% yield) after FCC (CyH:AcOEt dry load silica, 100:0 to 95:5).

M.p. 87–90 ºC

^{1}H NMR (300 MHz, $CDCl_3$) δ 8.16 (t, *J* = 1.9 Hz, 1H), 8.07 (ddd, *J* = 8.1, 2.2, 1.0 Hz, 1H), 7.63 (d, *J* = 7.8 Hz, 1H), 7.46 (t, *J* = 7.9 Hz, 1H), 6.58 (d, *J* = 15.7 Hz, 1H), 6.18 (dt, *J* = 15.6, 7.6 Hz, 1H), 3.77 (s, 6H), 3.00 (dd, *J* = 7.6, 1.2 Hz, 2H), 2.85 (d, *J* = 2.7 Hz, 2H), 2.09 (t, *J* = 2.7 Hz, 1H).

^{13}C NMR (75 MHz, $CDCl_3$) δ 170.1, 148.7, 138.8, 132.5, 132.2, 129.6, 127.0, 122.3, 121.1, 78.7, 72.1, 57.2, 53.1, 36.1, 23.3.

HRMS (ESI +) calculated for $[C_{17}H_{17}NNaO_6]^{+\cdot}$ $[M+Na]^{+\cdot}$ 354.0948 m/z; found 354.0952 m/z.

Dimethyl (*E*)-2-(3-(2-Methoxyphenyl)allyl)-2-(prop-2-yn-1-yl)malonate (1k)

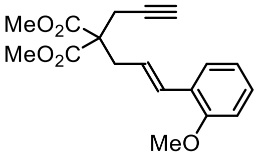

Prepared following general procedure GP3, using (*E*)-3-(2-methoxyphenyl)prop-2-en-1-ol (300 mg, 1.83 mmol). Allyl bromide was obtained as a pale yellow oil (380 mg, 1.67 mmol, 82%) and was used in the following step with propargyl malonate (170 µL, 1.12 mmol, 1.0 equiv). Enyne **1k** was obtained as a colorless oil (342 mg, 1.08 mmol, 97% yield) after FCC (CyH:AcOEt dry load silica, 100:0 to 90:10).

M.p. 60 ºC.

^{1}H NMR (300 MHz, $CDCl_3$) δ 7.36 (dd, *J* = 7.6, 1.7 Hz, 1H), 7.24 – 7.16 (m, 1H), 6.93 – 6.77 (m, 3H), 5.99 (dt, *J* = 15.6, 7.7 Hz, 1H), 3.82 (s, 3H), 3.76 (s, 6H), 2.98 (dd, *J* = 7.7, 1.3 Hz, 2H), 2.85 (d, *J* = 2.7 Hz, 2H), 2.05 (t, *J* = 2.7 Hz, 1H).

^{13}C NMR (75 MHz, $CDCl_3$) δ 170.4, 156.7, 129.8, 128.7, 127.0, 126.4, 123.8, 120.7, 111.0, 79.0, 71.7, 57.6, 55.60, 52.9, 36.4, 23.1.

HRMS (ESI +) calculated for $[C_{18}H_{20}NaO_5]^{+\cdot}$ $[M+Na]^{+\cdot}$ 339.1203 m/z; found 339.1218 m/z.

Dimethyl (*E*)-2-(3-(2-Fluorophenyl)allyl)-2-(prop-2-yn-1-yl)malonate (1l)

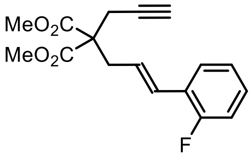

Prepared following general procedure GP3, using (*E*)-3-(2-fluorophenyl)prop-2-en-1-ol (191 mg, 1.25 mmol). Allyl bromide was obtained as a colorless oil (250 mg, 1.16 mmol, 93%) and was used in the following step with propargyl malonate (120 µL, 0.789 mmol, 1.0 equiv). Enyne **1l** was obtained as a colorless oil (236 mg, 0.775 mmol, 98% yield) after FCC (CyH:AcOEt dry load silica, 100:0 to 90:10).

^{1}H NMR (300 MHz, $CDCl_3$) δ 7.41 (td, *J* = 7.7, 1.8 Hz, 1H), 7.25 – 7.18 (m, 1H), 7.13 – 6.98 (m, 2H), 6.69 (d, *J* = 15.9 Hz, 1H), 6.12 (dt, *J* = 15.7, 7.7 Hz, 1H), 3.79 (s, 6H), 3.01 (dd, *J* = 7.6, 1.3 Hz, 2H), 2.88 (d, *J* = 2.7 Hz, 2H), 2.09 (t, *J* = 2.7 Hz, 1H).

^{13}C NMR (75 MHz, $CDCl_3$) δ 170.2, 129.0, 127.6, 127.3, 126.1, 124.2, 115.9, 115.6, 78.8, 71.9, 57.4, 53.0, 36.4, 23.1.

^{9}F NMR (282 MHz, $CDCl_3$) δ -118.4.

HRMS (ESI +) calculated for $[C_{17}H_{17}FNaO_4]^{+\cdot}$ $[M+Na]^{+\cdot}$ 327.1003 m/z; found 327.1005 m/z.

Dimethyl (*E*)-2-(3-(Naphthalen-1-yl)allyl)-2-(prop-2-yn-1-yl)malonate (1m)

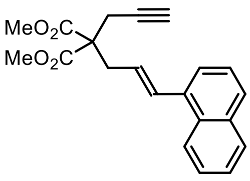

Prepared following general procedure GP3, using (*E*)-3-(naphthalen-1-yl)prop-2-en-1-ol (191 mg, 1.25 mmol). Allyl bromide was obtained as a white solid (250 mg, 1.16 mmol, 93%) and was used in the following step (this time as 1.3 equiv) with propargyl malonate (120 µL, 0.789 mmol, 1.0 equiv). Enyne **1m** was obtained as a white solid (261 mg, 0.776 mmol, 98% yield) after FCC (CyH:AcOEt dry load silica, 100:0 to 90:10).

Spectroscopic data matched those reported in the literature.[8]

^{1}H NMR (300 MHz, $CDCl_3$) δ 8.13 – 8.01 (m, 1H), 7.88 – 7.80 (m, 1H), 7.77 (d, *J* = 8.0 Hz, 1H), 7.56 – 7.37 (m, 5H), 7.34 – 7.26 (m, 1H), 6.02 (dt, *J* = 15.4, 7.7 Hz, 1H), 3.78 (s, 6H), 3.10 (dd, *J* = 7.7, 1.3 Hz, 2H), 2.92 (d, *J* = 2.7 Hz, 2H), 2.12 (t, *J* = 2.7 Hz, 1H).

^{13}C NMR (75 MHz, $CDCl_3$) δ 170.4, 135.0, 132.5, 128.6, 128.1, 126.6, 126.2, 125.9, 125.7, 124.2, 123.9, 79.0, 71.9, 57.4, 53.0, 36.4, 23.2.

HRMS (ESI +) calculated for $[C_{17}H_{17}FNaO_4]^{+\cdot}$ $[M+Na]^{+\cdot}$ 327.1003 m/z; found 327.1005 m/z.

Dimethyl (*E*)-2-(3-(Naphthalen-1-yl)allyl)-2-(prop-2-yn-1-yl)malonate (1n)

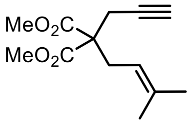

Under argon, to suspension of NaH (170 mg, 60% wt, 4.25 mmol, 1.1 equiv) in anhydrous THF (20mL) at room temperature, was added dropwise propargyl malonate (0.60 mL, 3.95 mmol) and the mixture was left stirring at the same temperature for 15 min. Prenyl bromide (0.58 mL, 95% wt, 4.7 mmol, 1.2 equiv) was then added dropwise at the same temperature and the resulting suspension was stirred for 24 h. The reaction was quenched by slow addition of an aqueous saturated NH_4Cl solution (10 mL) and extracted with Et_2O (15 mL x 3). The combined organic layers were washed with brine, dried over $MgSO_4$, filtered and the solvent was evaporated. The crude was purified by FCC (CyH:AcOEt, 100:0 slowly to 95:5) enyne **1n** as a colorless oil (867 mg, 0.364 mmol, 92% yield).

Spectroscopic data matched those reported in the literature.[10]

^{1}H NMR (300 MHz, $CDCl_3$) δ 4.94 – 4.84 (m, 1H), 3.75 – 3.70 (m, 6H), 2.82 – 2.73 (m, 4H), 1.99 (td, *J* = 2.7, 0.8 Hz, 1H), 1.69 (s, 3H), 1.64 (s, 3H).

^{13}C NMR (75 MHz, $CDCl_3$) δ 170.63, 137.11, 117.07, 79.40, 71.32, 57.29, 52.84, 30.90, 26.19, 22.65, 18.06.

Dimethyl (*E*)-2-(3,7-Dimethylocta-2,6-dien-1-yl)-2-(prop-2-yn-1-yl)malonate (1o)

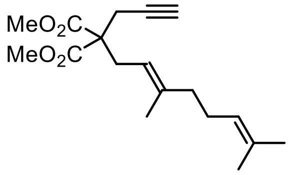

Prepared following general procedure GP3, using (*E*)-3,7-dimethylocta-2,6-dien-1-ol (500 mg, 3.25 mmol). Geranyl bromide was obtained as a colorless oil (733 mg, 3.17 mmol, 98%) and was used in the following step (this time as 1.3 equiv) with propargyl malonate (360 µL, 2.35 mmol, 1.0 equiv). Enyne **1o** was obtained as a white solid (612 mg, 2.00 mmol, 85% yield) after FCC (CyH:AcOEt dry load silica, 100:0 to 90:10).

Spectroscopic data matched those reported in the literature.[10]

^{1}H NMR (300 MHz, $CDCl_3$) δ 4.94 – 4.84 (m, 1H), 3.75 – 3.70 (m, 6H), 2.82 – 2.73 (m, 4H), 1.99 (td, J = 2.7, 0.8 Hz, 1H), 1.69 (s, 3H), 1.64 (s, 3H).

^{13}C NMR (75 MHz, $CDCl_3$) δ 170.63, 137.11, 117.07, 79.40, 71.32, 57.29, 52.84, 30.90, 26.19, 22.65, 18.06.

4. Nucleophile addition to 1,6-enynes

4.1 Optimization

High-throughput experiments

In order to benefit from the two-component combinatorial nature of the HCDC approach, HTE experiments were performed to screen the best solvent and combination of Au(I) complex and chiral metal salt (Figure S2).

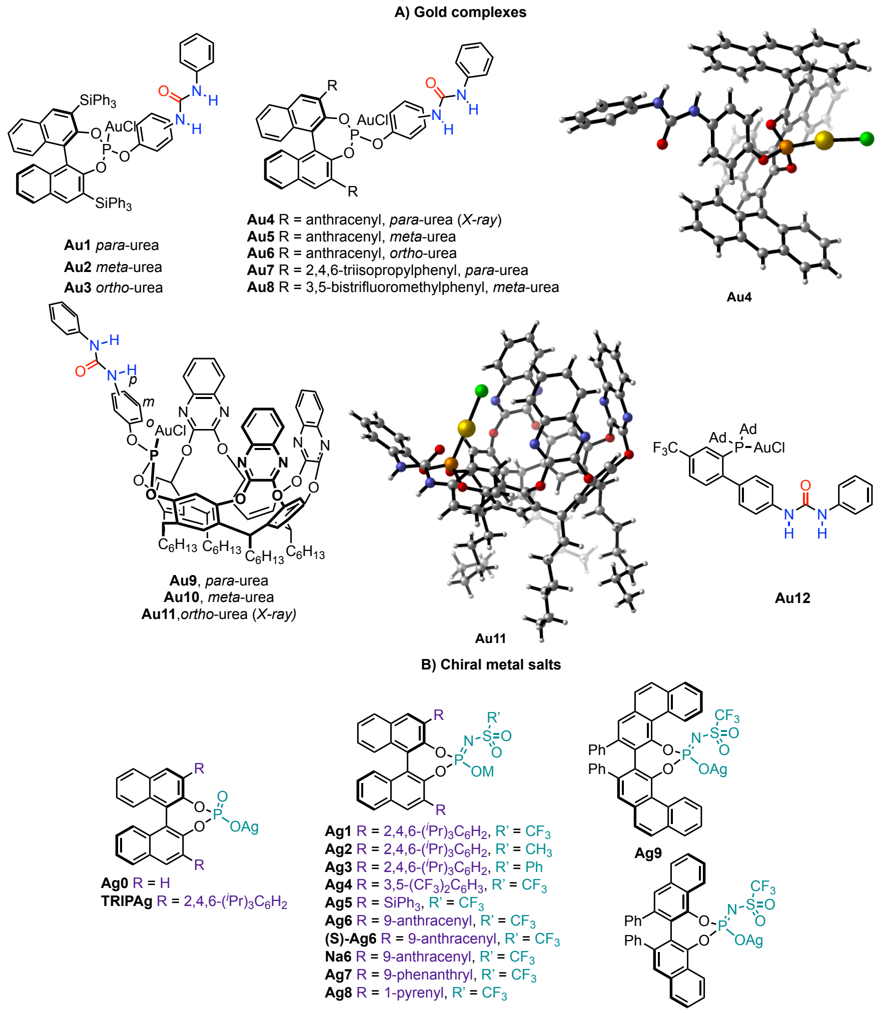

Figure S2 Library of Au(I) complexes and chiral metal salts.

HTE procedure used for solvent screening in the indole addition to 1,6-enyne 1a

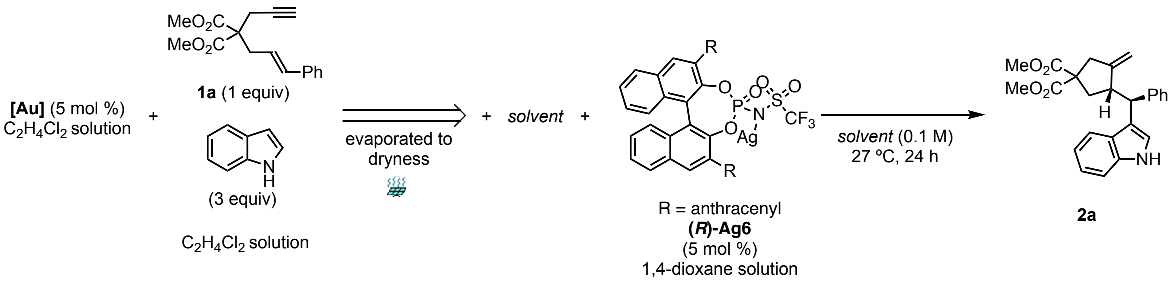

*Scheme S1 General HTE procedure used for solvent testing in the indole addition to 1,6-enyne **1a**.*

7 different *solvents* tested (columns 1 to 7 in plate)

6 different **[Au]** tested (rows A to F in plate)

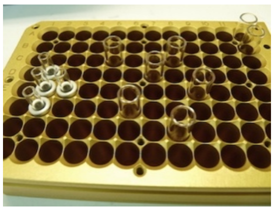

Figure S3 Plate used in HTE experiment.

The gold complexes were dissolved in dry $C_2H_4Cl_2$ in a 0.005 M solution (3.5 µmol). 70 µL of the solution (7 mol %, 0.7 µmol) were added to a 1 mL reaction vial. To the reaction vials containing the gold complexes were added 100 µL of a stock $C_2H_4Cl_2$ solution of 1,6-enyne 1a (0.1 M, 10 µmol, 1 equiv) and indole (0.3 M, 30 µmol, 3 equiv). The solvent was evaporated in a centrifuge under vacuum (Genevac apparatus). Then, 65 µL of the corresponding solvent was added in each column of the reaction plate. Finally, 35 µL of the solution of silver salt (R)-Ag6 in the corresponding solvent (0.02 M, 7 mol %, 0.7 µmol) were added. The reaction plate (32 vial plates were used) was capped and the reactions were left stirring at 27 ºC for 24 hour. After this time, 500 µL of a stock solution containing naphthalene as internal standard (0.005 M solution, 2.5 µmol, 0.25 equiv) and triethylamine (0.004 M, 2 µmol, 0.2 equiv) were added to each reaction vial. For the analysis plate preparation, 20 µL of the diluted reaction solution were added to 500 µL of CH_3CN and results were analyzed by UPLC and UPC2.

Table S1 High-throughput screening of solvents for the gold(I)-catalyzed indole addition to 1,6-enynes.[a]

	$CF_3C_6H_5$	$CH_3C_6H_5$	$C_2H_4Cl_2$	$CHCl_3$	CPME	1,4-dioxane
Au4	88	87	99	90	92	93
Au5	77	71	75	40	79	81
Au6	75	50	73	34	63	5
Au9	73	78	100	69	83	98
Au10	74	69	82	41	78	53
Au11	46	36	57	8	37	0

[a] *Yields calculated using naphthalene as internal standard in the UPC2. Color code: grey; ≤ 60%, yellow; 61-80%, green; > 80% .*

Table S2 High-throughput screening of solvents for the gold(I)-catalyzed nucleophile addition to enynes, er shown.[a]

	$CF_3C_6H_5$	$CH_3C_6H_5$	DCE	$CHCl_3$	CPME	1,4-Dioxane
Au4	92.5:7.5	95.5:4.5	87:13	96:4	96.5:3.5	97:3
Au5	46.5:53.5	57:43	58.5:41.5	55:45	69:31	71:29
Au6	44:56	55:45	43:57	53:47	34.5:65.5	35.5:64.5
Au9	66:34	84.5:15.5	72.5:27.5	78:22	88:12	94:6
Au10	48:52	46.5:53.5	48.5:51.5	52.5:47.5	52.5:47.5	44:56
Au11	47.5:52.5	41:59	53:47	48:52	37:63	-

[a] *er determined by UPC2 on chiral stationary phase. IB column (150 × 4.6 mm, 3 µm), CO_2:MeOH gradient method (100:0 for 1 min, then increasing to 60:40 over 4 min, hold for 1 min at 60:40, then decreasing to 100:0 over 0.25 min and hold for 1.25 min), 3.0 mL/min, 35 ºC, BPR 150 bar, 230 nm): naphthalene (2.39 min), **1a** (2.49 min), indole (3.60 min), **2a** en1 (4.55 min), **2a** en2 (4.69 min). Color code: dark green; >80% ee, light green; 62-80% ee, blue; 42-60% ee, yellow; 22-40% ee and grey; ≤ 20% ee.*

HTE procedure used for silver salt screening in the indole addition to 1,6-enyne 1a

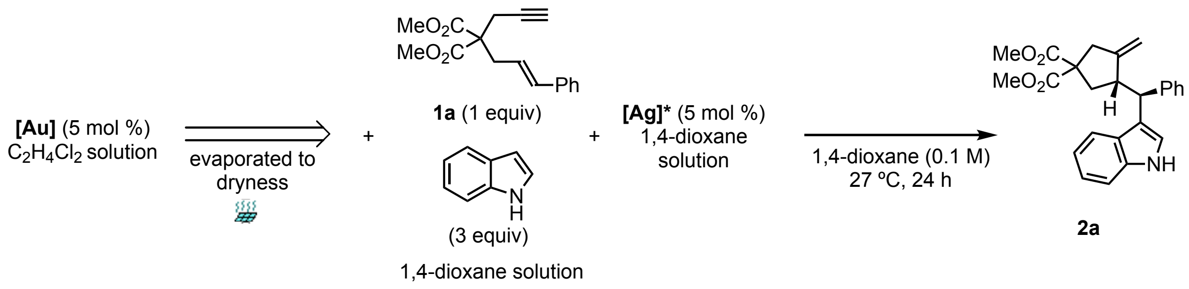

*Scheme S2 General HTE procedure used for silver salt testing in the indole addition to 1,6-enyne **1a**.*

12 different **[Ag]*** tested (columns 1 to 12 in plate)

8 different **[Au]** tested (rows A to H in plate)

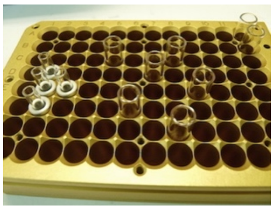

Figure S4 Plate used in HTE experiment.

The gold complexes were dissolved in dry $C_2H_4Cl_2$ in a 0.005 M solution (3.5 µmol). 50 µL of the solution (5 mol %, 0.25 µmol) were added to a 1 mL reaction vial. The solvent was evaporated in a centrifuge under vacuum (Genevac apparatus). To the reaction vials containing the gold complexes were added 20 µL of a stock dioxane solution of 1,6-enyne 1a (0.250 M, 5 µmol, 1 equiv) and indole (0.75 M, 15 µmol, 3 equiv). Finally, 30 µL of a solution of the corresponding silver salt in dry dioxane (0.008 M, 5 mol %, 0.25µmol) were added in each column of the reaction plate. The reaction plate (96 vial plates were used) was capped and the reactions were left stirring at 27 ºC for 24 hour. After this time, 463 µL of a stock solution containing naphthalene as internal standard (0.0027 M solution, 1.25 µmol, 0.25 equiv) and triethylamine (0.002 M, 1 µmol, 0.2 equiv) were added to each reaction vial. For the analysis plate preparation, 20 µL of the diluted reaction solution were added to 500 µL of CH_3CN and results were analyzed by UPLC and UPC2.

Table S3 High-throughput screening of gold (I) complexes and chiral silver salts.[a]

	Ag0	*TRIPAg*	*Ag1*	*Ag3*	*Ag4*	*Ag5*	*Ag6*	*Ag8*	*Ag9*	*Ag10*
Au1	6	0	76	3	66	64	72	68	53	55
Au4	6	0	78	40	71	71	79	79	76	75
Au5	9	0	61	4	40	57	66	56	43	49
Au6	0	0	4	0	9	0	6	7	34	33
Au7	0	0	33	0	17	13	47	42	61	55
Au9	0	0	80	3	50	44	70	62	66	64
Au10	2	0	17	0	12	16	61	36	66	60
Au11	0	0	3	0	0	0	1	4	5	10

[a] *Yield for the solvent screening calculated using naphthalene as internal standard in the UPC2. Color code: grey; ≤ 60%, green; > 61%.*

Table S4 High-throughput screening of gold (I) complexes and chiral silver salts, er shown.[a]

	Ag0	*TRIPAg*	*Ag1*	*Ag3*	*Ag4*	*Ag5*	*Ag6*	*Ag8*	*Ag9*	*Ag10*
Au1	58:42	-	92.5:7.5	-	68:32	72.5:27.5	90:10	84:16	55:45	65:35
Au4	71.5:28.5	-	99:1	95.5:4.5	93.5:6.5	88:12	98:2	95.5:4.5	87.5:12.5	87.5:12.5
Au5	24.5:75.5	-	71:29	48.5:51.5	62.5:37.5	55.5:44.5	73:27	53:47	48:52	66:34
Au6	-	-	68:32	-	31.5:68.5	-	42.5:57.5	45:55	46:54	38.5:61.5
Au7	-	-	62:38	-	67.5:32.5	55:45	61.5:38.5	55:45	64:36	48:52
Au9	-	-	90.5:9.5	-	84:16	30:70	94:6	90:10	80.5:19.5	64.5:35.5
Au10	-	-	51.5:48.5	-	62:38	36:64	47:53	33.5:66.5	64:36	54.5:45.5
Au11	-	-	51.5:48.5	-	-	-	-	41.5:58.5	45:55	30:70

[a] *er determined by UPC2 on chiral stationary phase. IB column (150 × 4.6 mm, 3 µm), CO_2:MeOH gradient method (100:0 for 1 min, then increasing to 60:40 over 4 min, hold for 1 min at 60:40, then decreasing to 100:0 over 0.25 min and hold for 1.25 min), 3.0 mL/min, 35 ºC, BPR 150 bar, 230 nm): naphthalene (2.39 min),* ***1a*** *(2.49 min), indole (3.60 min),* ***2a*** *en1 (4.55 min),* ***2a*** *en2 (4.69 min). Color code: dark green; >80% ee, light green; 62-80% ee, blue; 42-60% ee, yellow; 22-40% ee and grey; ≤ 20% ee.*

Further optimization experiments at laboratory scale

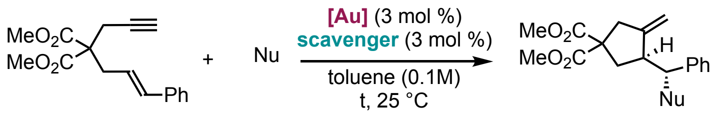

50 μmol
1a

2a

Table S5 Examples of match-missmatch scenario.[a]

Entry	[Au]	Scavenger	Nucleophile (equiv)	Time (h)	Yield (%)[a]	*er* [b]
**1**	Au4	(*R*)-Ag6	Indole (1.2 equiv)	44	82	95:5
**2**	Au4	(*S*)-Ag6	Indole (1.2 equiv)	44	61	61.5:38.5
**3**	Au4	(*R*)-Ag6	EtOH (4 equiv)	14	87	98:2
**4**	Au4	(*S*)-Ag6	EtOH (4 equiv)	14	85	57:43
**5**	Au1	(*R*)-Ag6	EtOH (4 equiv)	14	67	91:9
**6**	Au1	(*S*)-Ag6	EtOH (4 equiv)	14	53	47:53

[a] *Yield determined by ^{1}H-NMR analysis against dodecane as internal standard.*[b] *er determined by SFC on chiral stationary phase.*

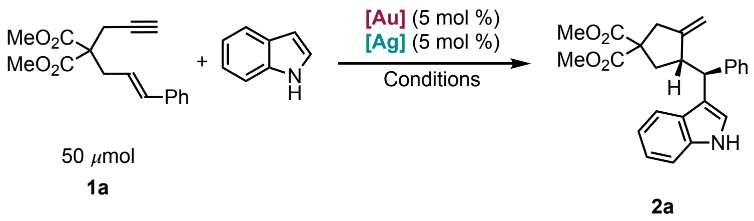

Table S6 Screening of conditions for the indole addition to 1,6-enynes.

Entry	[Au]	[Ag]	Solvent	Time (h)	Yield (%)[a]	*er* [b]
**1**	Au1	(*R*)-Ag6	Toluene	44	61	78:22
**2**	Au2	(*R*)-Ag6	Toluene	44	69	54:46
**3**	Au3	(*R*)-Ag6	Toluene	44	6	50:50
**4**	Au4	(*R*)-Ag6	Toluene	44	82	95:5
**5**	Au5	(*R*)-Ag6	Toluene	44	74	60:40
**6**	Au6	(*R*)-Ag6	Toluene	44	39	54:46
**7**	Au4	(*S*)-Ag6	Toluene	44	61	61.5:38.5
**8**	Au1	(*R*)-Ag1	1,4-dioxane	14	82	91:9
**9**	Au4	(*R*)-Ag1	1,4-dioxane	14	93[c]	>99:1
**10**	Au4	(*R*)-Ag6	1,4-dioxane	14	98	97.5:2.5
**11**	Au4	(*R*)-Ag8	1,4-dioxane	14	90	95:5
**12**	Au9	(*R*)-Ag6	1,4-dioxane	18	62	94:6
**13**	Au9	(*R*)-Ag1	1,4-dioxane	18	61	91:9
**14**	Au9	(*R*)-Ag8	1,4-dioxane	18	62	90:10

[a] Yield determined by ^{1}H-NMR analysis against dodecane as internal standard.[b] er determined by SFC on chiral stationary phase.[c] isolated yield.

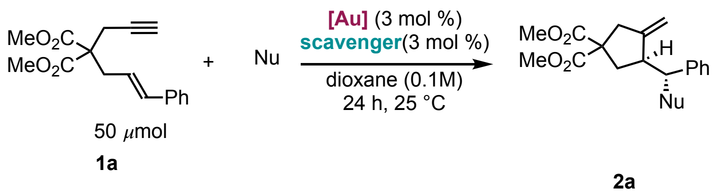

Table S7 Control experiments for the nucleophile addition

Entry	[Au]	Scavenger	Nucleophile (equiv)	Yield (%)[a]	*er* (%)[b]
**1**	Au4	-	Indole (1.2 equiv)	0	-
**2**	-	(*R*)-Ag1	Indole (1.2 equiv)	0	-
**3**	Au12	(*R*)-Ag1	Indole (1.2 equiv)	18	52:48
**4**	Au4	$AgSbF_6$	Indole (1.2 equiv)	76	92.5:7.5
**5**	Au4	$AgSbF_6$	EtOH(4 equiv)	78	85:15
**6**	Au4	NaBArF	EtOH (4 equiv)	5	60:40
**7**	Au4	AgNTf2	EtOH (4 equiv)	0	-

[a] *Yield determined by ^{1}H-NMR analysis against dodecane as internal standard.*[b] *er determined by SFC or HPLC on chiral stationary phase.*

4.2 Procedures and characterization

Nucleophile addition to 1,6-enynes

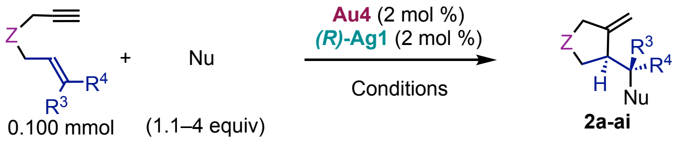

General procedure GP4: non-enantioselective nucleophile addition to 1,6-enynes

Under air and at room temperature, HPLC-grade CH_2Cl_2 or toluene (0.1 M) was added to a GCMS-vial containing the corresponding enyne (0.050 mmol). Then the desired nucleophile (1.1–4.0 equiv) was added followed by (acetonitrile)[(2-biphenyl)di-*tert*-butylphosphine]gold(I) hexafluoroantimonate (0.8 mg, 1.5 µmol, 0.03 equiv). The vial was capped and placed to stir at 23 ºC until TLC (CyH:AcOEt, 8:2), or GCMS analysis, indicated disappearance of the starting material. Then, the reaction mixture was quenched by the addition of triethylamine (ca 0.05 mL) and filtration through a silica-filled glass pipette. Volatiles were removed under reduced pressure and the crude product was purified by FCC or PTLC (silica, CyH:AcOEt or pentane:AcOEt eluent mixtures), obtaining the product in racemic form.

General procedure GP5: enantioselective nucleophile addition to 1,6-enynes

To a GMCS or MW vial containing a solution of enyne (0.100 mmol) and nucleophile (1.0–3.0 equiv) in solvent was added respectively a solution of complex **Au4** (2.3 mg, 2.0 µmol, 0.02 equiv) and a solution of **(*R*)-Ag1** (2.0mg, 2.0 µmol, 0.01 equiv) in 1,4-dioxane or toluene (0.1 M final concentration)

at the indicated temperature. The resulting mixture was stirred until starting material consumption, determined by TLC or GCMS analysis. The reaction was then quenched by addition of triethylamine (*ca* 0.1 mL) and filtered through a silica-filled glass pipette. Volatiles were removed under reduced pressure and the crude product was purified by FCC or PTLC (silica, CyH:AcOEt or pentane:AcOEt mixtures).

Note: for substrate **1d** with the *N*-Ts tether **Au7** and **(*R*)-Ag5** were used instead.

Dimethyl (*R*)-3-((*R*)-(1H-indol-3-yl)(phenyl)methyl)-4-methylenecyclopentane-1,1-dicarboxylate (2a)

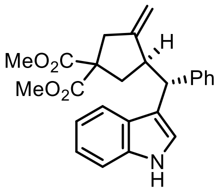

Prepared following general procedure **GP5** using dimethyl 2-cinnamyl-2-(prop-2-yn-1-yl)malonate (**1a**, 28.6 mg, 0.100 mmol) and indole (14.1 mg, 0.120 mmol, 1.2 equiv) as a nucleophile in 1,4-dioxane (1.0 mL, 0.1M), at 25 ºC for 6 h. The crude product was purified by FCC (silica, CyH:AcOEt 90:10 to 80:20). Compound **2a** was obtained as a white solid (40.1 mg, 0.994 mmol, 99% yield) with >99:1 *er*.

Spectroscopic data matched those reported in the literature.[11] The absolute configuration was assigned by comparison to **2v**.

^{1}H NMR (300 MHz, $CDCl_3$) δ 8.06 (brs, 1H), 7.54 (d, *J* = 8.0 Hz, 1H), 7.37 – 7.28 (m, 3H), 7.25 – 7.18 (m, 2H), 7.18 – 7.08 (m, 3H), 7.05 (td, *J* = 7.5, 1.1 Hz, 1H), 4.80 (s, 1H), 4.22 – 4.10 (m, 2H), 3.72 (s, 3H), 3.64 (s, 3H), 3.61 – 3.45 (m, 1H), 3.13 (dq, *J* = 15.9, 2.4 Hz, 1H), 2.97 – 2.85 (m, 1H), 2.76 (ddd, *J* = 13.6, 7.9, 1.5 Hz, 1H), 1.98 (dd, *J* = 13.7, 8.5 Hz, 1H).

^{13}C NMR (75 MHz, $CDCl_3$) δ 172.4, 172.2, 149.0, 144.5, 136.3, 128.6, 128.2, 127.3, 126.2, 122.1, 121.5, 119.50, 119.47, 118.7, 111.2, 110.2, 58.6, 52.9, 52.8, 48.1, 46.9, 41.9, 40.0.

SFC (IG (100 × 3 mm, 3 μm), 80:20 CO_2:*i*-PrOH, 1.5 mL/min, 35 ºC, BPR 150 bar, 280 nm): en2 (100%) 1.53 min.

$[\alpha]^{24.6}_D$ +2.8 (c 1.28, $CHCl_3$).

Dimethyl (*R*)-3-((*R*)-(5-Bromo-1H-indol-3-yl)(phenyl)methyl)-4-methylenecyclopentane-1,1-dicarboxylate (2b)

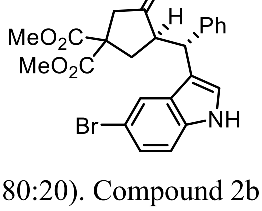

Prepared following general procedure GP5 using dimethyl 2-cinnamyl-2-(prop-2-yn-1-yl)malonate (1a, 28.6 mg, 0.100 mmol) and 5-bromoindole (23.5 mg, 0.120 mmol, 1.2 equiv) as a nucleophile in 1,4-dioxane (1.0 mL, 0.1M), at 25 ºC for 6 h. The crude product was purified by FCC (silica, CyH:AcOEt 90:10 to 80:20). Compound 2b was obtained as a white solid (44.8 mg, 0.929 mmol, 93% yield) with >99:1 *er*.

The absolute configuration was assigned by comparison to **2v**.

1H NMR (300 MHz, CDCl3) δ 8.16 (s, 1H), 7.65 (dt, J = 1.4, 0.7 Hz, 1H), 7.36 – 7.09 (m, 8H), 4.80 (t, J = 1.7 Hz, 1H), 4.11 (d, J = 2.3 Hz, 1H), 4.08 (d, J = 10.4 Hz, 1H), 3.73 (s, 3H), 3.65 (s, 3H), 3.57 –

3.35 (m, 1H), 3.17 – 2.99 (m, 1H), 2.91 (dd, J = 16.0, 1.5 Hz, 1H), 2.78 – 2.60 (m, 1H), 1.93 (dd, J = 13.7, 8.5 Hz, 1H).

13C NMR (75 MHz, CDCl3) δ 172.4, 172.2, 148.7, 144.0, 134.8, 129.0, 128.5, 128.4, 126.4, 125.0, 122.7, 121.9, 118.5, 112.84, 112.7, 110.3, 58.5, 53.0, 52.9, 47.9, 46.9, 41.8, 39.9.

HRMS (ESI +) calculated for [C25H25BrNO4] +· [M-H] +· 482.0961 m/z; found 482.0959 m/z.

SFC (IB-N (100 × 3 mm, 3 μm), 60:40 CO2:MeOH, 1.2 mL/min, 35 ºC, BPR 150 bar, 210 nm): en1 (major, 100%) 0.99 min.

[α]24.6D +38.7 (c 1.74, CHCl3).

Dimethyl (*R*)-3-((*R*)-(5-Methoxy-1H-indol-3-yl)(phenyl)methyl)-4-methylenecyclopentane-1,1-dicarboxylate (2c)

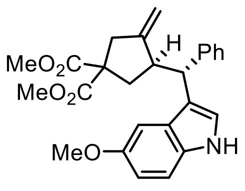

Prepared following general procedure **GP5** using dimethyl 2-cinnamyl-2-(prop-2-yn-1-yl)malonate (**1a**, 28.6 mg, 0.100 mmol) and 5-methoxyindole (17.7 mg, 0.120 mmol, 1.2 equiv) as a nucleophile in 1,4-dioxane (1.0 mL, 0.1M), at 25 ºC for 6 h. The crude product was purified by FCC (silica, CyH:AcOEt 90:10 to 80:20). Compound **2c** was obtained as a white solid (36.7 mg, 0.837 mmol, 84% yield) with >99:1 *er*. The absolute configuration was assigned by comparison to **2v**.

M.p. 52 ºC.

^{1}H NMR (300 MHz, $CDCl_3$) δ 7.95 (brs, 1H), 7.34 – 7.28 (m, 2H), 7.26 – 7.07 (m, 5H), 6.95 (d, *J* = 2.4 Hz, 1H), 6.80 (dd, *J* = 8.8, 2.4 Hz, 1H), 4.80 (s, 1H), 4.18 – 4.07 (m, 2H), 3.79 (s, 3H), 3.72 (s, 3H), 3.64 (s, 3H), 3.50 (qd, *J* = 8.3, 1.8 Hz, 1H), 3.11 (dd, *J* = 15.9, 2.1 Hz, 1H), 2.89 (d, *J* = 15.9 Hz, 1H), 2.77 (ddd, *J* = 13.6, 7.9, 1.4 Hz, 1H), 1.98 (dd, *J* = 13.7, 8.5 Hz, 1H).

^{13}C NMR (75 MHz, $CDCl_3$) δ 172.4, 172.2, 154.0, 149.1, 144.4, 131.5, 128.6, 128.2, 127.8, 126.2, 122.4, 118.5, 112.2, 111.8, 110.1, 101.6, 58.6, 56.0, 52.9, 52.8, 48.1, 46.9, 41.9, 39.9.

HRMS (ESI +) calculated for $[C_{26}H_{27}NNaO_5]^{+\cdot}$ $[M+Na]^{+\cdot}$ 456.1781 m/z; found 456.1783 m/z**.**

SFC (IB-N (100 × 3 mm, 3 μm), 80:30 CO_2:MeOH, 1.2 mL/min, 35 ºC, BPR 150 bar, 210 nm): en2 (major, 100%) min 1.25.

$[\alpha]^{24.6}_{D}$ +26.7 (c 1.13, $CHCl_3$).

Dimethyl (*R*)-3-((*R*)-(1-Methyl-1H-indol-3-yl)(phenyl)methyl)-4-methylenecyclopentane-1,1-dicarboxylate (2d)

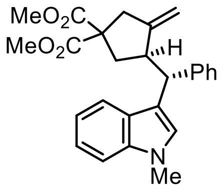

Prepared following general procedure **GP5** using dimethyl 2-cinnamyl-2-(prop-2-yn-1-yl)malonate (**1a**, 28.6 mg, 0.100 mmol) and *N*-methylindole (15.7 mg, 0.120 mmol, 1.2 equiv) as a nucleophile in 1,4-dioxane (1.0 mL, 0.1M), at 25 ºC for 6 h. The crude product was purified by FCC (silica, CyH:AcOEt 90:10 to 80:20). Compound **2d** was obtained as a white solid (41.1 mg, 0.984 mmol, 98% yield) with 99:1 *er*.

Spectroscopic data matched those reported in the literature.[11] The absolute configuration was assigned by comparison to **2v**.

^{1}H NMR (300 MHz, $CDCl_3$) δ 7.60 – 7.53 (m, 1H), 7.38 – 7.29 (m, 2H), 7.25 – 7.03 (m, 5H), 7.03 – 6.97 (m, 1H), 4.79 (s, 1H), 4.19 – 4.07 (m, 2H), 3.76 (s, 3H), 3.73 (s, 3H), 3.65 (s, 3H), 3.60 – 3.47 (m, 1H), 3.14 (dq, *J* = 15.9, 2.4 Hz, 1H), 2.94 – 2.83 (m, 1H), 2.77 (ddd, *J* = 13.6, 7.9, 1.4 Hz, 1H), 1.97 (dd, *J* = 13.7, 8.5 Hz, 1H).

^{13}C NMR (75 MHz, $CDCl_3$) δ 172.4, 172.2, 149.1, 144.7, 137.0, 128.6, 128.2, 127.7, 126.3, 126.1, 121.7, 119.5, 119.0, 117.3, 110.1, 109.2, 58.5, 52.9, 52.8, 48.2, 46.9, 41.9, 40.1, 32.9.

SFC (OD (100 × 3 mm, 3 μm), 80:20 CO_2:MeOH, 1.2 mL/min, 35 ºC, BPR 150 bar, 210 nm): en1 (major, 99%) 1.12 min, en2 (minor, 1%) 1.23 min.

$[\alpha]^{24.6}_D$ –7.25 (c 1.49, $CHCl_3$).

Dimethyl (*R*)-3-((*R*)-(2-Methyl-1H-indol-3-yl)(phenyl)methyl)-4-methylenecyclopentane-1,1-dicarboxylate (2e)

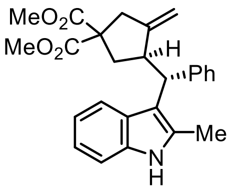

Prepared following general procedure **GP5** using dimethyl 2-cinnamyl-2-(prop-2-yn-1-yl)malonate (**1a**, 28.6 mg, 0.100 mmol) and 2-methylindole (15.7 mg, 0.120 mmol, 1.2 equiv) as a nucleophile in 1,4-dioxane (1.0 mL, 0.1M), at 25 ºC for 6 h. The crude product was purified by FCC (silica, CyH:AcOEt 90:10 to 80:20). Compound **2e** was obtained as a white solid (35.5 mg, 0.85 mmol, 85% yield) with >99:1 *er*. The absolute configuration was assigned by comparison to **2v**.

M.p. 61–66 ºC.

^{1}H NMR (300 MHz, $CDCl_3$) δ 7.79 – 7.68 (m, 2H), 7.41 (d, *J* = 7.3 Hz, 2H), 7.25 – 7.17 (m, 3H), 7.14 – 7.03 (m, 3H), 4.89 – 4.78 (m, 1H), 4.31 – 4.23 (m, 1H), 4.04 (s, 1H), 3.99 – 3.82 (m, 1H), 3.72 (s, 3H), 3.62 (s, 3H), 3.20 – 3.08 (m, 1H), 3.05 – 2.93 (m, 1H), 2.57 – 2.46 (m, 1H), 2.41 (s, 3H), 1.84 (dd, *J* = 13.5, 9.2 Hz, 1H).

^{13}C NMR (75 MHz, $CDCl_3$) δ 172.5, 172.4, 150.2, 144.8, 135.4, 131.3, 128.3, 128.2, 127.8, 125.9, 120.9, 119.6, 119.4, 114.5, 110.4, 109.6, 58.0, 52.8, 52.8, 47.9, 45.1, 41.8, 39.9, 12.7.

HRMS (ESI +) calculated for $[C_{26}H_{27}NNaO_4]^{+\cdot}$ $[M+Na]^{+\cdot}$ 440.1830 m/z; found 440.1830 m/z.

SFC (IB-N (100 × 3 mm, 3 μm), 80:20 CO_2:*i*-PrOH, 1.2 mL/min, 35 ºC, BPR 150 bar, 210 nm): en2 (major, 100%) 3.33 min.

$[\alpha]^{24.6}_D$ –9.49 (c 1.08, $CHCl_3$).

Dimethyl (*R*)-3-Methylene-4-((*R*)-phenyl(2,4,6-trimethoxyphenyl)methyl)cyclopentane-1,1-dicarboxylate (2f)

Prepared following general procedure **GP5**, using dimethyl 2-cinnamyl-2-(prop-2-yn-1-yl)malonate (**1a**, 28.6 mg, 0.100 mmol) and 1,3,5-trimethoxybenzene (33.6 mg, 0.200 mmol, 2.0 equiv) as a

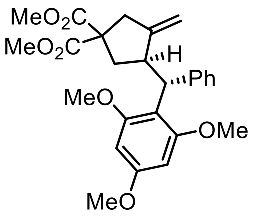

nucleophile in toluene (1.0 mL, 0.1M), at −10 ºC for 6 h. The crude product was purified by FCC (silica, CyH:AcOEt 90:10 to 80:20). Compound **2f** was obtained as a white solid (39.4 mg, 0.870 mmol, 87% yield) with 98:2 *er*.
Spectroscopic data matched those reported in the literature.[12] The absolute configuration was assigned by comparison to **2v**.

M.p. 74 ºC.

^{1}H NMR (500 MHz, $CDCl_3$) δ 7.48 – 7.44 (m, 2H), 7.19 (t, *J* = 7.7 Hz, 2H), 7.09 (t, *J* = 7.3 Hz, 1zH), 6.08 (s, 1H), 4.73 (q, *J* = 2.2 Hz, 1H), 4.50 (d, *J* = 11.0 Hz, 1H), 4.26 (qd, *J* = 2.2, 0.8 Hz, 1H), 3.99 (tdt, *J* = 11.9, 7.5, 2.4 Hz, 1H), 3.81 (s, 6H), 3.76 (s, 3H), 3.74 (s, 3H), 3.66 (s, 3H), 3.09 (dq, *J* = 16.5, 2.2 Hz, 1H), 3.01 (dq, *J* = 16.6, 1.9 Hz, 1H), 2.37 (ddd, *J* = 12.9, 7.5, 1.6 Hz, 1H).

^{13}C NMR (126 MHz, $CDCl_3$) δ 172.6, 172.5, 159.6, 151.5, 144.7, 128.8, 127.8, 125.6, 113.7, 108.2, 91.1, 57.8, 55.7, 55.3, 52.8, 52.7, 44.3, 43.1, 41.8, 40.1.

SFC (IG-3 (150 × 4.6 mm, 3 μm), 93:7 CO_2:*i*-PrOH, 2.0 mL/min, 35 ºC, BPR 140 bar, 230 nm): en1 (minor, 2%) 4.33 min, en2 (major, 98%) 4.72 min.

$[\alpha]^{24.6}_D$ +80.9 (c 1.79, $CHCl_3$).

Dimethyl (*R*)-3-Methylene-4-((*R*)-phenyl(2,4-dimethoxyphenyl)methyl)cyclopentane-1,1-dicarboxylate (2g)

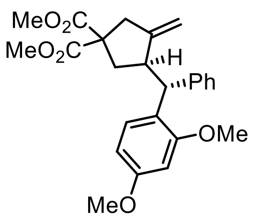

Prepared following general procedure **GP5**, using dimethyl 2-cinnamyl-2-(prop-2-yn-1-yl)malonate (**1a**, 28.6 mg, 0.100 mmol) and 1,3-dimethoxybenzene (27.6 mg, 0.200 mmol, 2.0 equiv) as a nucleophile in toluene (1.0 mL, 0.1M), at −10 ºC for 6 h. The crude product was purified by FCC (silica, CyH:AcOEt 90:10 to 80:20). Compound **2g** was obtained as a white solid (33.4 mg, 0.790 mmol, 79% yield) with 99:1 *er*.
Spectroscopic data matched those reported in the literature.[12] The absolute configuration was assigned by comparison to **2v**.

^{1}H NMR (300 MHz, $CDCl_3$) δ 7.33 – 7.27 (m, 2H), 7.26 – 7.17 (m, 3H), 7.14 – 7.06 (m, 1H), 6.46 (dd, *J* = 8.4, 2.5 Hz, 1H), 6.38 (d, *J* = 2.5 Hz, 1H), 4.72 (s, 1H), 4.18 (d, *J* = 11.5 Hz, 1H), 4.06 – 4.01 (m, 1H), 3.76 (s, 6H), 3.73 (s, 3H), 3.67 (s, 3H), 3.61 – 3.46 (m, 1H), 3.12 (dq, *J* = 16.0, 2.3 Hz, 1H), 2.90 (dd, *J* = 16.0, 1.5 Hz, 1H), 2.55 (ddd, *J* = 13.4, 7.7, 1.5 Hz, 1H), 1.71 (dd, *J* = 13.4, 9.2 Hz, 1H).

^{13}C NMR (75 MHz, $CDCl_3$) δ 172.5, 172.2, 159.2, 158.0, 149.5, 144.5, 128.7, 128.1, 126.0, 125.1, 109.7, 104.5, 98.8, 58.2, 55.5, 55.4, 52.9, 52.8, 48.4, 45.3, 41.9, 40.1.

SFC (IB-N (100 × 3 mm, 3 μm), 90:10 CO_2:MeOH, 1.2 mL/min, 35 ºC, BPR 150 bar, 210 nm): en1 (minor, 1%) 1.20 min, en2 (major, 99%) 1.67 min.

$[\alpha]^{24.6}_D$ +20.03 (c 1.46, $CHCl_3$).

Dimethyl (*R*)-3-((*R*)-(4-(Dimethylamino)phenyl)(phenyl)methyl)-4-methylenecyclopentane-1,1-dicarboxylate (2h)

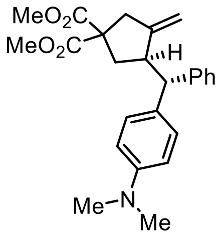

Prepared following general procedure **GP5**, using dimethyl 2-cinnamyl-2-(prop-2-yn-1-yl)malonate (**1a**, 28.6 mg, 0.100 mmol) and *N,N*-dimethtlaniline (13.9 µL, 0.110 mmol, 1.1 equiv) as a nucleophile in toluene (1.0 mL, 0.1M), at 25 ºC for 24 h. The crude product was purified by FCC (silica, CyH:AcOEt 90:10). Compound **2h** was obtained as a white solid (39.7 mg, 0.977 mmol, 98% yield) with >99:1 *er*. The absolute configuration was assigned by comparison to **2v**.

M.p. 104–107 ºC.

^{1}H NMR (300 MHz, $CDCl_3$) δ 7.32 – 7.27 (m, 2H), 7.26 – 7.19 (m, 2H), 7.19 – 7.08 (m, 3H), 6.71 – 6.62 (m, 2H), 4.78 – 4.73 (m, 1H), 4.17 – 4.11 (m, 1H), 3.76 – 3.70 (m, 4H), 3.68 (s, 3H), 3.58 – 3.44 (m, 1H), 3.14 – 3.04 (m, 1H), 2.96 – 2.84 (m, 7H), 2.55 (ddd, *J* = 13.4, 7.6, 1.3 Hz, 1H), 1.80 (dd, *J* = 13.5, 9.1 Hz, 1H).

^{13}C NMR (126 MHz, $CDCl_3$) δ 172.4, 172.3, 149.5, 149.2, 145.1, 132.1, 128.9, 128.4, 128.2, 126.1, 112.9, 109.7, 58.2, 56.0, 52.9, 52.8, 46.1, 41.9, 40.8, 40.0.

HRMS (ESI +) calculated for $[C_{25}H_{30}NO_4]^{+\cdot}$ $[M+H]^{+\cdot}$ 408.2169 m/z; found 408.2176 m/z.

SFC (IB-N (100 × 3 mm, 3 µm), 80:20 CO_2:MeOH, 1.2 mL/min, 35 ºC, BPR 150 bar, 210 nm): en1 (minor, 0.5%) 1.11 min, en2 (major, 99.5%) 1.35 min.

$[\alpha]^{24.6}_D$ +17.1 (c 0.56, $CHCl_3$).

Dimethyl (*S*)-3-Methylene-4-((*R*)-phenyl(phenylamino)methyl)cyclopentane-1,1-dicarboxylate (2i)

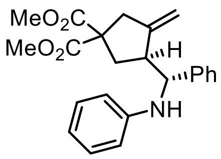

Prepared following general procedure **GP5**, using dimethyl 2-cinnamyl-2-(prop-2-yn-1-yl)malonate (**1a**, 28.6 mg, 0.100 mmol) and aniline (10.0 µL, 0.110 mmol, 1.1 equiv) as a nucleophile in toluene (1.0 mL, 0.1M), at 25 ºC for 4 h. The crude product was purified by FCC (silica, CyH:AcOEt 90:10). Compound **2i** was obtained as a white solid (32.4 mg, 0.854 mmol, 85% yield) with 99:1 *er*.

Spectroscopic data matched those reported in the literature.[13] The absolute configuration was assigned by comparison to **2v**.

^{1}H NMR (300 MHz, $CDCl_3$) δ 7.41 – 7.28 (m, 4H), 7.26 – 7.14 (m, 1H), 7.12 – 7.02 (m, 2H), 6.69 – 6.58 (m, 1H), 6.50 (dd, *J* = 8.6, 1.0 Hz, 2H), 5.06 (q, *J* = 2.0 Hz, 1H), 4.76 (q, *J* = 2.1 Hz, 1H), 4.54 (d, *J* = 5.0 Hz, 1H), 4.24 (brs, 1H), 3.75 (s, 3H), 3.69 (s, 3H), 3.18 – 2.87 (m, 3H), 2.39 – 2.15 (m, 2H).

^{13}C NMR (75 MHz, $CDCl_3$) δ 172.6, 172.0, 147.9, 147.3, 142.4, 129.1, 128.7, 127.2, 126.7, 117.64, 113.9, 109.2, 59.2, 57.8, 53.1, 52.9, 49.45 42.2, 35.0.

SFC (IG (100 × 3 mm, 3 µm), 80:20 CO_2:EtOH, 1.2 mL/min, 35 ºC, BPR 150 bar, 210 nm): en1 (major, 100%) 0.75 min.

[α]$^{24.6}_{D}$ +39.6 (c 1.27, $CHCl_3$).

Dimethyl (*S*)-3-((*R*)-((2-Bromophenyl)amino)(phenyl)methyl)-4-methylenecyclopentane-1,1-dicarboxylate (2j)

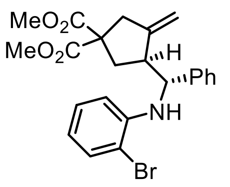

Prepared following general procedure **GP5**, using dimethyl 2-cinnamyl-2-(prop-2-yn-1-yl)malonate (**1a**, 28.6 mg, 0.100 mmol) and 2-bromoaniline (18.9 mg, 0.110 mmol, 1.1 equiv) as a nucleophile in toluene (1.0 mL, 0.1M), at 25 ºC for 3 h. The crude product was purified by FCC (silica, CyH:AcOEt 90:10). Compound **2i** was obtained as a white solid (42.5 mg, 0.927 mmol, 93% yield) with >99:1 *er*.

Spectroscopic data matched those reported in the literature.[13] The absolute configuration was assigned by comparison to **2v**.

^{1}H NMR (500 MHz, $CDCl_3$) δ 7.40 (dd, *J* = 7.9, 1.5 Hz, 1H), 7.35 – 7.29 (m, 4H), 7.24 (m, 1H), 6.97 (ddd, *J* = 8.5, 7.4, 1.5 Hz, 1H), 6.51 (td, *J* = 7.6, 1.5 Hz, 1H), 6.34 (dd, *J* = 8.2, 1.5 Hz, 1H), 5.07 (d, *J* = 2.3 Hz, 1H), 4.79 (d, *J* = 4.8 Hz, 1H), 4.70 (d, *J* = 2.2 Hz, 1H), 4.51 (t, *J* = 5.3 Hz, 1H), 3.72 (s, 3H), 3.69 (s, 3H), 3.16 (td, *J* = 8.8, 4.7 Hz, 1H), 3.01 (d, *J* = 2.1 Hz, 2H), 2.42 (dd, *J* = 13.7, 8.2 Hz, 1H), 2.27 (dd, *J* = 13.7, 9.9 Hz, 1H).

^{13}C NMR (126 MHz, $CDCl_3$) δ 171.4, 171.9, 147.1, 144.4, 141.4, 132.3, 128.7, 128.4, 127.5, 127.0, 118.3, 113.1, 110.5, 110.1, 60.4, 58.3, 53.0, 53.0, 49.5, 42.2, 35.6.

HRMS (ESI +) calculated for $[C_{23}H_{24}BrNNaO_4]^{+\cdot}$ $[M+Na]^{+\cdot}$ 480.0785 m/z; found 480.0781 m/z.

SFC (IG (100 × 3 mm, 3 μm), 95:5 CO_2:*i*-PrOH, 1.5 mL/min, 35 ºC, BPR 150 bar, 210 nm): en1 (major, 100%) 2.08 min.

[α]$^{24.6}_{D}$ –11.2 (c 1.27, $CHCl_3$).

Dimethyl (*S*)-3-((*R*)-((2-Iodophenyl)amino)(phenyl)methyl)-4-methylenecyclopentane-1,1-dicarboxylate (2k)

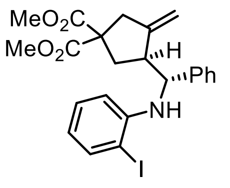

Prepared following general procedure **GP5**, using dimethyl 2-cinnamyl-2-(prop-2-yn-1-yl)malonate (**1a**, 28.6 mg, 0.100 mmol) and 2-iodoaniline (24.1 mg, 0.110 mmol, 1.1 equiv) as a nucleophile in toluene (1.0 mL, 0.1M), at 25 ºC for 4 h. The crude product was purified by FCC (silica, CyH:AcOEt 90:10). Compound **2k** was obtained as a white sticky solid (44.7 mg, 0.946 mmol, 95% yield) with 98.5:1.5 *er*.

Spectroscopic data matched those reported in the literature.[13] The absolute configuration was assigned by comparison to **2v**.

^{1}H NMR (300 MHz, $CDCl_3$) δ 7.64 (dd, *J* = 7.8, 1.5 Hz, 1H), 7.38 – 7.27 (m, 4H), 7.25 – 7.19 (m, 1H), 6.99 (ddd, *J* = 8.7, 7.4, 1.5 Hz, 1H), 6.39 (td, *J* = 7.7, 1.5 Hz, 1H), 6.27 (dd, *J* = 8.2, 1.3 Hz, 1H), 5.13 – 5.05 (m, 1H), 4.79 – 4.70 (m, 1H), 4.61 (brs, 1H), 4.58 – 4.46 (m, 1H), 3.72 (s, 3H), 3.70 (s, 3H), 3.24 – 3.10 (m, 1H), 3.05 (d, *J* = 2.1 Hz, 2H), 2.42 (dd, *J* = 13.7, 8.0 Hz, 1H), 2.28 (dd, *J* = 13.7, 10.2 Hz, 1H).

^{13}C NMR (75 MHz, $CDCl_3$) δ 171.9, 171.8, 147.1, 146.6, 141.2, 138.9, 129.3, 128.7, 127.4, 126.9, 119.1, 112.4, 110.2, 86.3, 60.8, 58.3, 53.00, 52.96, 49.6, 42.3, 35.6.

SFC (OD (100 × 3 mm, 3 μm), 95:5 CO_2:*i*-PrOH, 1.2 mL/min, 35 ºC, BPR 150 bar, 210 nm): en1 (minor, 1.5%) 2.87 min, en2 (major, 98.5%) 3.53 min.

$[\alpha]^{24.6}_D$ –29.4 (c .90, $CHCl_3$).

Dimethyl (*S*)-3-((*R*)-(((Benzyloxy)carbonyl)amino)(phenyl)methyl)-4-methylenecyclopentane-1,1-dicarboxylate (2l)

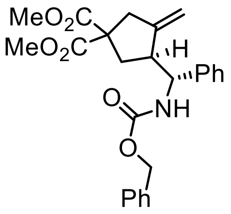

Prepared following general procedure **GP5**, using dimethyl 2-cinnamyl-2-(prop-2-yn-1-yl)malonate (**1a**, 28.6 mg, 0.100 mmol) and benzyl carbamate (30.3 mg, 0.200 mmol, 2.0 equiv) as a nucleophile in toluene (1.0 mL, 0.1M), at −10 ºC for 6 h. The crude product was purified by FCC (silica, CH_2Cl_2:AcOEt 99:1). Compound **2l** was obtained as a waxy white solid (41.7 mg, 0.953 mmol, 95% yield) with >99:1 *er*.

The absolute configuration was assigned by comparison to **2v**.

^{1}H NMR (500 MHz, $CDCl_3$) δ 7.39 – 7.30 (m, 7H), 7.28 (m, 3H), 5.51 (s, 1H), 5.08 (q, *J* = 12.4 Hz, 2H), 4.99 (q, *J* = 2.2 Hz, 2H), 4.69 (s, 1H), 3.72 (s, 3H), 3.69 (s, 3H), 3.14 (s, 1H), 2.99 (dd, *J* = 16.2, 1.7 Hz, 1H), 2.87 (dq, *J* = 16.2, 2.2 Hz, 1H), 2.40 – 2.31 (m, 1H), 2.09 (dd, *J* = 13.8, 9.0 Hz, 1H).

^{13}C NMR (101 MHz, $CDCl_3$) δ 172.2, 171.8, 156.1, 146.8, 128.6, 128.5, 128.0, 127.4, 126.6, 109.7, 66.8, 57.9, 53.0, 52.9, 47.2, 42.1, 35.5.

HRMS (ESI +) calculated for $[C_{25}H_{27}NNaO_6]^{+\cdot}$ $[M+Na]^{+\cdot}$ 460.1731 m/z; found 460.1733 m/z.

SFC (IG (100 × 3 mm, 3 μm), 75:25 CO_2:EtOH, 1.2 mL/min, 35 ºC, BPR 150 bar, 210 nm): en1 (major, 100%) 1.69 min.

$[\alpha]^{24.6}_D$ +47.62 (c 0.61, $CHCl_3$).

Dimethyl (*S*)-3-((*R*)-Hydroxy(phenyl)methyl)-4-methylenecyclopentane-1,1-dicarboxylate (2m)

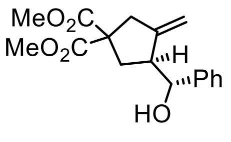

Prepared following general procedure **GP5**, using dimethyl 2-cinnamyl-2-(prop-2-yn-1-yl)malonate (**1a**, 28.6 mg, 0.100 mmol) and water (9.0 μL, 0.500 mmol, 5.0 equiv) as a nucleophile in HPLC-grade toluene (1.0 mL, 0.1M), at 25 ºC for 6 h. The crude product was purified by FCC (silica, CyH:AcOEt 90:10 to 80:20). Compound **2m** was obtained as a white solid (27.9 mg, 0.917 mmol, 92% yield) with 98:2 *er*.

Spectroscopic data matched those reported in the literature.[14] The absolute configuration was assigned by comparison to **2v**.

^{1}H NMR (300 MHz, $CDCl_3$) δ 7.39 – 7.30 (m, 4H), 7.29 – 7.22 (m, 1H), 5.14 (q, *J* = 2.1 Hz, 1H), 4.98 (d, *J* = 3.9 Hz, 1H), 4.91 (q, *J* = 2.1 Hz, 1H), 3.72 (s, 3H), 3.68 (s, 3H), 3.08 – 2.87 (m, 3H), 2.26 (qd, *J* = 13.6, 9.0 Hz, 3H).

^{13}**C NMR** (75 MHz, $CDCl_3$) δ 172.21, 172.16, 149.1, 142.5, 128.4, 127.4, 126.0, 108.5, 74.2, 58.4, 52.92, 52.88, 49.9, 42.2, 33.8.

SFC (IG (100 × 3 mm, 3 µm), 85:15 CO_2:MeOH, 1.2 mL/min, 35 ºC, BPR 150 bar, 210 nm): en1 (major, 98%) 1.31 min, en2 (minor, 2%) 1.49 min.

[α]$^{24.6}$$_D$ +70.8 (c 0.70, $CHCl_3$).

Dimethyl (*S*)-3-((*R*)-Methoxy(phenyl)methyl)-4-methylenecyclopentane-1,1-dicarboxylate (2n)

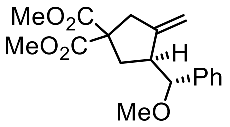

Prepared following general procedure **GP5**, using dimethyl 2-cinnamyl-2-(prop-2-yn-1-yl)malonate (**1a**, 28.6 mg, 0.100 mmol) and anhydrous MeOH (20.2 µL, 0.500 mmol, 5.0 equiv) as a nucleophile in 1,4-dioxane (1.0 mL, 0.1M), at 25 ºC for 2 h. The crude product was purified by FCC (silica, CyH:AcOEt 100:0 to 90:10). Compound **2n** was obtained as a white solid (28.3 mg, 0.889 mmol, 89% yield) with >99:1 *er*.

Spectroscopic data matched those reported in the literature.[15] The absolute configuration was assigned by comparison to **2v**.

^{1}**H NMR** (300 MHz, $CDCl_3$) δ 7.39 – 7.26 (m, 5H), 4.97 – 4.89 (m, 1H), 4.56 – 4.47 (m, 1H), 4.17 (d, *J* = 5.9 Hz, 1H), 3.74 (s, 3H), 3.67 (s, 3H), 3.20 (s, 3H), 3.04 – 2.82 (m, 3H), 2.51 – 2.25 (m, 2H).

^{13}**C NMR** (75 MHz, $CDCl_3$) δ 172.3, 172.2, 148.6, 140.7, 128.4, 127.7, 127.4, 108.6, 85.8, 58.7, 57.3, 52.8, 49.5, 42.2, 35.5.

HPLC (OJ-H (250 × 4.6 mm, 5 µm), 99.5:0.5 hexane:*i*-PrOH, 1.0 mL/min, 25 °C, 210 nm): en1 (major, 99.5%) 17.99 min, (minor, 0.5%) 20.25 min.

[α]$^{24.6}$$_D$ +72.5 (c 1.02, $CHCl_3$).

Dimethyl (*S*)-3-((*R*)-Ethoxy(phenyl)methyl)-4-methylenecyclopentane-1,1-dicarboxylate (2o)

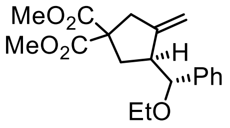

Prepared following general procedure **GP5**, using dimethyl 2-cinnamyl-2-(prop-2-yn-1-yl)malonate (**1a**, 28.6 mg, 0.100 mmol) and anhydrous EtOH (29.2 µL, 0.500 mmol, 5.0 equiv) as a nucleophile in 1,4-dioxane (1.0 mL, 0.1M), at 25 ºC for 5 h. The crude product was purified by FCC (silica, CyH:AcOEt 100:0 to 90:10). Compound **2o** was obtained as a white solid (32.1 mg, 0.966 mmol, 97% yield) with 98.5:1.5 *er*.

Spectroscopic data matched those reported in the literature.[16] The absolute configuration was assigned by comparison to **2v**.

^{1}**H NMR** (300 MHz, $CDCl_3$) δ 7.39 – 7.26 (m, 5H), 7.26 – 7.19 (m, 1H), 4.93 – 4.87 (m, 1H), 4.47 (s, 1H), 4.25 (d, *J* = 6.1 Hz, 1H), 3.74 (s, 3H), 3.67 (s, 3H), 3.46 – 3.22 (m, 2H), 3.07 – 2.79 (m, 3H), 2.45 (ddd, *J* = 13.5, 8.1, 1.4 Hz, 1H), 2.35 (dd, *J* = 13.5, 8.7 Hz, 1H), 1.13 (t, *J* = 7.0 Hz, 3H).

^{13}**C NMR** (75 MHz, $CDCl_3$) δ 172.3, 172.3, 148.7, 141.5, 128.3, 127.6, 127.3, 108.6, 83.9, 64.8, 58.8, 52.8, 49.5, 42.3, 35.5, 15.3.

HPLC (OJ-H (250 × 4.6 mm, 5 µm), 99:1 hexane:*i*-PrOH, 1.0 mL/min, 25 °C, 210 nm): en1 (major, 98.5%) 20.48 min, en2 (minor, 1.5%) 25.91 min.

[α]$^{24.6}$$_{D}$ +74.6 (c 1.12, $CHCl_3$).

Dimethyl (*S*)-3-((*R*)-Isopropoxy(phenyl)methyl)-4-methylenecyclopentane-1,1-dicarboxylate (2p)

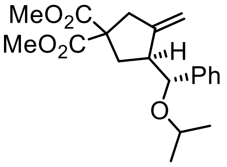

Prepared following general procedure **GP5**, using dimethyl 2-cinnamyl-2-(prop-2-yn-1-yl)malonate (**1a**, 28.6 mg, 0.100 mmol) and anhydrous 2-propanol (38.2 µL, 0.500 mmol, 5.0 equiv) as a nucleophile in 1,4-dioxane (1.0 mL, 0.1M), at 25 ºC for 5 h. The crude product was purified by FCC (silica, CyH:AcOEt 100:0 to 90:10). Compound **2p** was obtained as a waxy solid (26.0 mg, 0.750 mmol, 75% yield) with >99:1 *er*. The absolute configuration was assigned by comparison to **2v**.

^{1}H NMR (300 MHz, $CDCl_3$) δ 7.37 – 7.24 (m, 7H), 4.91 (s, 1H), 4.49 (s, 1H), 4.38 (d, *J* = 5.8 Hz, 1H), 3.74 (s, 3H), 3.67 (s, 3H), 3.45 (hept, *J* = 6.1 Hz, 1H), 2.99 (dq, *J* = 15.8, 2.7 Hz, 1H), 2.91 – 2.78 (m, 2H), 2.48 – 2.27 (m, 2H), 2.17 (s, 2H), 1.09 (d, *J* = 6.0 Hz, 3H), 1.03 (d, *J* = 6.2 Hz, 3H).

^{13}C NMR (75 MHz, $CDCl_3$) δ 172.5, 172.30 157.4, 149.4, 136.1, 133.0, 128.8, 127.8, 127.0, 122.0, 121.8, 120.6, 119.6, 119.4, 118.7, 111.0, 110.8, 108.9, 58.8, 55.6, 52.9, 52.8, 46.8, 41.8, 40.2, 39.4.

HPLC (OJ-H (250 × 4.6 mm, 5 µm), 99:1 hexane:*i*-PrOH, 1.0 mL/min, 25 °C, 210 nm): en1 (major, 100%) 18.83 min.

[α]$^{24.6}$$_{D}$ +83.6 (c 1.09, $CHCl_3$).

Dimethyl (*S*)-3-((*R*)-(Benzyloxy)(phenyl)methyl)-4-methylenecyclopentane-1,1-dicarboxylate (2q)

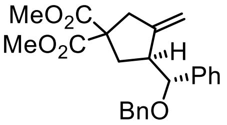

Prepared following general procedure **GP5**, using dimethyl 2-cinnamyl-2-(prop-2-yn-1-yl)malonate (**1a**, 28.6 mg, 0.100 mmol) and benzyl alcohol (41.6 µL, 0.400 mmol, 4.0 equiv) as a nucleophile in 1,4-dioxane (1.0 mL, 0.1M), at 25 ºC for 6 h. The crude product was purified by FCC (silica, CyH:AcOEt 90:10 to 80:20). Compound **2q** was obtained as a colorless oil (36.0 mg, 0.910 mmol, 91% yield) with 98.5:1.5 *er*.

^{1}H NMR (300 MHz, $CDCl_3$) δ 7.42 – 7.26 (m, 10H), 4.95 – 4.87 (m, 1H), 4.53 – 4.36 (m, 3H), 4.25 (d, *J* = 11.8 Hz, 1H), 3.71 (s, 3H), 3.68 (s, 3H), 3.04 – 2.81 (m, 3H), 2.59 – 2.32 (m, 2H).

^{13}C NMR (75 MHz, $CDCl_3$) δ 172.3, 172.2, 148.3, 140.8, 138.6, 128.4, 128.3, 127.8, 127.5, 108.9, 83.5, 70.9, 58.8, 52.8, 49.5, 42.3, 35.6.

HRMS (ESI +) calculated for $[C_{24}H_{26}NaO_5]^{+\cdot}$ $[M+Na]^{+\cdot}$ 4171672 m/z; found 4171667m/z.

SFC (OJ (100 × 3 mm, 3 µm), 99:1 CO_2:MeOH, 1.2 mL/min, 35 ºC, BPR 150 bar, 210 nm): en1 (major, 98.5%) 1.76 min, en2 (minor, 1.5%) 2.45 min.

[α]$^{24.6}$$_{D}$ +78.2 (c 1.43, $CHCl_3$).

Dimethyl (*S*)-3-((*R*)-Allyloxy(phenyl)methyl)-4-methylenecyclopentane-1,1-dicarboxylate (2r)

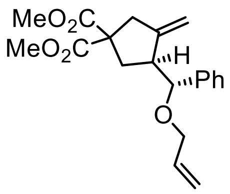

Prepared following general procedure **GP5**, using dimethyl 2-cinnamyl-2-(prop-2-yn-1-yl)malonate (**1a**, 28.6 mg, 0.100 mmol) and allyl alcohol (27.1 µL, 0.400 mmol, 4.0 equiv) as a nucleophile in 1,4-dioxane (1.0 mL, 0.1M), at 25 ºC for 6 h. The crude product was purified by FCC (silica, CyH:AcOEt 90:10 to 80:20). Compound **2r** was obtained as a colorless oil (34.0 mg, 0.987 mmol, 99% yield) with 98.5:1.5 *er*.

Spectroscopic data matched those reported in the literature.[16] The absolute configuration was assigned by comparison to **2v**.

^{1}H NMR (500 MHz, $CDCl_3$) δ 7.35 – 7.26 (m, 5H), 5.91 – 5.78 (m, 1H), 5.21 (dq, *J* = 17.2, 1.7 Hz, 1H), 5.12 (dd, *J* = 10.4, 1.6 Hz, 1H), 4.92 (s, 1H), 4.50 (s, 1H), 4.34 (d, *J* = 5.9 Hz, 1H), 3.92 (ddt, *J* = 12.9, 5.0, 1.5 Hz, 1H), 3.77 – 3.70 (m, 4H), 3.68 (s, 3H), 3.01 – 2.85 (m, 3H), 2.46 (ddd, *J* = 13.4, 8.1, 1.5 Hz, 1H), 2.37 (dd, *J* = 13.5, 8.9 Hz, 1H).

^{13}C NMR (126 MHz, $CDCl_3$) δ 172.3, 172.2, 148.6, 141.098, 135.1, 128.4, 127.7, 127.4, 116.6, 108.8, 83.2, 70.0, 58.8, 52.8, 49.5, 42.3, 35.5.

HPLC (OJ-H (250 × 4.6 mm, 5 µm), 99:1 hexane:*i*-PrOH, 1.0 mL/min, 25 °C, 210 nm): en1 (major, 98.5%) 8.59 min, en2 (minor, 1.5%).

$[\alpha]^{24.6}_{D}$ +77.6 (c 1.18, $CHCl_3$).

Dimethyl (*S*)-3-Methylene-4-((*R*)-((3-oxocyclohex-1-en-1-yl)oxy)(phenyl)methyl)cyclopentane-1,1-dicarboxylate (2s)

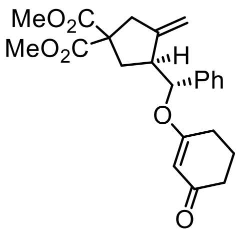

Prepared following general procedure **GP5**, using dimethyl 2-cinnamyl-2-(prop-2-yn-1-yl)malonate (**1a**, 28.6 mg, 0.100 mmol) and cyclohexan-1,3-dione (22.4 mg, 0.200 mmol, 2.0 equiv) as a nucleophile in 1,4-dioxane (1.0 mL, 0.1M), at 25 ºC for 6 h. The crude product was purified by FCC (silica, CyH:AcOEt 90:10 to 80:20). Compound **2s** was obtained as a colorless oil (35.3 mg, 0.886 mmol, 89% yield) with 97.5:2.5 *er*.

Spectroscopic data matched those reported in the literature.[17] The absolute configuration was assigned by comparison to **2v**.

^{1}H NMR (300 MHz, $CDCl_3$) δ 7.38 – 7.26 (m, 3H), 7.20 (dd, *J* = 7.9, 1.5 Hz, 2H), 5.17 (s, 1H), 5.12 (d, *J* = 5.2 Hz, 1H), 5.04 – 4.97 (m, 1H), 4.66 – 4.57 (m, 1H), 3.73 (s, 3H), 3.68 (s, 3H), 3.07 (d, *J* = 5.1 Hz, 1H), 2.94 (s, 2H), 2.42 (dt, *J* = 14.3, 7.1 Hz, 3H), 2.35 – 2.17 (m, 3H), 1.95 (q, *J* = 6.6 Hz, 2H).

^{13}C NMR (75 MHz, $CDCl_3$) δ 199.7, 176.7, 171.9, 171.82 147.2, 138.1, 128.8, 128.3, 126.3, 109.4, 105.2, 81.8, 58.6, 53.0, 49.1, 42.1, 36.7, 34.9, 29.1, 21.2.

SFC (IC (100 × 3 mm, 3 µm), 80:20 CO_2:EtOH, 1.2 mL/min, 35 ºC, BPR 150 bar, 210 nm): en1 (major, 97.5%) 1.55 min, en2 (minor, 2.5%) 1.91 min.

$[\alpha]^{24.6}_{D}$ +59.2 (c 1.4, $CHCl_3$).

Dimethyl (*R*)-3-((*R*)-(1H-indol-3-yl)(4-(trifluoromethyl)phenyl)methyl)-4-methylenecyclopentane-1,1-dicarboxylate (2t)

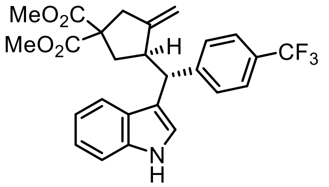

Prepared following general procedure **GP5** using dimethyl (*E*)-2-(prop-2-yn-1-yl)-2-(3-(4-(trifluoromethyl)phenyl)allyl)malonate (**1e**, 35.4 mg, 0.100 mmol) and indole (14.1 mg, 0.120 mmol, 1.2 equiv) as a nucleophile in 1,4-dioxane (1.0 mL, 0.1M), at 25 ºC for 6 h. The crude product was purified by FCC (silica, CyH:AcOEt 90:10 to 80:20). Compound **2t** was obtained as a white solid (47.0 mg, 0.997 mmol, >99% yield) with >99:1 *er*.

The absolute configuration was assigned by comparison to **2v**.

M.p. 61 ºC.

^{1}H NMR (300 MHz, $CDCl_3$) δ 8.11 (s, 1H), 7.55 – 7.40 (m, 5H), 7.41 – 7.32 (m, 1H), 7.25 – 7.14 (m, 2H), 7.12 – 7.02 (m, 1H), 4.82 (s, 1H), 4.24 (d, *J* = 10.4 Hz, 1H), 4.16 – 4.06 (m, 1H), 3.74 (d, *J* = 3.7 Hz, 3H), 3.66 (s, 3H), 3.59 – 3.46 (m, 1H), 3.15 (dq, *J* = 16.0, 2.5 Hz, 1H), 2.91 (dd, *J* = 16.0, 1.5 Hz, 1H), 2.78 (ddd, *J* = 13.7, 7.9, 1.6 Hz, 1H), 2.00 (dd, *J* = 13.7, 8.2 Hz, 1H).

^{13}C NMR (75 MHz, $CDCl_3$) δ 172.3, 172.2, 148.7, 148.5, 136.3, 128.9, 127.1, 125.2 (q, *J* = 3.8 Hz), 122.5, 121.6, 119.8, 119.3, 117.9, 111.3, 110.5, 58.5, 53.0, 52.9, 47.9, 46.9, 41.7, 39.9.

^{19}F NMR (282 MHz, $CDCl_3$) δ -62.3.

HRMS (ESI +) calculated for $[C_{26}H_{24}F_3NNaO_4]^{+\cdot}$ $[M+Na]^{+\cdot}$ 494.1550 m/z; found 494.1532 m/z.

SFC (OD (100 × 3 mm, 3 μm), 85:15 CO_2:*i*-PrOH, 1.2 mL/min, 35 ºC, BPR 150 bar, 210 nm): en2 (major, 100%) 1.74 min.

$[\alpha]^{24.6}_D$ –4.6 (c 0.31, $CHCl_3$).

Dimethyl (*R*)-3-((*R*)-(1H-Indol-3-yl)(4-nitrophenyl)methyl)-4-methylenecyclopentane-1,1-dicarboxylate (2u)

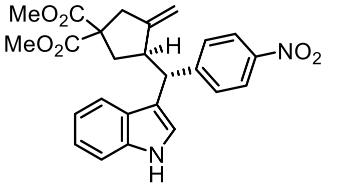

Prepared following general procedure **GP5** using dimethyl (*E*)-2-(prop-2-yn-1-yl)-2-(3-(4-(nitro)phenyl)allyl)malonate (**1f**, 33.1 mg, 0.100 mmol) and indole (14.1 mg, 0.120 mmol, 1.2 equiv) as a nucleophile in 1,4-dioxane (1.0 mL, 0.1M), at 25 ºC for 22 h. The crude product was purified by FCC (silica, CyH:AcOEt 90:10 to 80:20). Compound **2u** was obtained as a yellow solid (43.0 mg, 0.959 mmol, 96% yield) with >99:1 *er*.

The absolute configuration was assigned by comparison to **2v**.

M.p. 75–77 ºC.

^{1}H NMR (500 MHz, $CDCl_3$) δ 8.14 (s, 1H), 8.11 – 8.05 (m, 2H), 7.46 (d, *J* = 8.7 Hz, 2H), 7.43 (d, *J* = 8.3 Hz, 1H), 7.35 (d, *J* = 8.2 Hz, 1H), 7.20 (d, *J* = 2.5 Hz, 1H), 7.17 (ddd, *J* = 8.2, 7.0, 1.1 Hz, 1H), 7.05 (ddd, *J* = 8.0, 7.0, 1.0 Hz, 1H), 4.80 (d, *J* = 1.6 Hz, 1H), 4.26 (d, *J* = 10.6 Hz, 1H), 4.07 (dt, *J* = 2.8, 1.5

Hz, 1H), 3.72 (s, 3H), 3.64 (s, 3H), 3.51 (dtd, *J* = 9.9, 7.9, 1.8 Hz, 1H), 3.15 (dq, *J* = 15.9, 2.3 Hz, 1H), 2.94 – 2.86 (m, 1H), 2.79 (ddd, *J* = 13.8, 7.9, 1.5 Hz, 1H), 2.00 (dd, *J* = 13.8, 7.9 Hz, 1H).

^{13}C NMR (126 MHz, $CDCl_3$) δ 172.2, 172.1, 152.2, 148.5, 146.5, 136.4, 129.4, 126.9, 123.6, 122.7, 121.8, 120.0, 119.1, 117.1, 111.4, 110.7, 58.5, 53.0, 53.0, 48.0, 46.9, 41.6, 39.8, 31.1.

HRMS (ESI +) calculated for $[C_{25}H_{24}N_2NaO_6]^{+\cdot}$ $[M+Na]^{+\cdot}$ 471.1527 m/z; found 471.1527 m/z.

SFC (IA (100 × 3 mm, 3 μm), 80:20 CO_2:*i*-PrOH, 1.2 mL/min, 35 ºC, BPR 150 bar, 210 nm): en2 (major, 100%) 3.08 min.

$[\alpha]^{24.6}_D$ –5.45 (c 0.99, $CHCl_3$).

Dimethyl (*R*)-3-((*R*)-(4-Chlorophenyl)(1H-indol-3-yl)methyl)-4-methylenecyclopentane-1,1-dicarboxylate (2v)

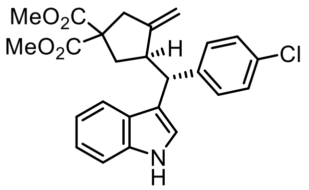

Prepared following general procedure **GP5** using dimethyl (*E*)-2-(prop-2-yn-1-yl)-2-(3-(4-chlorophenyl)allyl)malonate (**1g**, 32.1 mg, 0.100 mmol) and indole (14.1 mg, 0.120 mmol, 1.2 equiv) as a nucleophile in 1,4-dioxane (1.0 mL, 0.1M), at 25 ºC for 22 h. The crude product was purified by FCC (silica, CyH:AcOEt 90:10 to 80:20). Compound **2v** was obtained as a white solid (43.1 mg, 0.984 mmol, 98% yield) with 99:1 *er*.

The absolute configuration was assigned by X-Ray crystallography.

M.p. 129 ºC.

^{1}H NMR (300 MHz, , $CDCl_3$) δ 8.10 (s, 1H), 7.47 (d, *J* = 8.0 Hz, 1H), 7.32 (d, *J* = 8.1 Hz, 1H), 7.25 – 7.10 (m, 6H), 7.04 (td, *J* = 7.5, 7.1, 1.0 Hz, 1H), 4.81 (s, 1H), 4.28 – 4.01 (m, 2H), 3.72 (s, 3H), 3.63 (s, 3H), 3.53 – 3.36 (m, 1H), 3.12 (dq, *J* = 15.9, 2.4 Hz, 1H), 2.89 (dd, *J* = 15.9, 1.3 Hz, 1H), 2.75 (ddd, *J* = 13.6, 7.9, 1.4 Hz, 1H), 1.97 (dd, *J* = 13.7, 8.2 Hz, 1H).

^{13}C NMR (75 MHz, $CDCl_3$) δ 172.3, 172.2, 148.7, 143.0, 136.3, 131.8, 130.0, 128.3, 127.1, 122.3, 121.5, 119.6, 119.3, 118.2, 111.3, 110.5, 58.6, 53.0, 52.9, 47.5, 46.9, 41.8, 39.9.

$[\alpha]^{24.6}_D$ +2.41 (c 1.59, $CHCl_3$).

SFC (OJ (100 × 3 mm, 3 μm), 80:20 CO_2:EtOH, 1.2 mL/min, 35 ºC, BPR 150 bar, 210 nm): en1 (minor, 1%) 1.93 min, en2 (major, 99%) 2.43 min.

HRMS (ESI +) calculated for $[C_{25}H_{24}ClNNaO_4]^{+\cdot}$ $[M+Na]^{+\cdot}$ 460.1286 m/z; found 460.1296 m/z.

Dimethyl (*R*)-3-((*R*)-(1H-indol-3-yl)(4-methoxyphenyl)methyl)-4-methylenecyclopentane-1,1-dicarboxylate (2w)

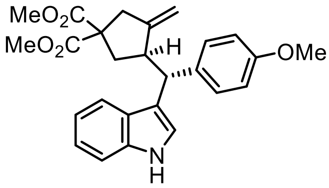

Prepared following general procedure **GP5** using dimethyl (*E*)-2-(prop-2-yn-1-yl)-2-(3-(4-methoxyphenyl)allyl)malonate (**1h**, 31.6 mg, 0.100 mmol) and indole (14.1 mg, 0.120 mmol, 1.2 equiv) as a nucleophile in 1,4-dioxane (1.0 mL, 0.1M), at 25 ºC for 6 h. The crude product was

purified by FCC (silica, pentane:AcOEt 90:10 to 80:20). Compound **2w** was obtained as a colorless oil (39.0 mg, 0.900 mmol, 90% yield) with 87:13 *er*.

The absolute configuration was assigned by comparison to **2v**.

M.p. 50–54 ºC.

^{1}H NMR (300 MHz, $CDCl_3$) δ 8.07 (brs, 1H), 7.55 (d, *J* = 7.9 Hz, 1H), 7.33 (d, *J* = 8.0 Hz, 1H), 7.27 – 7.20 (m, 2H), 7.19 – 7.10 (m, 2H), 7.10 – 7.03 (m, 1H), 6.84 – 6.72 (m, 2H), 4.83 (s, 1H), 4.19 (s, 1H), 4.14 (d, *J* = 10.3 Hz, 1H), 3.76 (s, 3H), 3.75 (s, 3H), 3.66 (s, 3H), 3.50 (q, *J* = 7.7 Hz, 1H), 3.13 (d, *J* = 15.9 Hz, 1H), 2.91 (d, *J* = 15.9 Hz, 1H), 2.76 (dd, *J* = 13.6, 8.0 Hz, 1H), 1.98 (dd, *J* = 13.6, 8.5 Hz, 1H).

^{13}C NMR (75 MHz, $CDCl_3$) δ 172.4, 172.2, 157.9, 149.0, 136.7, 136.3, 129.5, 127.3, 122.1, 121.4, 119.5, 119.5, 119.0, 113.6, 111.2, 110.2, 58.6, 55.3, 52.9, 52.8, 47.3, 47.0, 41.9, 40.0.

HRMS (ESI +) calculated for $[C_{26}H_{27}NNaO_5]^{+\cdot}$ $[M+Na]^{+\cdot}$ 456.1781 m/z; found 434.1794 m/z.

SFC (IG (100 × 3 mm, 3 μm), 80:20 CO_2:*i*-PrOH, 1.2 mL/min, 35 ºC, BPR 150 bar, 210 nm): en1 (minor, 13%) 1.58 min, en2 (major, 87%) 2.04 min.

$[\alpha]^{24.6}_D$ +3.85 (c 1.28, $CHCl_3$).

Dimethyl (*R*)-3-((*R*)-(1H-indol-3-yl)(3-methoxyphenyl)methyl)-4-methylenecyclopentane-1,1-dicarboxylate (2x)

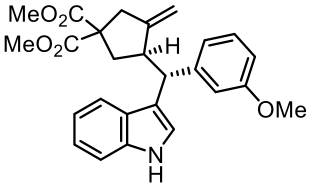

Prepared following general procedure **GP5** using dimethyl (*E*)-2-(prop-2-yn-1-yl)-2-(3-(3-methoxyphenyl)allyl)malonate (**1i**, 31.6 mg, 0.100 mmol) and indole (14.1 mg, 0.120 mmol, 1.2 equiv) as a nucleophile in 1,4-dioxane (1.0 mL, 0.1M), at 25 ºC for 6 h. The crude product was purified by FCC (silica, CyH:AcOEt 90:10 to 85:15). Compound **2x** was obtained as a white solid (42.4 mg, 0.978 mmol, 98% yield) with >99:1 *er*.

Spectroscopic data matched those reported in the literature. The absolute configuration was assigned by comparison to **2v**.

M.p. 67 ºC.

^{1}H NMR (500 MHz, $CDCl_3$) δ 8.09 (s, 1H), 7.57 (dq, *J* = 8.0, 0.8 Hz, 1H), 7.30 (dt, *J* = 8.1, 0.9 Hz, 1H), 7.17 – 7.10 (m, 2H), 7.05 (ddd, *J* = 8.0, 7.0, 1.0 Hz, 1H), 6.94 (dt, *J* = 7.7, 1.3 Hz, 1H), 6.89 (dd, *J* = 2.6, 1.6 Hz, 1H), 6.67 (ddd, *J* = 8.2, 2.6, 0.9 Hz, 1H), 4.81 (dt, *J* = 2.5, 1.4 Hz, 1H), 4.20 (h, *J* = 1.0 Hz, 1H), 4.15 – 4.09 (m, 1H), 3.74 (s, 3H), 3.72 (s, 3H), 3.63 (s, 3H), 3.52 (dq, *J* = 8.3, 1.9 Hz, 1H), 3.12 (dq, *J* = 16.0, 2.4 Hz, 1H), 2.90 (dq, *J* = 15.9, 1.5 Hz, 1H), 2.74 (ddd, *J* = 13.7, 7.9, 1.7 Hz, 1H), 1.96 (dd, *J* = 13.7, 8.5 Hz, 1H).

^{13}C NMR (126 MHz, $CDCl_3$) δ 172.4, 172.2, 159.5, 148.9, 146.2, 136.3, 129.1, 127.3, 122.1, 121.5, 121.2, 119.5, 119.5, 118.6, 114.8, 111.2, 111.1, 110.2, 58.5, 55.2, 52.9, 52.8, 48.1, 46.8, 41.9, 40.0.

HRMS (ESI +) calculated for $[C_{26}H_{28}NO_5]^{+\cdot}$ $[M+H]^{+\cdot}$ 434.1962 m/z; found 434.1947 m/z.

SFC (OD (100 × 3 mm, 3 μm), 85:15 CO_2:MeOH, 1.2 mL/min, 35 ºC, BPR 150 bar, 210 nm): en1 (major, 99.5%) 2.67 min, en2 (minor, 0.5%) 3.32 min.

[α]$^{24.6}_{D}$ +4.64 (c 1.00, $CHCl_3$).

Dimethyl (*R*)-3-((*R*)-(1H-indol-3-yl)(3-nitrophenyl)methyl)-4-methylenecyclopentane-1,1-dicarboxylate (2y)

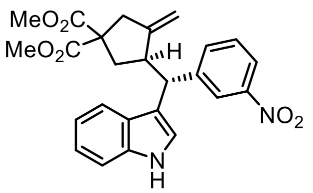

Prepared following general procedure **GP5** using dimethyl (*E*)-2-(prop-2-yn-1-yl)-2-(3-(3-nitrophenyl)allyl)malonate (**1j**, 33.1 mg, 0.100 mmol) and indole (14.1 mg, 0.120 mmol, 1.2 equiv) as a nucleophile in 1,4-dioxane (1.0 mL, 0.1M), at 25 ºC for 6 h. The crude product was purified by FCC (silica, CyH:AcOEt 90:10 to 80:20). Compound **2y** was obtained as a yellow solid (42.7 mg, 0.952 mmol, 95% yield) with >99:1 *er*.

Spectroscopic data matched those reported in the literature. The absolute configuration was assigned by comparison to **2v**.

M.p. 57–61 ºC

^{1}H NMR (300 MHz, $CDCl_3$) δ 8.23 – 8.12 (m, 2H), 7.99 (ddd, *J* = 8.2, 2.3, 1.0 Hz, 1H), 7.68 – 7.60 (m, 1H), 7.45 (d, *J* = 7.9 Hz, 1H), 7.40 (d, *J* = 7.9 Hz, 1H), 7.37 – 7.33 (m, 1H), 7.23 (d, *J* = 2.3 Hz, 1H), 7.20 – 7.11 (m, 1H), 7.10 – 6.98 (m, 1H), 4.80 (s, 1H), 4.26 (d, *J* = 10.6 Hz, 1H), 4.02 (s, 1H), 3.73 (s, 3H), 3.64 (s, 3H), 3.54 – 3.44 (m, 1H), 3.16 (dq, *J* = 15.9, 1.9 Hz, 1H), 2.90 (d, *J* = 16.8 Hz, 1H), 2.85 – 2.76 (m, 1H), 2.00 (dd, *J* = 13.8, 7.7 Hz, 1H).

^{13}C NMR (75 MHz, $CDCl_3$) δ 172.2, 148.5, 148.2, 146.7, 136.4, 135.0, 129.1, 126.9, 123.4, 122.6, 121.7, 121.5, 119.9, 119.02, 117.3, 111.4, 110.9, 58.6, 53.0, 52.9, 47.8, 47.1, 41.6, 39.8

HRMS (ESI +) calculated for $[C_{25}H_{24}N_2NaO_6]^{+\cdot}$ $[M+H]^{+\cdot}$ 471.1527 m/z; found 471.1540 m/z.

SFC (IB-N (100 × 3 mm, 3 μm), 70:30 CO_2:*i*-PrOH, 1.2 mL/min, 35 ºC, BPR 150 bar, 210 nm): en1 (major, 99.5%) 1.43 min, en2 (minor, 0.5%) 1.95 min.

[α]$^{24.6}_{D}$ –13.45 (c 1.04, $CHCl_3$).

Dimethyl (*R*)-3-((*R*)-(1H-indol-3-yl)(2-methoxyphenyl)methyl)-4-methylenecyclopentane-1,1-dicarboxylate (2z)

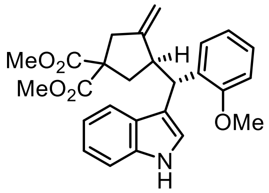

Prepared following general procedure **GP5** using dimethyl (*E*)-2-(prop-2-yn-1-yl)-2-(3-(2-methoxyphenyl)allyl)malonate (**1k**, 31.6 mg, 0.100 mmol) and indole (14.1 mg, 0.120 mmol, 1.2 equiv) as a nucleophile in 1,4-dioxane (1.0 mL, 0.1M), at 25 ºC for 6 h. The crude product was purified by FCC (silica, CyH:AcOEt 90:10 to 85:15). Compound **2z** was obtained as a white solid (37.5 mg, 0.865 mmol, 86% yield) with 93:7 *er*.

The absolute configuration was assigned by comparison to **2v**.

M.p. 54–60 ºC.

^{1}H NMR (300 MHz, $CDCl_3$) δ 8.01 (s, 1H), 7.56 (d, *J* = 7.9 Hz, 1H), 7.30 (d, *J* = 8.0 Hz, 1H), 7.23 (dd, *J* = 7.5, 1.6 Hz, 1H), 7.19 – 6.99 (m, 4H), 6.80 (t, *J* = 7.7 Hz, 2H), 4.78 (d, *J* = 10.8 Hz, 1H), 4.69 (s,

1H), 4.04 (s, 1H), 3.85 (s, 3H), 3.72 (s, 3H), 3.64 (s, 3H), 3.45 (q, *J* = 8.1 Hz, 1H), 3.21 (d, *J* = 15.7 Hz, 1H), 2.86 (d, *J* = 15.7 Hz, 1H), 2.77 (dd, *J* = 13.7, 7.9 Hz, 1H), 1.98 (dd, *J* = 13.7, 7.9 Hz, 1H).

^{13}C NMR (75 MHz, $CDCl_3$) δ 172.6, 172.3, 157.4, 149.4, 136.1, 133.1, 128.8, 127.8, 127.0, 122.0, 121.8, 120.6, 119.7, 119.4, 118.7, 111.0, 110.8, 108.9, 58.85 55.6, 52.9, 52.8, 46.8, 41.8, 40.2, 39.5.

HRMS (ESI +) calculated for $[C_{26}H_{27}NNaO_5]^{+\cdot}$ $[M+Na]^{+\cdot}$ 456.1781 m/z; found 456.1795 m/z.

SFC (IC (100 × 3 mm, 3 μm), 90:10 CO_2:EtOH, 1.2 mL/min, 35 ºC, BPR 150 bar, 210 nm): en1 (minor, 7%) 2.13 min, en2 (major, 93%) 2.55 min.

$[\alpha]^{24.6}_D$ –35.60 (c 1.10, $CHCl_3$).

Dimethyl (*R*)-3-((*R*)-(1H-indol-3-yl)(2-fluorophenyl)methyl)-4-methylenecyclopentane-1,1-dicarboxylate (2aa)

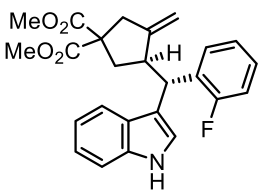

Prepared following general procedure **GP5** using dimethyl (*E*)-2-(prop-2-yn-1-yl)-2-(3-(2-fluorophenyl)allyl)malonate (**1l**, 30.4 mg, 0.100 mmol) and indole (14.1 mg, 0.120 mmol, 1.2 equiv) as a nucleophile in 1,4-dioxane (1.0 mL, 0.1M), at 25 ºC for 30 h. The crude product was purified by FCC (silica, CyH:AcOEt 90:10 to 80:20). Compound **2aa** was obtained as a white solid (31.0 mg, 0.736 mmol, 74% yield) with 98.5:1.5 *er*.

The absolute configuration was assigned by comparison to **2v**.

M.p. 54–58 ºC.

^{1}H NMR (300 MHz, $CDCl_3$) δ 8.07 (s, 1H), 7.56 (d, *J* = 7.7 Hz, 1H), 7.36 – 7.26 (m, 3H), 7.22 (d, *J* = 2.3 Hz, 1H), 7.19 – 7.12 (m, 1H), 7.11 – 7.02 (m, 2H), 7.01 – 6.92 (m, 2H), 4.76 (s, 1H), 4.54 (d, *J* = 11.1 Hz, 1H), 4.07 (s, 1H), 3.72 (s, 3H), 3.64 (s, 3H), 3.54 – 3.43 (m, 1H), 3.26 – 3.13 (m, 1H), 2.92 – 2.83 (m, 1H), 2.82 – 2.73 (m, 1H), 1.97 (dd, *J* = 13.8, 7.6 Hz, 1H).

^{13}C NMR (75 MHz, $CDCl_3$) δ 172.4, 172.1, 149.0, 136.2, 131.4 (d, *J* = 13.8 Hz), 129.7 (d, *J* = 4.4 Hz), 127.7 (d, *J* = 8.4 Hz), 127.4, 124.0 (d, *J* = 3.4 Hz), 121.89, 121.87, 121.0 (d, *J* = 198.9 Hz), 119.2, 117.6, 115.3 (d, *J* = 23.1 Hz), 111.2, 109.8, 58.8, 52.9 (d, *J* = 5.9 Hz), 46.1, 41.7, 40.2.

^{19}F NMR (282 MHz, $CDCl_3$) δ -117.8.

HRMS (ESI +) calculated for $[C_{17}H_{17}FNaO_4]^{+\cdot}$ $[M+Na]^{+\cdot}$ 327.1003 m/z; found 327.1005 m/z.

SFC (OJ (100 × 3 mm, 3 μm), 80:20 CO_2:*i*-PrOH, 1.2 mL/min, 35 ºC, BPR 150 bar, 210 nm): en1 (minor, 1.5%) 2.28 min, en2 (major, 98.5%) 3.03 min.

$[\alpha]^{24.6}_D$ –23.33 (c 1.01, $CHCl_3$).

Dimethyl (*R*)-3-((*R*)-(1H-indol-3-yl)(naphthalen-1-yl)methyl)-4-methylenecyclopentane-1,1-dicarboxylate (2ab)

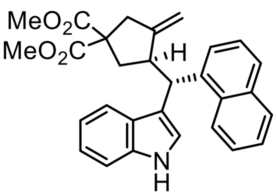

Prepared following general procedure **GP5** using dimethyl (*E*)-2-(3-(naphthalen-1-yl)allyl)-2-(prop-2-yn-1-yl)malonate (**1m**, 33.1 mg, 0.100 mmol) and indole (14.1 mg, 0.120 mmol, 1.2 equiv) as a nucleophile in 1,4-dioxane (1.0 mL, 0.1M), at 25 ºC for 6 h. The crude product was purified by FCC (silica, CyH:AcOEt 90:10 to 80:20). Compound **2ab** was obtained as a white solid (32.3 mg, 0.712 mmol, 70% yield) with 98.5:1.5 *er*.

The absolute configuration was assigned by comparison to **2v**.

M.p. 68–71 ºC.

^{1}H NMR (300 MHz, , $CDCl_3$) δ 8.39 – 8.23 (m, 1H), 7.99 (s, 1H), 7.82 (dd, *J* = 7.7, 1.8 Hz, 1H), 7.68 (d, *J* = 8.1 Hz, 1H), 7.60 (dd, *J* = 7.3, 1.2 Hz, 1H), 7.54 – 7.33 (m, 4H), 7.31 – 7.26 (m, 1H), 7.18 – 7.06 (m, 2H), 6.99 (ddd, *J* = 8.1, 7.0, 1.1 Hz, 1H), 5.19 (d, *J* = 9.4 Hz, 1H), 4.73 – 4.62 (m, 1H), 4.25 – 4.15 (m, 1H), 3.70 (s, 3H), 3.67 (s, 3H), 3.13 (dd, *J* = 16.4, 2.2 Hz, 1H), 2.94 (d, *J* = 16.2 Hz, 1H), 2.69 (dd, *J* = 13.5, 7.7 Hz, 1H), 2.23 (dd, *J* = 13.6, 9.1 Hz, 1H).

^{13}C NMR (75 MHz, $CDCl_3$) δ 172.5, 172.4, 149.4, 140.5, 136.3, 134.0, 132.3, 129.0, 127.4, 126.8, 126.0, 125.5, 125.3, 125.14, 123.6, 122.4, 122.1, 119.6, 119.6, 118.7, 111.1, 109.6, 58.5, 52.9, 52.9, 47.5, 41.9, 41.6, 39.8.

HRMS (ESI +) calculated for $[C_{29}H_{27}NNaO_4]^{+\cdot}$ $[M+Na]^{+\cdot}$ 476.1832 m/z; found 476.1840 m/z.

SFC (IC (100 × 3 mm, 3 µm), 85:15 CO_2:*i*-PrOH, 1.2 mL/min, 35 ºC, BPR 150 bar, 210 nm): en1 (minor, 1.5%) 2.02 min, en2 (major, 98.5%) 2.55 min.

$[\alpha]^{24.6}_D$ –45.81 (c 1.29, $CHCl_3$).

Dimethyl (*S*)-3-(2-(1H-indol-3-yl)propan-2-yl)-4-methylenecyclopentane-1,1-dicarboxylate (2ac)

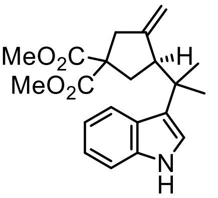

Prepared following general procedure **GP5** using dimethyl (*E*)-2-(3-(naphthalen-1-yl)allyl)-2-(prop-2-yn-1-yl)malonate (**1n**, 23.8 mg, 0.100 mmol) and indole (23.4 mg, 0.200 mmol, 2.0 equiv) as a nucleophile in toluene (1.0 mL, 0.1M), at -10 ºC for 14 h. The crude product was purified by FCC (silica, CyH:AcOEt 90:10 to 85:15). Compound **2ac** was obtained as a white solid (32.3 mg, 0.712 mmol, 70% yield) with 99:1 *er*. Spectroscopic data matched those reported in the literature.[18] The absolute configuration was assigned by comparison to **2v**.

M.p. 114–117 ºC.

^{1}H NMR (300 MHz, $CDCl_3$) δ 7.94 (s, 1H), 7.84 (d, *J* = 8.0 Hz, 1H), 7.35 (d, *J* = 8.0 Hz, 1H), 7.24 – 7.14 (m, 1H), 7.08 (td, *J* = 7.6, 1.2 Hz, 1H), 6.93 (d, *J* = 2.4 Hz, 1H), 4.93 (s, 1H), 4.45 (s, 1H), 3.67 (s, 6H), 3.45 (t, *J* = 8.7 Hz, 1H), 2.91 – 2.73 (m, 2H), 2.42 (dd, *J* = 13.7, 8.4 Hz, 1H), 1.88 (dd, *J* = 13.7, 9.2 Hz, 1H), 1.45 (s, 3H), 1.36 (s, 3H).

^{13}C NMR (75 MHz, $CDCl_3$) δ 172.3, 172.1, 149.1, 137.3, 125.8, 124.7, 121.8, 121.3, 121.1, 119.2 111.4, 110.6, 58.8, 52.7, 49.6, 44.2, 38.0, 36.6, 25.7, 24.8.

SFC (OJ (100 × 3 mm, 3 μm), 80:20 CO_2:MeOH, 1.2 mL/min, 35 ºC, BPR 150 bar, 210 nm): en1 (minor, 1%) 2.35 min, en2 (minor, 99%) 3.11 min.

[α]$^{24.6}$$_D$ +32.90 (c 0.84, $CHCl_3$).

Dimethyl (*S*)-3-((*S*)-2-Ethoxy-6-methylhept-5-en-2-yl)-4-methylenecyclopentane-1,1-dicarboxylate (2ad)

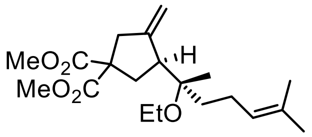

Prepared following general procedure **GP5** using dimethyl (*E*)-2-(3,7-dimethylocta-2,6-dien-1-yl)-2-(prop-2-yn-1-yl)malonate (**1n**, 30.6 mg, 0.100 mmol) and EtOH (29.2 µL, 0.500 mmol, 5.0 equiv) as a nucleophile in 1,4-dioxane (1.0 mL, 0.1M), at 23 ºC for 14 h. The crude product was purified by FCC (silica, CyH:AcOEt 90:10 to 85:15). Compound **2ad** was obtained as a white solid (31.3 mg, 0.925 mmol, 92% yield) with >99:1 *er*.

Spectroscopic data matches those reported in the literature.[19] The absolute configuration was assigned by comparison to **2v**.

^{1}H NMR (300 MHz, $CDCl_3$) δ 5.17 – 5.07 (m, 1H), 5.06 – 4.99 (m, 1H), 4.93 – 4.86 (m, 1H), 3.75 (s, 3H), 3.73 (s, 3H), 3.36 (qd, *J* = 6.9, 1.2 Hz, 2H), 3.03 – 2.87 (m, 2H), 2.87 – 2.77 (m, 1H), 2.56 (ddd, *J* = 13.7, 8.4, 1.7 Hz, 1H), 2.15 (dd, *J* = 13.8, 8.8 Hz, 1H), 2.08 – 1.95 (m, 2H), 1.70 (q, *J* = 1.3 Hz, 3H), 1.69 – 1.51 (m, 6H), 1.15 (t, *J* = 7.0 Hz, 3H), 1.08 (s, 3H).

^{13}C NMR (75 MHz, $CDCl_3$) δ 172.3, 172.1, 148.8, 131.5, 124.8, 110.6, 78.2, 58.7, 56.2, 52.8, 52.8, 48.4, 44.0, 35.8, 35.4, 25.8, 22.1, 20.1, 17.8, 15.9.

[α]$^{24.6}$$_D$ +11.7 (c 0.84, $CHCl_3$).

3-((*R*)-((*R*)-4,4-bis(methoxymethyl)-2-methylenecyclopentyl)(phenyl)methyl)-1H-indole (2ae)

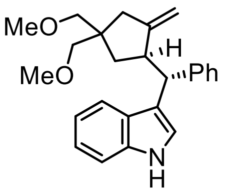

Prepared following general procedure **GP5**, (*E*)-(4,4-bis(methoxymethyl)hept-1-en-6-yn-1-yl)benzene (**1c**, 25.8 mg, 0.100 mmol) and indole (35.1 mg, 0.300 mmol,3.0 equiv) as a nucleophile in 1,4-dioxane (1.0 mL, 0.1M), at 25 ºC for 6 h. The crude product was purified by FCC (silica, CyH:AcOEt 90:10). Compound **2ae** was obtained as a colorless oil (23.5 mg, 0.626 mmol, 63% yield) with 96:4 *er*.

The absolute configuration was assigned by comparison to **2v**.

HRMS (ESI +) calculated for $[C_{25}H_{29}NNaO_2]^{+\cdot}$ $[M+Na]^{+\cdot}$ 398.2090 m/z; found 398.2090 m/z.

^{1}H NMR (300 MHz, $CDCl_3$) δ 8.01 (s, 1H), 7.53 (d, *J* = 8.0 Hz, 1H), 7.38 – 7.30 (m, 3H), 7.27 – 7.20 (m, 2H), 7.18 – 7.09 (m, 3H), 7.04 (ddd, *J* = 8.0, 7.1, 1.1 Hz, 1H), 4.75 (s, 1H), 4.19 (d, *J* = 10.0 Hz, 1H), 4.15 (d, *J* = 0.9 Hz, 1H), 3.45 – 3.32 (m, 4H), 3.29 (s, 3H), 3.25 – 3.20 (m, 4H), 2.39 – 2.20 (m, 2H), 1.91 (dd, *J* = 14.1, 8.1 Hz, 1H), 1.53 (dd, *J* = 13.8, 7.9 Hz, 1H).

^{13}C NMR (75 MHz, $CDCl_3$) δ 152.2, 144.9, 136.2, 128.6, 127.9, 127.5, 125.8, 121.9, 121.5, 119.5, 119.2, 119.1, 110.9, 108.9, 75.3, 59.3, 59.2, 48.3, 46.9, 45.7, 40.2, 37.9.

SFC (IB-N (100 × 3 mm, 3 µm), 80:20 CO_2:MeOH, 1.2 mL/min, 35 ºC, BPR 150 bar, 210 nm): en1 (major, 96%) 2.62 min, en2 (minor, 4%) 3.65 min.

[α]$^{24.6}_{D}$ +45.5 (c 0.27, $CHCl_3$).

Diisopropyl (*R*)-3-((*R*)-(1H-indol-3-yl)(phenyl)methyl)-4-methylenecyclopentane-1,1-dicarboxylate (2af)

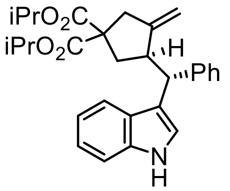

Prepared following general procedure **GP5**, using diisopropyl 2-cinnamyl-2-(prop-2-yn-1-yl)malonate (**1b**, 34.2 mg, 0.100 mmol) and indole (14.1 mg, 0.120 mmol, 1.2 equiv) as a nucleophile in 1,4-dioxane (1.0 mL, 0.1M), at 25 ºC for 6 h. The crude product was purified by FCC (silica, CyH:AcOEt 90:10). Compound **2af** was obtained as a colorless oil (41.1 mg, 0.916 mmol, 92% yield) with 93:7 *er*.

The absolute configuration was assigned by comparison to **2v**.

^{1}H NMR (300 MHz, $CDCl_3$) δ 8.08 (s, 1H), 7.54 (d, *J* = 7.9 Hz, 1H), 7.37 – 7.28 (m, 3H), 7.25 – 7.18 (m, 2H), 7.17 – 7.07 (m, 3H), 7.07 – 6.98 (m, 1H), 5.06 (hept, 1H), 4.95 (hept, *J* = 6.3 Hz, 1H), 4.78 (s, 1H), 4.17 (d, *J* = 10.3 Hz, 1H), 4.13 (s, 1H), 3.60 – 3.44 (m, 1H), 3.17 – 3.00 (m, 1H), 2.86 (dd, *J* = 15.9, 1.3 Hz, 1H), 2.71 (ddd, *J* = 13.5, 7.8, 1.4 Hz, 1H), 1.95 (dd, *J* = 13.6, 8.5 Hz, 1H), 1.21 (d, *J* = 6.3 Hz, 6H), 1.18 (d, *J* = 6.2 Hz, 3H), 1.09 (d, *J* = 6.3 Hz, 3H).

^{13}C NMR (75 MHz, $CDCl_3$) δ 171.4, 171.3, 149.3, 144.5, 136.2, 128.5, 128.1, 127.23 126.0, 121.9, 121.4, 119.4, 119.3, 118.7, 111.04 109.7, 68.9, 68.8, 58.53, 47.9, 46.8, 41.7, 39.6, 21.6, 21.5, 21.5.

HRMS (ESI +) calculated for $[C_{29}H_{33}NNaO_4]^{+\cdot}$ $[M+Na]^{+\cdot}$ 482.2302 m/z; found 482.2312 m/z.

SFC (IB-N (100 × 3 mm, 3 µm), 85:15 CO_2:MeOH, 1.2 mL/min, 35 ºC, BPR 150 bar, 210 nm): en1 (major, 93%) 2.14 min, en2 (minor, 7%) 2.70 min.

[α]$^{24.6}_{D}$ –0.4 (c 1.14, $CHCl_3$).

3-((*R*)-((*R*)-4-Methylene-1-tosylpyrrolidin-3-yl)(phenyl)methyl)-1H-indole (2ag)

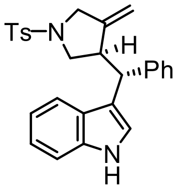

Prepared following general procedure **GP5**, using **Au8** (3.5 mg, 3 mol%), **(*R*)-Ag5** (3.3 mg, 3 mol%), *N*-(*E*)-cinnamyl-4-methyl-*N*-(prop-2-yn-1-yl)benzenesulfonamide (**1d**, 32.5 mg, 0.100 mmol) and indole (17.6 mg, 0.150 mmol, 1.5 equiv) as a nucleophile in toluene (1.0 mL, 0.1M), at 25 ºC for 24 h. The crude product was purified by FCC (silica, CyH:AcOEt 80:20). Compound **2ag** was obtained as an off-white solid (40.3 mg, 0.911 mmol, 91% yield) with 99:1 *er*.

Spectroscopic data matched those reported in the literature. The absolute configuration was assigned by comparison to **2ai.**

^{1}H NMR (300 MHz, $CDCl_3$) δ 8.06 (s, 1H), 7.60 (d, *J* = 8.2 Hz, 2H), 7.33 (d, *J* = 8.2 Hz, 2H), 7.24 (d, *J* = 8.1 Hz, 2H), 7.20 – 6.99 (m, 7H), 4.79 – 4.70 (m, 1H), 4.28 – 4.19 (m, 1H), 3.96 (d, *J* = 10.2 Hz, 1H), 3.91 – 3.79 (m, 2H), 3.50 – 3.30 (m, 3H), 2.43 (s, 3H).

^{13}**C NMR** (75 MHz, $CDCl_3$) δ 145.2, 143.7, 143.3, 136.5, 132.9, 129.8, 128.4, 128.3, 127.9, 127.0, 126.4, 122.4, 121.2, 119.6, 119.4, 118.1, 111.3, 110.3, 53.2, 52.5, 47.9, 45.9, 21.7.

SFC (IC (100 × 3 mm, 3 μm), 80:20 CO_2:EtOH, 1.2 mL/min, 35 ºC, BPR 150 bar, 210 nm): en1 (minor, 1%) 4.66 min, en2 (major, 99%) 5.11 min.

$[\alpha]^{24.6}_D$ –38.4 (c 0.54, $CHCl_3$).

(*R*)-3-((*R*)-Methoxy(phenyl)methyl)-4-methylene-1-tosylpyrrolidine (2ah)

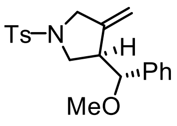

Prepared following general procedure **GP5**, using **Au8** (2.4 mg, 2 mol%), **(*R*)-Ag5** (2.0 mg, 2 mol%), *N*-(*E*)-cinnamyl-4-methyl-*N*-(prop-2-yn-1-yl)benzenesulfonamide (**1d**, 32.5 mg, 0.100 mmol) and anhydrous MeOH (20.2 μL, 0.500 mmol, 5.0 equiv) as a nucleophile in toluene (1.0 mL, 0.1M), at 25 ºC for 14 h. The crude product was purified by FCC (silica, CyH:AcOEt 80:20). Compound **2ah** was obtained as white solid (34.8 mg, 0.974 mmol, 97% yield) with 98:2 *er*.

Spectroscopic data matched those reported in the literature. The absolute configuration was assigned by comparison to **2ai**.

^{1}**H NMR** (300 MHz, $CDCl_3$) δ 7.77 – 7.69 (m, 2H), 7.38 – 7.26 (m, 6H), 7.16 (dd, *J* = 7.6, 1.8 Hz, 2H), 4.76 (q, *J* = 1.9 Hz, 1H), 4.20 (q, *J* = 2.2 Hz, 1H), 3.91 (d, *J* = 8.3 Hz, 1H), 3.88 – 3.80 (m, 1H), 3.76 – 3.64 (m, 2H), 3.29 – 3.19 (m, 1H), 3.10 (s, 3H), 2.91 – 2.78 (m, 1H), 2.44 (s, 3H).

^{13}**C NMR** (75 MHz, $CDCl_3$) δ 143.7, 143.6, 139.5, 133.0, 129.8, 128.4, 128.2, 128.0, 127.7, 110.1, 83.9, 57.0, 52.4, 50.7, 50.6, 21.7.

SFC (IA (100 × 3 mm, 3 μm), 85:15 CO_2:MeOH, 1.2 mL/min, 35 ºC, BPR 150 bar, 210 nm): en1 (major, 98%) 1.27 min, en2 (minor, 2%) 2.31 min.

$[\alpha]^{24.6}_D$ +63.0 (c 1.62, $CHCl_3$).

(*1R,5R,6S*)-6-Phenyl-3-tosyl-3-azabicyclo[3.1.0]hexane-1-carbaldehyde (2ai)

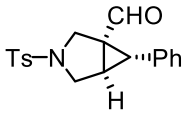

Prepared following general procedure **GP5**, using **Au8** (3.5 mg, 3 mol%), **(*R*)-Ag5** (3.3 mg, 3 mol%), *N*-(*E*)-cinnamyl-4-methyl-*N*-(prop-2-yn-1-yl)benzenesulfonamide (**1d**, 32.5 mg, 0.100 mmol) and diphenyl sulfoxide (30.3 mg, 0.150 mmol, 1.5 equiv) as a nucleophile in toluene (1.0 mL, 0.1M), at 0 ºC for 24 h. The crude product was purified by PTLC (silica, pentane: CH_2Cl_2 30:70). Compound **2ai** was obtained as white solid (32.1 mg, 0.940 mmol, 94% yield) with 99:1 *er*.

Spectroscopic data matched those reported in the literature.[20] The absolute configuration was assigned by X-Ray crystallography.

^{1}**H NMR** (300 MHz, $CDCl_3$) δ 8.71 (s, 1H), 7.78 – 7.70 (m, 2H), 7.40 – 7.27 (m, 6H), 7.25 – 7.21 (m, 1H), 3.84 (d, *J* = 9.6 Hz, 1H), 3.73 (d, *J* = 10.0 Hz, 1H), 3.47 (d, *J* = 10.0 Hz, 1H), 3.24 – 3.14 (m, 1H), 3.11 (d, *J* = 5.4 Hz, 1H), 2.83 (dd, *J* = 5.4, 4.0 Hz, 1H), 2.44 (s, 3H).

^{13}C NMR (75 MHz, $CDCl_3$) δ 197.4, 144.2, 133.4, 132.7, 130.0, 129.05, 129.00, 127.8, 49.2, 48.2, 45.0, 34.2, 29.9, 21.7.

SFC (OD (100 × 3 mm, 3 μm), 70:30 CO_2:EtOH, 1.2 mL/min, 35 ºC, BPR 150 bar, 210 nm): en1 (minor, 1%) 0.83 min, en2 (minor, 99%) 1.25 min.

[α]$^{24.6}_{D}$ –63.5 (c 0.40, $CHCl_3$).

Dimethyl (*1R,8aS*)-1-Phenyl-4,6,8,8a-tetrahydro-1H-cyclopenta[c]oxepine-7,7(3H)-dicarboxylate (3a)

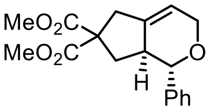

To a solution of **2r** (13.8 mg, 40.0 μmol, 98.5:1.5 *er*) in anhydrous CH_2Cl_2 (0.1M) was added Grubbs-II catalyst (1.7 mg, 2 μmol, 5 mol%). The mixture was heated to 50 °C and left stirring overnight. Next day, after 17h, the reaction was filtered through a silica-filled glass pipette washing with CH_2Cl_2. Compound **3a** was obtained as an orange oil (11.8 mg, 37.3 μmol, 93% yield) with 99:1 *er*.

Spectroscopic data matched those reported in the literature.[21] The absolute configuration was assigned by comparison of the configuration of the starting material.

^{1}H NMR (300 MHz, $CDCl_3$) δ 7.39 – 7.27 (m, 5H), 5.77 – 5.65 (m, 1H), 4.98 (d, *J* = 6.2 Hz, 1H), 4.18 (tq, *J* = 4.9, 2.4 Hz, 2H), 3.71 (s, 3H), 3.66 (s, 3H), 3.13 – 2.92 (m, 2H), 2.91 – 2.78 (m, 1H), 2.05 (dd, *J* = 12.8, 7.3 Hz, 1H), 1.64 (t, *J* = 12.9 Hz, 1H).

^{13}C NMR (75 MHz, $CDCl_3$) δ 172.89, 172.21, 139.62, 138.66, 128.52, 127.61, 126.84, 118.57, 63.76, 56.66, 53.04, 52.91, 41.82, 38.48, 35.66.

SFC (IG (100 × 3 mm, 3 μm), 95:5 CO_2:MeOH, 1.2 mL/min, 35 ºC, BPR 150 bar, 210 nm): en1 (major, 99%) 2.60 min, en2 (minor, 1%) 3.795 min.

[α]$^{24.6}_{D}$ +73.1 (c 0.45, $CHCl_3$).

Dimethyl (*5aR,8aS*)-5a-Methyl-1-phenyl-4,5,5a,6,8,8a-hexahydrocyclopenta[c]azepine-7,7(3H)-dicarboxylate (3b)

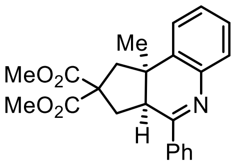

To a MW vial containing $Pd(OAc)_2$ (2.1 mg, 9.5 μmol, 10 mol%), PPh_3 (5.0 mg, 19 μmol, 20 mol%) and K_2CO_3 (26.0 mg, 19.0 μmol, 2 equiv) wad added **2k** (48 mg, 95.0 μmol, 98.5:1.5 *er*) as a solution in anhydrous and degassed DMF (0.63 mL). The mixture was heated to 110 °C and left stirring for 24h. The solvent was evaporated and the crude was purified by PTLC (pentane:Et_2O 90:10). Compounds **3b** was obtained as a white solid (25.6 mg, 65.4 μmol, 69% yield) with 98.5:1.5 *er*.

Spectroscopic data matched those reported in the literature.[13] The absolute configuration was assigned based on the configuration of the starting material.

^{1}H NMR (300 MHz, $CDCl_3$) δ 8.11 (dd, *J* = 6.7, 3.0 Hz, 2H), 7.50 (ddt, *J* = 9.6, 6.4, 2.7 Hz, 4H), 7.39 (dd, *J* = 7.3, 1.7 Hz, 1H), 7.33 – 7.26 (m, 2H), 7.23 (dd, *J* = 7.3, 1.6 Hz, 1H), 3.80 (s, 3H), 3.51 (s, 3H),

3.43 (d, J = 14.1 Hz, 1H), 3.31 (dd, J = 12.3, 7.7 Hz, 1H), 2.66 (dd, J = 13.7, 7.7 Hz, 1H), 2.55 (d, J = 14.2 Hz, 1H), 2.18 (dd, J = 13.7, 12.4 Hz, 1H), 1.21 (s, 3H).

^{13}C NMR (75 MHz, $CDCl_3$) δ 173.37, 171.41, 165.80, 142.44, 138.64, 131.71, 130.79, 128.78, 128.57, 127.90, 127.74, 126.95, 125.66, 58.47, 53.23, 52.90, 46.91, 46.49, 44.51, 38.81, 28.67.

SFC (IG (100 × 3 mm, 3 μm), 85:15 CO_2:EtOH, 1.2 mL/min, 35 ºC, BPR 150 bar, 210 nm): en1 (major, 98.5%) 1.05 min, en2 (minor, 1.5%) 1.30 min.

$[\alpha]^{24.6}_D$ +217.0 (c 0.87, $CHCl_3$).

(*R*)-4-((*R*)-Methoxy(phenyl)methyl)-3-methylene-1-tosylpyrrolidin-2-one (3c)

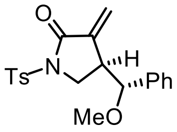

To a white suspension of **2w** (17.9 mg, 50.0 μmol, 96% ee) in HPLC-grade MeCN (0.33 mL) and distilled water (0.17 mL), was added *N*-hydroxyphtalamide (1.6 mg, 10.0 μmol, 20 mol%). Then $NaClO_2$ (17.5 mg, 155 μmol, 3.1 equiv) was added portionwise turning the white suspension into an orange one. The mixture is then warmed up to 50 ºC and left stirring for 24 h (the orange suspension turned into a colorless solution upon warming up). The reaction was worked up by diluting it in AcOEt (2 mL) and an aqueous 0.1 M solution of $Na_2S_2O_3$ (2 mL). The organic phase was then washed with an aqueous saturated $NaHCO_3$ solution (2 mL), water (2 mL) and brine (2 mL), dried over $MgSO_4$, filtered, and evaporated. The crude was purified by PTLC (pentane:Et_2O 60:40). Compound **3c** was obtained as a colorless oil (9.2 mg, 25.0 μmol, 50% yield) with 96% ee.

^{1}H NMR (500 MHz, $CDCl_3$) δ 7.97 – 7.93 (m, 2H), 7.38 – 7.30 (m, 6H), 7.25 – 7.18 (m, 2H), 5.99 (d, J = 2.4 Hz, 1H), 4.68 (d, J = 2.0 Hz, 1H), 4.12 (dd, J = 10.5, 3.2 Hz, 1H), 4.00 (d, J = 7.9 Hz, 1H), 3.83 (dd, J = 10.4, 8.1 Hz, 1H), 3.17 (s, 3H), 2.44 (s, 3H).

^{13}C NMR (126 MHz, $CDCl_3$) δ 165.8, 145.3, 138.4, 138.1, 135.2, 129.8, 128.7, 128.4, 127.8, 123.5, 85.1, 57.1, 46.9, 42.9, 21.8.

HRMS (ESI +) calculated for $[C_{20}H_{22}NO_4S]^{+\cdot}$ $[M+H]^{+\cdot}$ 372.1264 m/z; found 372.1266 m/z.

SFC (IC (100 × 3 mm, 3 μm), 80:20 CO_2:ACN, 1.2 mL/min, 35 ºC, BPR 150 bar, 210 nm): en1 (minor, 2%) 2.26 min, en2 (major, 98%) 3.30 min.

$[\alpha]^{24.6}_D$ +93.9 (c 0.33, $CHCl_3$).

4.3 Assigment of Absolute Configuration

After revising previous reports for the preparation of enantioenriched products presented in this manuscript, we noticed that the absolute configuration had only been assigned once by the group of Michelet and coworkers.[14] The assignment was done by preparing the corresponding diastereomers of **2m** with *(S)*-α-methoxyphenyl acetic acid followed by their separation by column chromatography. The absolute configuration was then determined by comparing the chemical shift variation in the ^{1}H NMR spectra of the benzylic proton provoked by the shielding of the methoxy group (Scheme S1).[22,23,24] As a result the absolute configuration assigned by Michelet was (*S,R*)

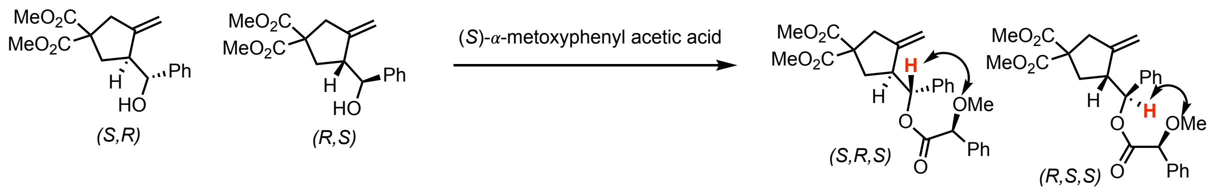

*Scheme S3 Diastereomers prepared from water addition product **2m** by Michelet, the H whose chemical shift was compared is highlighted in red.*

However, we observed discrepancies between the values of the optical rotation of our products **2m-o** and the ones reported in the literature. After obtaining the X-Ray structure of compound **2v** and assuming a uniform enantioselective model, we concluded that the absolute configuration of our products was (*S,R*). Hence, we consider that the absolute configuration reported by the group of Michelet was misassigned. Therefore, many posterior publications[25] that relied on this previously reported configuration should be revised.

5. DFT details

Calculations were carried out using the Gaussian09 package[26] at the density functional theory (DFT) level by means of the B3LYP[27] functional. Reported geometries and energies were calculated including the corrected dispersion GD3.[28] The 6-31G(d)[29] basis set was implemented on all atoms (C, H, N, O, F, P, S), except for Au for which the SDD[30] basis set and its corresponding Stuttgart/Dresden Effective Core Potential (ECP) were used. All systems were calculated in toluene whose solvation effects were accounted for using the implicit polarizable continuum model (PCM)[31]. Each stationary point was characterized by vibrational analysis. Transition states (TS) were identified by the presence of a single imaginary frequency while all minima presented only real frequencies. To further confirm the identity of the TS, relaxation experiments of the imaginary frequency were performed connecting reactants and products. Reported energies have been corrected with the thermal and entropic corrections by means of single point calculations at the 6-311G(d,p)[29] level on all atoms (C, H, N, O, F, P, S), except for Au for which the SDD basis set and ECP were used. All dataset collection of computational results of this manuscript is available in the ioChem-BD[32] repository and can be accessed through https://iochem-bd.iciq.es/browse/handle/100/60425.

Given the relatively large size of the system studied (1124 electrons) some assumptions were made to simplify the calculations. The chloride scavenging step was not considered and it was assumed to be quantitative and almost instantaneous. No conformational analysis was run, TS guesses were drawn assuming that the hydrogen bond donor group would place the chiral counterion close to the reaction center.

5.1 5.1 Discussion

The mechanism of this specific reaction has not, to our knowledge, been studied before. However, the mechanism should be like previously described cyclizations of 1,6-enynes (Scheme S1) followed by addition of the nucleophile to the gold(I) carbene intermediate generated upon attack of the alkene to the alkyne.[33] The stereospecific nucleophile attack would take place on the cyclopropyl gold carbene intermediate,[34] leading to a final product.

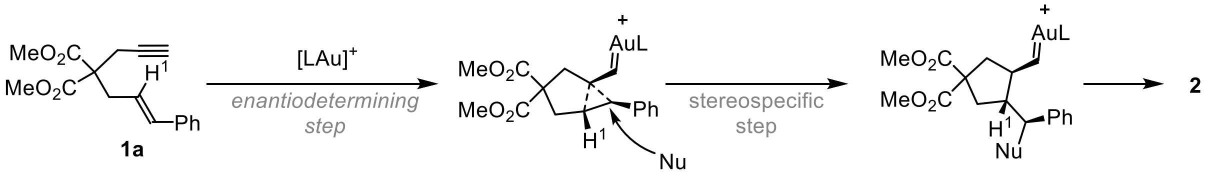

Scheme S4 Mechanism for a classical 1,6-enyne cycloisomerisation followed by nucleophile addition

The enantiodetermining step was studied for the gold(I)-catalyzed indole addition to **1a** using **(*R*)-Au4** and the corresponding counterion of **(*R*)-Ag6** leading to **2a** in 95:5 er. Two possible pathways were calculated (Figure S3), one for each enantiotopic face (Re or Si) of the alkene giving product R or S. Out of the different pathways for each possible product, two of them presented the lowest TS connecting intermediates 1a–2a and 3a–4a with 6.9 and 7.9 Kcal respectively. A Curtin-Hammet scenario was found (Figure S3), where the two orientations of the alkene (**int1a** and **int3a**) are in equilibrium, and the less stable intermediate (**int1a**) has the lowest energy barrier (2.0 Kcal/mol), giving rise to (*S*)-**2a**, in agreement with the experimental results. The hydrogen bond interactions were observed in all the intermediates calculated, which indicate that they serve as anchor for additional stabilizing interactions. Indeed, all intermediates found shared extended and strong attractive non-covalent interactions based on π–π or C-H–π systems. **TSint1a-2a** presented a dual hydrogen bond interaction to the oxygen of the phosphoramidate P=O (2.15 and 1.90 Å), which together with the T-shaped-π attractive interaction between the C-H of the urea and the π-system of binaphtol counterion puts closer the two parts. Additional interactions between the substrate and the catalyst appear as stabilizing interactions of the transition state such as a strong and extended sandwich-type π-π system (3.49 Å) as well as T-shaped-type between the carbonyl π system of the urea and the C-H in para position of the cynnamyl group (2.88 Å). On the other hand, in **TSint3a-4a** the hydrogen bond interactions to the P=O (2.00 Å) and S=O (2.00 Å) bonds, which approximates the counterion and the gold (I) complex favoring two strong attractive T-shaped-π interactions of the substrate with the anthracenyl motifs of gold (3.32 Å) and with the counterion (2.52 Å), play the major role in stabilizing the transition state.

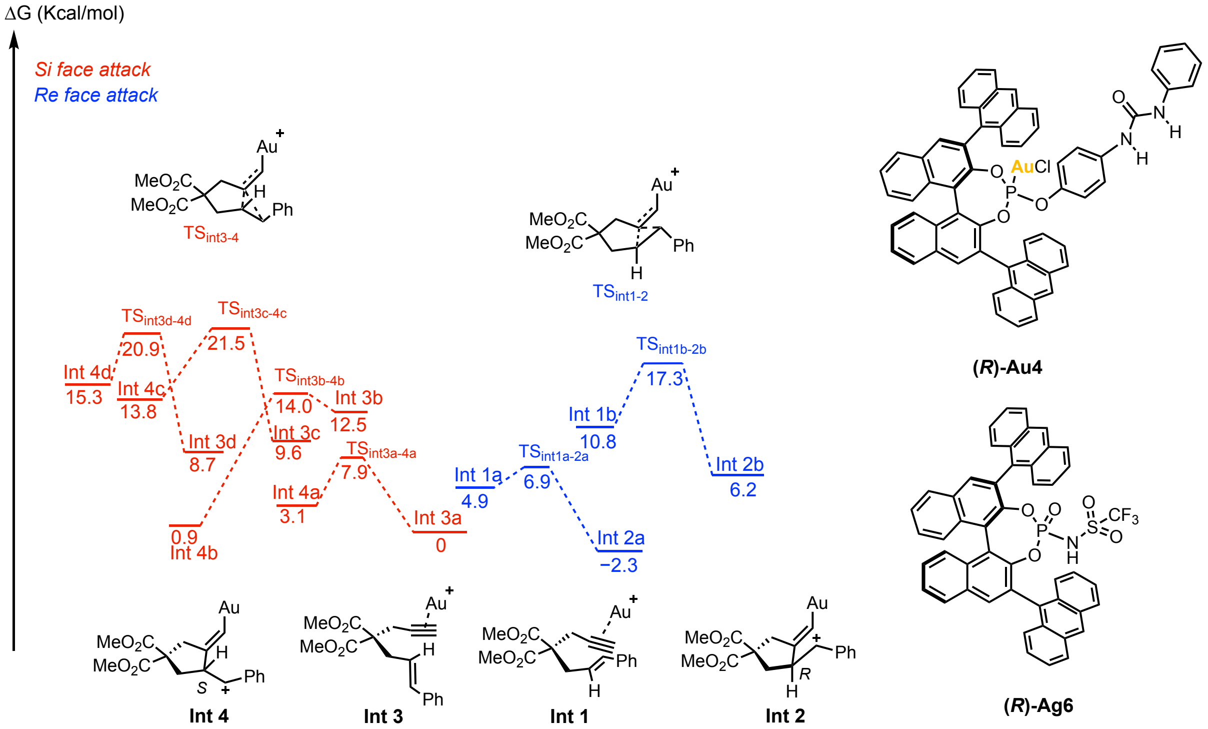

Figure S3 Free energy profiles for the 5-exo-dig cyclisation for the matched case of the cyclisation of ***1a*** *using* ***(R)-Au4*** *and* ***(R)-Ag6****. Pathways for the Si and Re face attack of the alkene are depicted in red and blue respectively. The energy values are given in Kcal/mol and represent the relative free energies.*

5.2 5.2 NCI plots

In the lowest-energy transition states, **TS**$_{\mathbf{Int1a\text{-}2a}}$ and **TS**$_{\mathbf{Int3a\text{-}4a}}$, we could observe larger green surfaces, which mainly correspond to aryl-aryl sandwich or T-shaped interactions, than in the other possible transition states coming for the other minima found. Strong attractive interactions are blue, corresponding to the hydrogen bond interactions, and strong repulsive interactions are red. Comparing the two TS, **TS**$_{\mathbf{Int1a\text{-}2a}}$ present weaker but more extended non-covalent interactions. Thus, we can confirm that non-covalent interactions that occur in the chiral pocket of this new type of catalysts have been found to be the key stereocontrol elements in cycloisomerization of enyne **1a**.

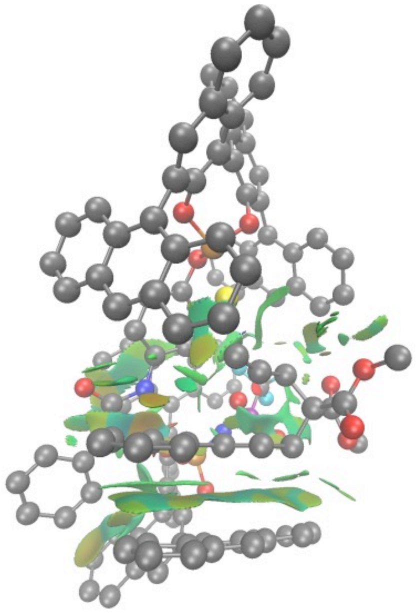

a) TS$_{int1a-2a}$

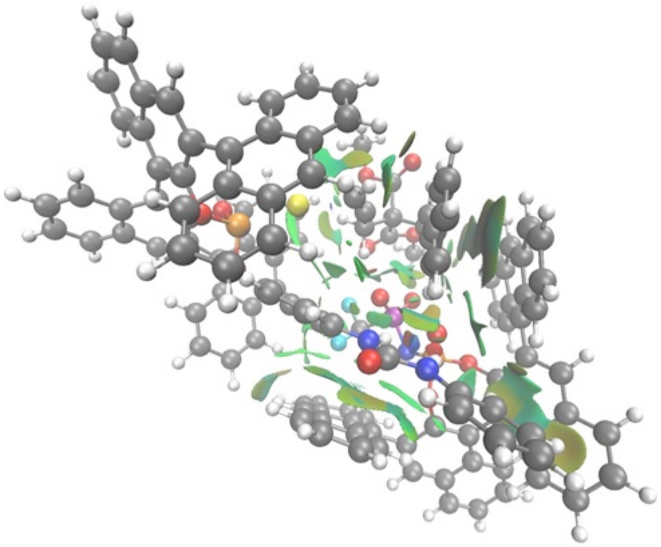

a) TS$_{int1a-2a}$ (zoomed in)

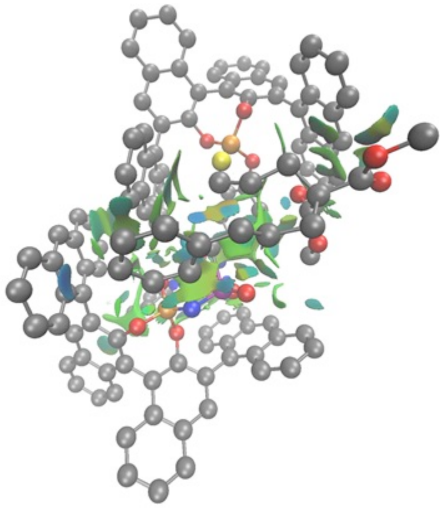

TS$_{int3a-4a}$

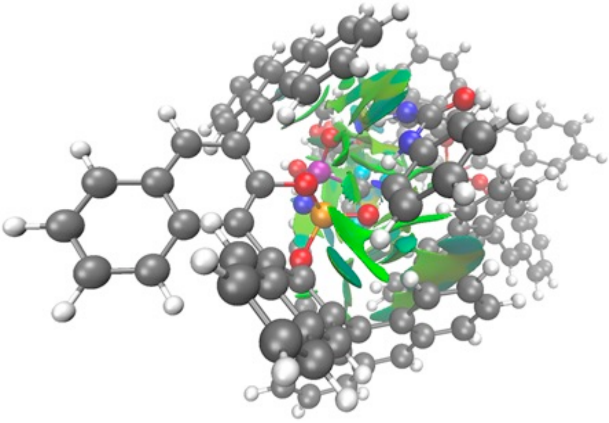

TS$_{int3a-4a}$ (zoomed in)

Figure S4 Color code: P, orange; Au, yellow; F, cyan; O, red; N, blue; S, purple; C, gray; and H, white. On the left hydrogens omitted for clarity. On the right, hydrogens are included to see the hydrogen bond interactions.

6. X-Ray structures

Complex Au4 (CCDC-2266407)

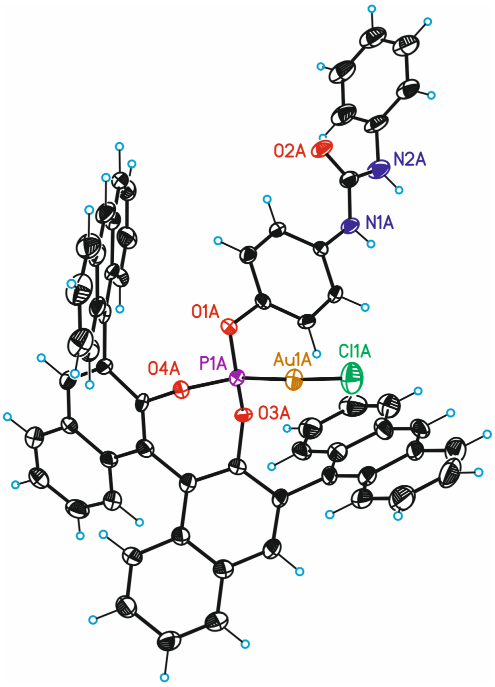

Identification code AMZ-04-067

Empirical formula C195.75 H130.75 Au3 Cl20.25 N6 O12 P3

Formula weight 4160.48

Temperature 0(2)K

Wavelength 0.71073 Å

Crystal system triclinic

Space group P 1

Unit cell dimensions a = 13.17025(19)Å a= 66.0966(13)°.

b = 17.6661(2)Å b = 78.2394(12)°.

c = 20.8530(3)Å g = 83.1689(12)°.

Volume4338.97(11) Å^3

Z 1

Density (calculated) 1.592 Mg/m^3

Absorption coefficient 2.934 mm^{-1}

F(000) 2070

Crystal size 0.700 x 0.020 x 0.020 mm^3

Theta range for data collection 3.455 to 30.414°.

Index ranges -18<=h<=17,-19<=k<=23,-27<=l<=27

Reflections collected 68959

Independent reflections 34268[R(int) = 0.0547]

Completeness to theta =30.414° 84.1%

Absorption correction Multi-scan

Max. and min. transmission 1.00 and 0.83

Refinement method Full-matrix least-squares on F^2

Data / restraints / parameters 34268/ 540/ 2290

Goodness-of-fit on F^2 1.002

Final R indices [I>2sigma(I)] R1 = 0.0417, wR2 = 0.0944

R indices (all data) R1 = 0.0522, wR2 = 0.0969

Flack parameter x =-0.016(2)

Largest diff. peak and hole 1.668 and -3.107 e.$Å^{-3}$

Complex Au11(CCDC-2266408)

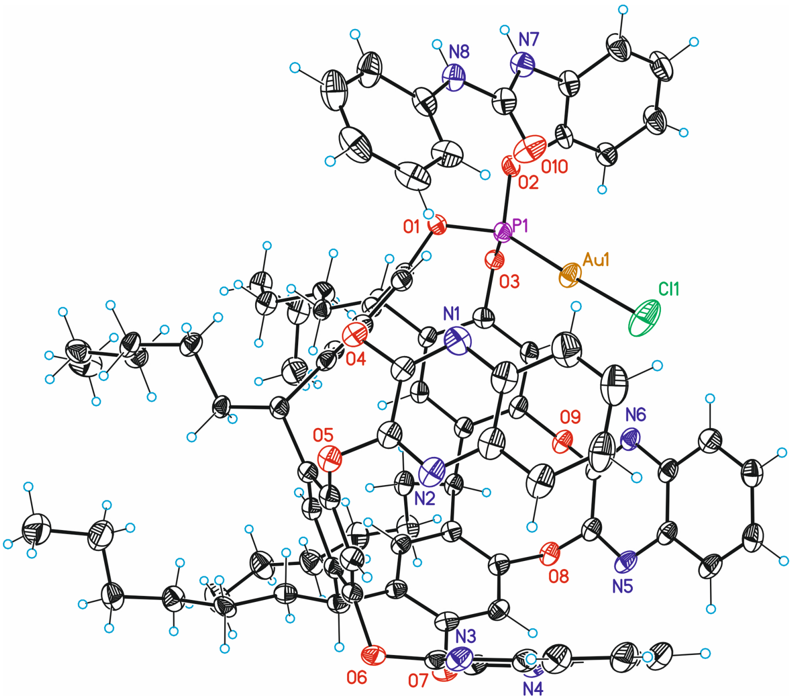

Identification code GO312LT_C2c

Empirical formula C96.70 H97.55 Au Cl N11.85 O10 P

Formula weight 1849.10

Temperature 100(2)K

Wavelength 0.71073 Å

Crystal system monoclinic

Space group C 2/c

Unit cell dimensions a = 35.9742(6)Å a= 90°.

b = 13.3931(2)Å b = 114.898(2)°.

c = 40.3962(7)Å g = 90°.

Volume 17654.2(6) Å^3

Z 8

Density (calculated) 1.391 Mg/m^3

Absorption coefficient 1.782 mm^{-1}

F(000) 7614

Crystal size 0.500 x 0.500 x 0.400 mm^3

Theta range for data collection 3.352 to 32.452°.

Index ranges -53<=h<=52,-20<=k<=19,-59<=l<=48

Reflections collected 149847

Independent reflections 29372[R(int) = 0.0782]

Completeness to theta =32.452° 92.3%

Absorption correction Multi-scan

Max. and min. transmission 1.00 and 0.44

Refinement method Full-matrix least-squares on F^2

Data / restraints / parameters 29372/ 833/ 1315

Goodness-of-fit on F^2 1.079

Final R indices [I>2sigma(I)] R1 = 0.0681, wR2 = 0.1612

R indices (all data) R1 = 0.0957, wR2 = 0.1727

Largest diff. peak and hole 6.063 and -2.493 e.Å^{-3}

Compound 2v (CCDC-2266406)

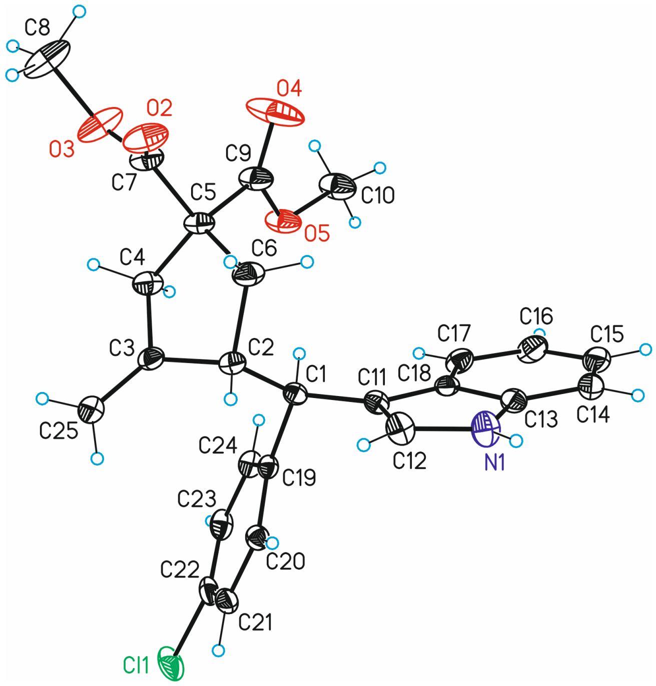

Identification code AMZ-04-364

Empirical formula C25 H24 Cl N O4

Formula weight 437.90

Temperature 100(2)K

Wavelength 0.71073 Å

Crystal system orthorhombic

Space group P 21 21 21

Unit cell dimensions a = 7.85740(13)Å a= 90°.

b = 12.4594(2)Å b = 90°.

c = 22.5242(4)Å g = 90°.

Volume2205.07(7) Å^3

Z 4

Density (calculated) 1.319 Mg/m^3

Absorption coefficient 0.205 mm^{-1}

F(000) 920

Crystal size 0.200 x 0.100 x 0.050 mm^3

Theta range for data collection 1.808 to 32.372°.

Index ranges -11<=h<=11,-18<=k<=18,-33<=l<=32

Reflections collected 49017

Independent reflections 7510[R(int) = 0.0301]

Completeness to theta =32.372° 96.9%

Absorption correction Multi-scan

Max. and min. transmission 1.00 and 0.78

Refinement method Full-matrix least-squares on F^2

Data / restraints / parameters 7510/ 0/ 285

Goodness-of-fit on F^2 1.060

Final R indices [I>2sigma(I)] R1 = 0.0314, wR2 = 0.0824

R indices (all data) R1 = 0.0344, wR2 = 0.0840

Flack parameter x =-0.008(10)

Largest diff. peak and hole 0.361 and -0.169 e.Å^{-3}

Compound 2ai (CCDC-2266405)

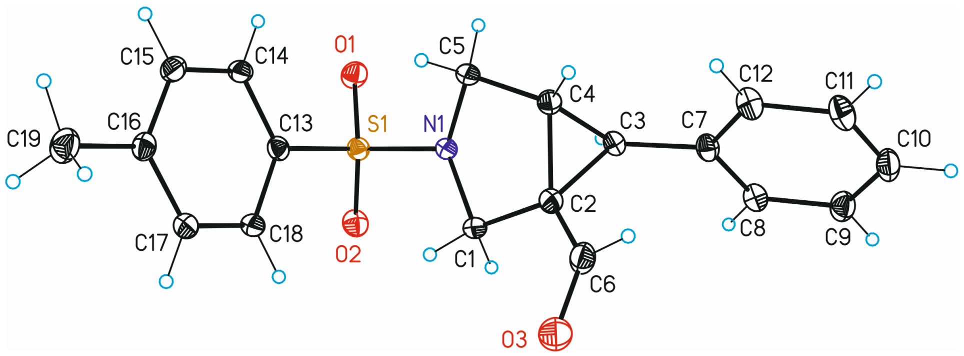

Identification code AMZ-04-453

Empirical formula C20 H21 Cl2 N O3 S

Formula weight 426.34

Temperature 100(2)K

Wavelength 0.71073 Å

Crystal system monoclinic

Space group P 21

Unit cell dimensions a = 12.7492(4)Å a= 90°.

b = 6.00710(10)Å b = 113.735(3)°.

c = 14.1369(4)Å g = 90°.

Volume991.11(5) Å^3

Z 2

Density (calculated) 1.429 Mg/m^3

Absorption coefficient 0.454 mm^{-1}

F(000) 444

Crystal size 0.400 x 0.200 x 0.200 mm^3

Theta range for data collection 2.781 to 32.139°.

Index ranges -18<=h<=18,-9<=k<=8,-20<=l<=20

Reflections collected 19275

Independent reflections 6340[R(int) = 0.0227]

Completeness to theta =32.139° 95.0%

Absorption correction Multi-scan

Max. and min. transmission 1.00 and 0.86

Refinement method Full-matrix least-squares on F^2

Data / restraints / parameters 6340/ 1/ 245

Goodness-of-fit on F^2 1.060

Final R indices [I>2sigma(I)] R1 = 0.0374, wR2 = 0.0927

R indices (all data) R1 = 0.0408, wR2 = 0.0966

Flack parameter x =0.009(11)

Largest diff. peak and hole 0.916 and -0.964 e.Å-3

7. NMR spectra

Compound **(*R*)-B1** ^{1}H, $^{31}P\{^{1}H\}$, and $^{13}C\{^{1}H\}$ NMR spectra in CD_2Cl_2

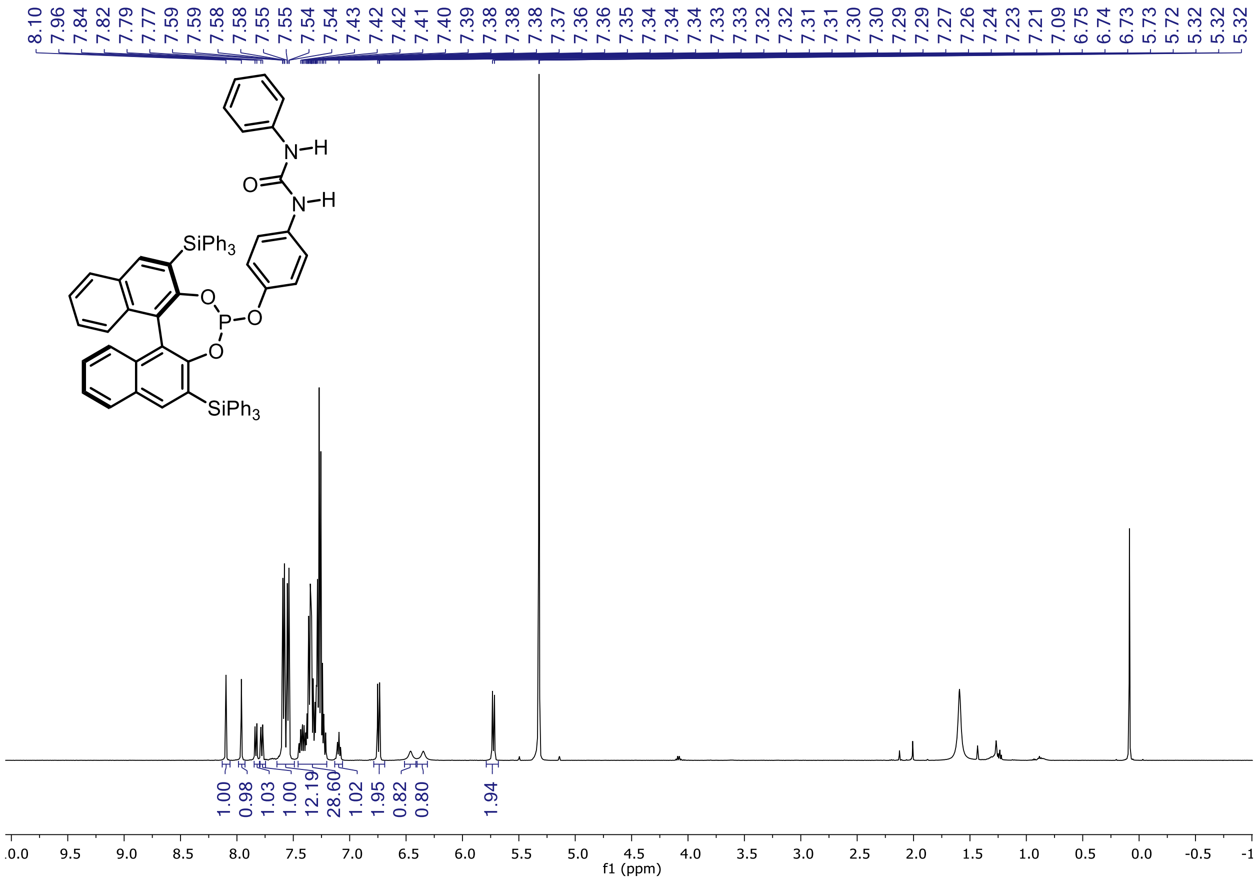

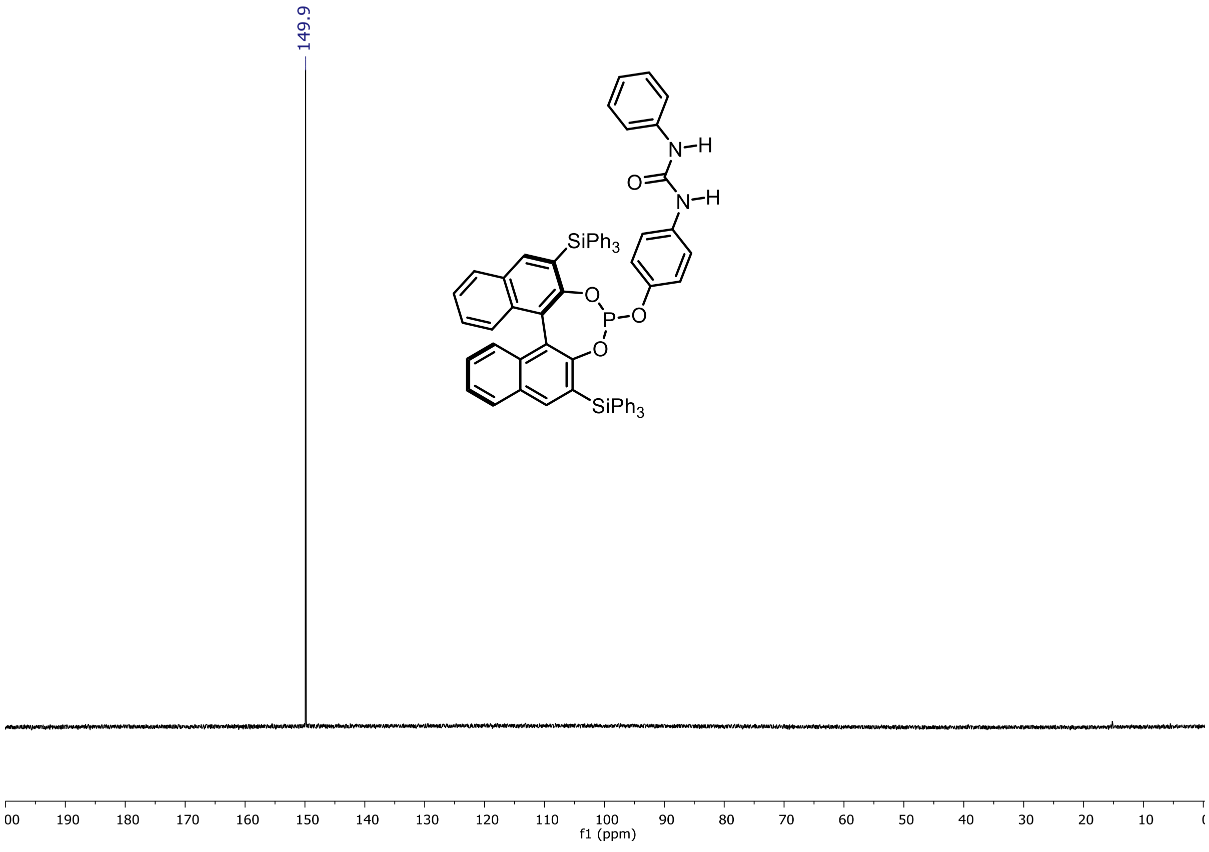

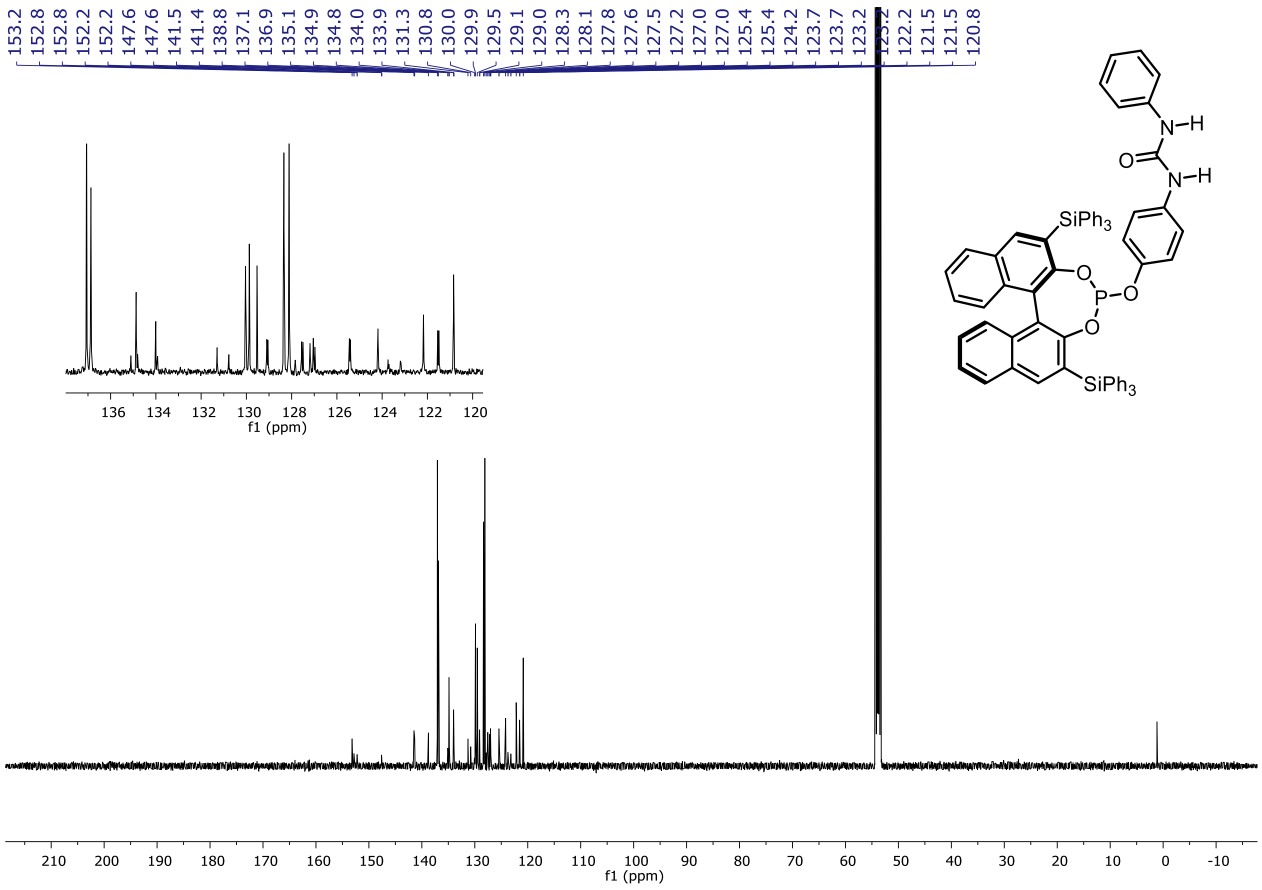
153.2
152.8
152.8
152.2
152.2
147.6
147.6
141.5
141.4
138.8
137.1
136.9
135.1
134.9
134.8
134.0
133.9
131.3
130.8
130.0
129.9
129.5
129.1
129.0
128.3
128.1
127.8
127.6
127.5
127.2
127.0
127.0
125.4
124.2
123.7
123.7
123.2
123.2
122.2
121.5
121.5
120.8
136
134
132
130
128
126
124
122
120
f1 (ppm)
210
200
190
180
170
160
150
140
130
120
110
100
90
80
70
60
50
40
30
20
10
0
-10
f1 (ppm)
N–H
O
N–H
SiPh3
O
P–O
O
SiPh3

Compound **(*R*)-B2** ^{1}H, ^{31}P{^{1}H}, and ^{13}C{^{1}H} NMR spectra in CD_2Cl_2

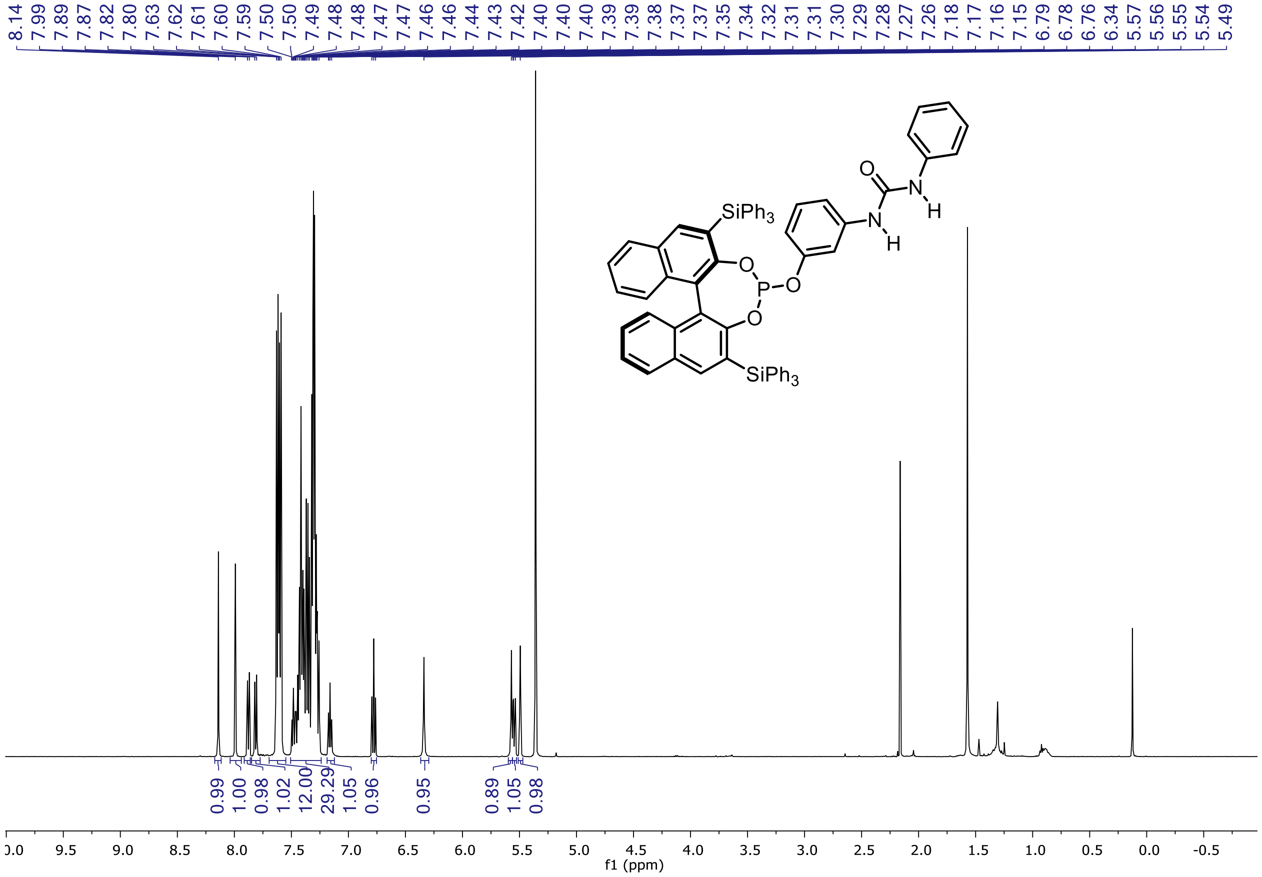

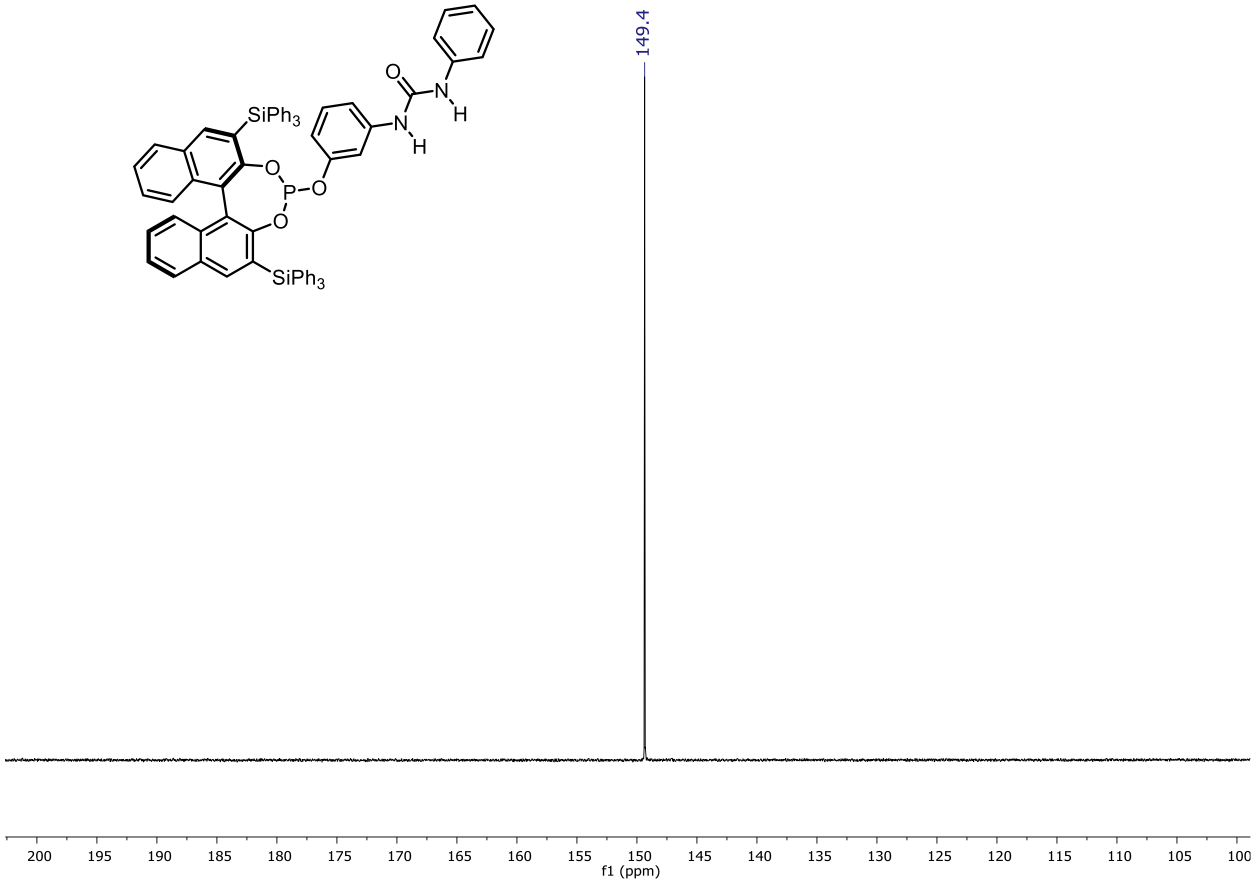

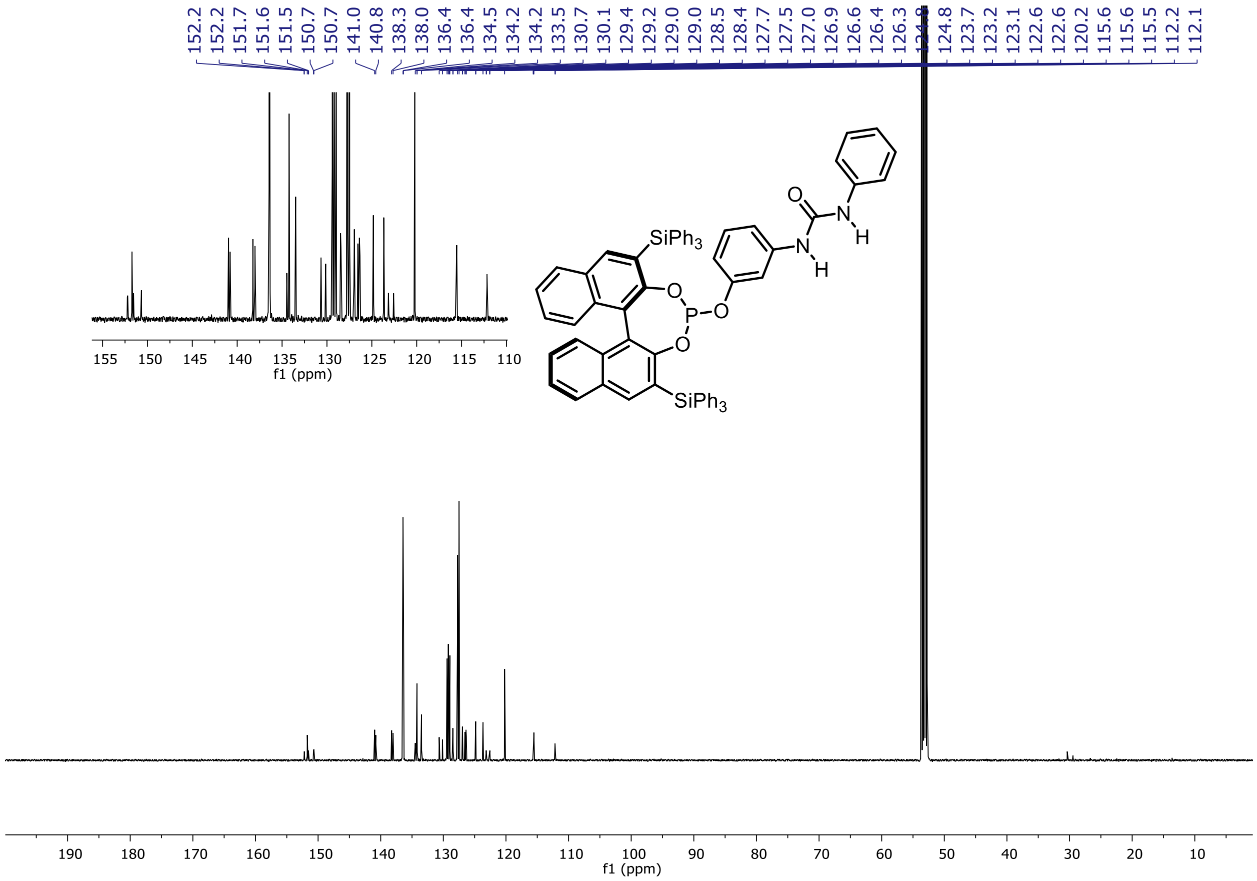
152.2
152.2
151.7
151.6
151.5
150.7
150.7
141.0
140.8
138.3
138.0
136.4
136.4
134.5
134.2
134.2
133.5
130.7
130.1
129.4
129.2
129.0
129.0
128.5
128.4
127.7
127.5
127.0
126.9
126.6
126.4
126.3
124.8
123.7
123.2
123.1
122.6
122.6
120.2
115.6
115.5
112.2
112.1
SiPh3
SiPh3
O
P
O
N
H
f1 (ppm)

Compound **(*R*)-B3** ^{1}H, $^{31}P\{^{1}H\}$, and $^{13}C\{^{1}H\}$ NMR spectra in CD_2Cl_2

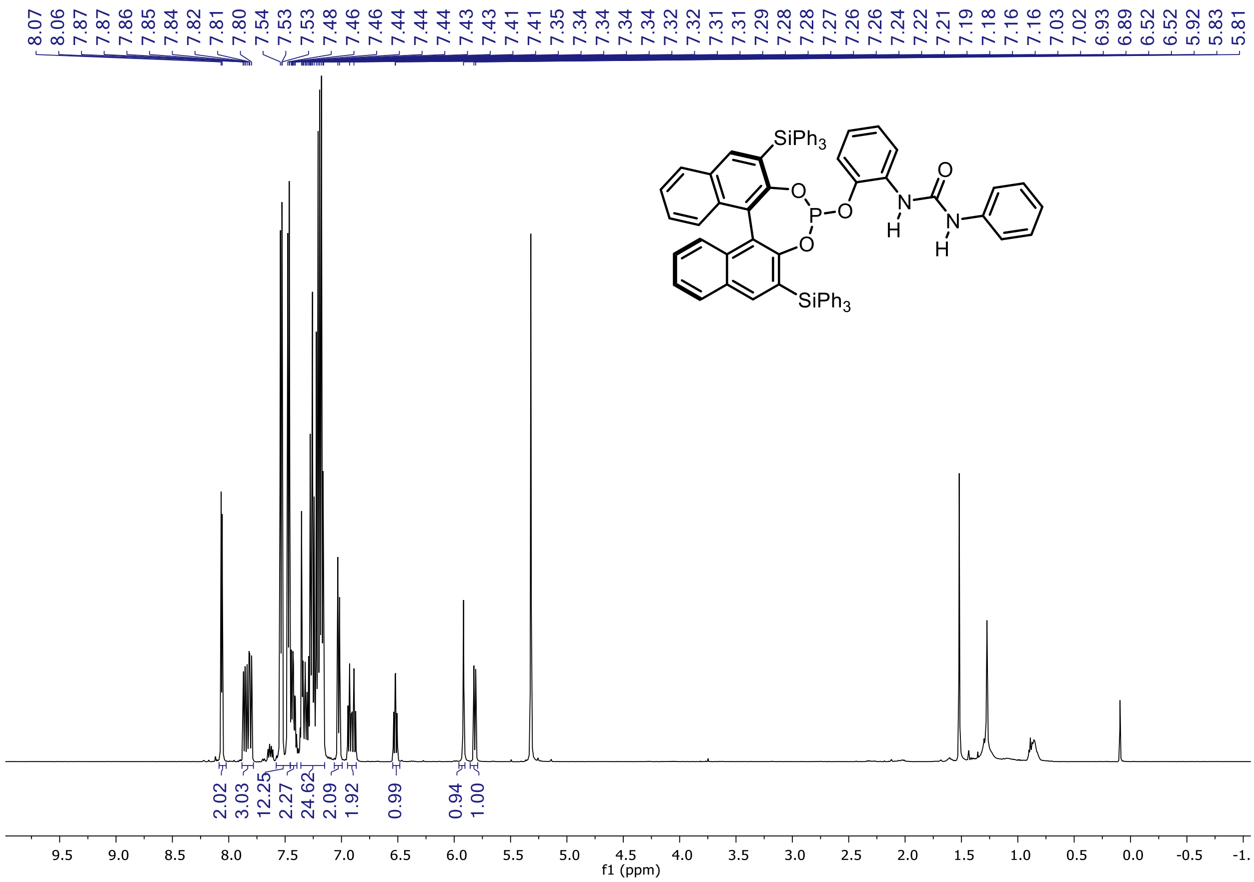

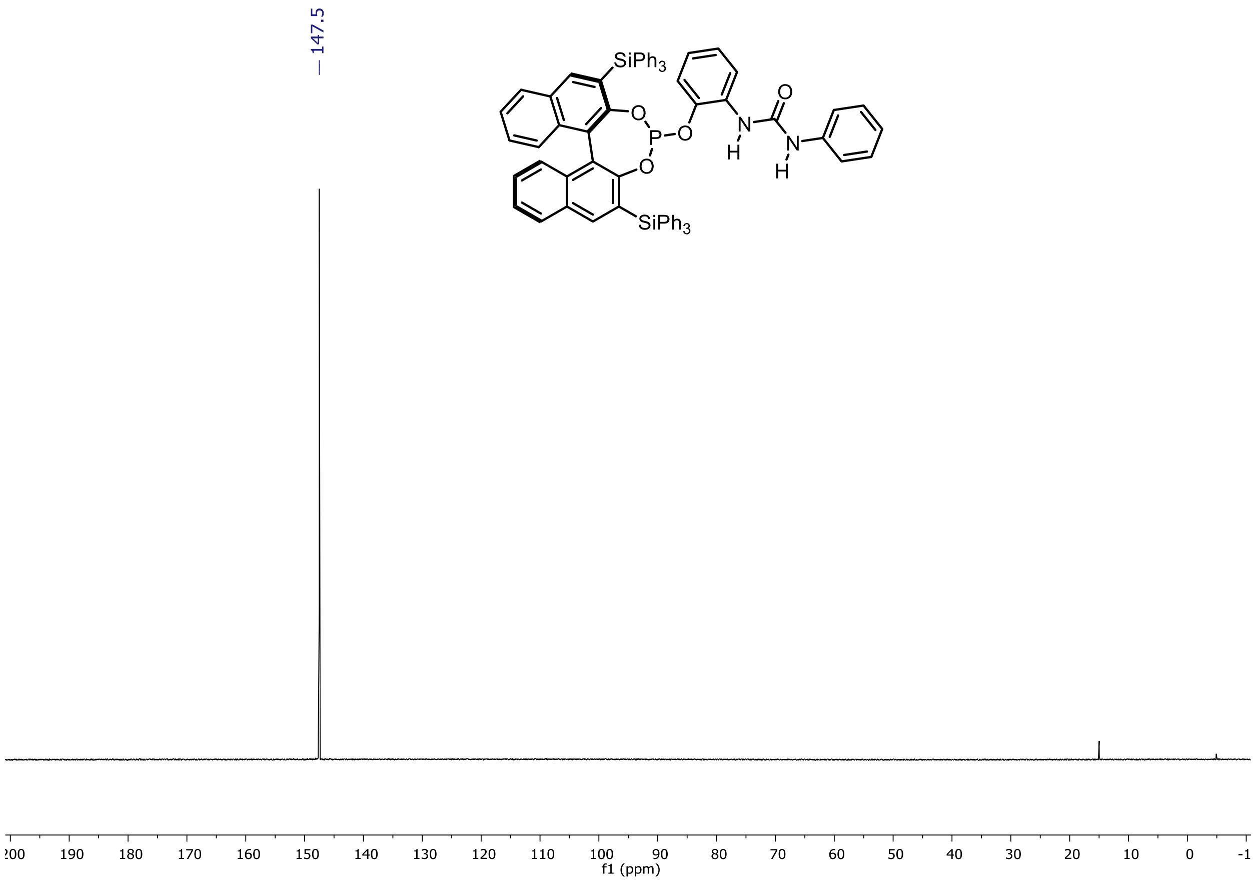

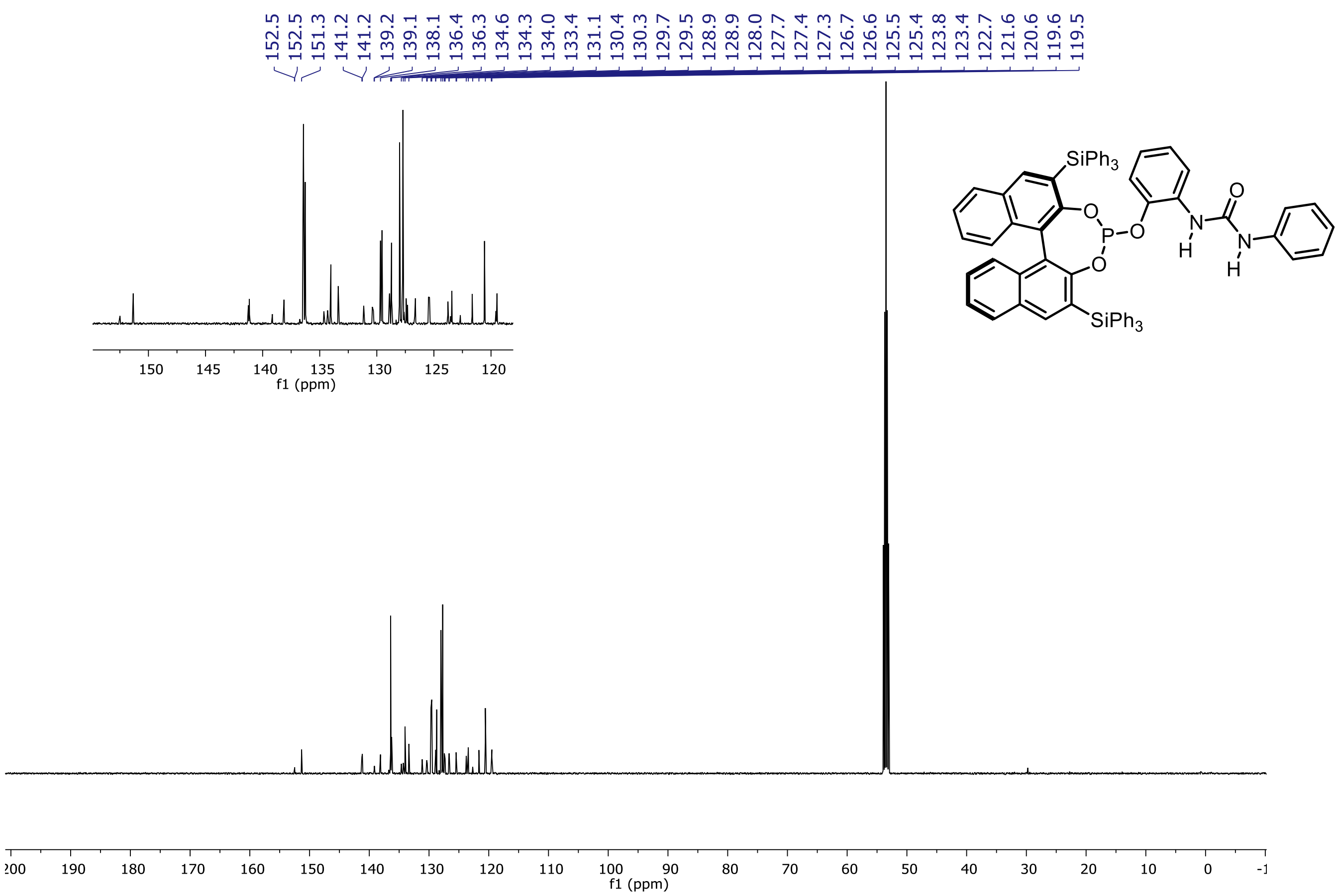
152.5
152.5
151.3
141.2
141.2
139.2
139.1
138.1
136.4
136.3
134.6
134.3
134.0
133.4
131.1
130.4
130.3
129.7
129.5
128.9
128.9
128.0
127.7
127.4
127.3
126.7
126.6
125.5
125.4
123.8
123.4
122.7
121.6
120.6
119.6
119.5
f1 (ppm)
SiPh3
O
P
N
H

Compound **(*R*)-B4** ^{1}H, ^{31}P{^{1}H}, and ^{13}C{^{1}H} NMR spectra in CD_2Cl_2

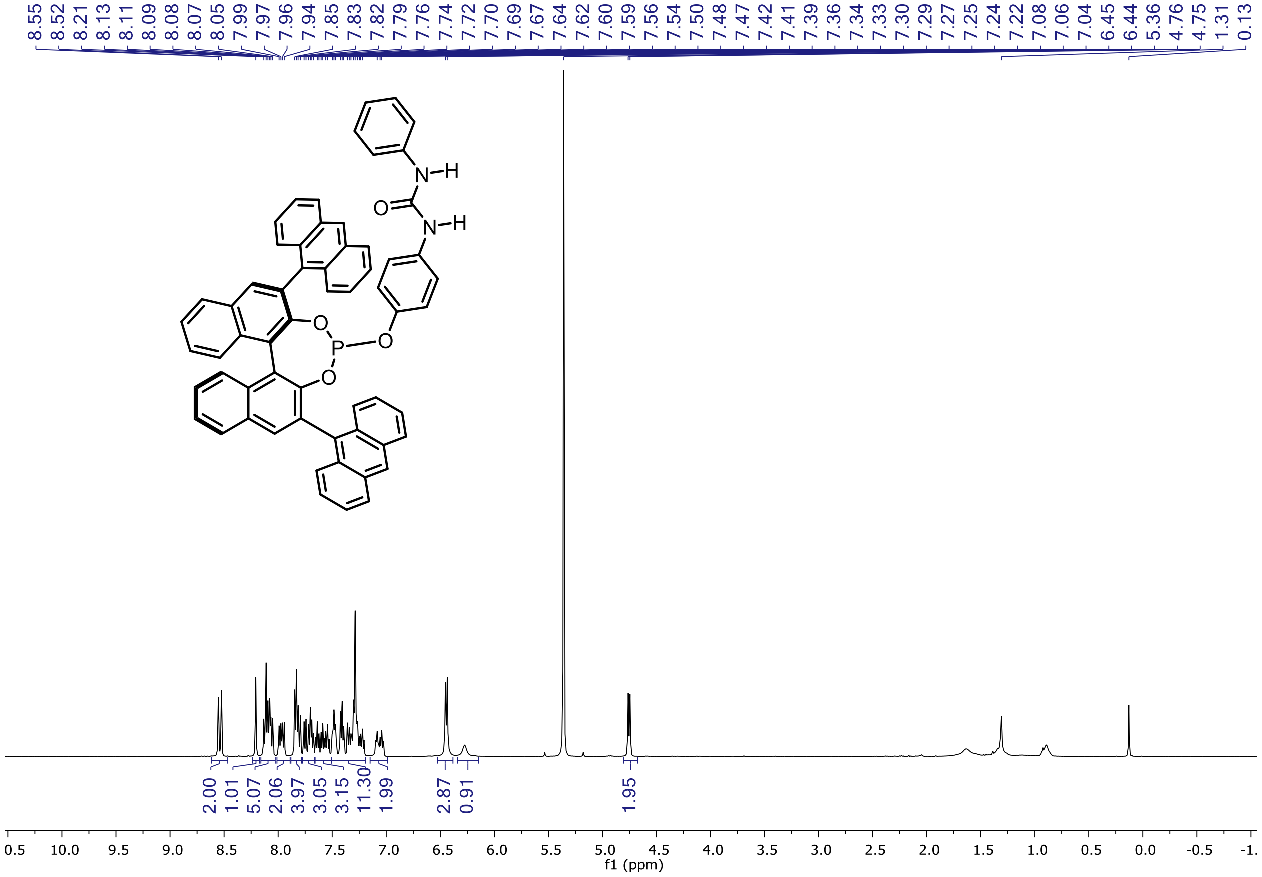

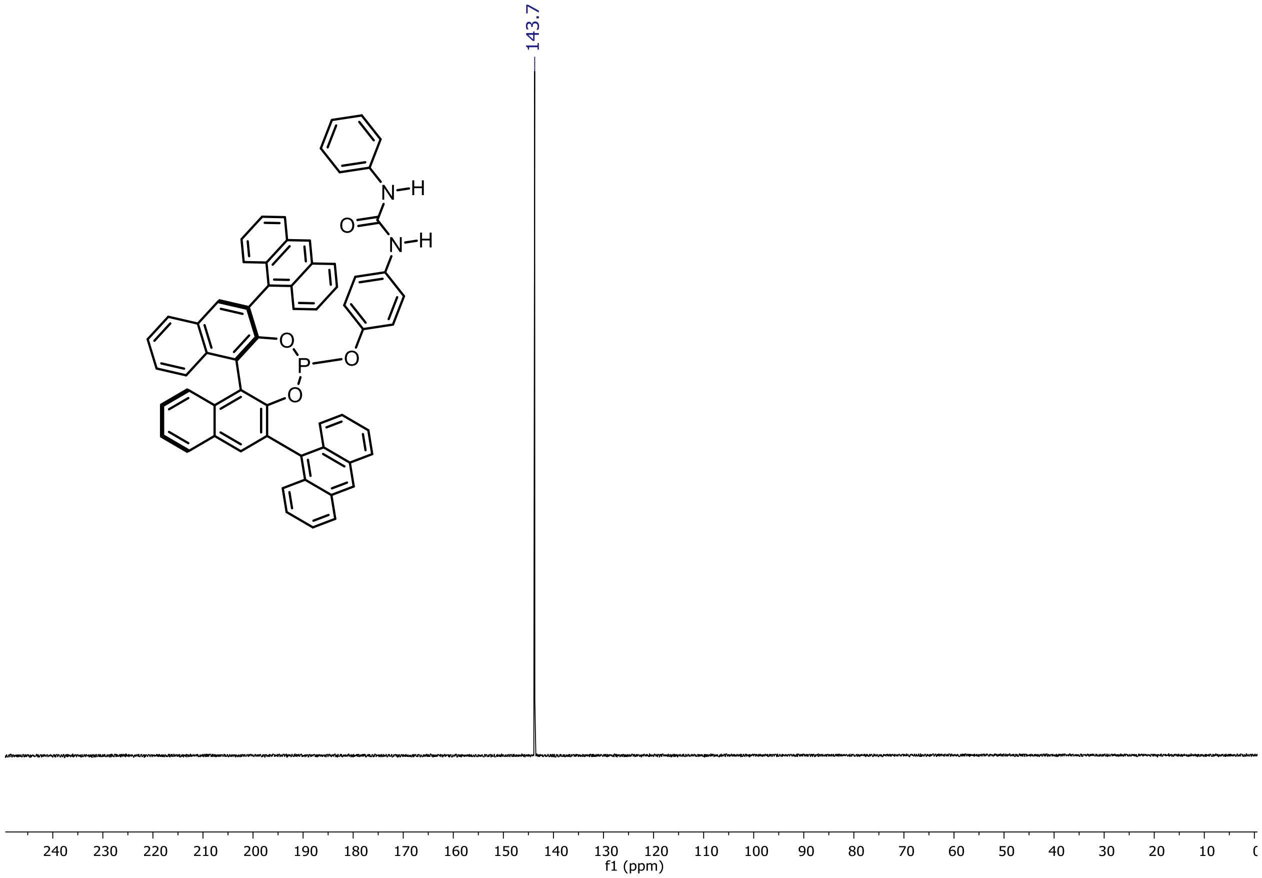

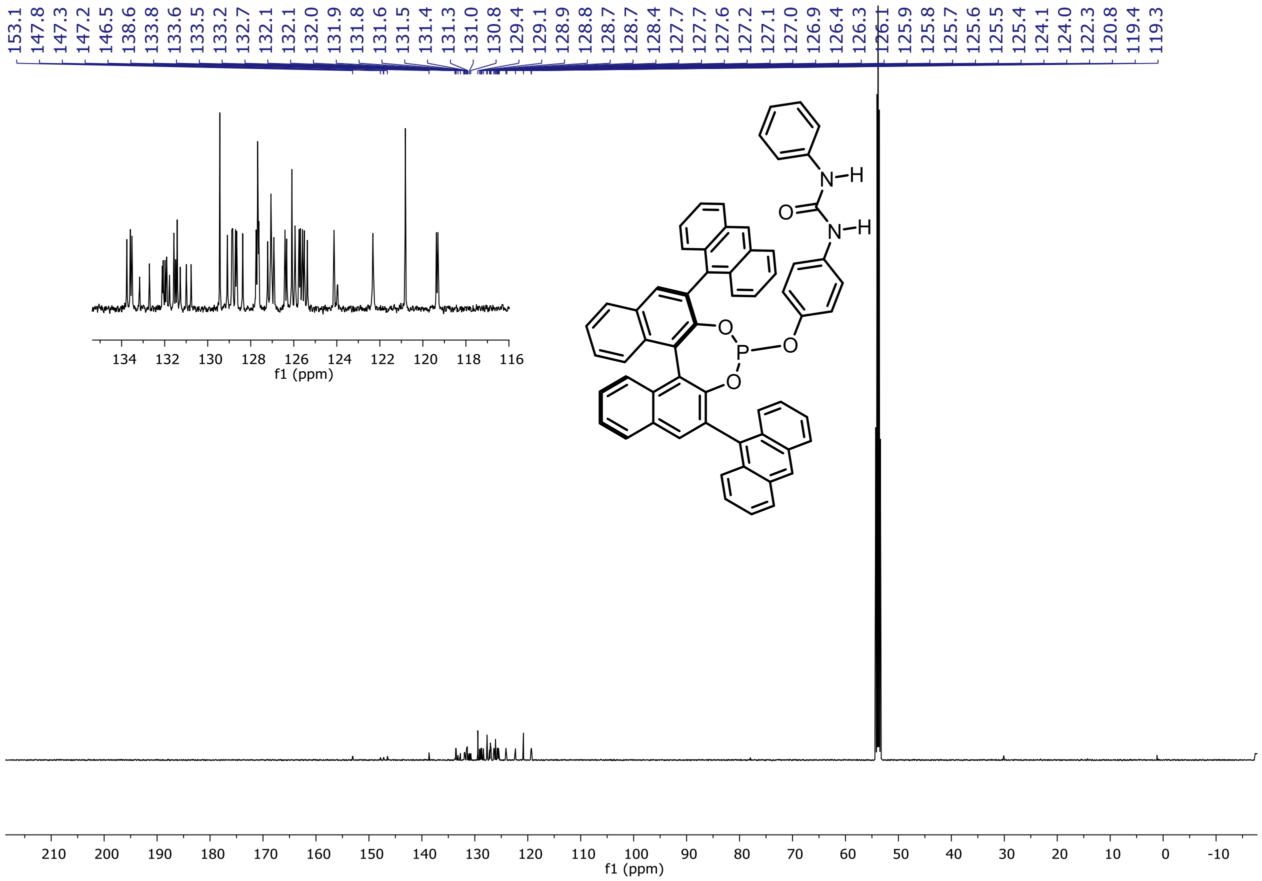
153.1
147.8
147.3
147.2
146.5
138.6
133.8
133.6
133.5
133.2
132.7
132.1
132.1
132.0
131.9
131.8
131.6
131.5
131.4
131.3
131.0
130.8
129.4
129.1
128.9
128.8
128.7
128.4
127.7
127.7
127.6
127.2
127.1
127.0
126.9
126.4
126.3
126.1
125.9
125.8
125.7
125.6
125.5
125.4
124.1
124.0
122.3
120.8
119.4
119.3
f1 (ppm)
f1 (ppm)

Compound **(*R*)-B5** ^{1}H, $^{31}P\{^{1}H\}$, and $^{13}C\{^{1}H\}$ NMR spectra in $(CD_3)_2CO$

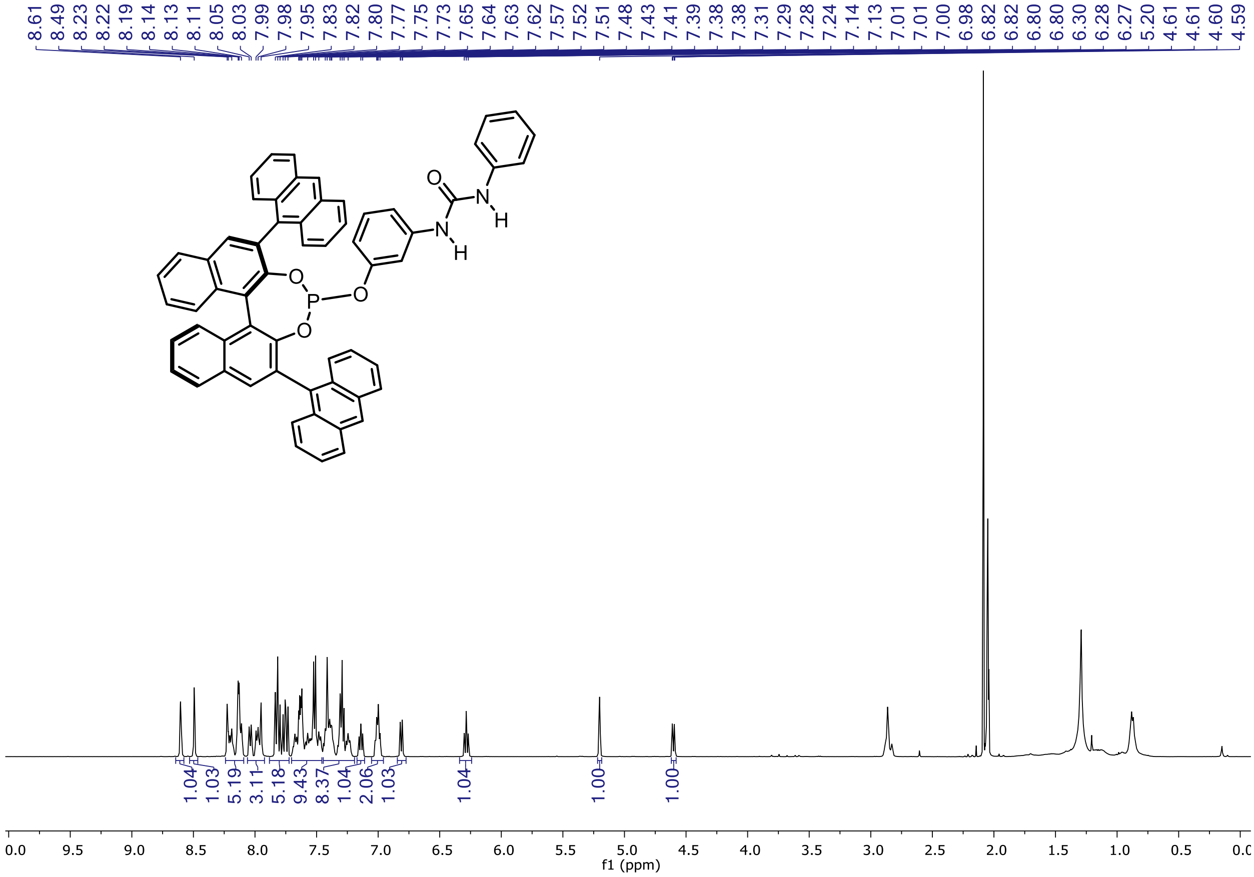

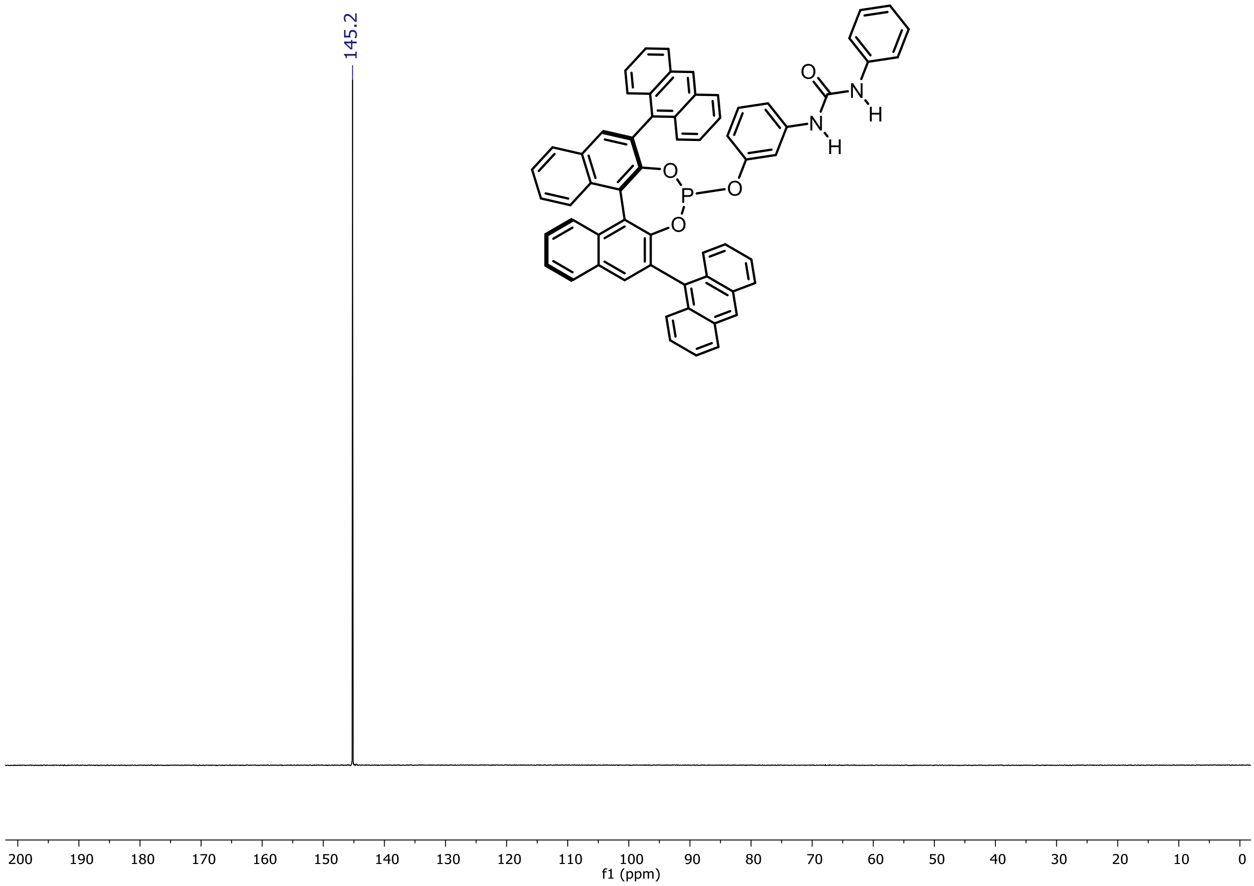

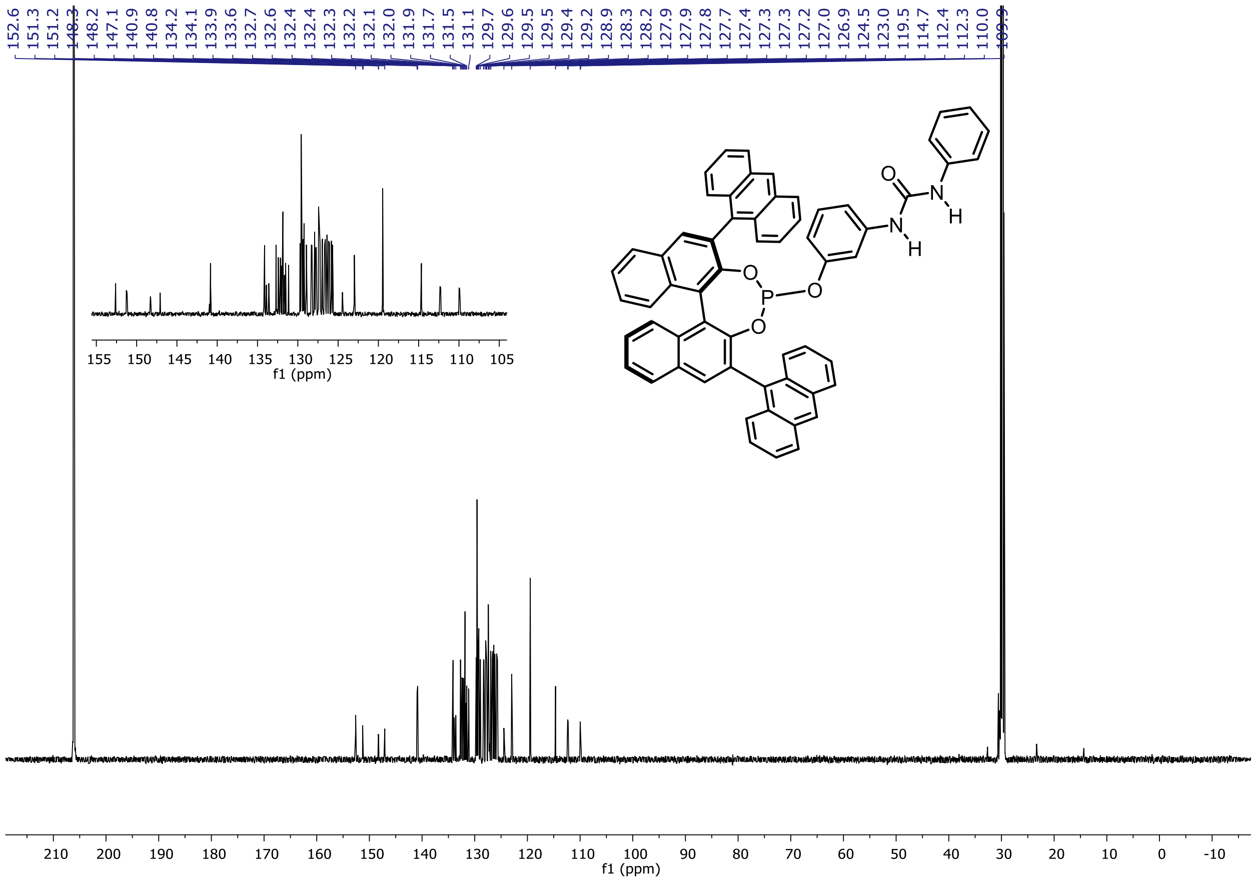
152.6
151.3
151.2
148.3
148.2
147.1
140.9
140.8
134.2
134.1
133.9
133.6
132.7
132.6
132.4
132.4
132.3
132.2
132.1
132.0
131.9
131.7
131.5
131.1
129.7
129.6
129.5
129.5
129.4
129.2
128.9
128.3
128.2
127.9
127.9
127.8
127.7
127.4
127.3
127.3
127.2
127.0
126.9
124.5
123.0
119.5
114.7
112.4
112.3
110.0
155 150 145 140 135 130 125 120 115 110 105
f1 (ppm)
210 200 190 180 170 160 150 140 130 120 110 100 90 80 70 60 50 40 30 20 10 0 -10
f1 (ppm)
O
N
H
N
H
O
P
O
O

Compound **(*R*)-B6** ^{1}H, $^{31}P\{^{1}H\}$, and $^{13}C\{^{1}H\}$ NMR spectra in $(CD_3)_2CO$

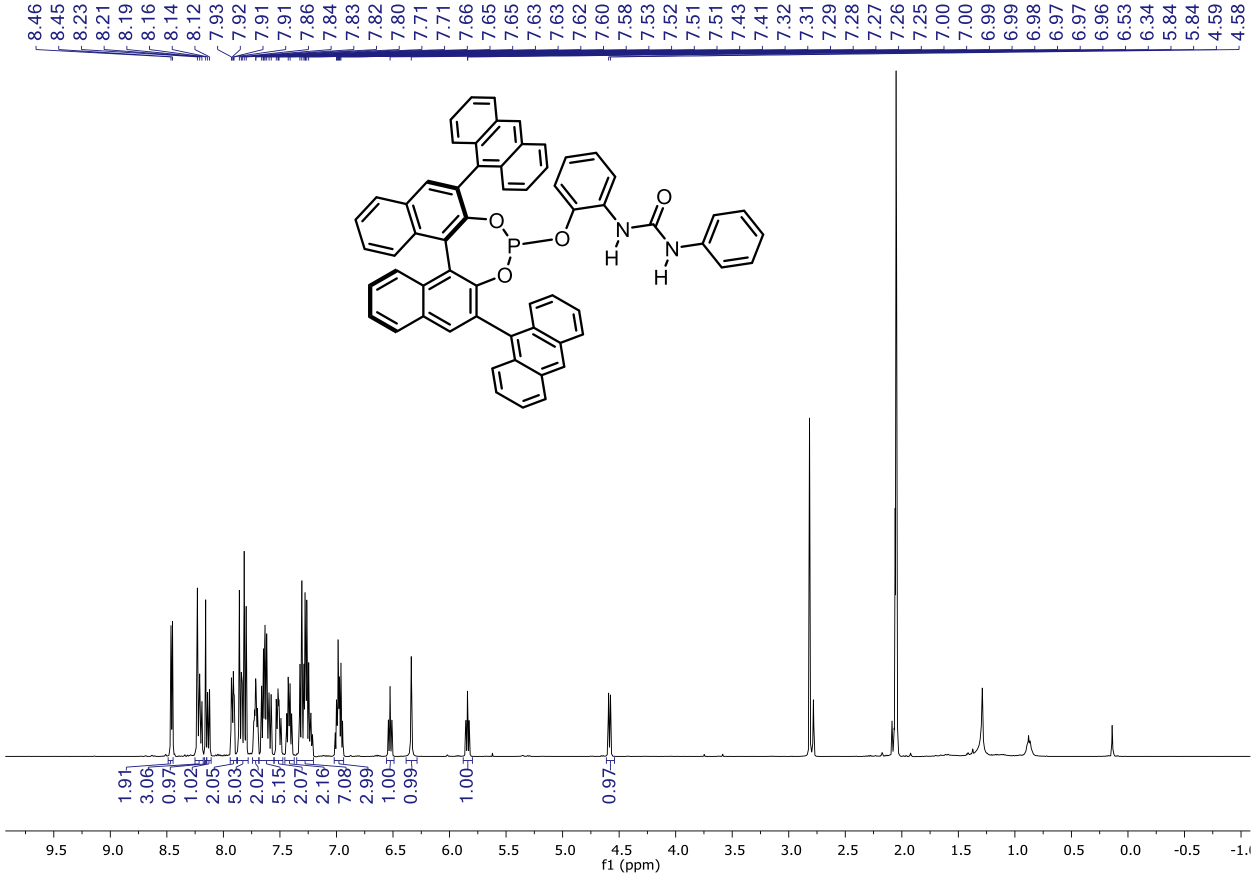

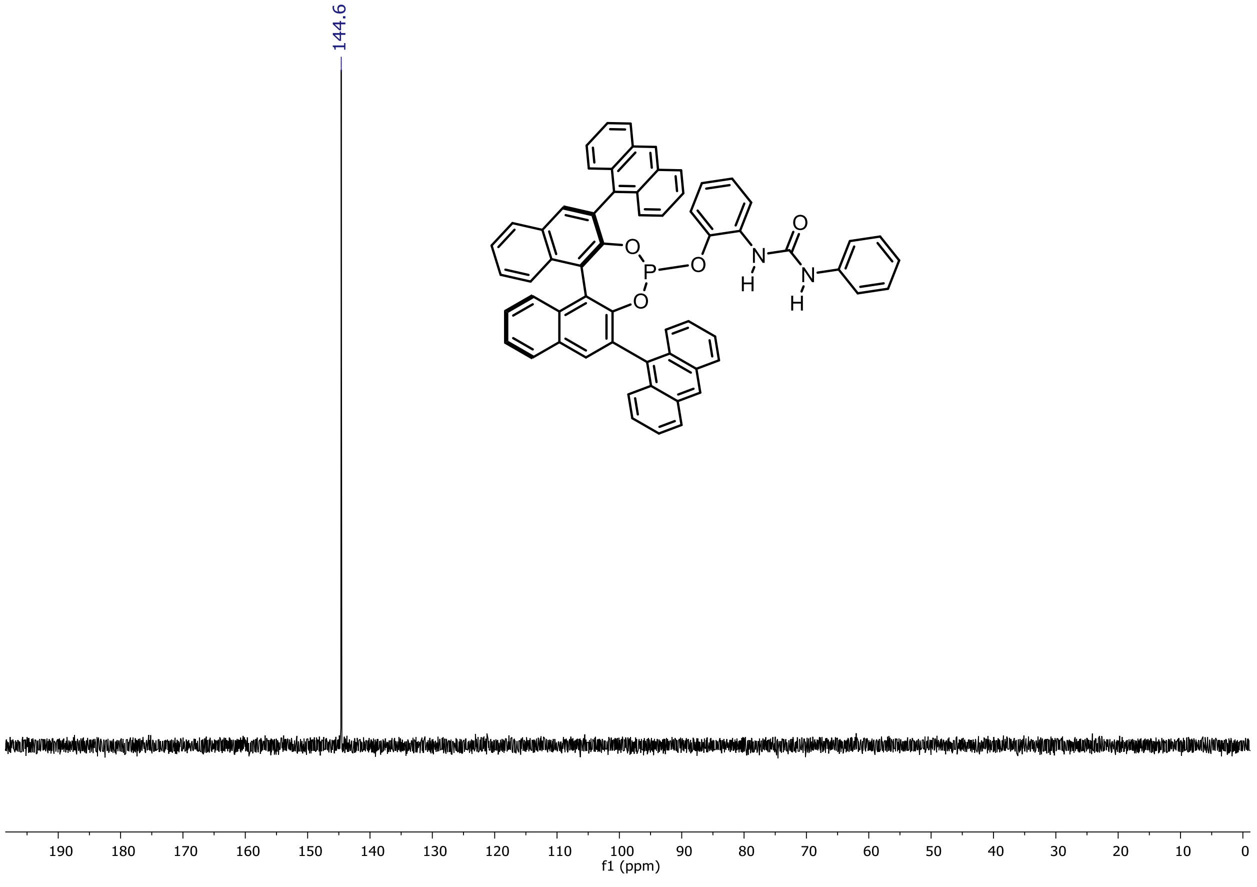

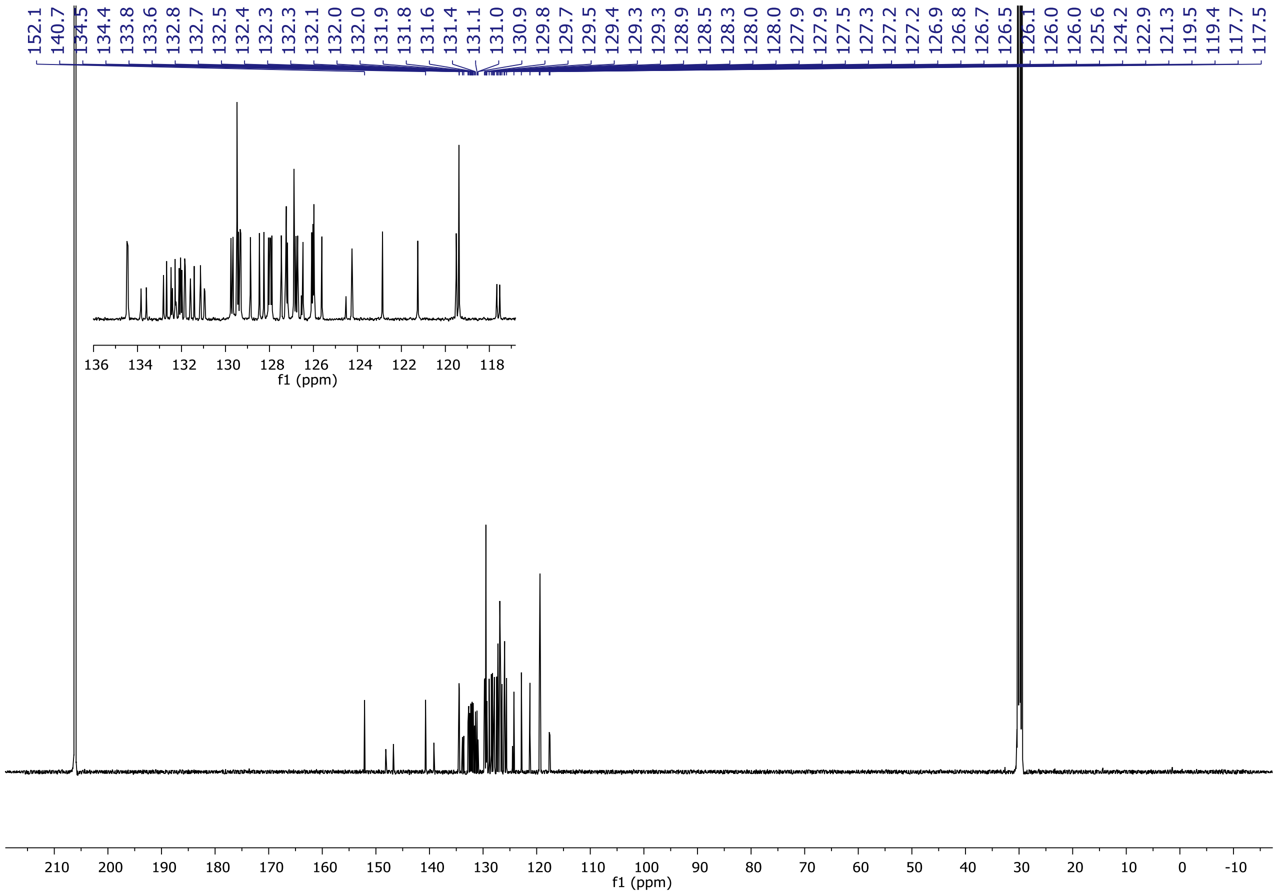
152.1
140.7
134.5
134.4
133.8
133.6
132.8
132.7
132.5
132.4
132.3
132.3
132.1
132.0
132.0
131.9
131.8
131.6
131.4
131.1
131.0
130.9
129.8
129.7
129.5
129.4
129.3
129.3
128.9
128.5
128.3
128.0
128.0
127.9
127.9
127.5
127.3
127.2
127.2
126.9
126.8
126.7
126.5
126.1
126.0
126.0
125.6
124.2
122.9
121.3
119.5
119.4
117.7
117.5
f1 (ppm)
f1 (ppm)

Compound (***R***)-**B7** ^{1}H, $^{31}P\{^{1}H\}$, $^{19}F\{^{1}H\}$, and $^{13}C\{^{1}H\}$ NMR spectra in CD_2Cl_2

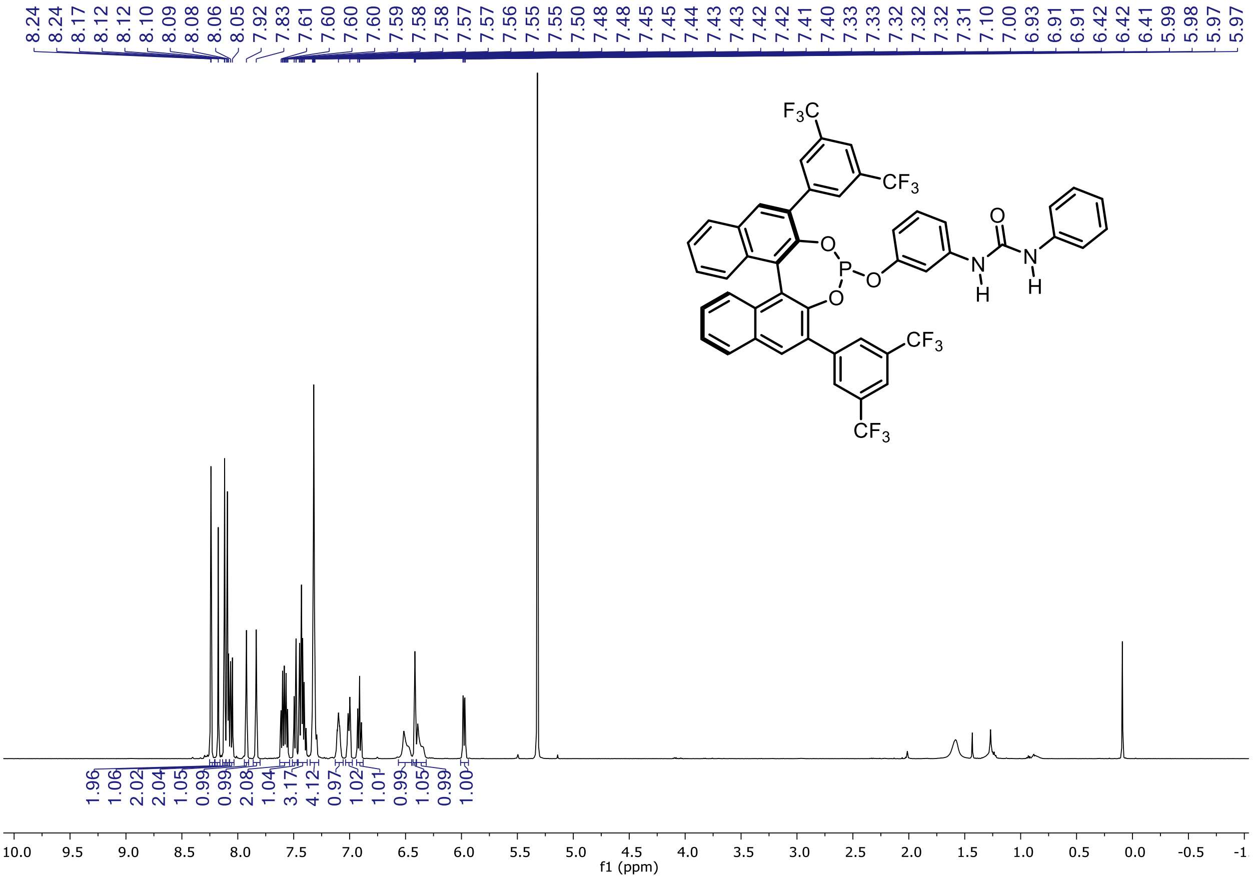

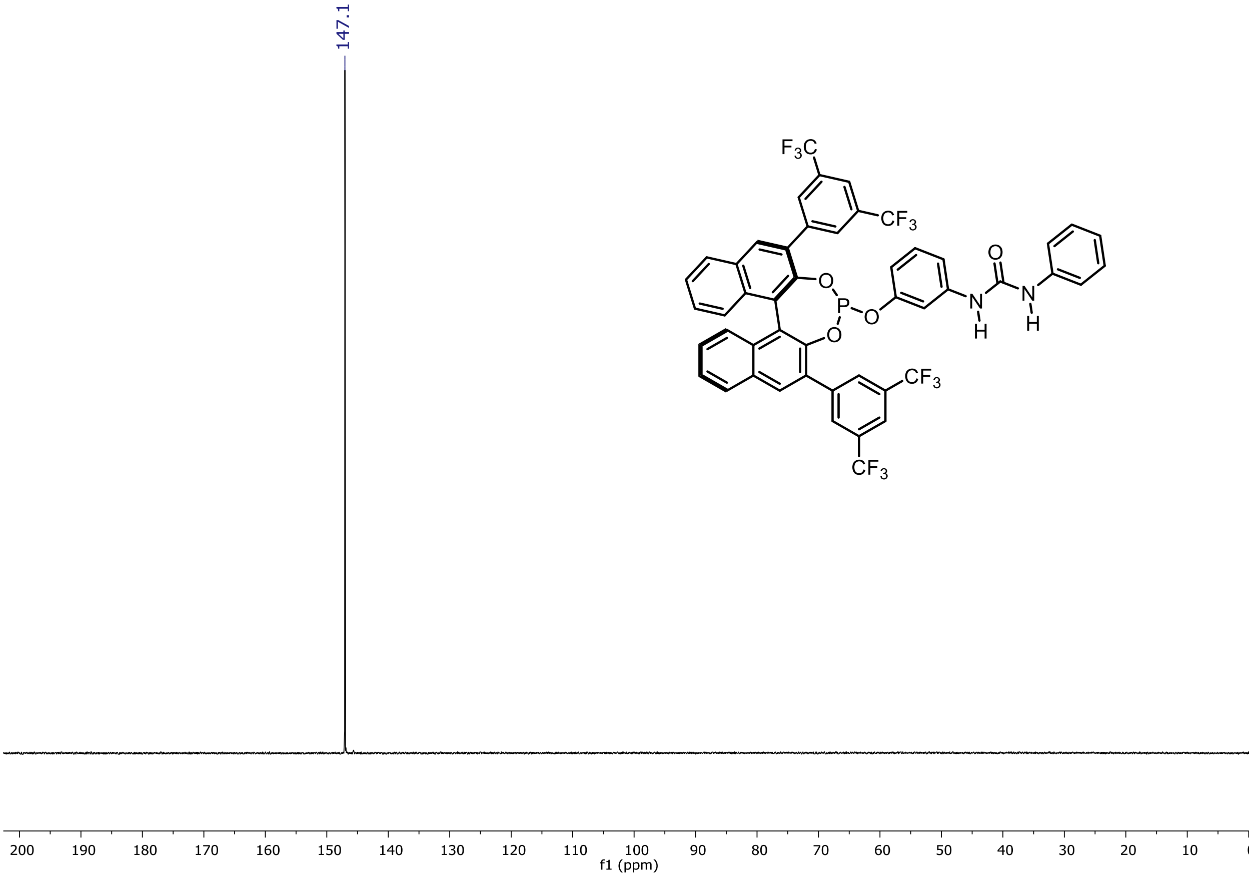

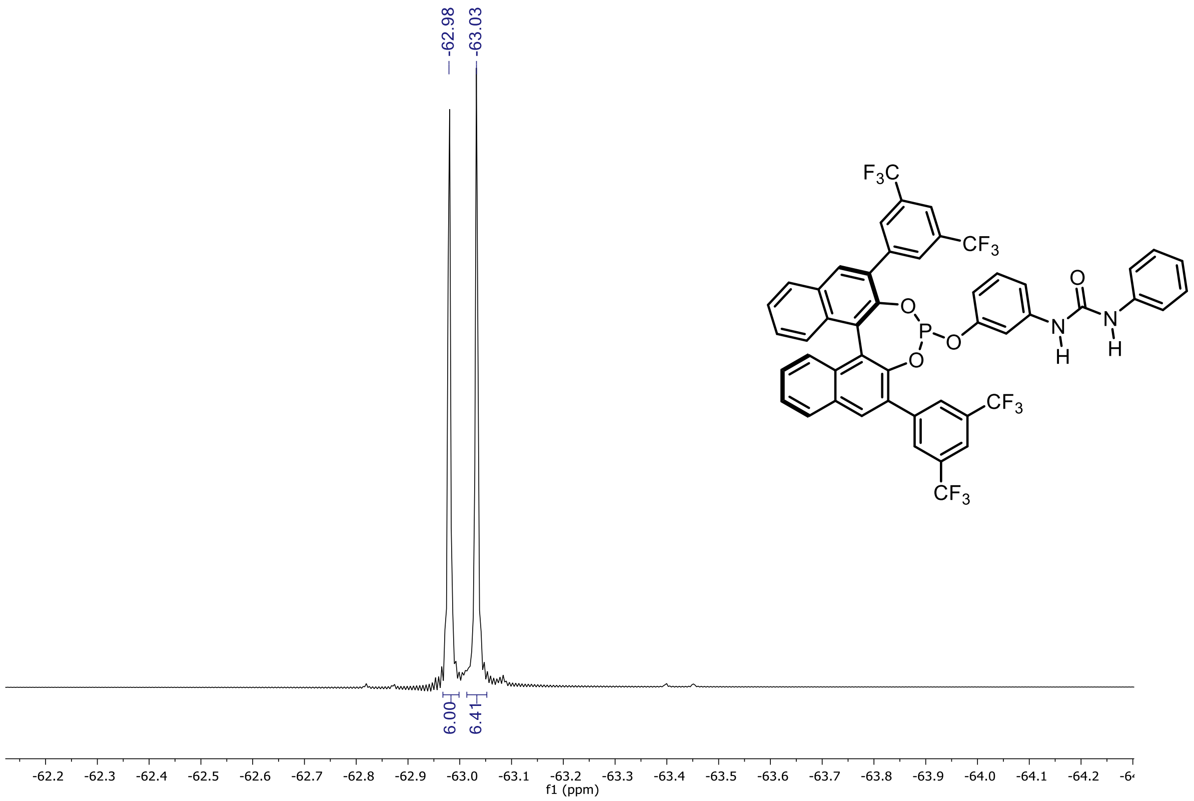

-62.98
-63.03
6.00
6.41
-62.2 -62.3 -62.4 -62.5 -62.6 -62.7 -62.8 -62.9 -63.0 -63.1 -63.2 -63.3 -63.4 -63.5 -63.6 -63.7 -63.8 -63.9 -64.0 -64.1 -64.2
f1 (ppm)
F3C
CF3
O
N
H
P
CF3
CF3

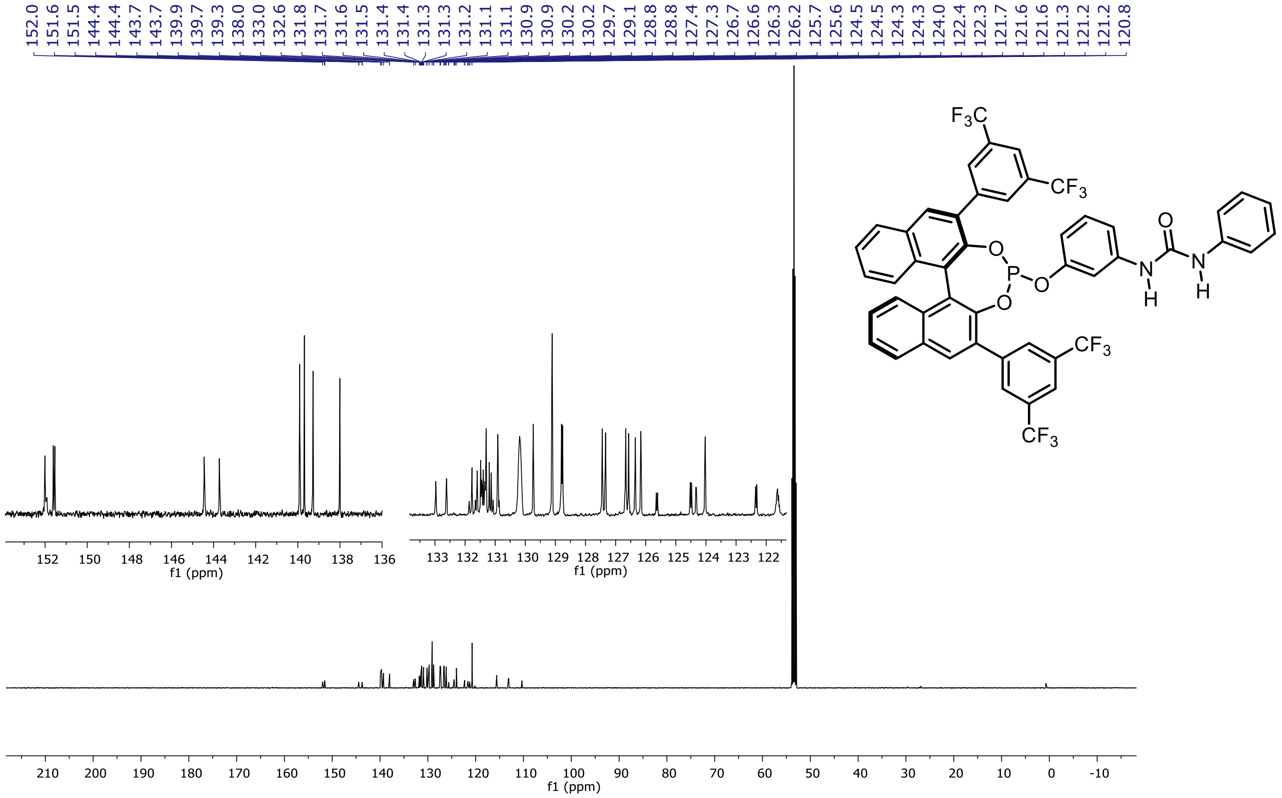

152.0 151.6 151.5 144.4 144.4 143.7 143.7 139.9 139.7 139.3 138.0 133.0 132.6 131.8 131.7 131.6 131.5 131.4 131.4 131.3 131.3 131.2 131.1 131.1 130.9 130.9 130.2 130.2 129.7 129.1 128.8 128.8 127.4 127.3 126.7 126.6 126.3 126.2 125.7 125.6 124.5 124.5 124.3 124.3 124.0 122.4 122.3 121.7 121.6 121.6 121.3 121.2 121.2 120.8
152 150 148 146 144 142 140 138 136
f1 (ppm)
133 132 131 130 129 128 127 126 125 124 123 122
f1 (ppm)
210 200 190 180 170 160 150 140 130 120 110 100 90 80 70 60 50 40 30 20 10 0 -10
f1 (ppm)
F3C
CF3
O
N
H
P
CF3
CF3

Compound **(*R*)-B8** ^{1}H, $^{31}P\{^{1}H\}$ and $^{13}C\{^{1}H\}$ NMR spectra in CD_2Cl_2

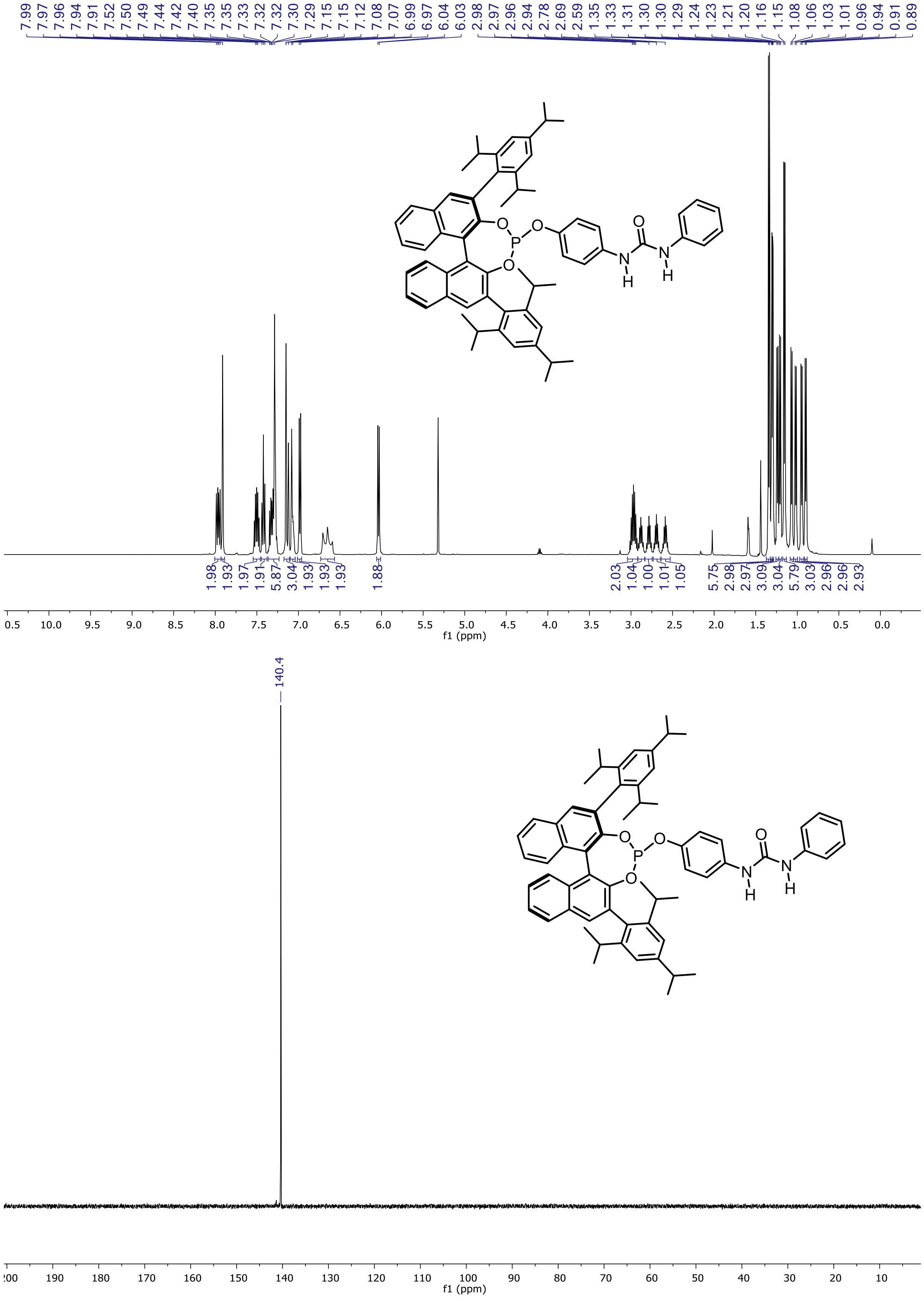

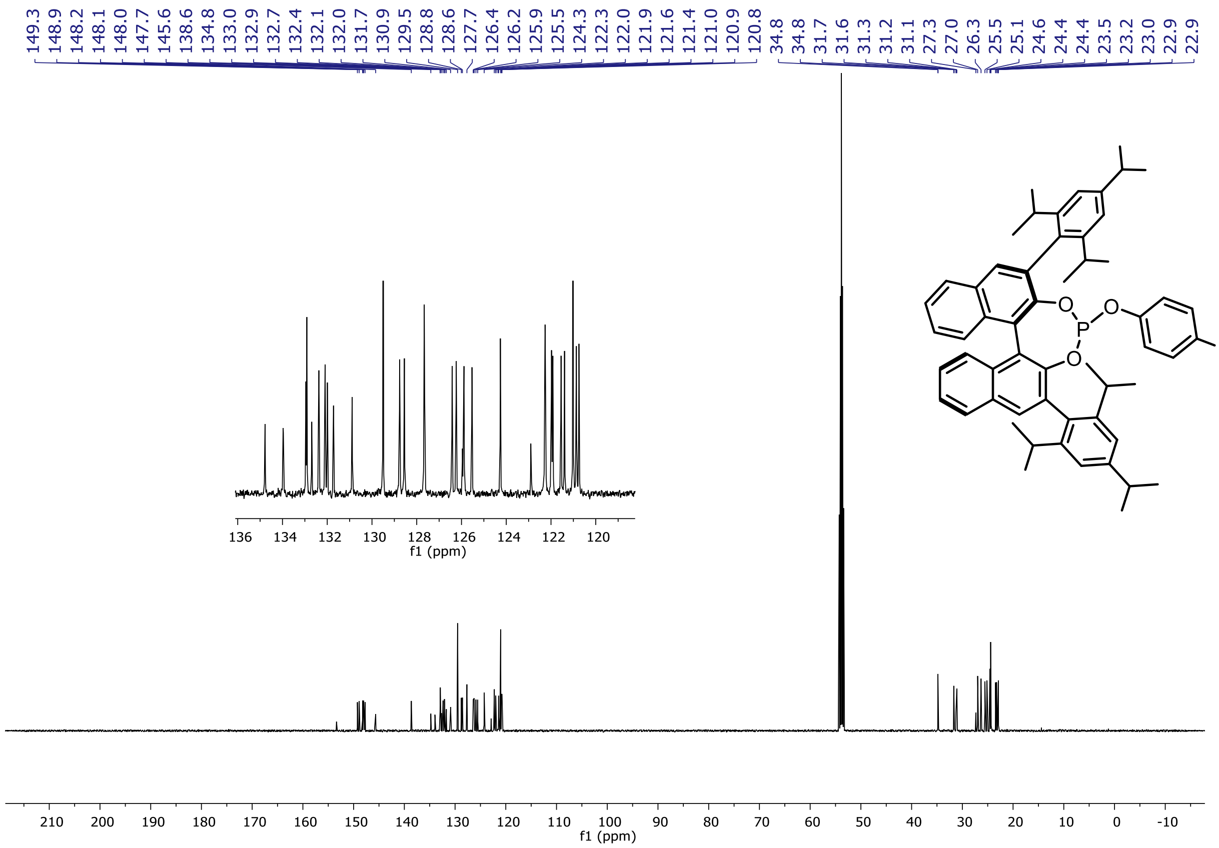
149.3
148.9
148.2
148.1
148.0
147.7
145.6
138.6
134.8
133.0
132.9
132.7
132.4
132.1
132.0
131.7
130.9
129.5
128.8
128.6
127.7
126.4
126.2
125.9
125.5
124.3
122.3
122.0
121.9
121.6
121.4
121.0
120.9
120.8
34.8
34.8
31.7
31.6
31.3
31.2
31.1
27.3
27.0
26.3
25.5
25.1
24.6
24.4
24.4
23.5
23.2
23.0
22.9
22.9
f1 (ppm)
f1 (ppm)
O
P
N
H

Compound **B9** ^{1}H, ^{31}P{^{1}H}, and ^{13}C{^{1}H} NMR spectra in $CDCl_3$

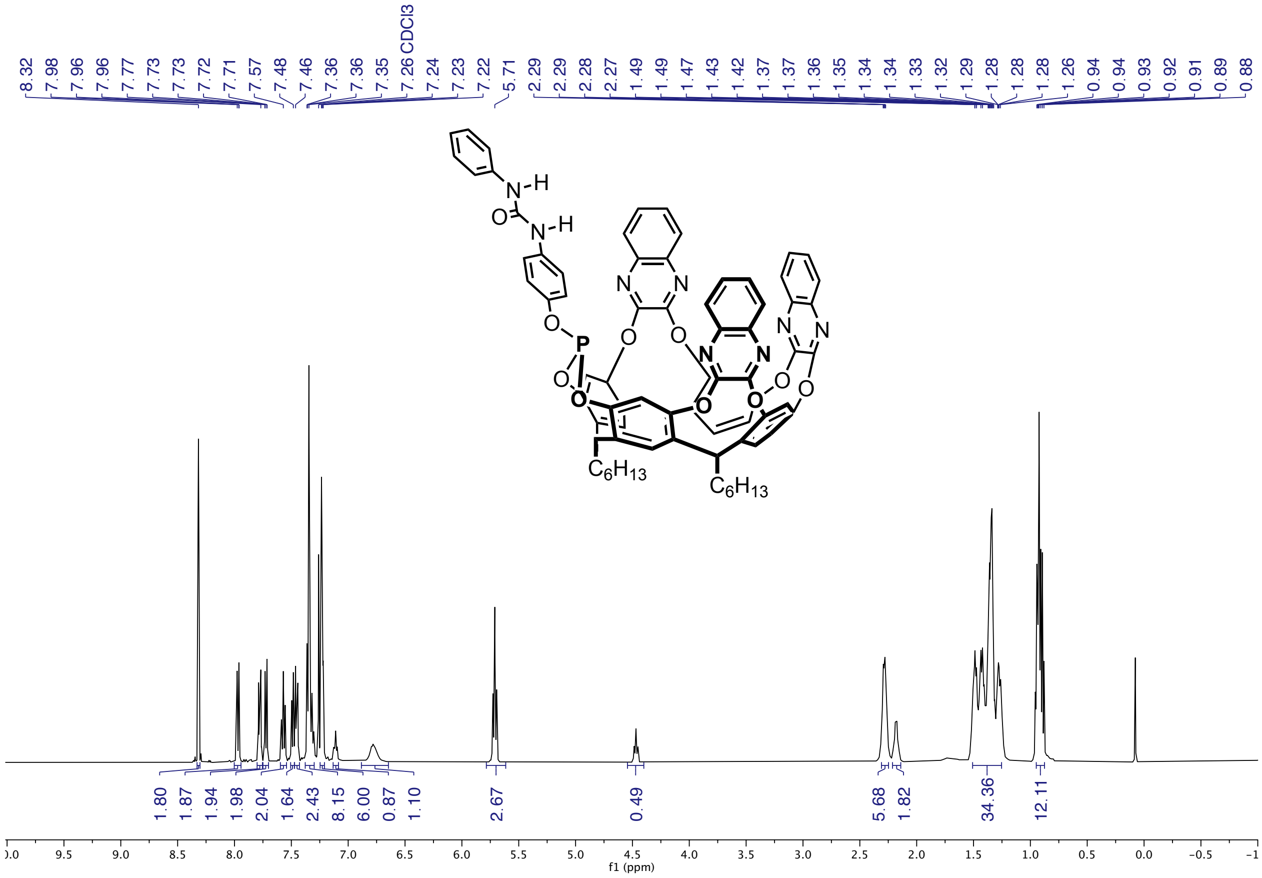

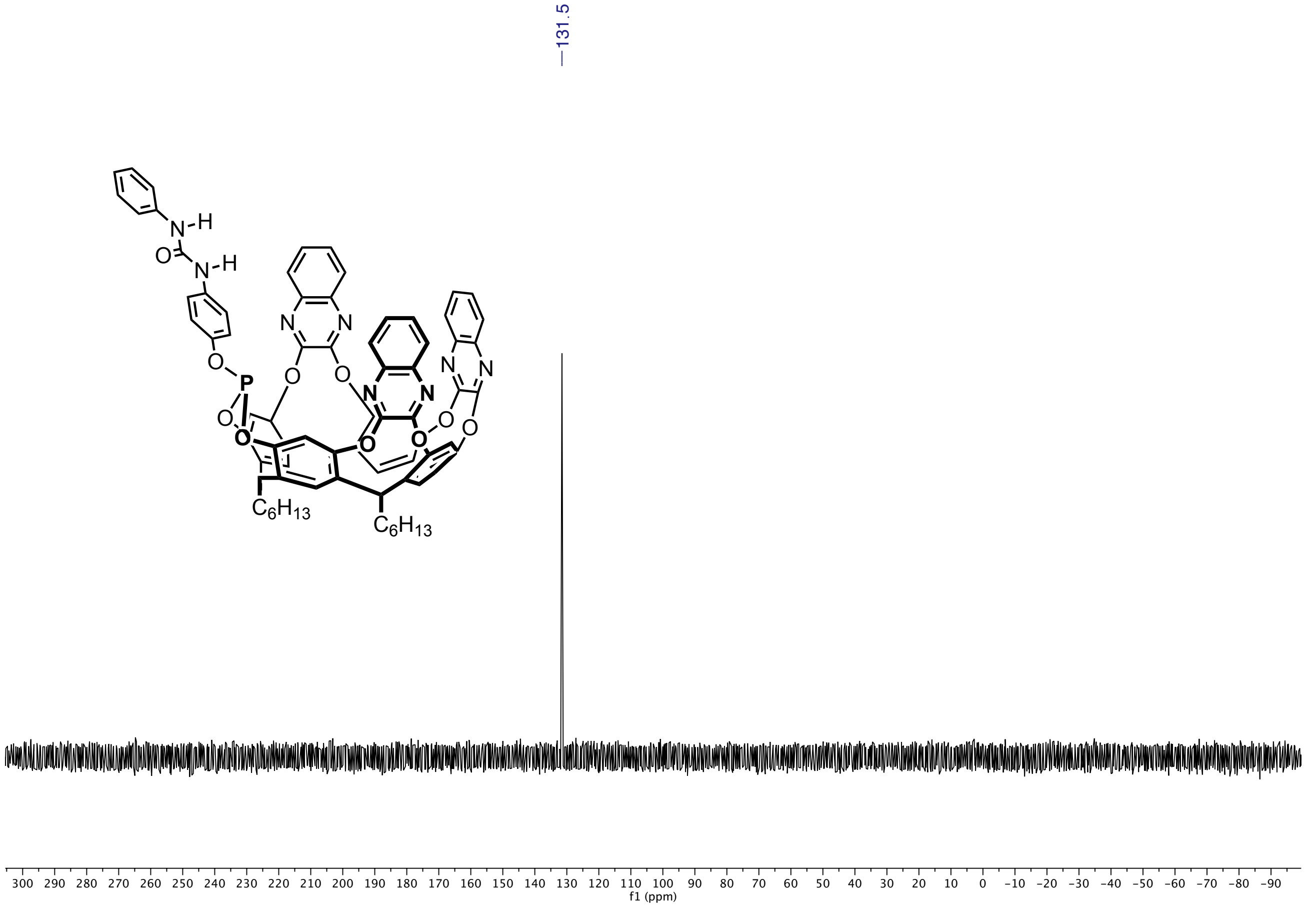

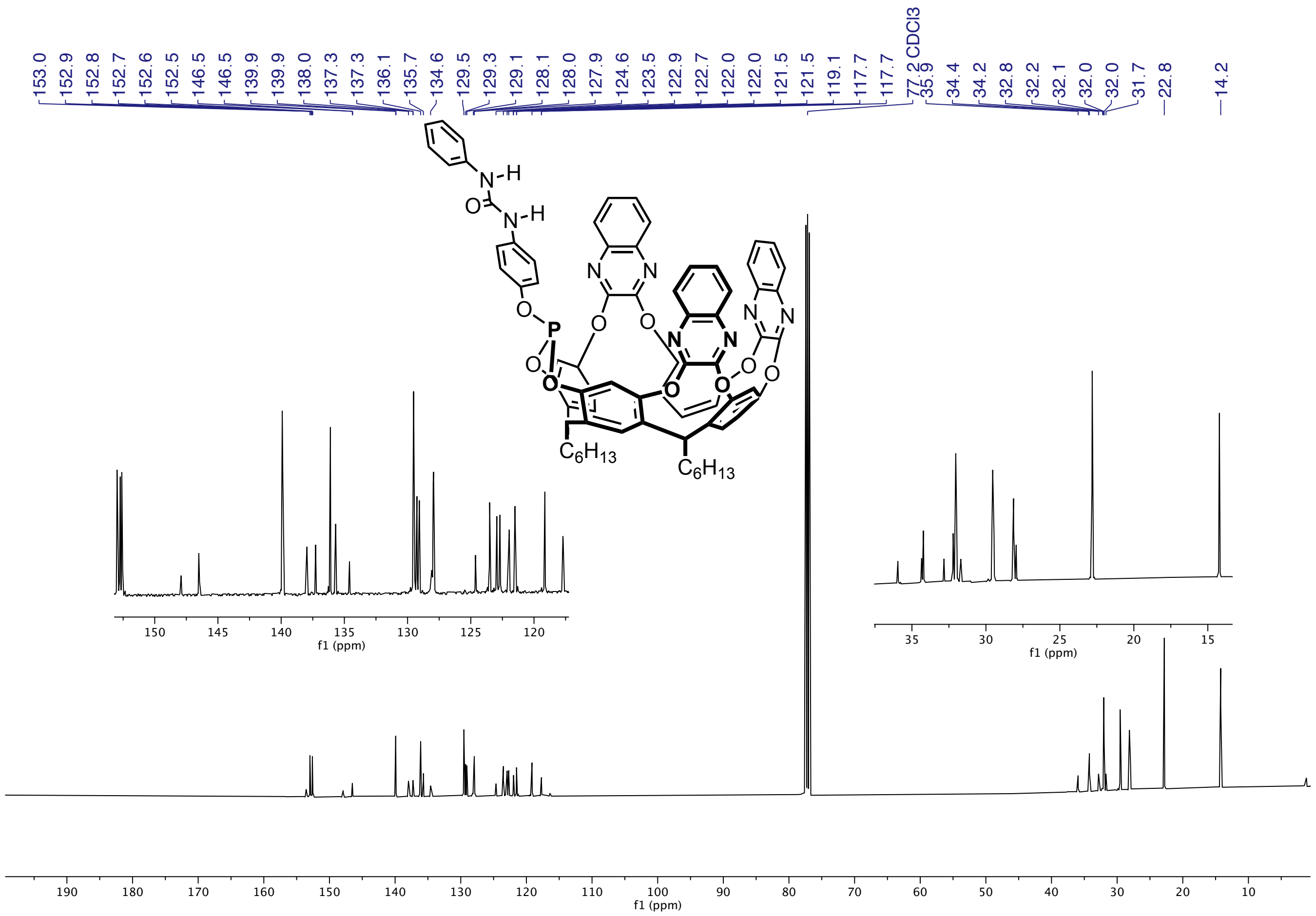

153.0
152.9
152.8
152.7
152.6
152.5
146.5
146.5
139.9
139.9
138.0
137.3
137.3
136.1
135.7
134.6
129.5
129.3
129.1
128.1
128.0
127.9
124.6
123.5
122.9
122.7
122.0
122.0
121.5
121.5
119.1
117.7
117.7
77.2 CDCl3
35.9
34.4
34.2
32.8
32.2
32.1
32.0
32.0
31.7
22.8
14.2
N-H
O
N-H
O
P
O
O
N
N
O
O
N
N
O
O
N
N
O
O
C_6H_{13}
C_6H_{13}
f1 (ppm)
f1 (ppm)
f1 (ppm)

Compound **B10** ^{1}H, $^{31}P\{^{1}H\}$, and $^{13}C\{^{1}H\}$ NMR spectra in $CDCl_3$

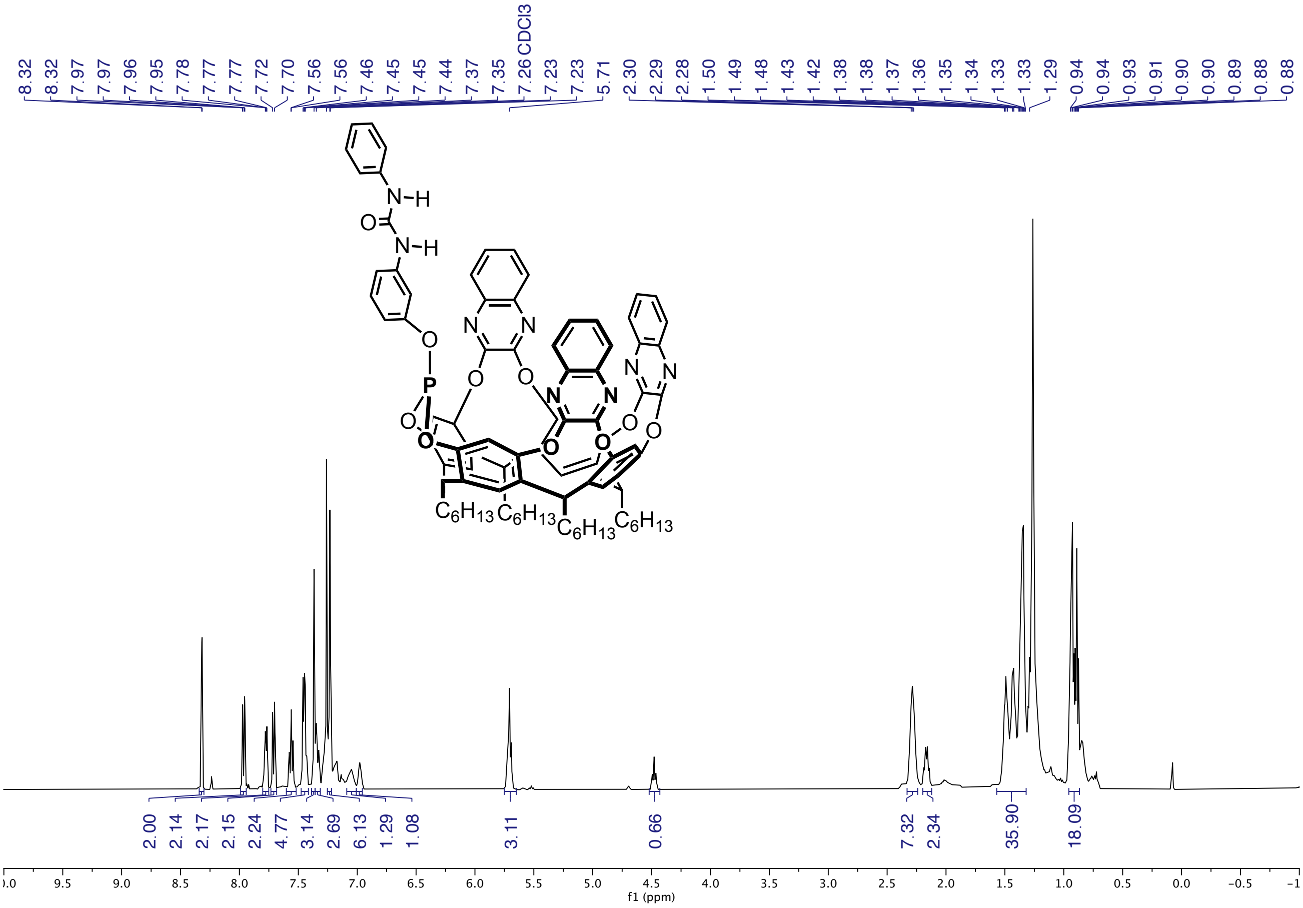

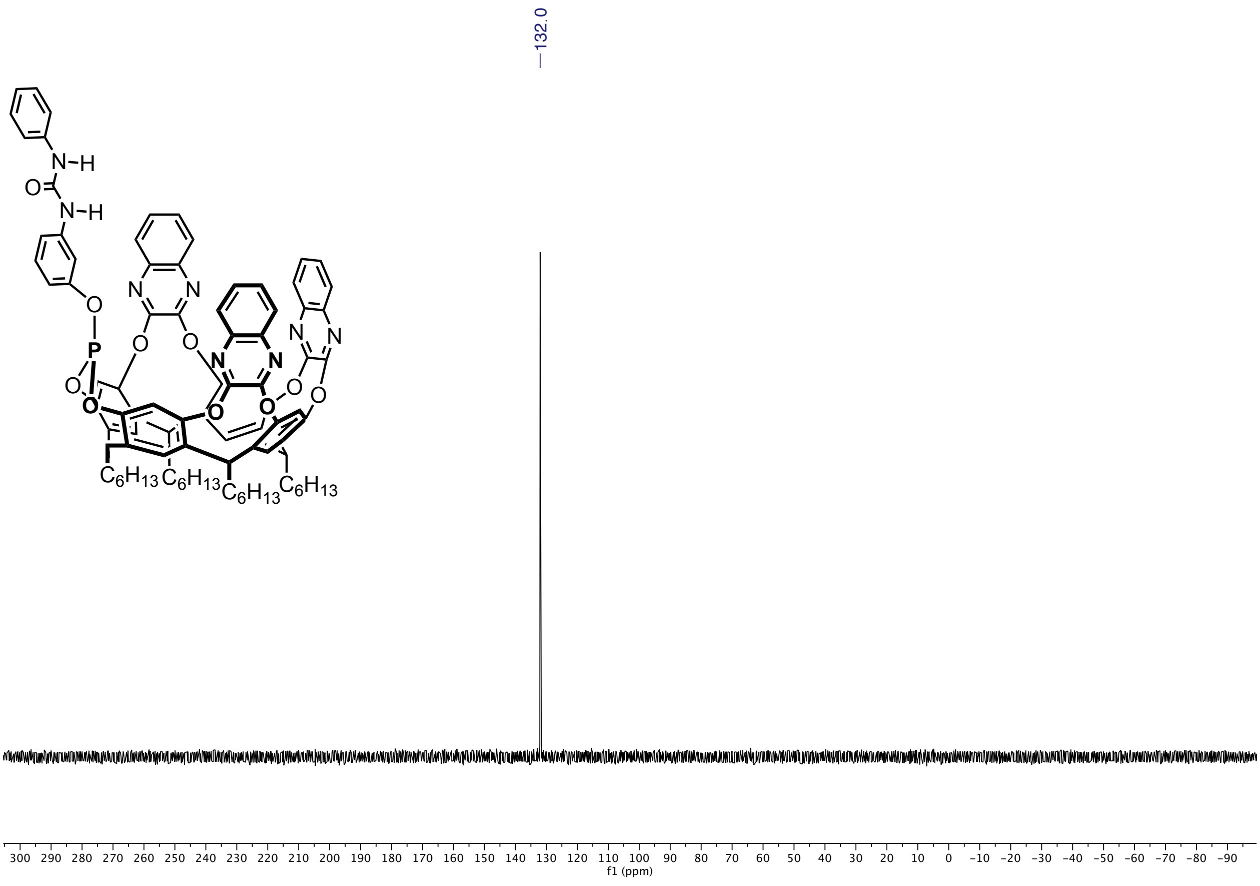

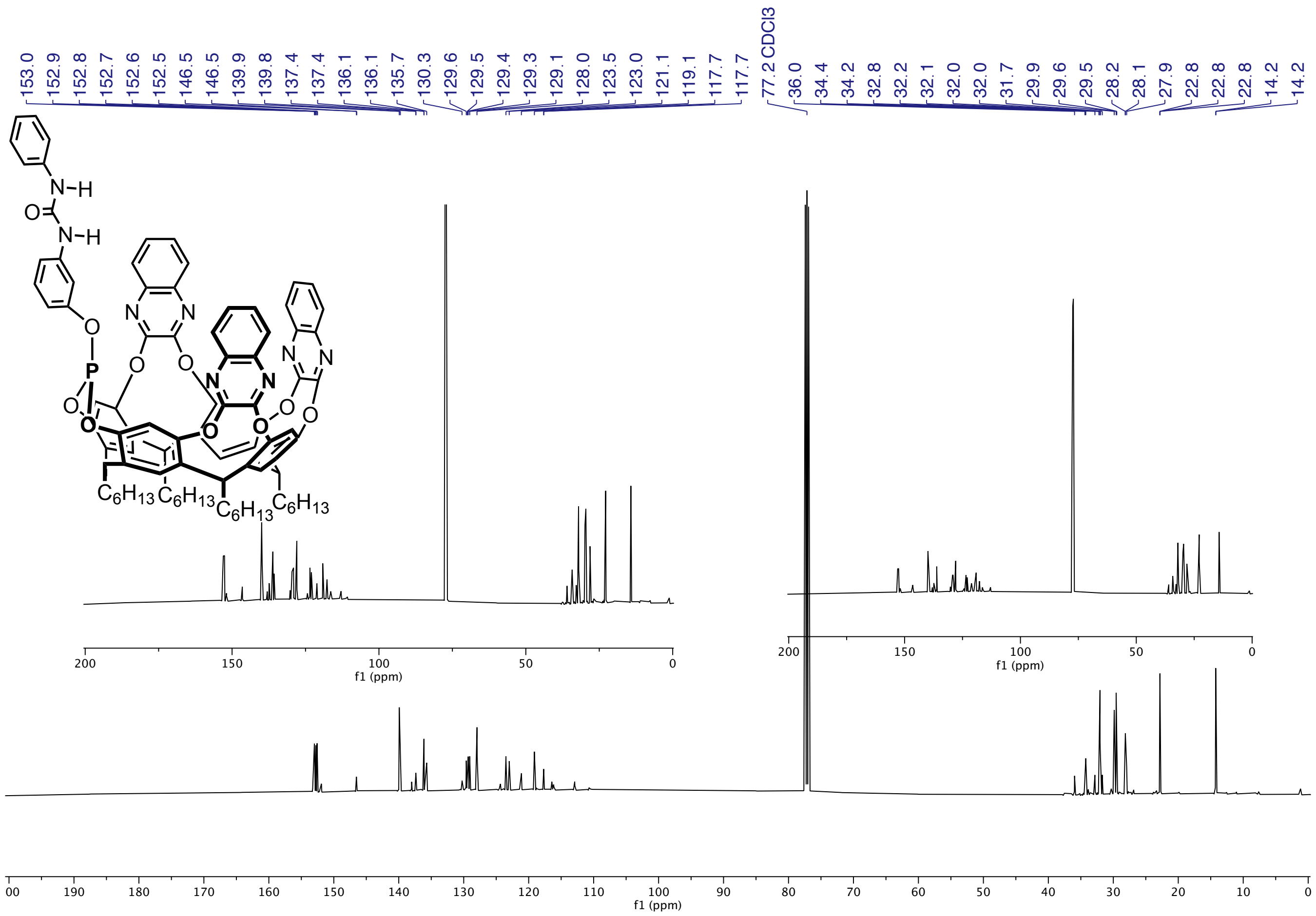
153.0
152.9
152.8
152.7
152.6
152.5
146.5
146.5
139.9
139.8
137.4
137.4
136.1
136.1
135.7
130.3
129.6
129.5
129.4
129.3
129.1
128.0
123.5
123.0
121.1
119.1
117.7
117.7
77.2 CDCl3
36.0
34.4
34.2
32.8
32.2
32.1
32.0
32.0
31.7
29.9
29.6
29.5
28.2
28.1
27.9
22.8
22.8
22.8
14.2
14.2
f1 (ppm)

Compound **B11** ^{1}H, $^{31}P\{^{1}H\}$, and $^{13}C\{^{1}H\}$ NMR spectra in $CDCl_3$

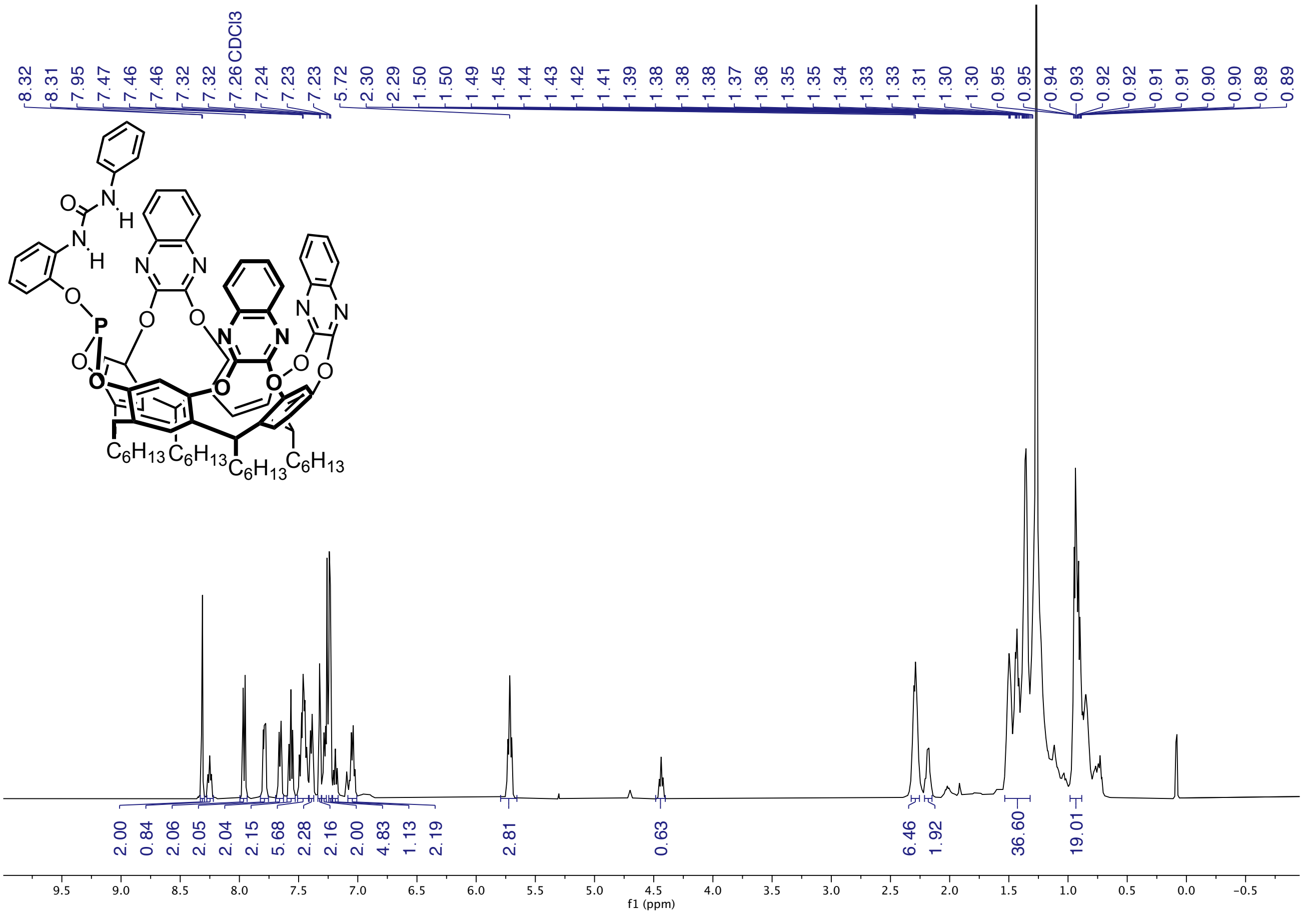

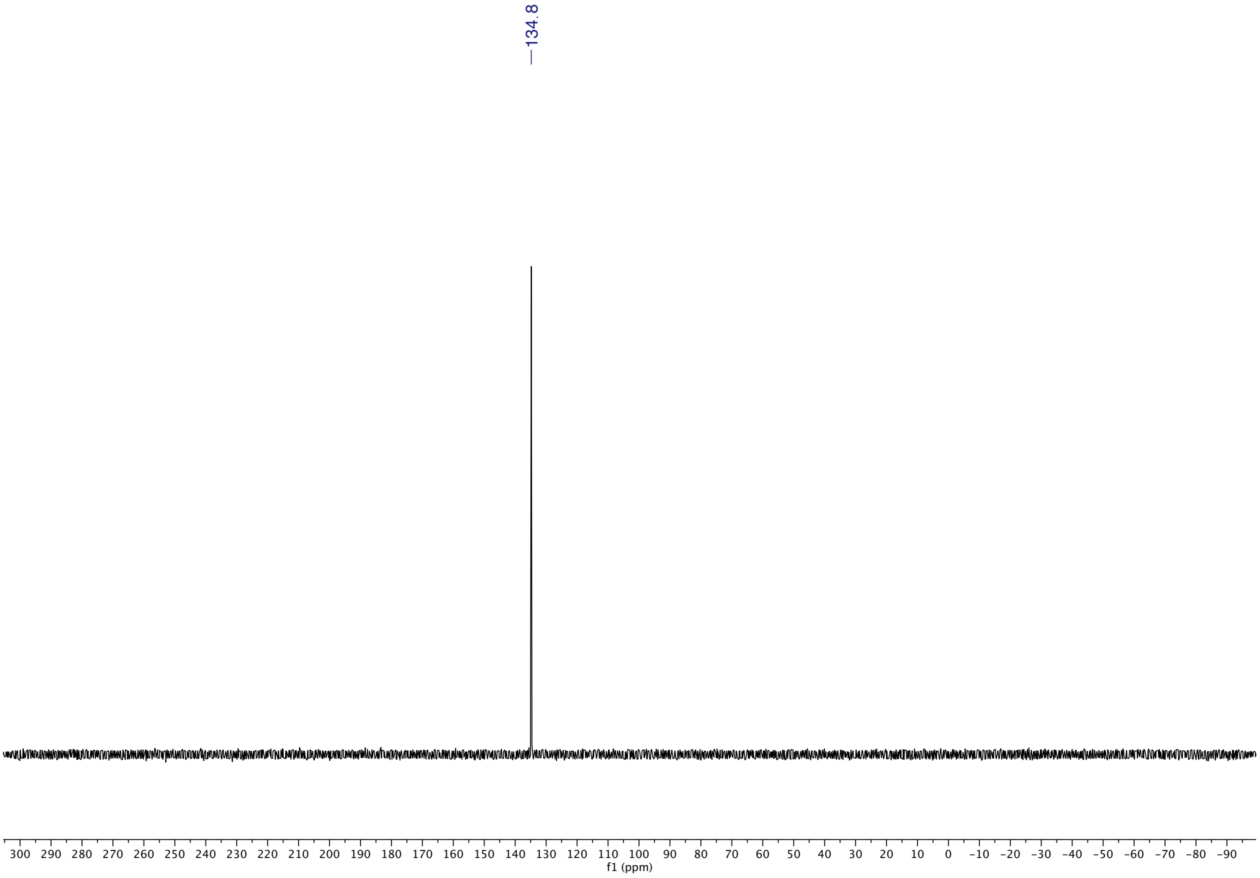

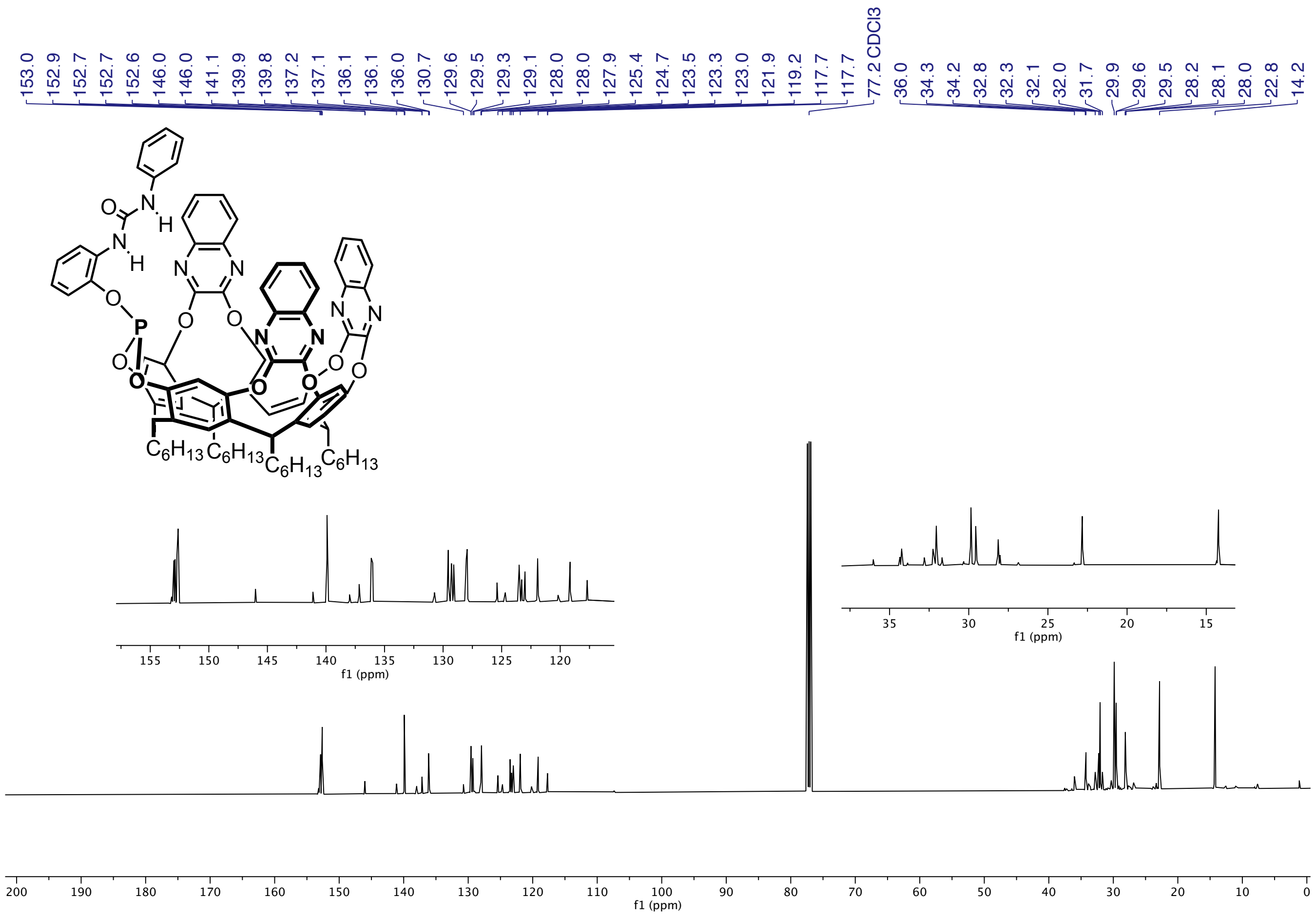
153.0
152.9
152.7
152.7
152.6
146.0
146.0
141.1
139.9
139.8
137.2
137.1
136.1
136.1
136.0
130.7
129.6
129.5
129.3
129.1
128.0
128.0
127.9
125.4
124.7
123.5
123.3
123.0
121.9
119.2
117.7
117.7
77.2 CDCl3
36.0
34.3
34.2
32.8
32.3
32.1
32.0
31.7
29.9
29.6
29.5
28.2
28.1
28.0
22.8
14.2
f1 (ppm)

Compound **(*R*)-Au1** ^{1}H, ^{31}P{^{1}H}, and ^{13}C{^{1}H} NMR spectra in CD_2Cl_2

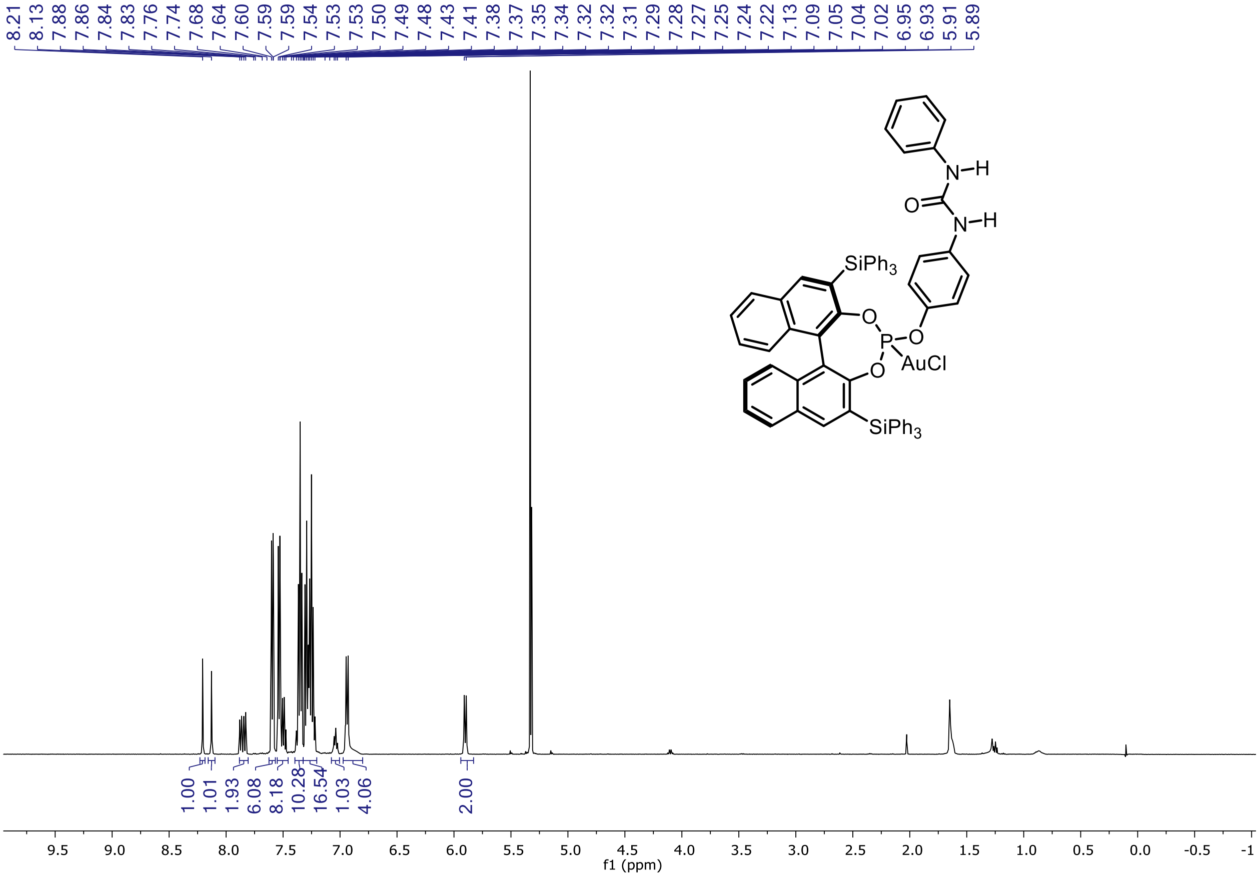

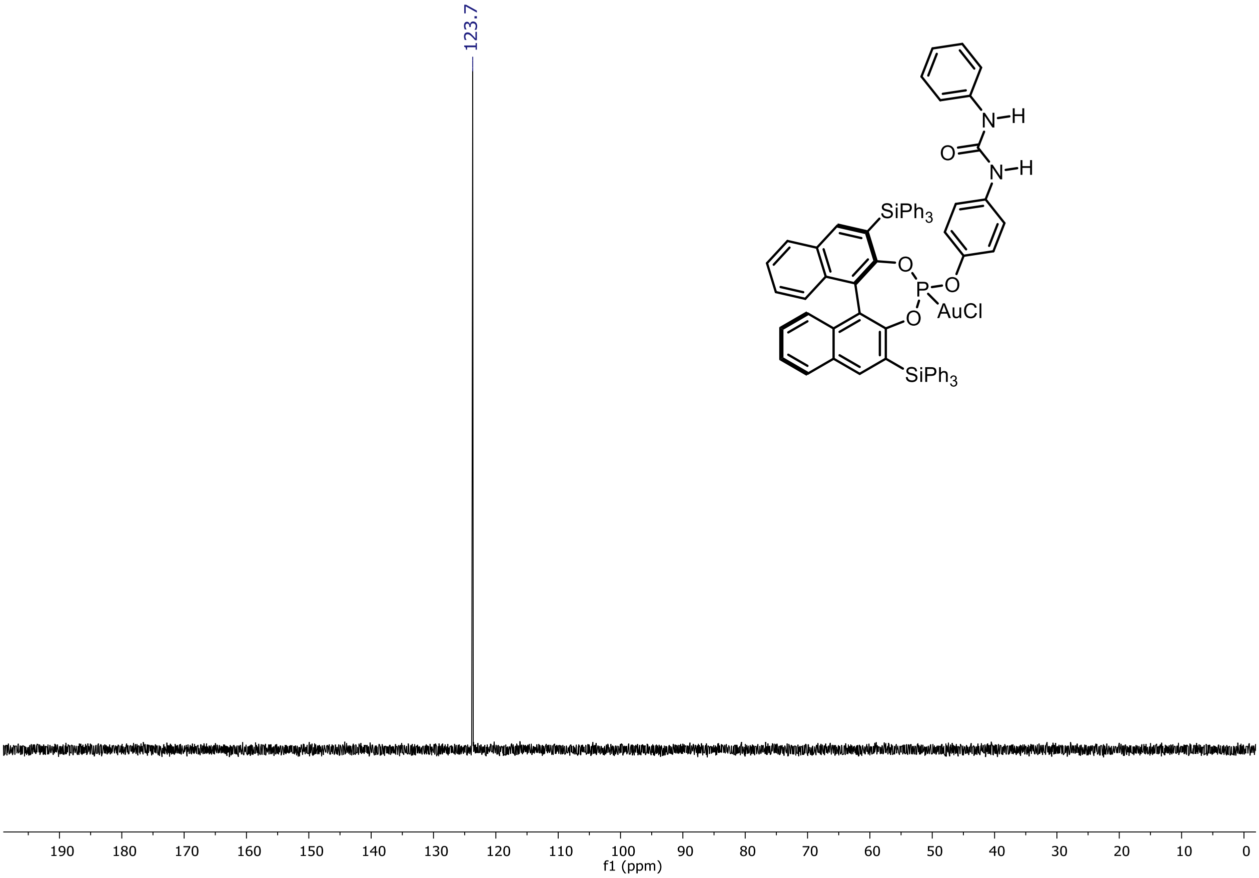

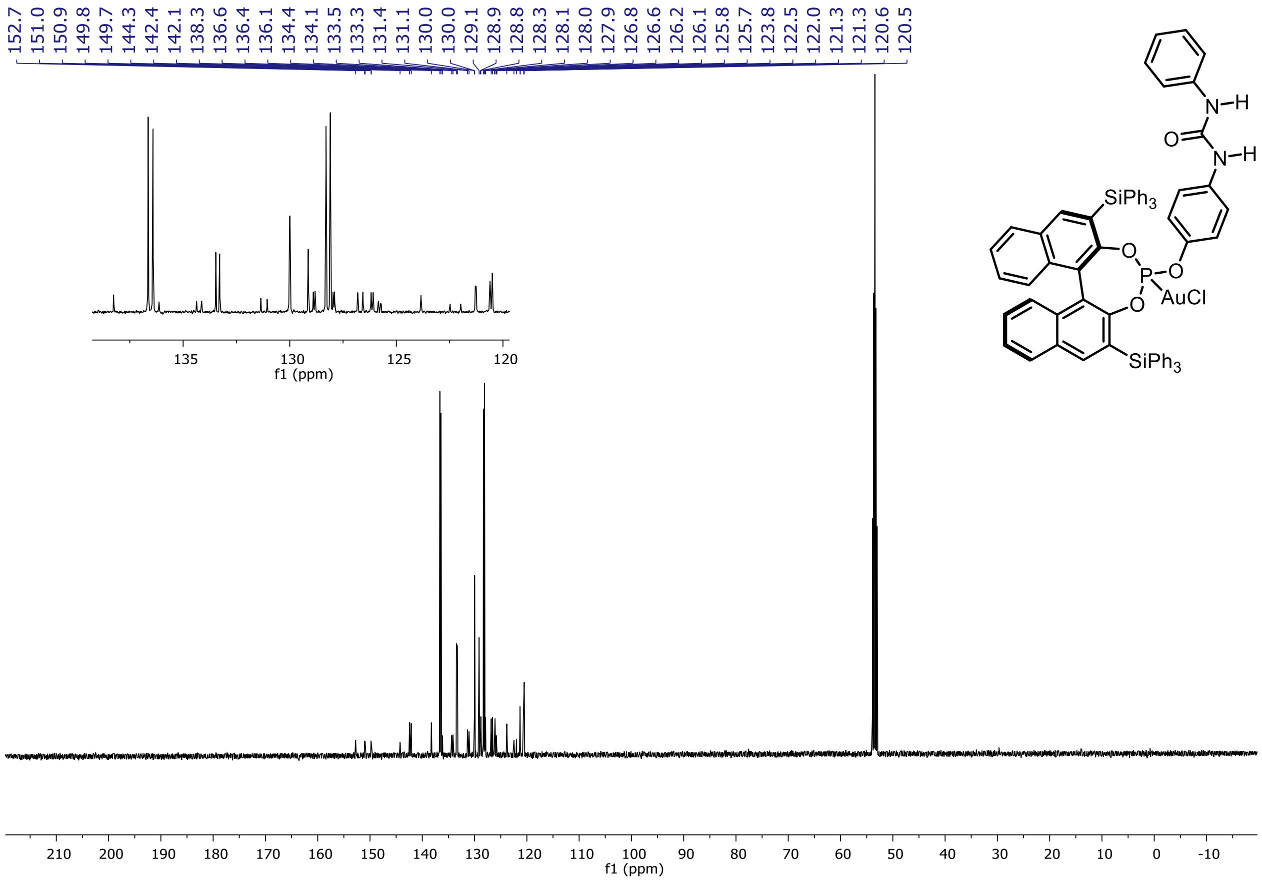
152.7
151.0
150.9
149.8
149.7
144.3
142.4
142.1
138.3
136.6
136.4
136.1
134.4
134.1
133.5
133.3
131.4
131.1
130.0
130.0
129.1
128.9
128.8
128.3
128.1
128.0
127.9
126.8
126.6
126.2
126.1
125.8
125.7
123.8
122.5
122.0
121.3
121.3
120.6
120.5
f1 (ppm)
f1 (ppm)
N–H
O
N–H
SiPh3
O
P
O
AuCl
O
SiPh3

Compound **(*R*)-Au2** ^{1}H, ^{31}P{^{1}H}, and ^{13}C{^{1}H} NMR spectra in CD_2Cl_2

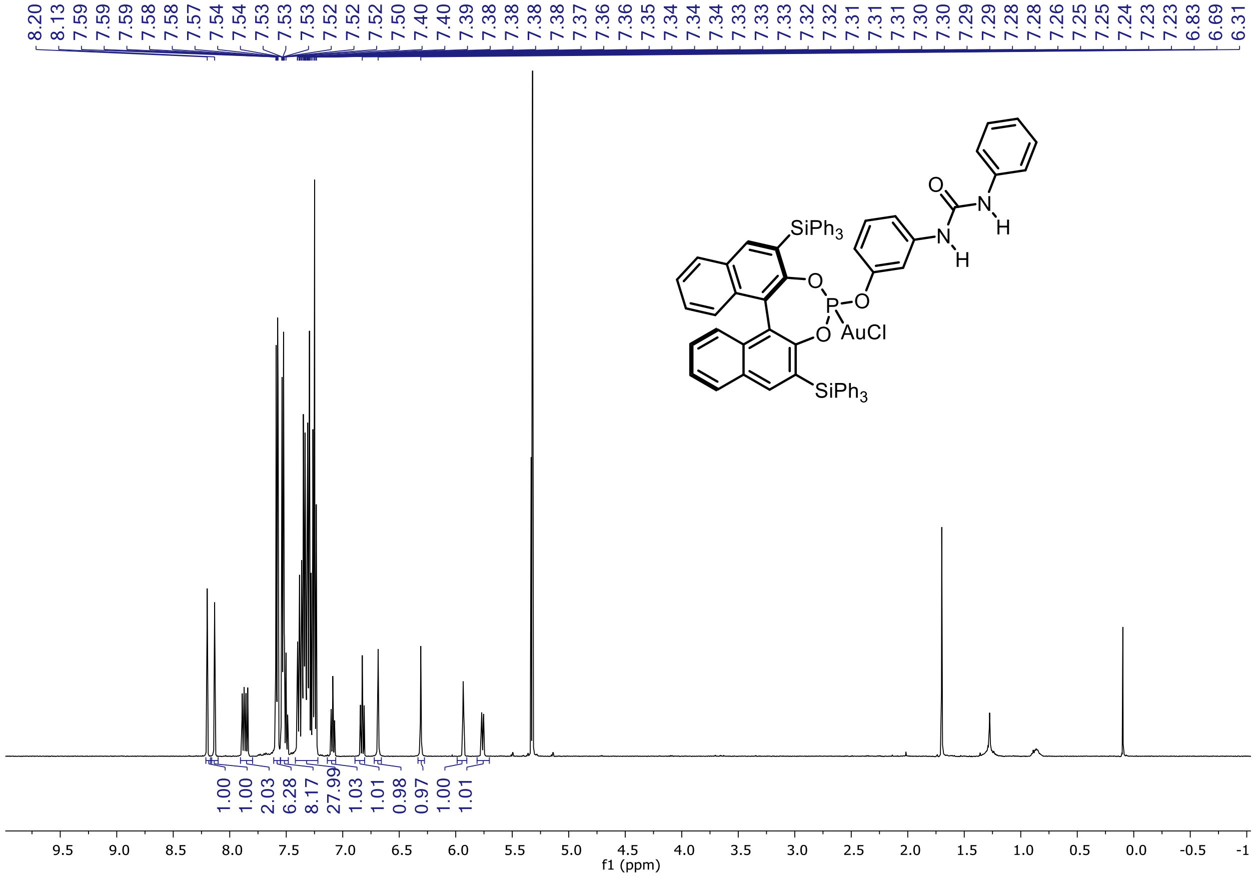

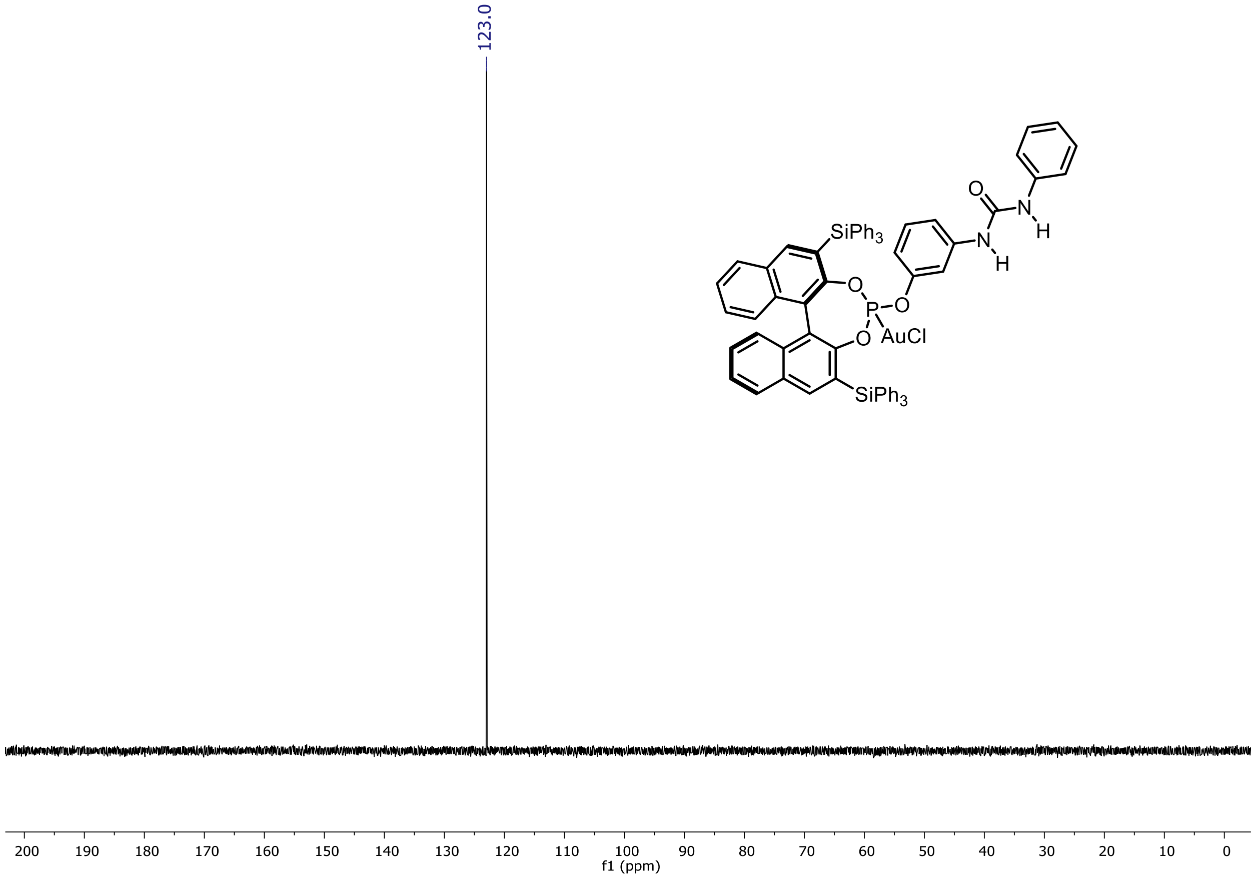

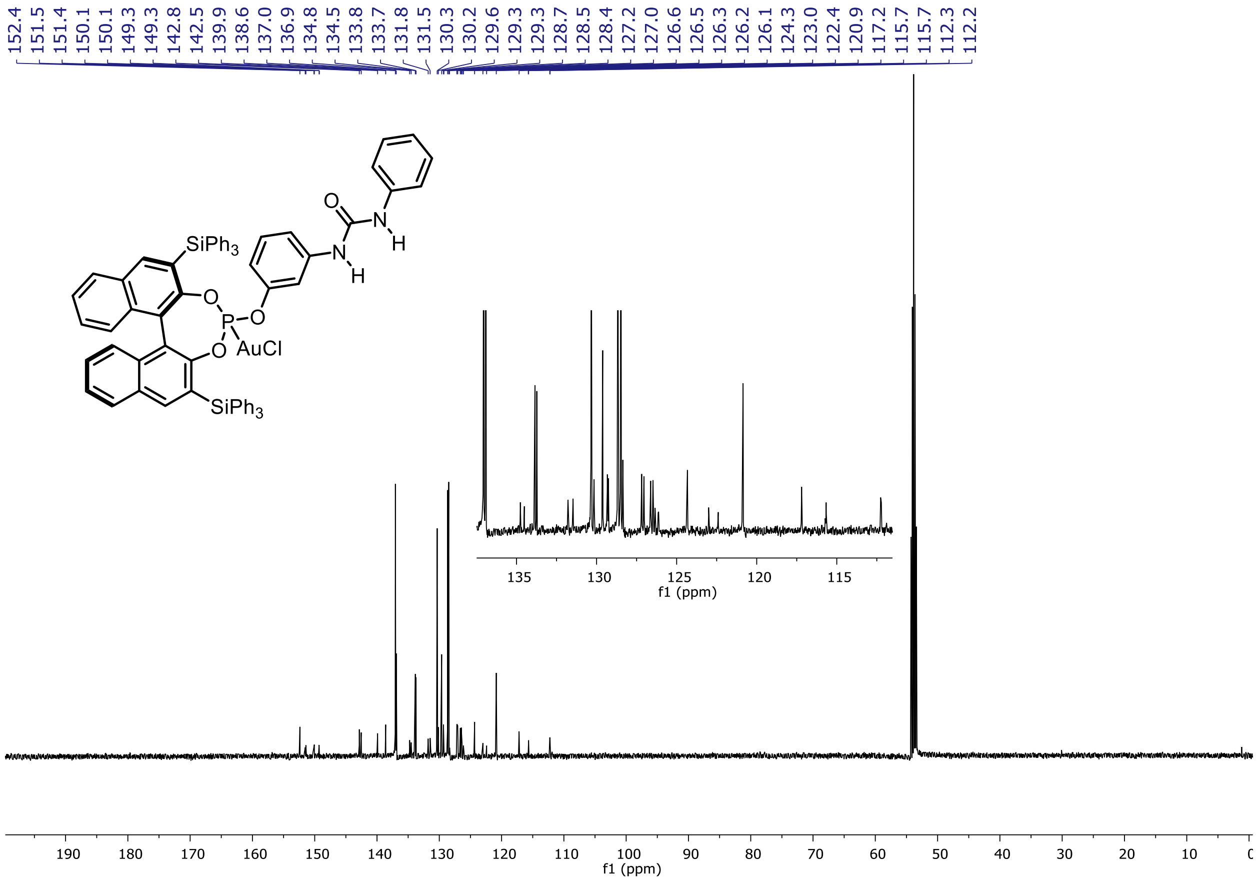

152.4
151.5
151.4
150.1
150.1
149.3
149.3
142.8
142.5
139.9
138.6
137.0
136.9
134.8
134.5
133.8
133.7
131.8
131.5
130.3
130.2
129.6
129.3
129.3
128.7
128.5
128.4
127.2
127.0
126.6
126.5
126.3
126.2
126.1
124.3
123.0
122.4
120.9
117.2
115.7
115.7
112.3
112.2
SiPh3
O
P
O
AuCl
SiPh3
N
H
O
N
H
135
130
125
120
115
f1 (ppm)
190
180
170
160
150
140
130
120
110
100
90
80
70
60
50
40
30
20
10
f1 (ppm)

Compound **(*R*)-Au3** ^{1}H, $^{31}P\{^{1}H\}$, and $^{13}C\{^{1}H\}$ NMR spectra in $CDCl_3$

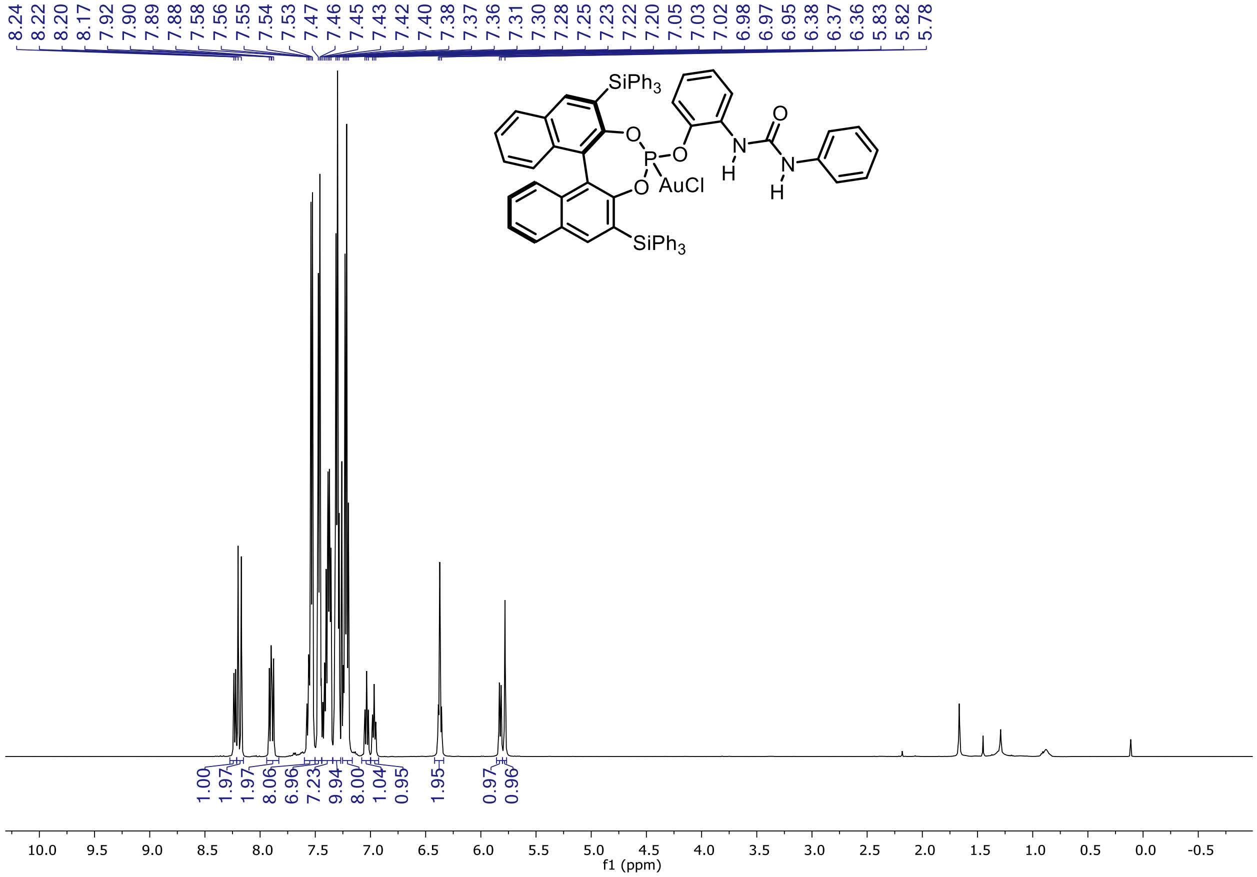

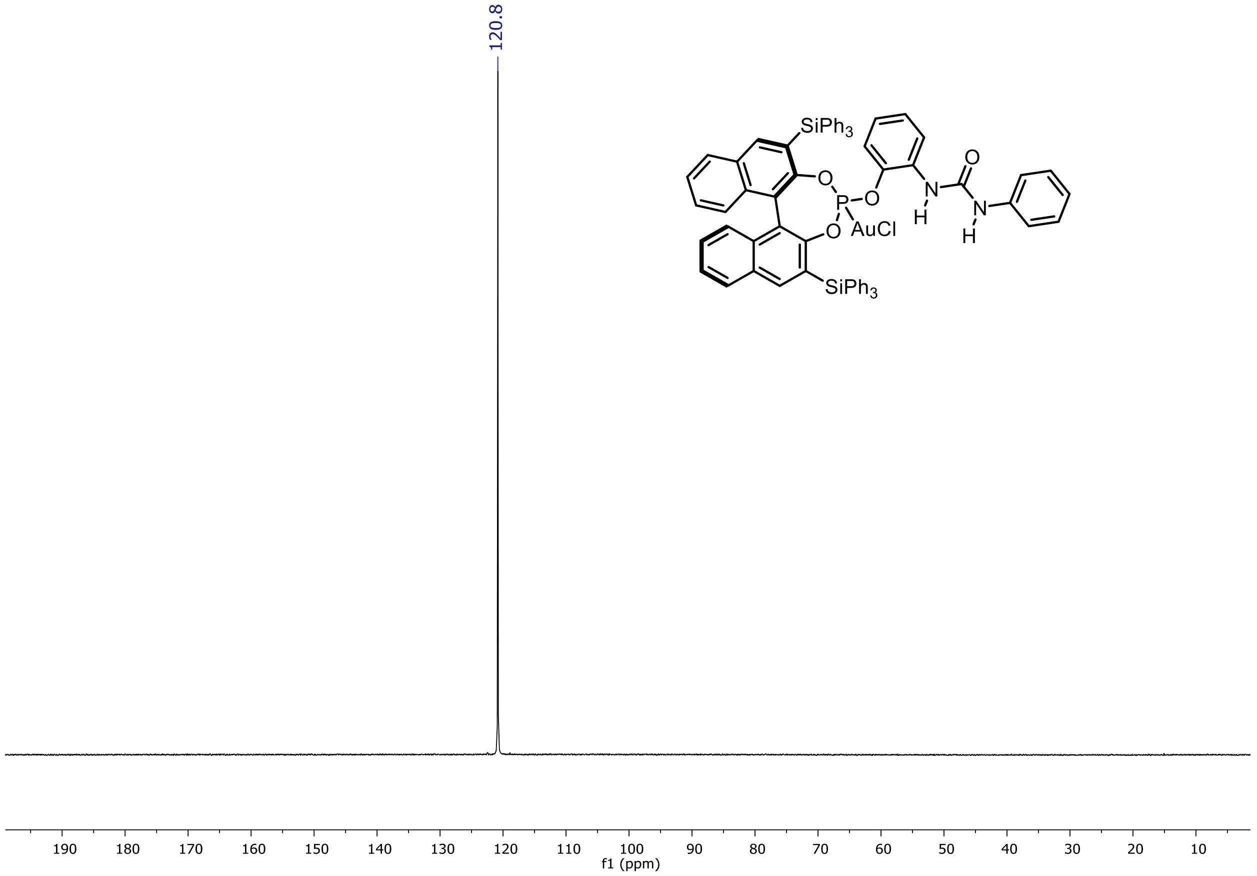

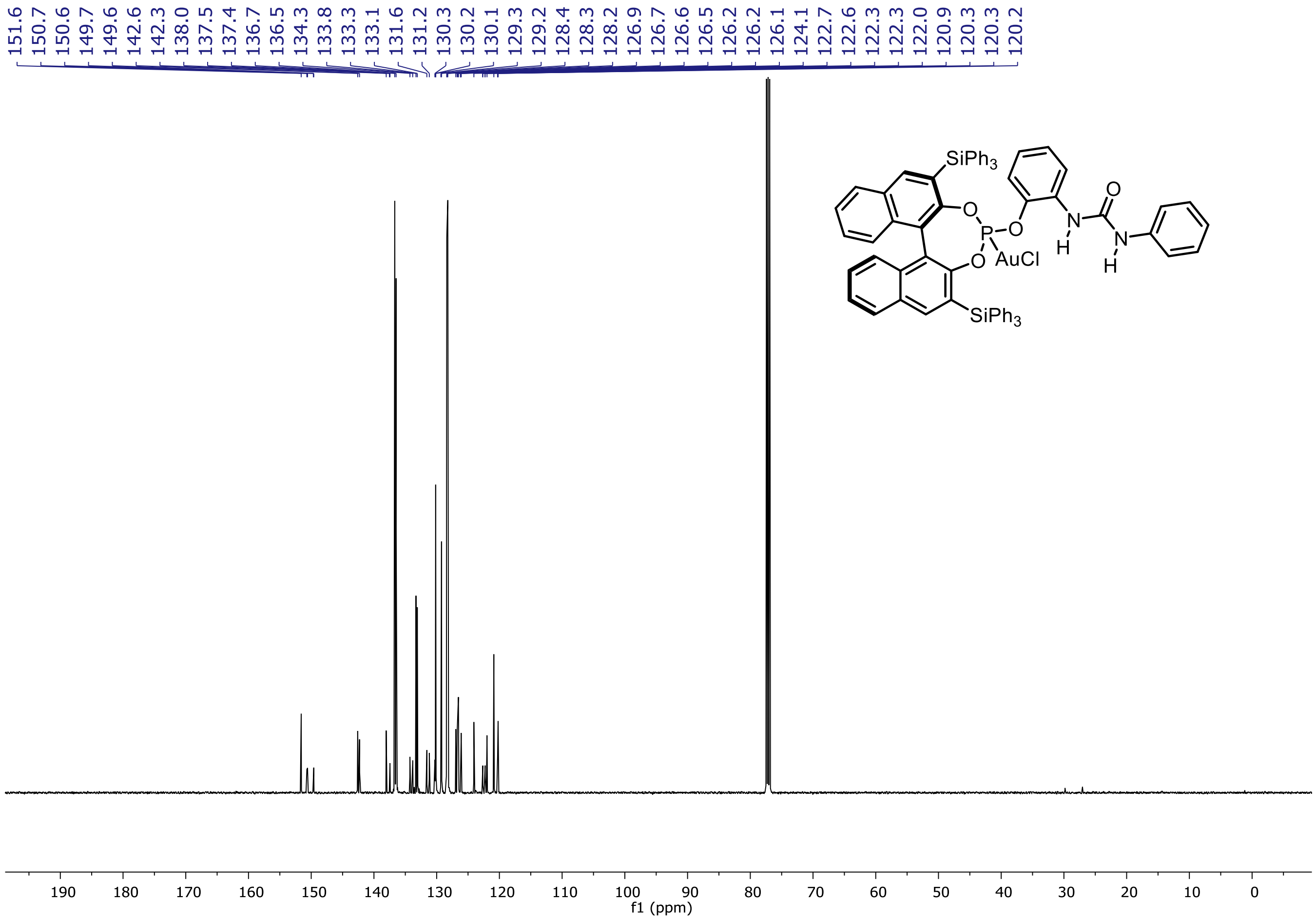
151.6
150.7
150.6
149.7
149.6
142.6
142.3
138.0
137.5
137.4
136.7
136.5
134.3
133.8
133.3
133.1
131.6
131.2
130.3
130.2
130.1
129.3
129.2
128.4
128.3
128.2
126.9
126.7
126.6
126.5
126.2
126.2
126.1
124.1
122.7
122.6
122.3
122.3
122.0
120.9
120.3
120.3
120.2
SiPh3
O
P
O
AuCl
N
H
N
H
SiPh3
190
180
170
160
150
140
130
120
110
100
90
80
70
60
50
40
30
20
10
0
f1 (ppm)

Compound **(*R*)-Au4** ^{1}H, ^{31}P{^{1}H}, and ^{13}C{^{1}H} NMR spectra in CD_2Cl_2

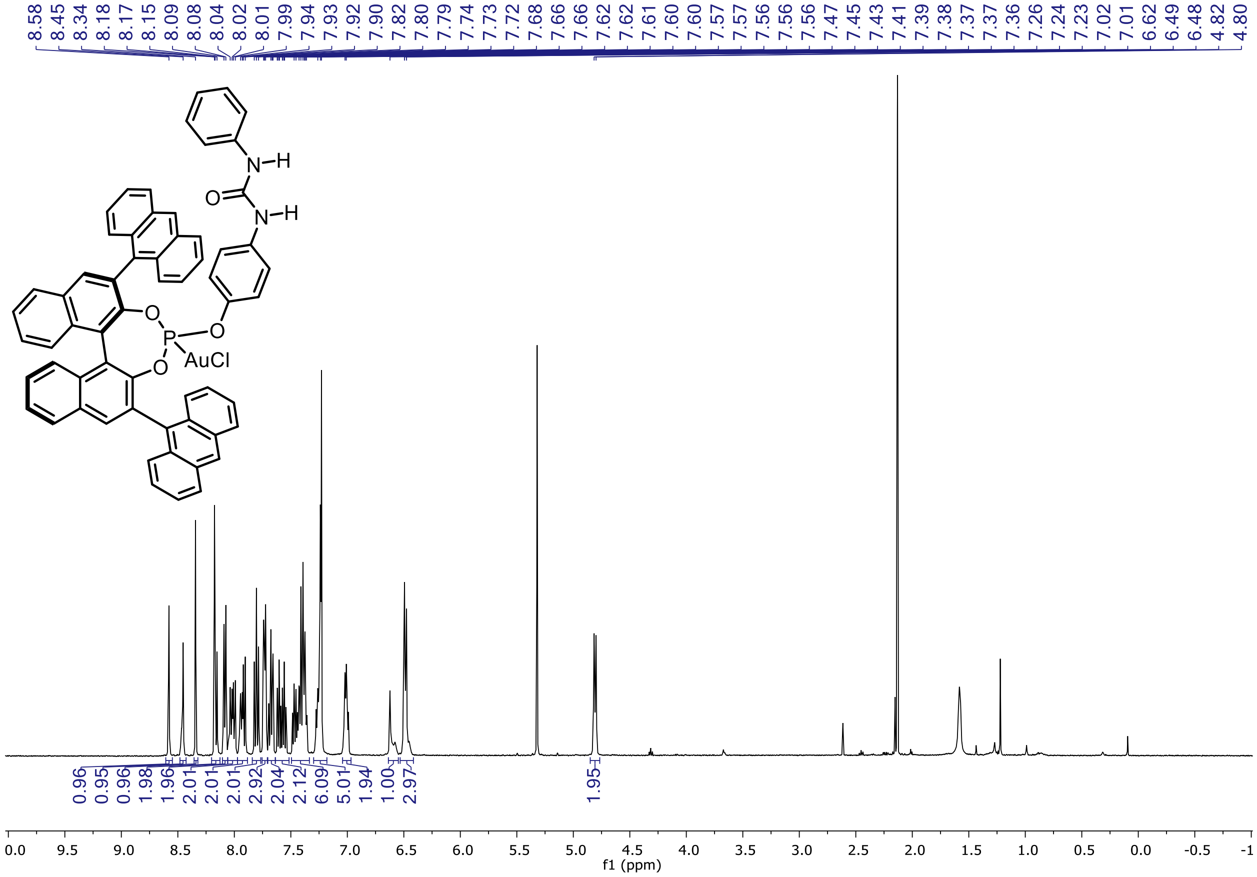

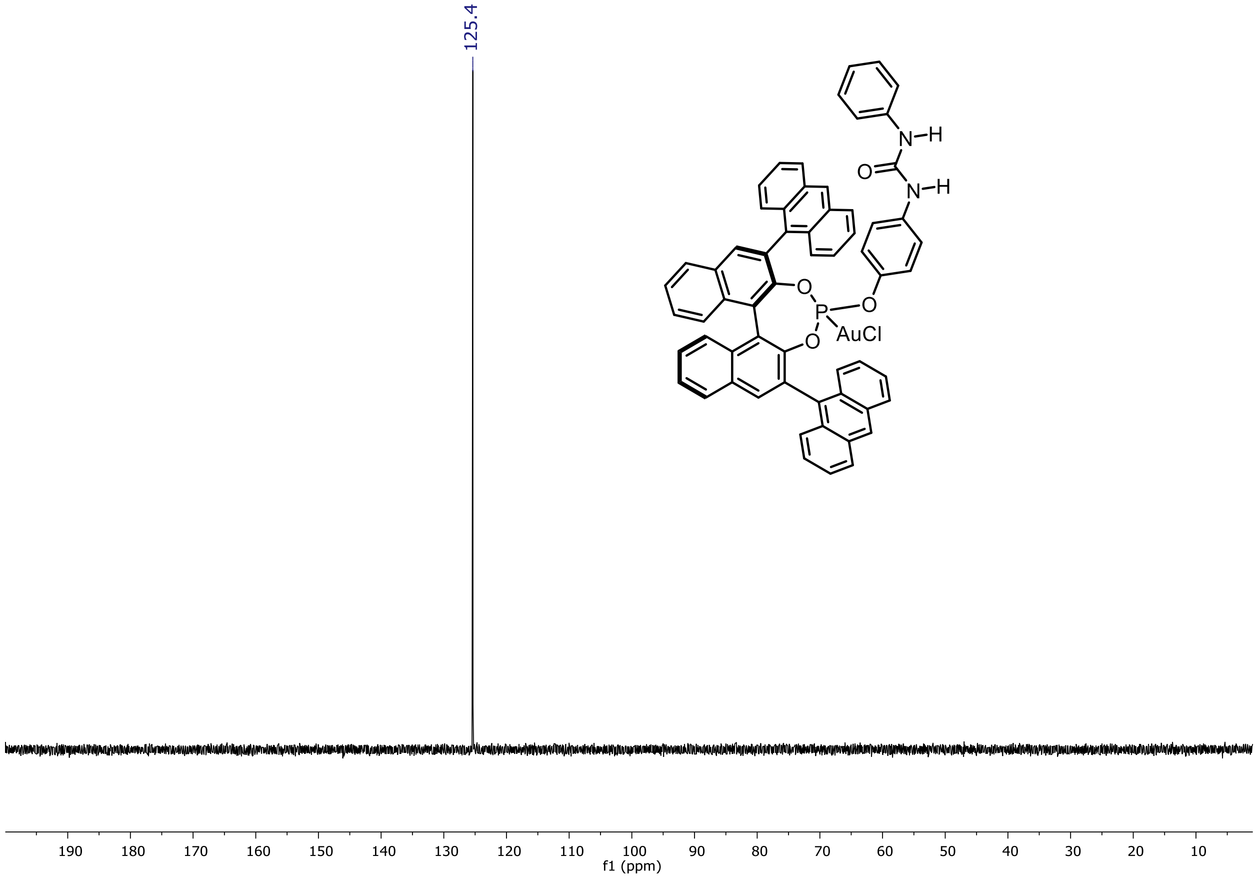

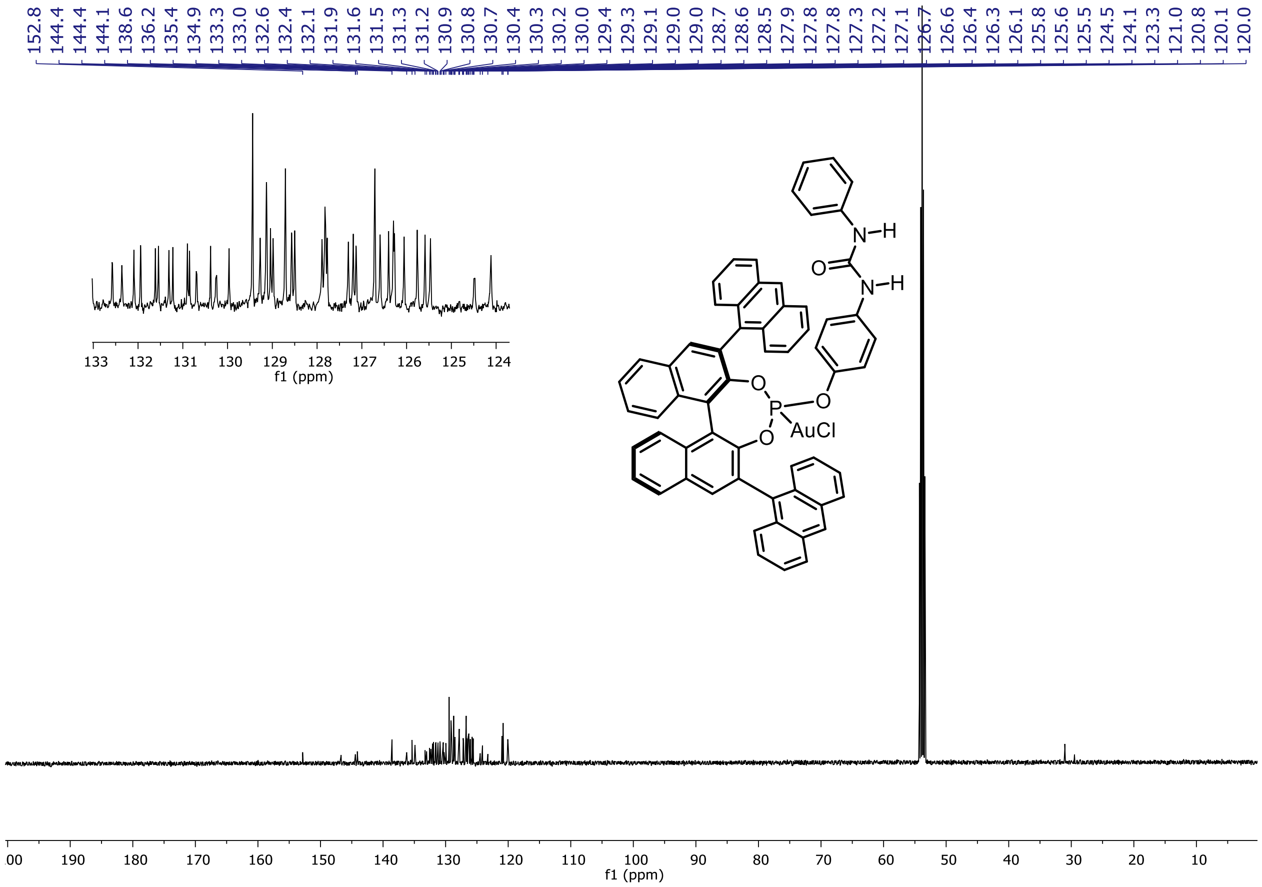

Compound **(*R*)-Au5** ^{1}H, $^{31}P\{^{1}H\}$, and $^{13}C\{^{1}H\}$ NMR spectra in CD_2Cl_2

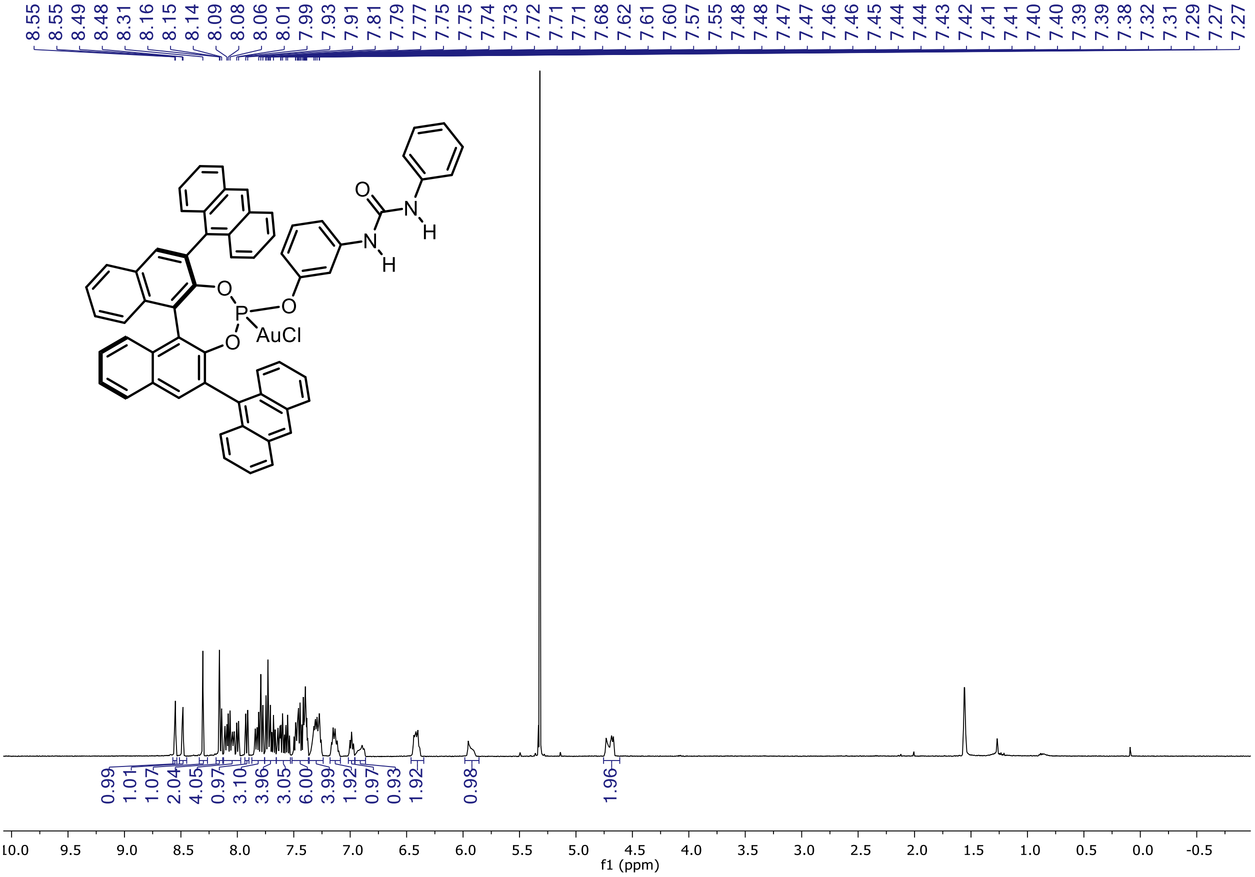

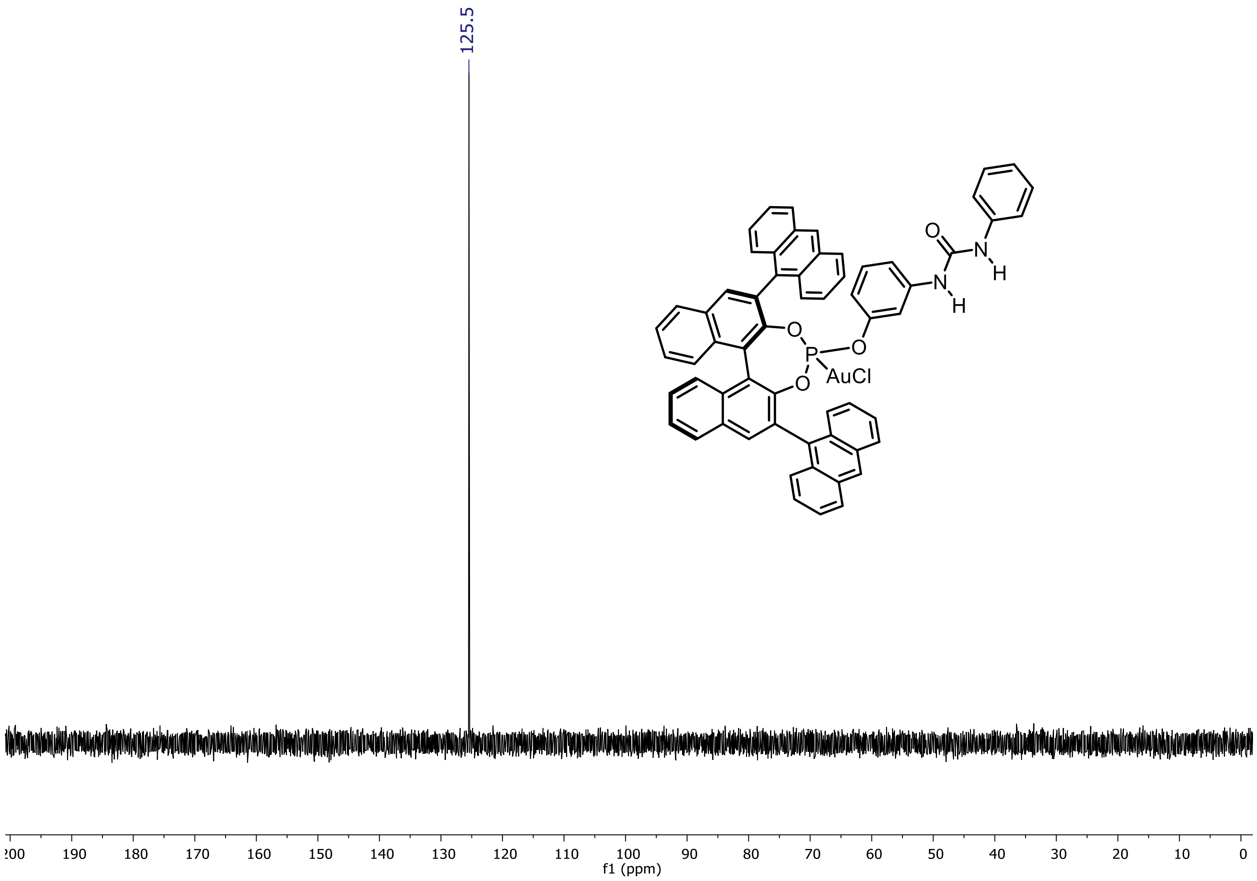
125.5
O
N
H
N
H
O
O
P
O
AuCl
200 190 180 170 160 150 140 130 120 110 100 90 80 70 60 50 40 30 20 10 0
f1 (ppm)

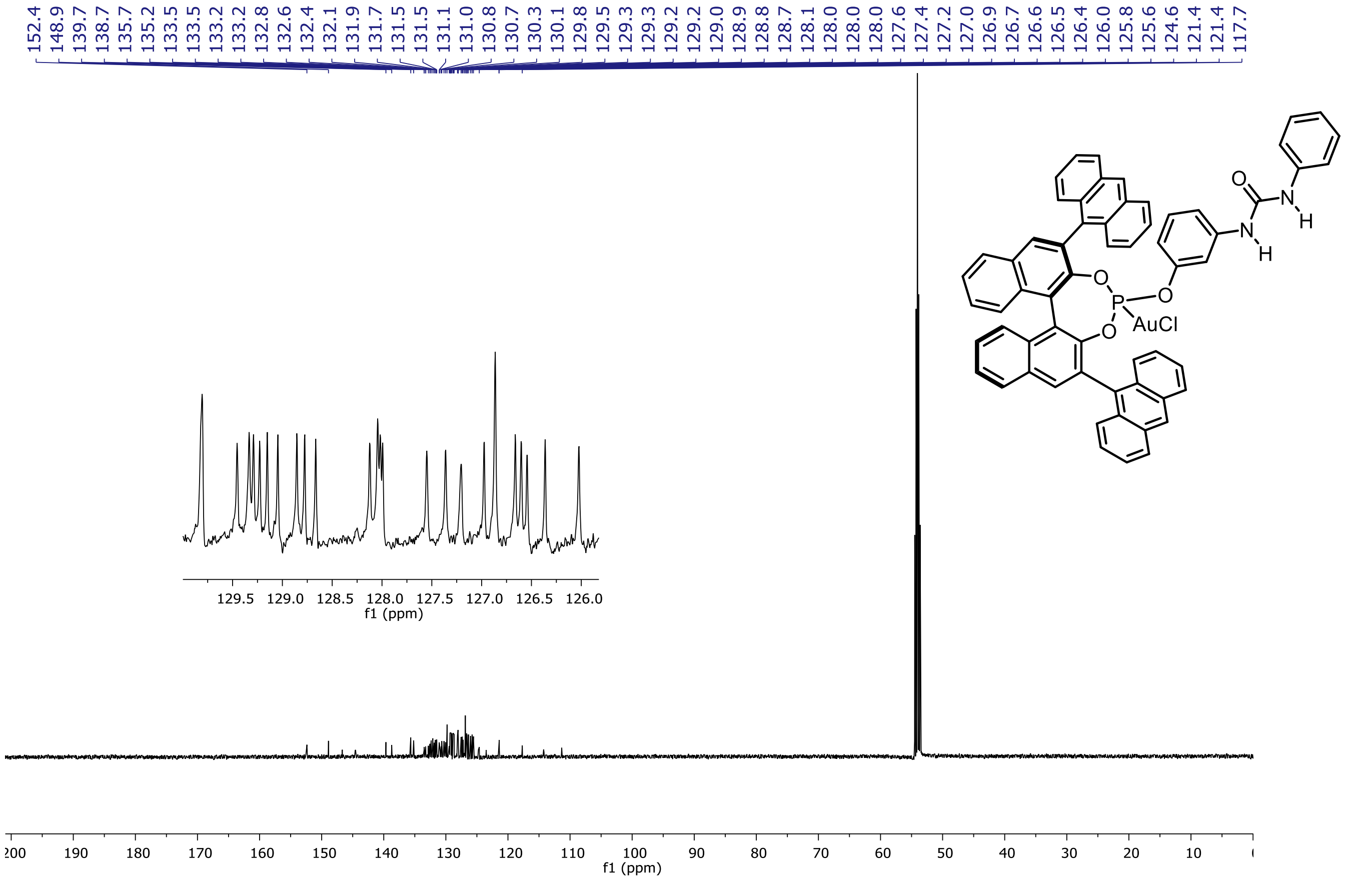
152.4 148.9 139.7 138.7 135.7 135.2 133.5 133.5 133.2 133.2 132.8 132.6 132.4 132.1 131.9 131.7 131.5 131.5 131.1 131.0 130.8 130.7 130.3 130.1 129.8 129.5 129.3 129.3 129.2 129.2 129.0 128.9 128.8 128.7 128.1 128.0 128.0 128.0 127.6 127.4 127.2 127.0 126.9 126.7 126.6 126.5 126.4 126.0 125.8 125.6 124.6 121.4 121.4 117.7
O
N
H
N
H
O
O
P
O
AuCl
129.5 129.0 128.5 128.0 127.5 127.0 126.5 126.0
f1 (ppm)
200 190 180 170 160 150 140 130 120 110 100 90 80 70 60 50 40 30 20 10
f1 (ppm)

Compound **(*R*)-Au6** ^{1}H, ^{31}P{^{1}H}, and ^{13}C{^{1}H} NMR spectra in $CDCl_3$

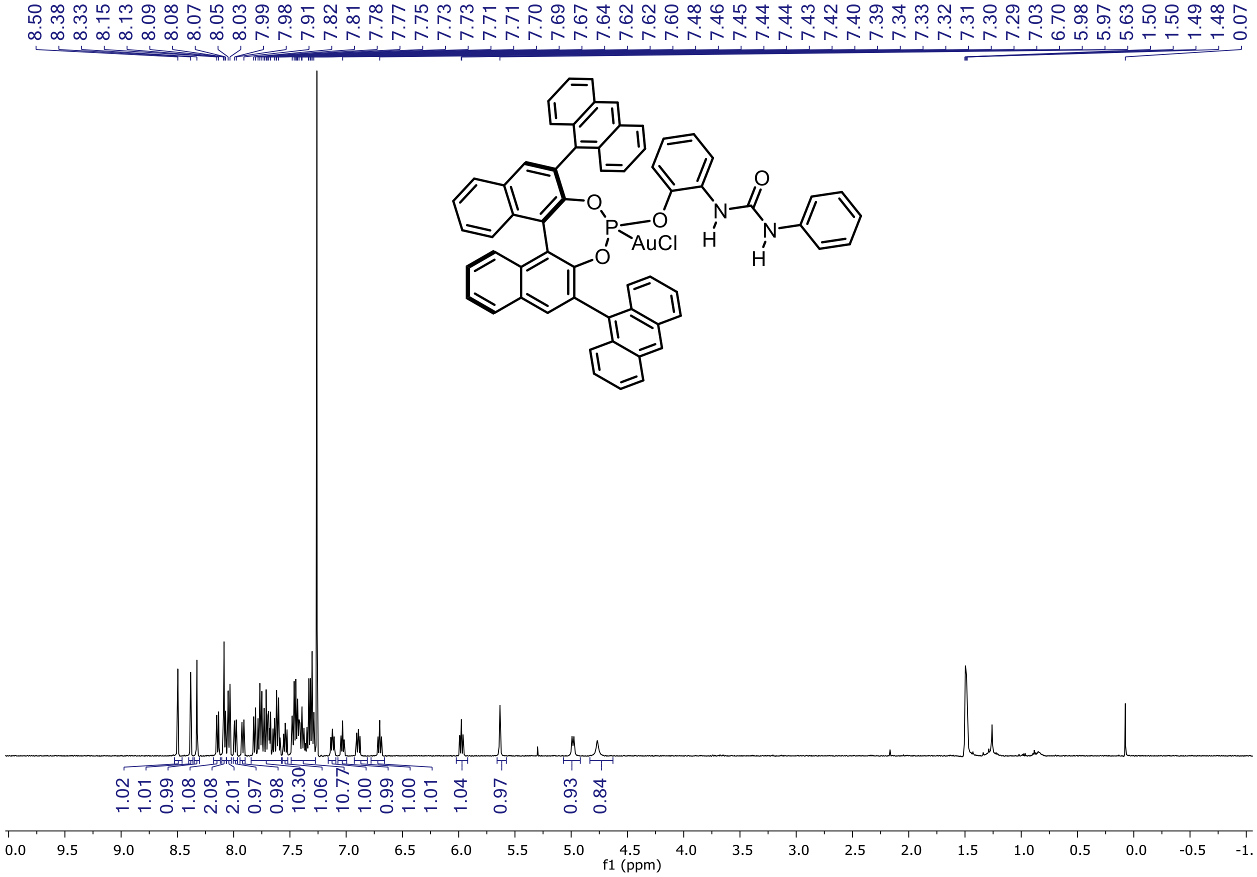

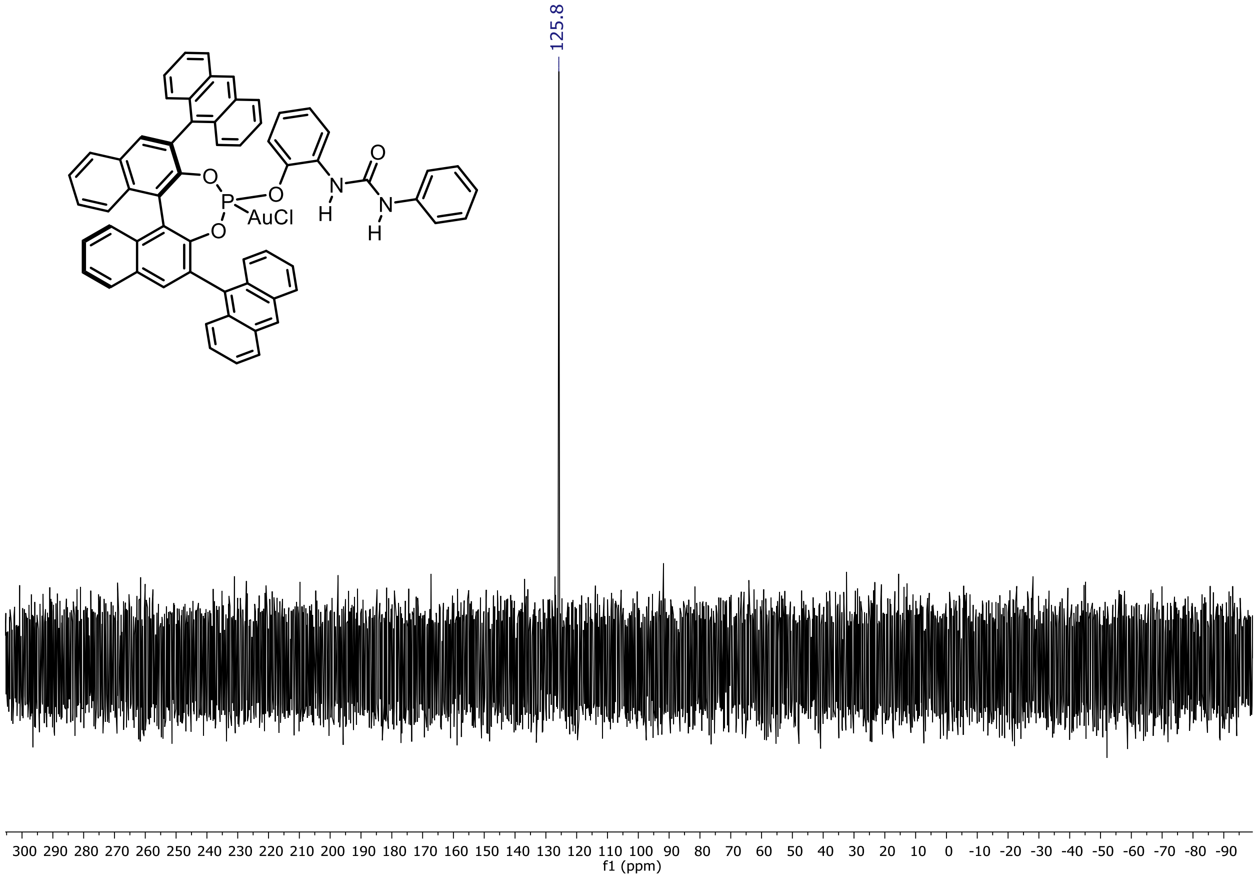

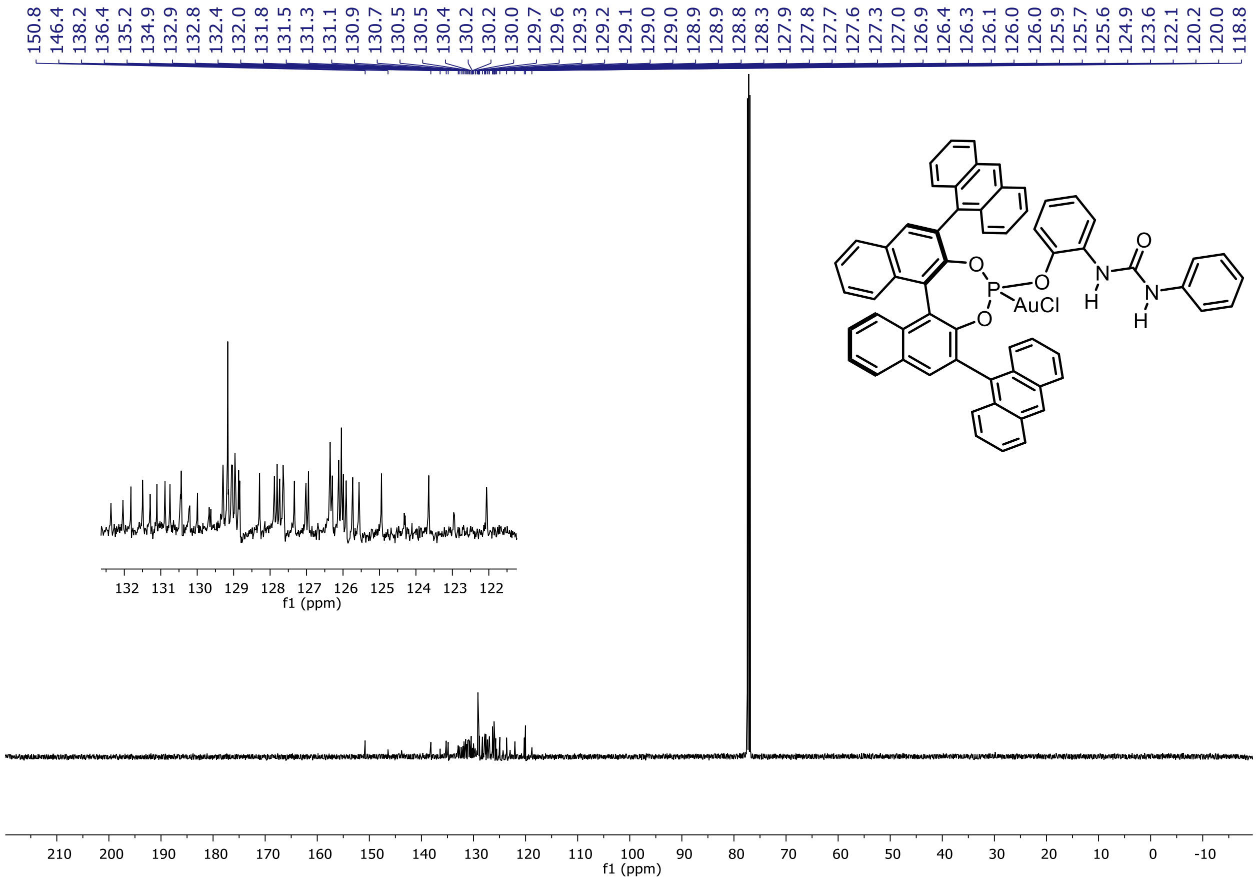

150.8
146.4
138.2
136.4
135.2
134.9
132.9
132.8
132.4
132.0
131.8
131.5
131.3
131.1
130.9
130.7
130.5
130.4
130.2
130.2
130.0
129.7
129.6
129.3
129.2
129.1
129.0
129.0
128.9
128.9
128.8
128.3
127.9
127.8
127.7
127.6
127.3
127.0
126.9
126.4
126.3
126.1
126.0
126.0
125.9
125.7
125.6
124.9
123.6
122.1
120.2
120.0
118.8
AuCl
132 131 130 129 128 127 126 125 124 123 122
f1 (ppm)
210 200 190 180 170 160 150 140 130 120 110 100 90 80 70 60 50 40 30 20 10 0 -10
f1 (ppm)

Compound (***R***)-**Au7** ^{1}H, $^{31}P\{^{1}H\}$, $^{19}F\{^{1}H\}$ and $^{13}C\{^{1}H\}$ NMR spectra in $CDCl_3$

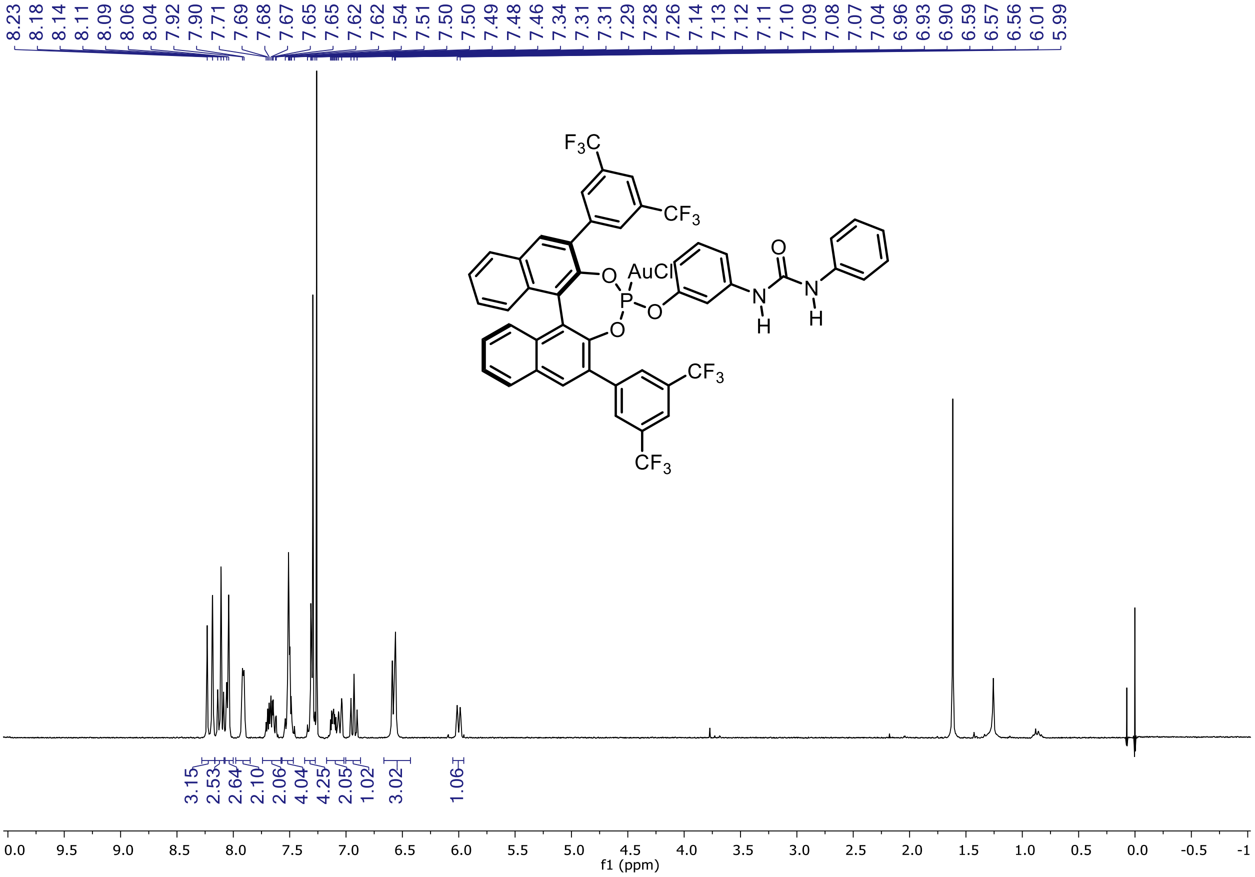

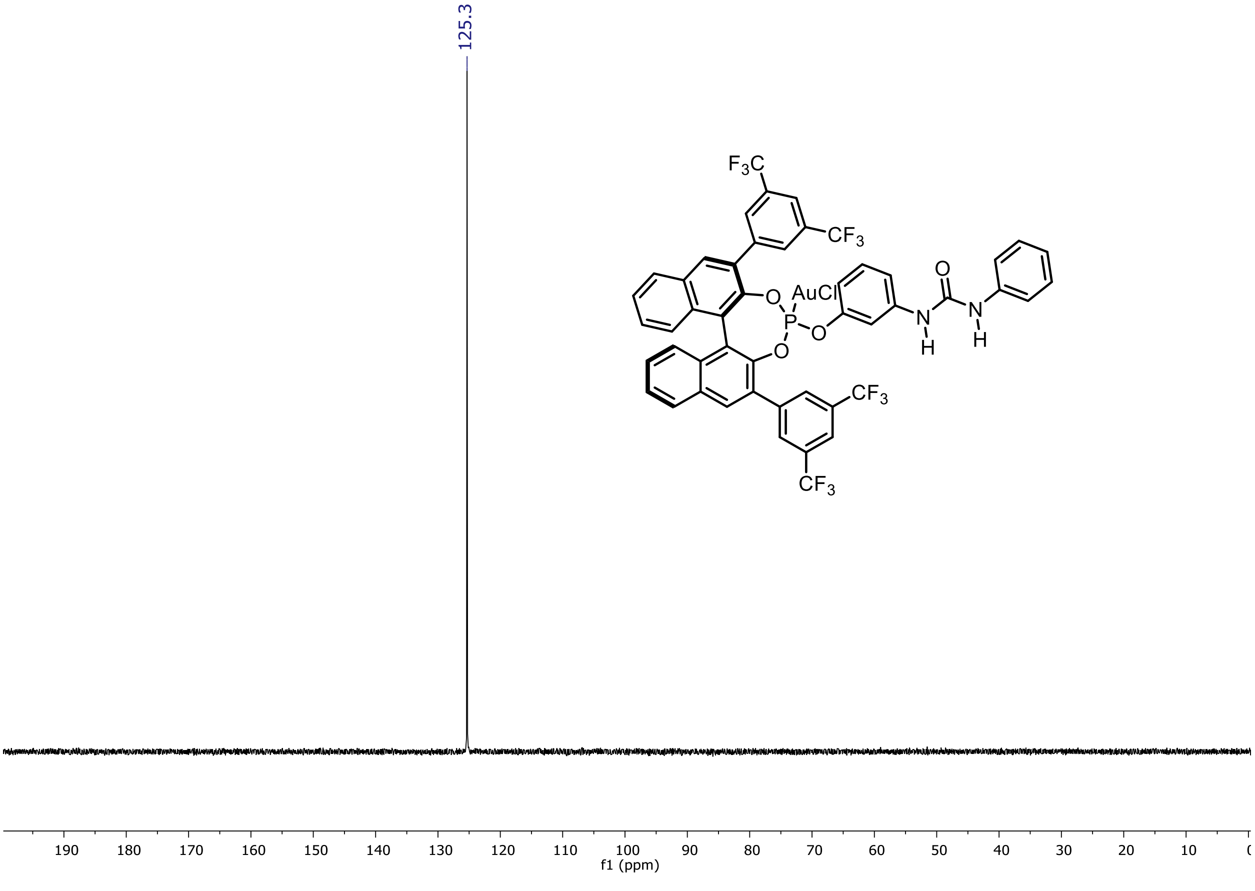

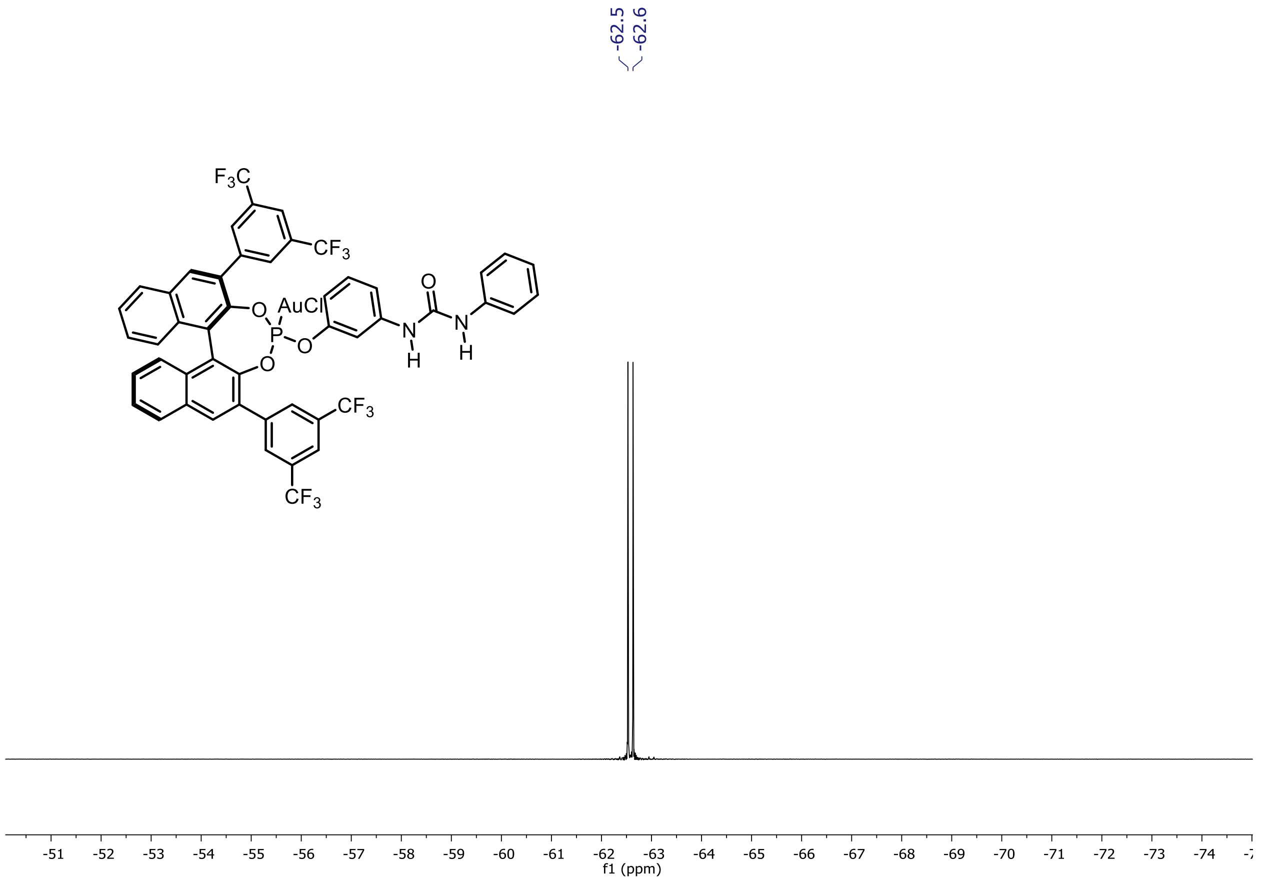
-62.5
-62.6
F3C
CF3
AuCl
O
P
O
O
N
H
N
H
O
CF3
CF3
-51 -52 -53 -54 -55 -56 -57 -58 -59 -60 -61 -62 -63 -64 -65 -66 -67 -68 -69 -70 -71 -72 -73 -74
f1 (ppm)

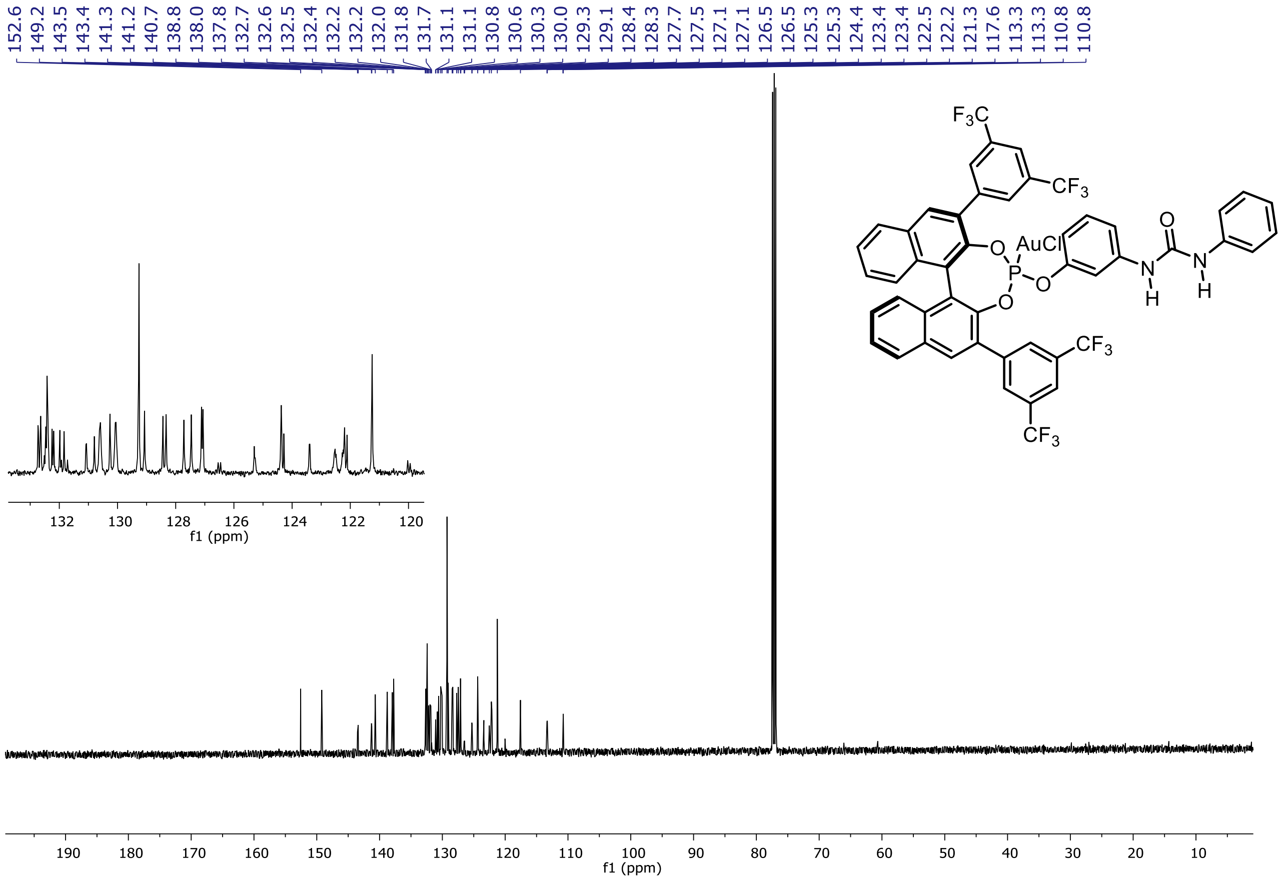
152.6 149.2 143.5 143.4 141.3 141.2 140.7 138.8 138.0 137.8 132.7 132.6 132.5 132.4 132.2 132.2 132.0 131.8 131.7 131.1 131.1 130.8 130.6 130.3 130.0 129.3 129.1 128.4 128.3 127.7 127.5 127.1 127.1 126.5 126.5 125.3 125.3 124.4 123.4 123.4 122.5 122.2 121.3 117.6 113.3 113.3 110.8 110.8
F3C
CF3
AuCl
O
P
O
O
N
H
N
H
O
CF3
CF3
132 130 128 126 124 122 120
f1 (ppm)
190 180 170 160 150 140 130 120 110 100 90 80 70 60 50 40 30 20 10
f1 (ppm)

Compound **(*R*)-Au8** ^{1}H, ^{31}P{^{1}H}, and ^{13}C{^{1}H} NMR spectra in CDCl$_3$

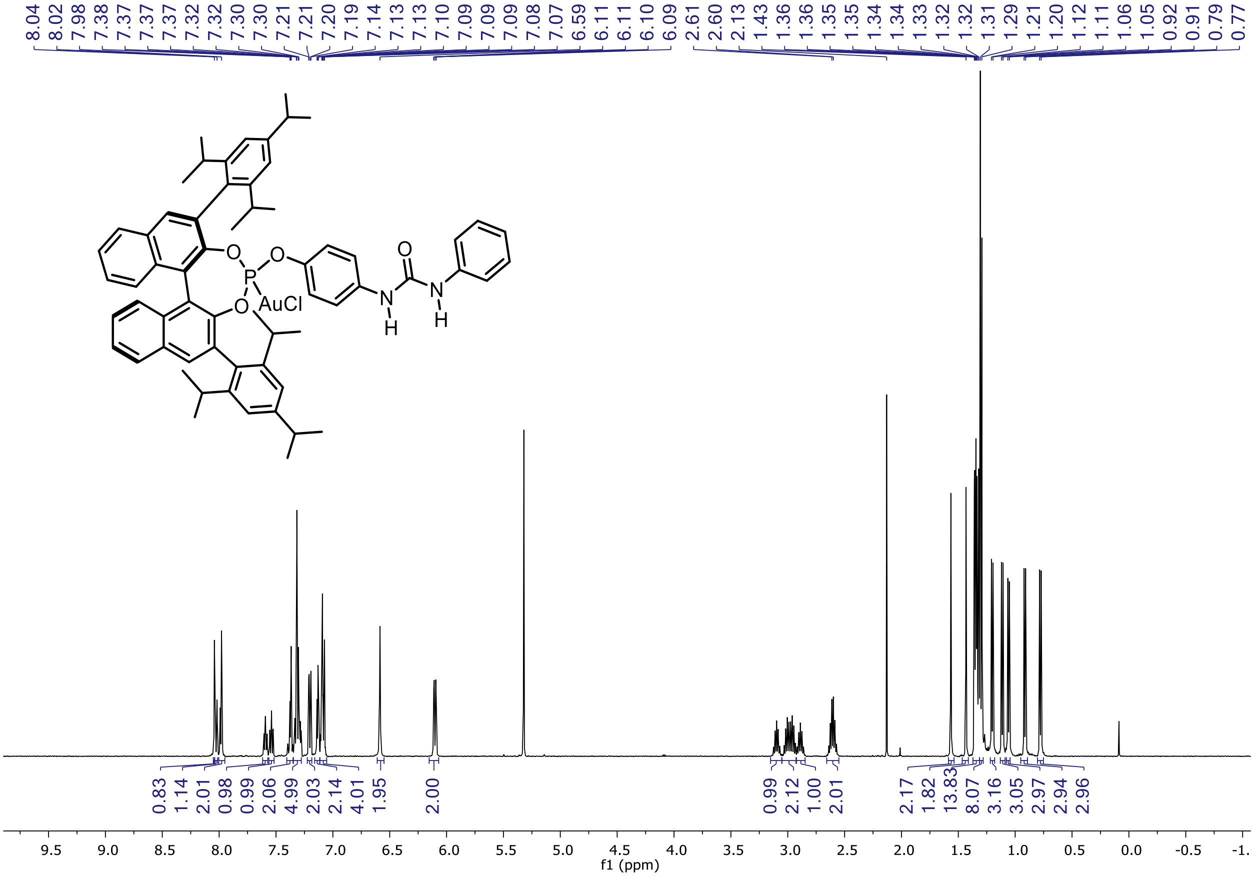

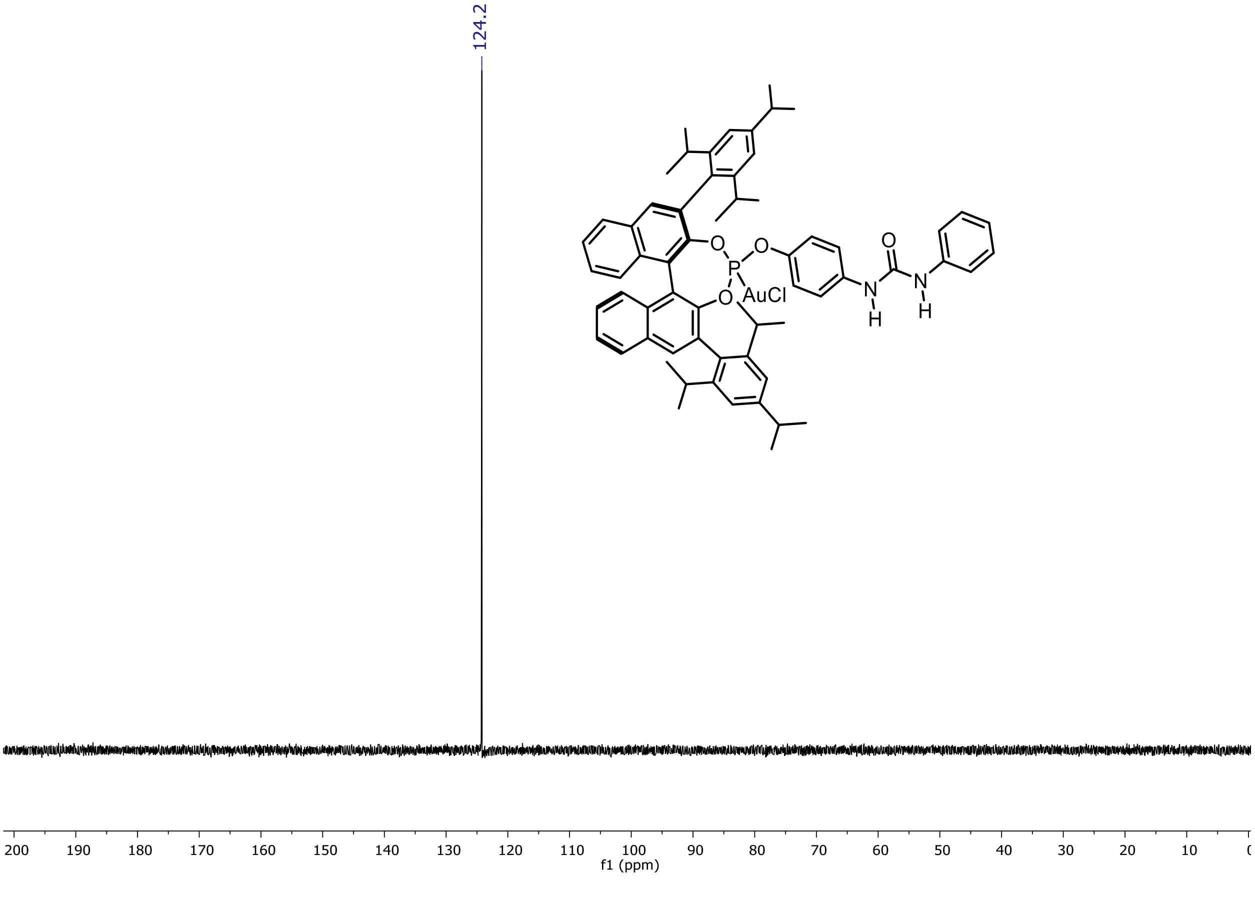

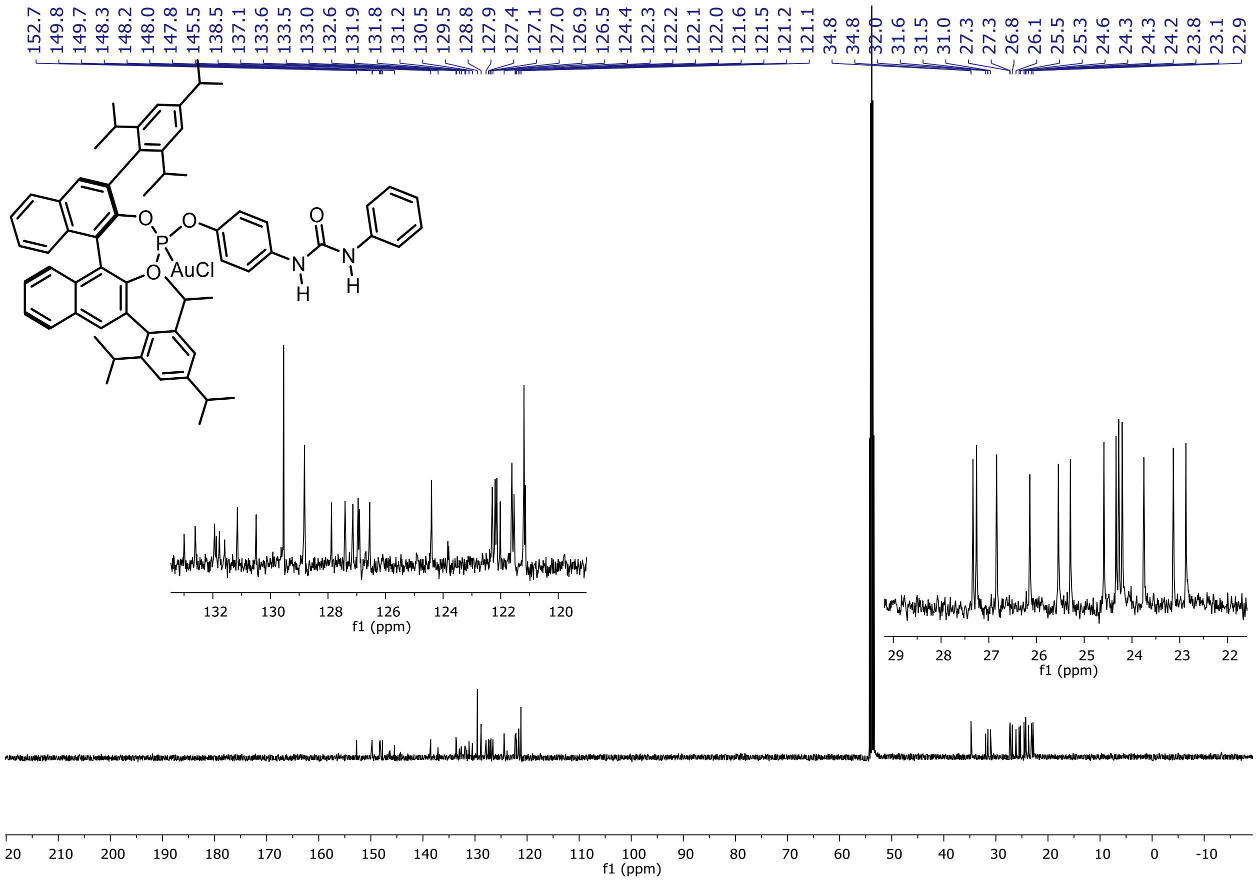
152.7
149.8
149.7
148.3
148.2
148.0
147.8
145.5
138.5
137.1
133.6
133.5
133.0
132.6
131.9
131.8
131.2
130.5
129.5
128.8
127.9
127.4
127.1
127.0
126.9
126.5
124.4
122.3
122.2
122.1
122.0
121.6
121.5
121.2
121.1
34.8
34.8
32.0
31.6
31.5
31.0
27.3
27.3
26.8
26.1
25.5
25.3
24.6
24.3
24.3
24.2
23.8
23.1
22.9
AuCl
f1 (ppm)
f1 (ppm)
f1 (ppm)

Compound **Au9** ^{1}H, $^{31}P\{^{1}H\}$, and $^{13}C\{^{1}H\}$ NMR spectra in $CDCl_3$

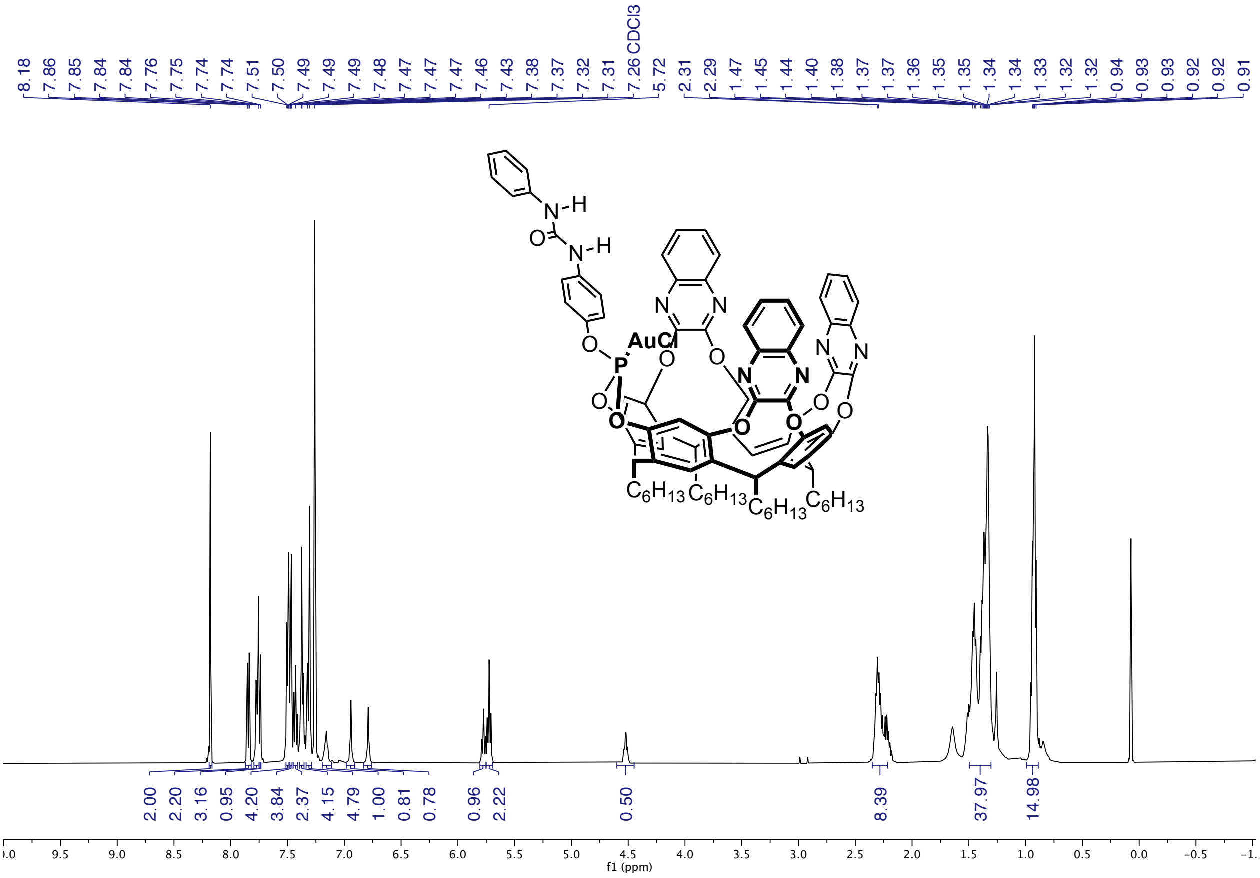

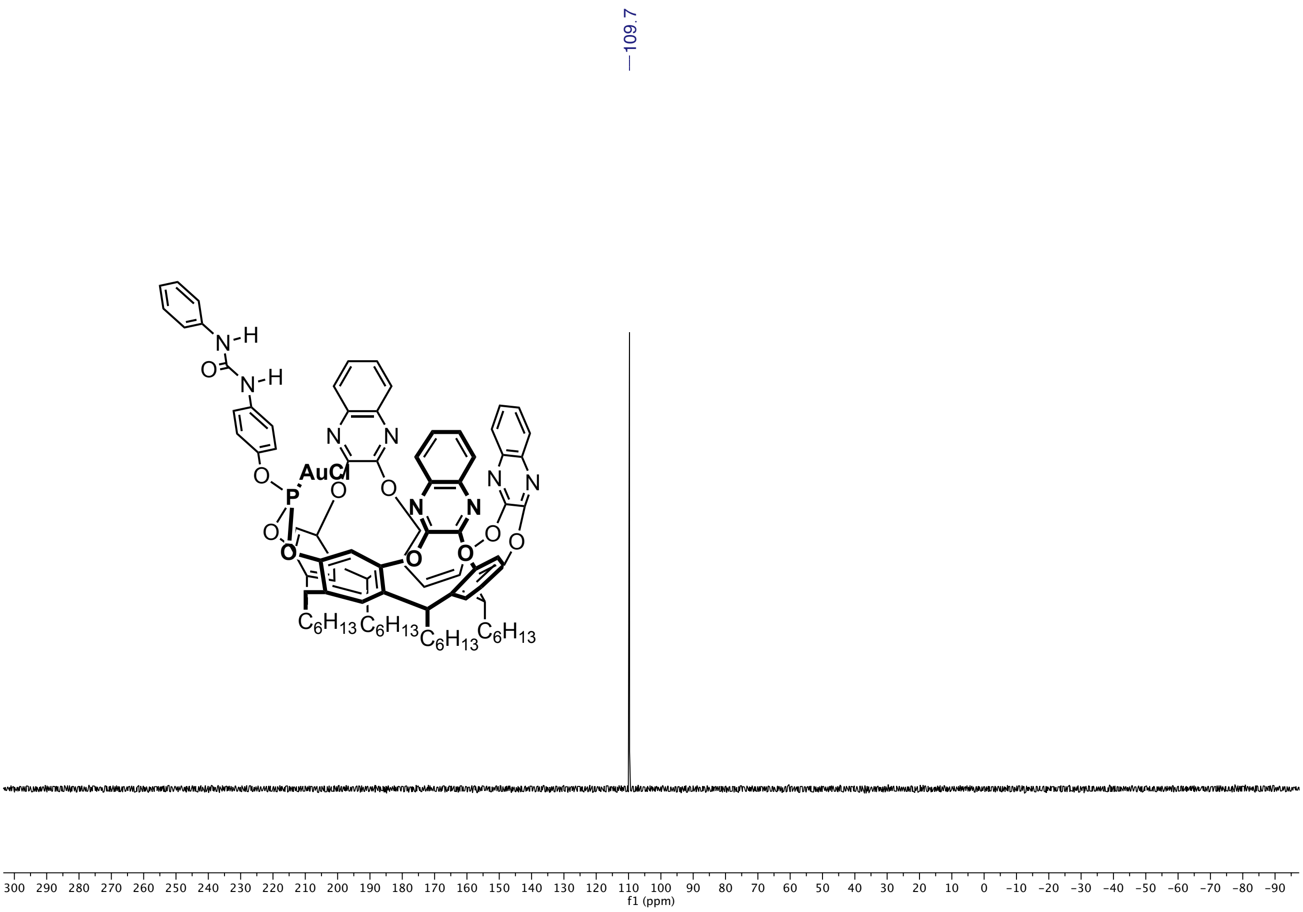

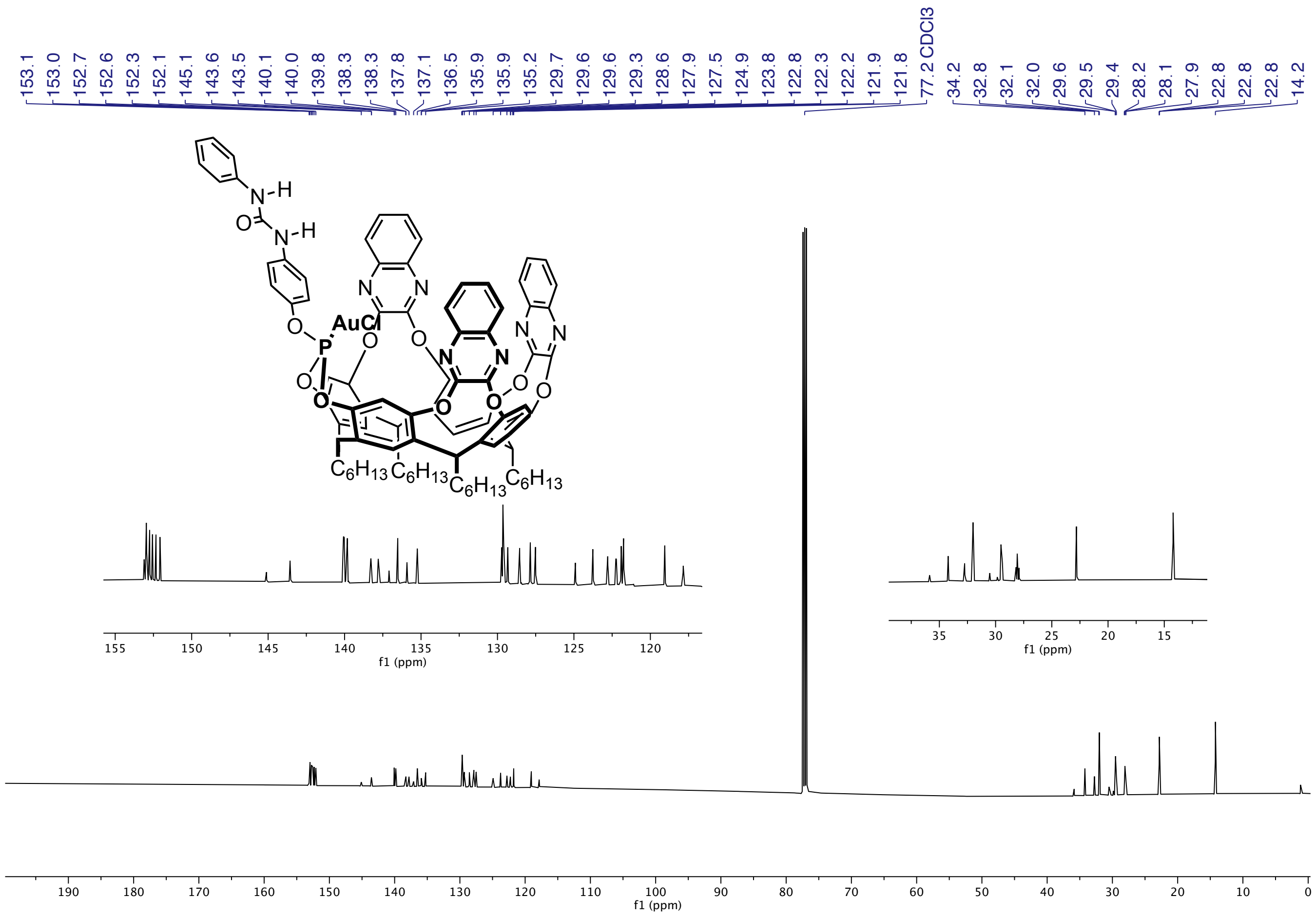

153.1
153.0
152.7
152.6
152.3
152.1
145.1
143.6
143.5
140.1
140.0
139.8
138.3
138.3
137.8
137.1
136.5
135.9
135.9
135.2
129.7
129.6
129.6
129.3
128.6
127.9
127.5
124.9
123.8
122.8
122.3
122.2
121.9
121.8
77.2 CDCl3
34.2
32.8
32.1
32.0
29.6
29.5
29.4
28.2
28.1
27.9
22.8
22.8
22.8
14.2
AuCl
P
N-H
O
N
C6H13
f1 (ppm)

Compound **Au10** ^{1}H, $^{31}P\{^{1}H\}$, and $^{13}C\{^{1}H\}$ NMR spectra in $CDCl_3$

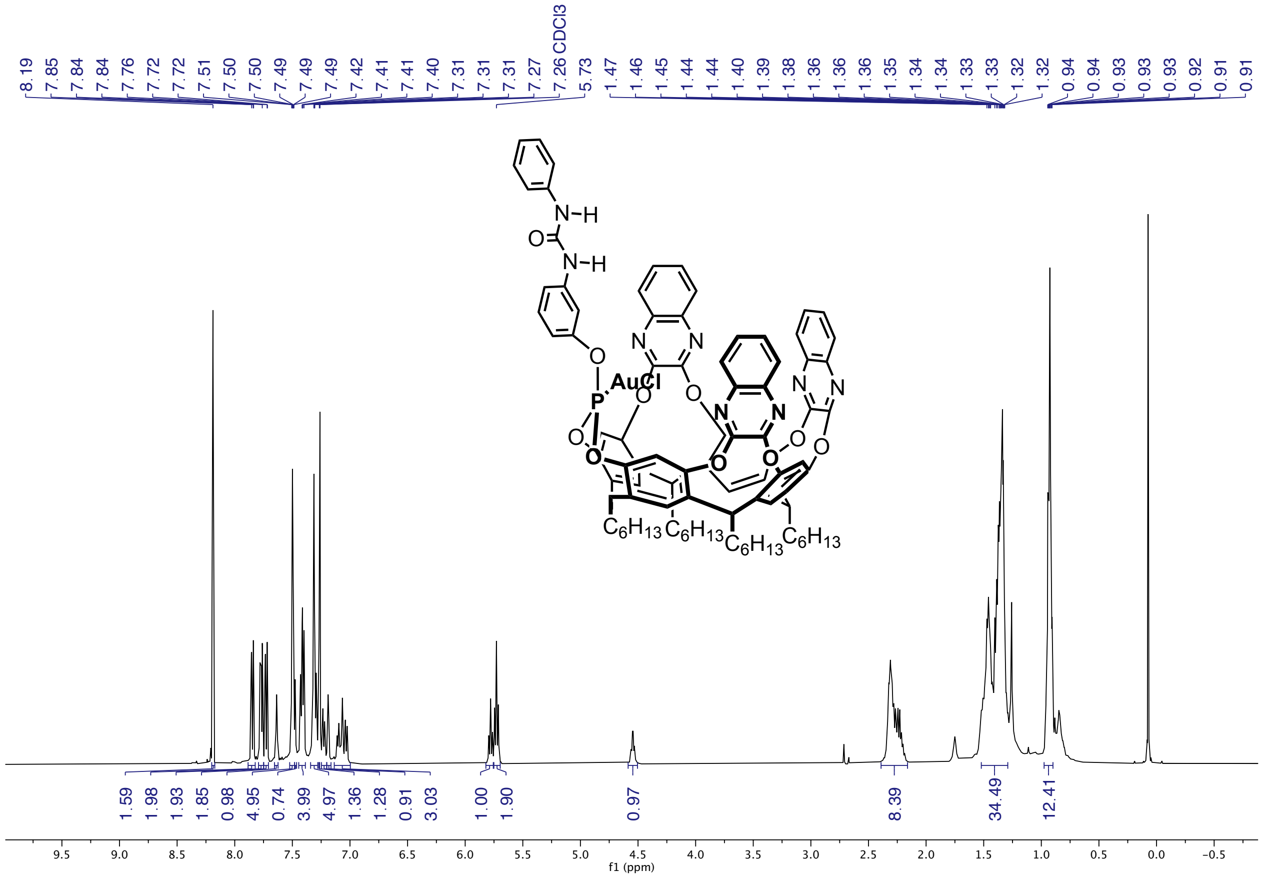

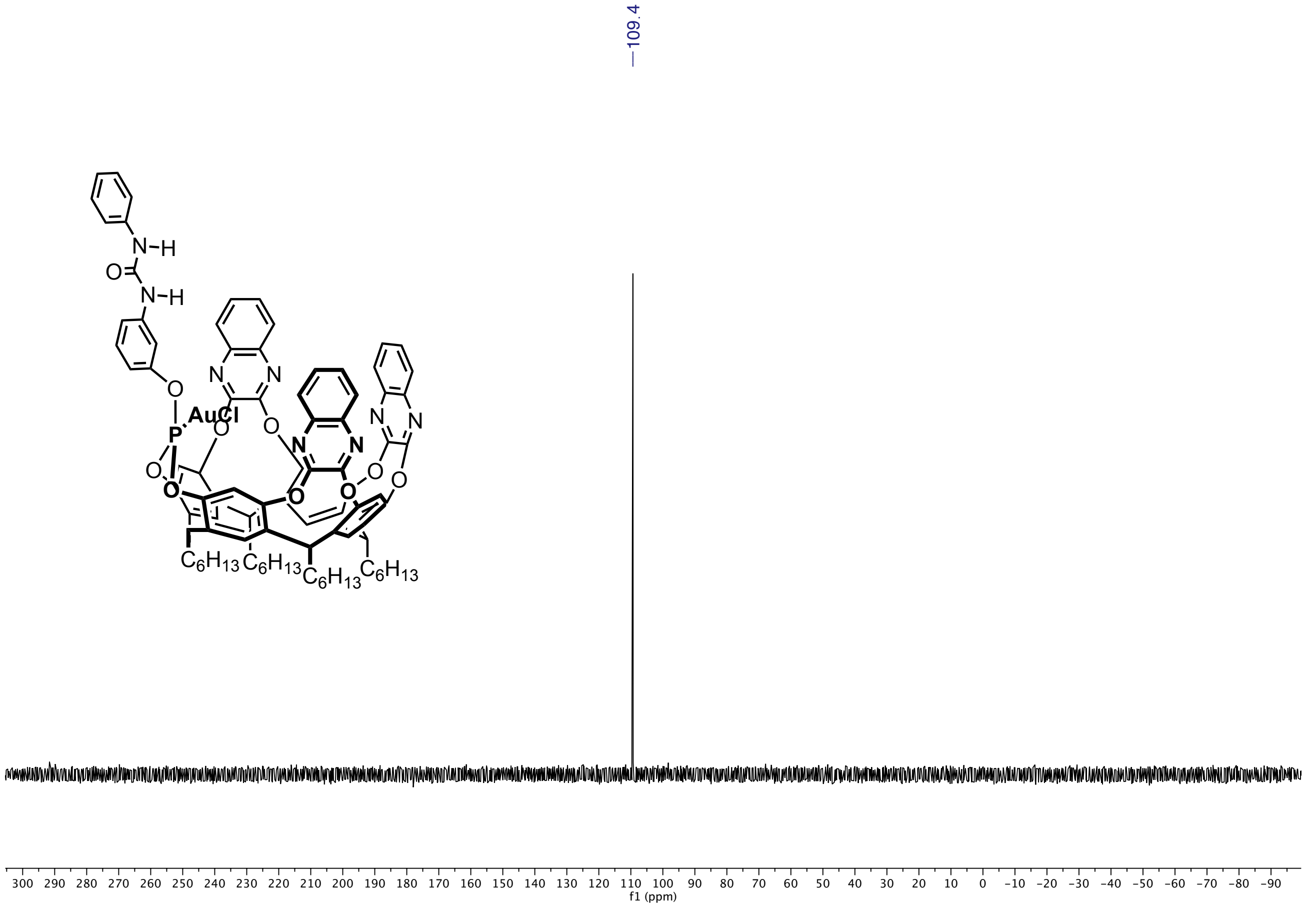

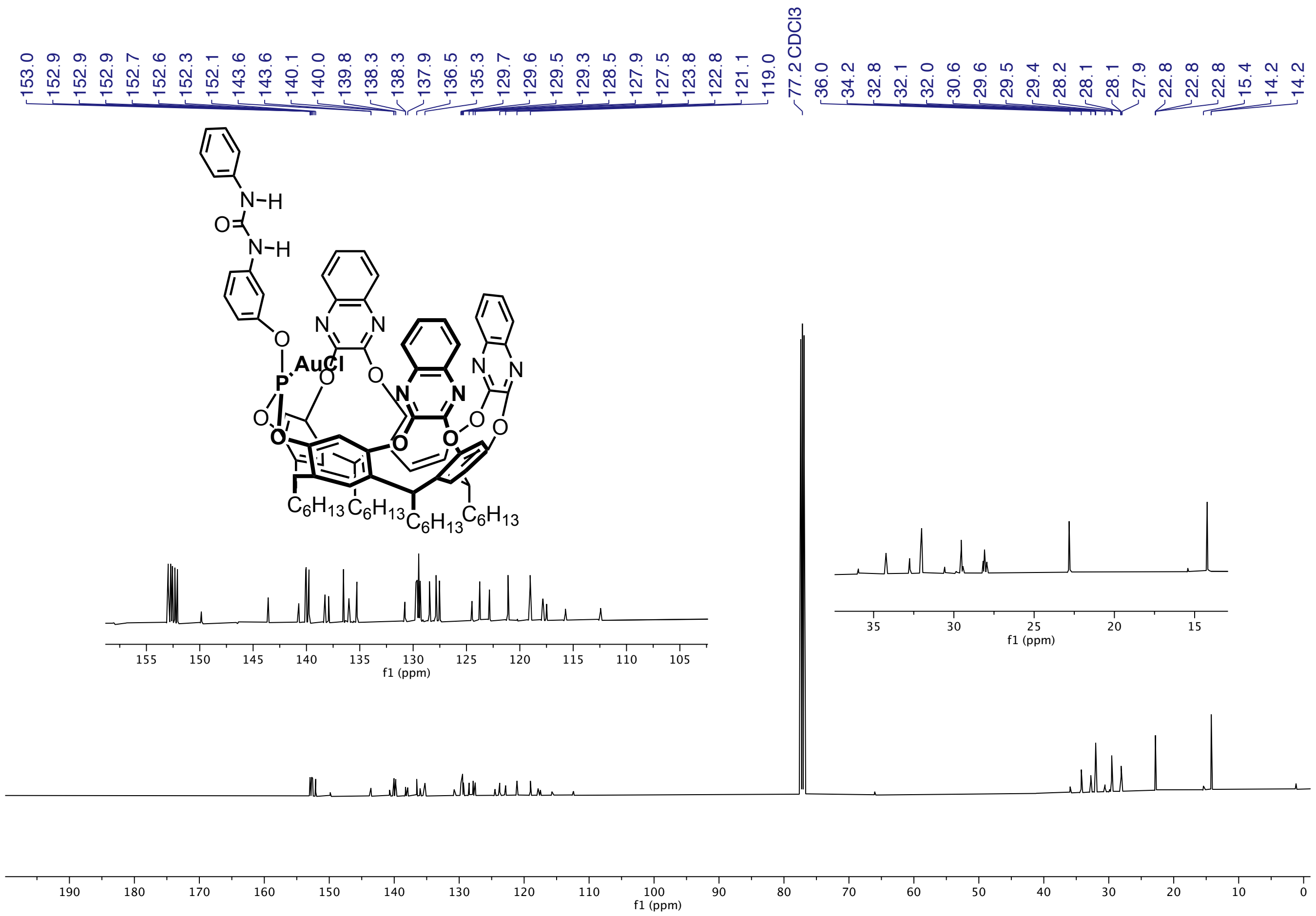
153.0
152.9
152.9
152.9
152.7
152.6
152.3
152.1
143.6
143.6
140.1
140.0
139.8
138.3
138.3
137.9
136.5
135.3
129.7
129.6
129.5
129.3
128.5
127.9
127.5
123.8
122.8
121.1
119.0
77.2 CDCl3
36.0
34.2
32.8
32.1
32.0
30.6
29.6
29.5
29.4
28.2
28.1
28.1
27.9
22.8
22.8
22.8
15.4
14.2
14.2
N-H
O
N-H
O
AuCl
P
N
N
N
N
O
O
O
O
O
O
O
O
O
N
N
O
C_6H_{13} C_6H_{13} C_6H_{13} C_6H_{13}
f1 (ppm)
f1 (ppm)
f1 (ppm)

Compound **Au11** ^{1}H, $^{31}P\{^{1}H\}$, and $^{13}C\{^{1}H\}$ NMR spectra in $CDCl_3$

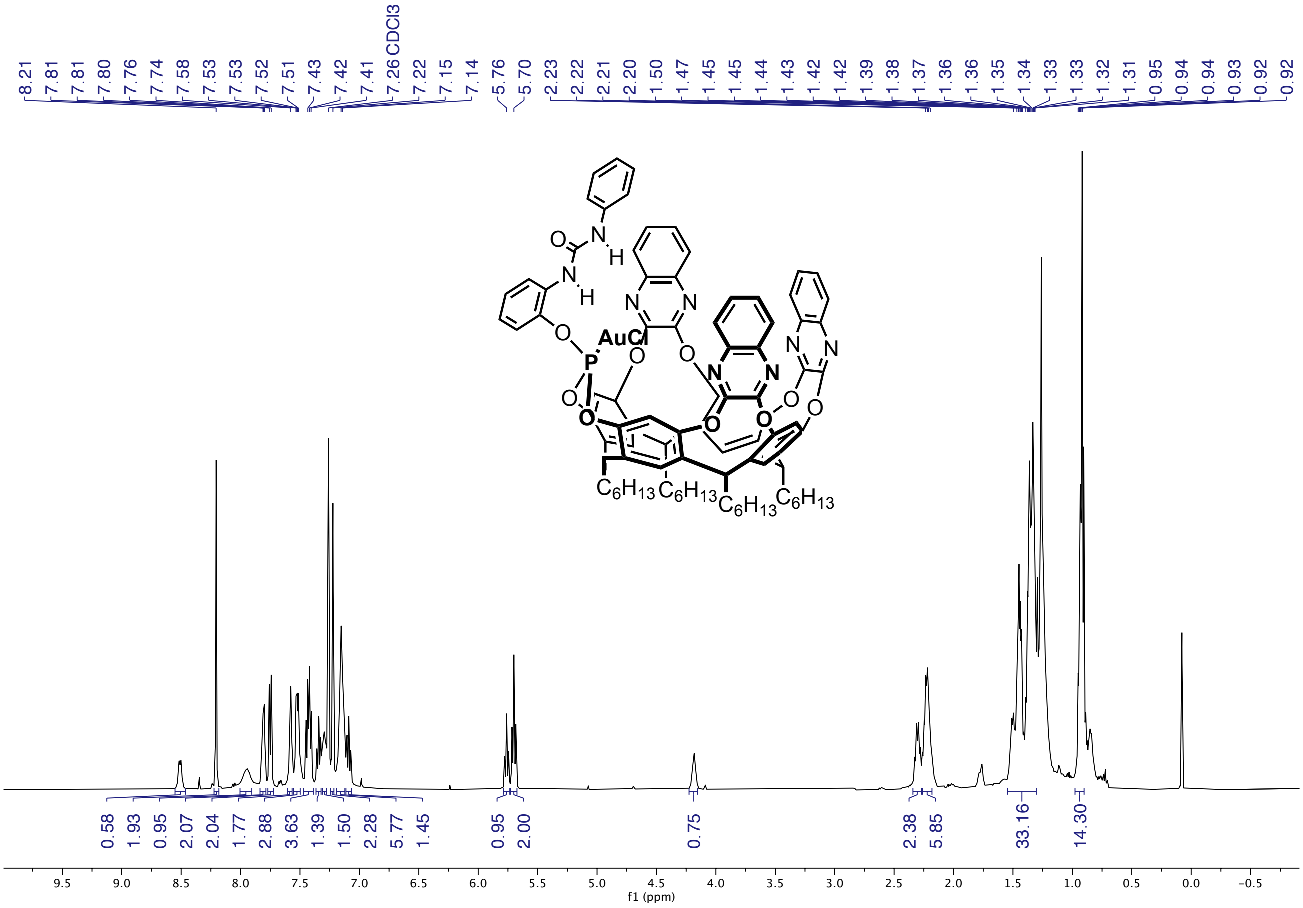

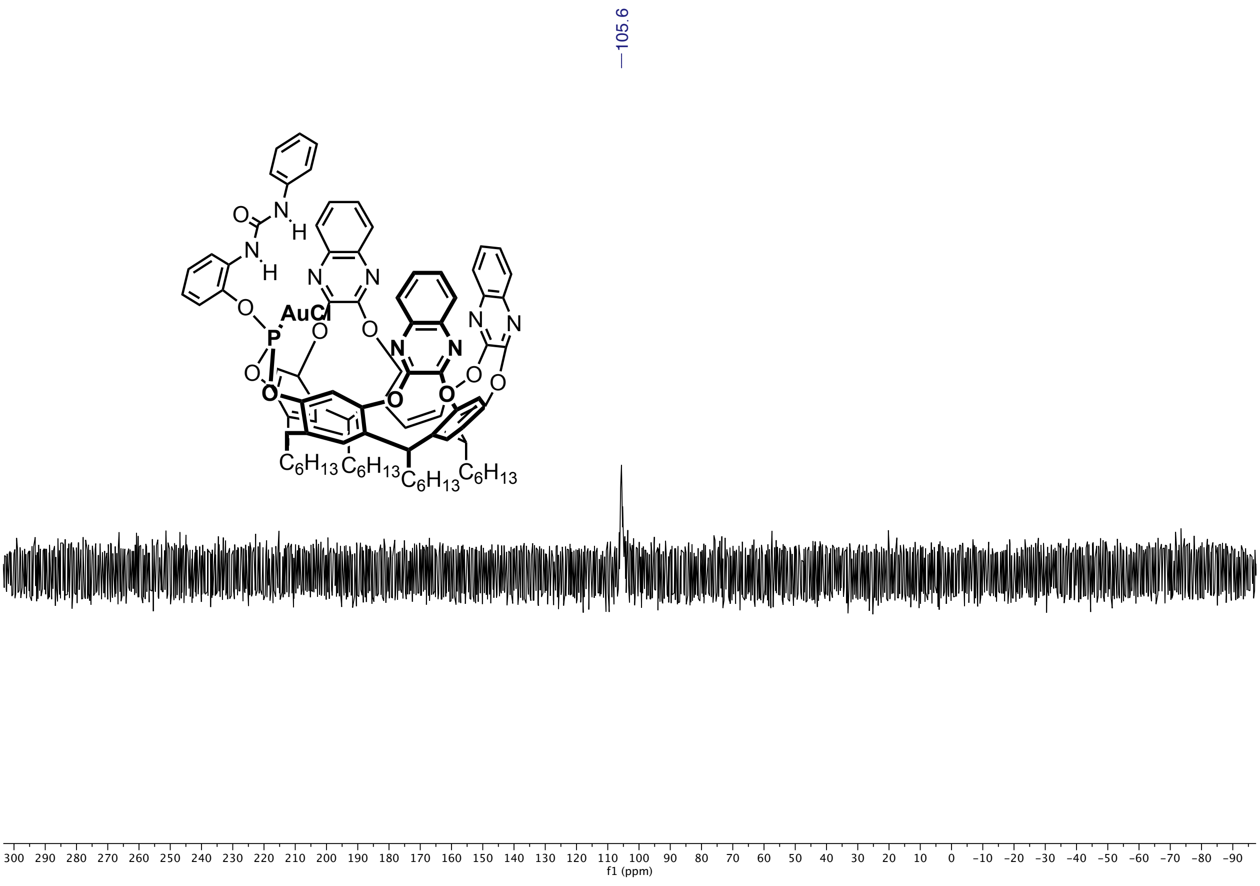

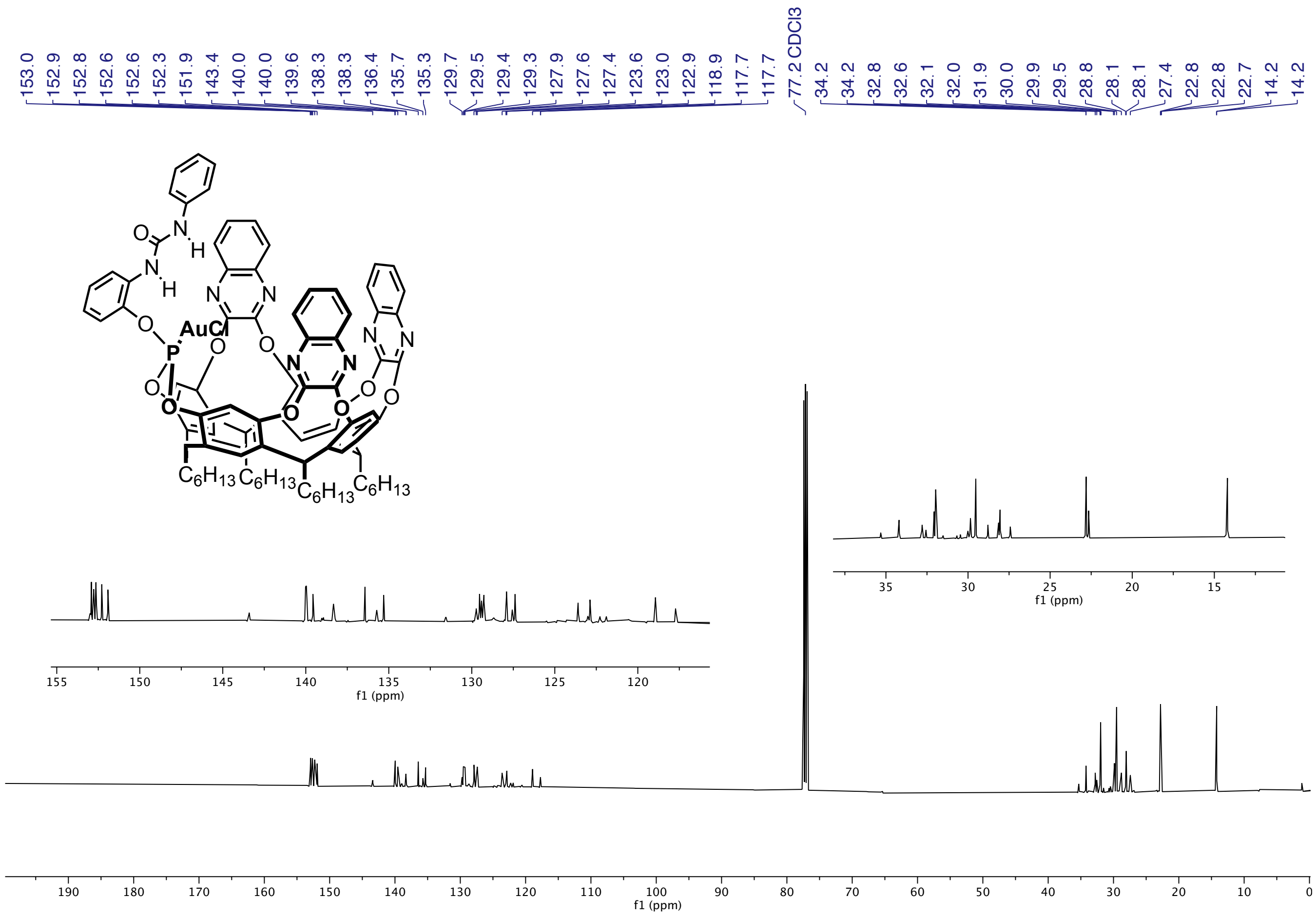

153.0
152.9
152.8
152.6
152.6
152.3
151.9
143.4
140.0
140.0
139.6
138.3
138.3
136.4
135.7
135.3
129.7
129.5
129.4
129.3
127.9
127.6
127.4
123.6
123.0
122.9
118.9
117.7
117.7
77.2 CDCl3
34.2
34.2
32.8
32.6
32.1
32.0
31.9
30.0
29.9
29.5
28.8
28.1
28.1
27.4
22.8
22.8
22.7
14.2
14.2
AuCl
f1 (ppm)
f1 (ppm)
f1 (ppm)

Compound **1a** ^{1}H and ^{13}C{^{1}H} NMR spectra in $CDCl_3$

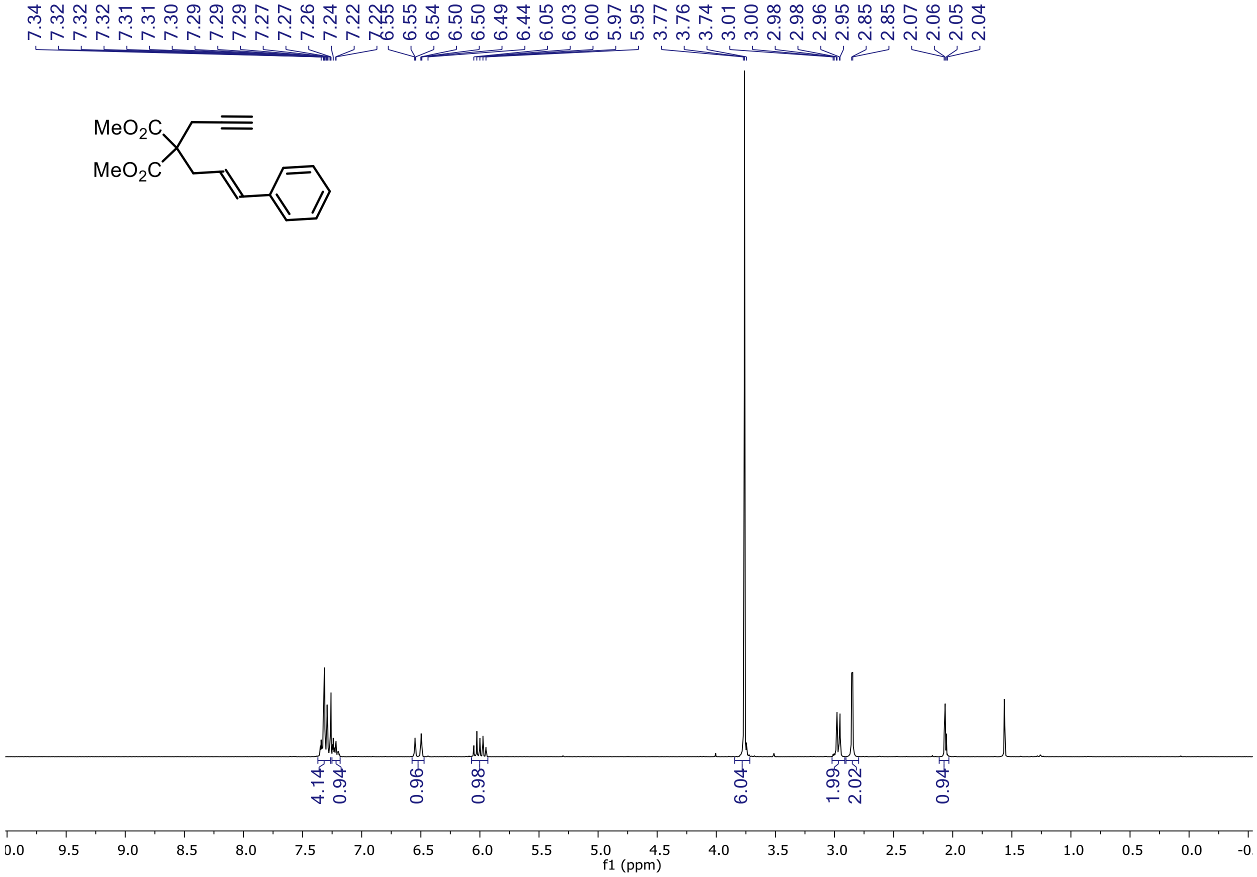

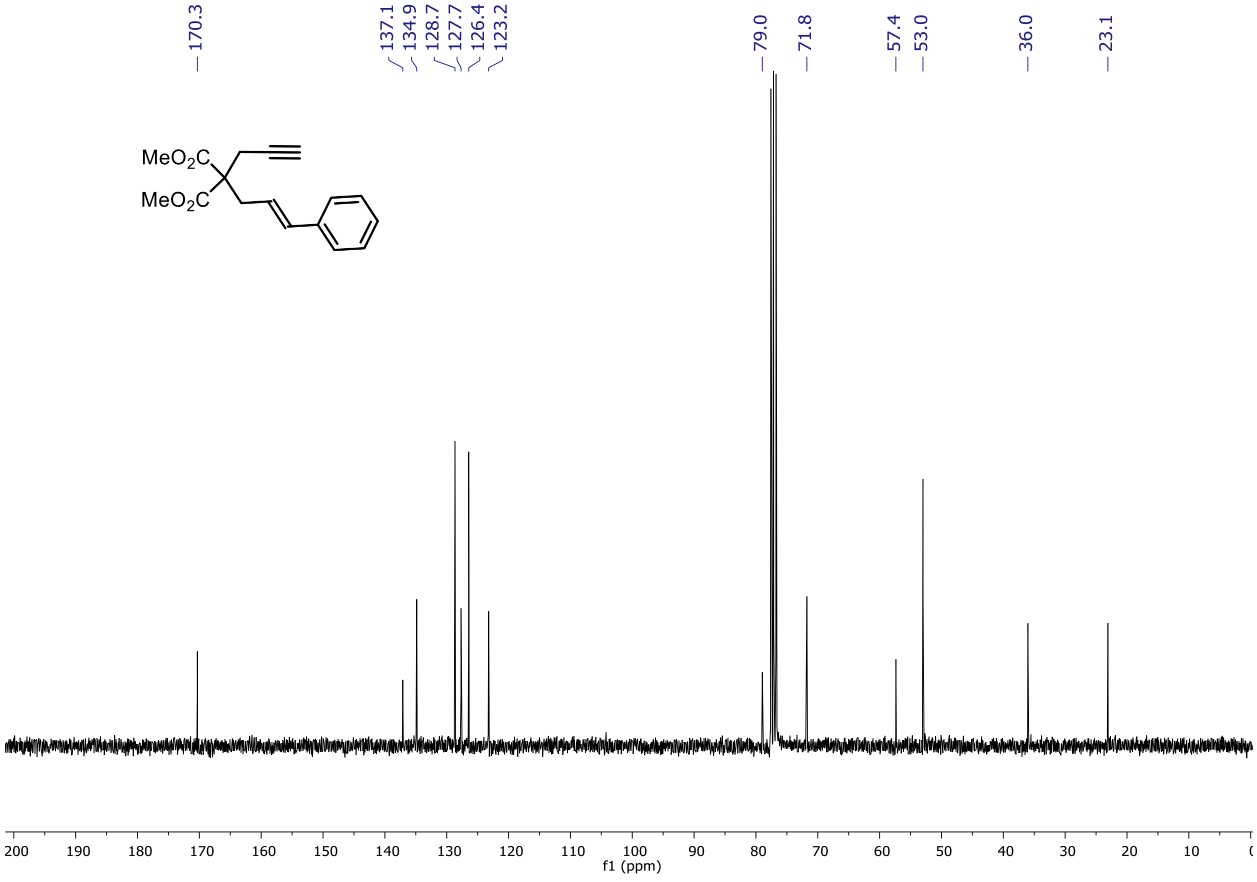

Compound **1b** ^{1}H and ^{13}C{^{1}H} NMR spectra in $CDCl_3$

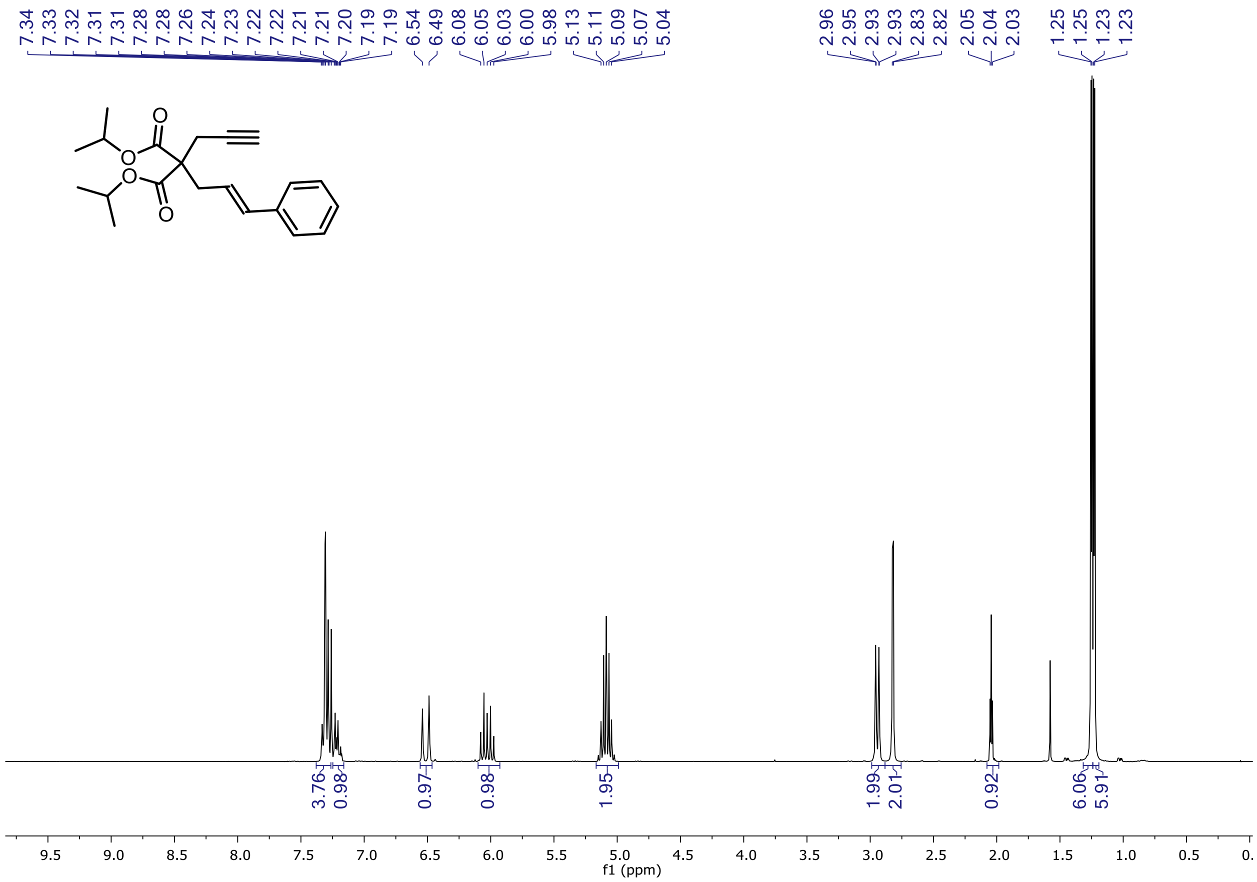

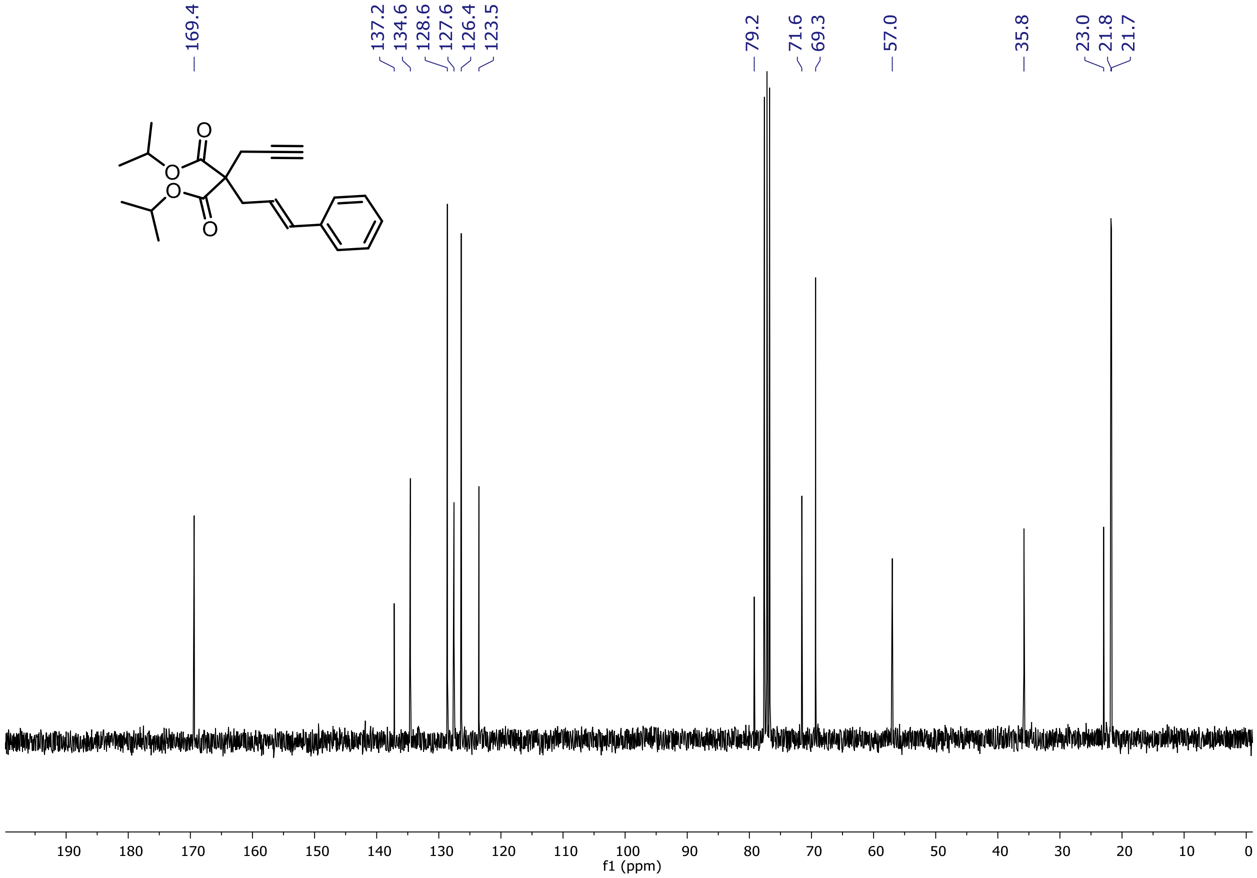

Compound **1c** ^{1}H and $^{13}C\{^{1}H\}$ NMR spectra in $CDCl_3$

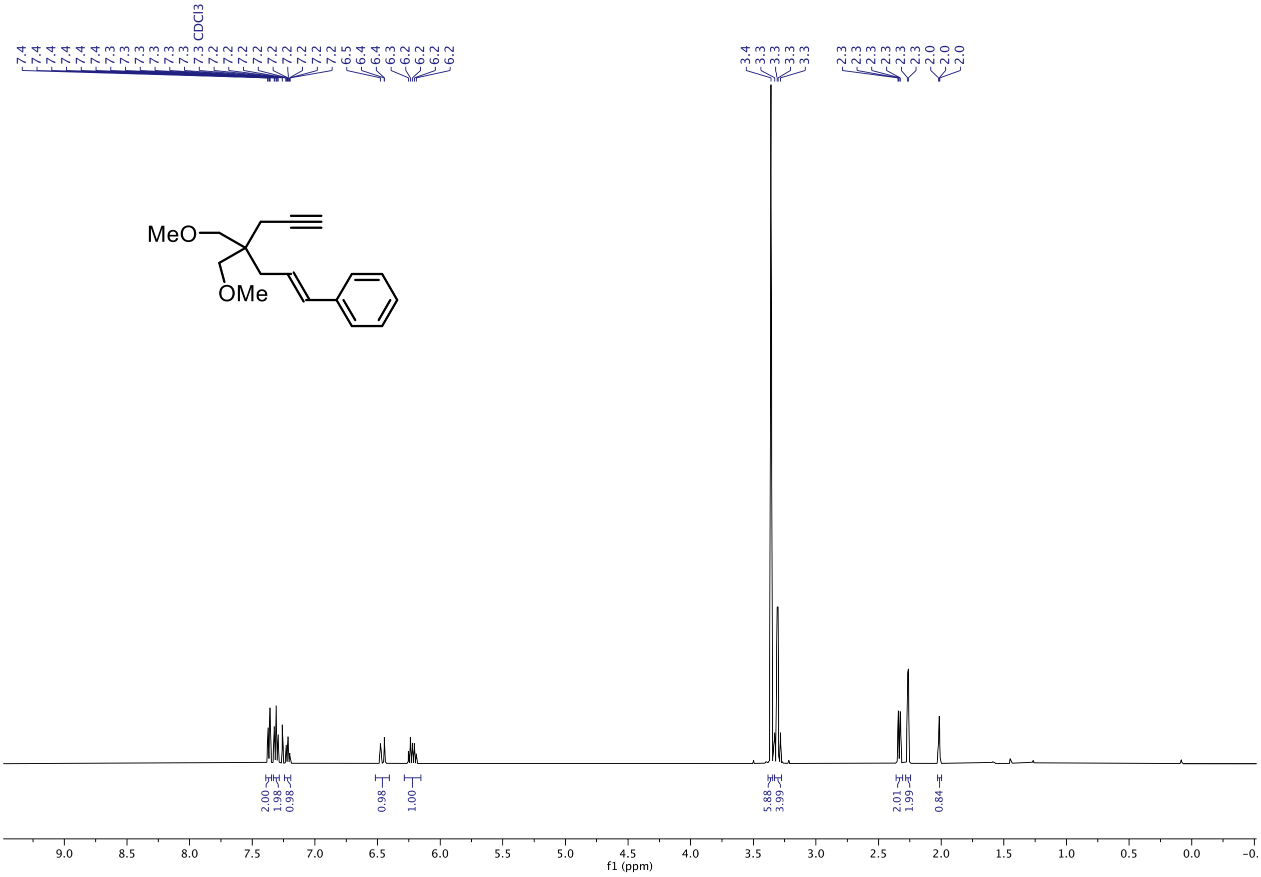

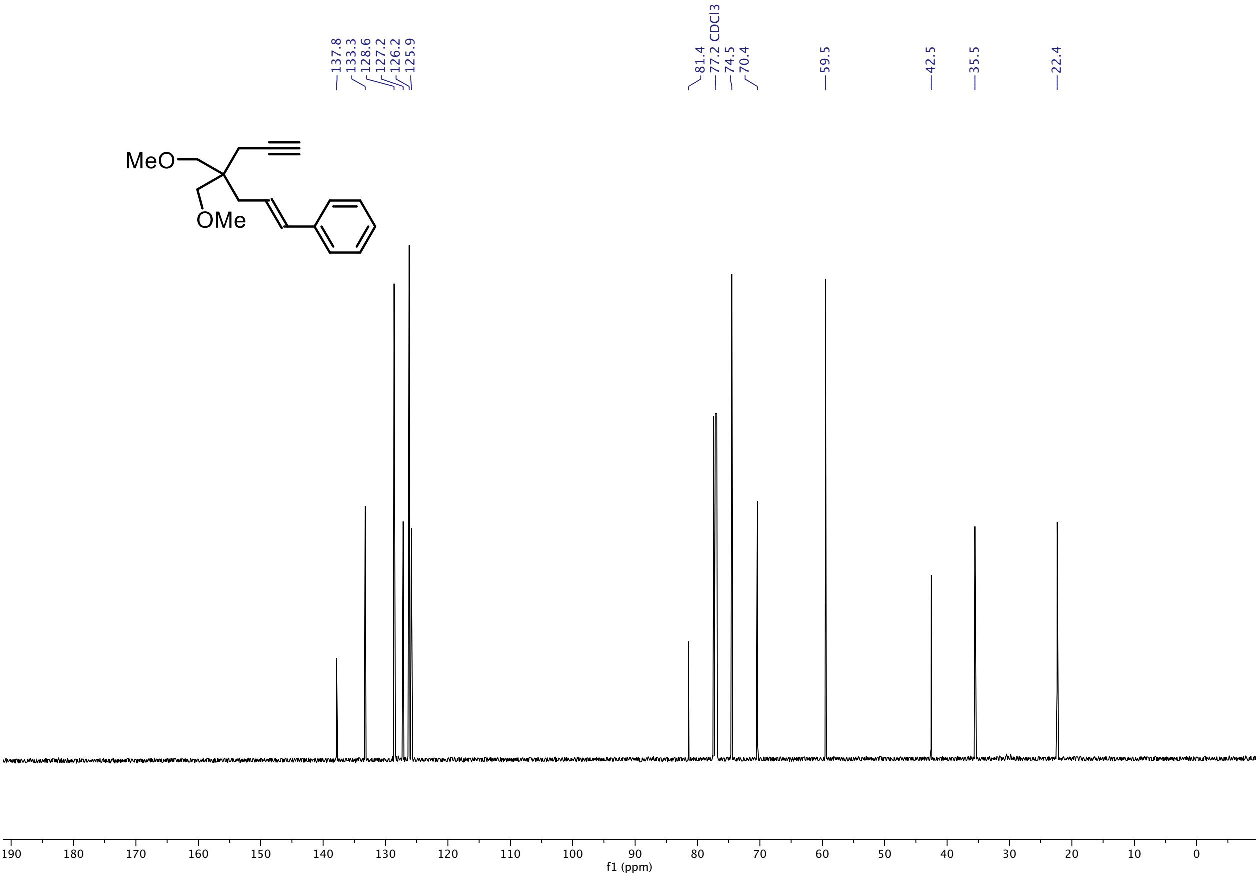

Compound **1d** ^{1}H and ^{13}C{^{1}H} NMR spectra in $CDCl_3$

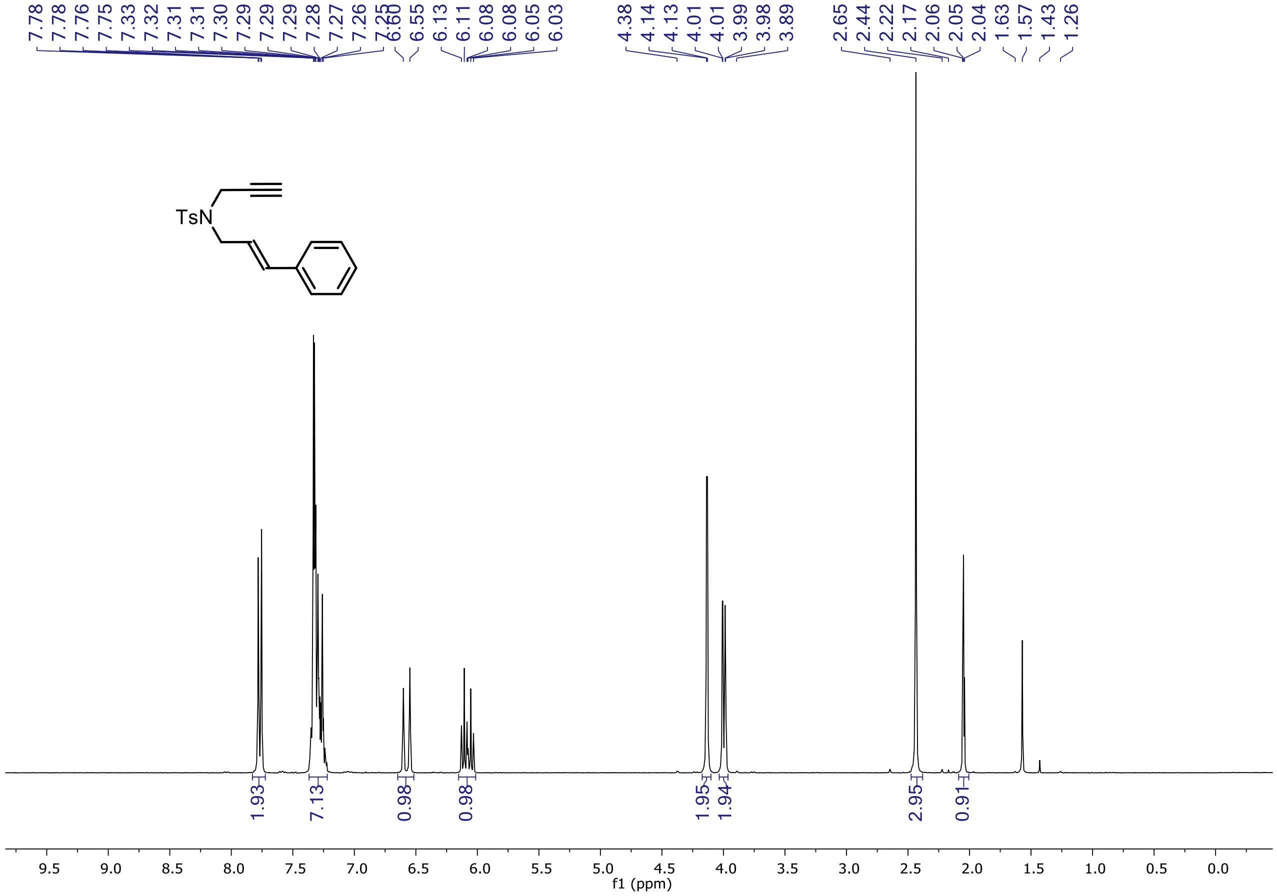

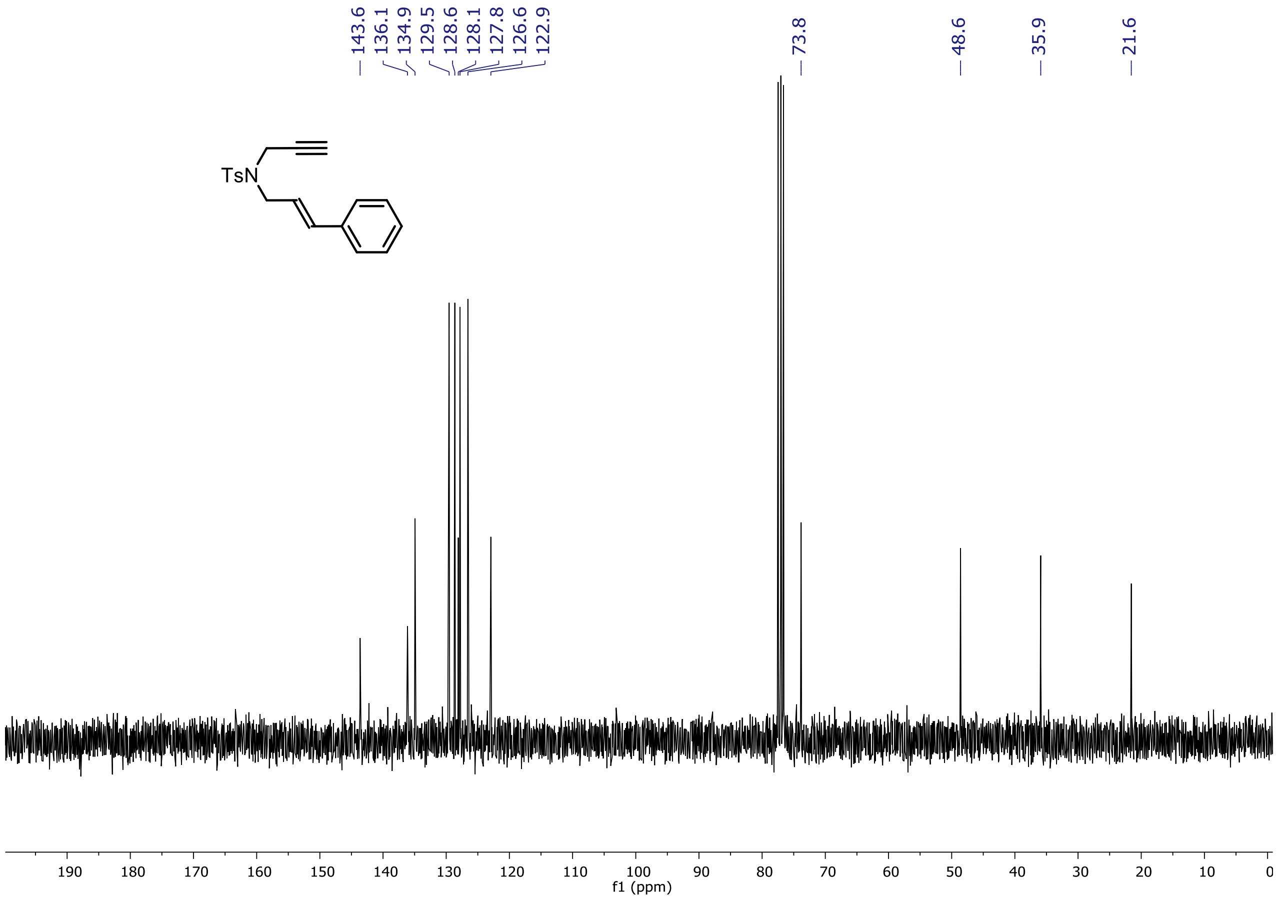

Compound **1e** ^{1}H, ^{19}F and $^{13}C\{^{1}H\}$ NMR spectra in $CDCl_3$

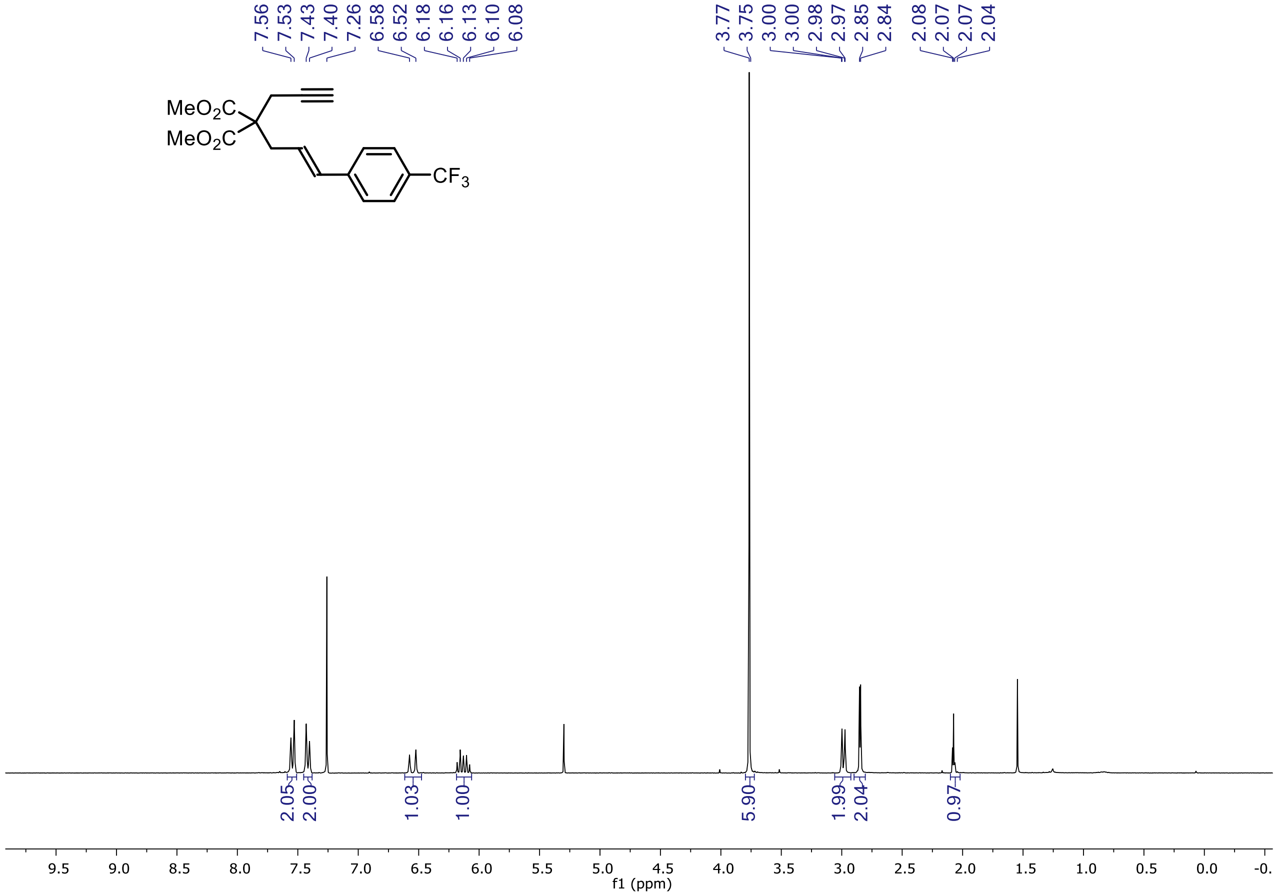

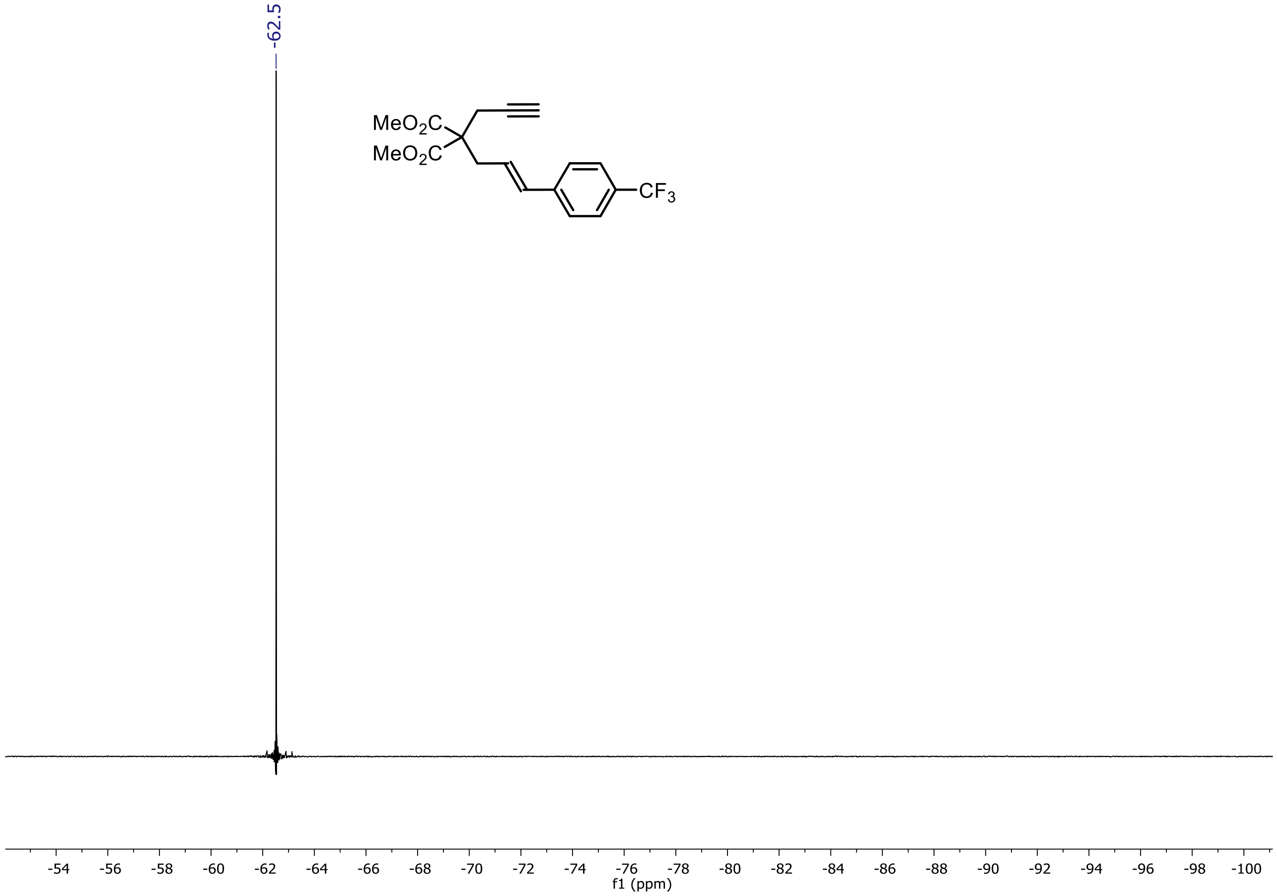

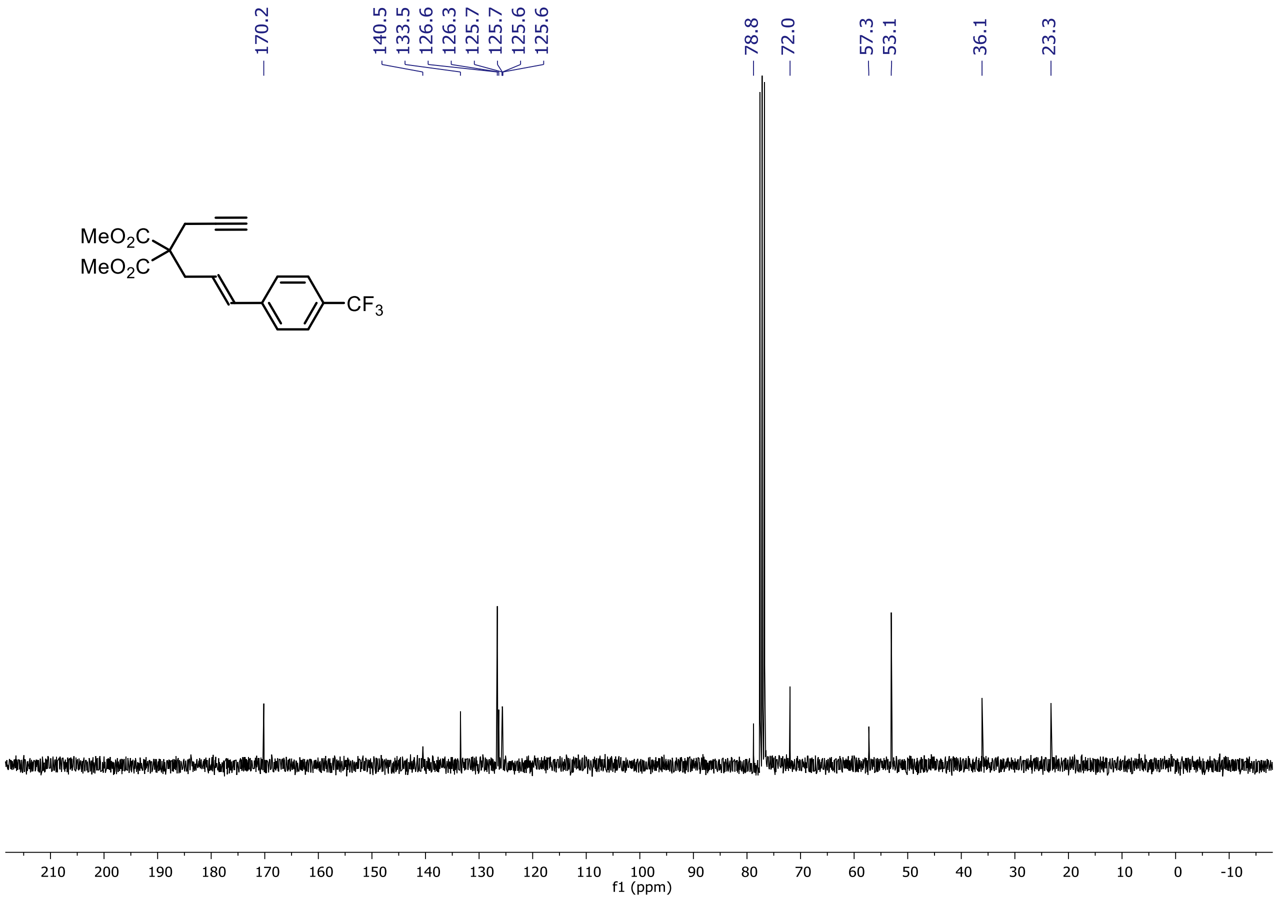

170.2
140.5
133.5
126.6
126.3
125.7
125.7
125.6
125.6
78.8
72.0
57.3
53.1
36.1
23.3
MeO2C
MeO2C
CF3
210 200 190 180 170 160 150 140 130 120 110 100 90 80 70 60 50 40 30 20 10 0 -10
f1 (ppm)

Compound **1f** ^{1}H and ^{13}C{^{1}H} NMR spectra in $CDCl_3$

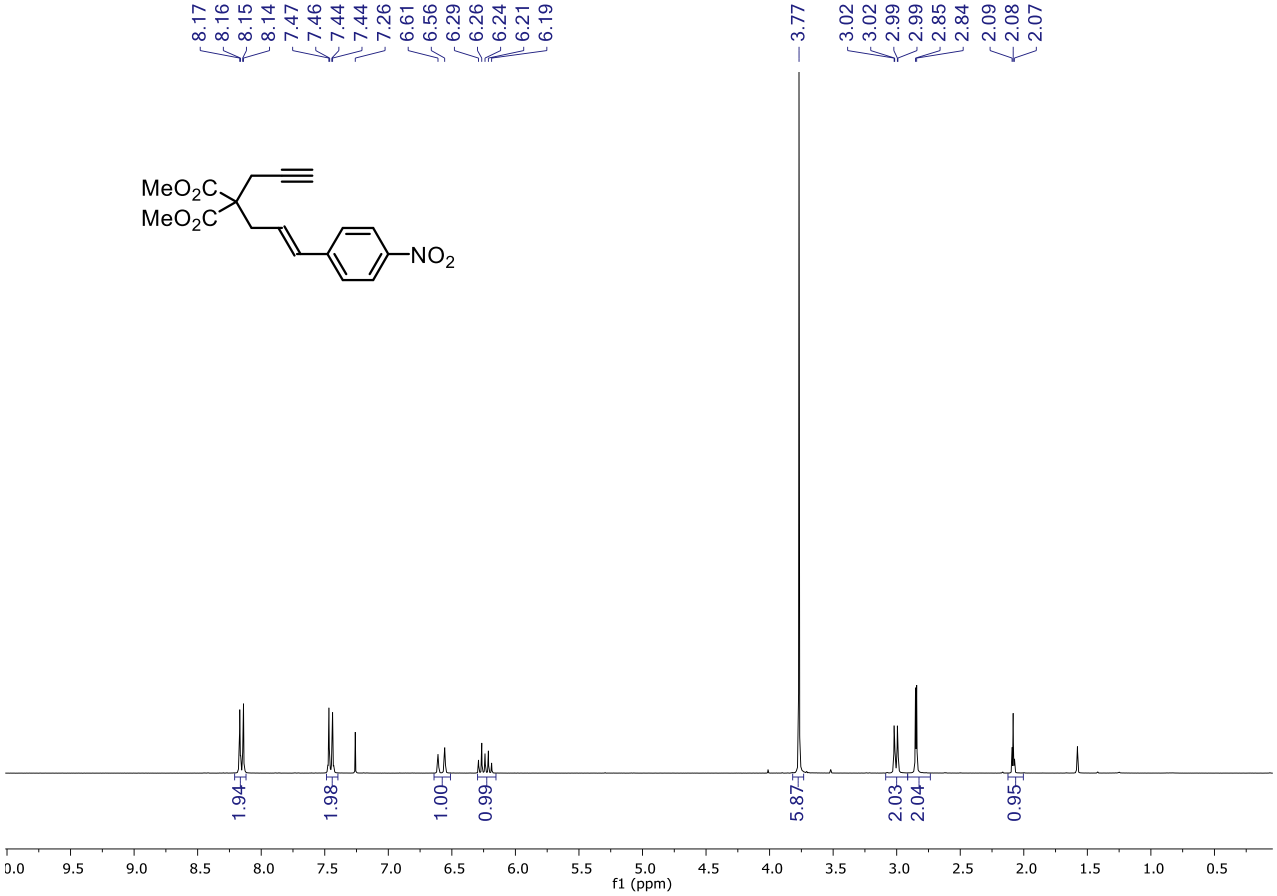

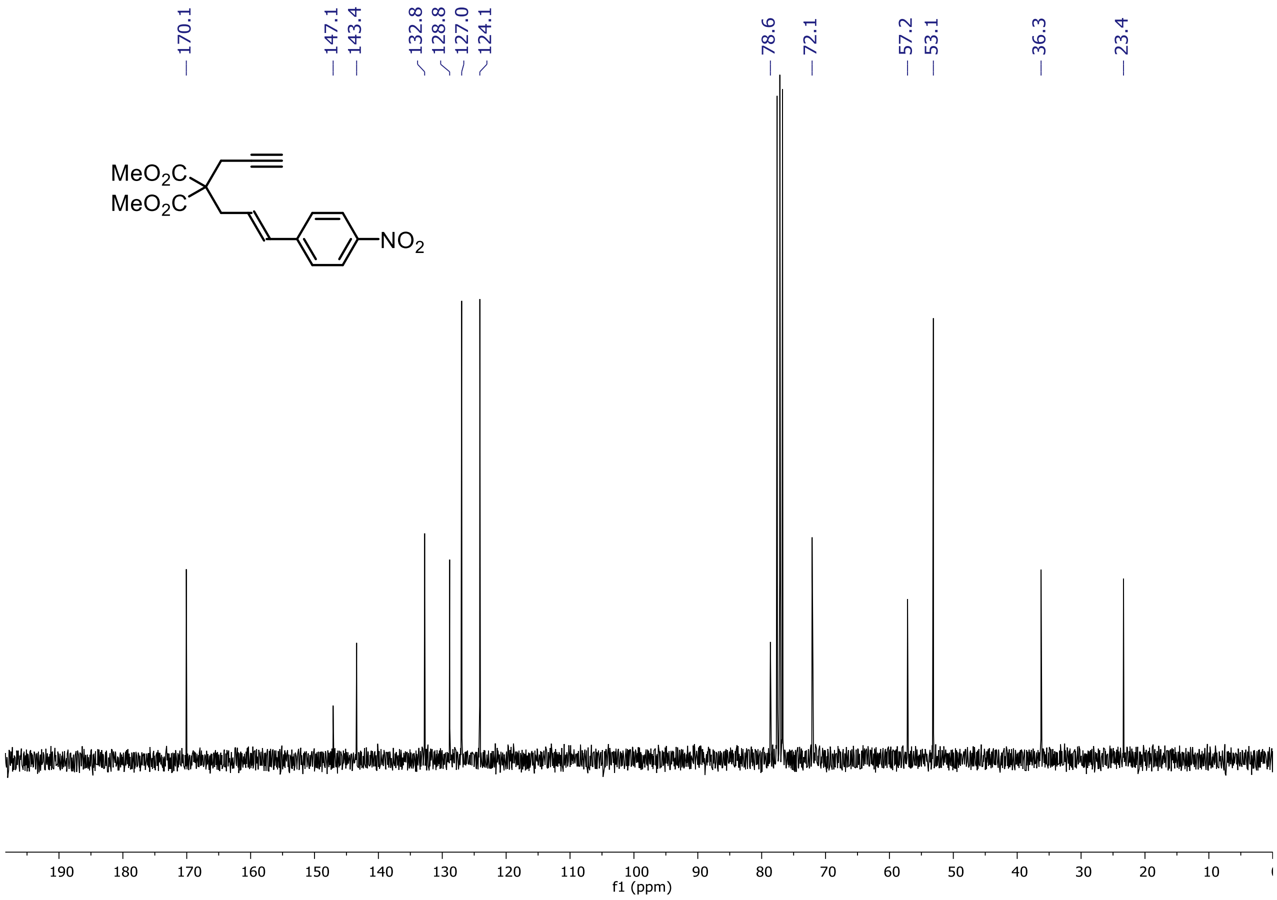

Compound **1g** ^{1}H, and ^{13}C{^{1}H} NMR spectra in $CDCl_3$

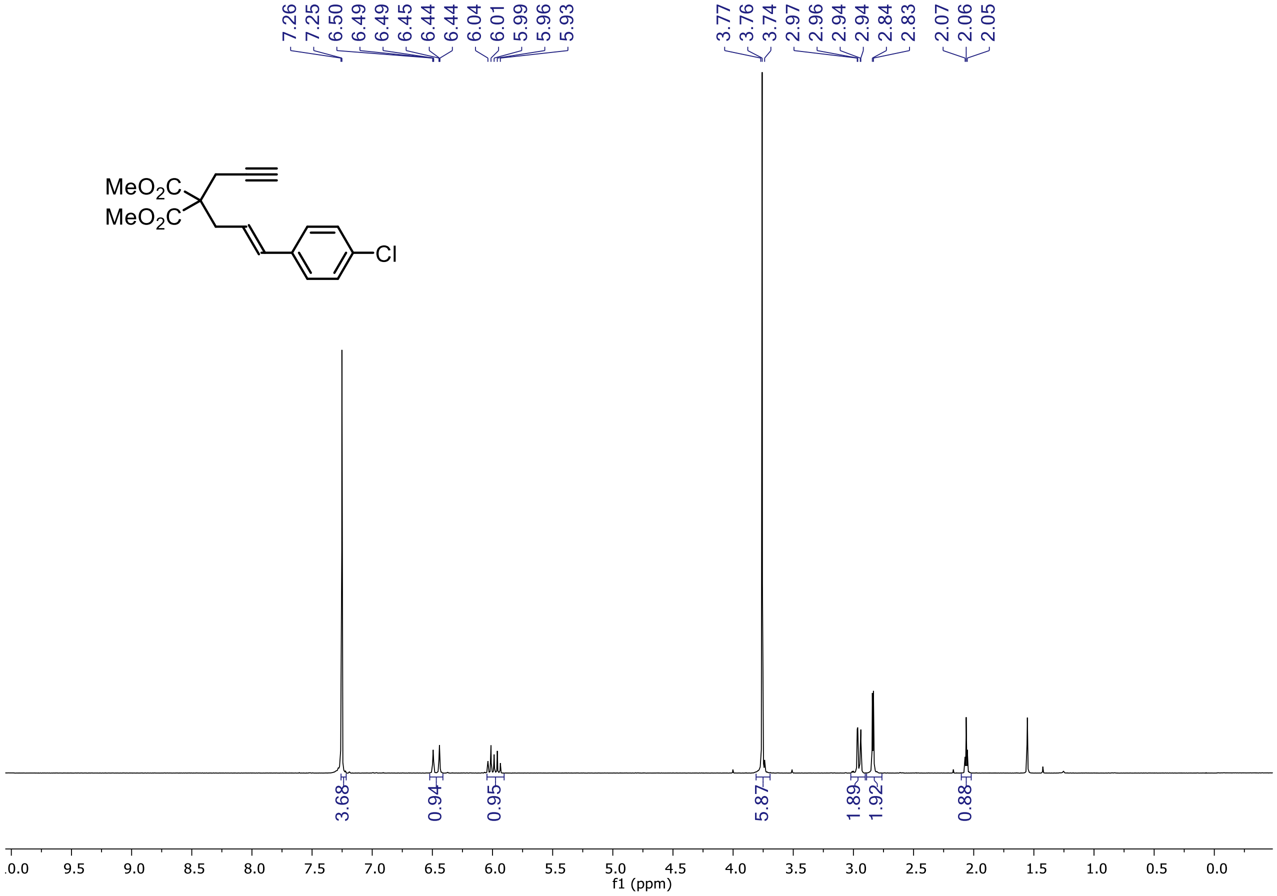

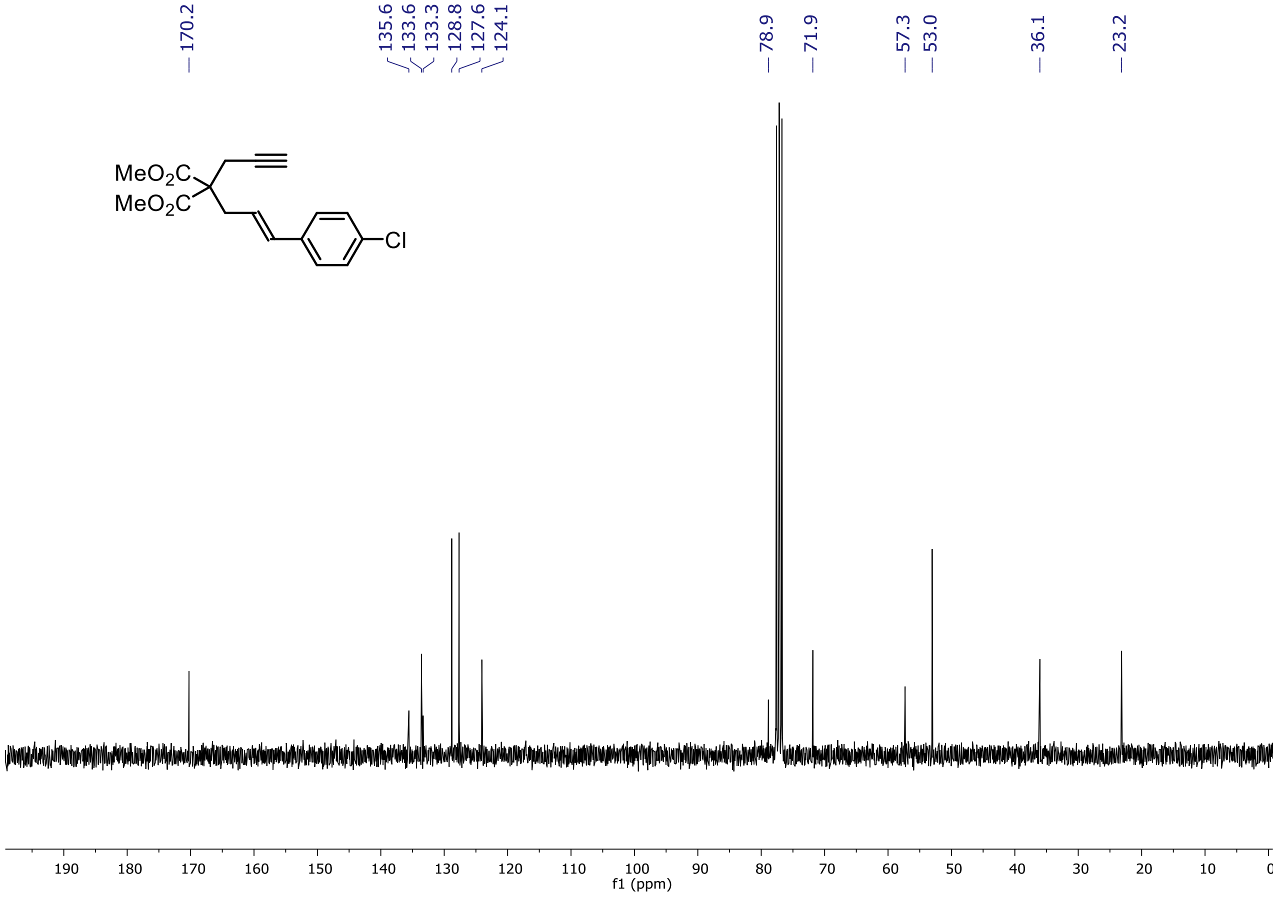

Compound **1h** ^{1}H and $^{13}C\{^{1}H\}$ NMR spectra in $CDCl_3$

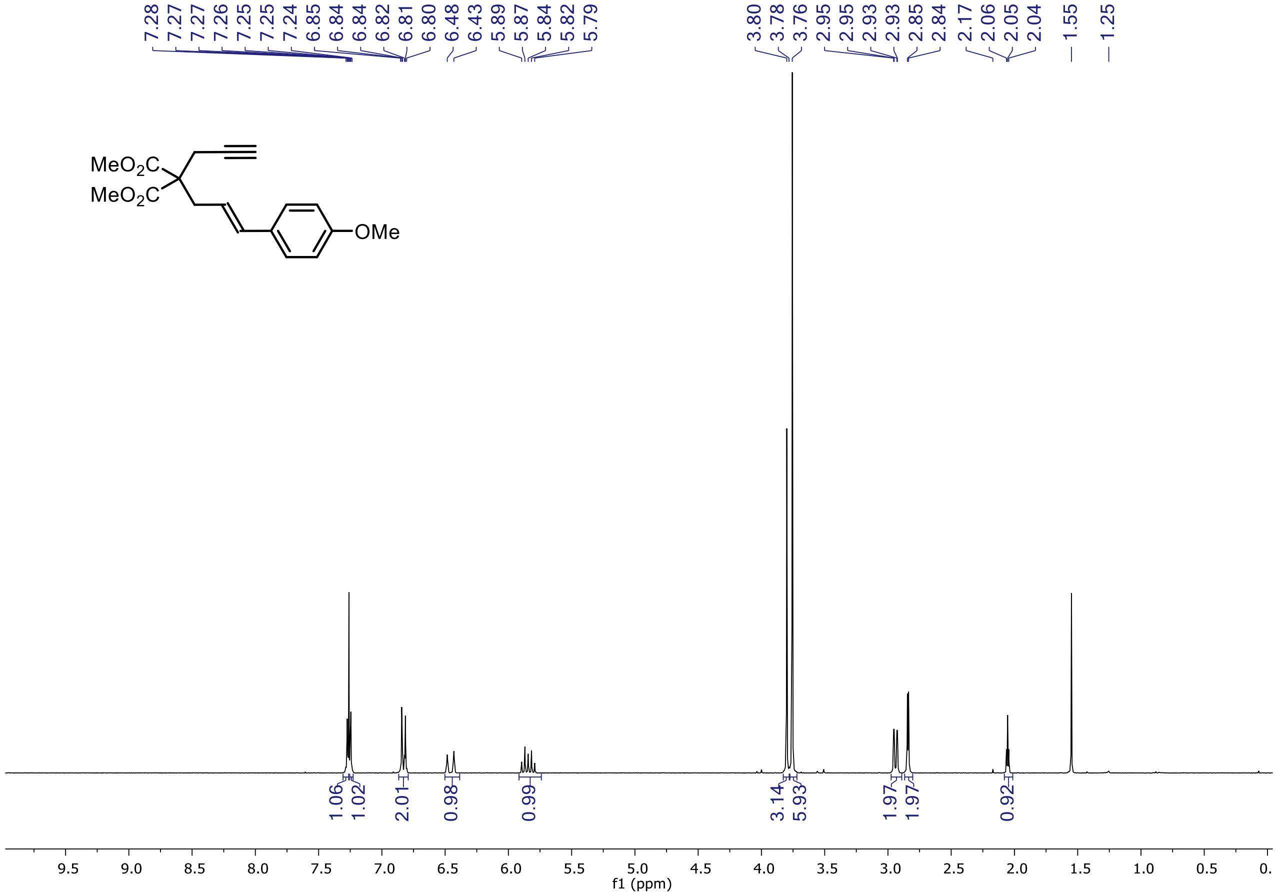

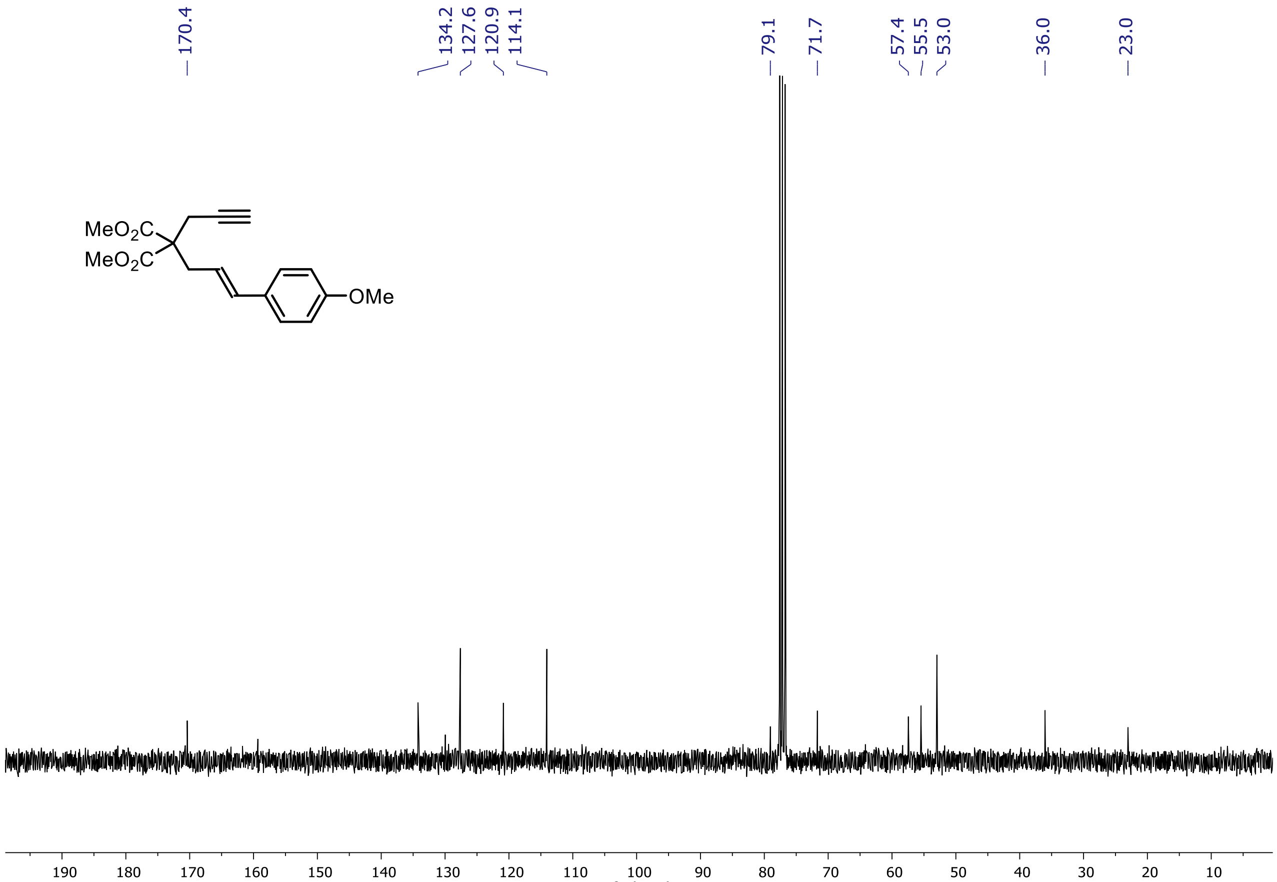

Compound **1i** ^{1}H and ^{13}C{^{1}H} NMR spectra in $CDCl_3$

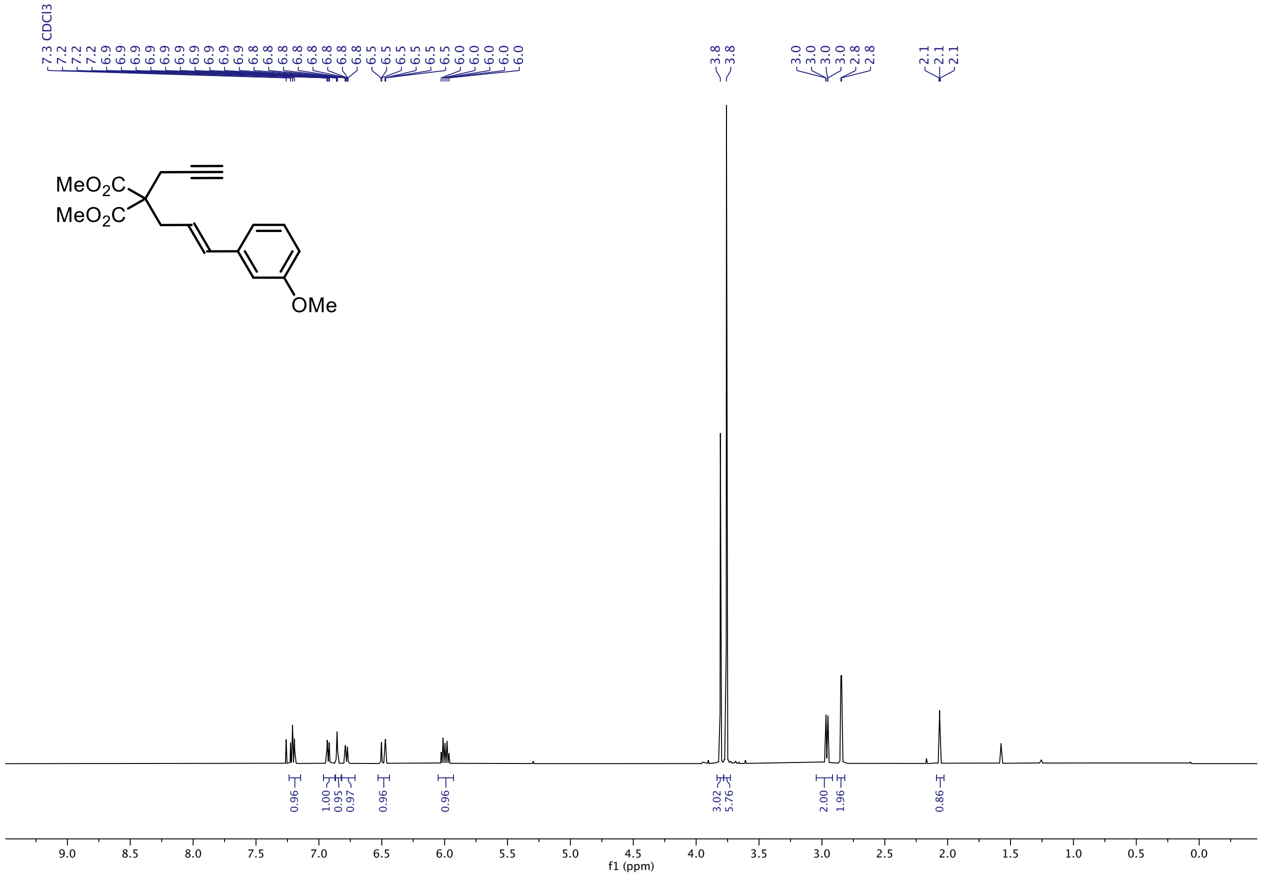

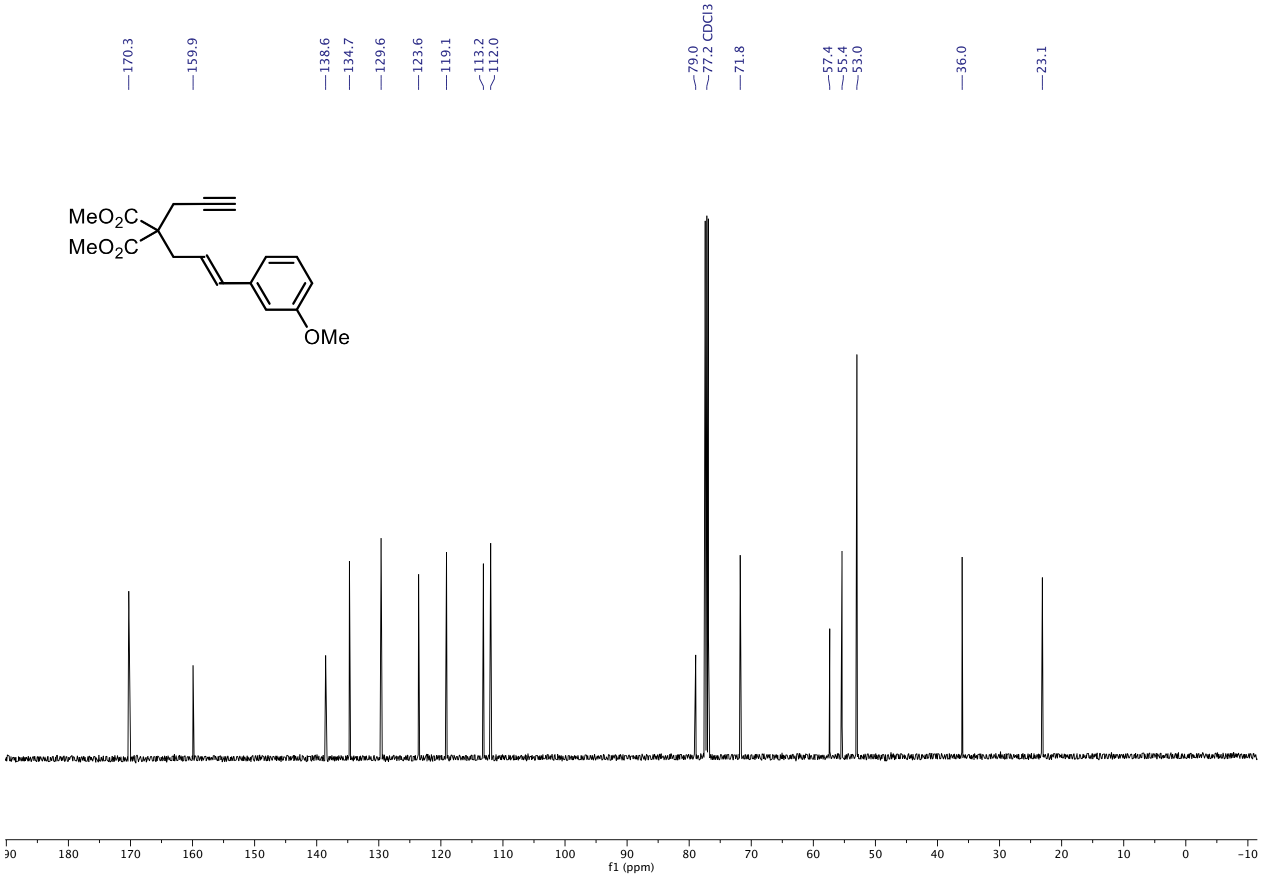

Compound **1j** ^{1}H and $^{13}C\{^{1}H\}$ NMR spectra in $CDCl_3$

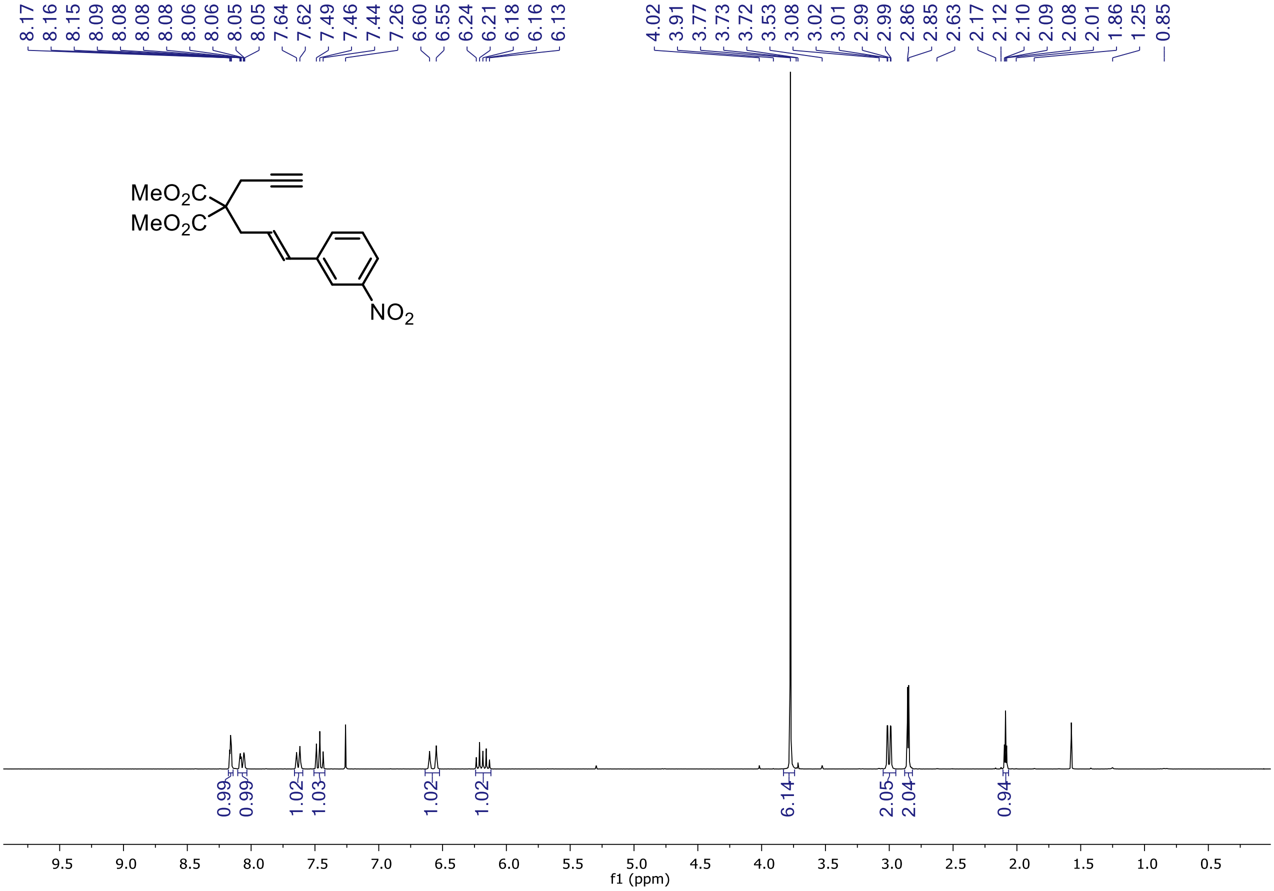

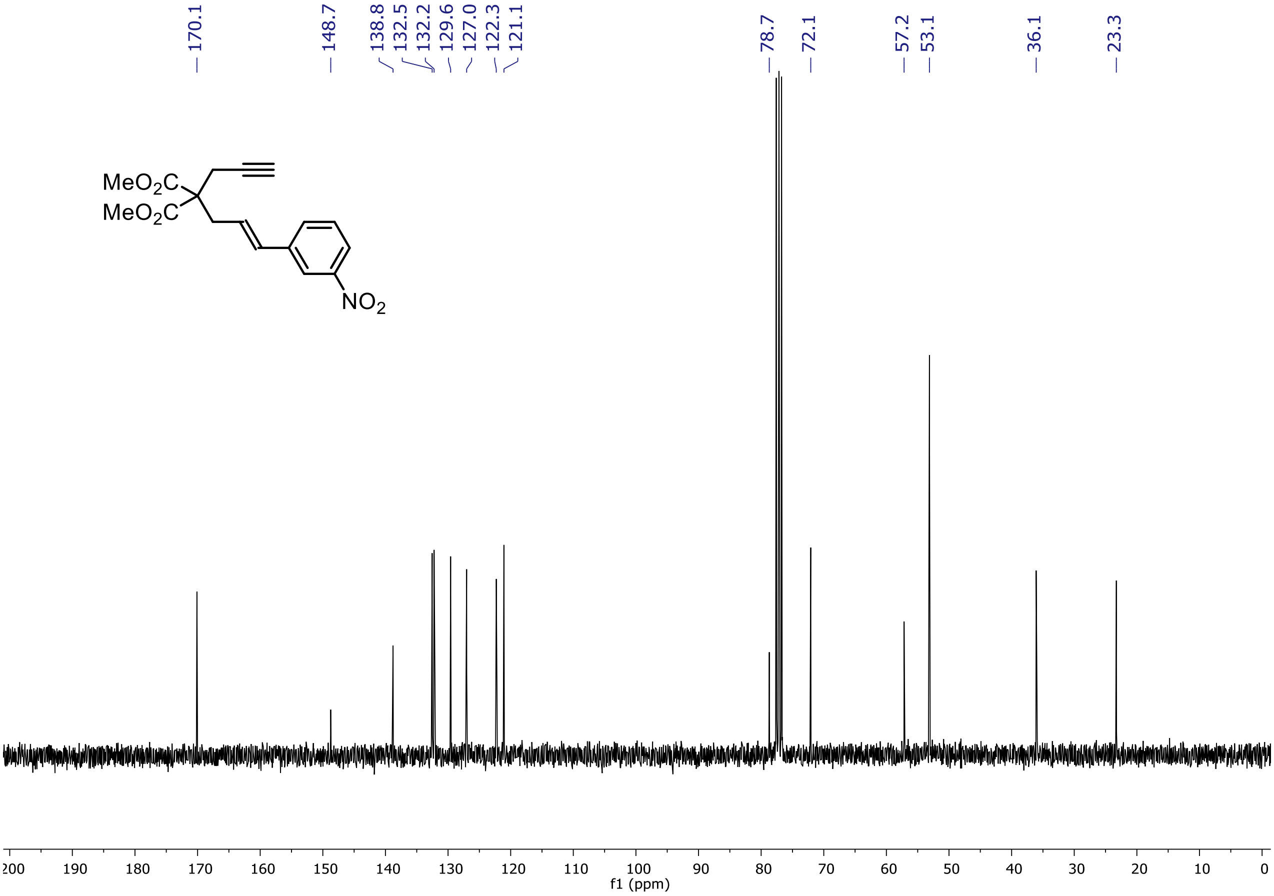

Compound **1k** ^{1}H and ^{13}C{^{1}H} NMR spectra in $CDCl_3$

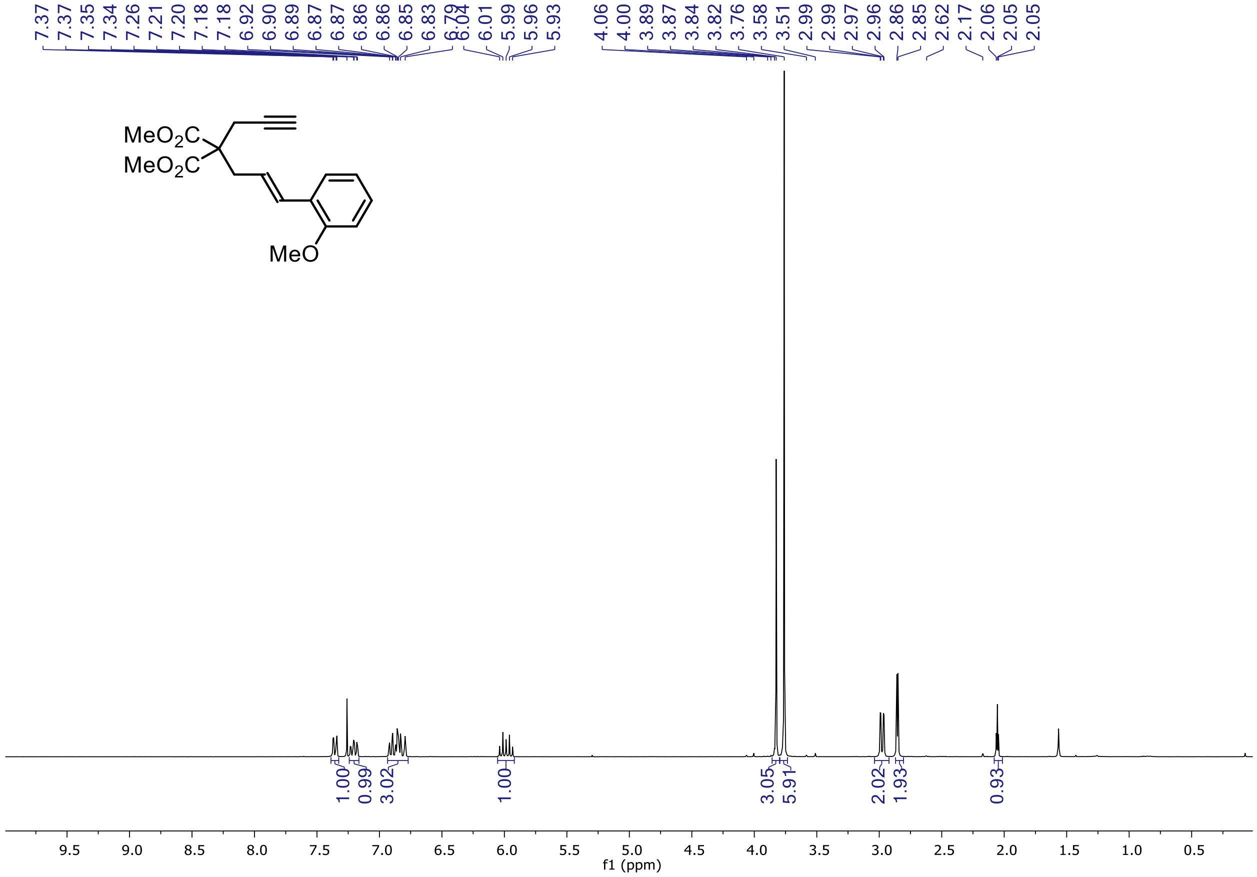

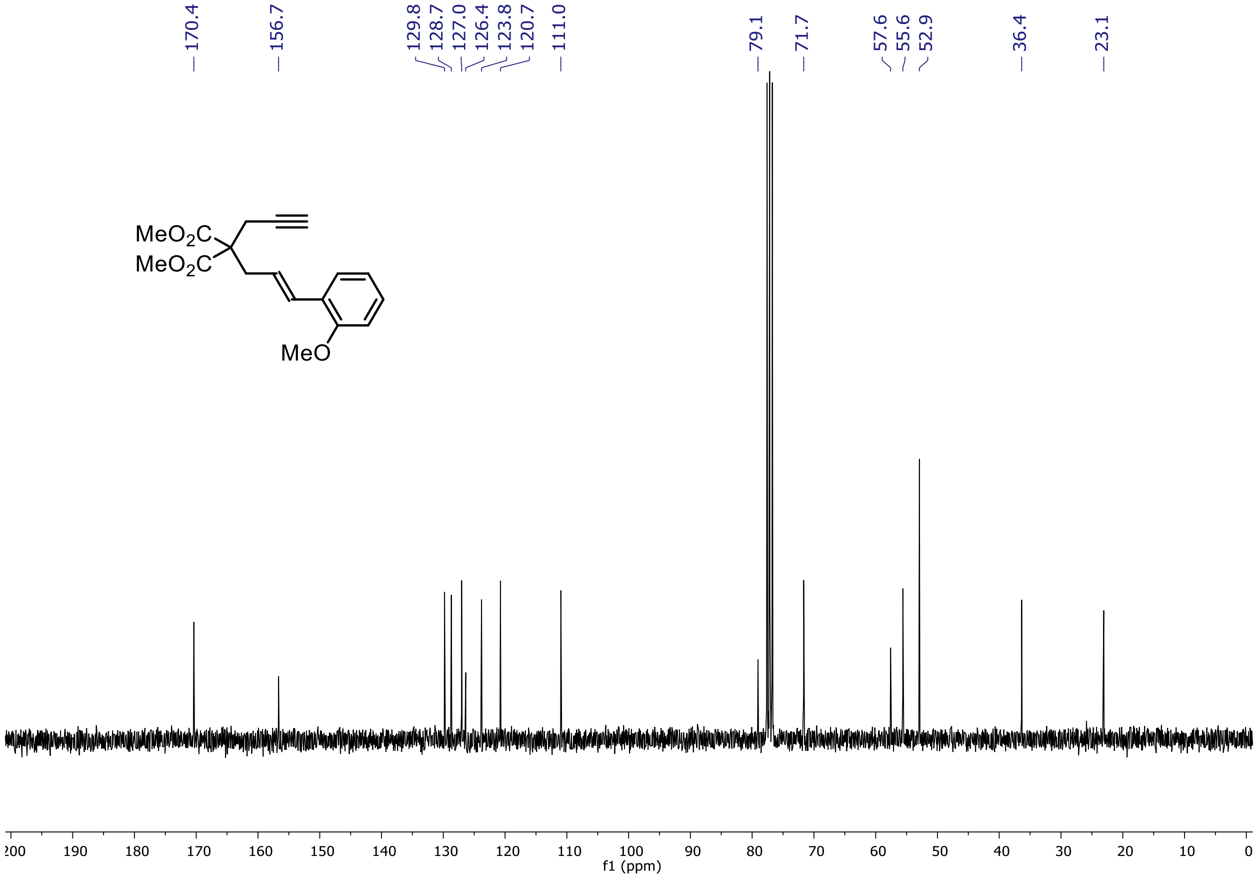

Compound **1l** ^{1}H, ^{19}F and $^{13}C\{^{1}H\}$ NMR spectra in $CDCl_3$

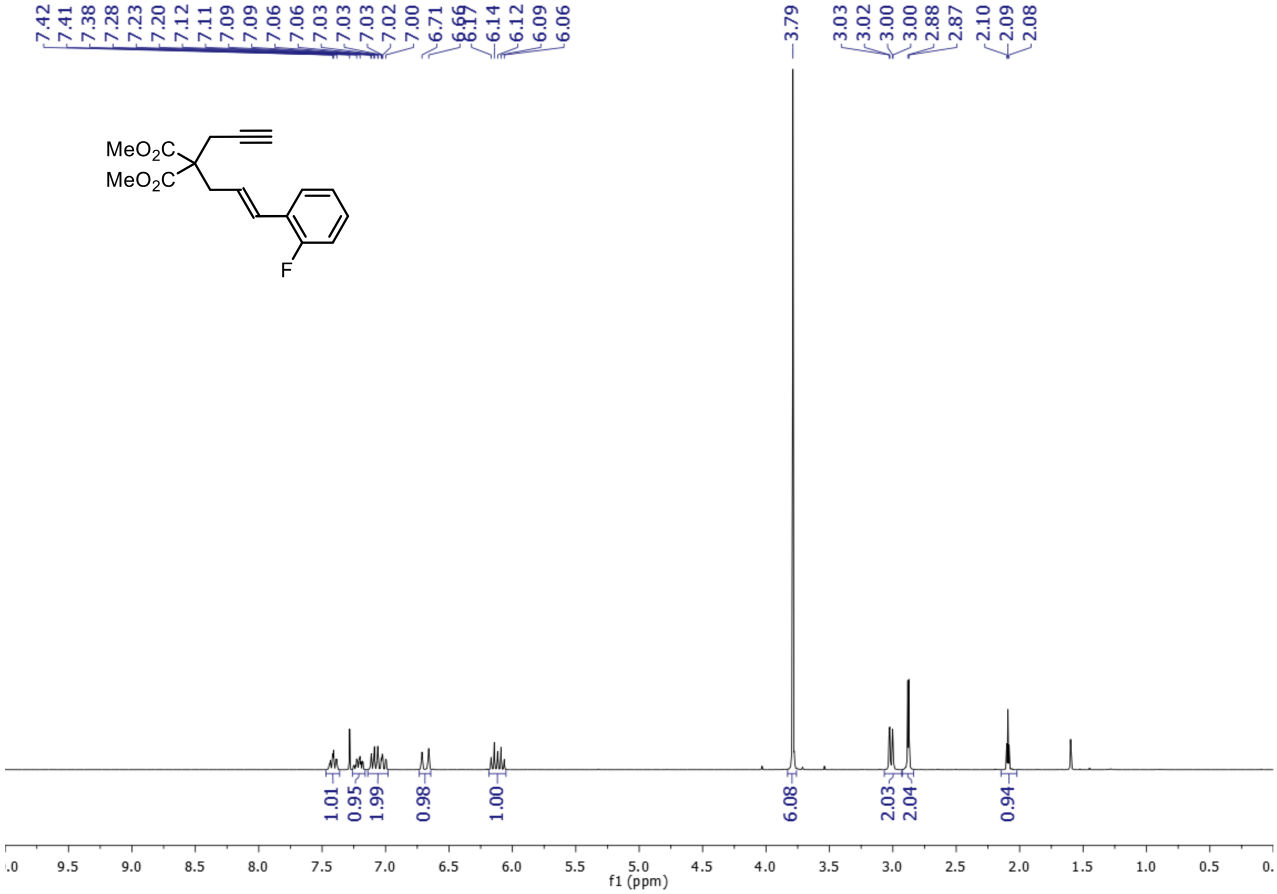

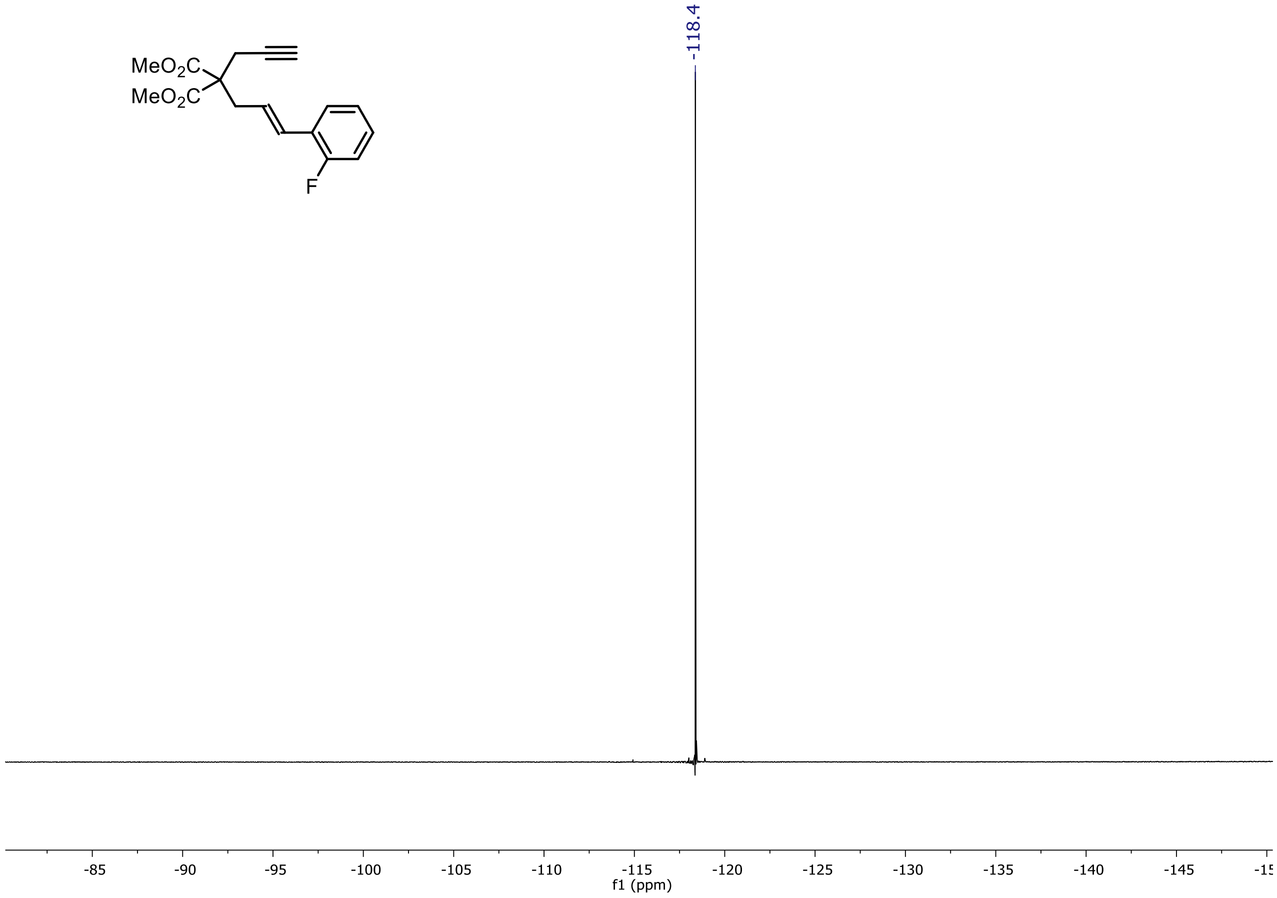

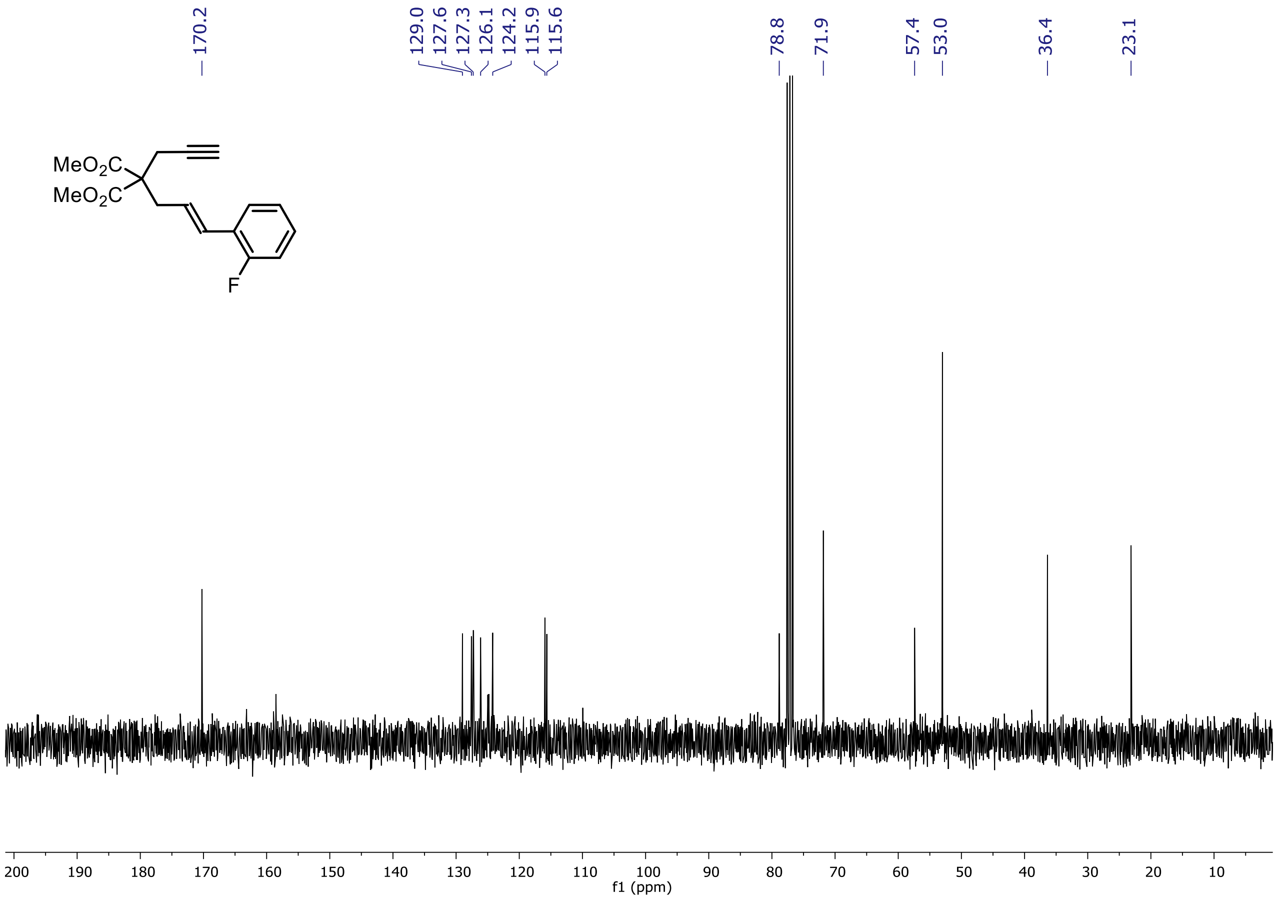

170.2
129.0
127.6
127.3
126.1
124.2
115.9
115.6
78.8
71.9
57.4
53.0
36.4
23.1
MeO2C
MeO2C
F
200 190 180 170 160 150 140 130 120 110 100 90 80 70 60 50 40 30 20 10
f1 (ppm)

Compound **1m** ^{1}H and ^{13}C{^{1}H} NMR spectra in $CDCl_3$

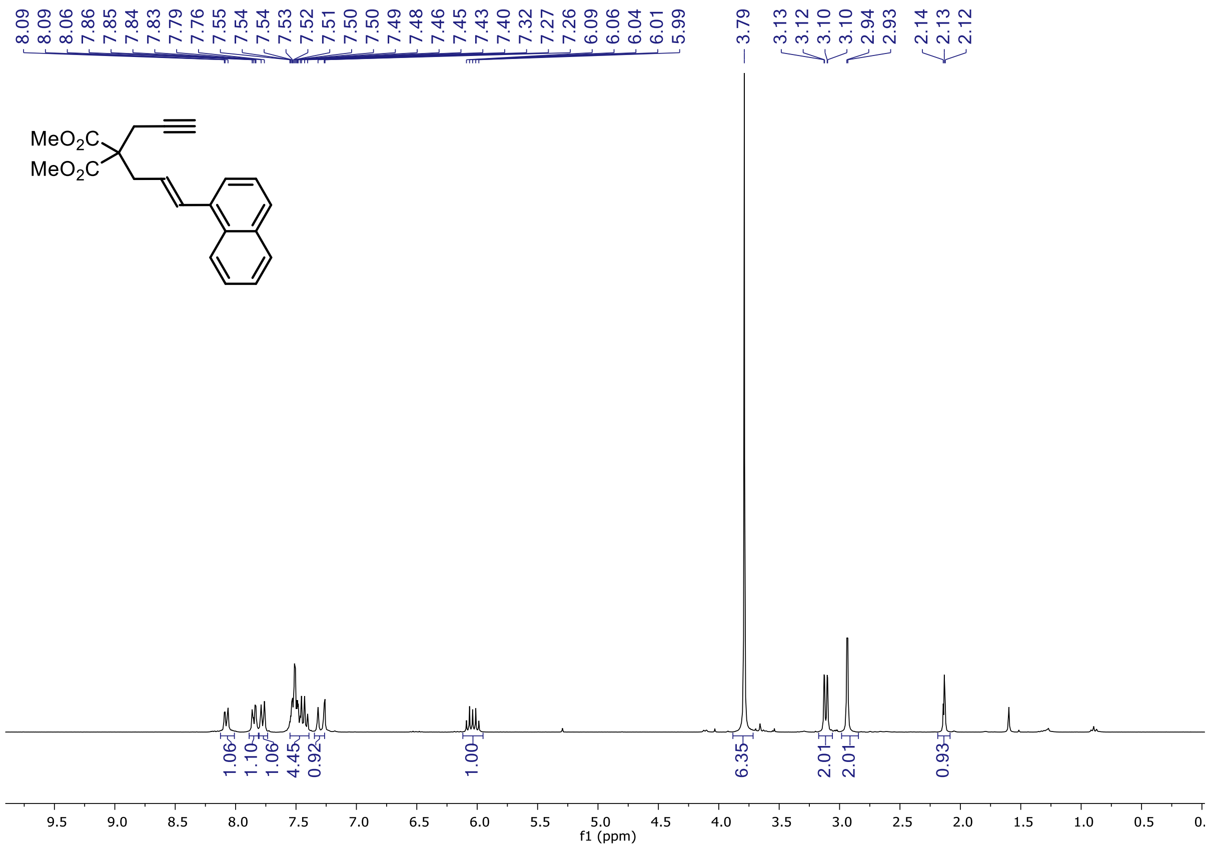

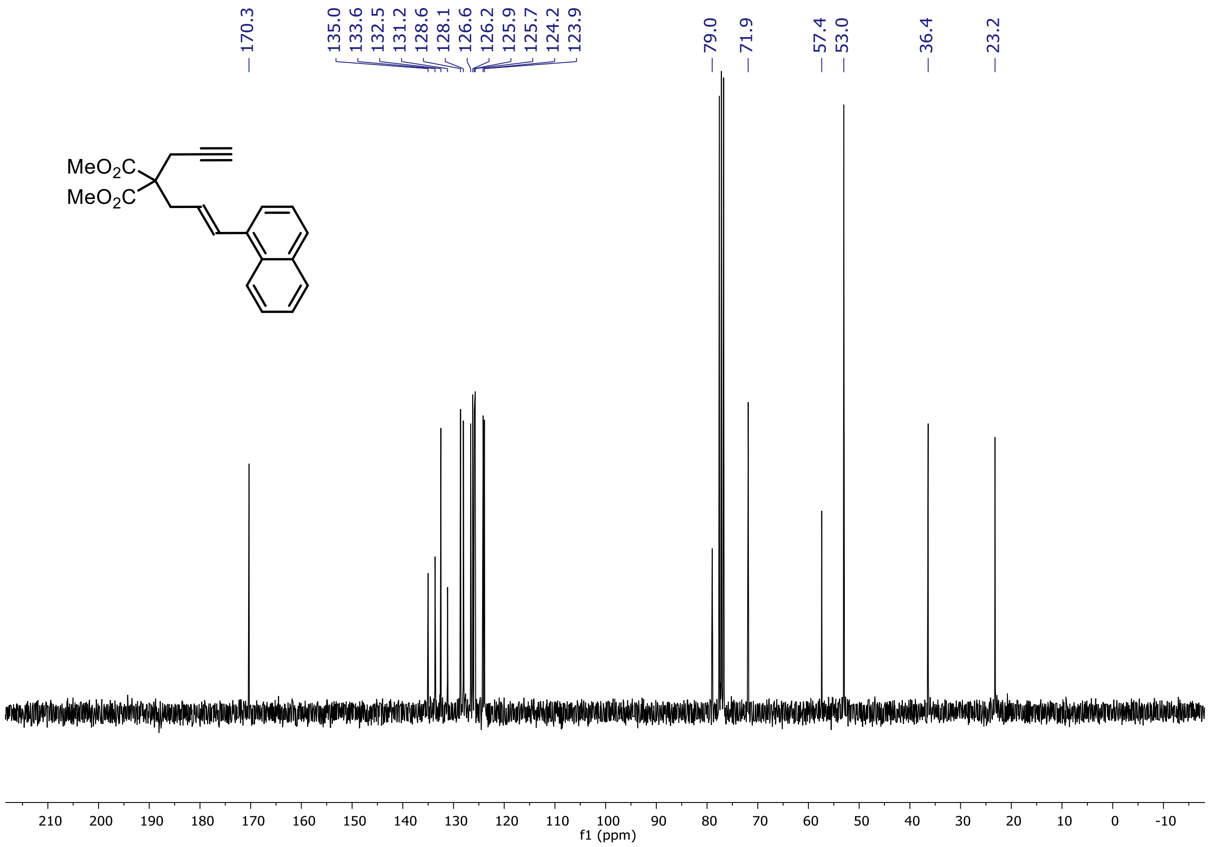

Compound **1n** ^{1}H and $^{13}C\{^{1}H\}$ NMR spectra in $CDCl_3$

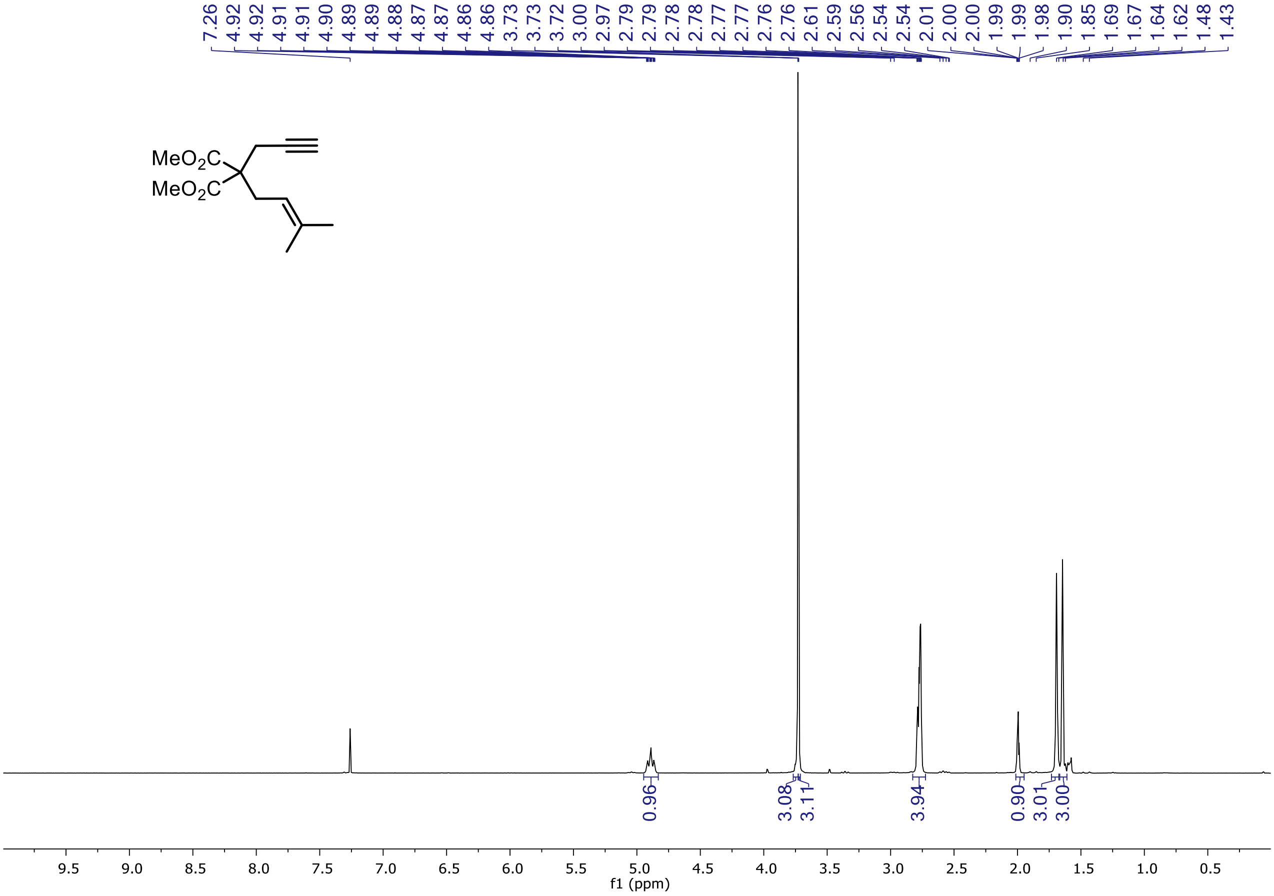

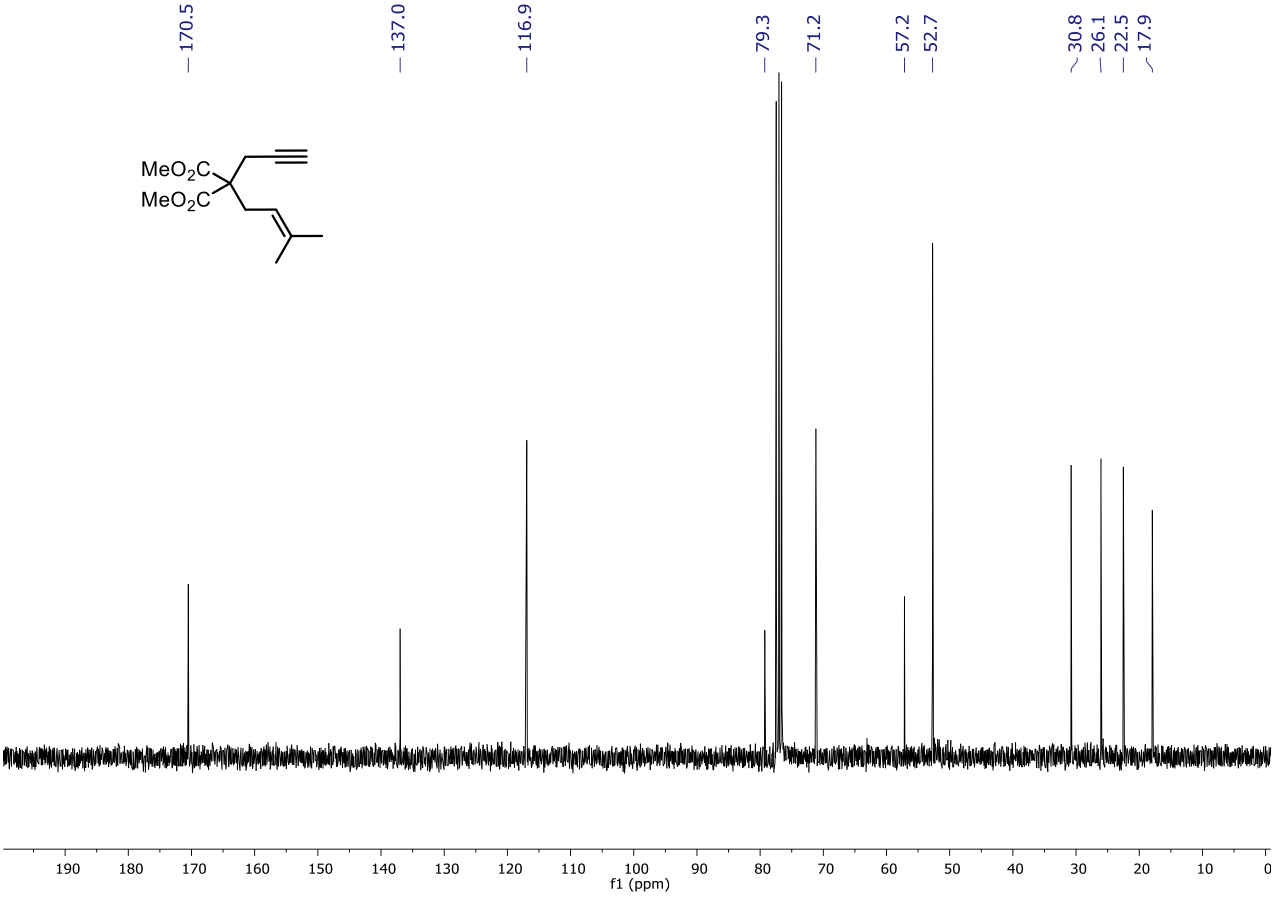

Compound **1o** ^{1}H and $^{13}C\{^{1}H\}$ NMR spectra in $CDCl_3$

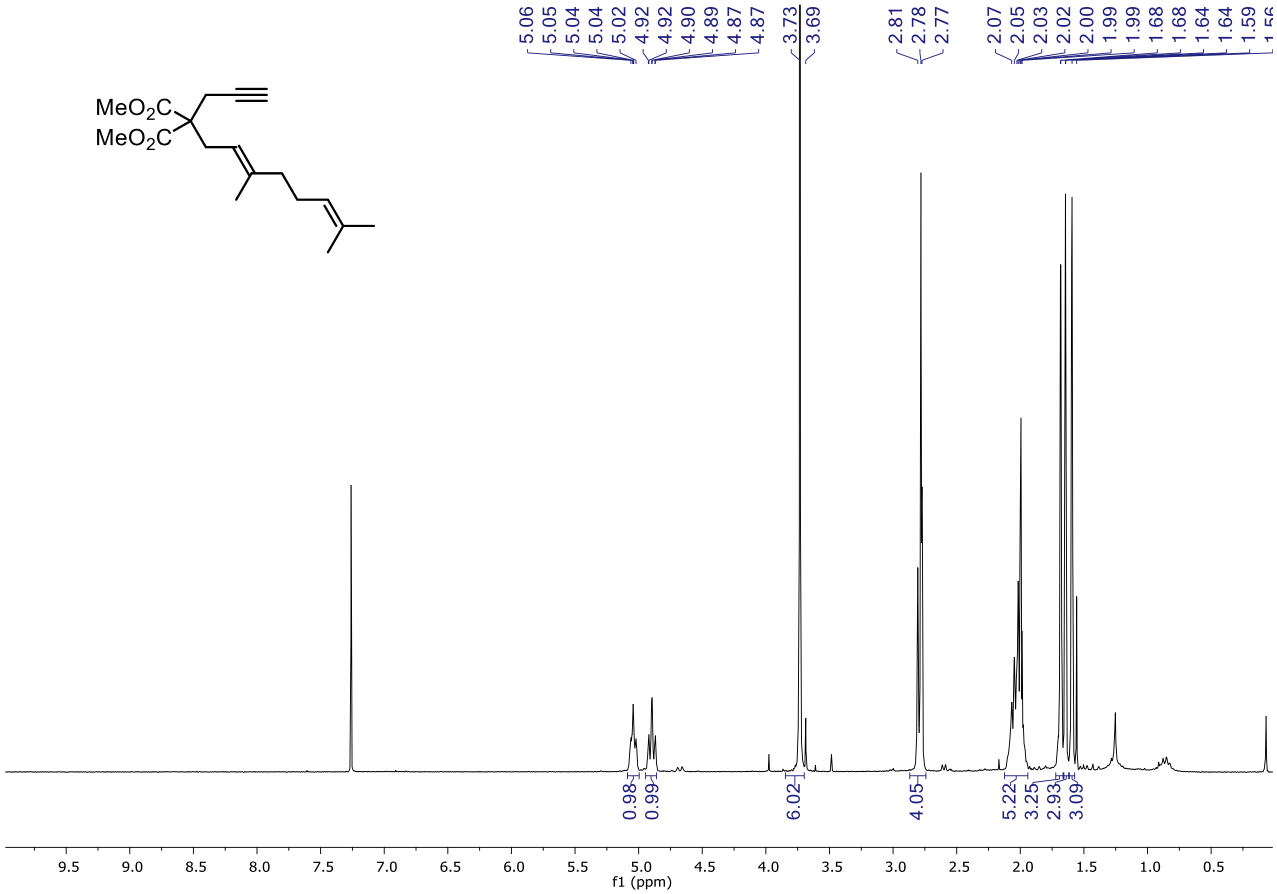

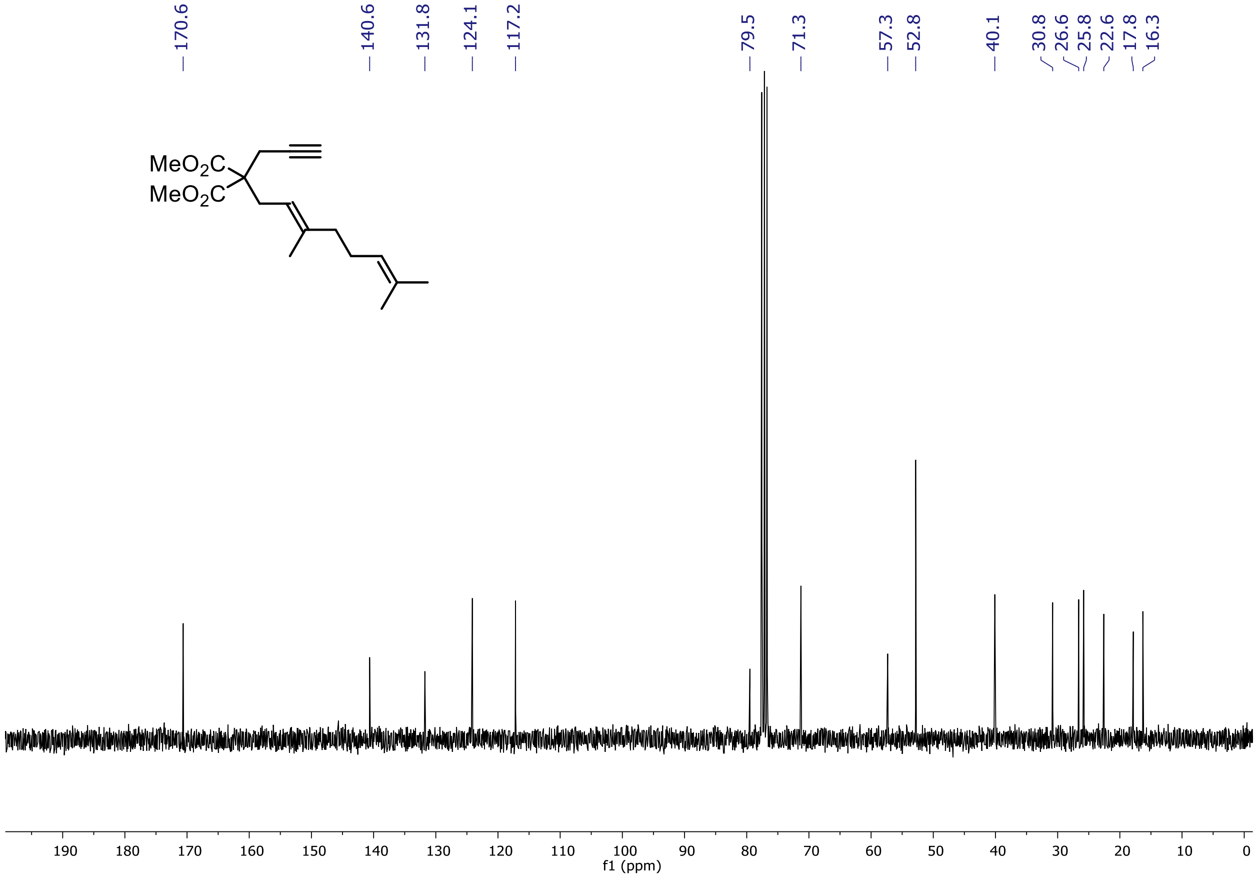

Compound **2a** ^{1}H and $^{13}C\{^{1}H\}$ NMR spectra in $CDCl_3$

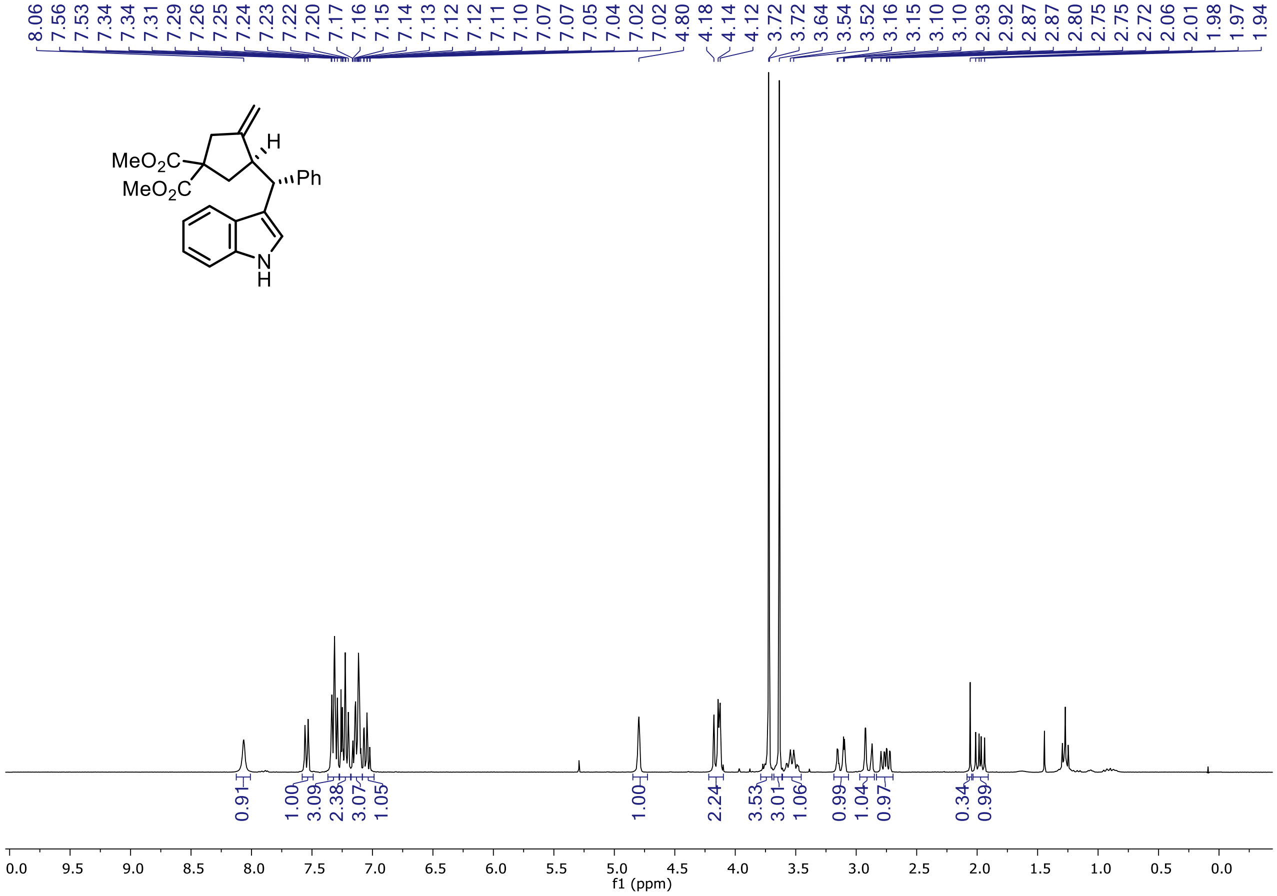

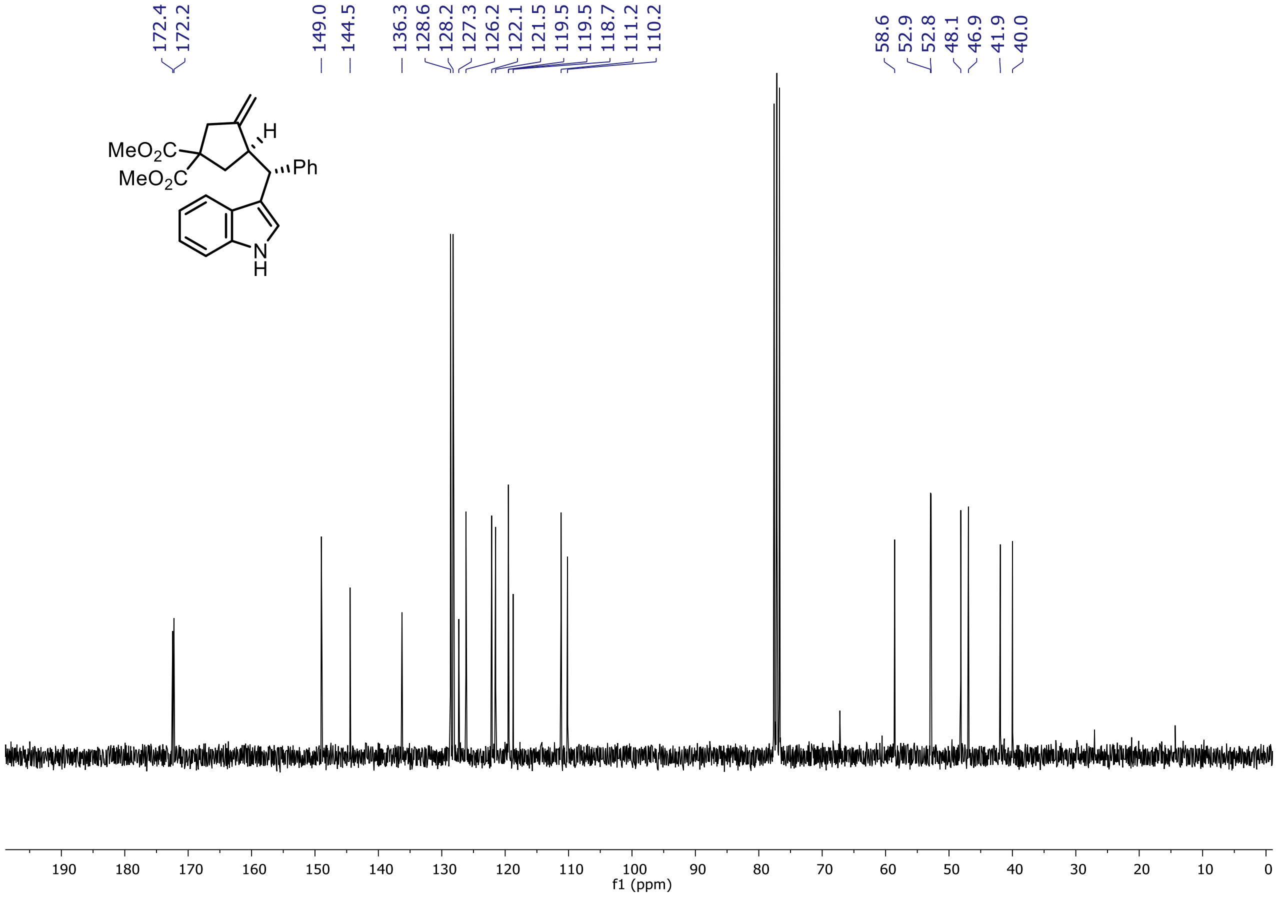

Compound **2b** ^{1}H and ^{13}C{^{1}H} NMR spectra in $CDCl_3$

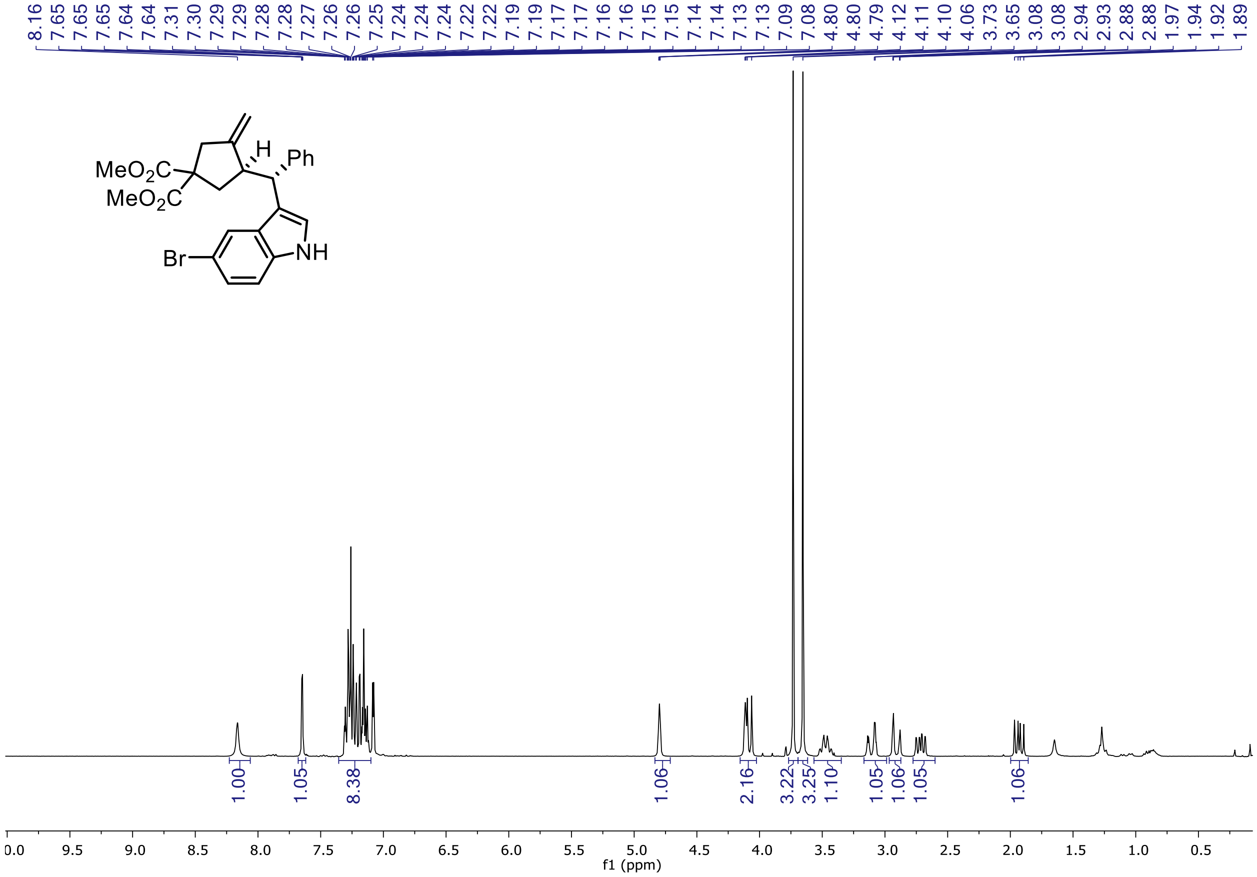

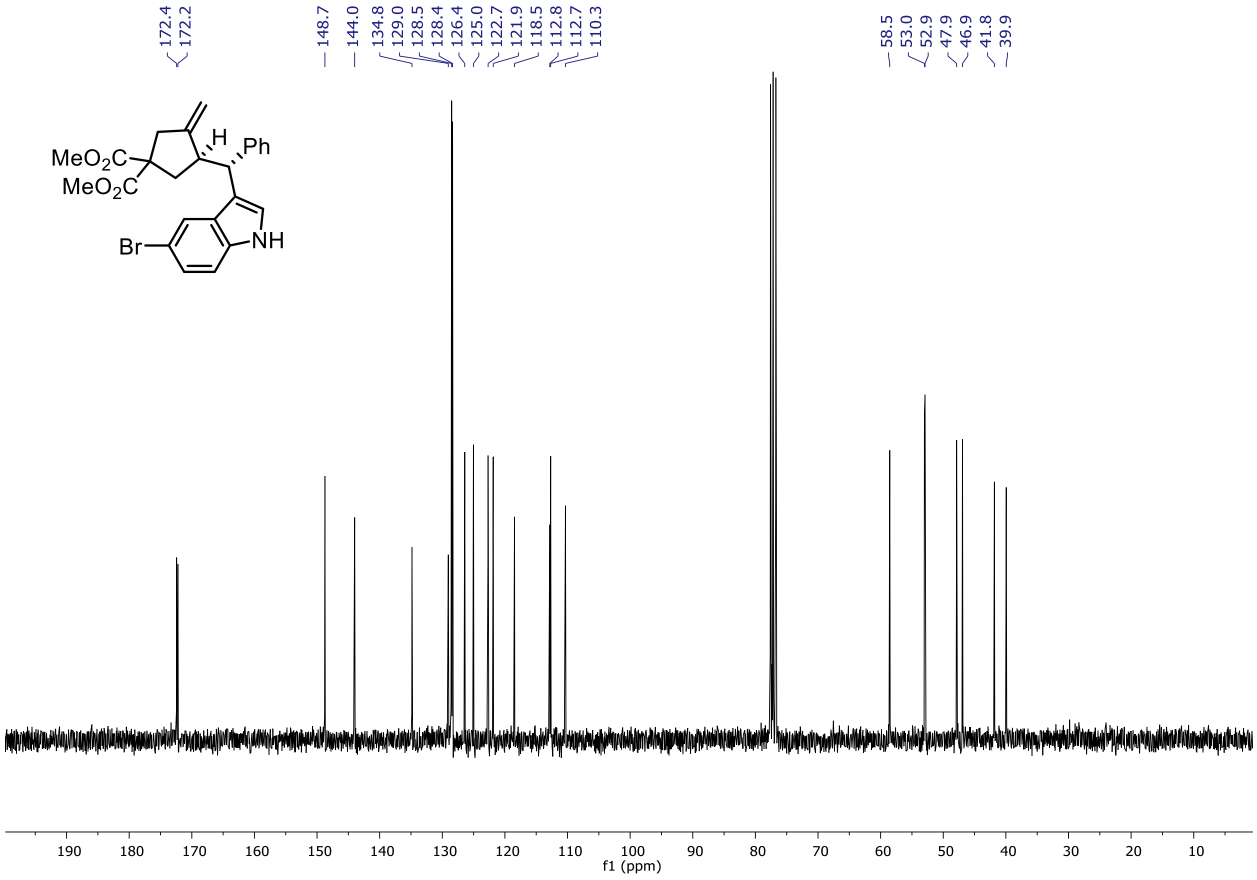

Compound **2c** ^{1}H and $^{13}C\{^{1}H\}$ NMR spectra in $CDCl_3$

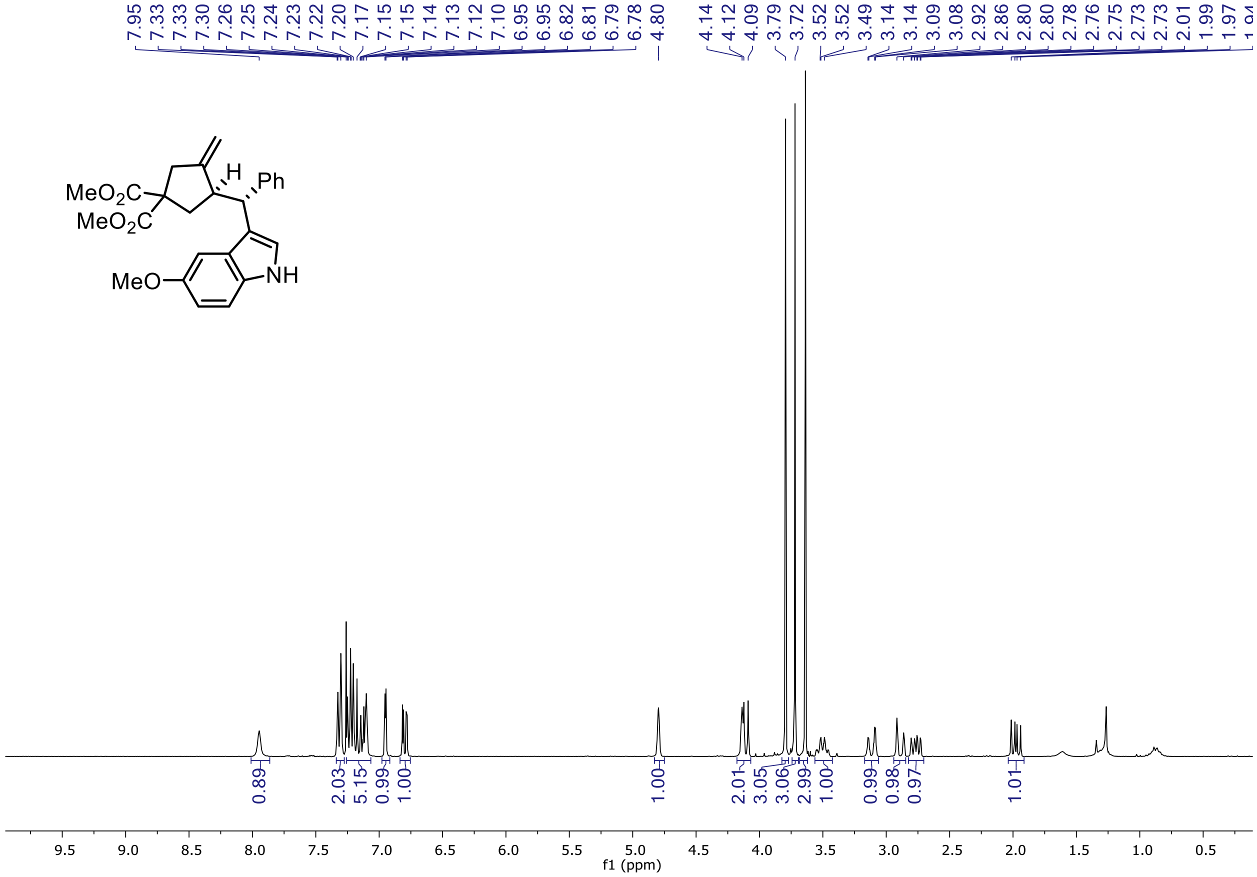

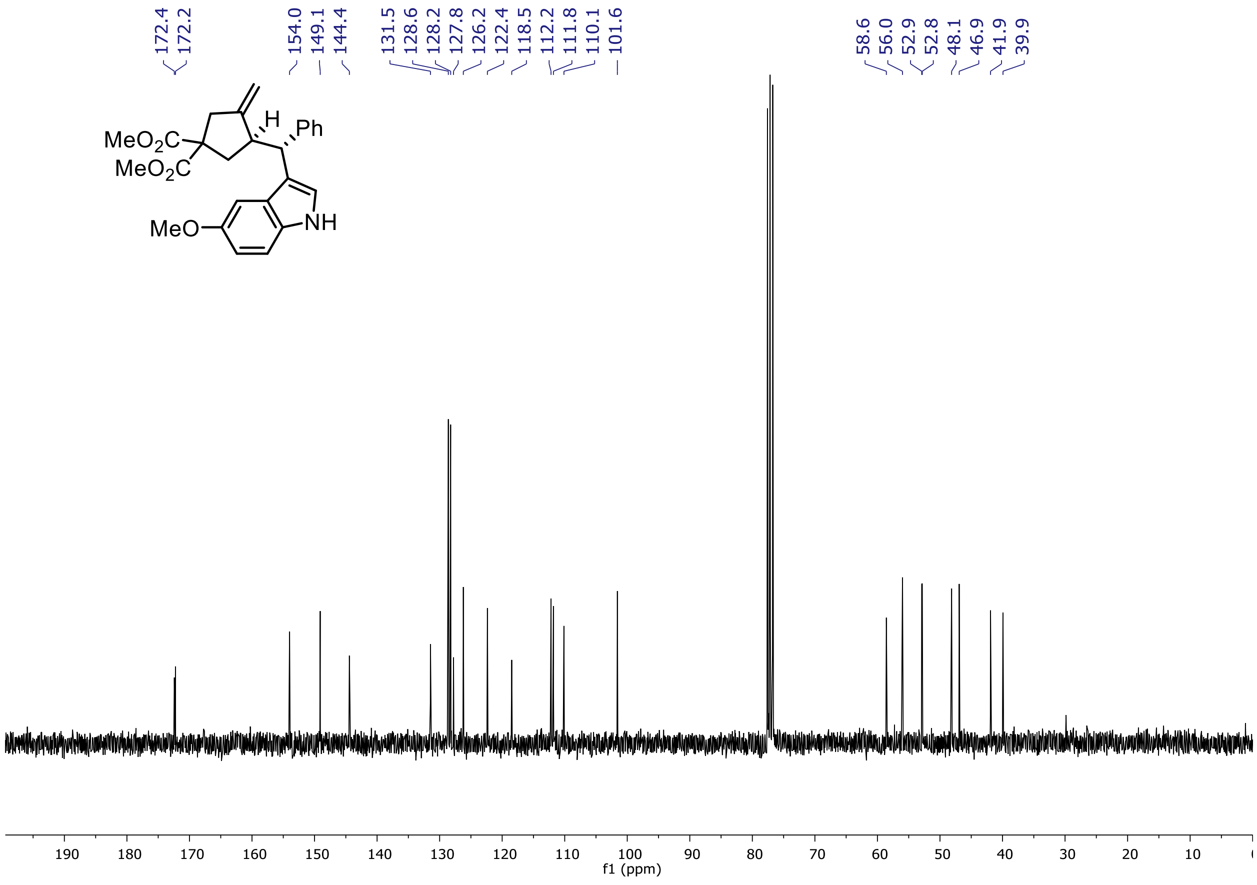

Compound **2d** ^{1}H and $^{13}C\{^{1}H\}$ NMR spectra in $CDCl_3$

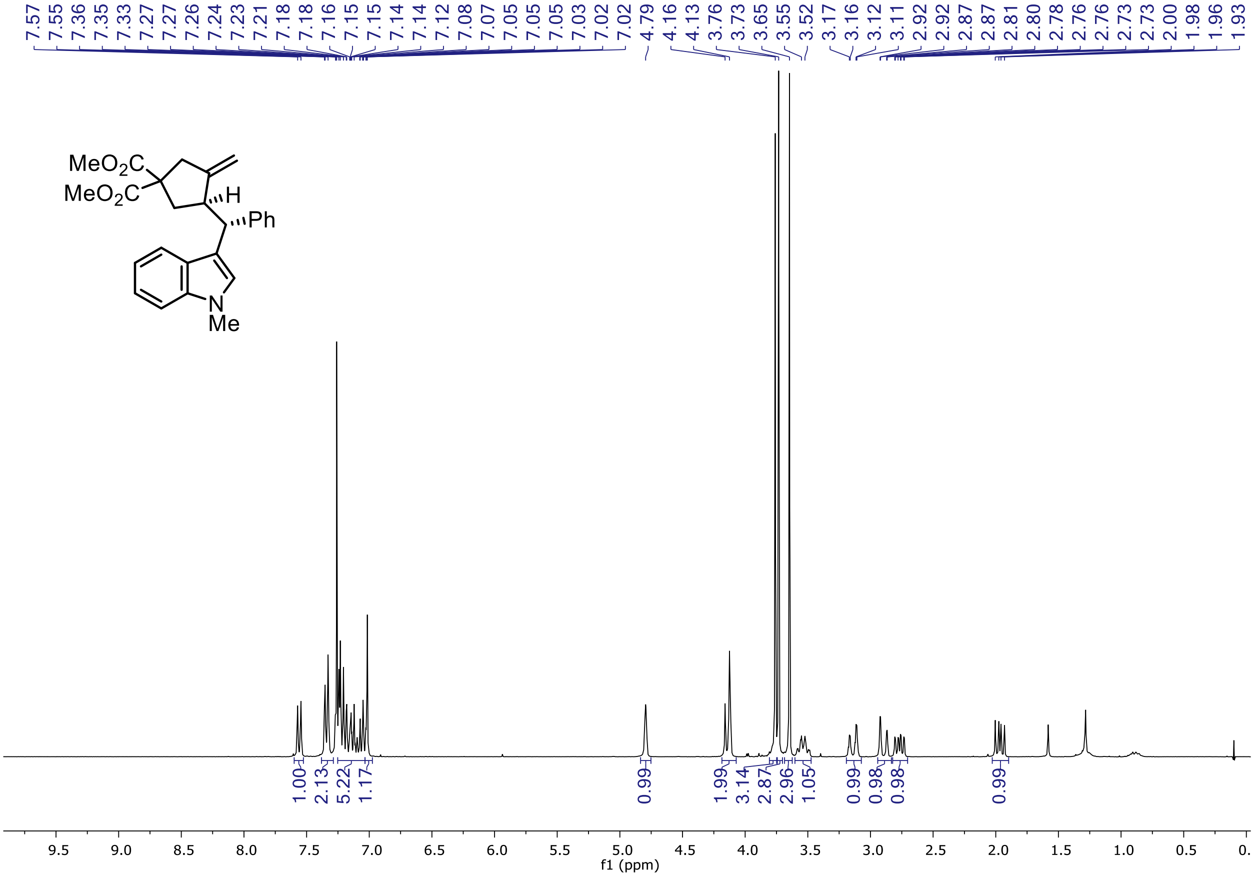

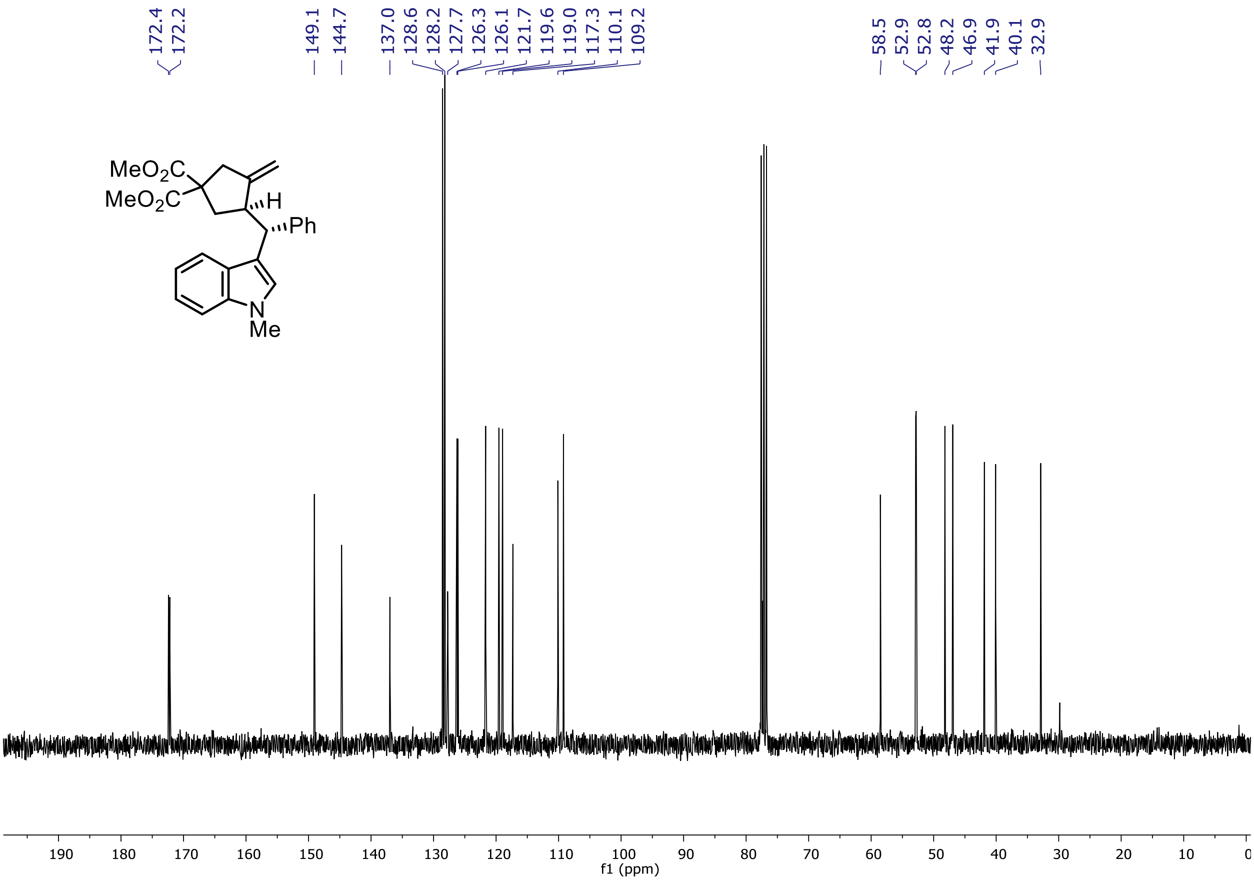

Compound **2e** ^{1}H and ^{13}C{^{1}H} NMR spectra in $CDCl_3$

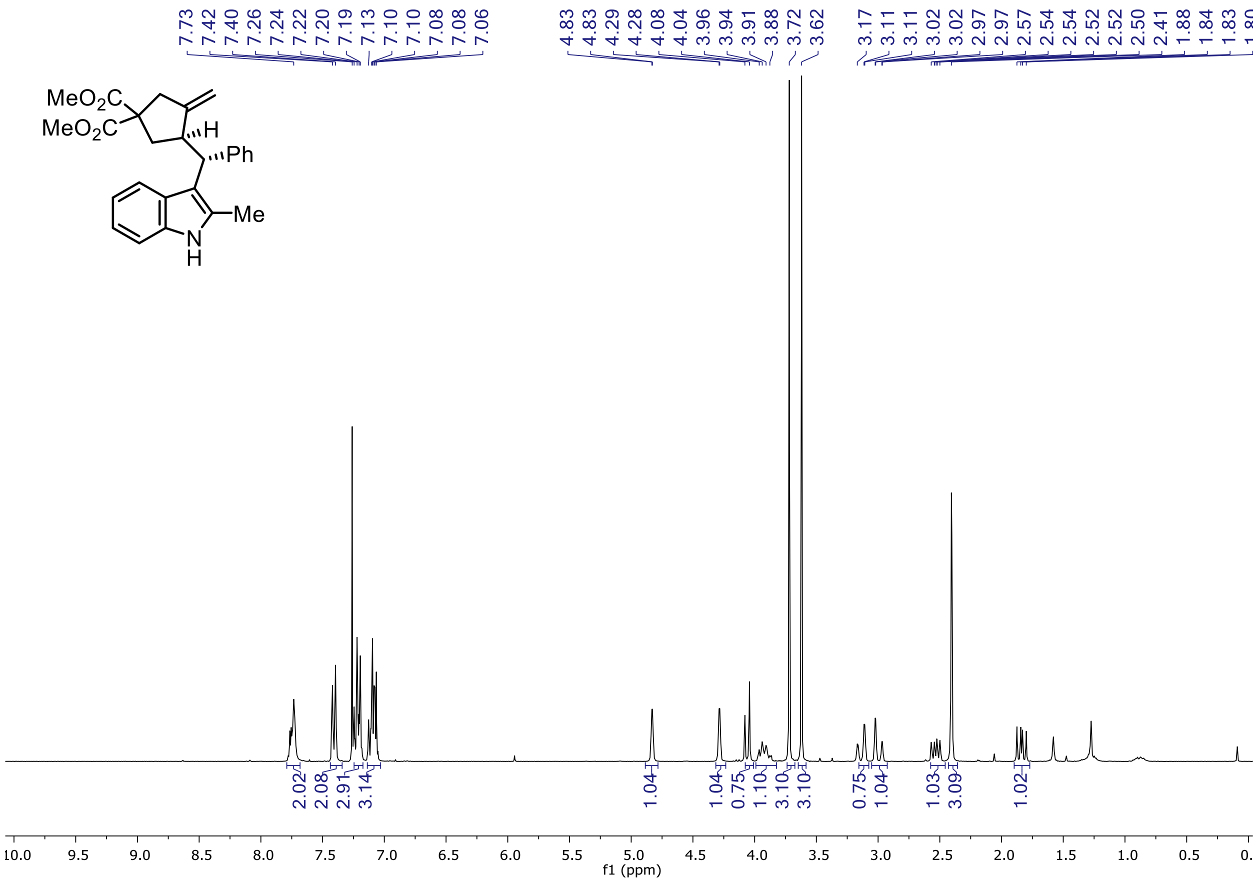

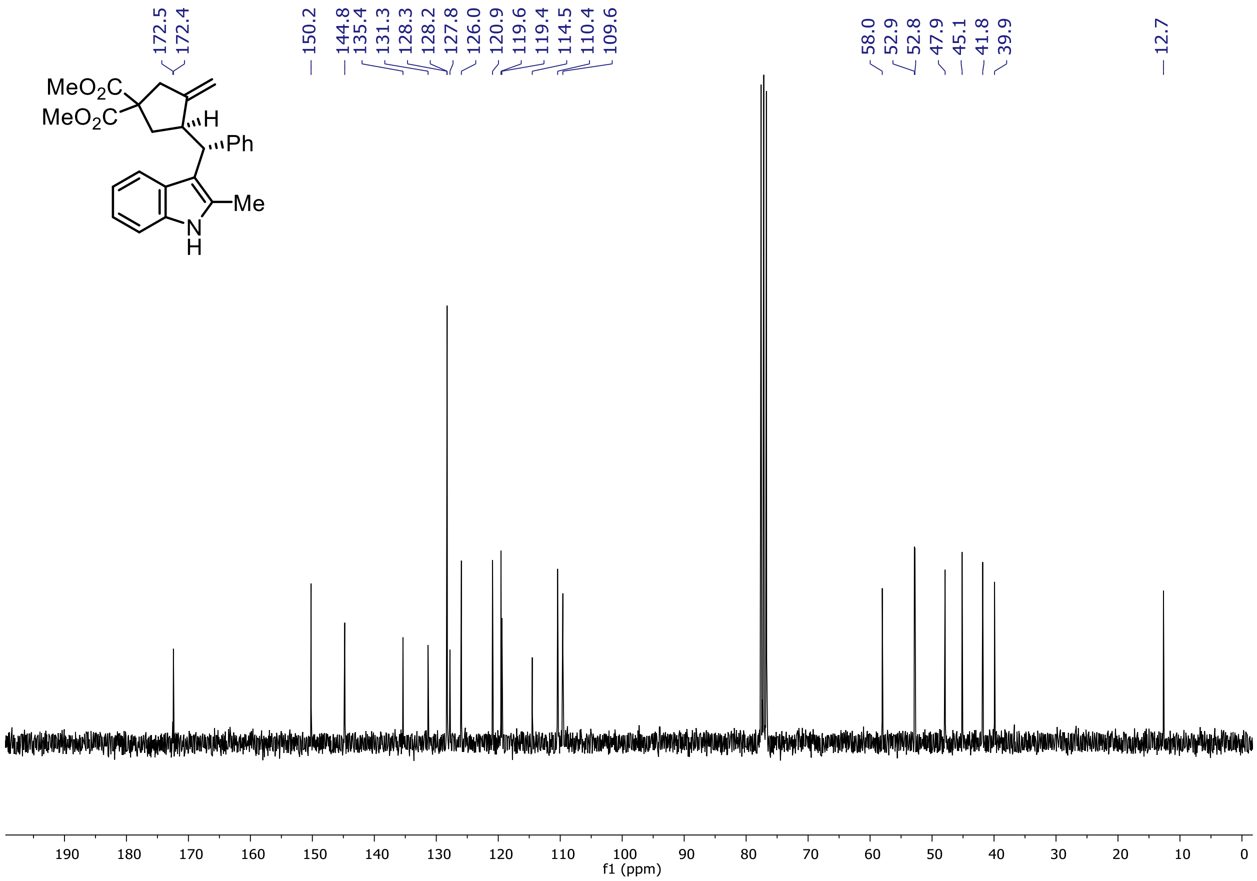

Compound **2f** ^{1}H and ^{13}C{^{1}H} NMR spectra in CDCl$_3$

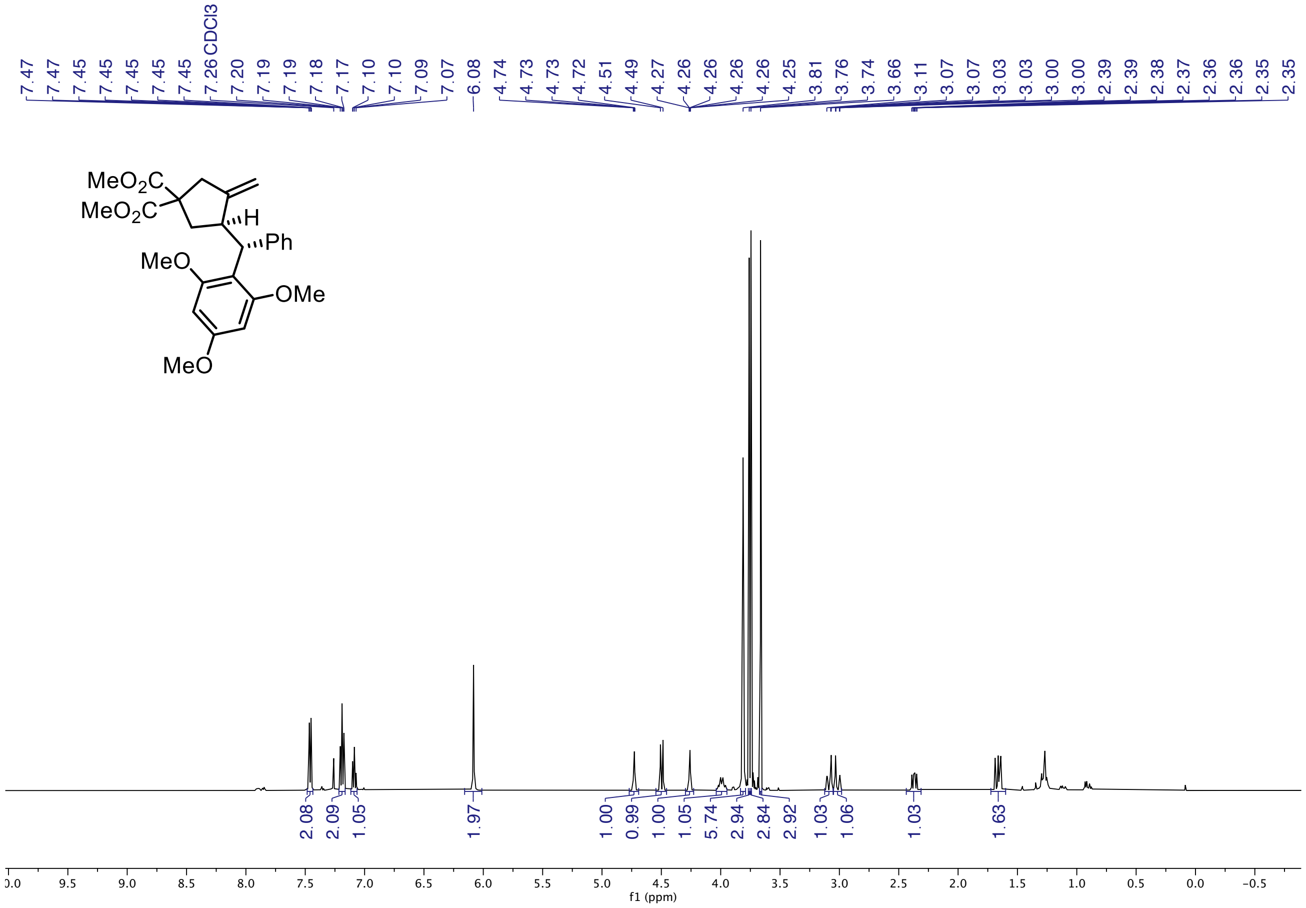

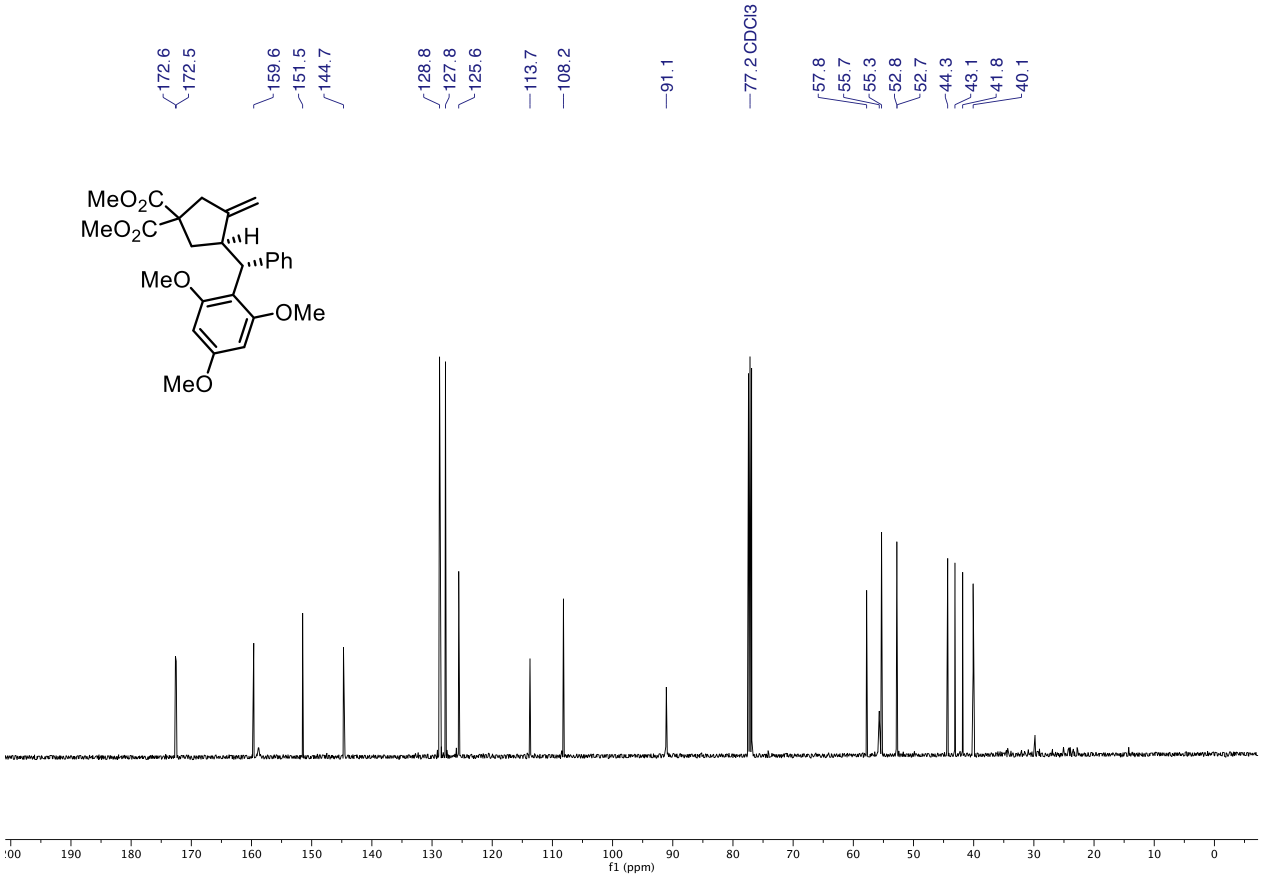

Compound **2g** ^{1}H and $^{13}C\{^{1}H\}$ NMR spectra in $CDCl_3$

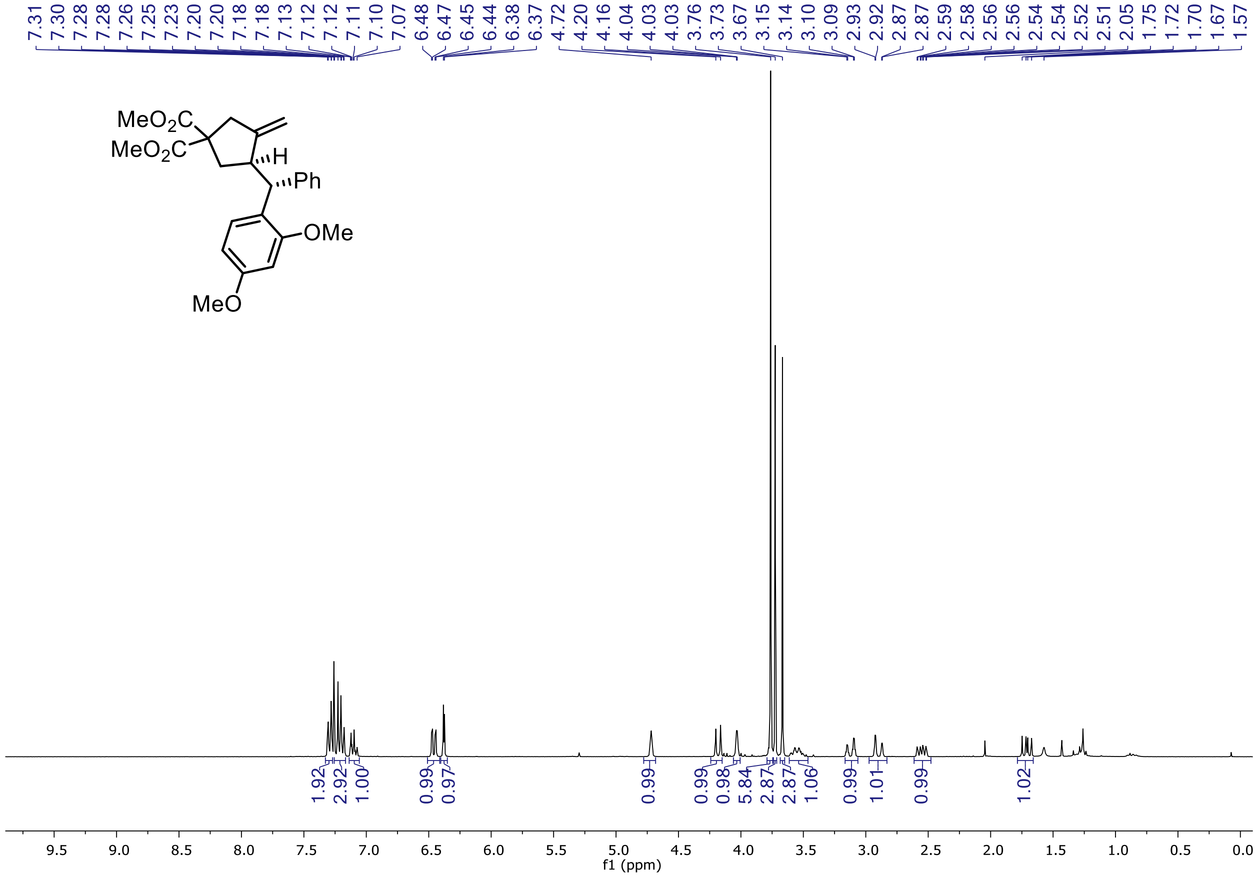

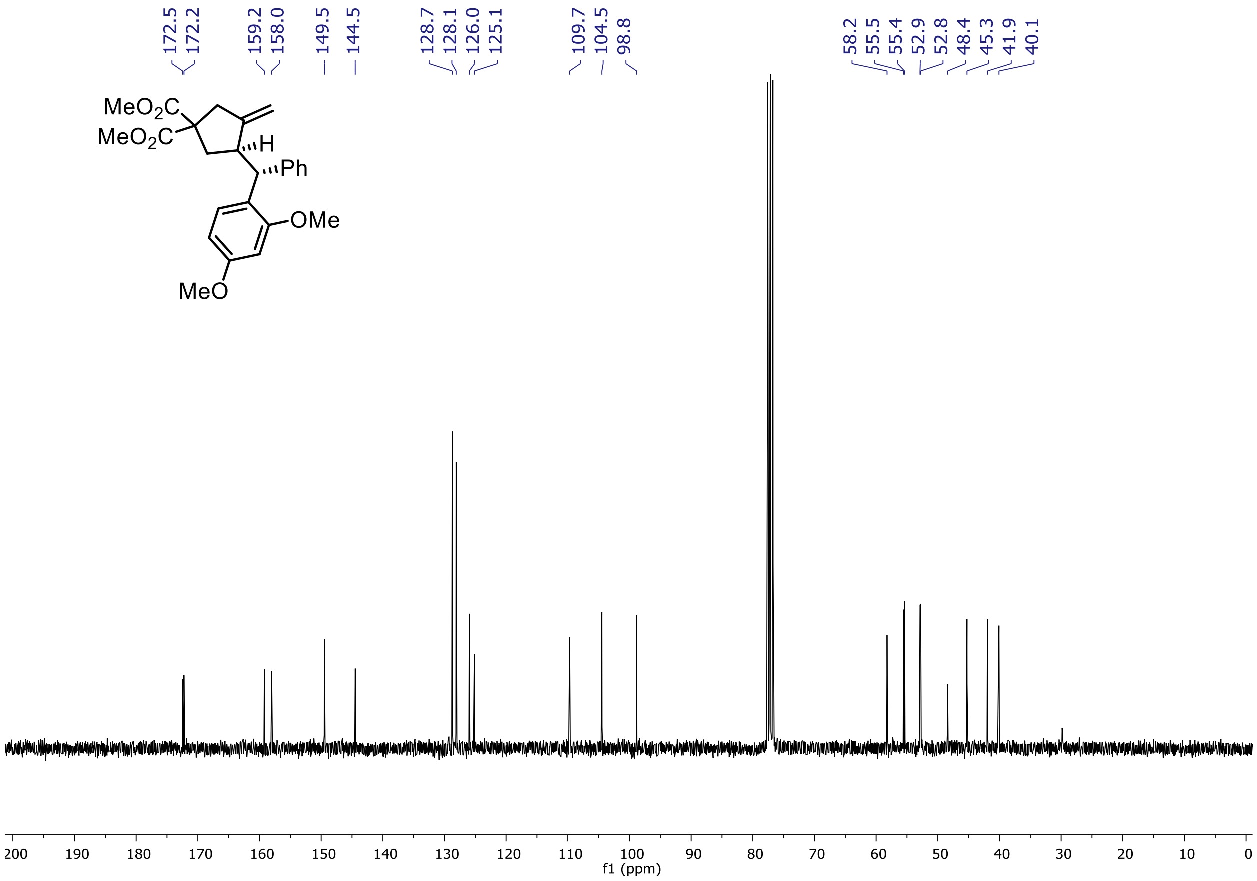

Compound **2h** ^{1}H and $^{13}C\{^{1}H\}$ NMR spectra in $CDCl_3$

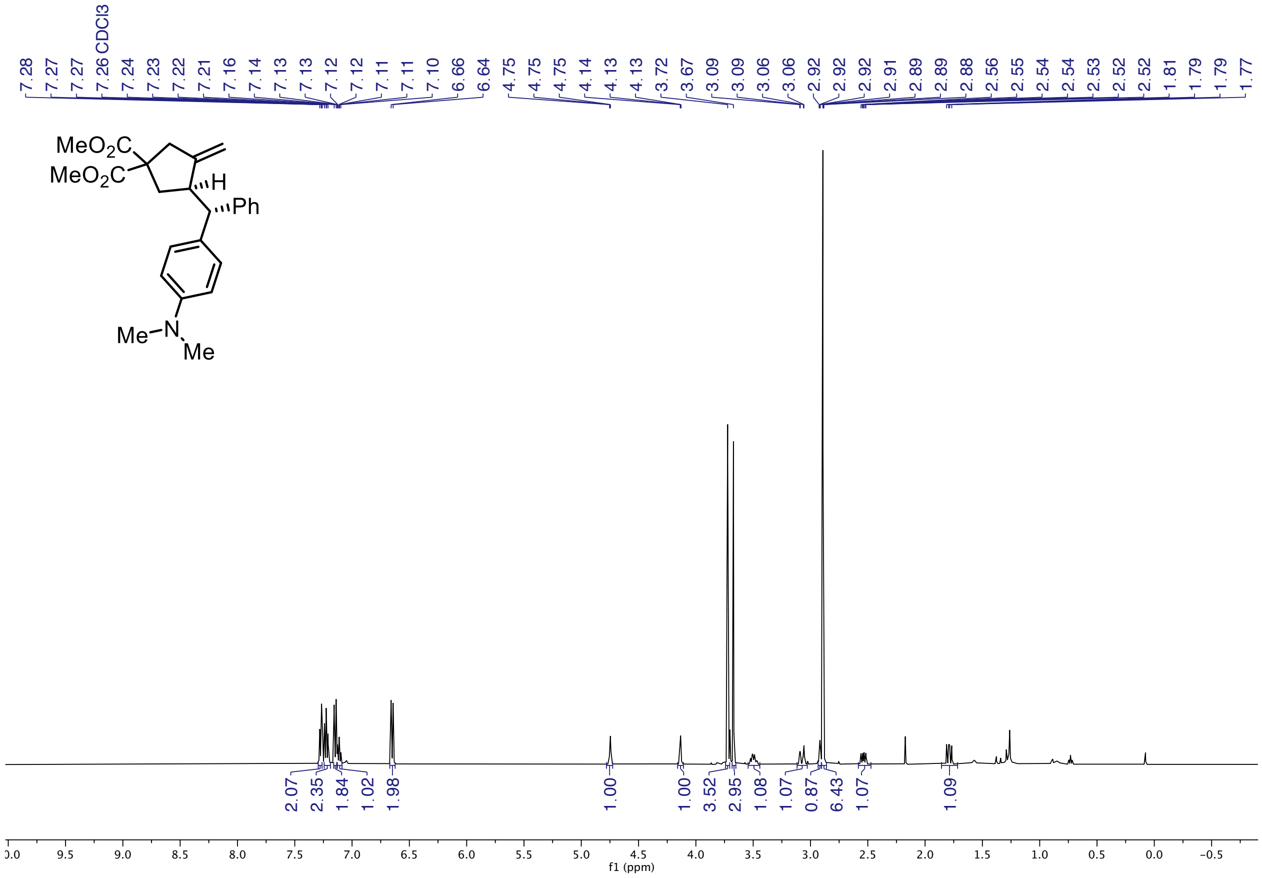

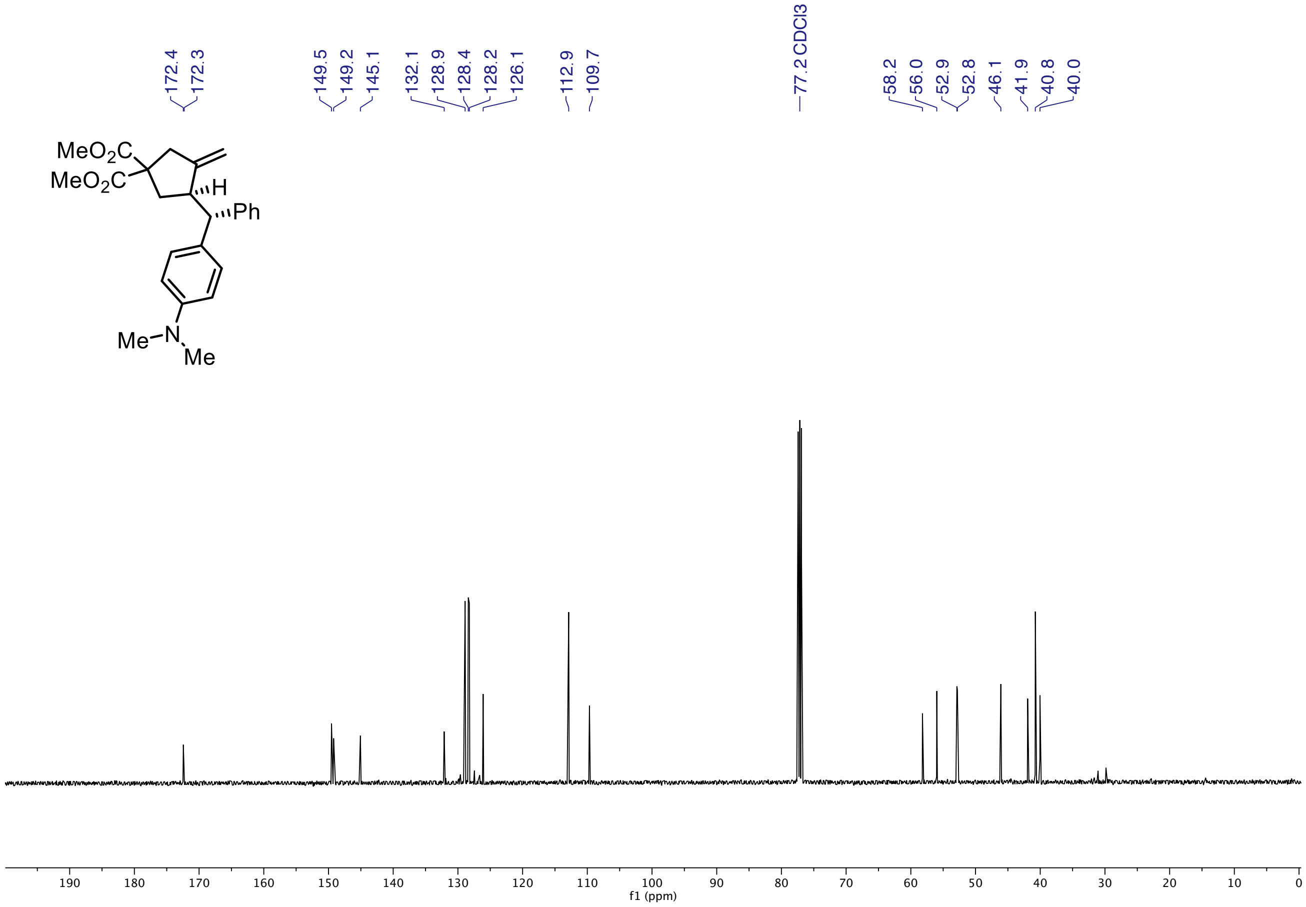

Compound **2i** ^{1}H and $^{13}C\{^{1}H\}$ NMR spectra in $CDCl_3$

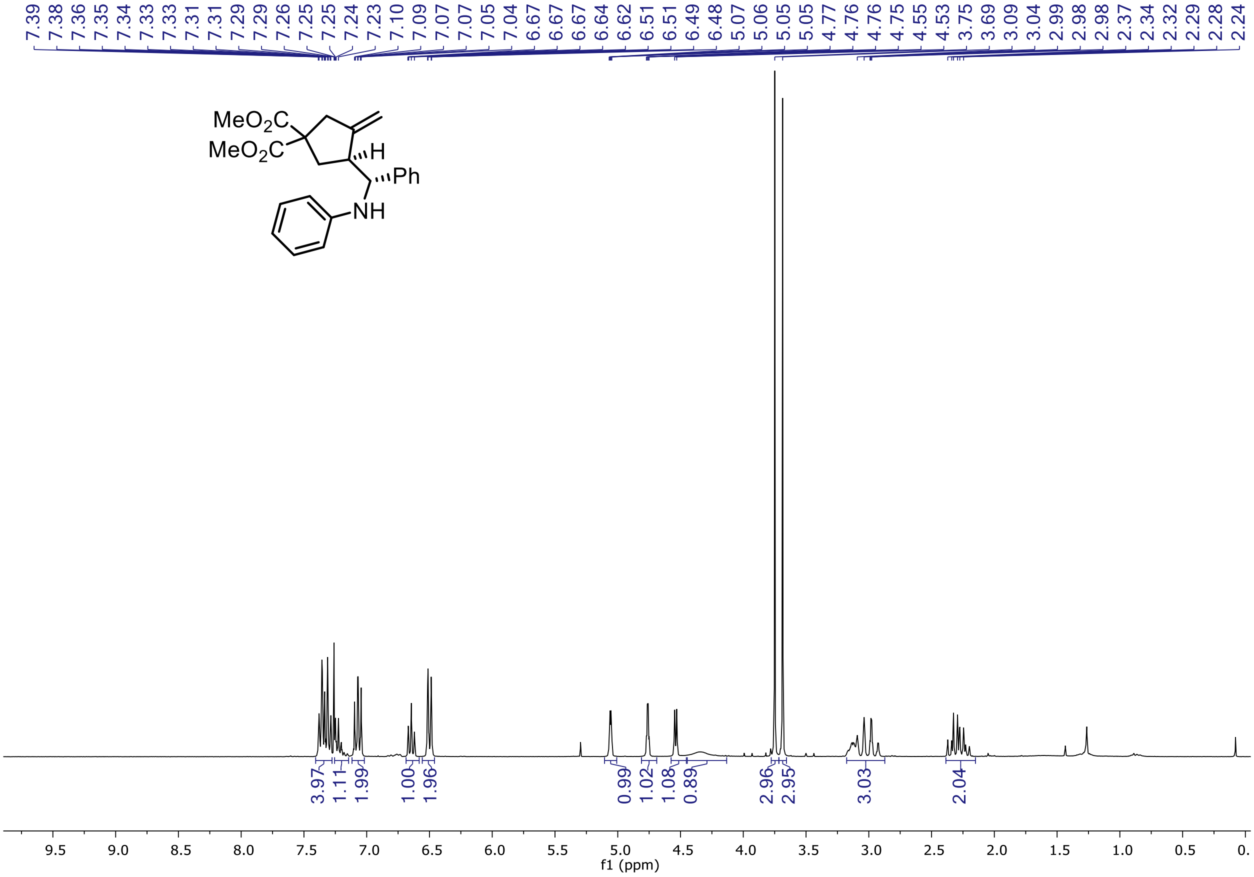

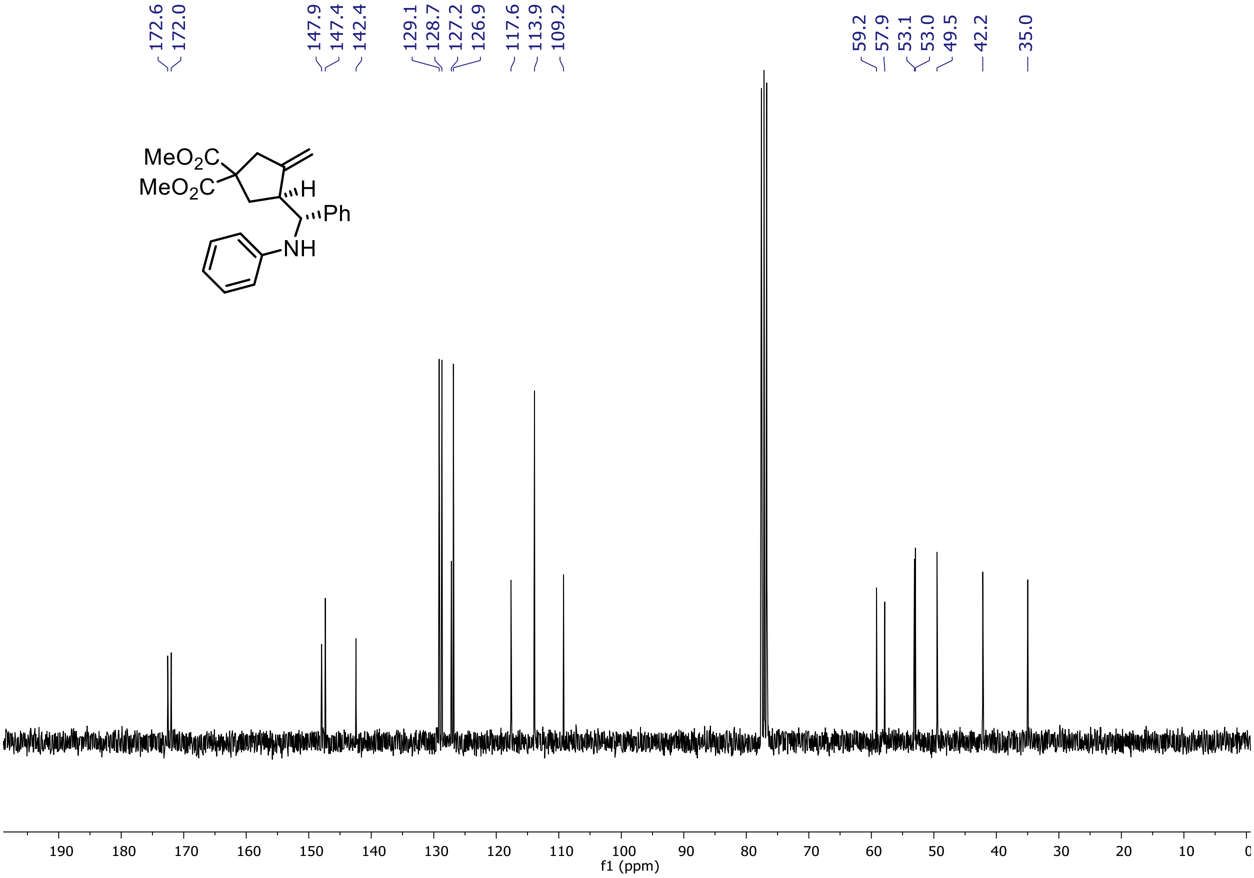

Compound **2j** ^{1}H and ^{13}C{^{1}H} NMR spectra in $CDCl_3$

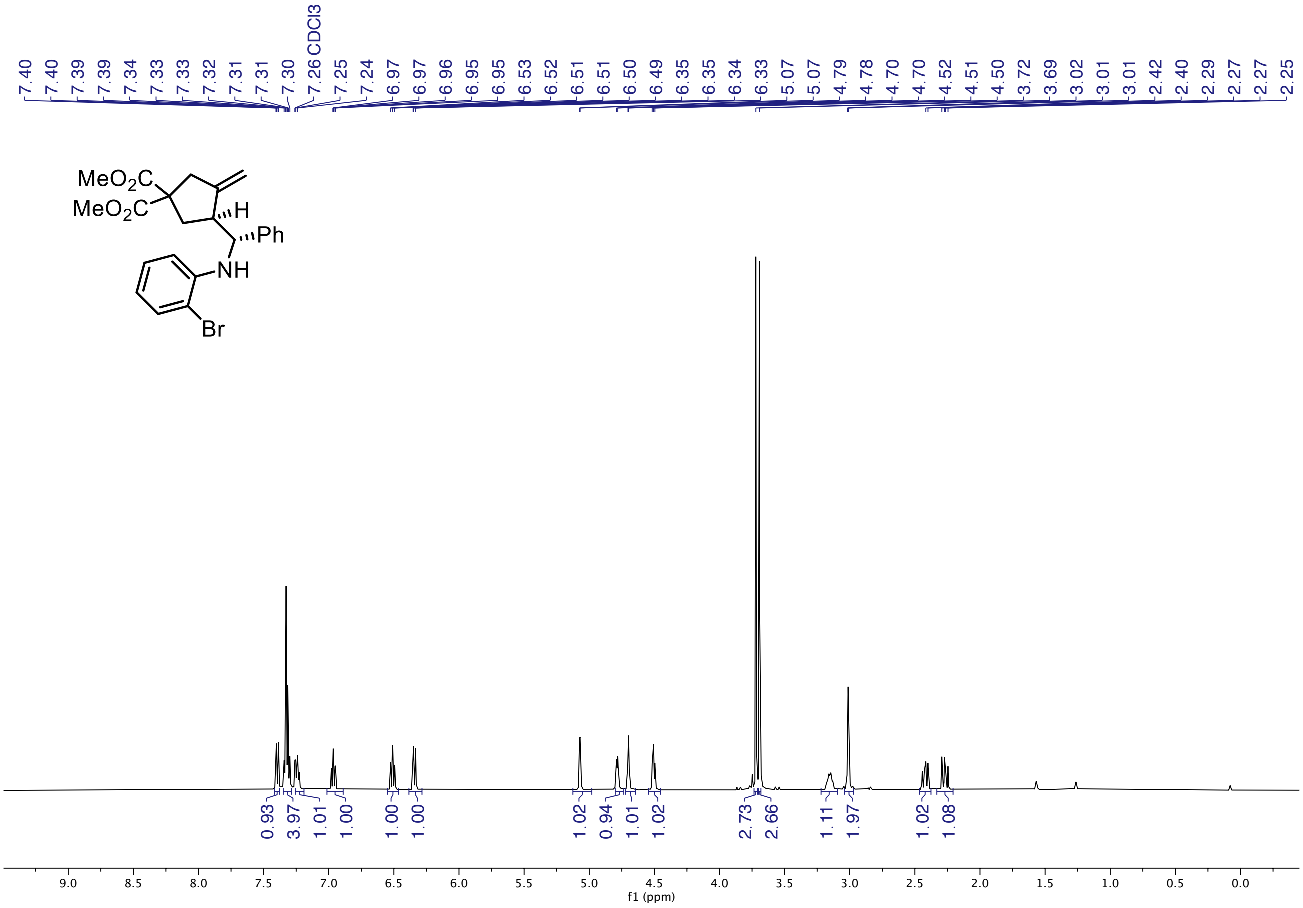

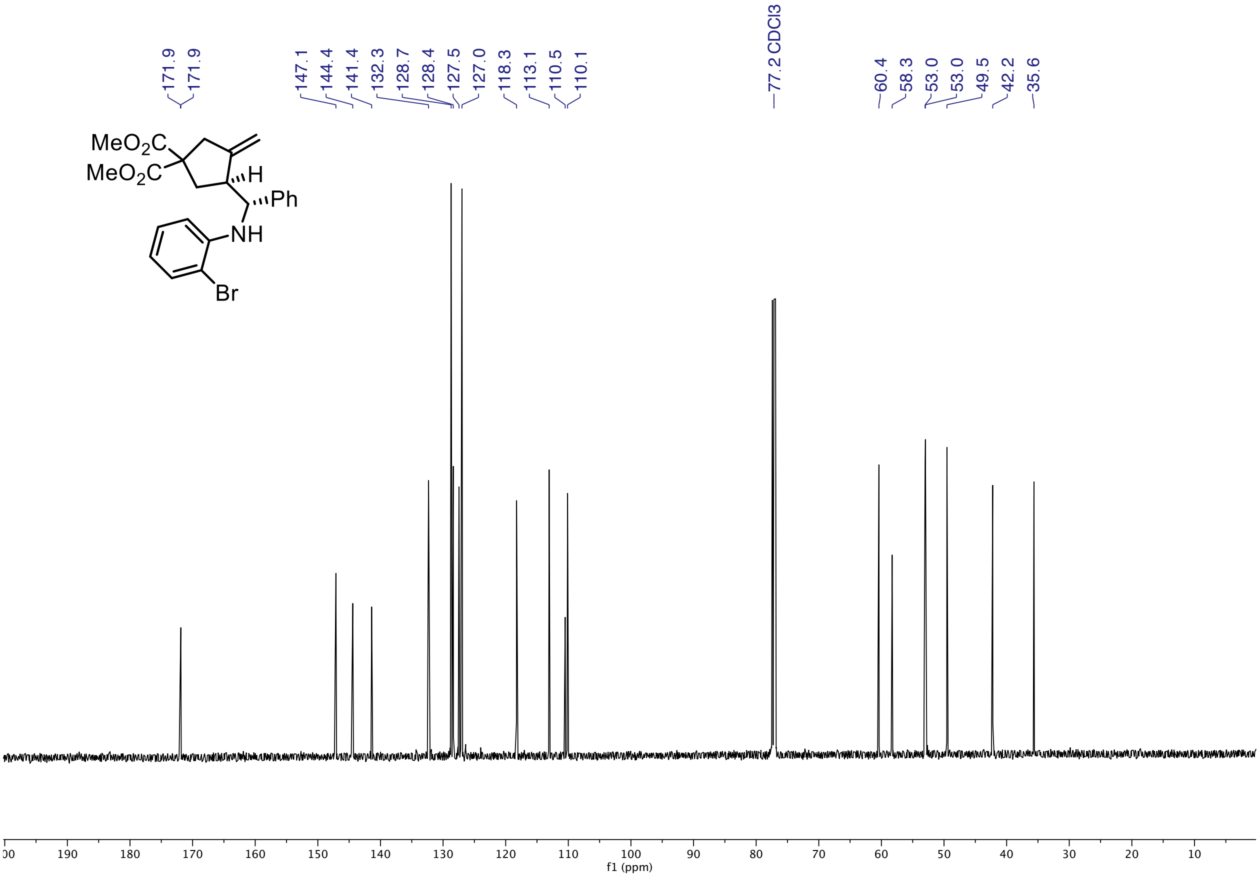

Compound **2k** ^{1}H and $^{13}C\{^{1}H\}$ NMR spectra in $CDCl_3$

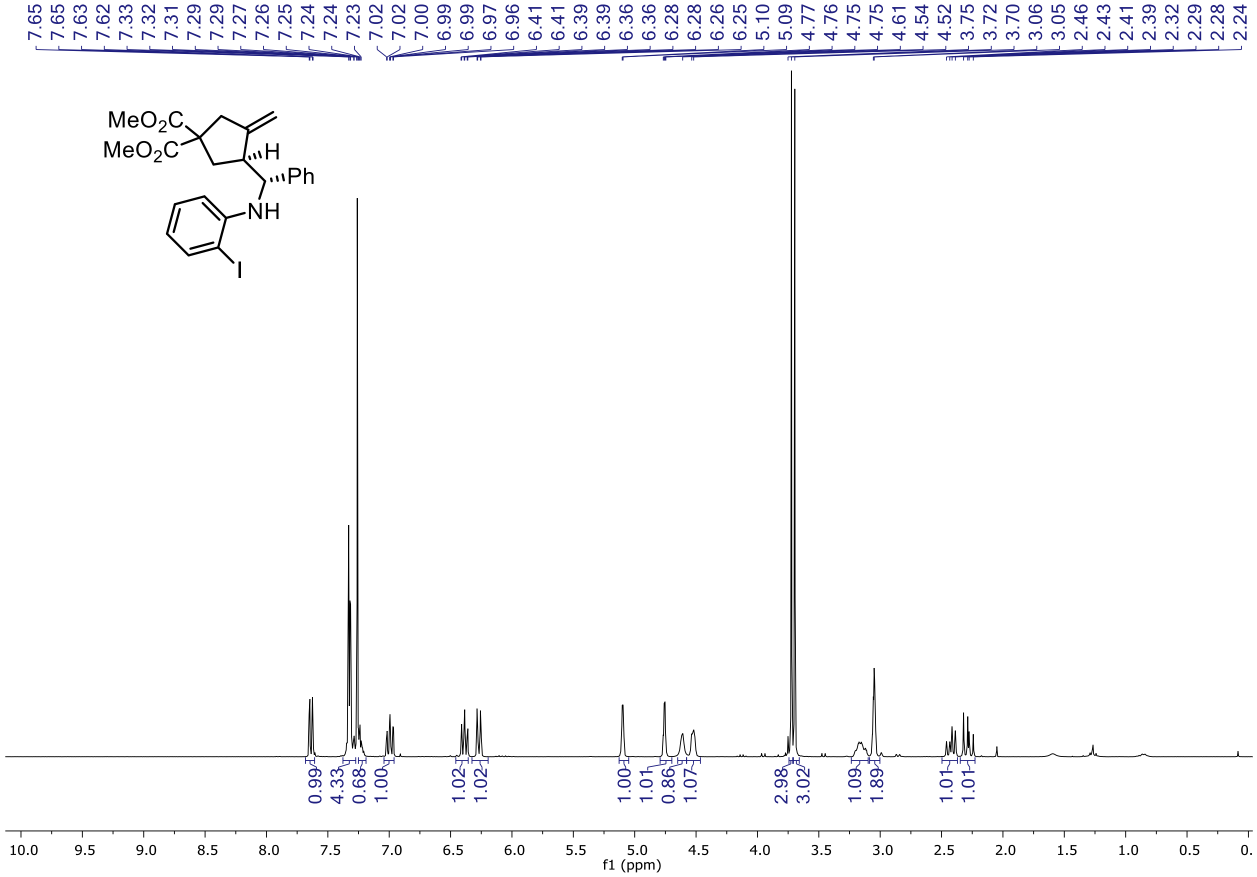

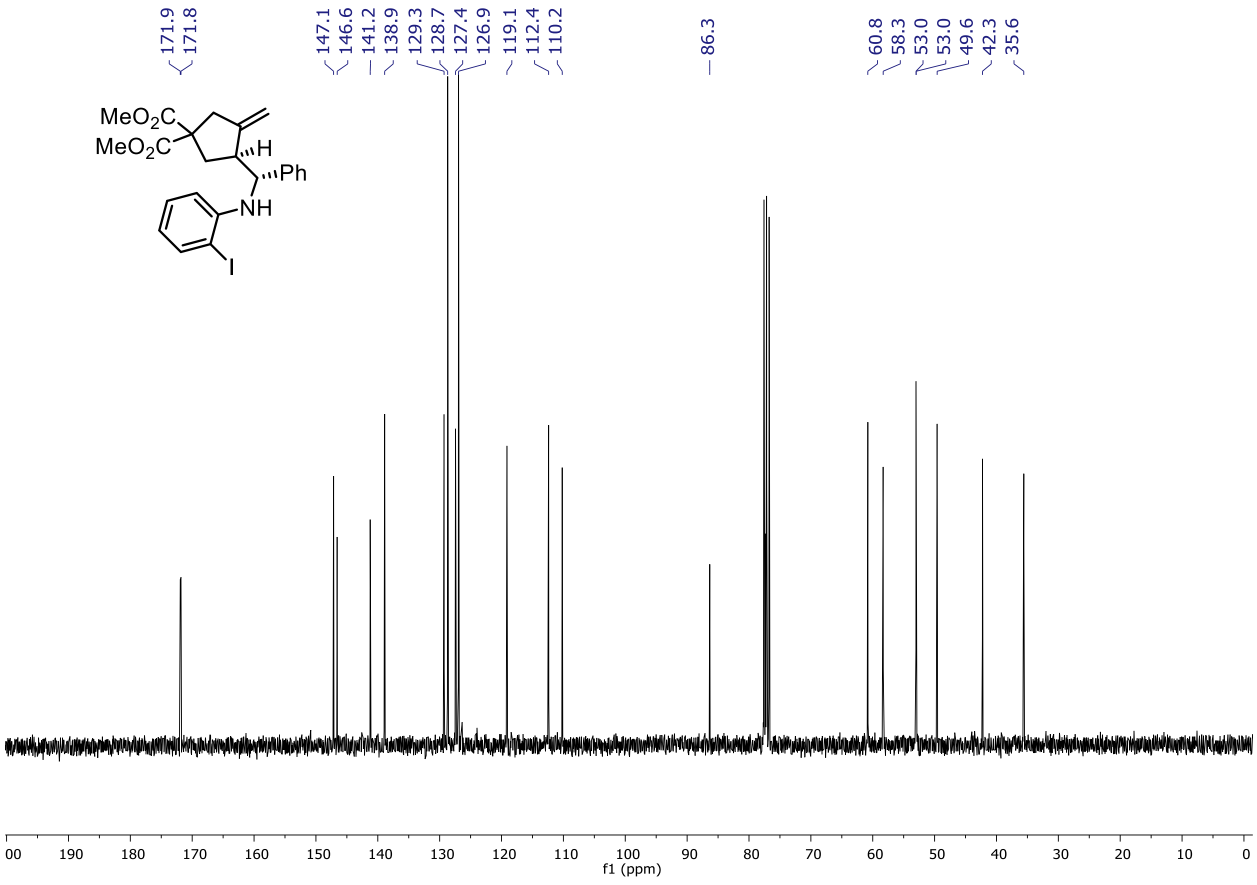

Compound **2l** ^{1}H and ^{13}C{^{1}H} NMR spectra in $CDCl_3$

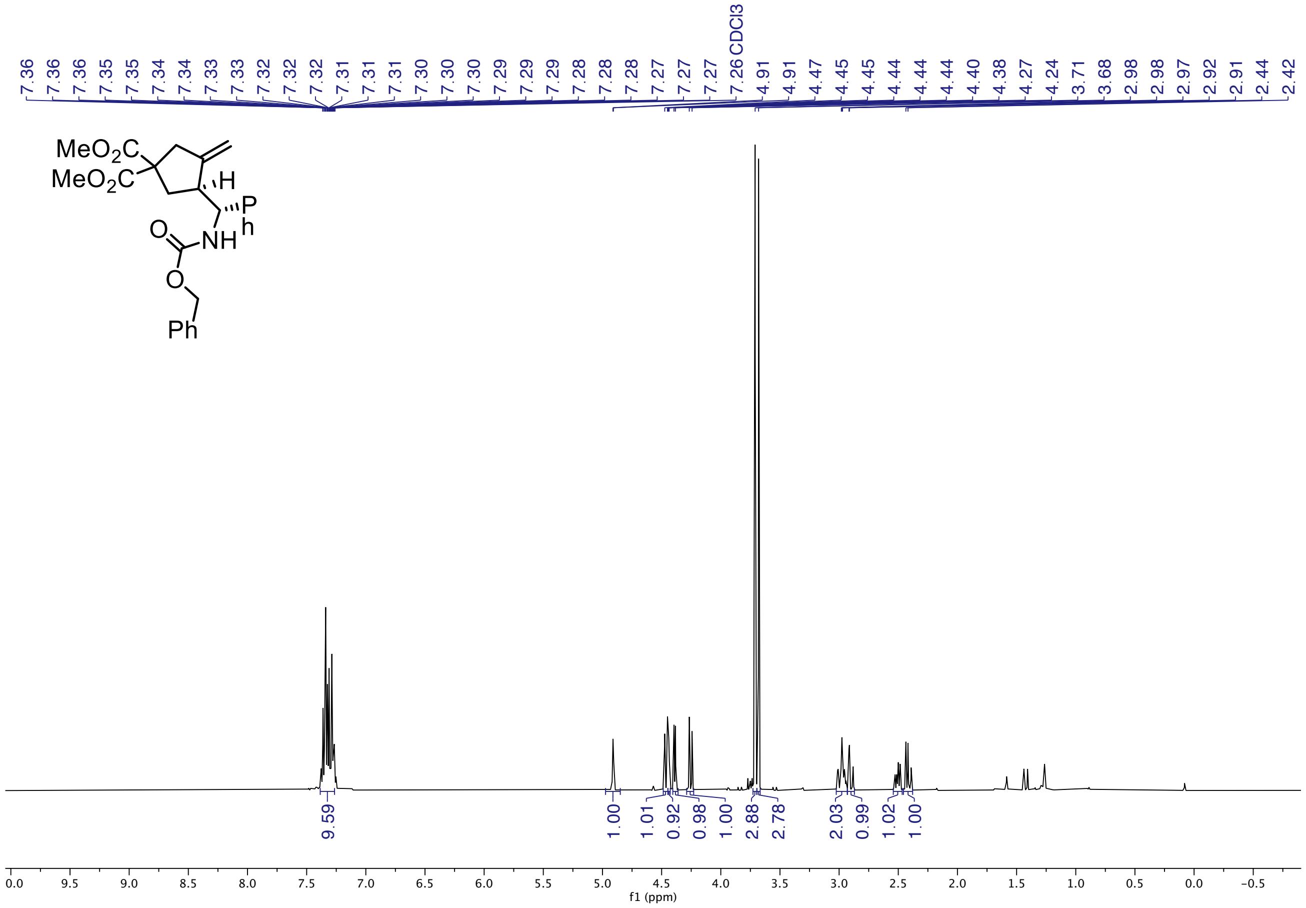

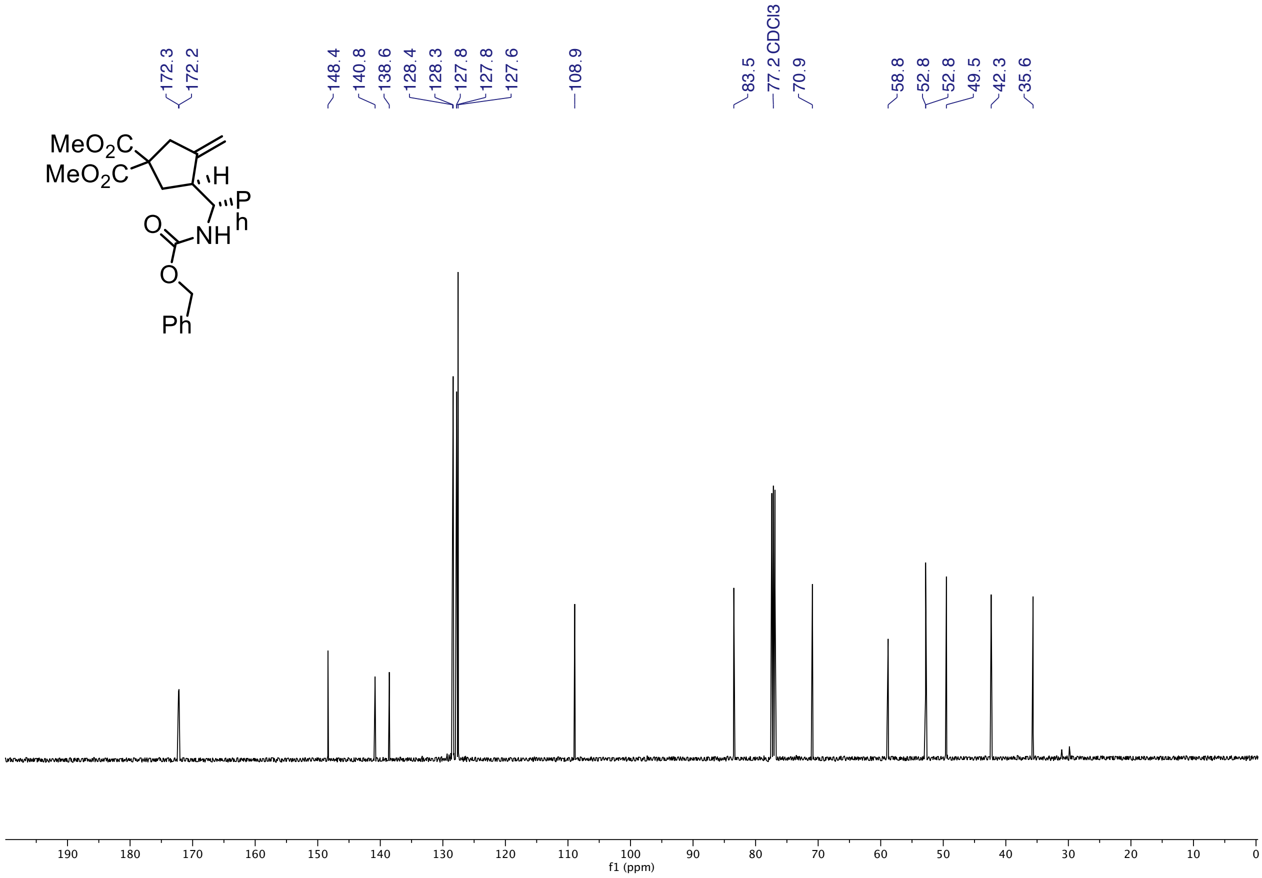

Compound **2m** ^{1}H and ^{13}C{^{1}H} NMR spectra in $CDCl_3$

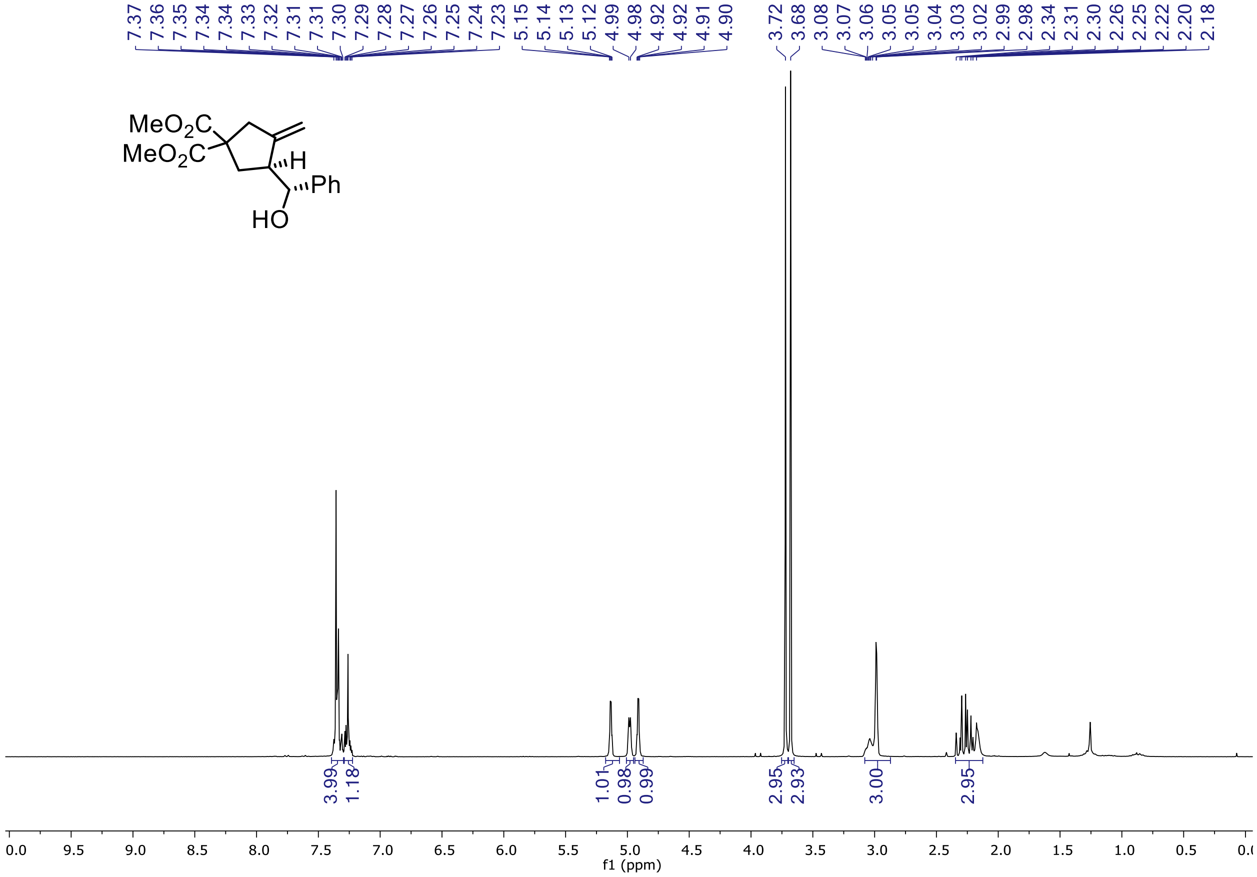

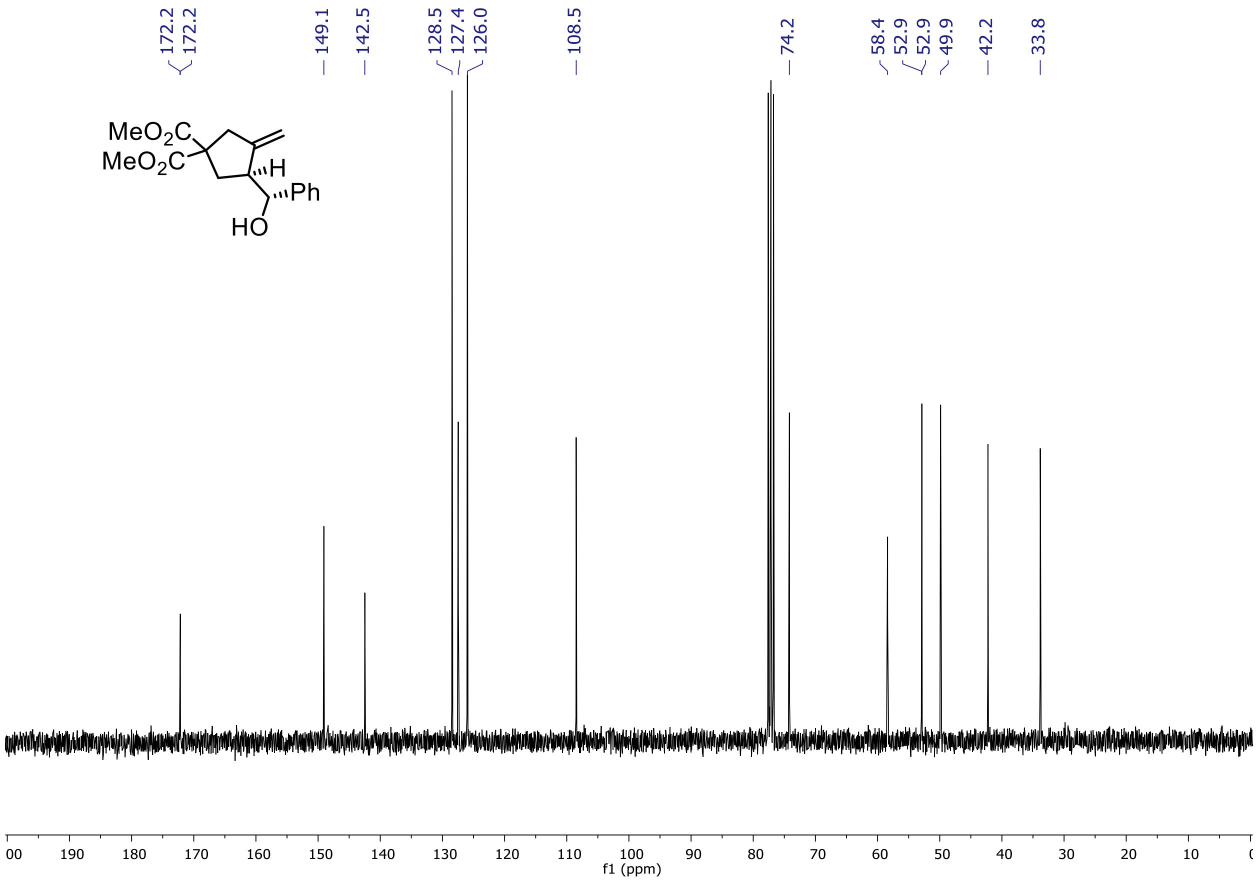

Compound **2n** ^{1}H and $^{13}C\{^{1}H\}$ NMR spectra in $CDCl_3$

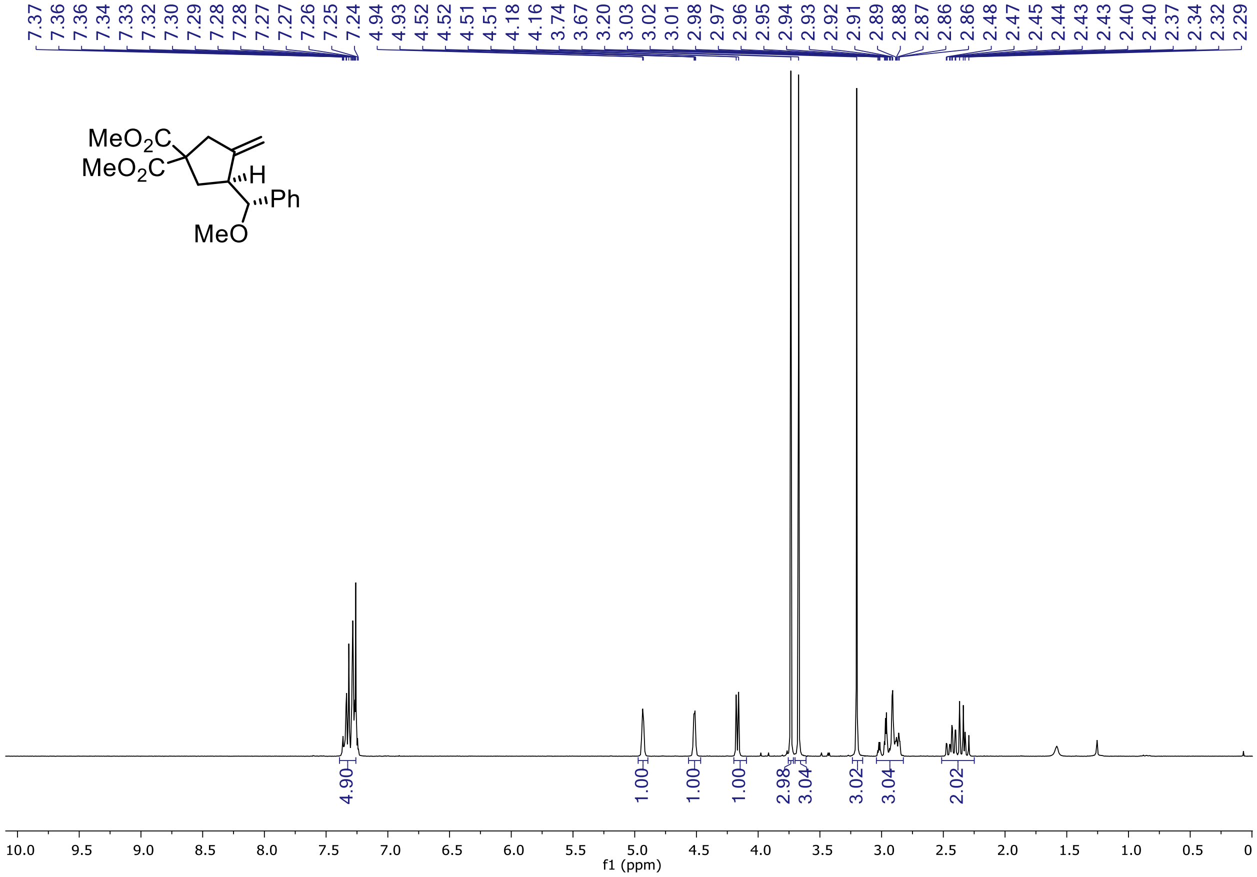

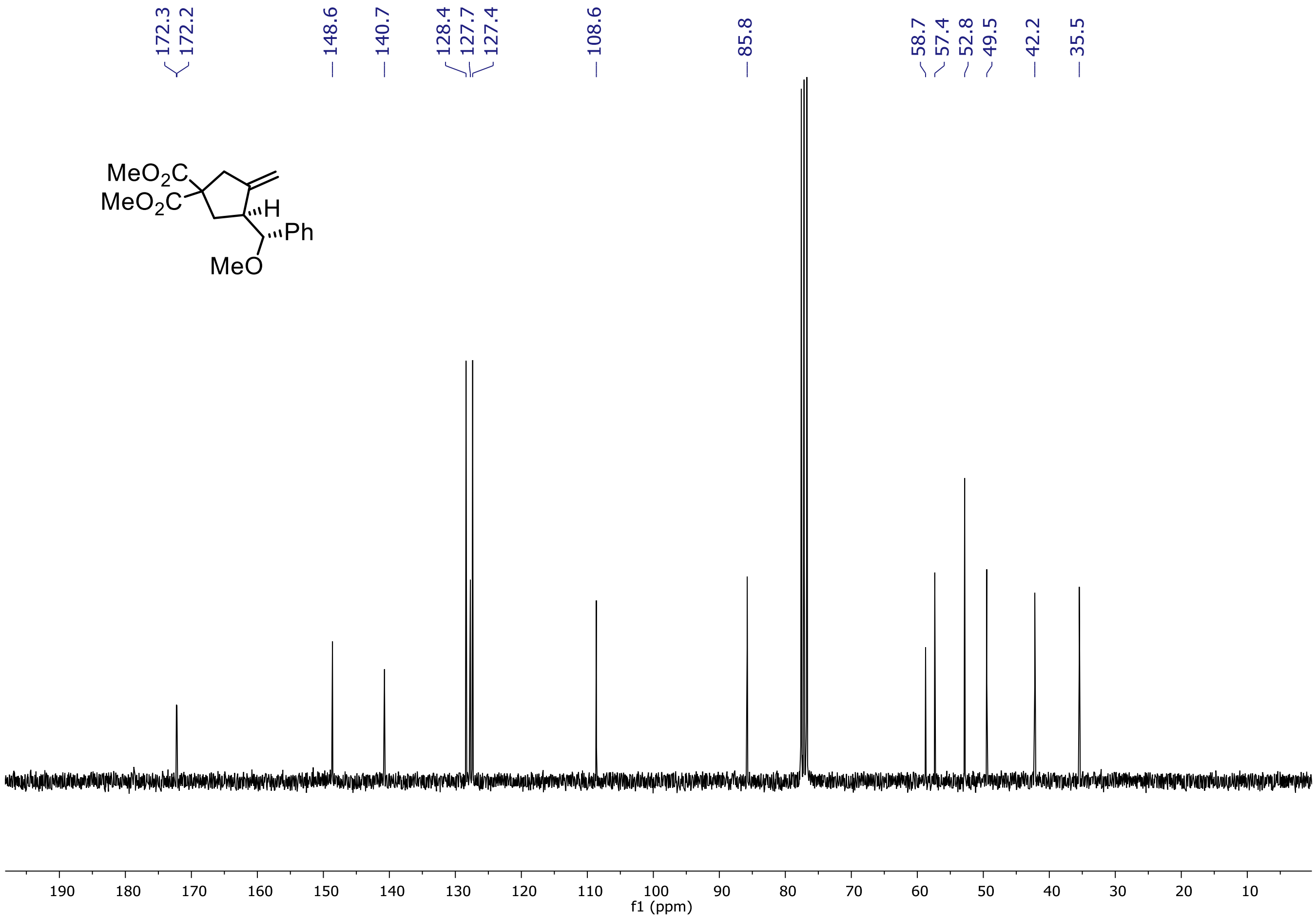

172.3
172.2
148.6
140.7
128.4
127.7
127.4
108.6
85.8
58.7
57.4
52.8
49.5
42.2
35.5
MeO2C
MeO2C
H
Ph
MeO
190 180 170 160 150 140 130 120 110 100 90 80 70 60 50 40 30 20 10
f1 (ppm)

Compound **2o** ^{1}H and $^{13}C\{^{1}H\}$ NMR spectra in $CDCl_3$

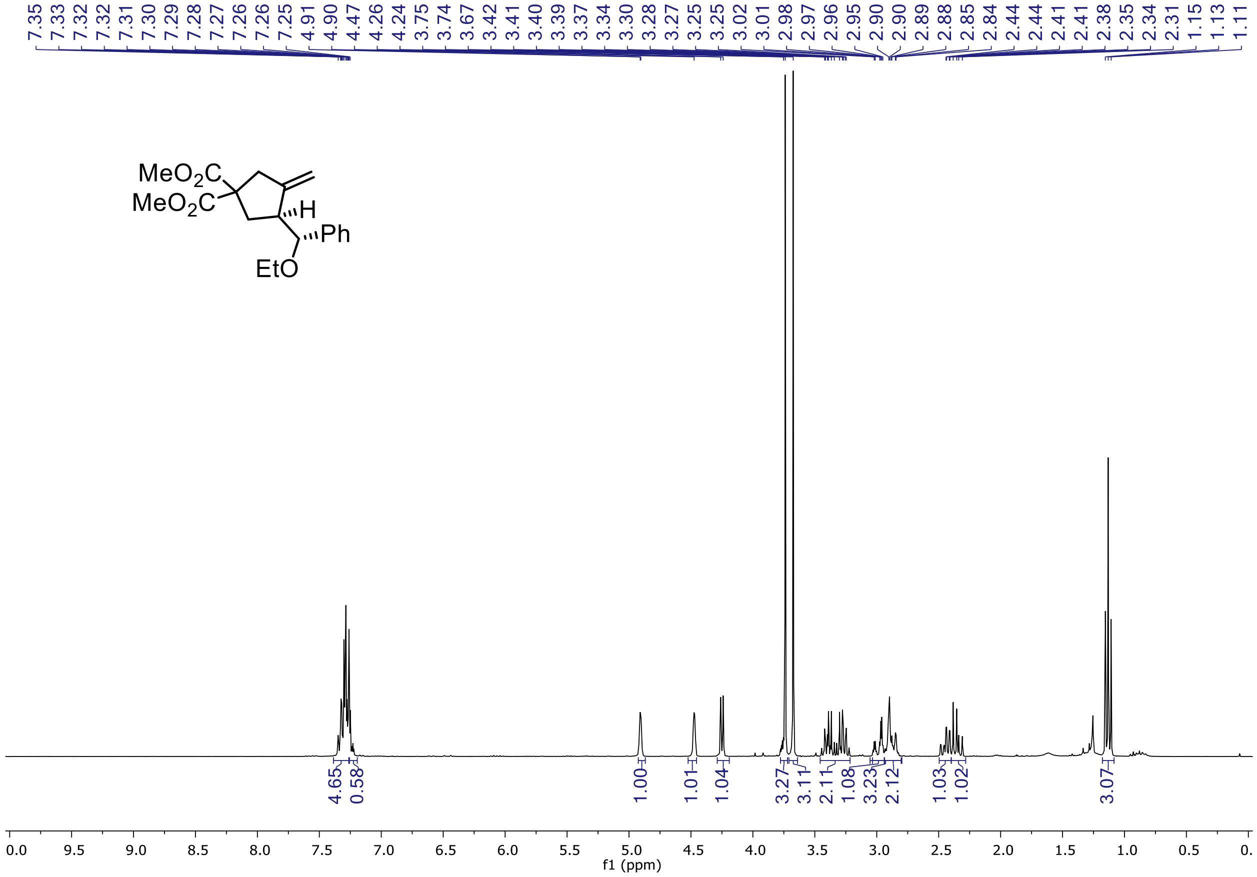

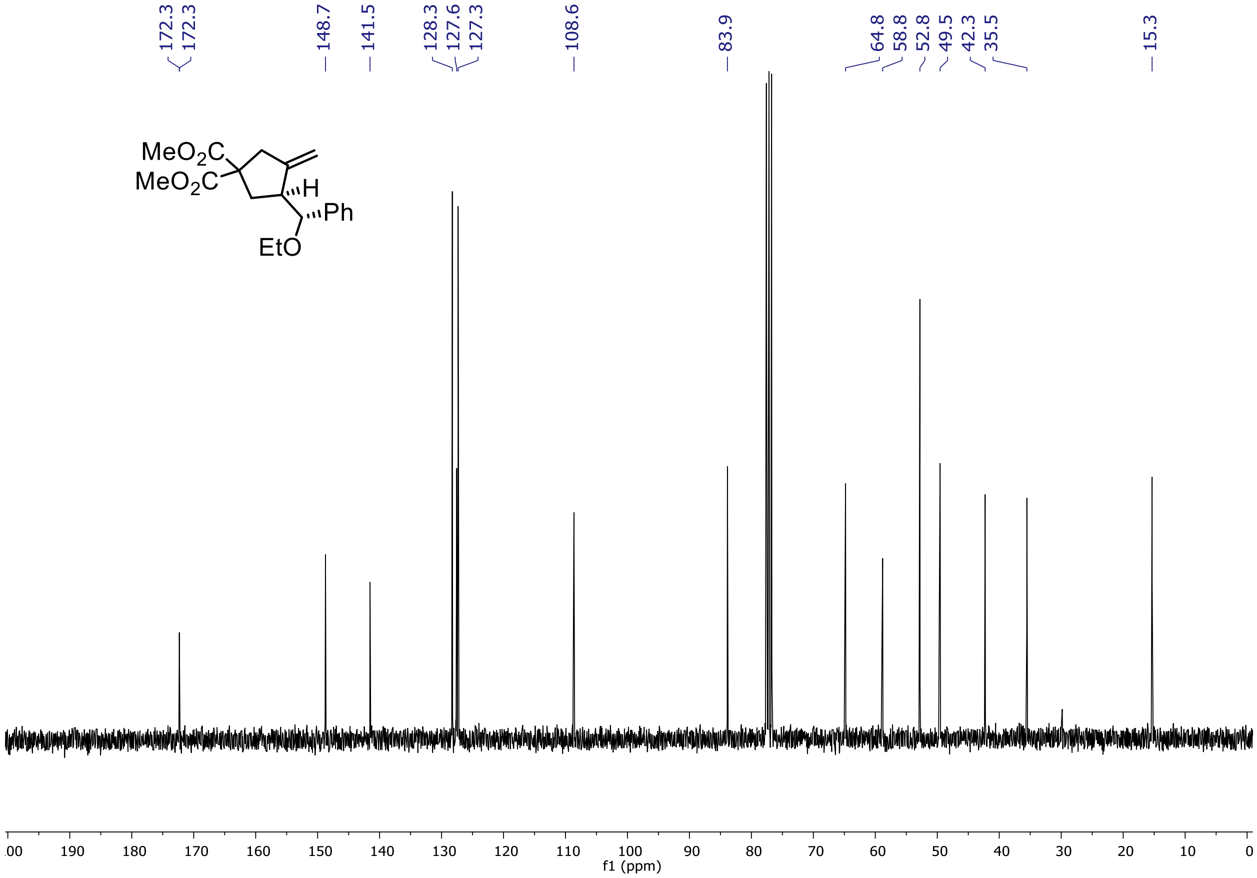

Compound **2p** ^{1}H and $^{13}C\{^{1}H\}$ NMR spectra in $CDCl_3$

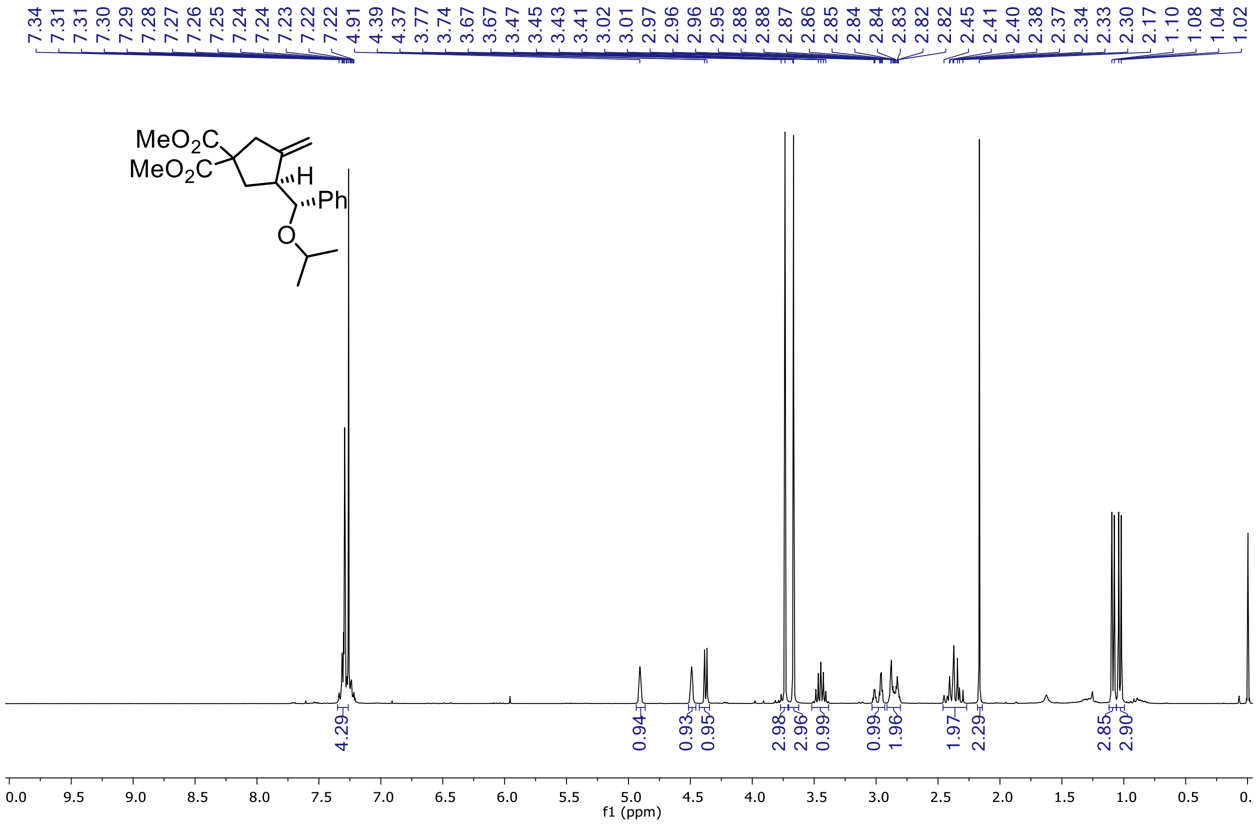

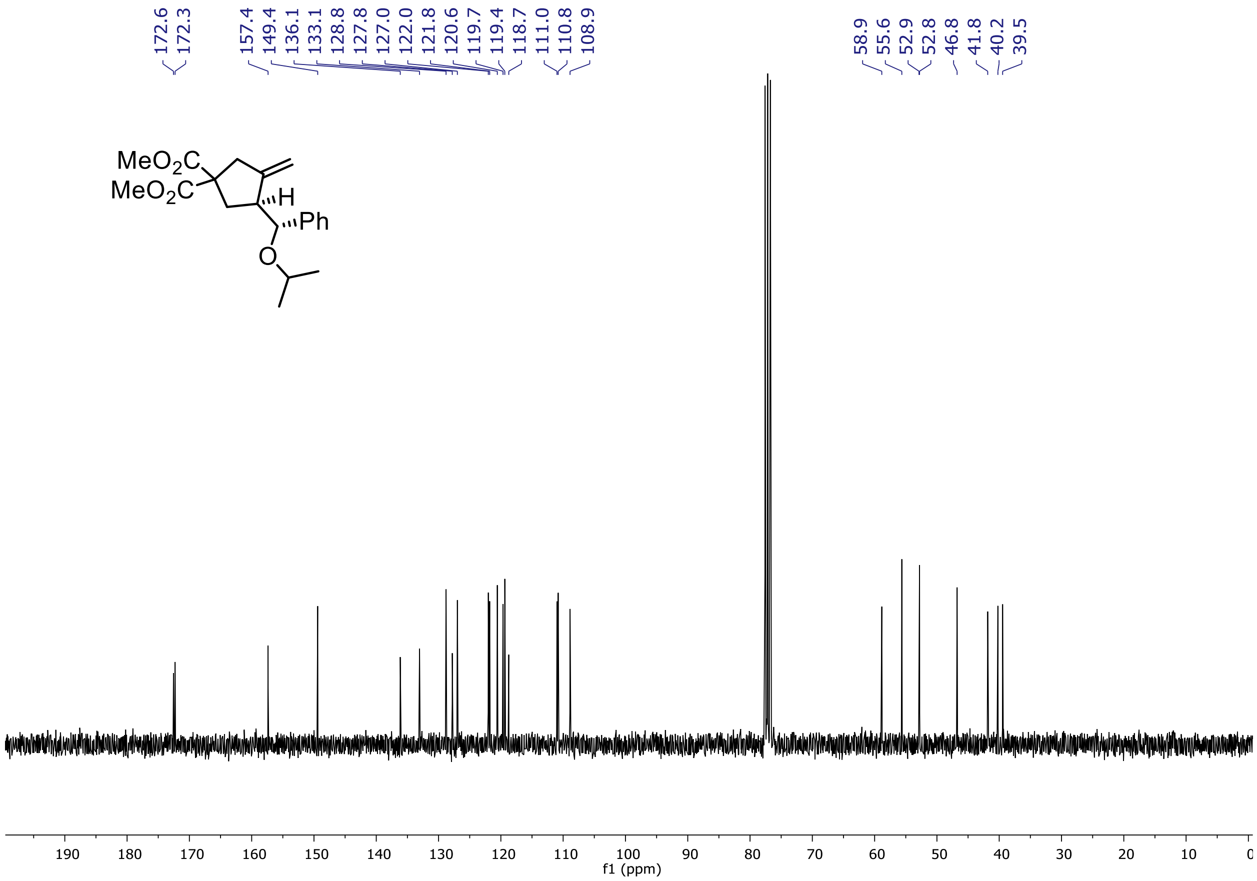

Compound **2q** ^{1}H and $^{13}C\{^{1}H\}$ NMR spectra in $CDCl_3$

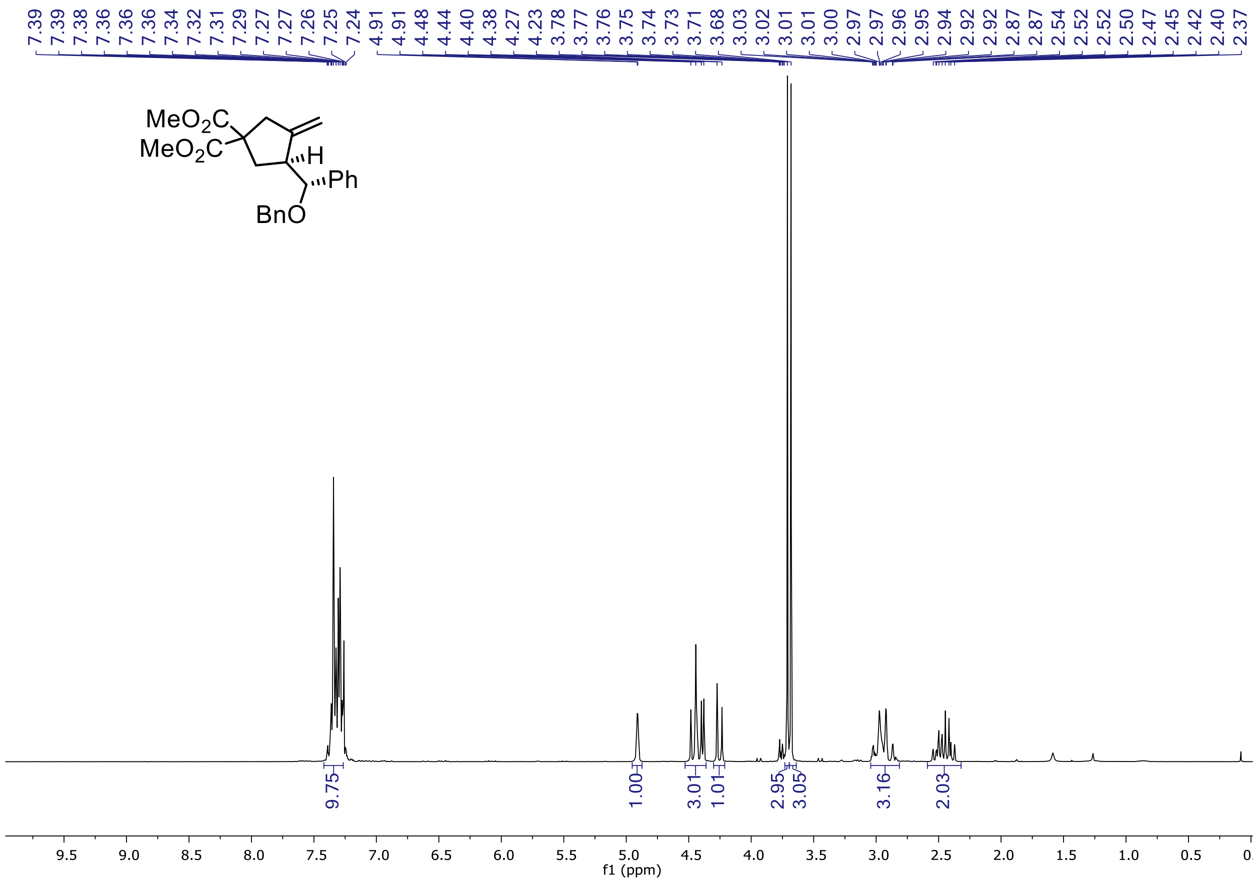

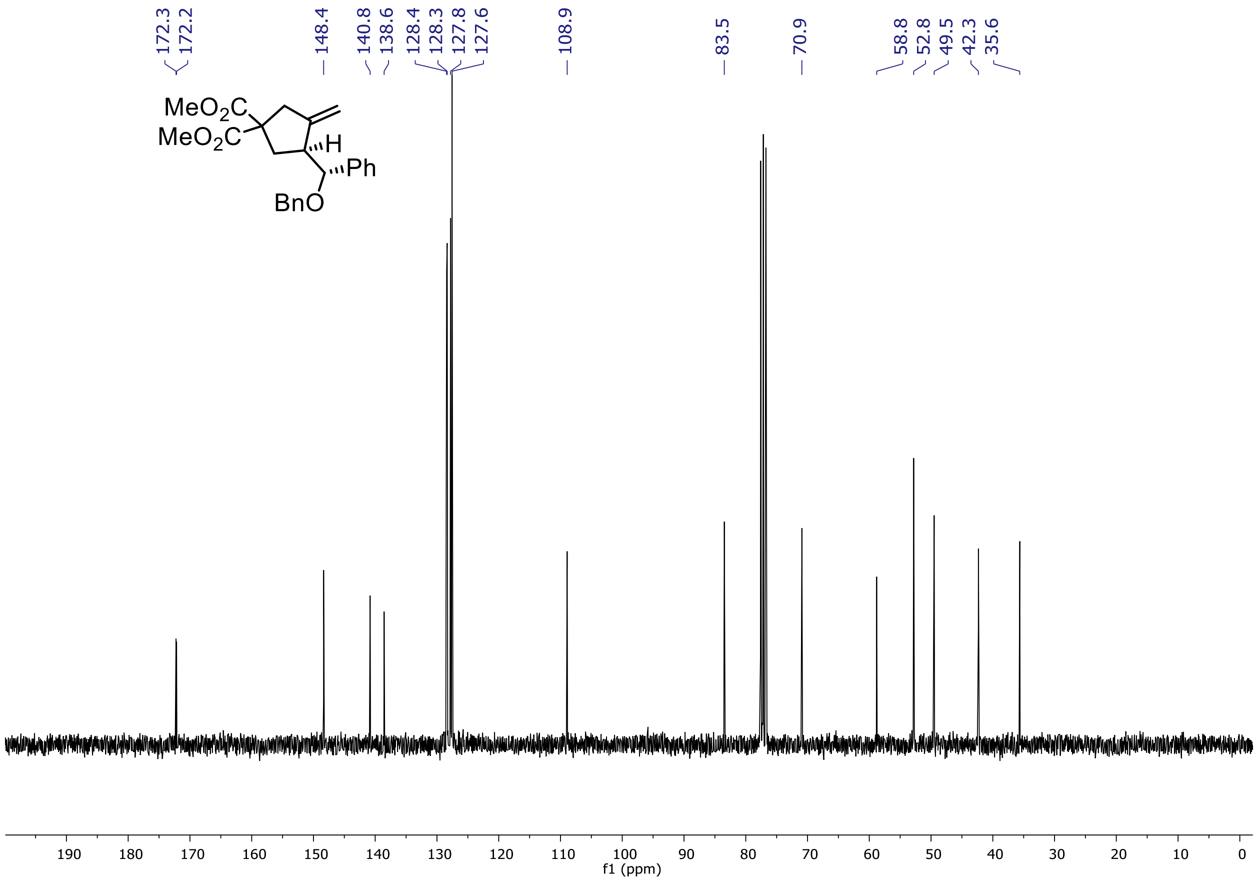

Compound **2r** ^{1}H and $^{13}C\{^{1}H\}$ NMR spectra in $CDCl_3$

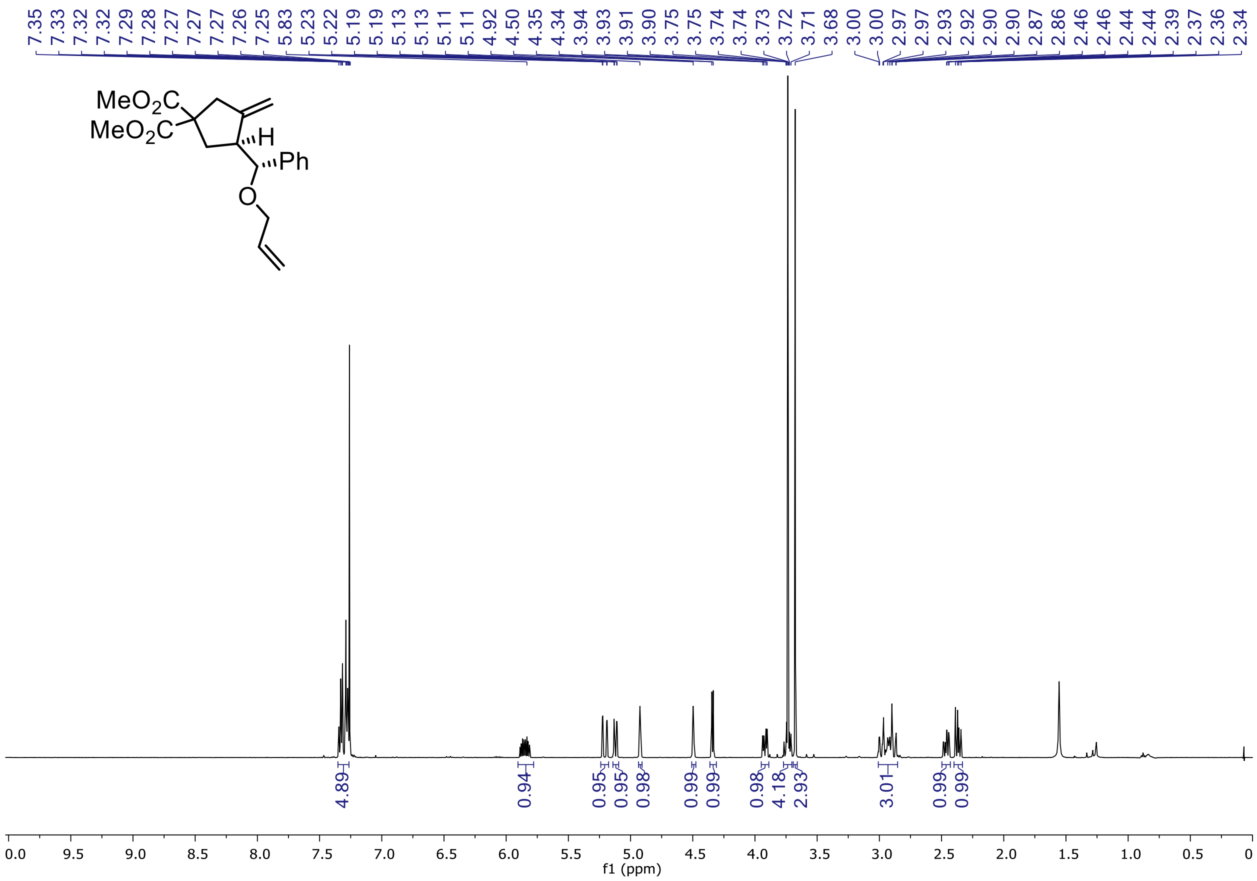

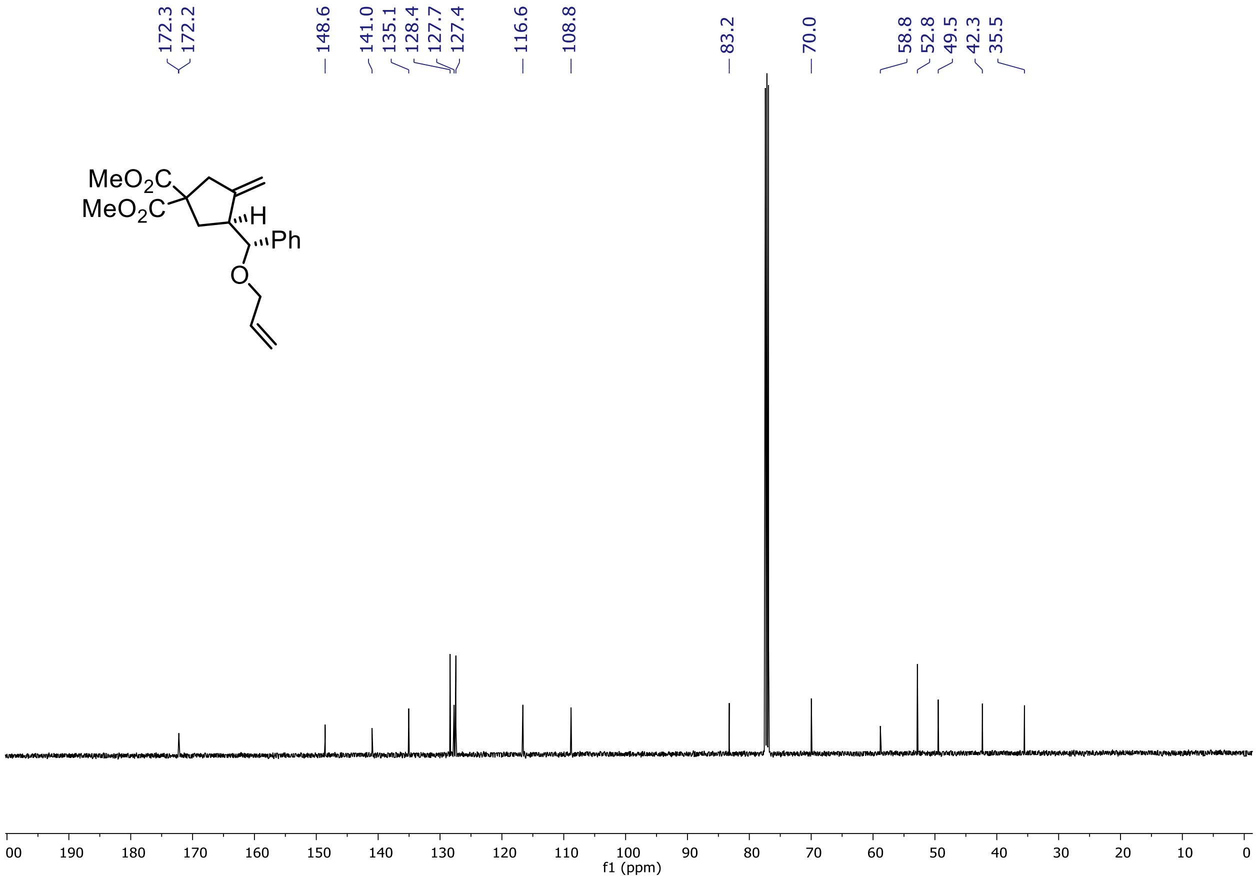

Compound **2s** ^{1}H and ^{13}C{^{1}H} NMR spectra in $CDCl_3$

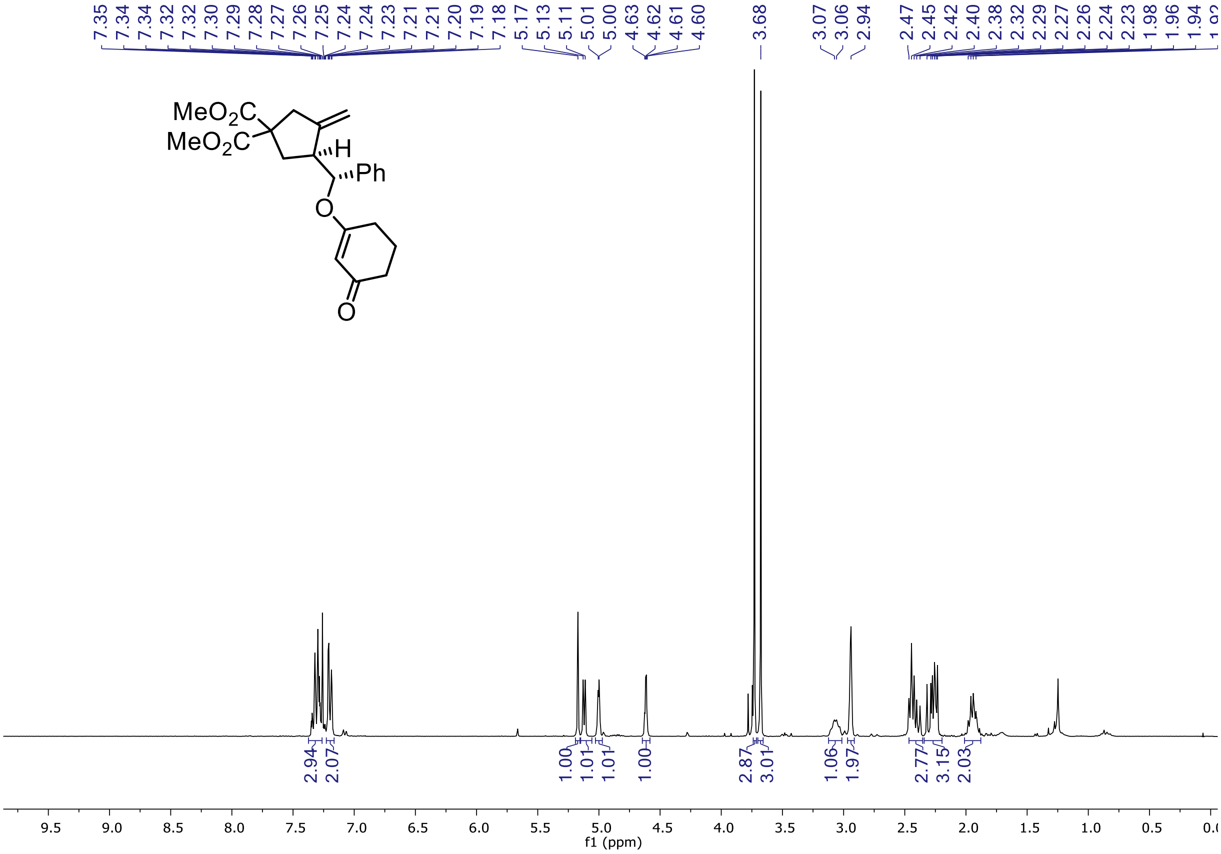

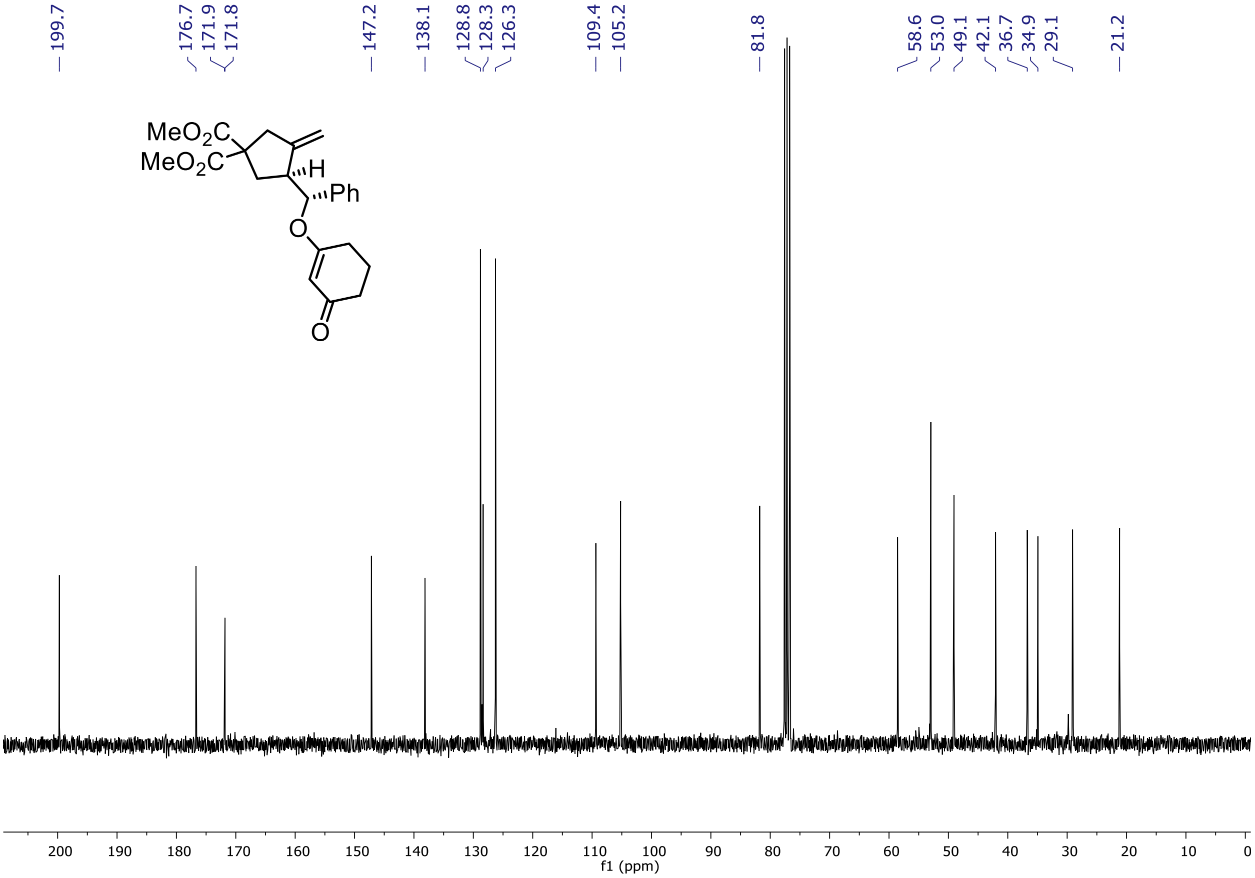

Compound **2t** ^{1}H, ^{19}F and $^{13}C\{^{1}H\}$ NMR spectra in $CDCl_3$

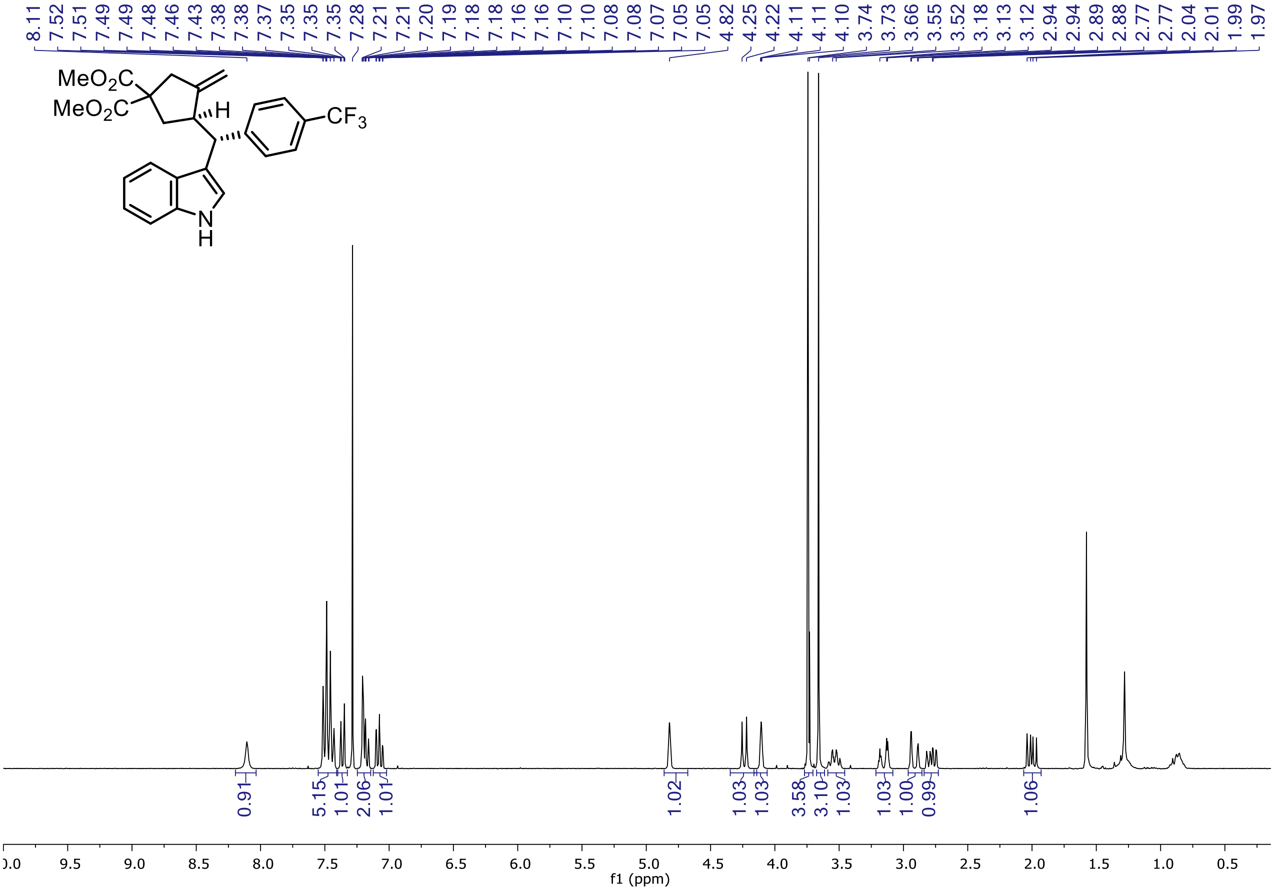

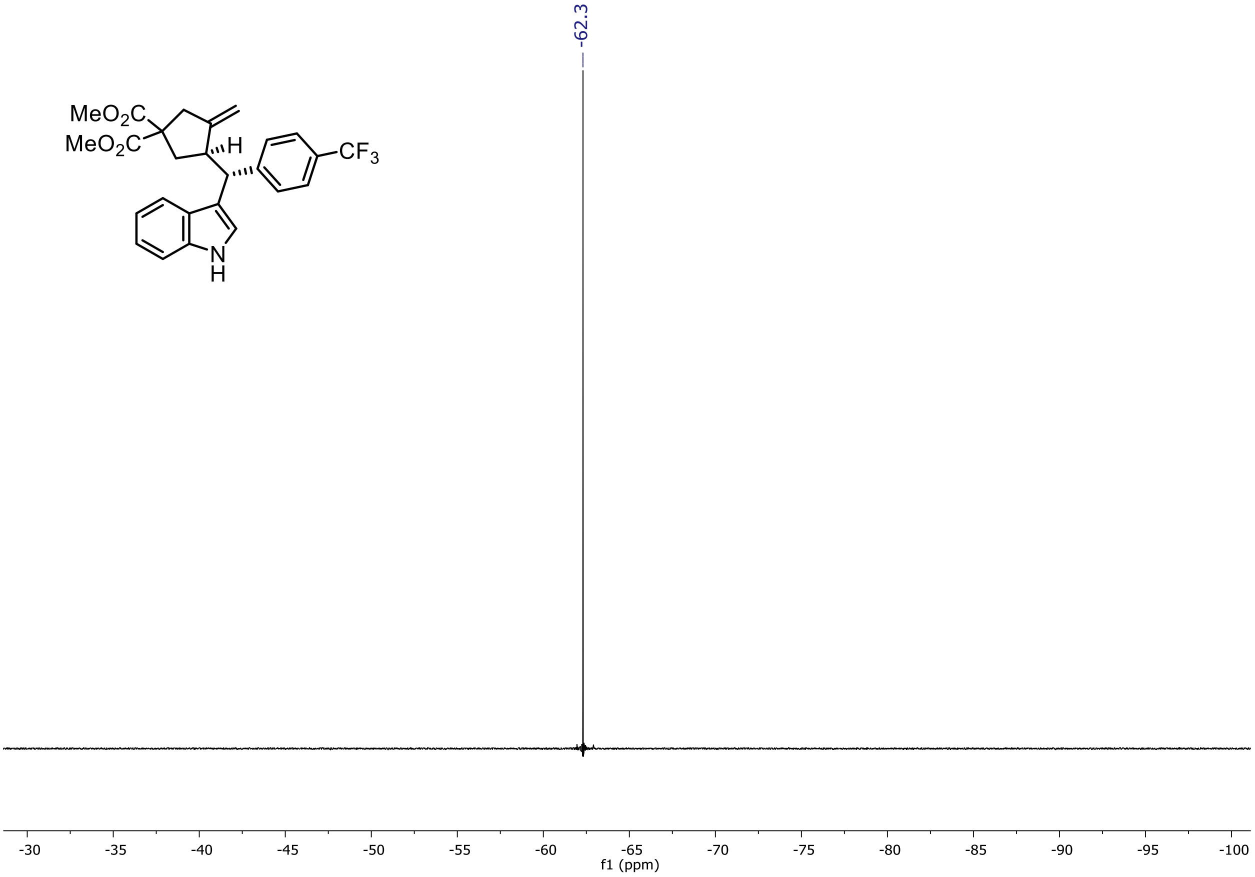

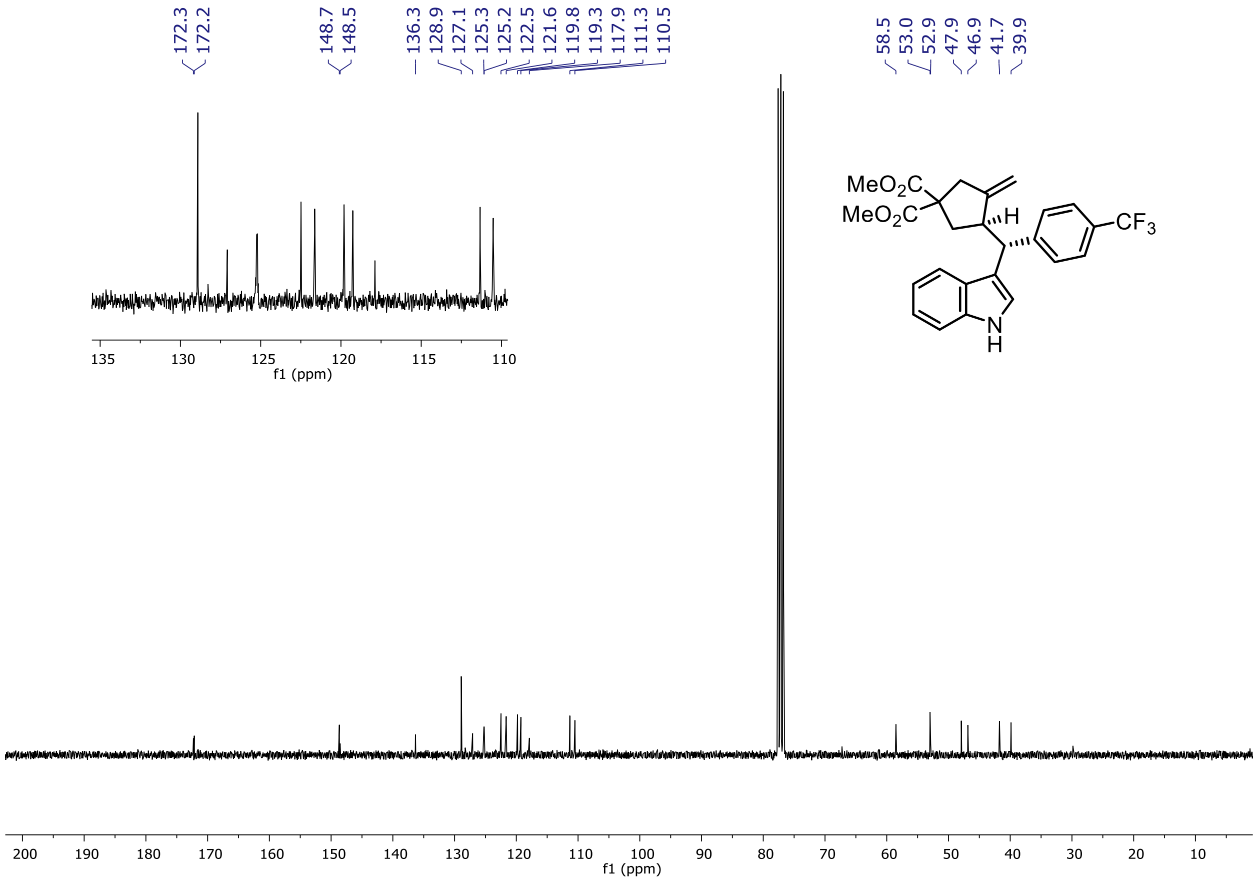

172.3
172.2
148.7
148.5
136.3
128.9
127.1
125.3
125.2
122.5
121.6
119.8
119.3
117.9
111.3
110.5
58.5
53.0
52.9
47.9
46.9
41.7
39.9
MeO2C
MeO2C
H
CF3
N
H
135
130
125
120
115
110
f1 (ppm)
200
190
180
170
160
150
140
130
120
110
100
90
80
70
60
50
40
30
20
10
f1 (ppm)

Compound **2u** ^{1}H and $^{13}C\{^{1}H\}$ NMR spectra in $CDCl_3$

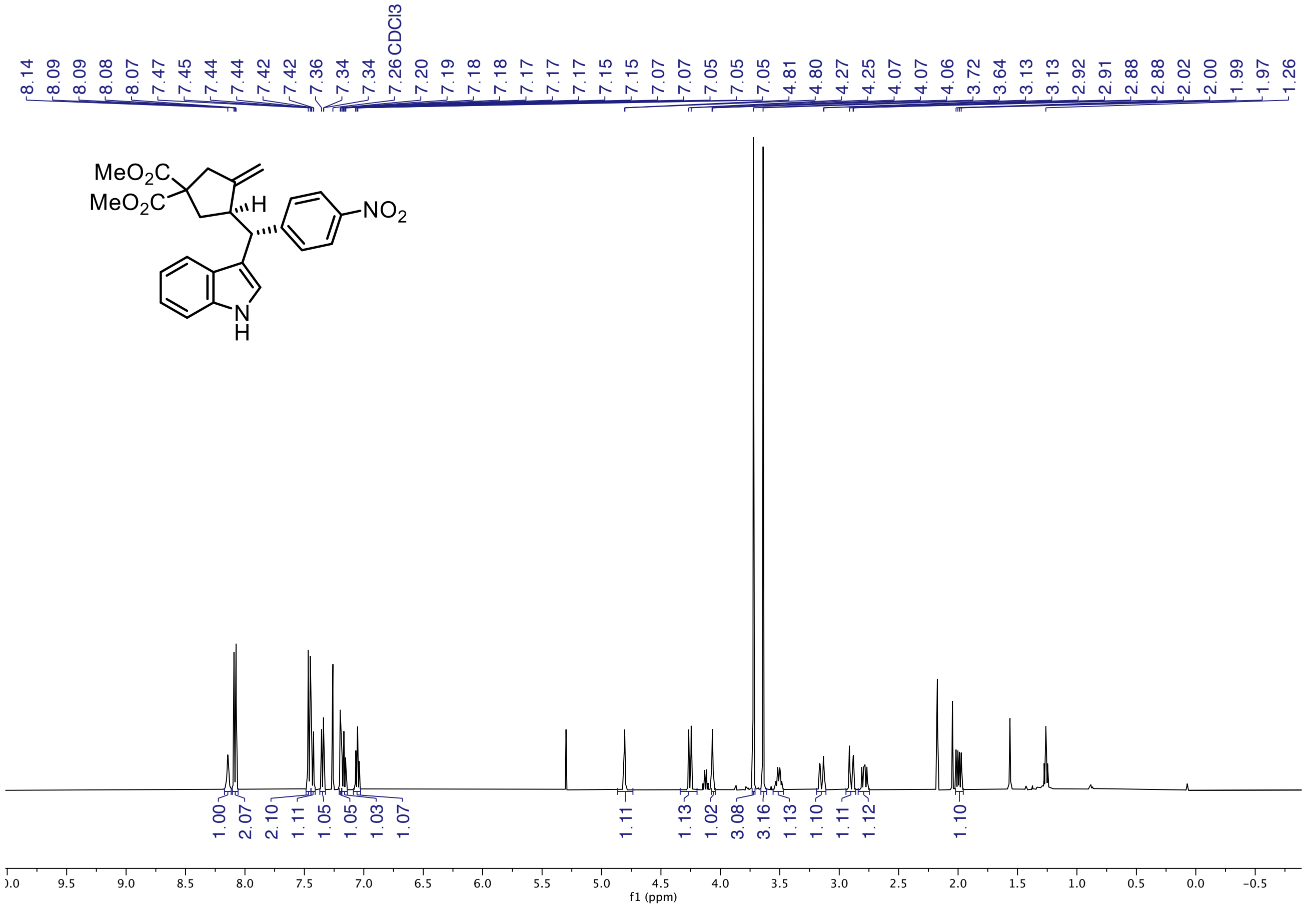

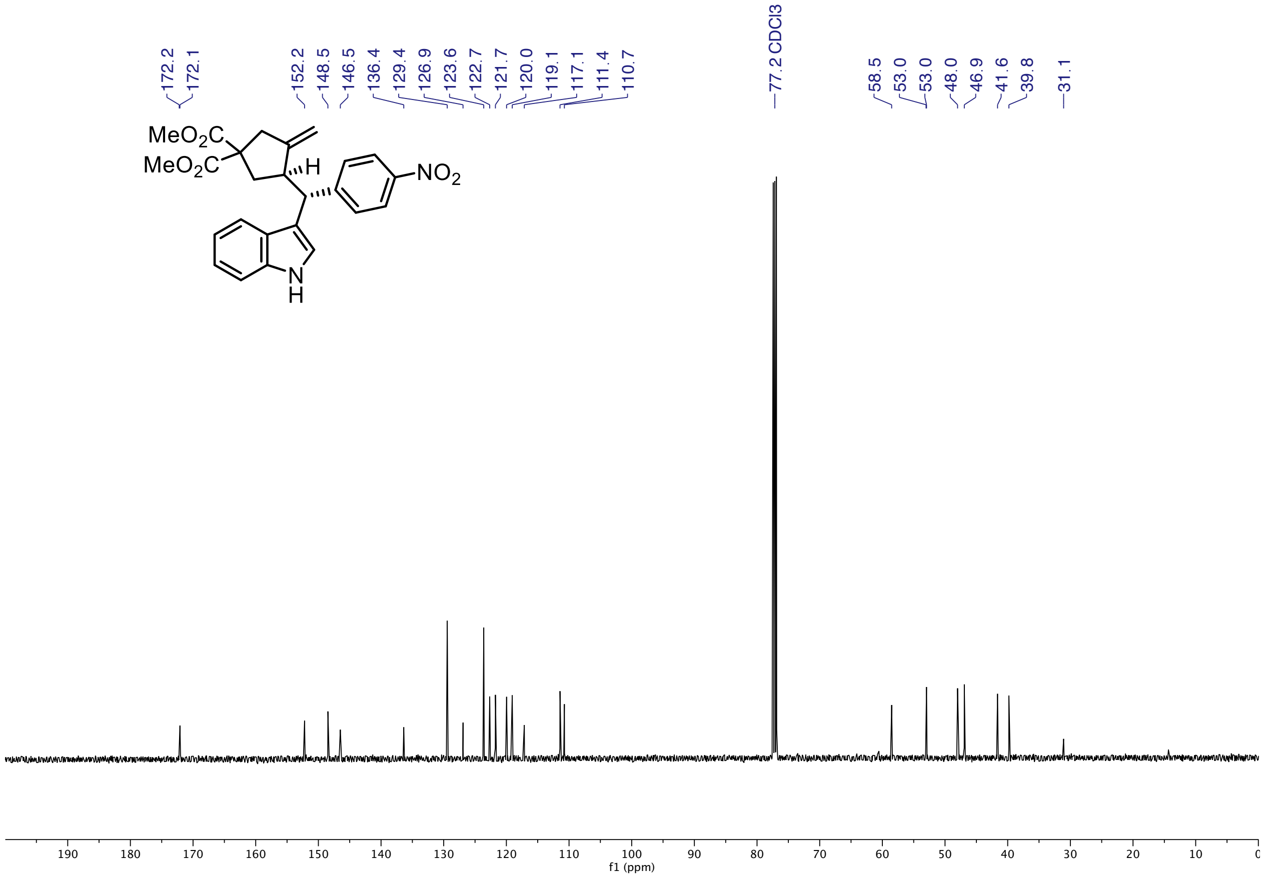

Compound **2v** ^{1}H and $^{13}C\{^{1}H\}$ NMR spectra in $CDCl_3$

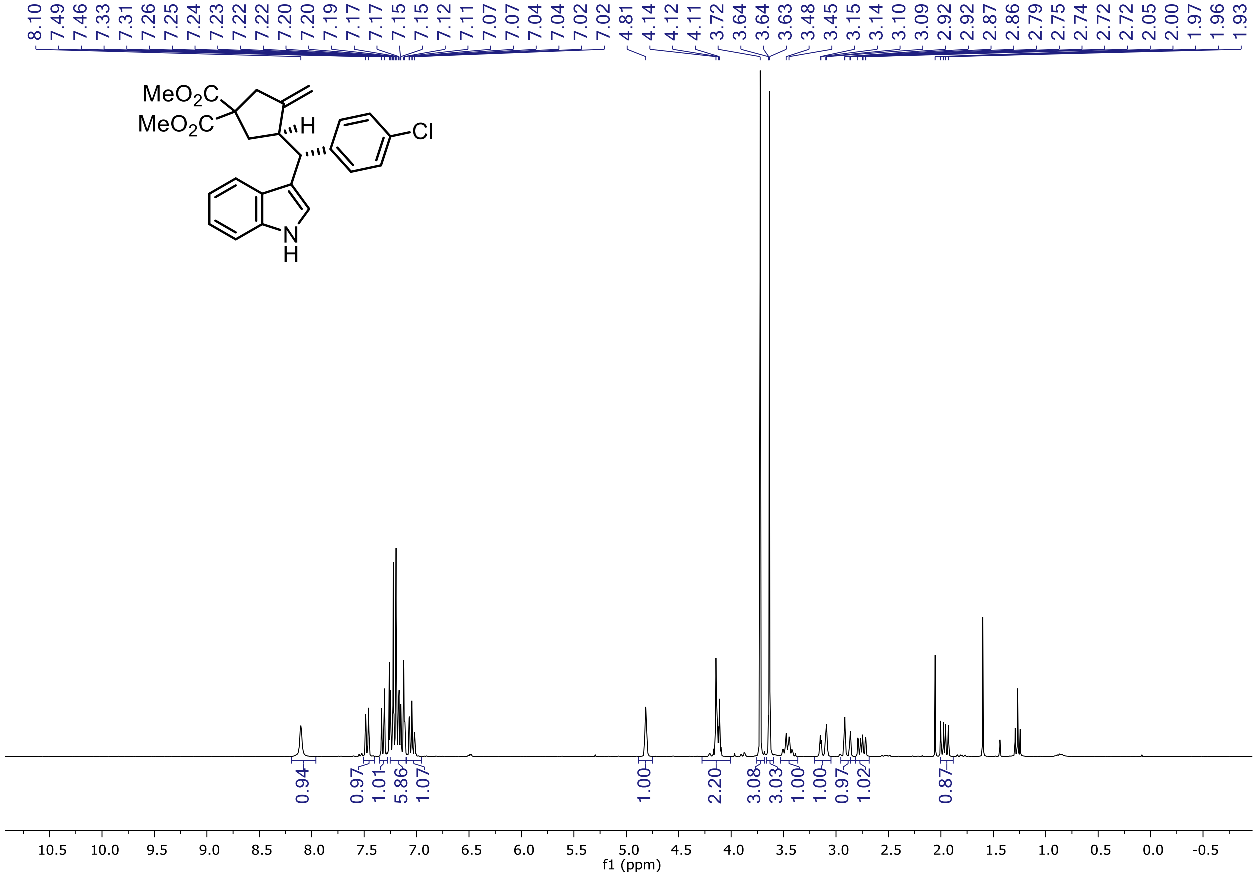

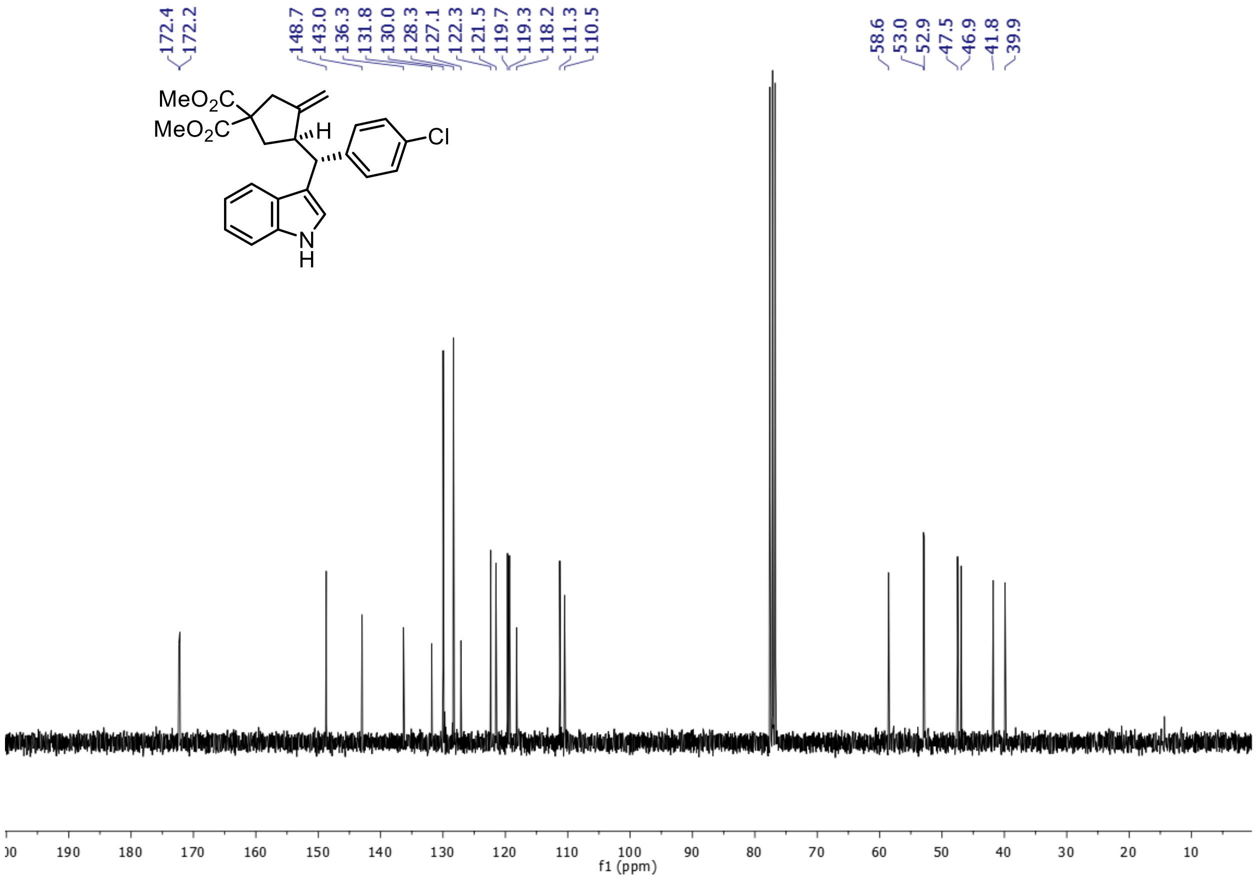

Compound **2w** ^{1}H and $^{13}C\{^{1}H\}$ NMR spectra in $CDCl_3$

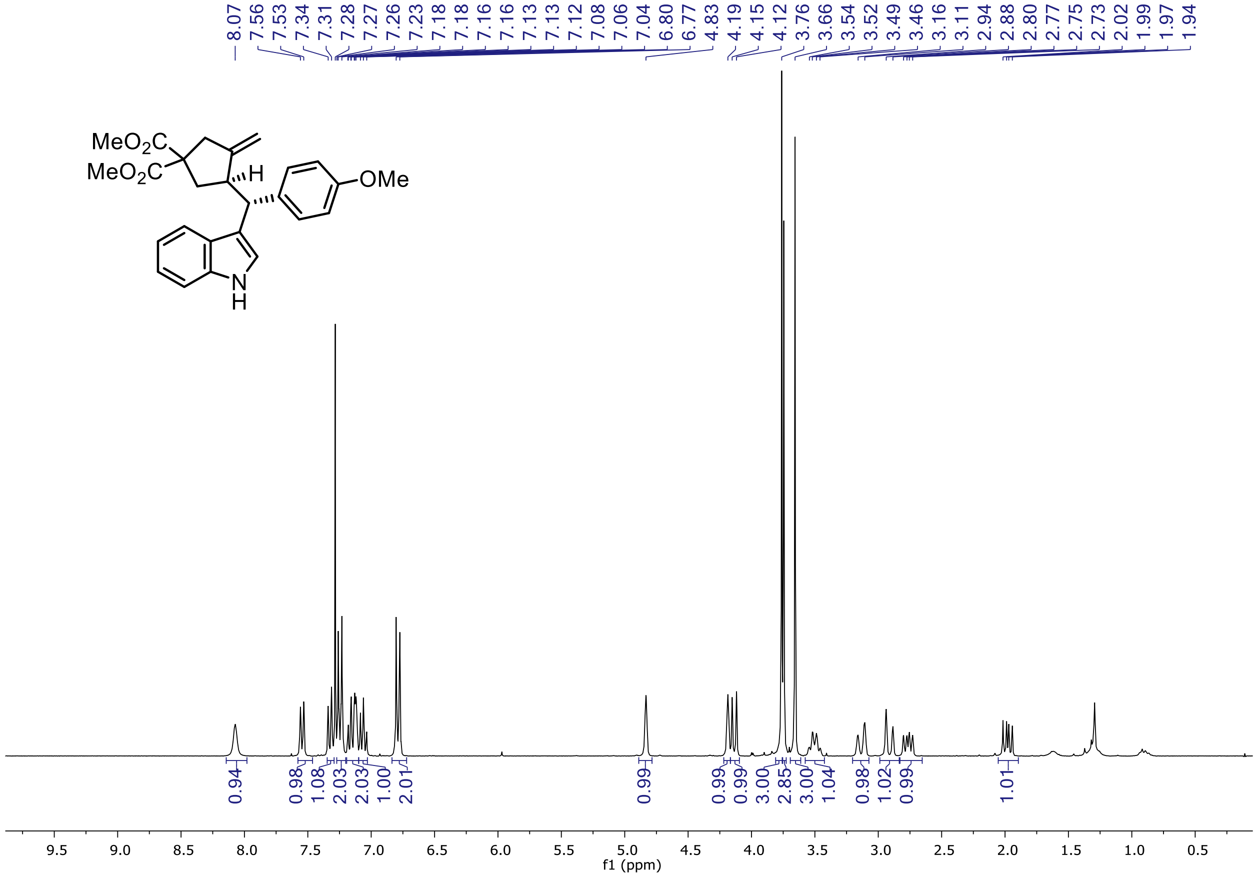

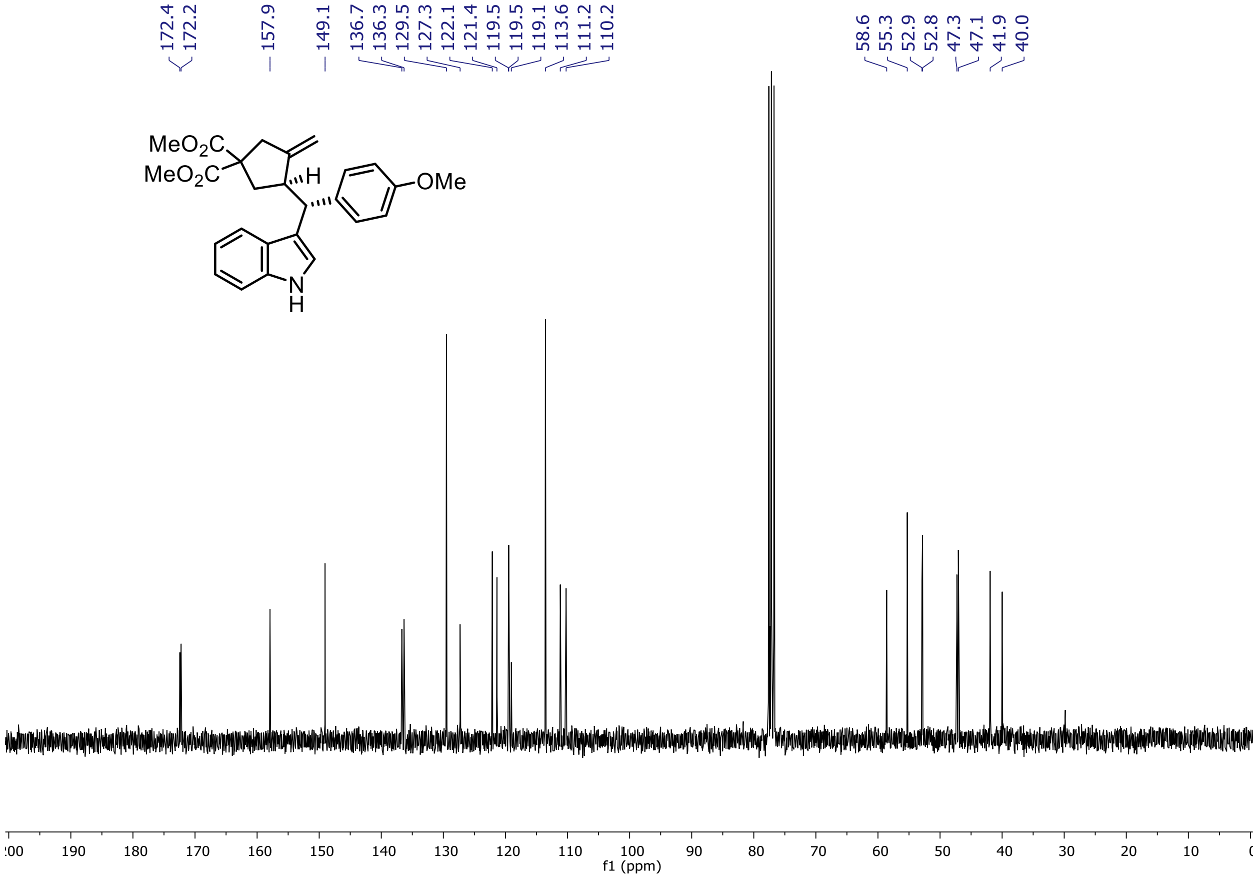

Compound **2x** ^{1}H and ^{13}C{^{1}H} NMR spectra in $CDCl_3$

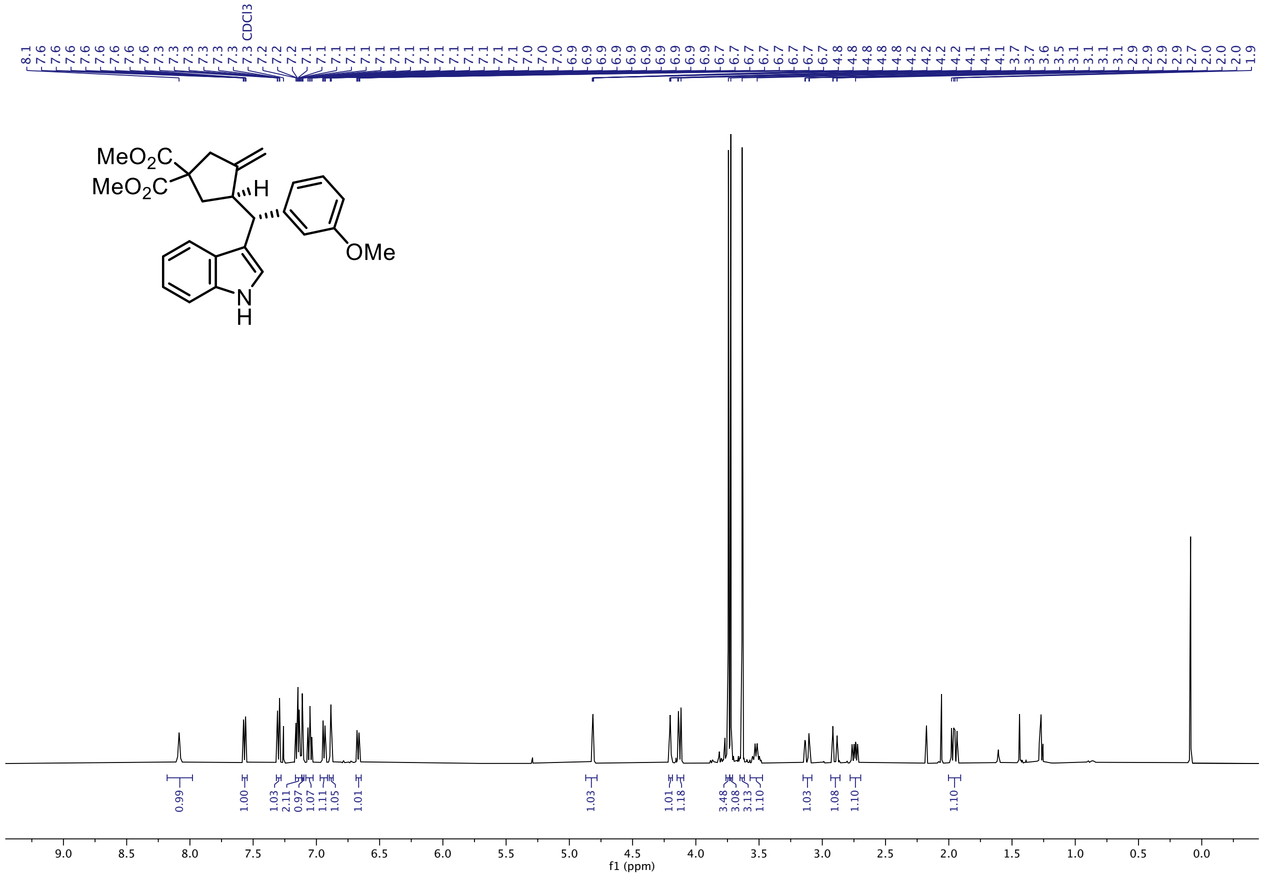

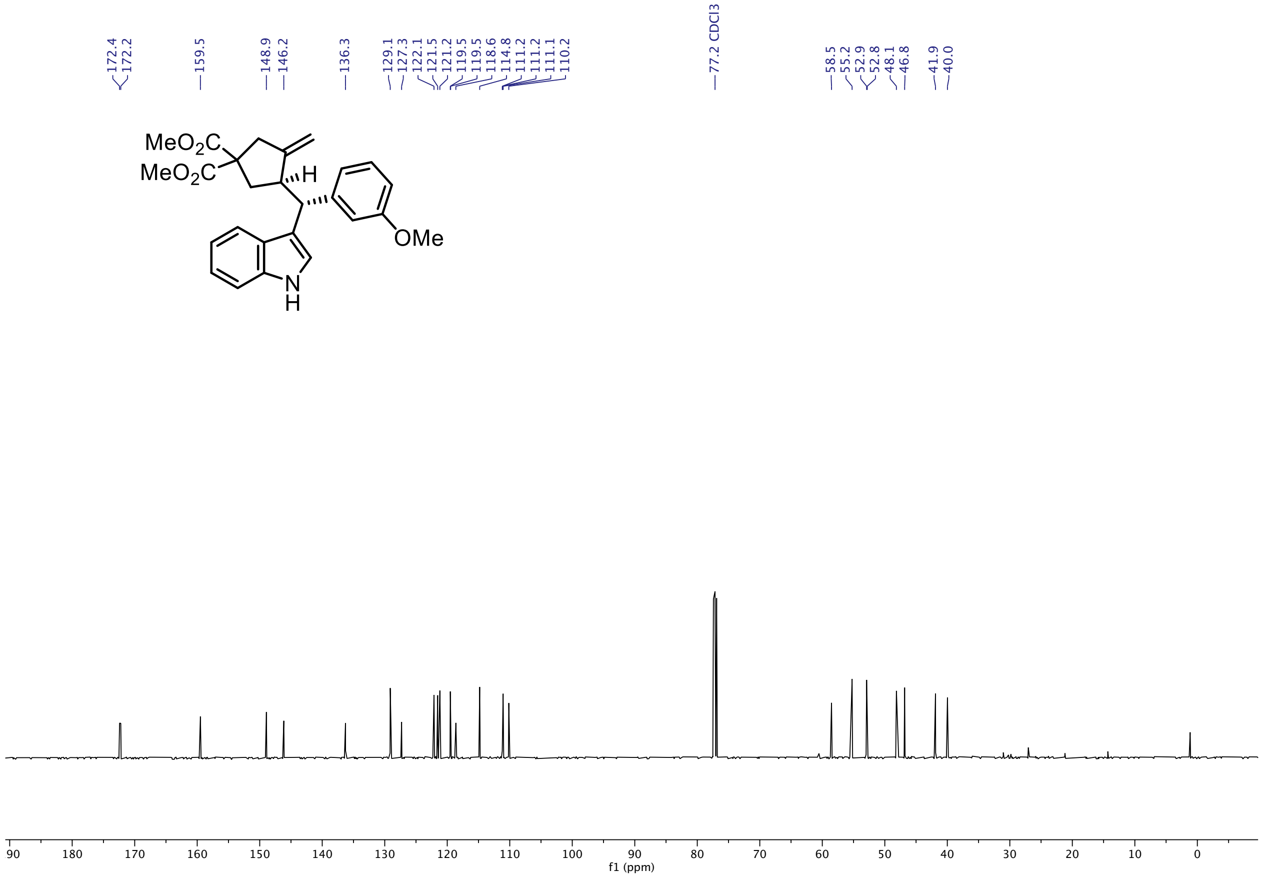

Compound **2y** ^{1}H and ^{13}C{^{1}H} NMR spectra in $CDCl_3$

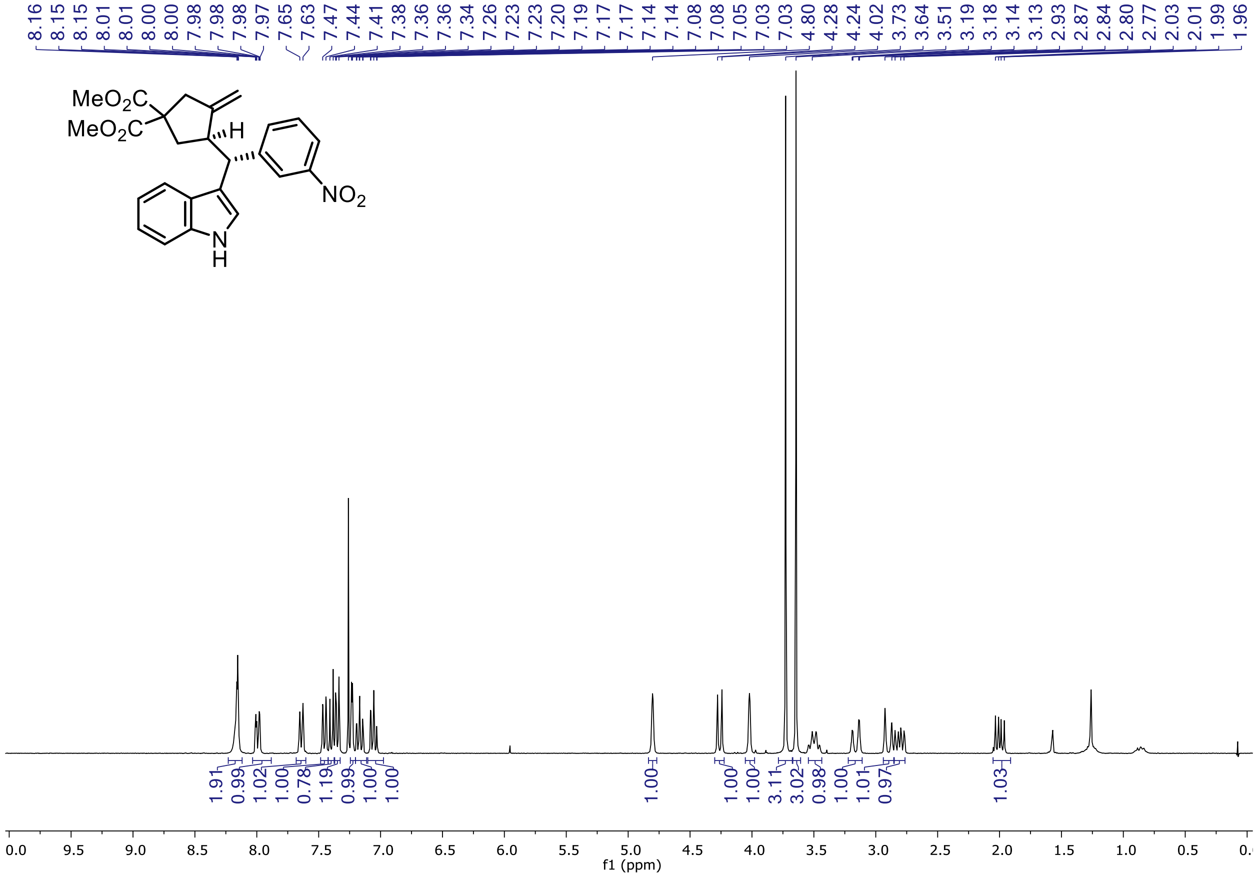

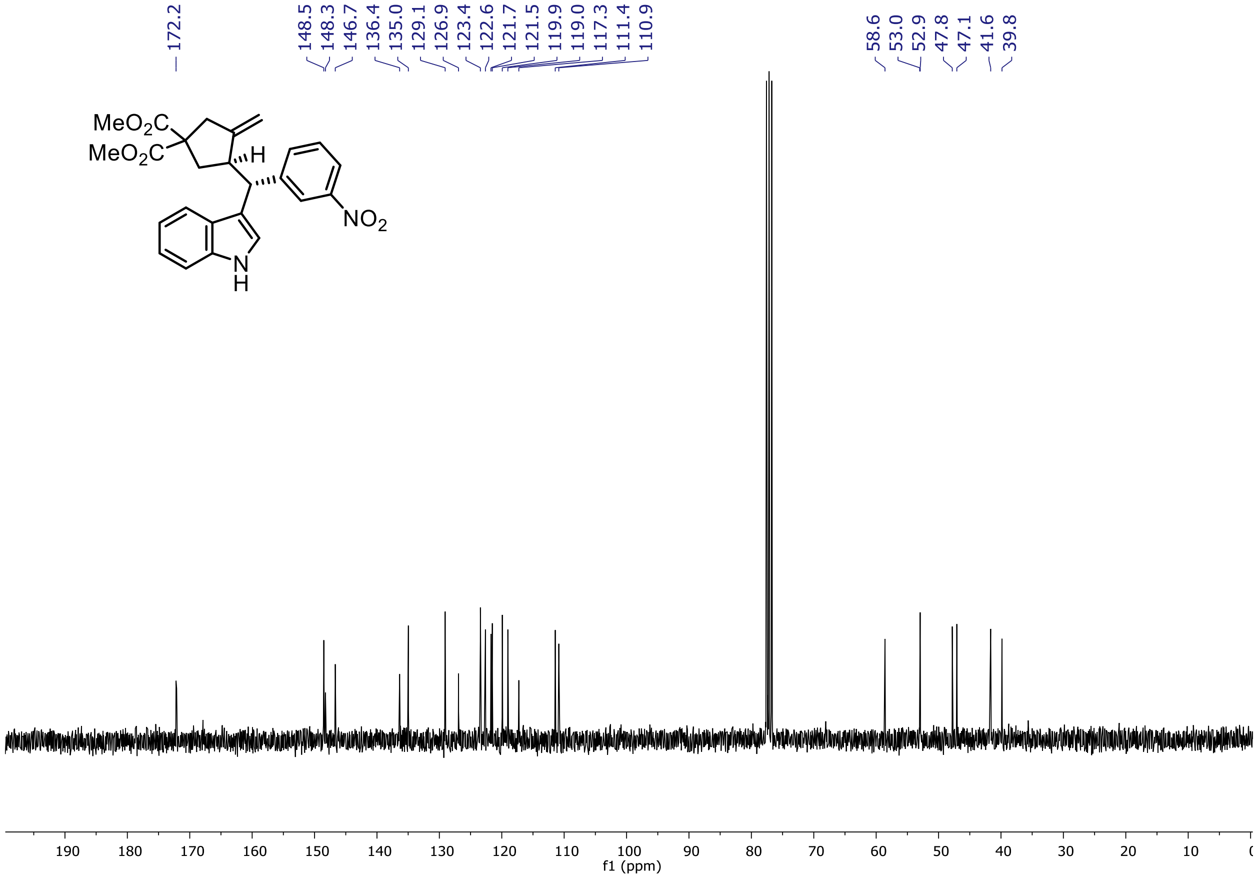

Compound **2z** ^{1}H and ^{13}C{^{1}H} NMR spectra in $CDCl_3$

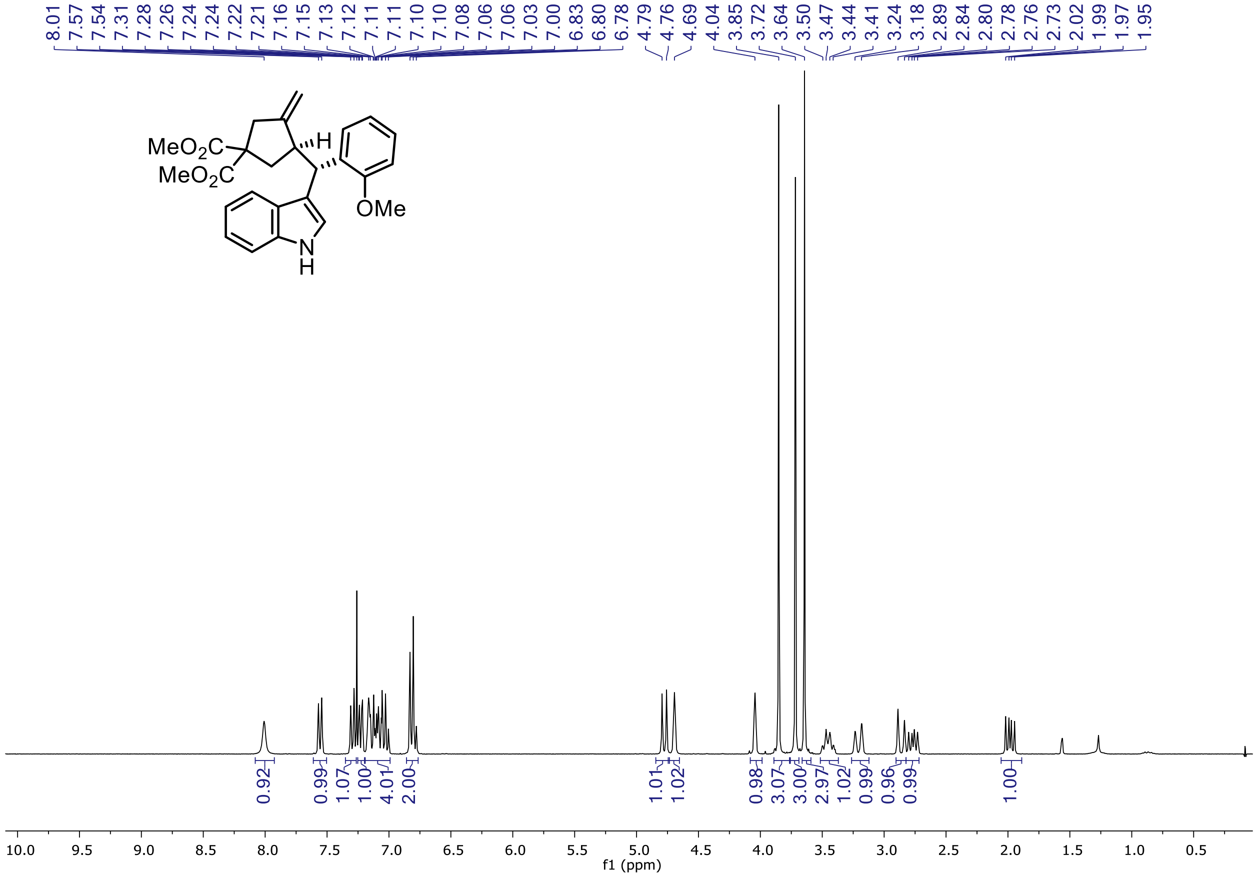

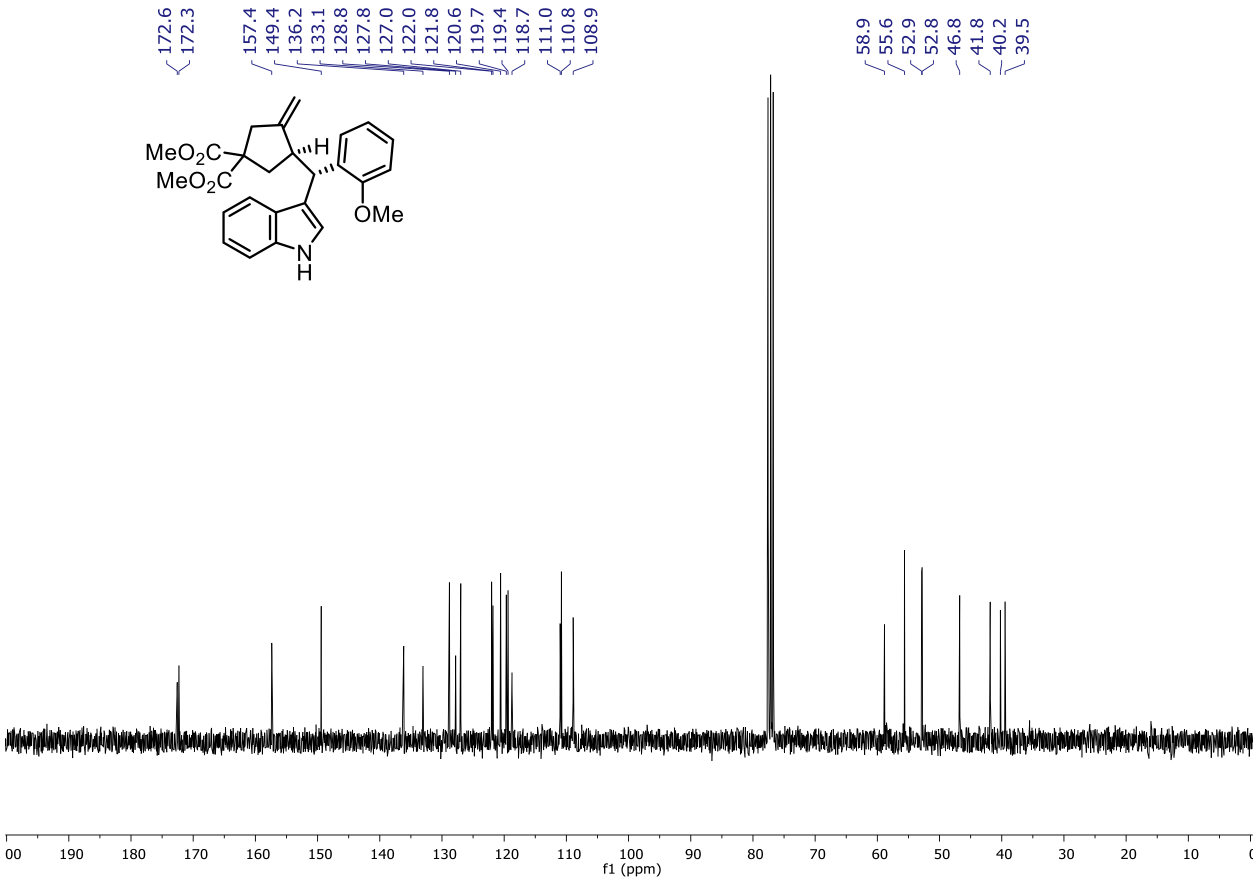

Compound **2aa** ^{1}H and ^{13}C{^{1}H} NMR spectra in $CDCl_3$

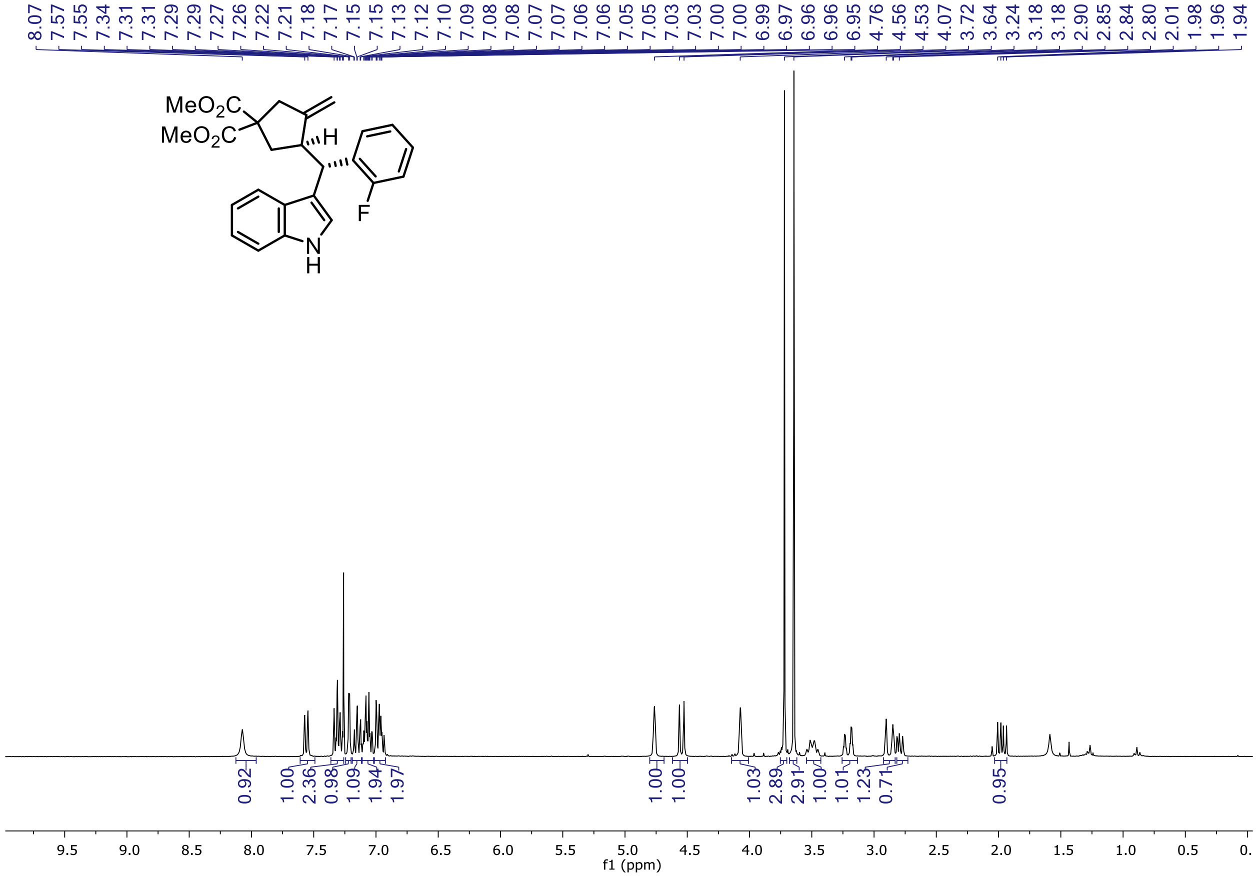

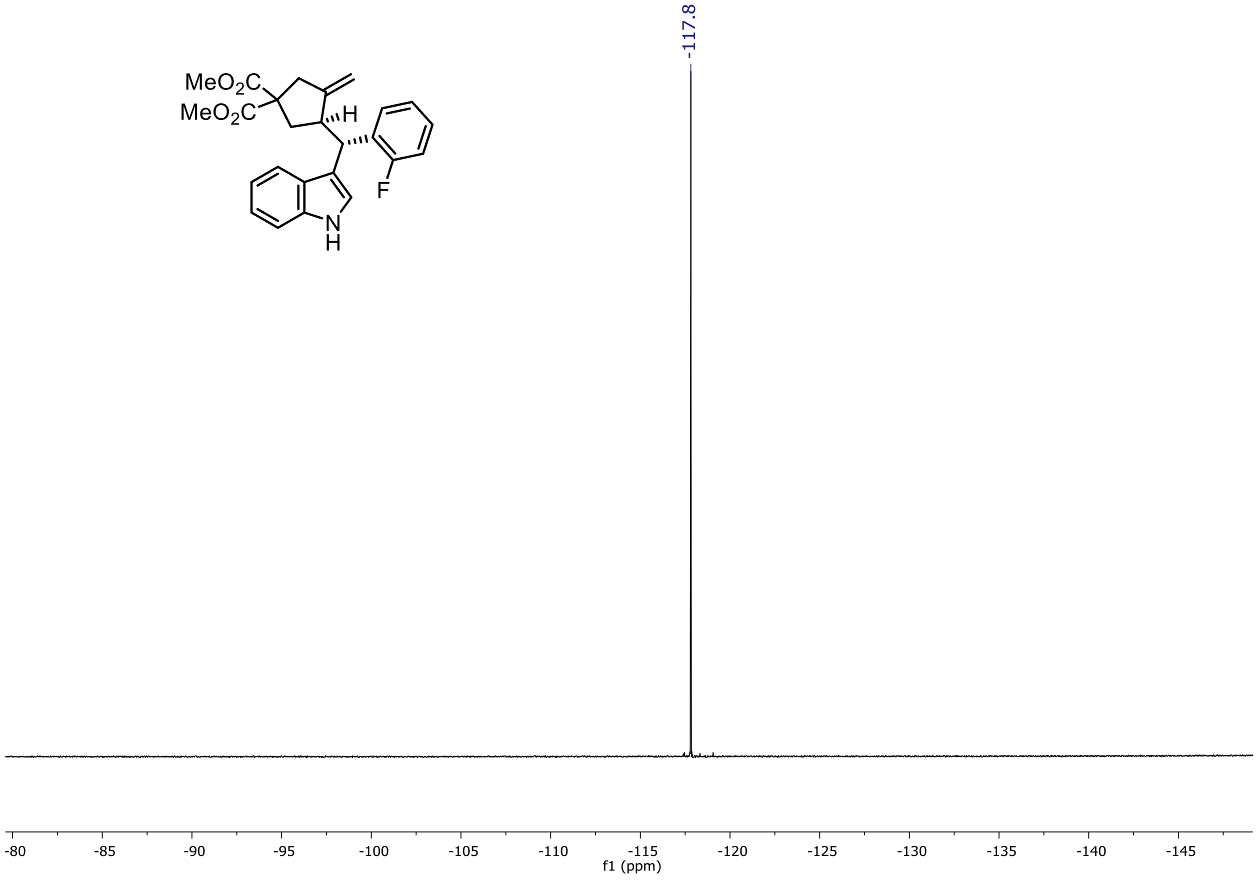

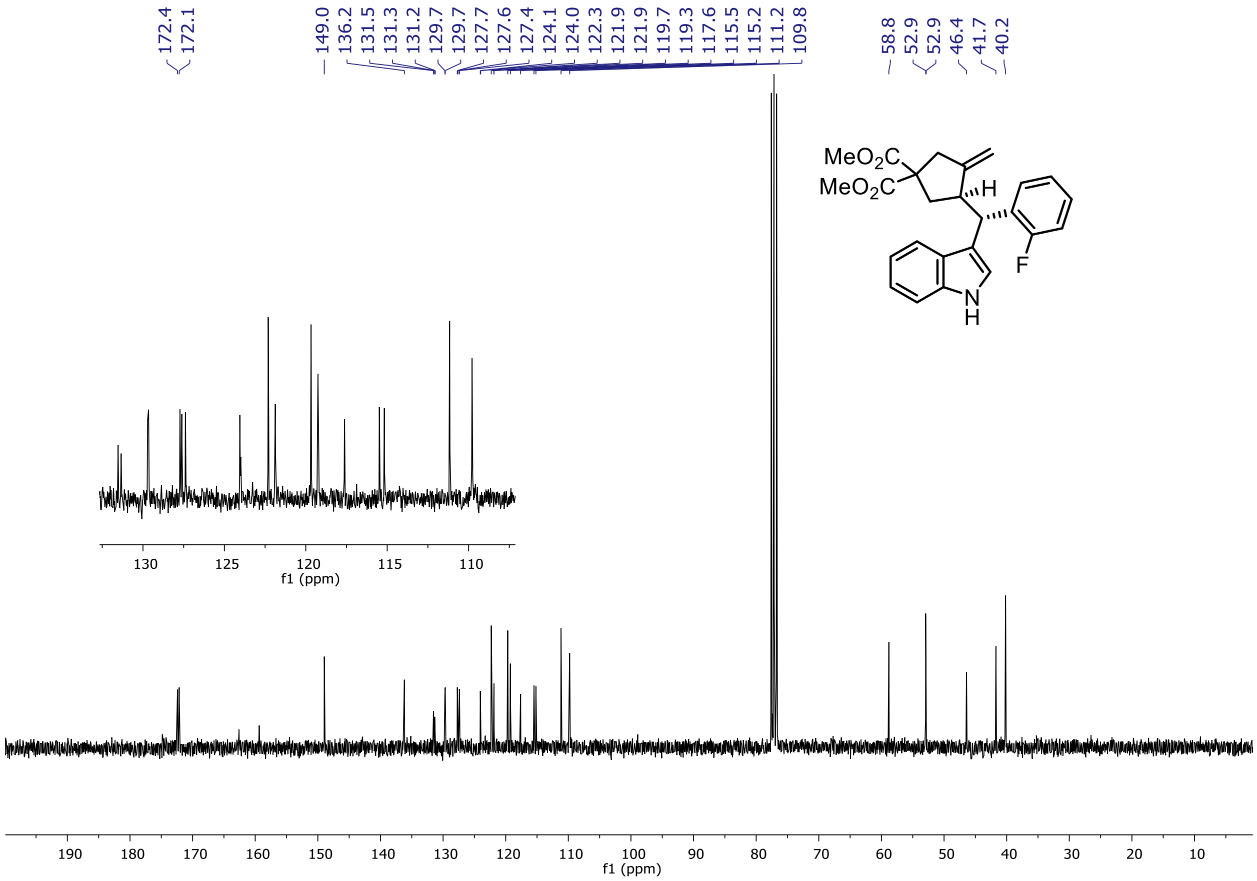
172.4
172.1
149.0
136.2
131.5
131.3
131.2
129.7
129.7
127.7
127.6
127.4
124.1
124.0
122.3
121.9
121.9
119.7
119.3
117.6
115.5
115.2
111.2
109.8
58.8
52.9
52.9
46.4
41.7
40.2
MeO2C
MeO2C
H
F
N
H
f1 (ppm)
f1 (ppm)

Compound **2ab** ^{1}H and $^{13}C\{^{1}H\}$ NMR spectra in $CDCl_3$

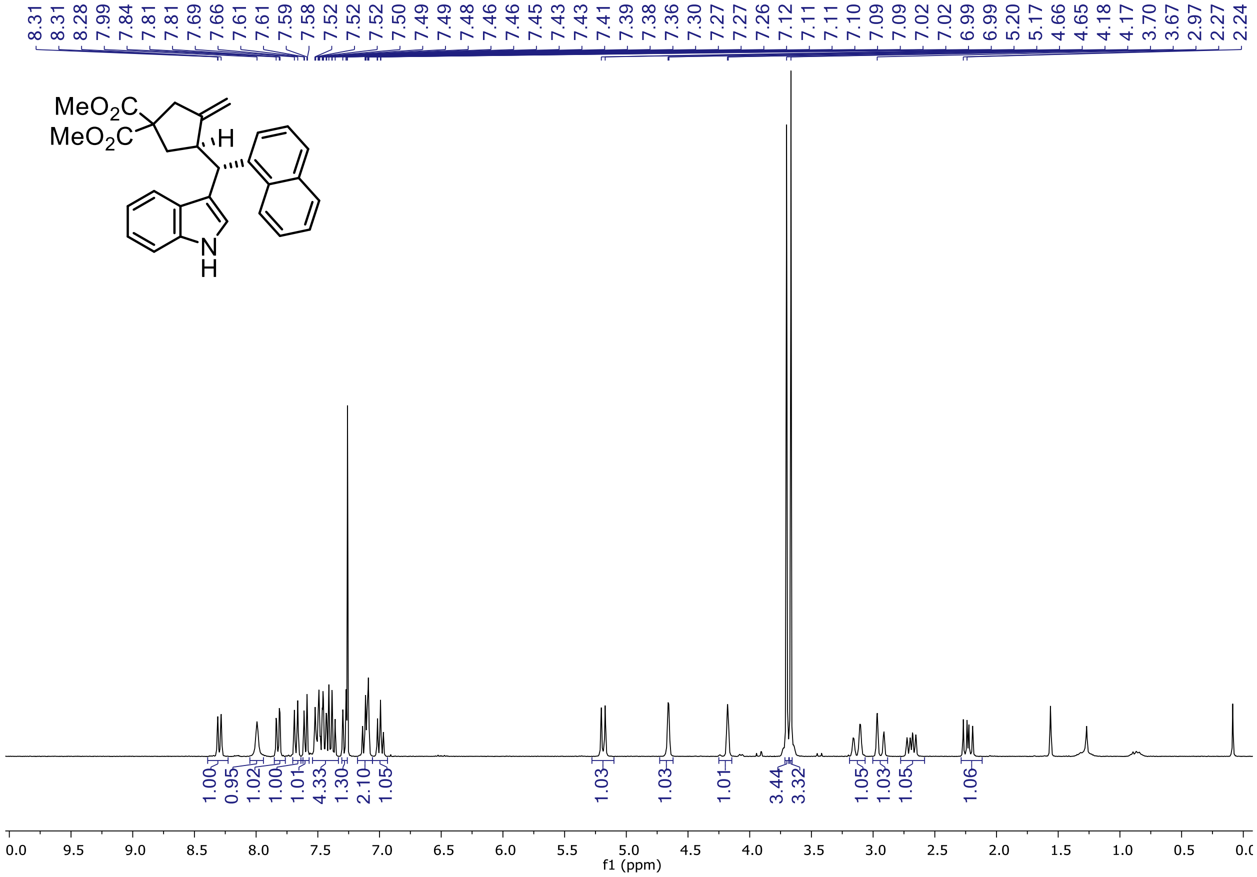

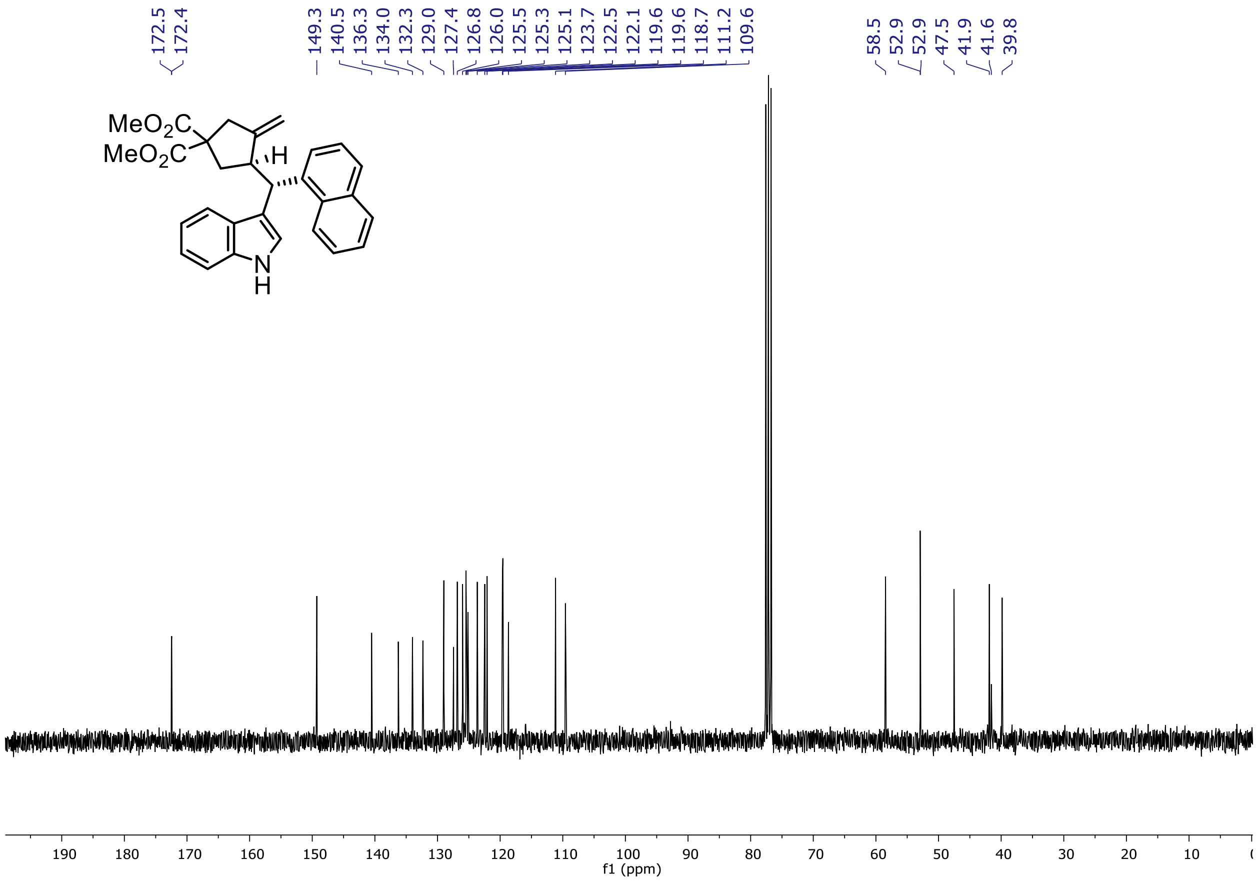

Compound **2ac** ^{1}H and $^{13}C\{^{1}H\}$ NMR spectra in $CDCl_3$

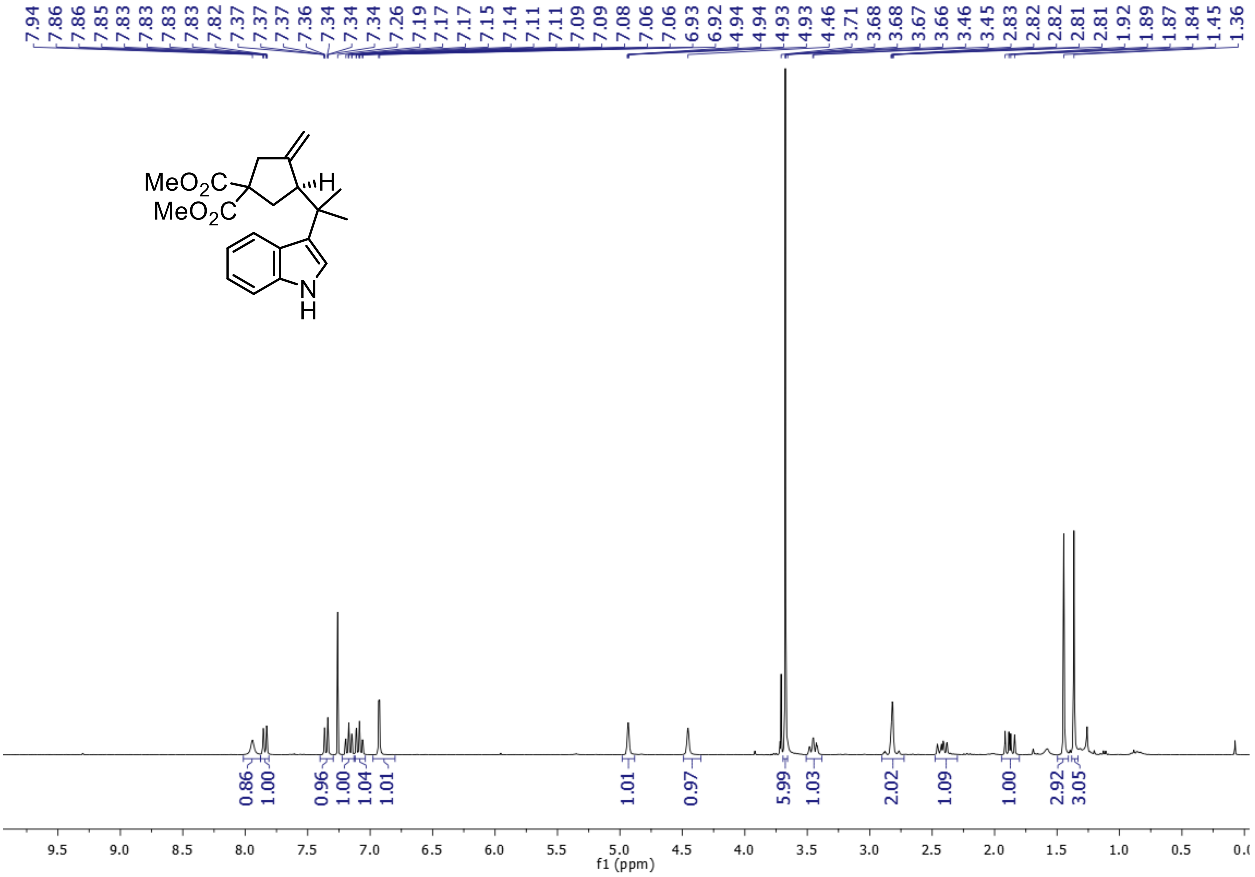

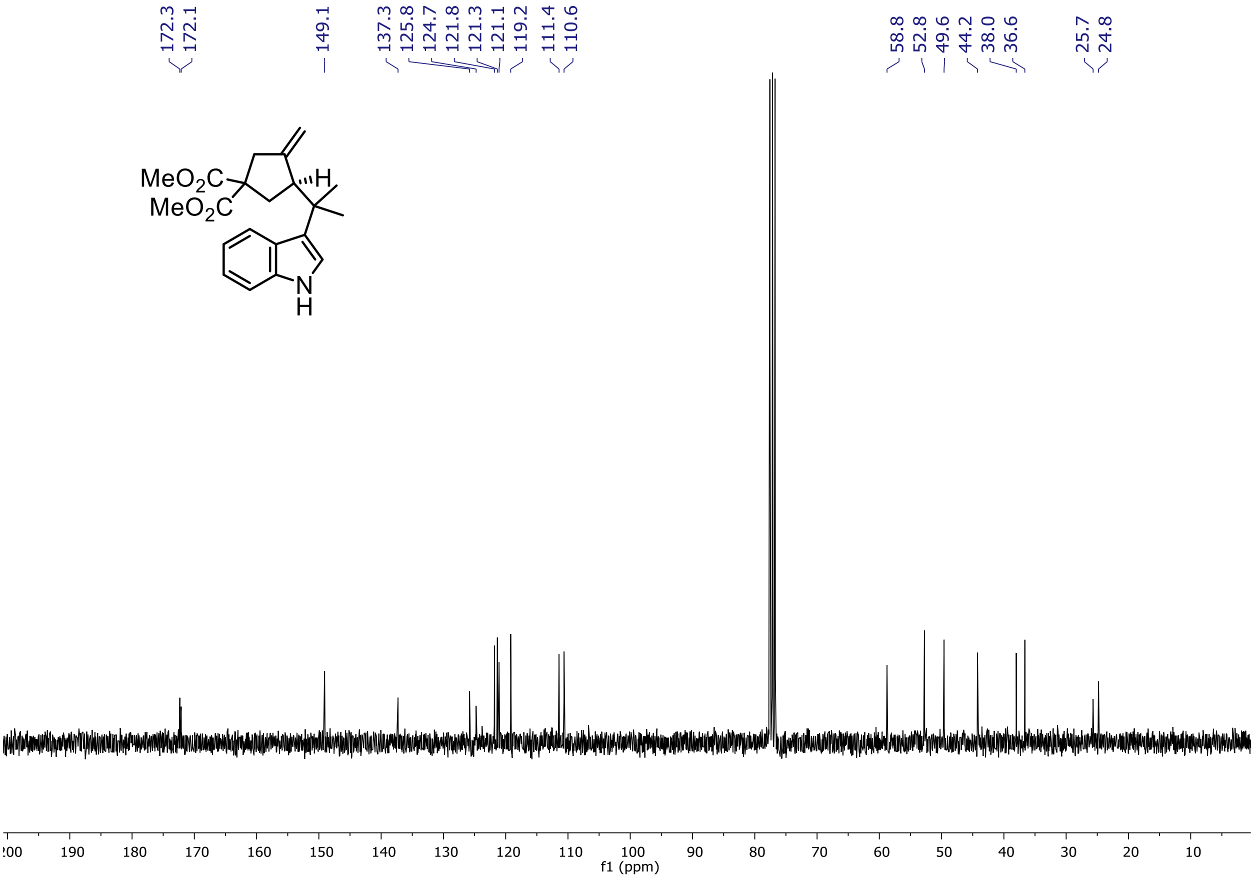

Compound **2ad** ^{1}H and ^{13}C{^{1}H} NMR spectra in $CDCl_3$

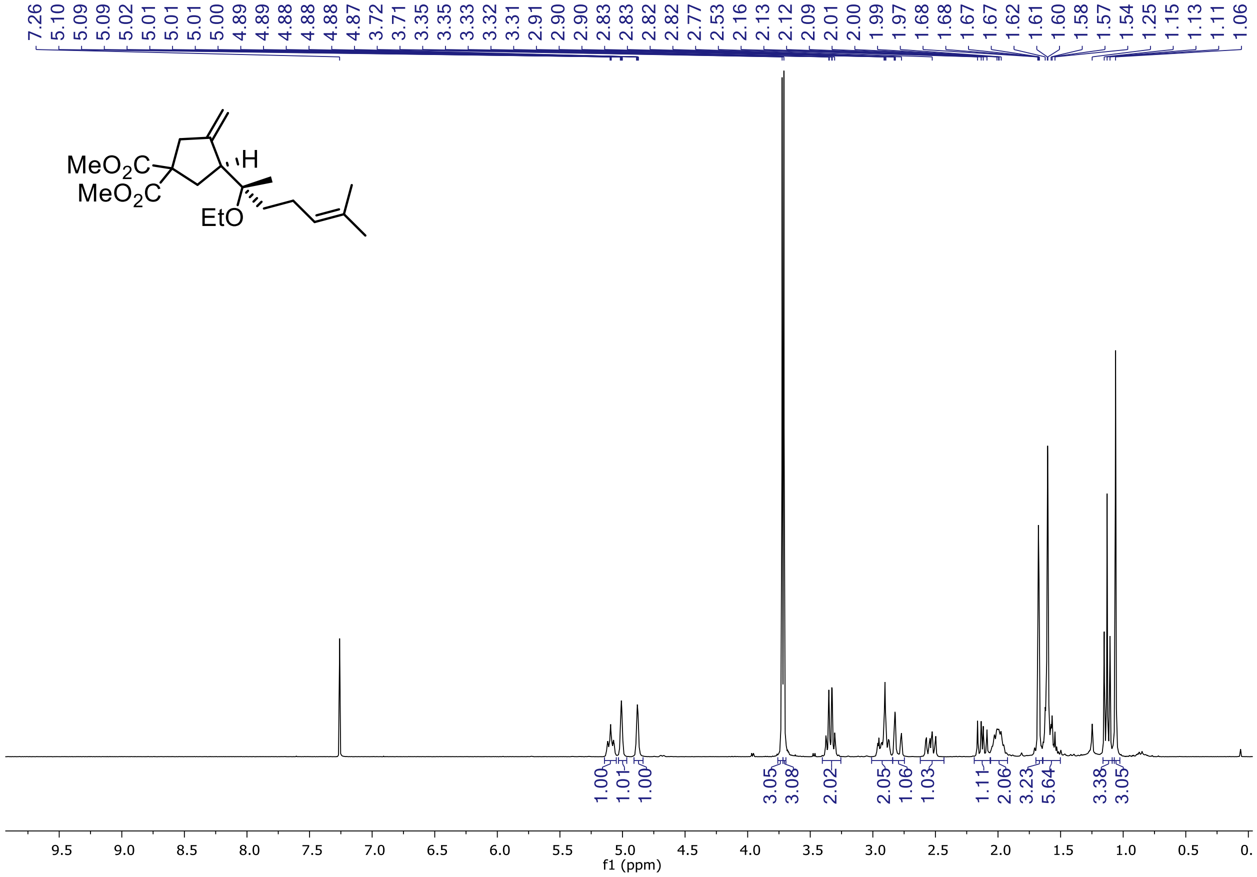

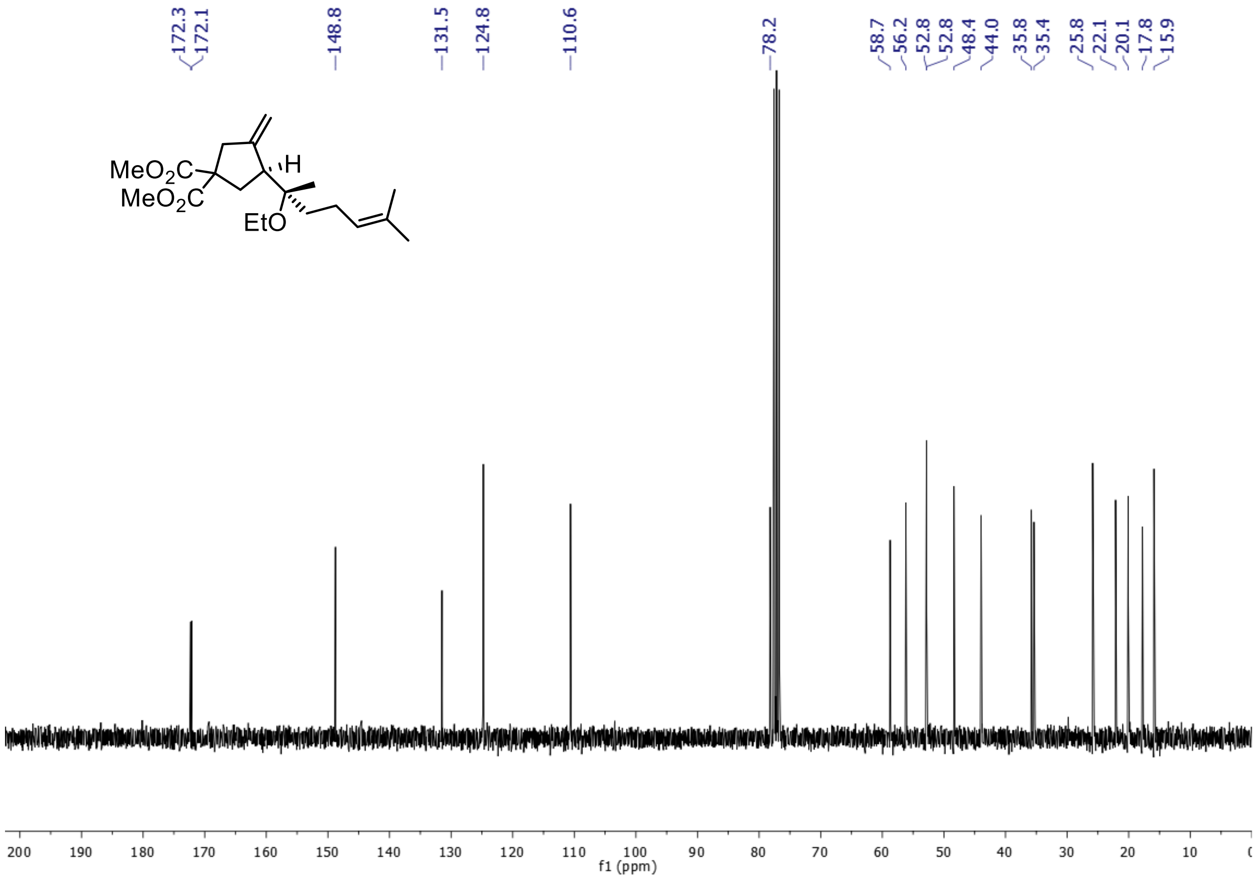

Compound **2ae** ^{1}H and ^{13}C{^{1}H} NMR spectra in $CDCl_3$

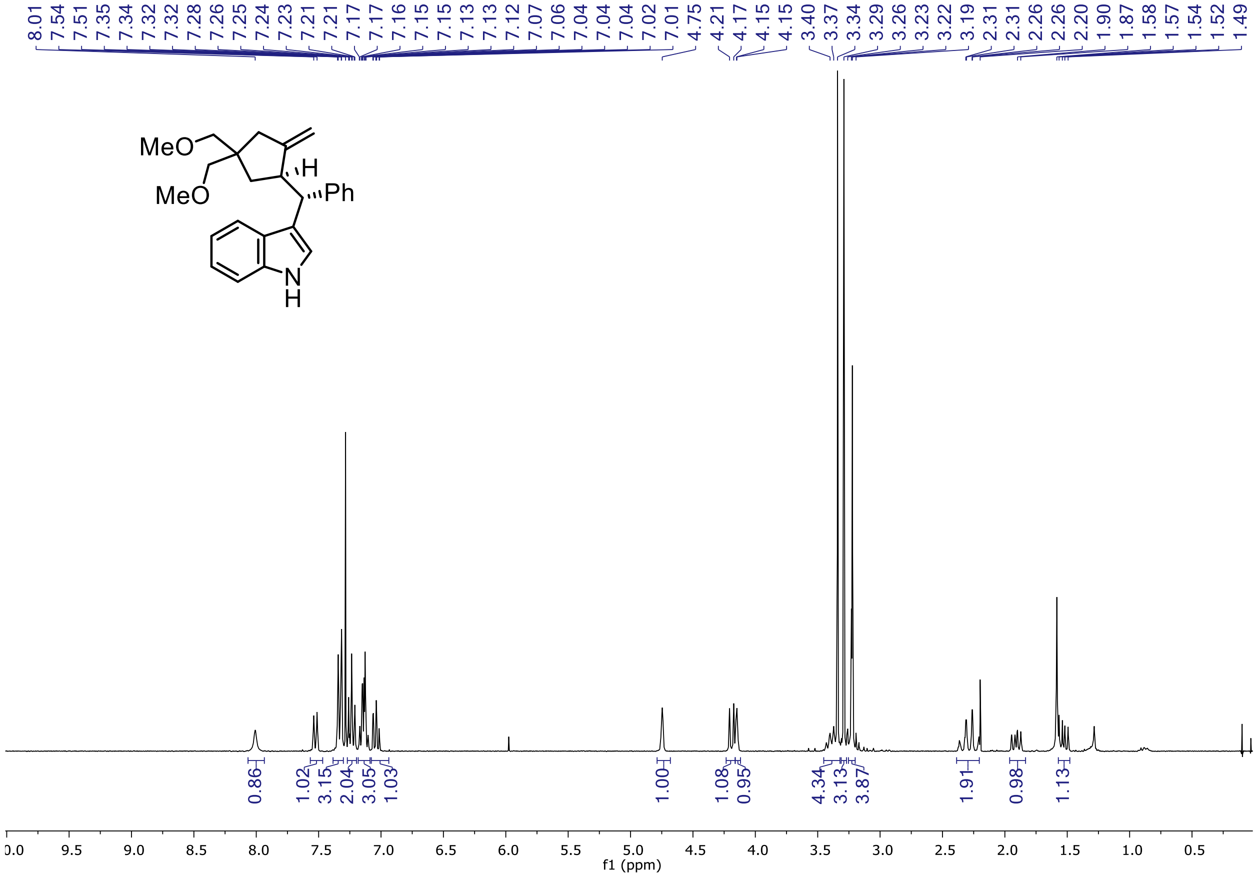

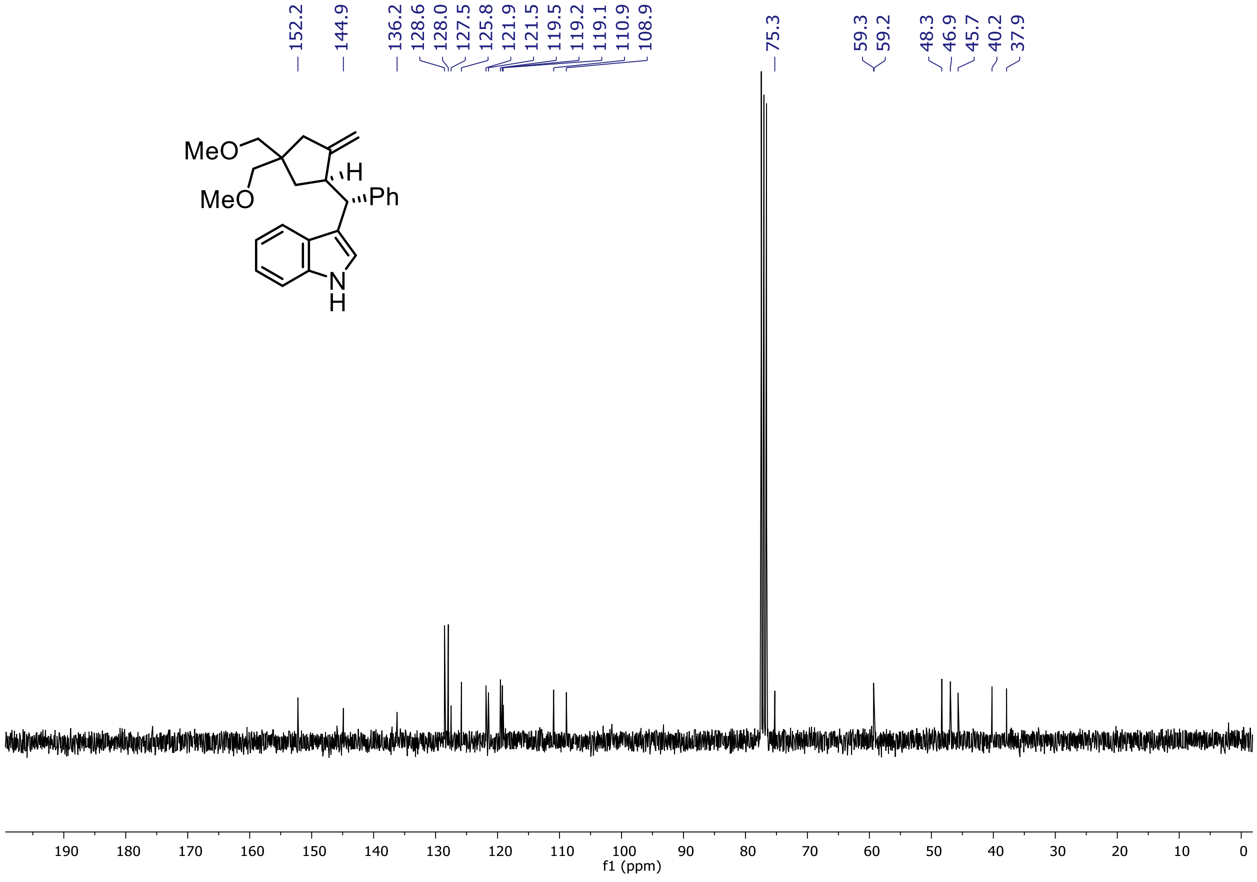

Compound **2af** ^{1}H and ^{13}C{^{1}H} NMR spectra in $CDCl_3$

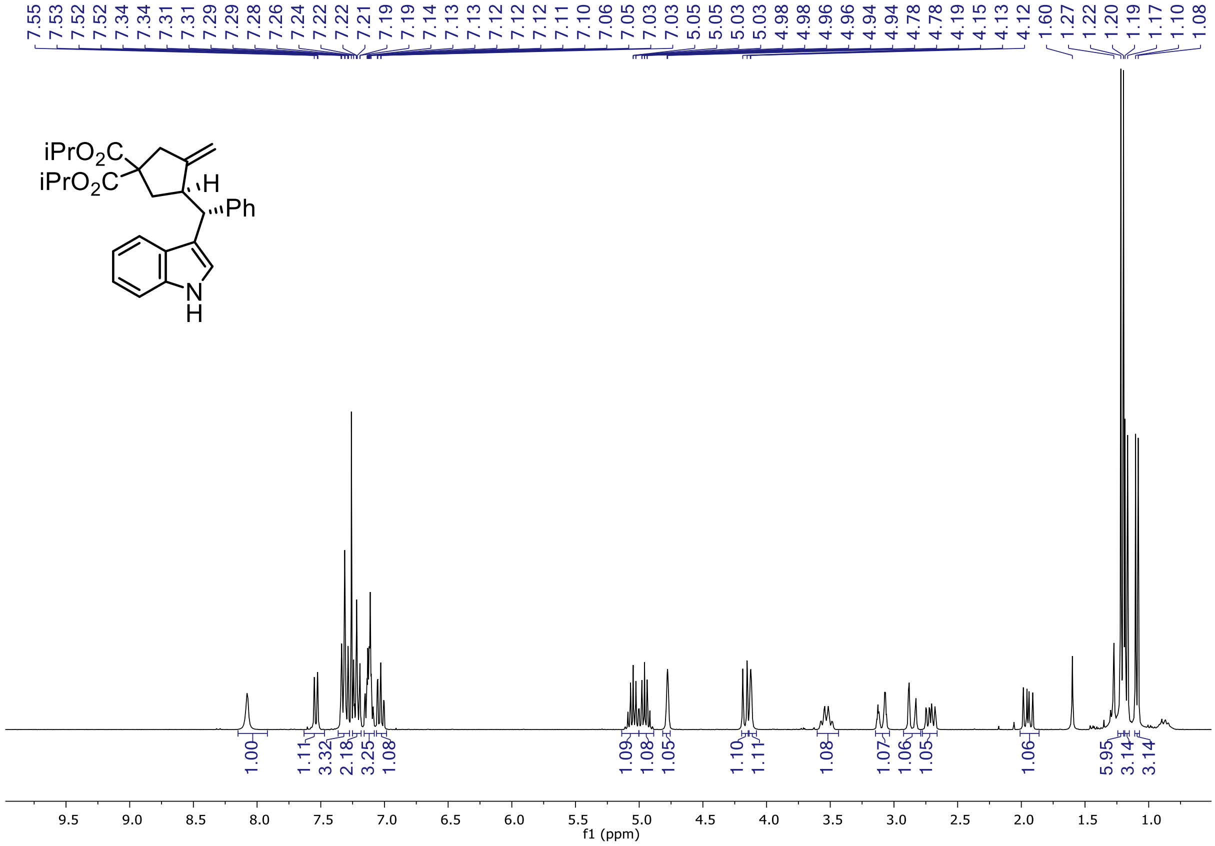

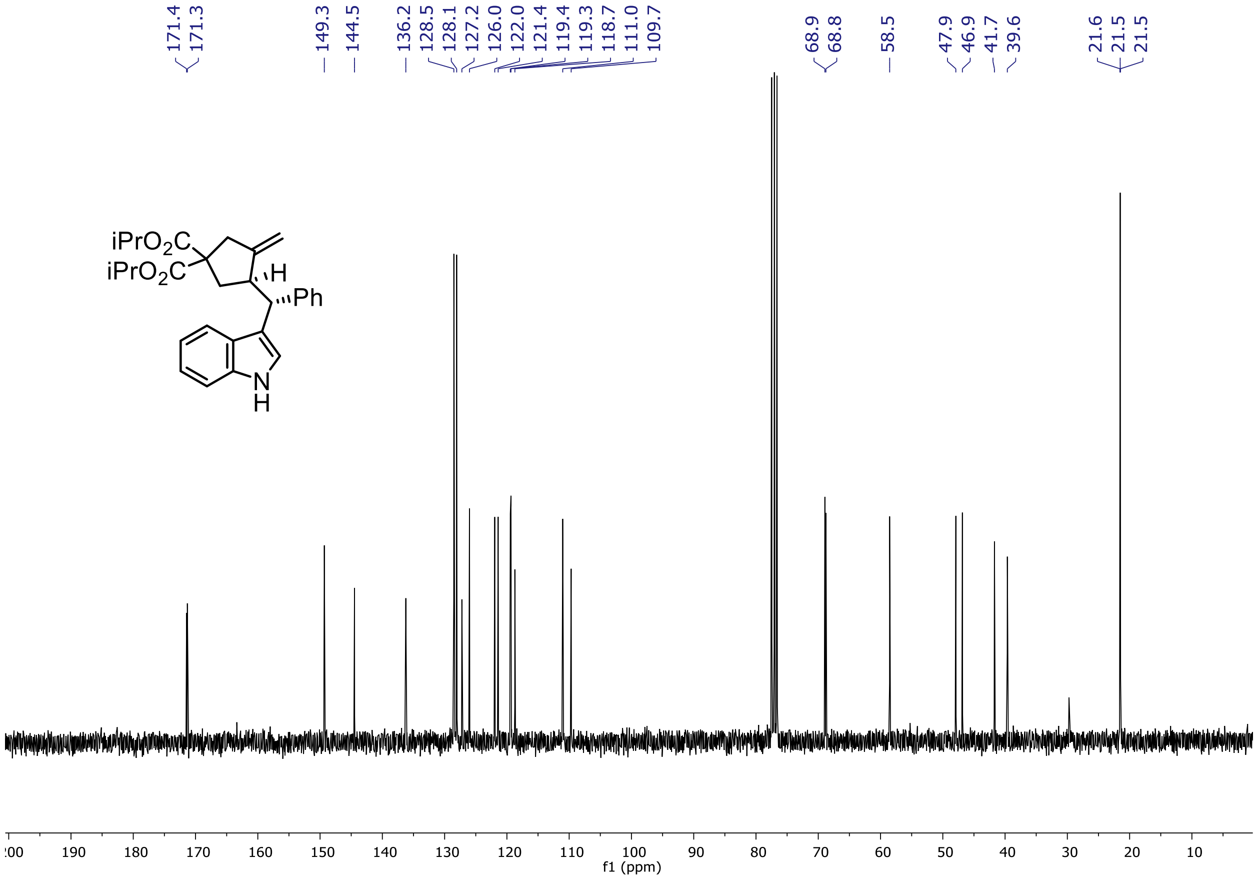

Compound **2ag** ^{1}H and $^{13}C\{^{1}H\}$ NMR spectra in $CDCl_3$

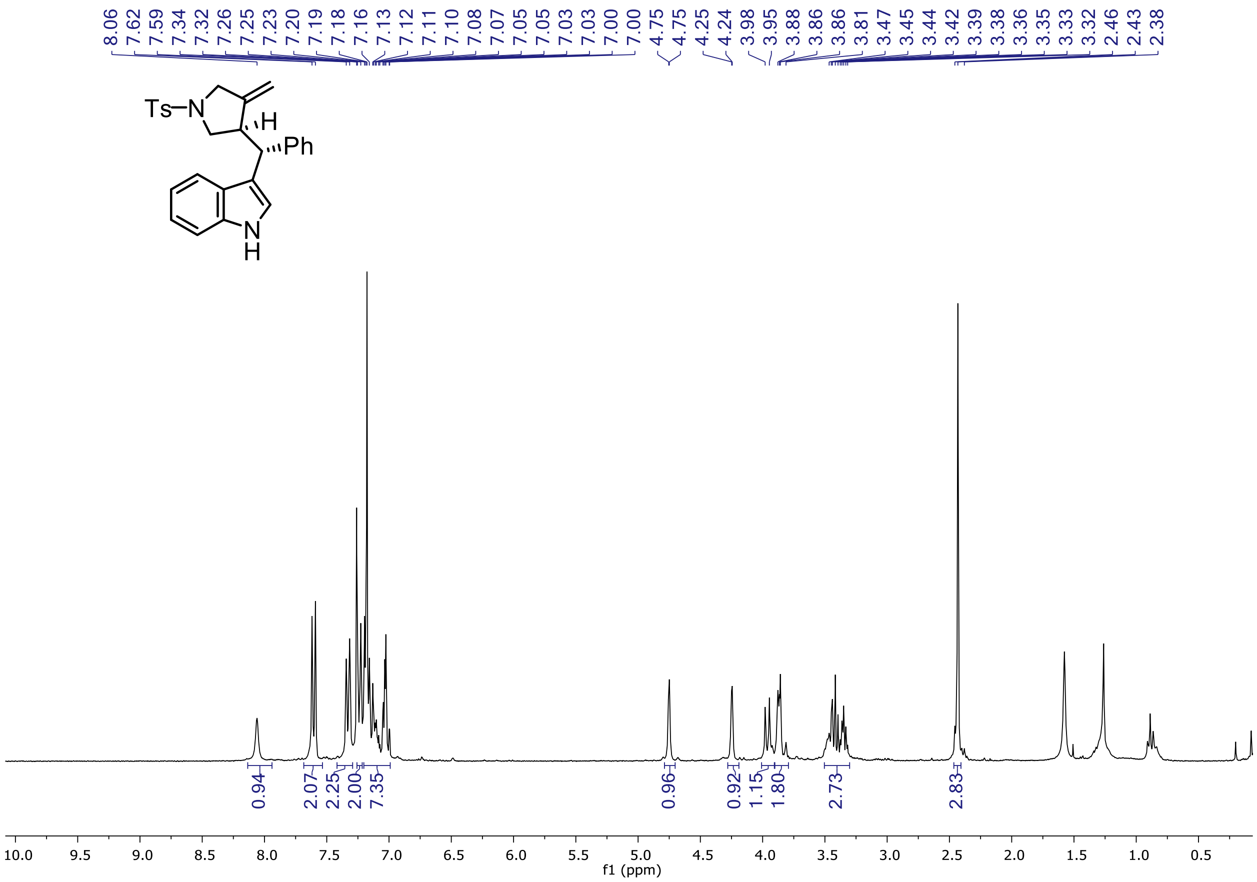

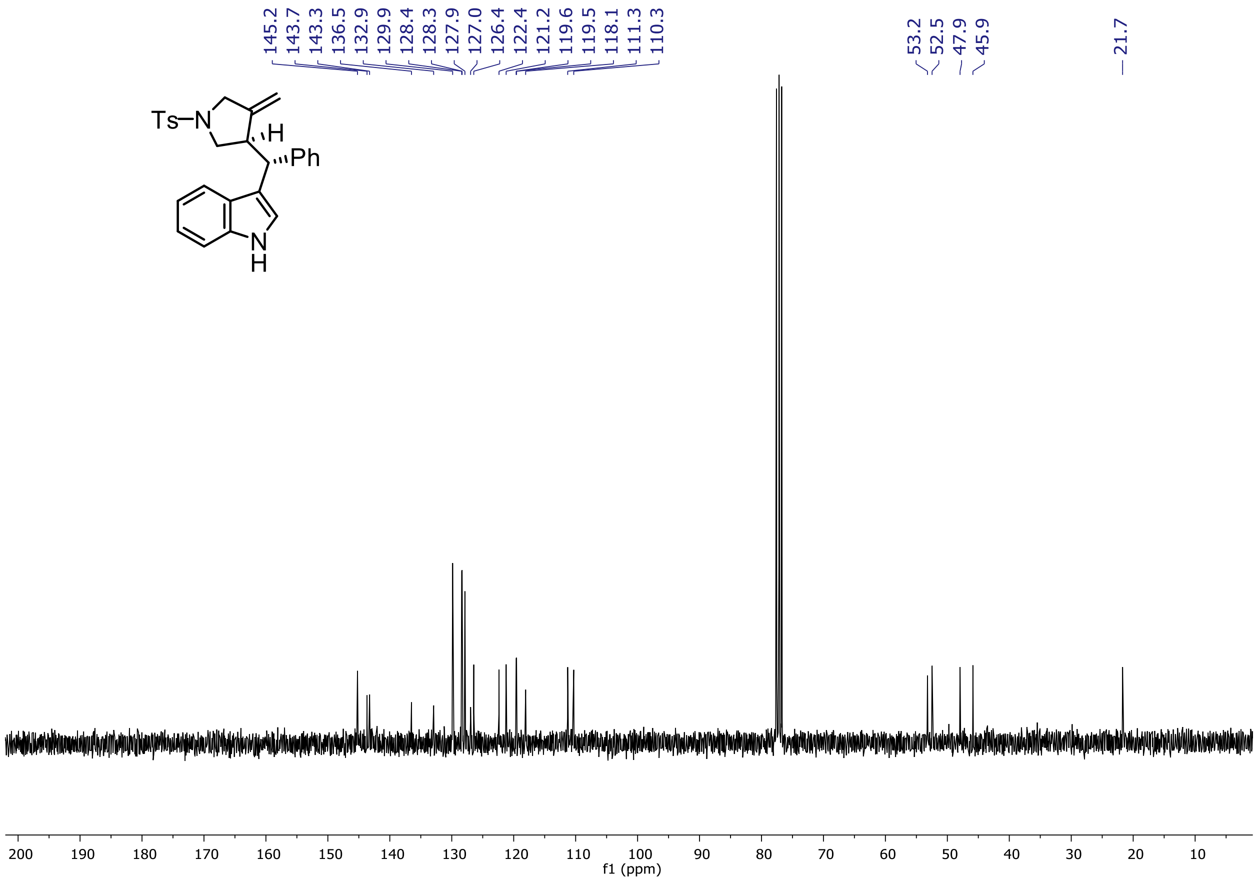

Compound **2ah** ^{1}H and $^{13}C\{^{1}H\}$ NMR spectra in $CDCl_3$

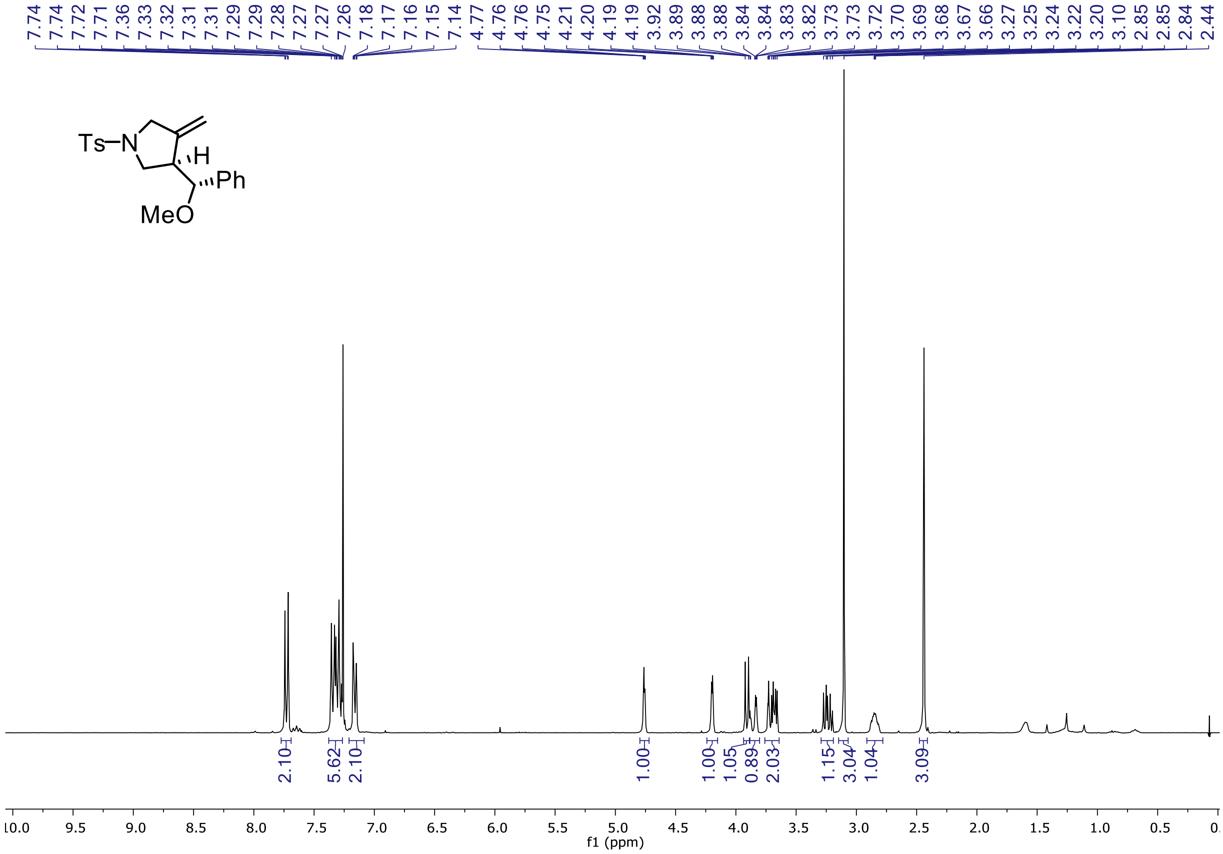

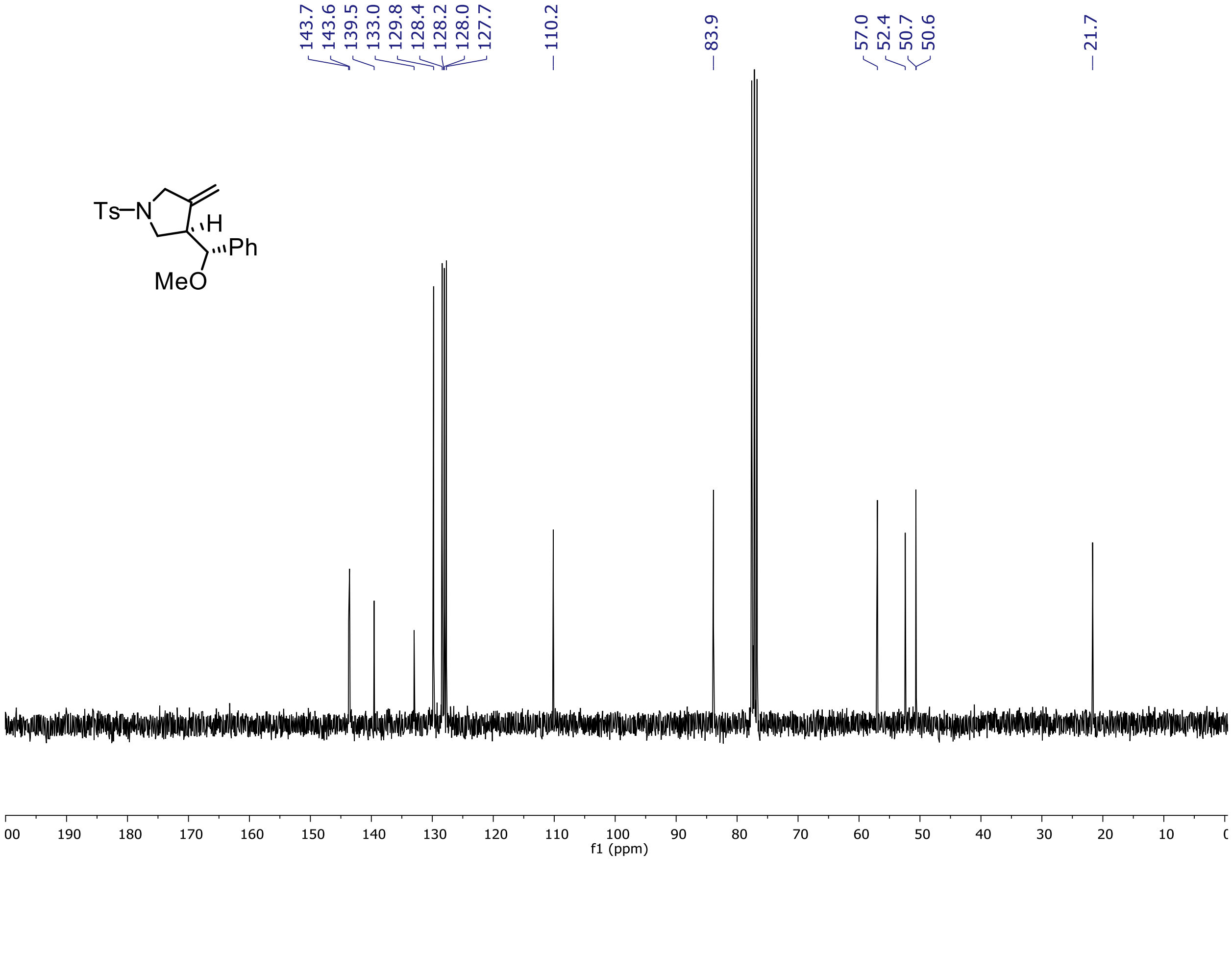

Compound **2ai** ^{1}H and $^{13}C\{^{1}H\}$ NMR spectra in $CDCl_3$

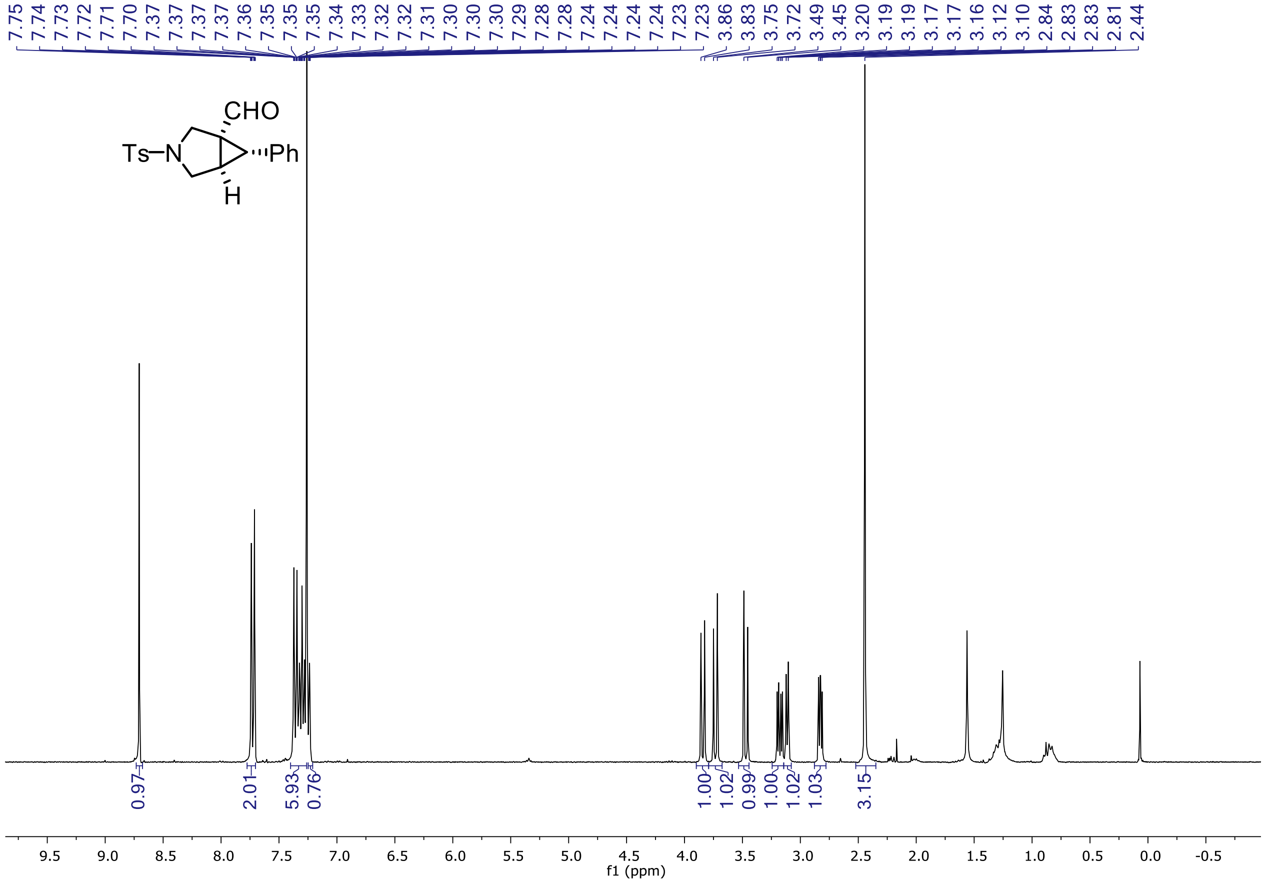

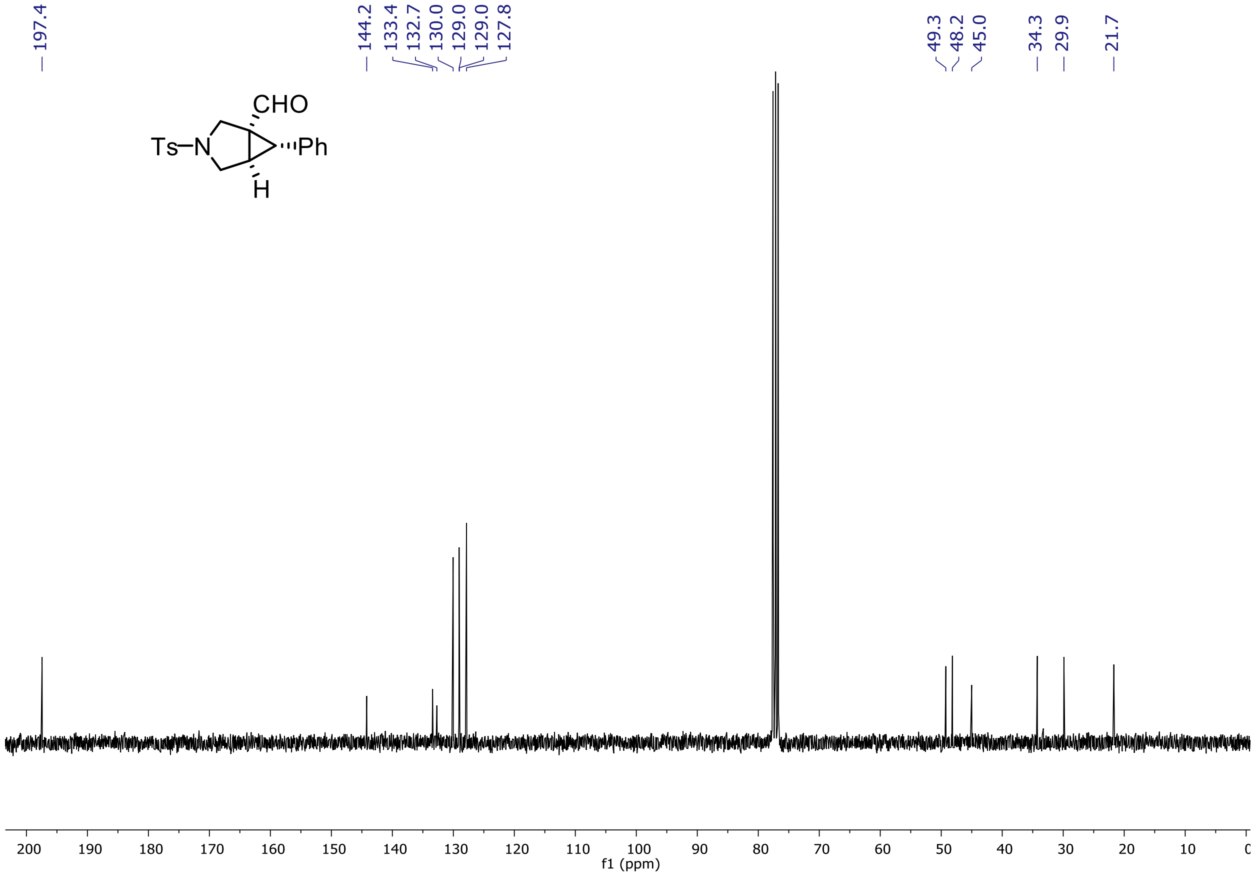

Compound **3a** ^{1}H and ^{13}C{^{1}H} NMR spectra in $CDCl_3$

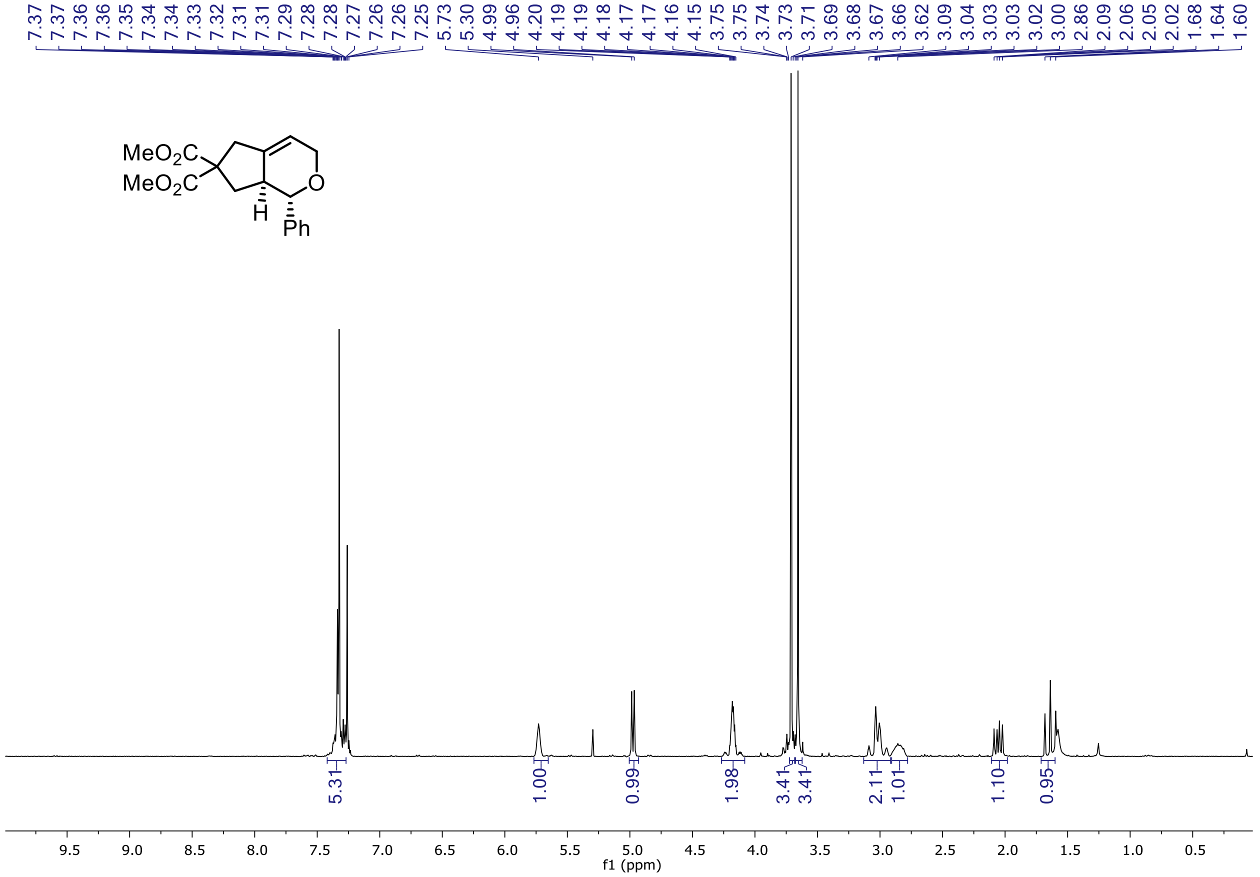

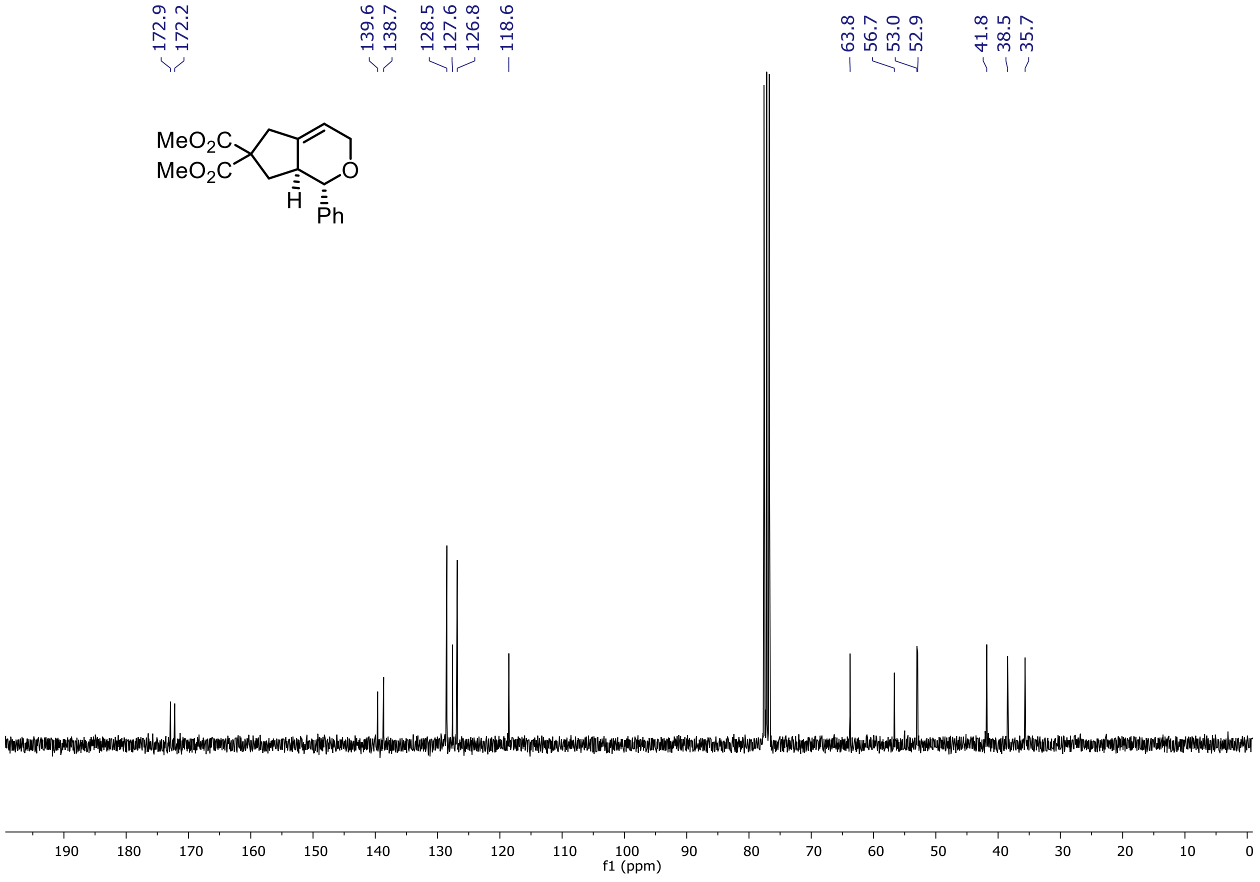

Compound **3b** ^{1}H and $^{13}C\{^{1}H\}$ NMR spectra in $CDCl_3$

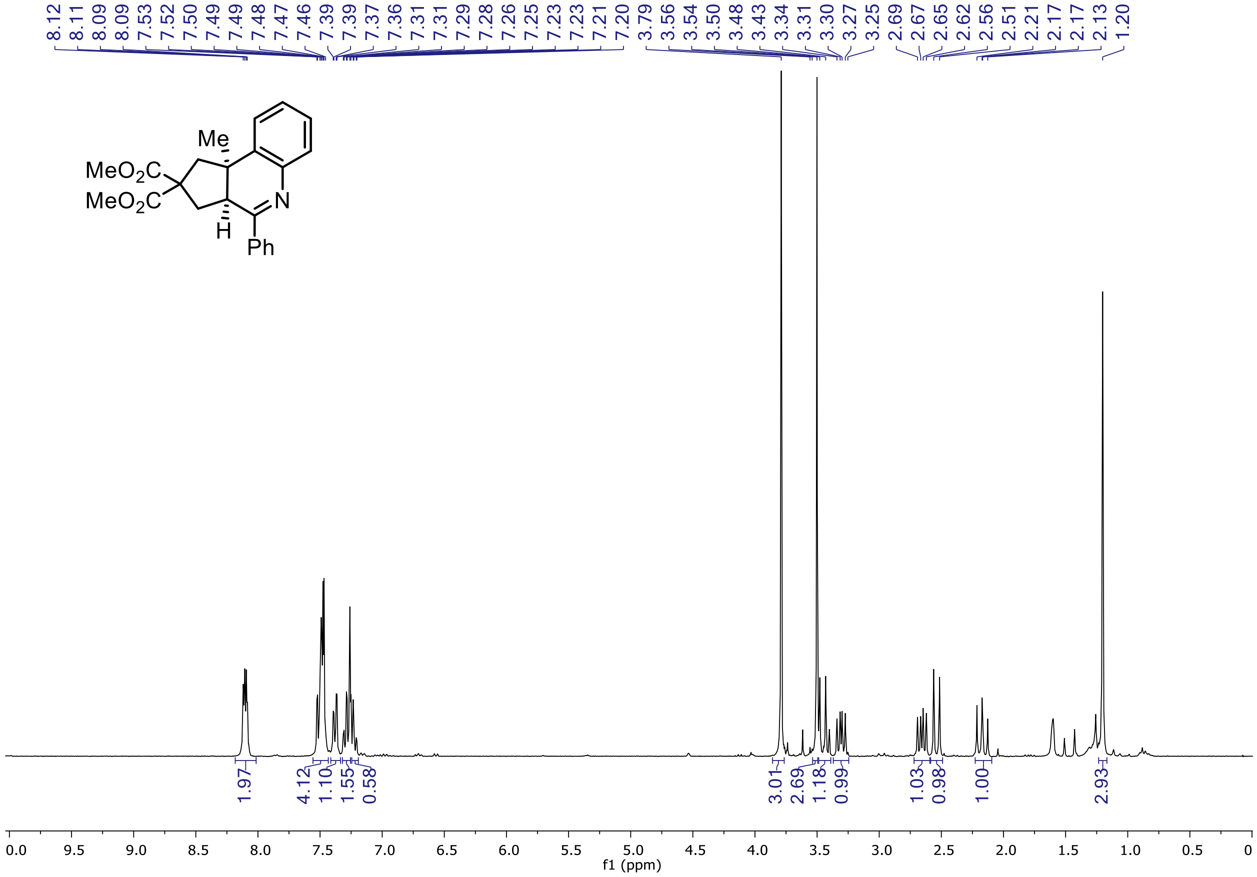

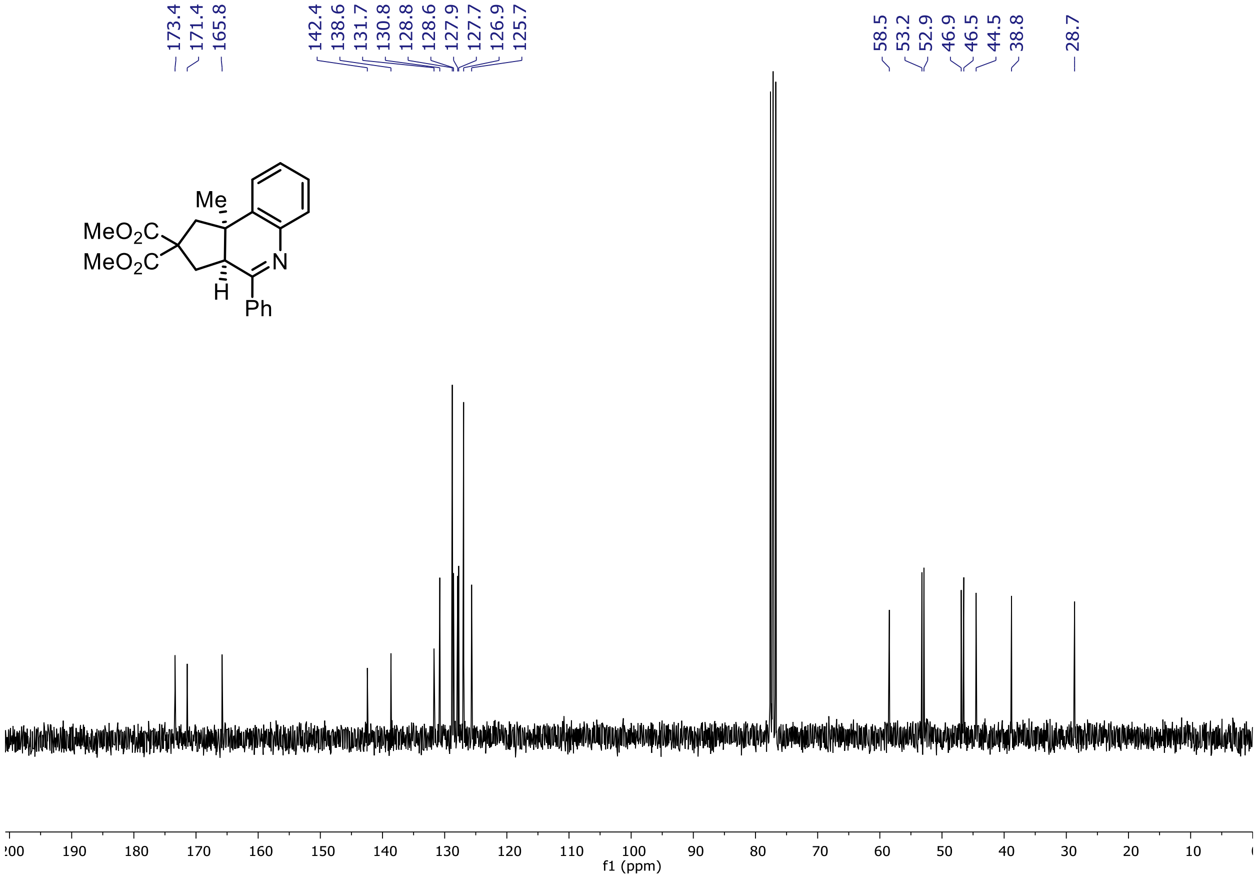

Compound **3c** ^{1}H and $^{13}C\{^{1}H\}$ NMR spectra in $CDCl_3$

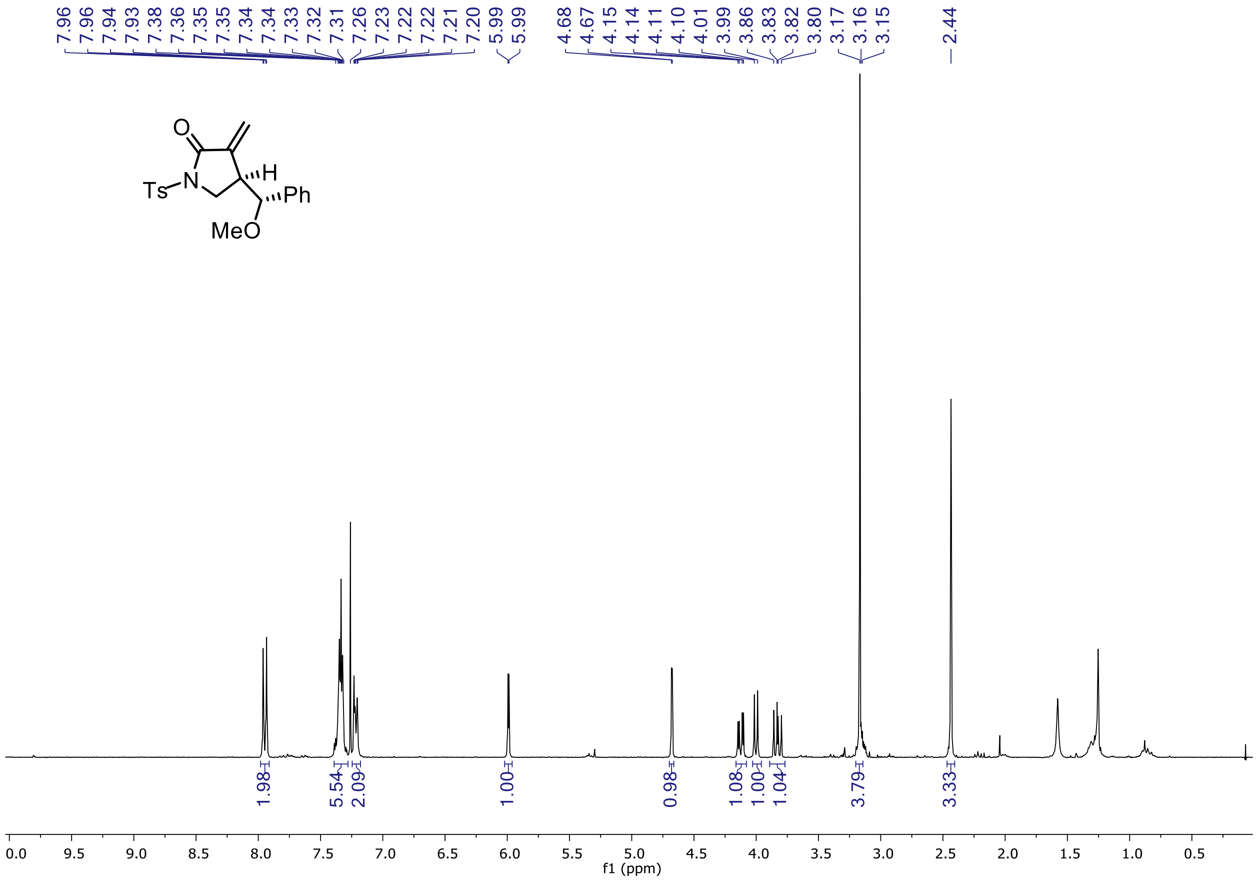

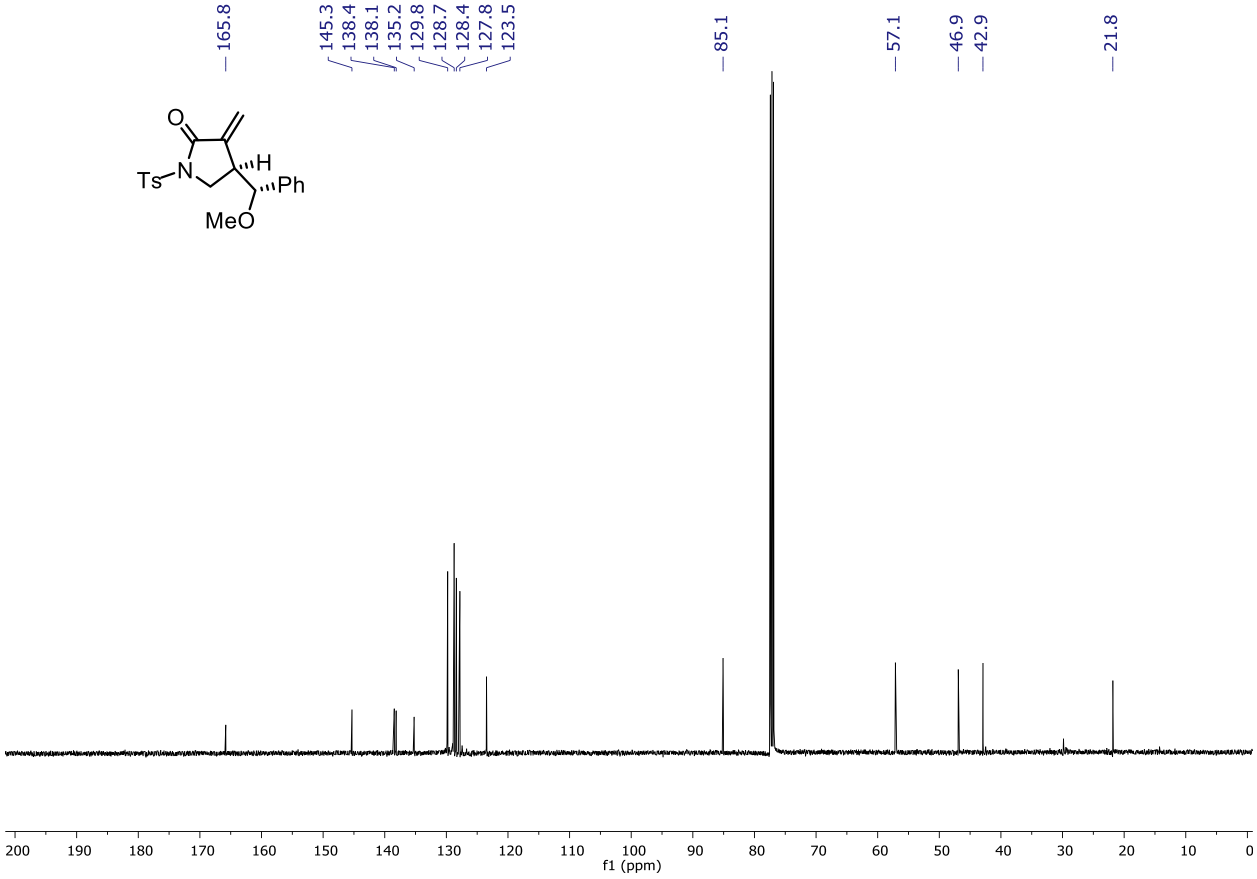

8. HPLC and SFC traces

Dimethyl (*R*)-3-((*R*)-(1H-indol-3-yl)(phenyl)methyl)-4-methylenecyclopentane-1,1-dicarboxylate (2a)

SFC (IG (100 × 3 mm, 3 μm), 80:20 CO_2:*i*-PrOH, 1.5 mL/min, 35 ºC, BPR 150 bar, 280 nm): en2 (100%) 1.53 min.

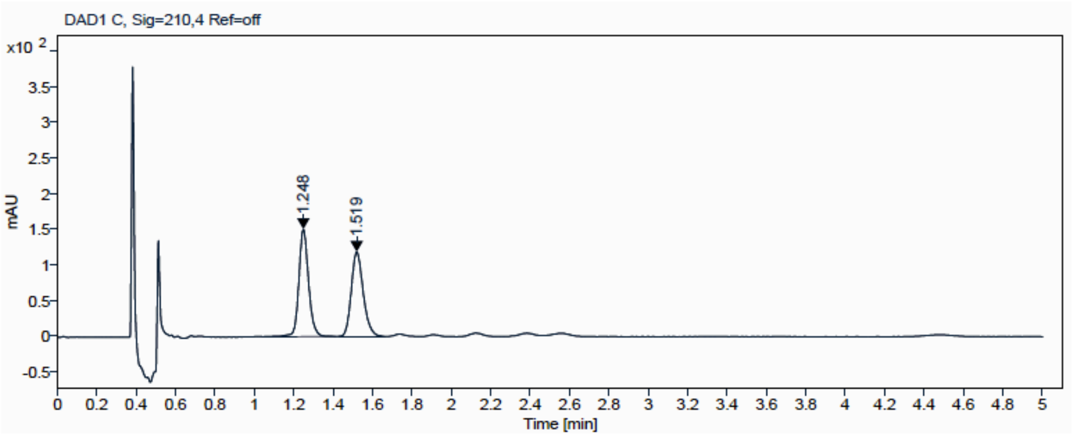

Signal: DAD1 C, Sig=210,4 Ref=off

RT [min]	Type	Width [min]	Area	Height	Area%	Name
1.248	VV R	0.0522	517.5399	150.1847	50.4569	
1.519	MM	0.0713	508.1678	118.8083	49.5431	

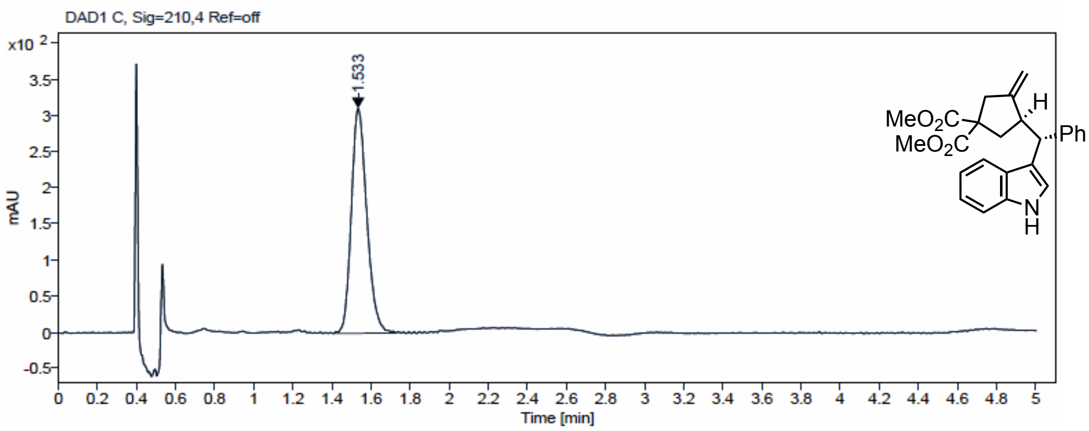

Signal: DAD1 C, Sig=210,4 Ref=off

RT [min]	Type	Width [min]	Area	Height	Area%	Name
1.533	VV R	0.0827	1713.3667	311.5497	100.0000	

Dimethyl (*R*)-3-((*R*)-(5-Bromo-1H-indol-3-yl)(phenyl)methyl)-4-methylenecyclopentane-1,1-dicarboxylate (2b)

SFC (IB-N (100 × 3 mm, 3 μm), 60:40 CO_2:MeOH, 1.2 mL/min, 35 ºC, BPR 150 bar, 210 nm): en1 (major, 100%) 0.99 min.

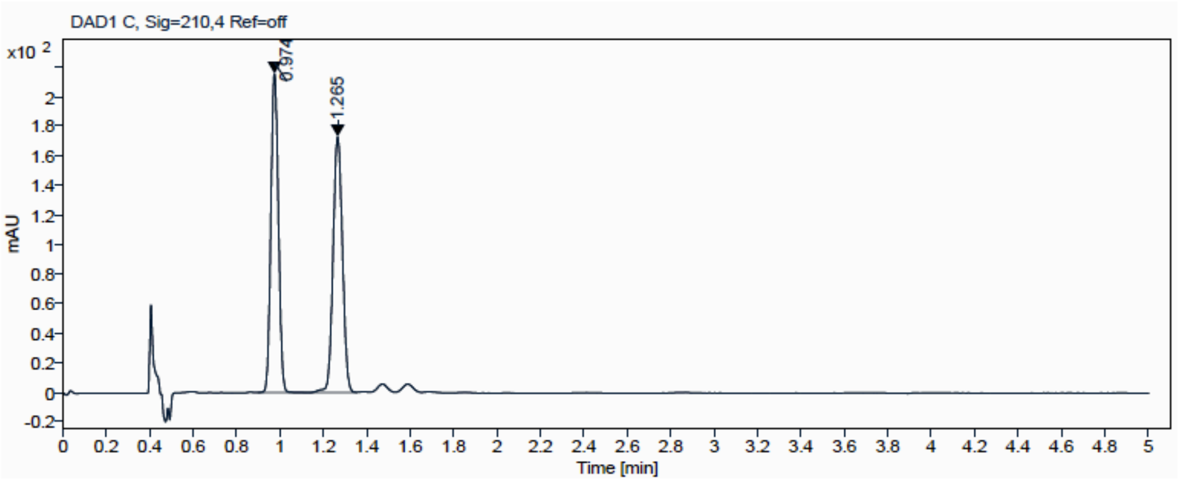

Signal: DAD1 C, Sig=210,4 Ref=off

RT [min]	Type	Width [min]	Area	Height	Area%	Name
0.974	BB	0.0380	517.6258	215.4048	49.8525	
1.265	BB	0.0477	520.6887	172.7875	50.1475	

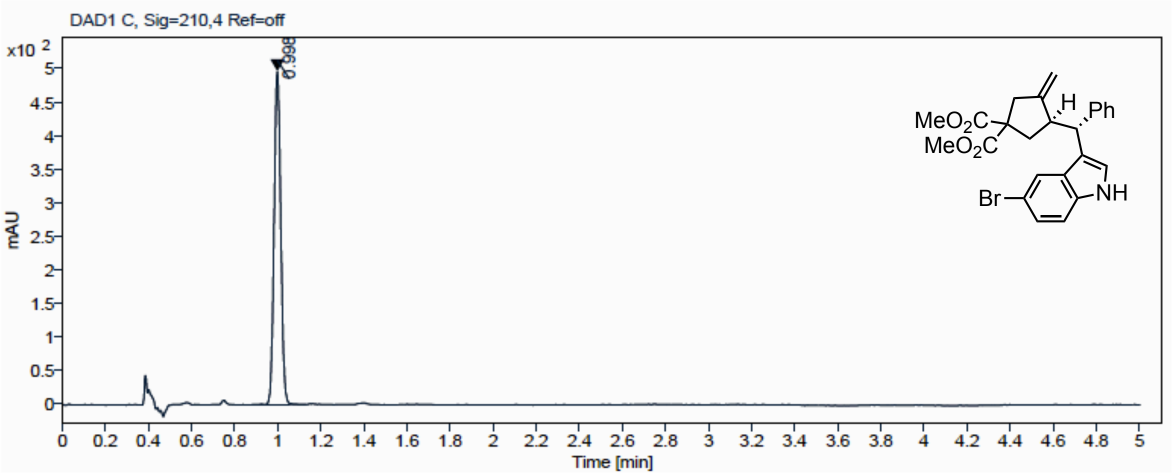

Signal: DAD1 C, Sig=210,4 Ref=off

RT [min]	Type	Width [min]	Area	Height	Area%	Name
0.998	VV R	0.0337	1071.2405	496.1527	100.0000	

Dimethyl (*R*)-3-((*R*)-(5-Methoxy-1H-indol-3-yl)(phenyl)methyl)-4-methylenecyclopentane-1,1-dicarboxylate (2c)

SFC (IB-N (100 × 3 mm, 3 μm), 80:30 CO_2:MeOH, 1.2 mL/min, 35 ºC, BPR 150 bar, 210 nm): en2 (major, 100%) min 1.25.

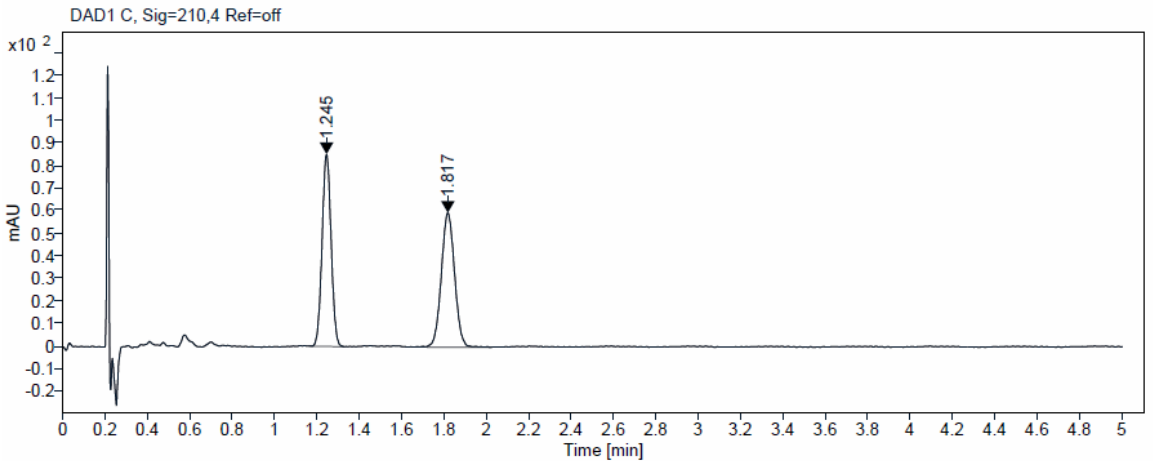

Signal: DAD1 C, Sig=210,4 Ref=off

RT [min]	Type	Width [min]	Area	Height	Area%	Name
1.245	BB	0.0460	252.0262	85.1069	49.2173	
1.817	VV R	0.0685	260.0419	59.4974	50.7827	

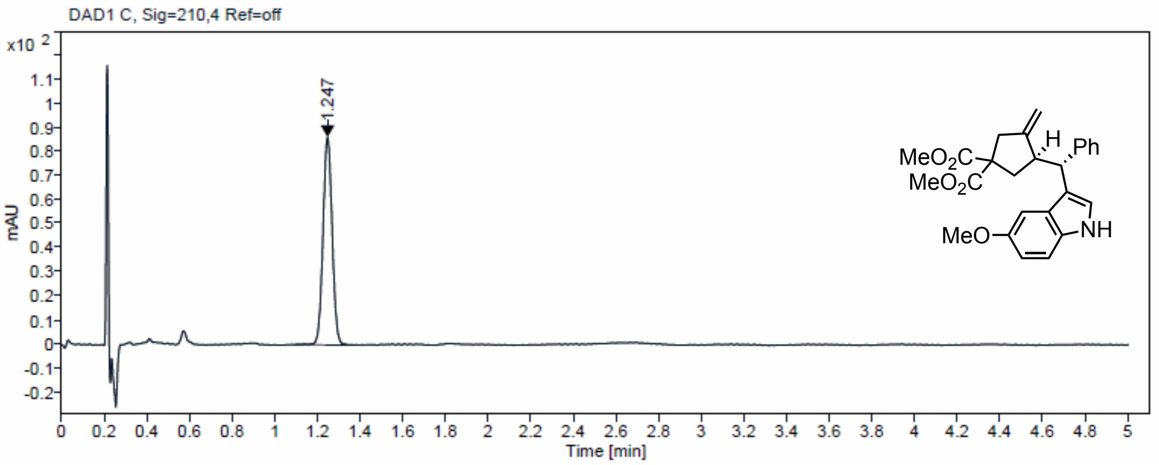

Signal: DAD1 C, Sig=210,4 Ref=off

RT [min]	Type	Width [min]	Area	Height	Area%	Name
1.247	VV R	0.0464	257.8689	86.3141	100.0000	

Dimethyl (*R*)-3-((*R*)-(1-Methyl-1H-indol-3-yl)(phenyl)methyl)-4-methylenecyclopentane-1,1-dicarboxylate (2d)

SFC (OD (100 × 3 mm, 3 μm), 80:20 CO_2:MeOH, 1.2 mL/min, 35 ºC, BPR 150 bar, 210 nm): en1 (major, 99%) 1.12 min, en2 (minor, 1%) 1.23 min.

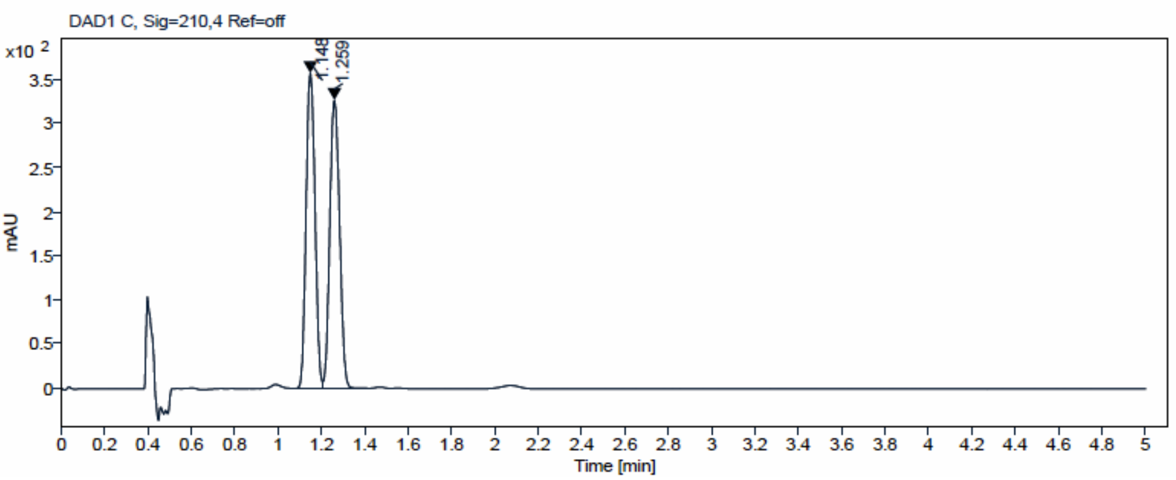

Signal: DAD1 C, Sig=210,4 Ref=off

RT [min]	Type	Width [min]	Area	Height	Area%	Name
1.148	BV	0.0458	1022.2726	357.6277	49.8722	
1.259	VV R	0.0502	1027.5138	327.0334	50.1278	

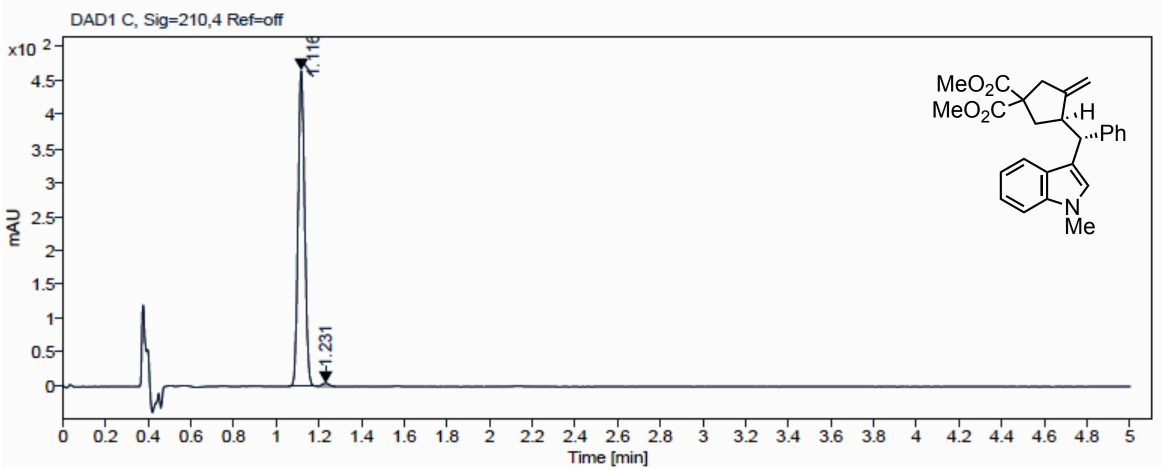

Signal: DAD1 C, Sig=210,4 Ref=off

RT [min]	Type	Width [min]	Area	Height	Area%	Name
1.116	MM	0.0357	996.5963	465.8330	98.9308	
1.231	MM	0.0388	10.7711	4.6273	1.0692	

Dimethyl (*R*)-3-((*R*)-(2-Methyl-1H-indol-3-yl)(phenyl)methyl)-4-methylenecyclopentane-1,1-dicarboxylate (2e)

SFC (IB-N (100 × 3 mm, 3 µm), 80:20 CO_2:*i*-PrOH, 1.2 mL/min, 35 ºC, BPR 150 bar, 210 nm): en2 (major, 100%) 3.33 min.

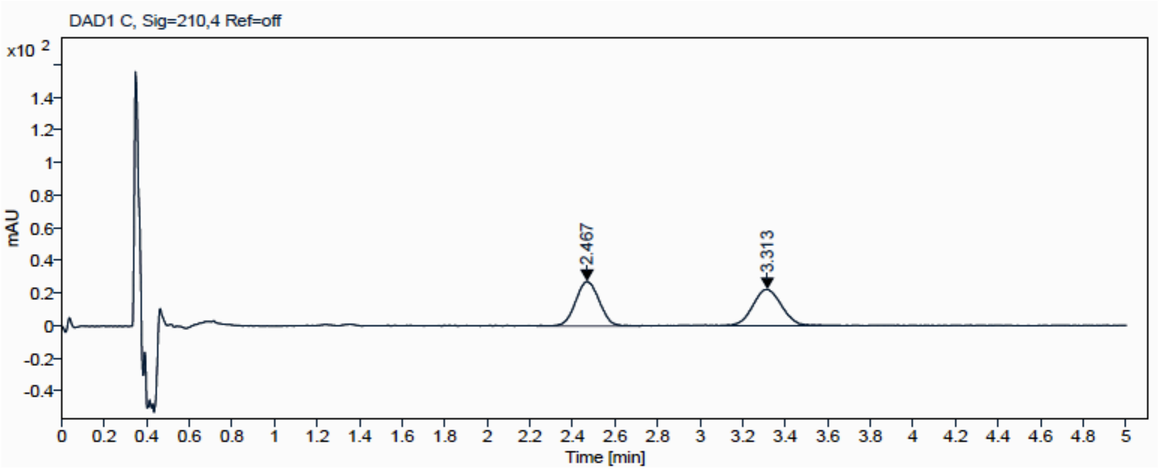

Signal: DAD1 C, Sig=210,4 Ref=off

RT [min]	Type	Width [min]	Area	Height	Area%	Name
2.467	VV R	0.1046	211.1951	27.3359	49.6433	
3.313	VV R	0.1235	214.2300	22.3221	50.3567	

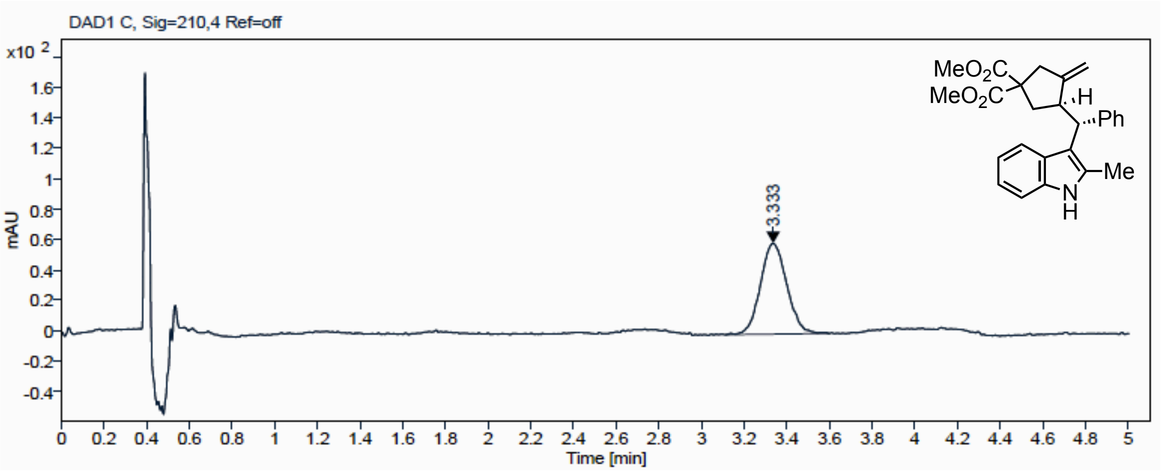

Signal: DAD1 C, Sig=210,4 Ref=off

RT [min]	Type	Width [min]	Area	Height	Area%	Name
3.333	VV R	0.1218	511.3831	59.4743	100.0000	

Dimethyl (*R*)-3-Methylene-4-((*R*)-phenyl(2,4,6-trimethoxyphenyl)methyl)cyclopentane-1,1-dicarboxylate (2f)

SFC (IG-3 (150 × 4.6 mm, 3 µm), 93:7 CO_2:*i*-PrOH, 2.0 mL/min, 35 ºC, BPR 140 bar, 230 nm): en1 (minor, 2%) 4.33 min, en2 (major, 98%) 4.72 min.

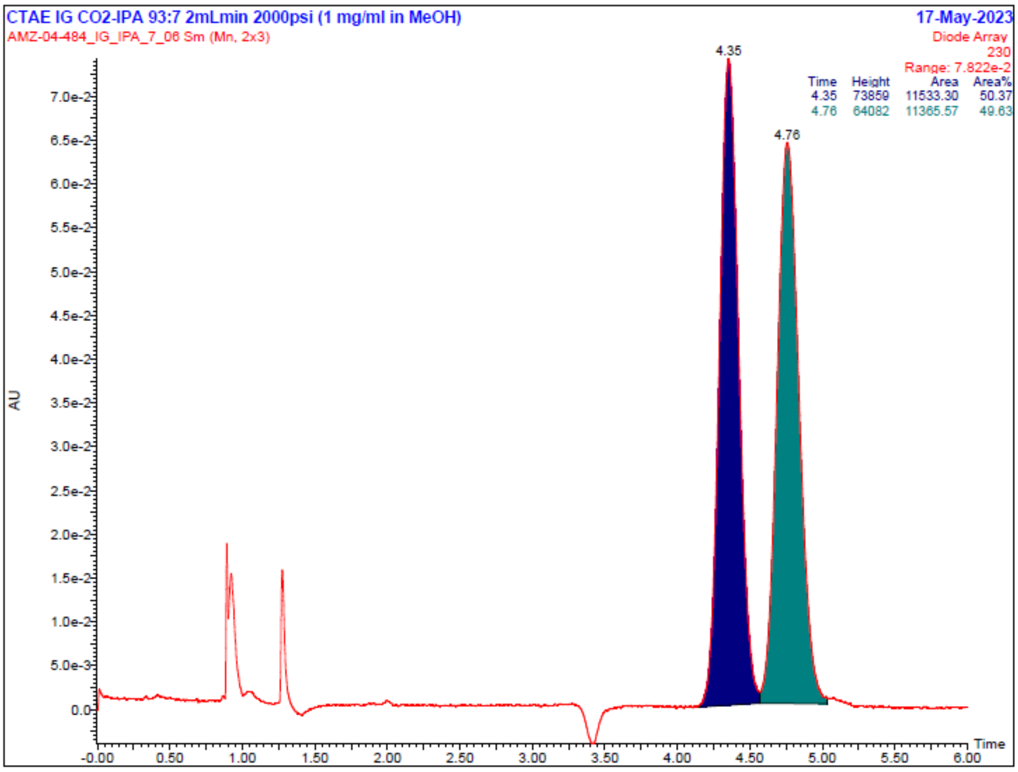

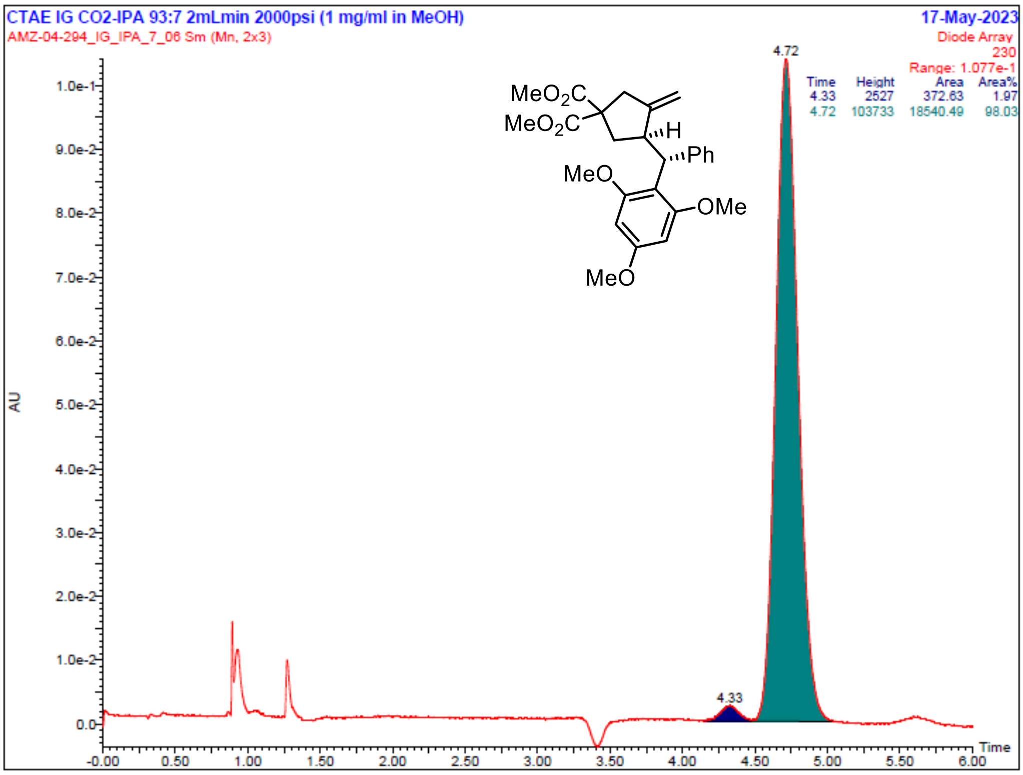

Dimethyl (*R*)-3-((*R*)-(2,4-Dimethoxyphenyl)(phenyl)methyl)-4-methylenecyclopentane-1,1-dicarboxylate (2g)

SFC (IB-N (100 × 3 mm, 3 μm), 90:10 CO_2:MeOH, 1.2 mL/min, 35 ºC, BPR 150 bar, 210 nm): en1 (minor, 1%) 1.20 min, en2 (major, 99%) 1.67 min.

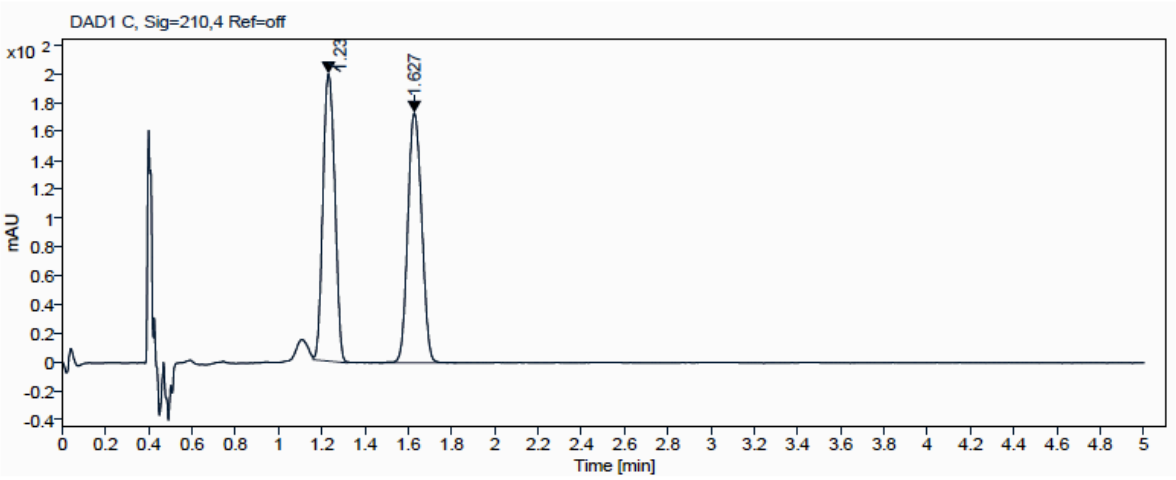

Signal: DAD1 C, Sig=210,4 Ref=off

RT [min]	Type	Width [min]	Area	Height	Area%	Name
1.230	MM	0.0637	762.2244	199.4647	49.6145	
1.627	VV R	0.0711	774.0703	173.4767	50.3855	

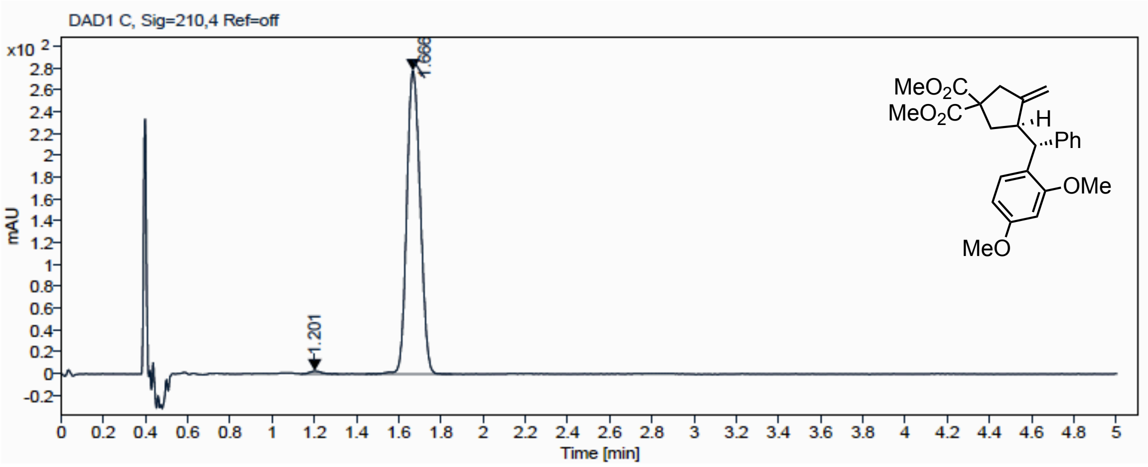

Signal: DAD1 C, Sig=210,4 Ref=off

RT [min]	Type	Width [min]	Area	Height	Area%	Name
1.201	BV R	0.0525	10.7651	2.7306	0.8640	
1.666	VV R	0.0708	1235.2424	278.2974	99.1360	

Dimethyl (*R*)-3-((*R*)-(4-(Dimethylamino)phenyl)(phenyl)methyl)-4-methylenecyclopentane-1,1-dicarboxylate (2h)

SFC (IB-N (100 × 3 mm, 3 μm), 80:20 CO_2:MeOH, 1.2 mL/min, 35 ºC, BPR 150 bar, 210 nm): en1 (minor, 0.5%) 1.11 min, en2 (major, 99.5%) 1.35 min.

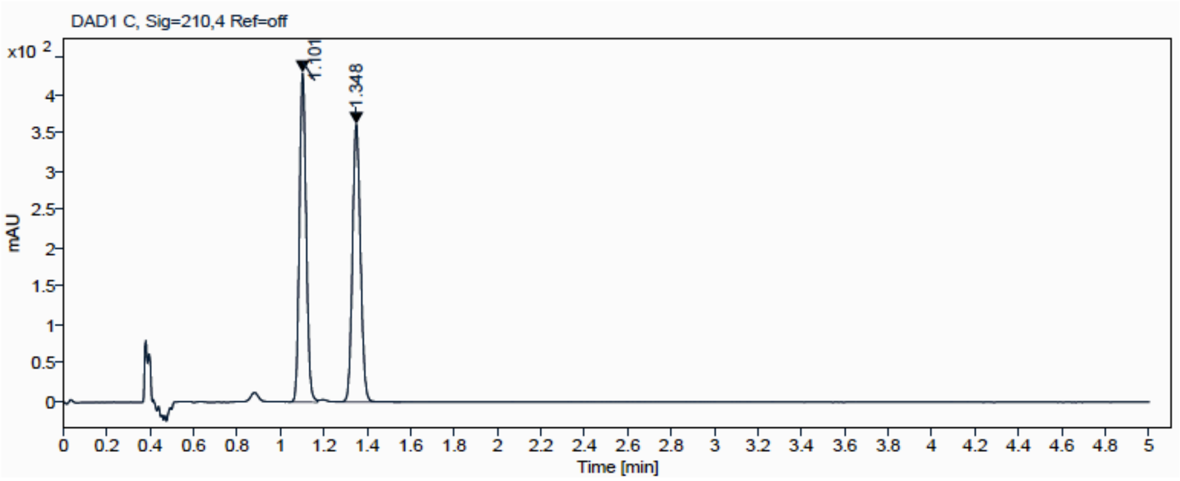

Signal: DAD1 C, Sig=210,4 Ref=off

RT [min]	Type	Width [min]	Area	Height	Area%	Name
1.101	MM	0.0361	931.6603	429.8140	50.0319	
1.348	VV R	0.0395	930.4731	362.0683	49.9681	

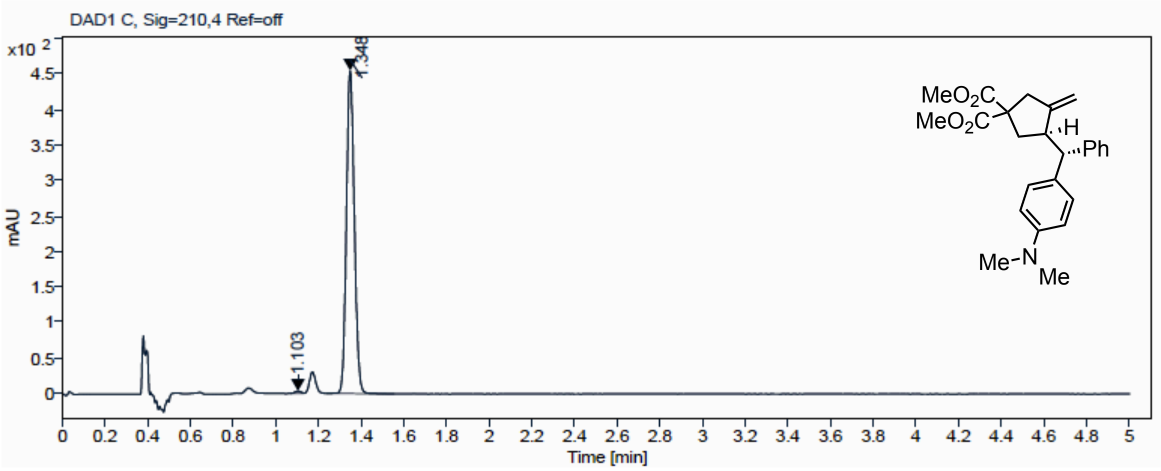

Signal: DAD1 C, Sig=210,4 Ref=off

RT [min]	Type	Width [min]	Area	Height	Area%	Name
1.103	MM	0.0340	6.8119	3.3351	0.5755	
1.348	VV R	0.0402	1176.8610	455.3448	99.4245	

Dimethyl (*S*)-3-Methylene-4-((*R*)-phenyl(phenylamino)methyl)cyclopentane-1,1-dicarboxylate (2i)

SFC (IG (100 × 3 mm, 3 μm), 80:20 CO_2:EtOH, 1.2 mL/min, 35 ºC, BPR 150 bar, 210 nm): en1 (major, 100%) 0.75 min.

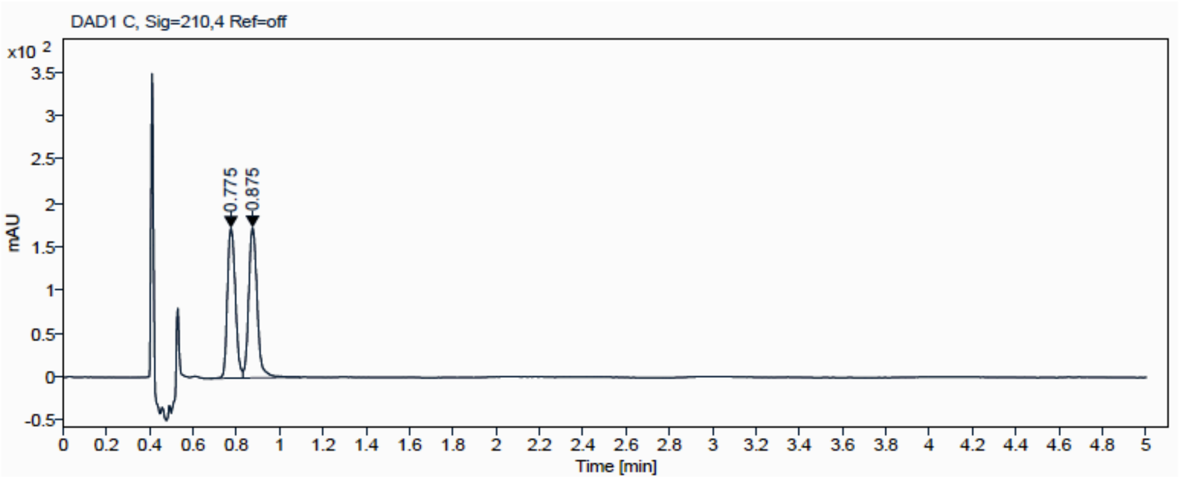

Signal: DAD1 C, Sig=210,4 Ref=off

RT [min]	Type	Width [min]	Area	Height	Area%	Name
0.775	BV	0.0408	439.4219	172.0909	48.2864	
0.875	VV R	0.0429	470.6097	172.3547	51.7136	

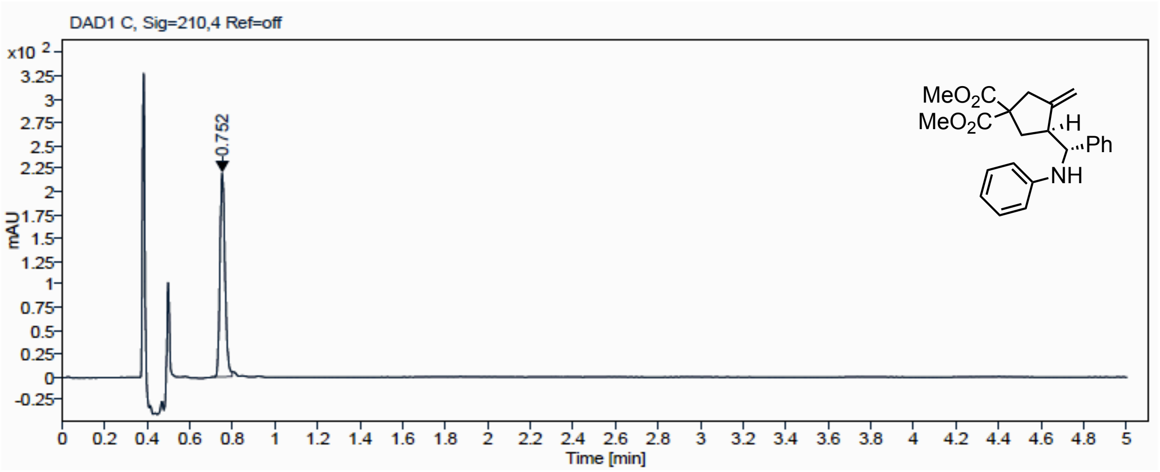

Signal: DAD1 C, Sig=210,4 Ref=off

RT [min]	Type	Width [min]	Area	Height	Area%	Name
0.752	MM	0.0275	364.3580	220.5252	100.0000	

Dimethyl (*S*)-3-((*R*)-((2-Bromophenyl)amino)(phenyl)methyl)-4-methylenecyclopentane-1,1-dicarboxylate (2j)

SFC (IG (100 × 3 mm, 3 µm), 95:5 CO_2:*i*-PrOH, 1.5 mL/min, 35 ºC, BPR 150 bar, 210 nm): en1 (major, 100%) 2.08 min.

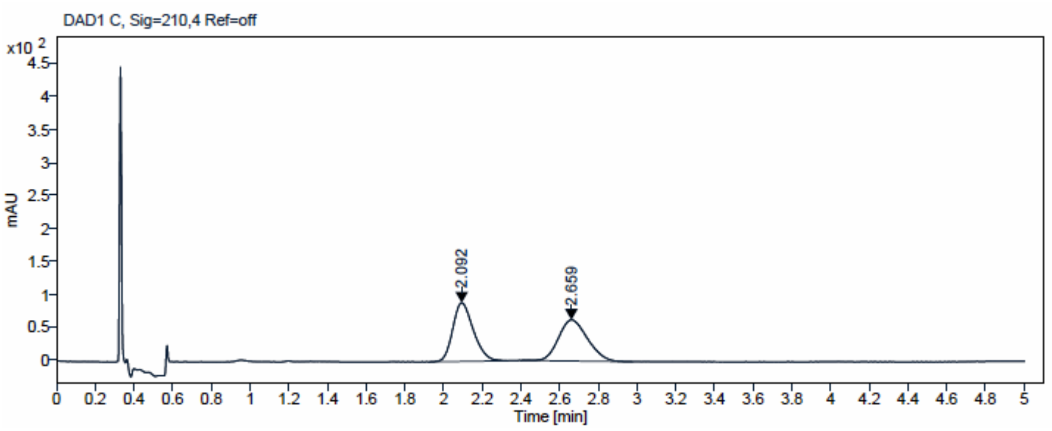

Signal: DAD1 C, Sig=210,4 Ref=off

RT [min]	Type	Width [min]	Area	Height	Area%	Name
2.092	VV R	0.1120	659.6279	88.9343	50.2981	
2.659	VV R	0.1561	651.8096	62.3031	49.7019	

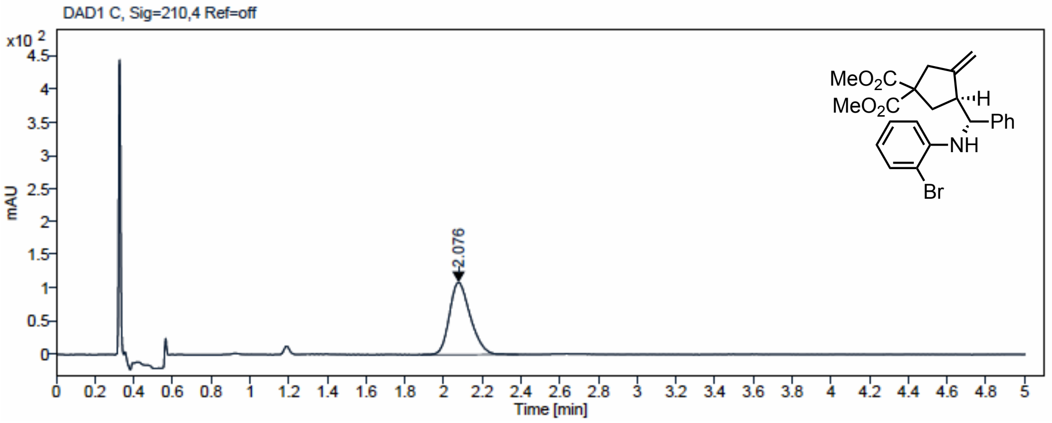

Signal: DAD1 C, Sig=210,4 Ref=off

RT [min]	Type	Width [min]	Area	Height	Area%	Name
2.076	VV R	0.1132	821.1177	109.1832	100.0000	

Dimethyl (*S*)-3-((*R*)-((2-Iodophenyl)amino)(phenyl)methyl)-4-methylenecyclopentane-1,1-dicarboxylate (2k)

SFC (OD (100 × 3 mm, 3 μm), 95:5 CO_2:*i*-PrOH, 1.2 mL/min, 35 ºC, BPR 150 bar, 210 nm): en1 (minor, 1.5%) 2.87 min, en2 (major, 98.5%) 3.53 min.

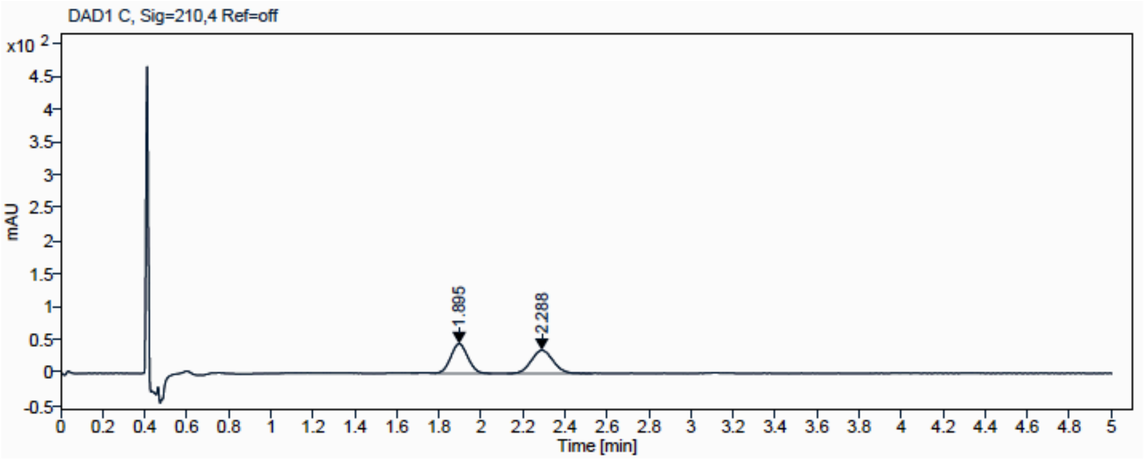

Signal: DAD1 C, Sig=210,4 Ref=off

RT [min]	Type	Width [min]	Area	Height	Area%	Name
1.895	VB R	0.0912	257.9467	44.7999	50.4242	
2.288	VV R	0.1064	253.6069	35.0712	49.5758	

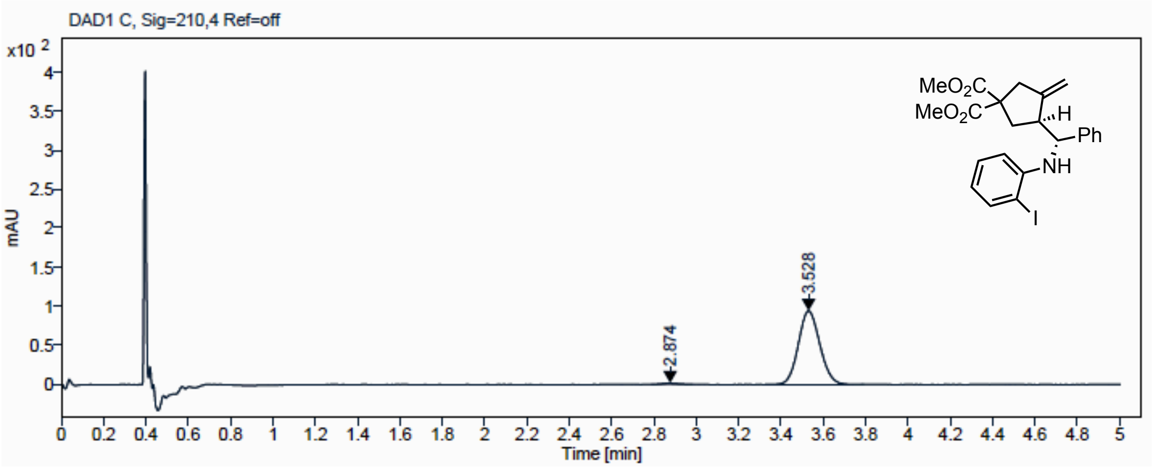

Signal: DAD1 C, Sig=210,4 Ref=off

RT [min]	Type	Width [min]	Area	Height	Area%	Name
2.874	BB	0.0621	9.0828	1.8094	1.3364	
3.528	VV R	0.1071	670.5387	94.6883	98.6636	

Dimethyl (*S*)-3-((*R*)-(((Benzyloxy)carbonyl)amino)(phenyl)methyl)-4-methylenecyclopentane-1,1-dicarboxylate (2l)

SFC (IG (100 × 3 mm, 3 μm), 75:25 CO_2:EtOH, 1.2 mL/min, 35 ºC, BPR 150 bar, 210 nm): en1 (major, 100%) 1.69 min.

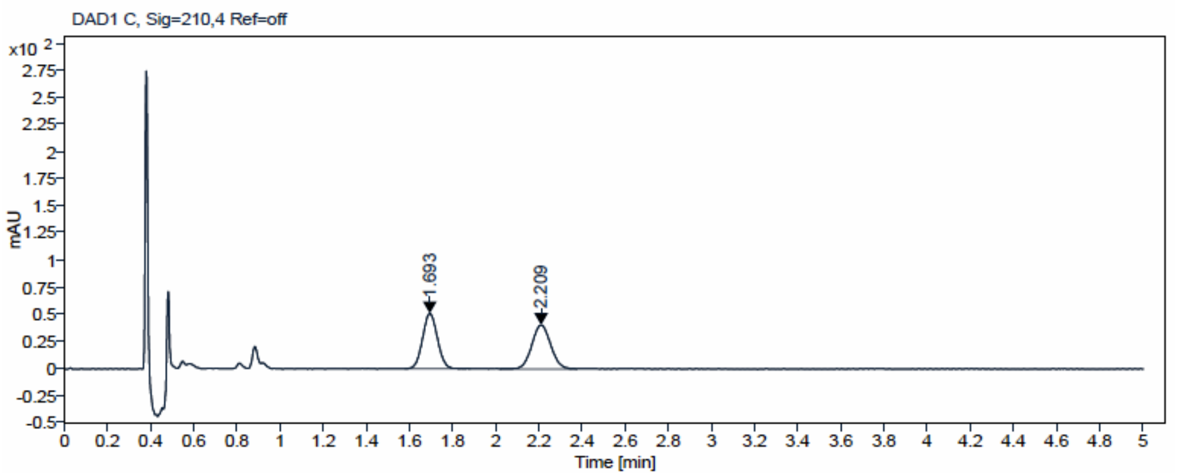

Signal: DAD1 C, Sig=210,4 Ref=off

RT [min]	Type	Width [min]	Area	Height	Area%	Name
1.693	BV R	0.0739	246.7306	51.0692	50.0388	
2.209	VV R	0.0971	246.3481	40.4159	49.9612	

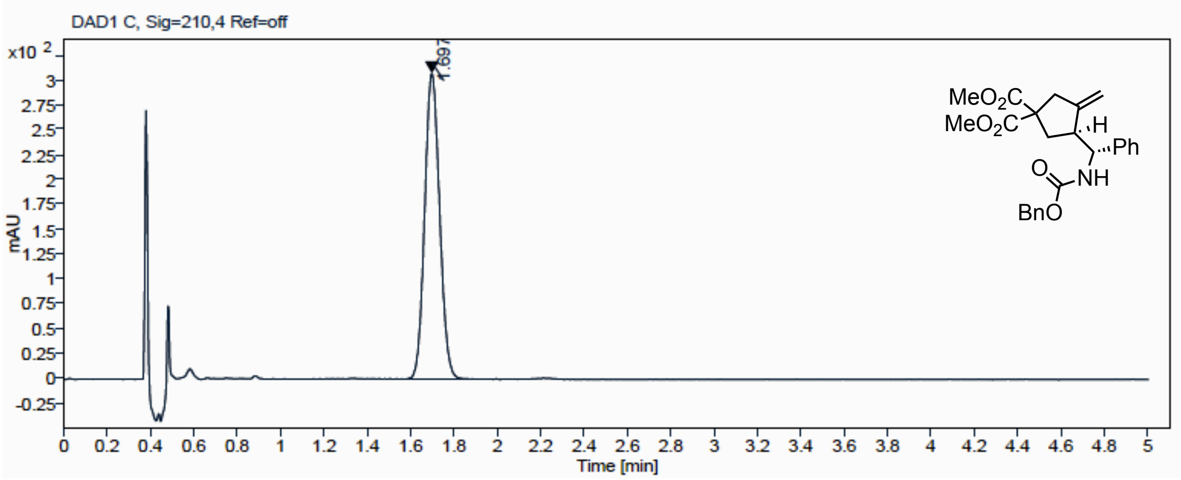

Signal: DAD1 C, Sig=210,4 Ref=off

RT [min]	Type	Width [min]	Area	Height	Area%	Name
1.697	BV R	0.0748	1471.3632	307.4377	100.0000	

Dimethyl (*S*)-3-((*R*)-Hydroxy(phenyl)methyl)-4-methylenecyclopentane-1,1-dicarboxylate (2m)

SFC (IG (100 × 3 mm, 3 μm), 85:15 CO_2:MeOH, 1.2 mL/min, 35 ºC, BPR 150 bar, 210 nm): en1 (major, 98%) 1.31 min, en2 (minor, 2%) 1.49 min.

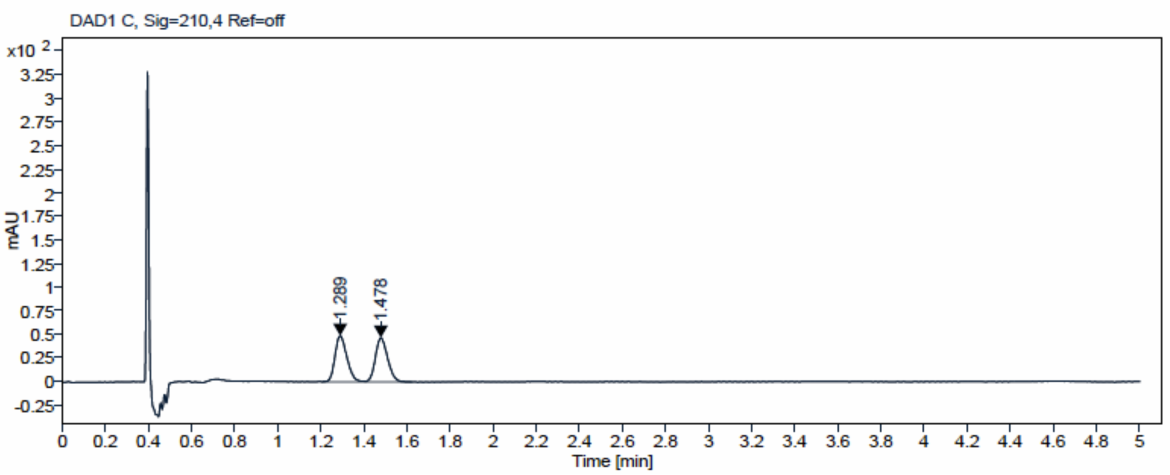

Signal: DAD1 C, Sig=210,4 Ref=off

RT [min]	Type	Width [min]	Area	Height	Area%	Name
1.289	BV R	0.0584	186.9551	48.5936	50.3319	
1.478	VV R	0.0621	184.4896	46.6599	49.6681	

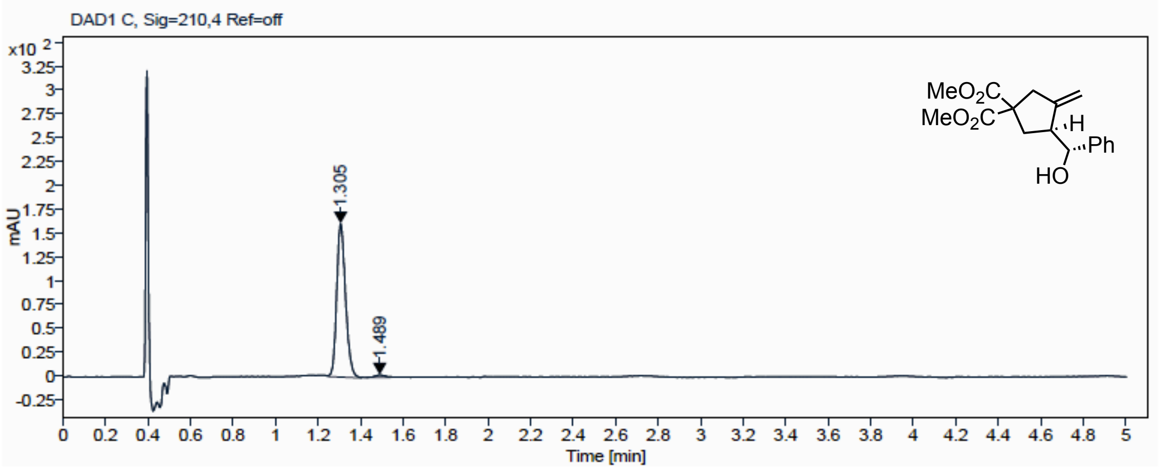

Signal: DAD1 C, Sig=210,4 Ref=off

RT [min]	Type	Width [min]	Area	Height	Area%	Name
1.305	MM	0.0481	464.2198	160.9637	98.1356	
1.489	BV R	0.0435	8.8196	2.6715	1.8644	

Dimethyl (*S*)-3-((*R*)-Methoxy(phenyl)methyl)-4-methylenecyclopentane-1,1-dicarboxylate (2n)

HPLC (OJ-H (250 × 4.6 mm, 5 μm), 99.5:0.5 hexane:*i*-PrOH, 1.0 mL/min, 25 °C, 210 nm): en1 (major, 99.5%) 17.99 min, (minor, 0.5%) 20.25 min.

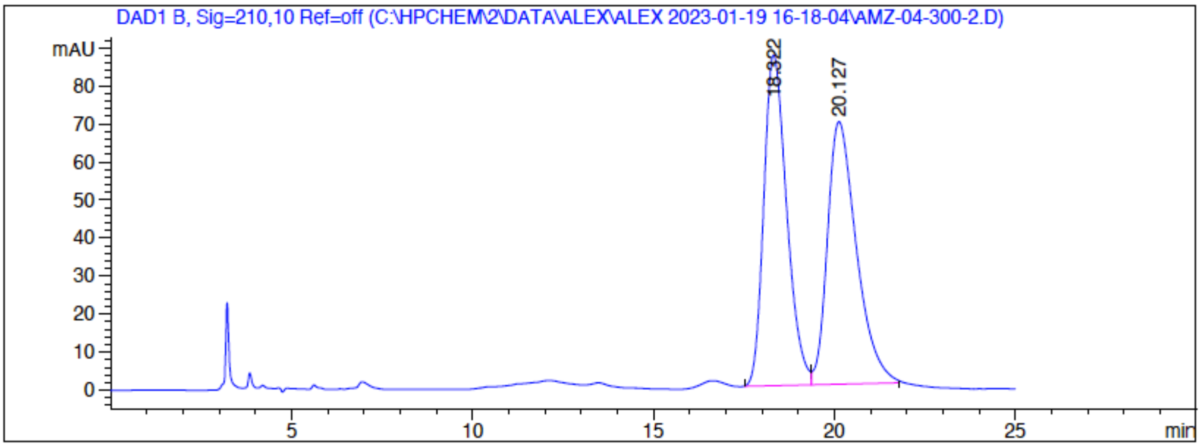

Signal 2: DAD1 B, Sig=210,10 Ref=off

Peak #	RetTime [min]	Type	Width [min]	Area [mAU*s]	Height [mAU]	Area %
1	18.322	BV	0.6805	3833.48096	86.92737	49.9355
2	20.127	VB	0.8461	3843.38916	68.97473	50.0645

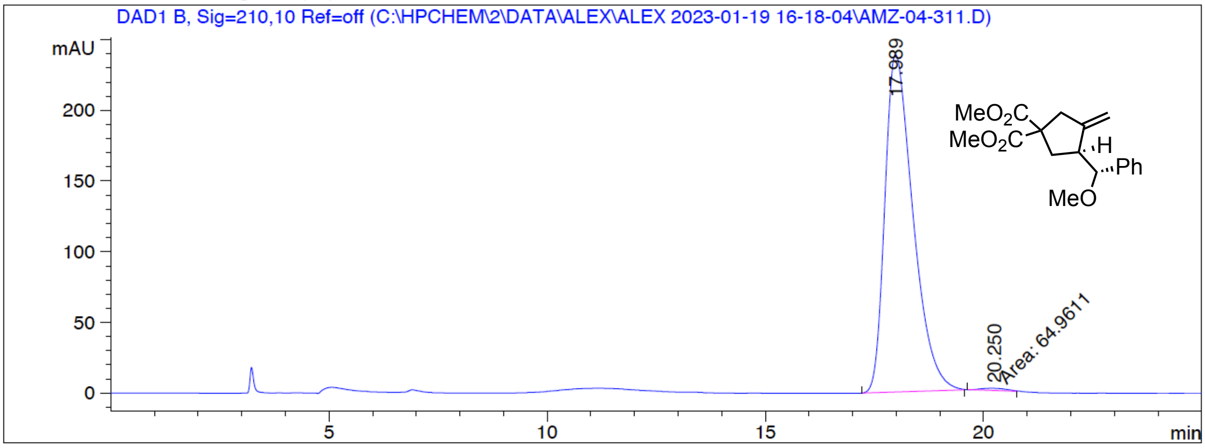

Peak #	RetTime [min]	Type	Width [min]	Area [mAU*s]	Height [mAU]	Area %
1	17.989	BB	0.6938	1.07620e4	238.76918	99.4000
2	20.250	MM	0.6650	64.96105	1.62816	0.6000

Dimethyl (*S*)-3-((*R*)-Methoxy(phenyl)methyl)-4-methylenecyclopentane-1,1-dicarboxylate (2o)

HPLC (OJ-H (250 × 4.6 mm, 5 µm), 99:1 hexane:*i*-PrOH, 1.0 mL/min, 25 °C, 210 nm): en1 (major, 98.5%) 20.48 min, en2 (minor, 1.5%) 25.91 min.

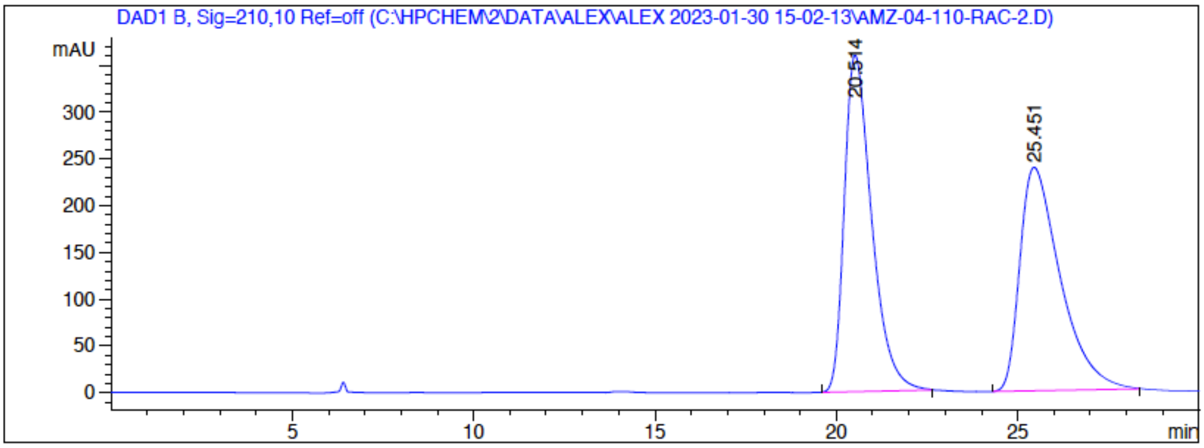

Peak #	RetTime [min]	Type	Width [min]	Area [mAU*s]	Height [mAU]	Area %
1	20.514	BB	0.8038	1.88764e4	359.98361	50.4546
2	25.451	BB	1.1450	1.85362e4	238.94757	49.5454

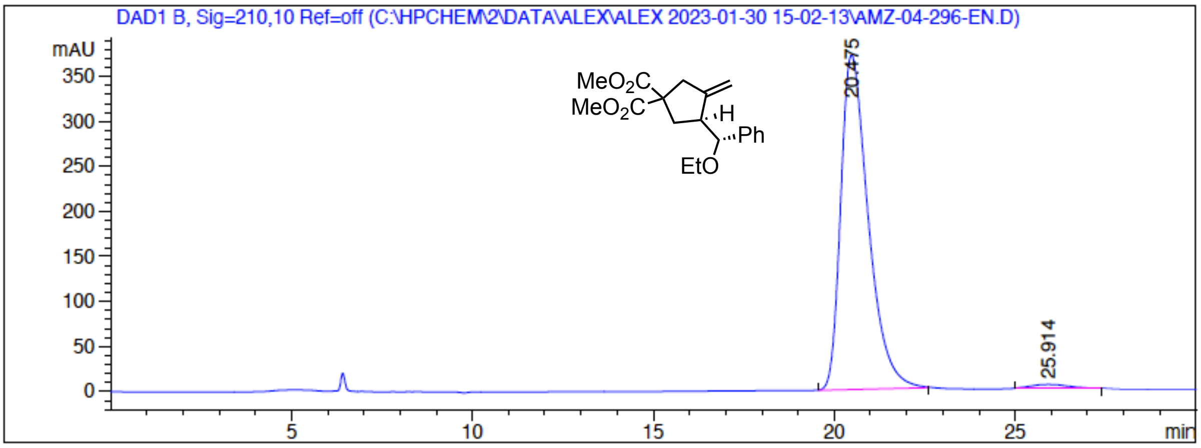

Peak #	RetTime [min]	Type	Width [min]	Area [mAU*s]	Height [mAU]	Area %
1	20.475	BB	0.8062	1.96247e4	371.55560	98.4446
2	25.914	BB	0.8466	310.06226	4.38682	1.5554

Dimethyl (*S*)-3-((*R*)-Isopropoxy(phenyl)methyl)-4-methylenecyclopentane-1,1-dicarboxylate (2p)

HPLC (OJ-H (250 × 4.6 mm, 5 μm), 99:1 hexane:*i*-PrOH, 1.0 mL/min, 25 °C, 210 nm): en1 (major, 100%) 18.83 min.

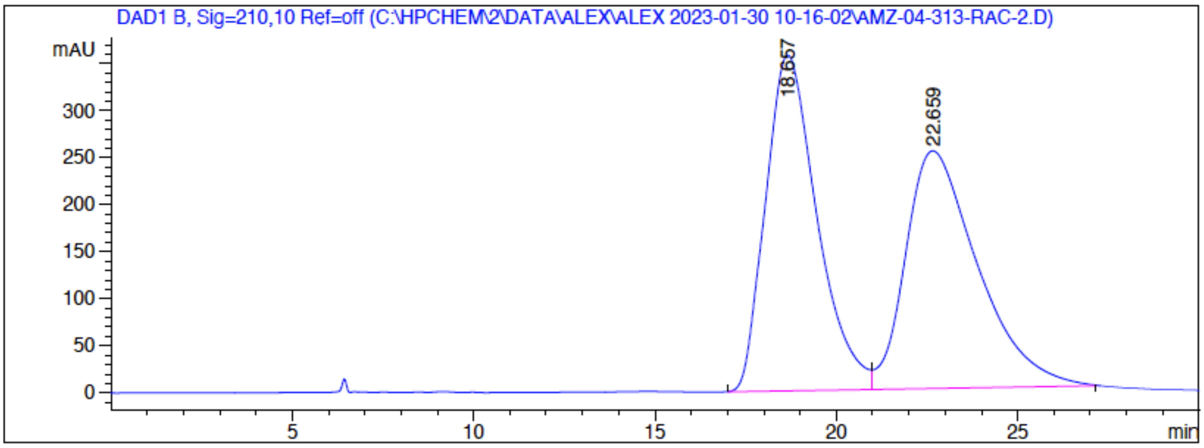

Signal 2: DAD1 B, Sig=210,10 Ref=off

Peak #	RetTime [min]	Type	Width [min]	Area [mAU*s]	Height [mAU]	Area %
1	18.657	BV	1.4643	3.52150e4	357.17358	50.1686
2	22.659	VB	2.0092	3.49784e4	252.65797	49.8314

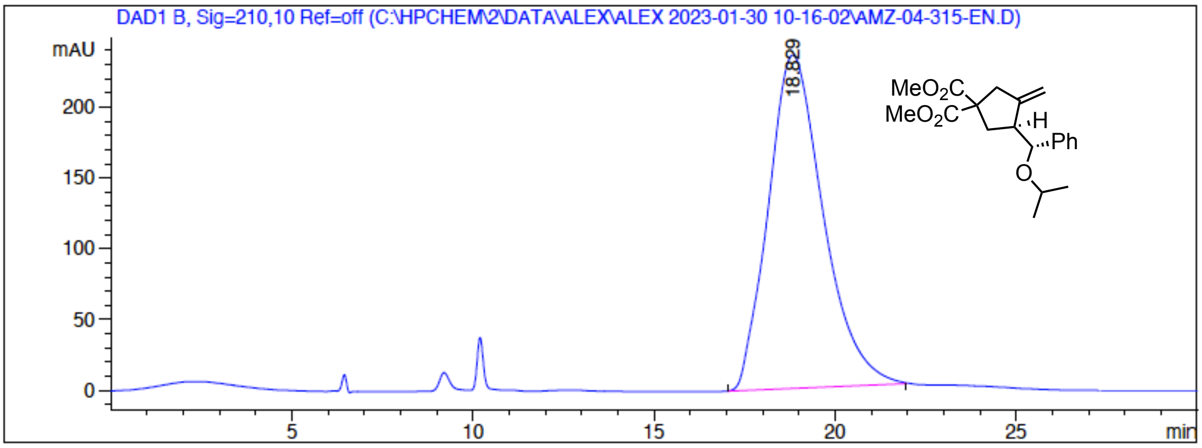

Signal 2: DAD1 B, Sig=210,10 Ref=off

Peak #	RetTime [min]	Type	Width [min]	Area [mAU*s]	Height [mAU]	Area %
1	18.829	BB	1.5859	2.48413e4	235.26097	100.0000

Dimethyl (*S*)-3-((*R*)-(Benzyloxy)(phenyl)methyl)-4-methylenecyclopentane-1,1-dicarboxylate (2q)

SFC (OJ (100 × 3 mm, 3 μm), 99:1 CO_2:MeOH, 1.2 mL/min, 35 ºC, BPR 150 bar, 210 nm): en1 (major, 98.5%) 1.76 min, en2 (minor, 1.5%) 2.45 min.

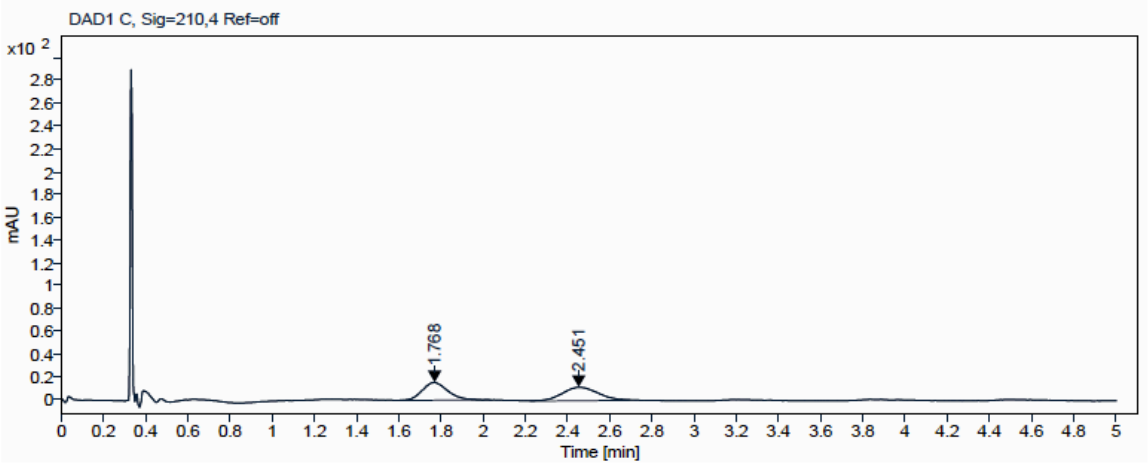

Signal: DAD1 C, Sig=210,4 Ref=off

RT [min]	Type	Width [min]	Area	Height	Area%	Name
1.768	BV R	0.1041	131.1833	15.5155	48.2200	
2.451	MM	0.1978	140.8685	11.8717	51.7800	

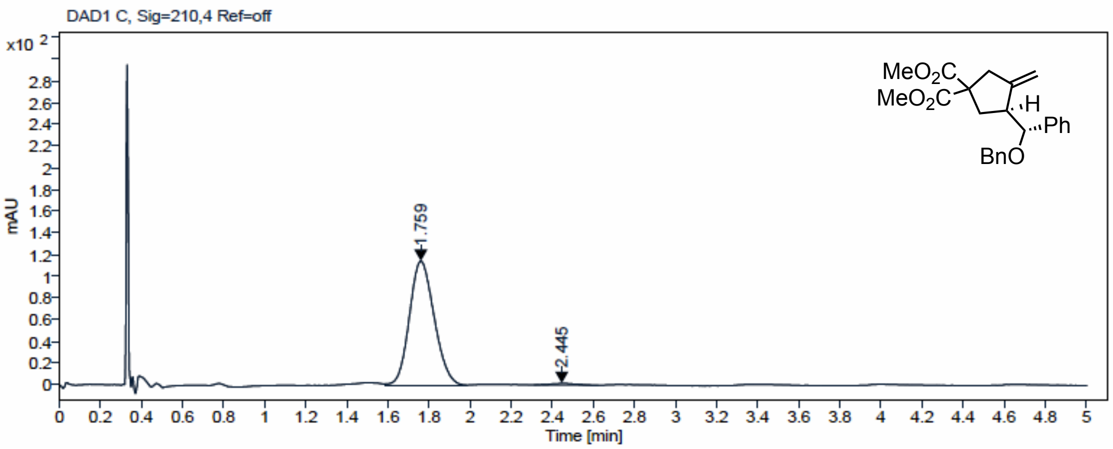

Signal: DAD1 C, Sig=210,4 Ref=off

RT [min]	Type	Width [min]	Area	Height	Area%	Name
1.759	MM	0.1398	961.7709	114.6381	98.5246	
2.445	BV R	0.1047	14.4028	1.6940	1.4754	

Dimethyl (*S*)-3-((*R*)-(Allyloxy)(phenyl)methyl)-4-methylenecyclopentane-1,1-dicarboxylate (2r)

HPLC (OJ-H (250 × 4.6 mm, 5 µm), 99:1 hexane:*i*-PrOH, 1.0 mL/min, 25 °C, 210 nm): en1 (major, 98.5%) 8.59 min, en2 (minor, 1.5%).

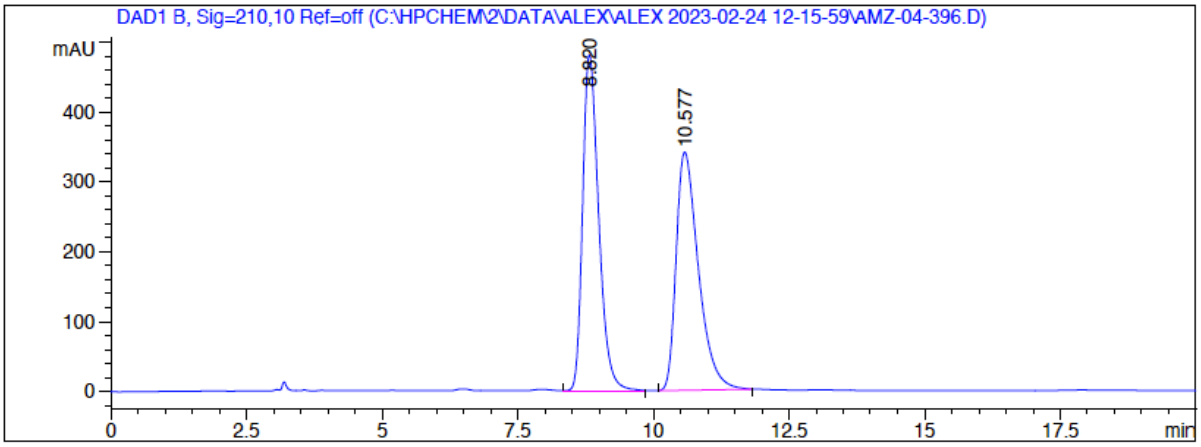

Signal 2: DAD1 B, Sig=210,10 Ref=off

Peak #	RetTime [min]	Type	Width [min]	Area [mAU*s]	Height [mAU]	Area %
1	8.820	VB	0.3176	9891.60742	481.61716	50.3829
2	10.577	BB	0.4349	9741.26660	341.37454	49.6171

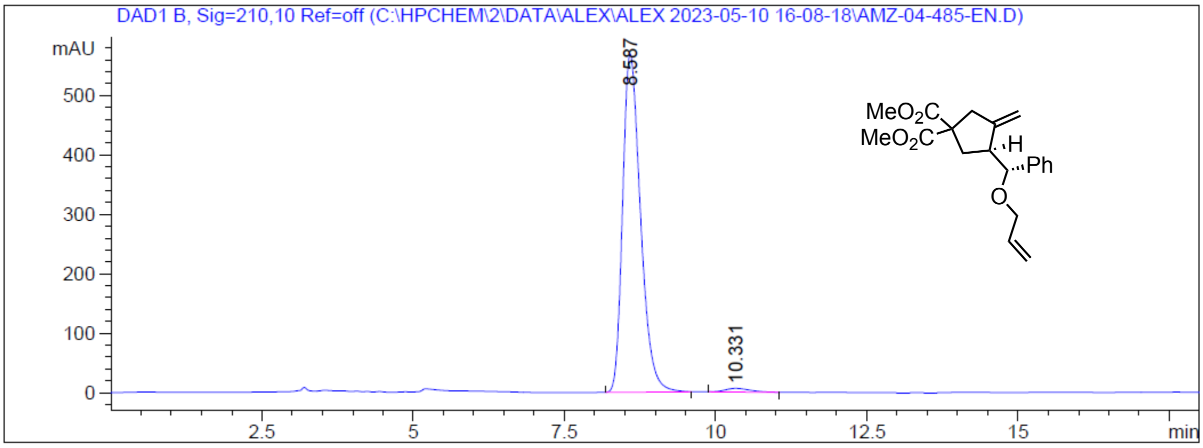

Signal 1: DAD1 A, Sig=220,10 Ref=off

Peak #	RetTime [min]	Type	Width [min]	Area [mAU*s]	Height [mAU]	Area %
1	8.588	BB	0.3152	4772.66602	232.79164	98.6088
2	10.336	BB	0.4090	67.33331	2.49366	1.3912

Dimethyl (*S*)-3-Methylene-4-((*R*)-((3-oxocyclohex-1-en-1-yl)oxy)(phenyl)methyl)cyclopentane-1,1-dicarboxylate (2s)

SFC (IC (100 × 3 mm, 3 μm), 80:20 CO_2:EtOH, 1.2 mL/min, 35 ºC, BPR 150 bar, 210 nm): en1 (major, 97.5%) 1.55 min, en2 (minor, 2.5%) 1.91 min.

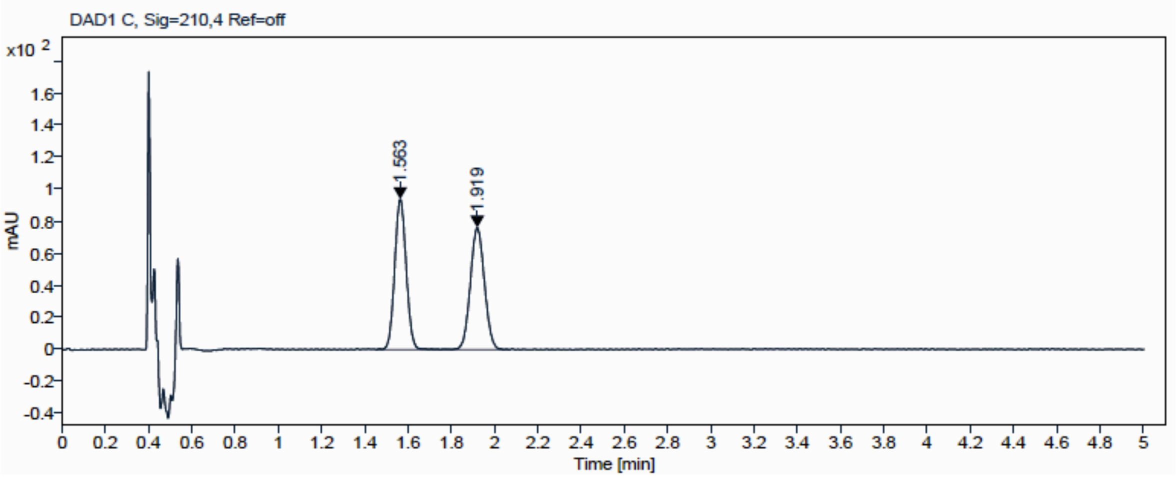

Signal: DAD1 C, Sig=210,4 Ref=off

RT [min]	Type	Width [min]	Area	Height	Area%	Name
1.563	VV R	0.0572	347.8777	94.0484	50.0706	
1.919	VV R	0.0706	346.8964	76.2137	49.9294	

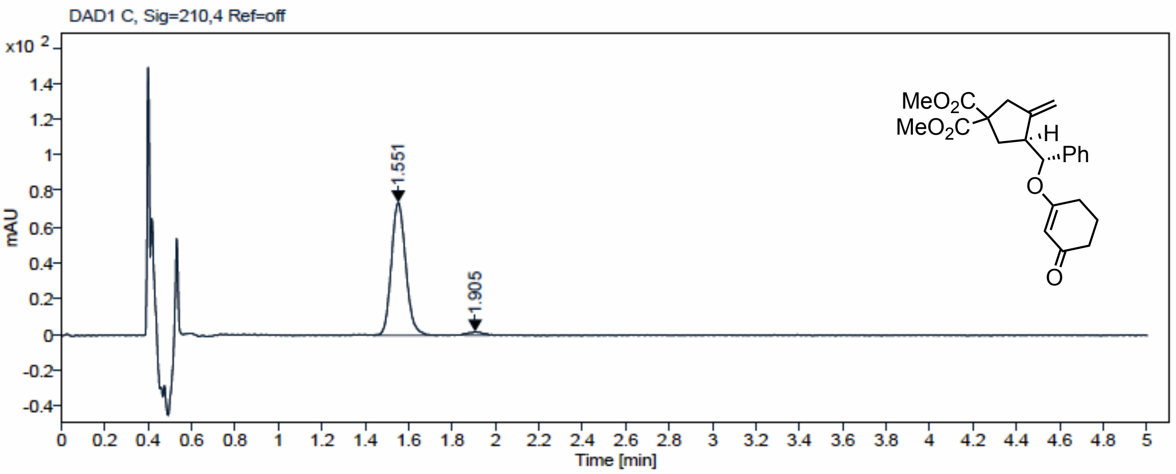

Signal: DAD1 C, Sig=210,4 Ref=off

RT [min]	Type	Width [min]	Area	Height	Area%	Name
1.551	VB R	0.0729	342.7358	74.2280	97.4464	
1.905	MM	0.0791	8.9815	1.8920	2.5536	

Dimethyl (*R*)-3-((*R*)-(1H-indol-3-yl)(4-(trifluoromethyl)phenyl)methyl)-4-methylenecyclopentane-1,1-dicarboxylate (2t)

SFC (OD (100 × 3 mm, 3 μm), 85:15 CO_2:*i*-PrOH, 1.2 mL/min, 35 ºC, BPR 150 bar, 210 nm): en2 (major, 100%) 1.74 min.

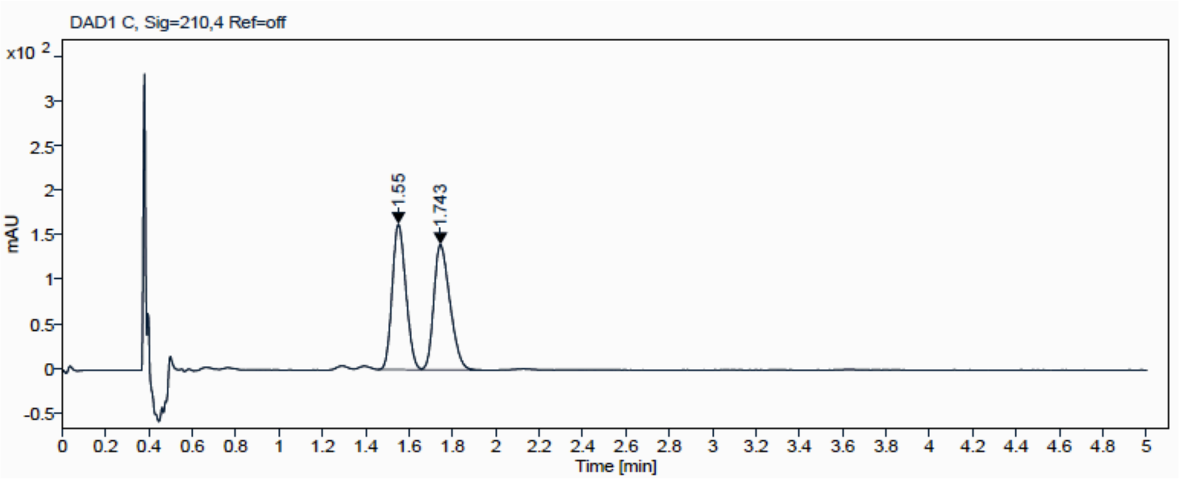

Signal: DAD1 C, Sig=210,4 Ref=off

RT [min]	Type	Width [min]	Area	Height	Area%	Name
1.550	BV	0.0685	709.2023	162.1245	49.4947	
1.743	VB	0.0795	723.6841	139.5813	50.5053	

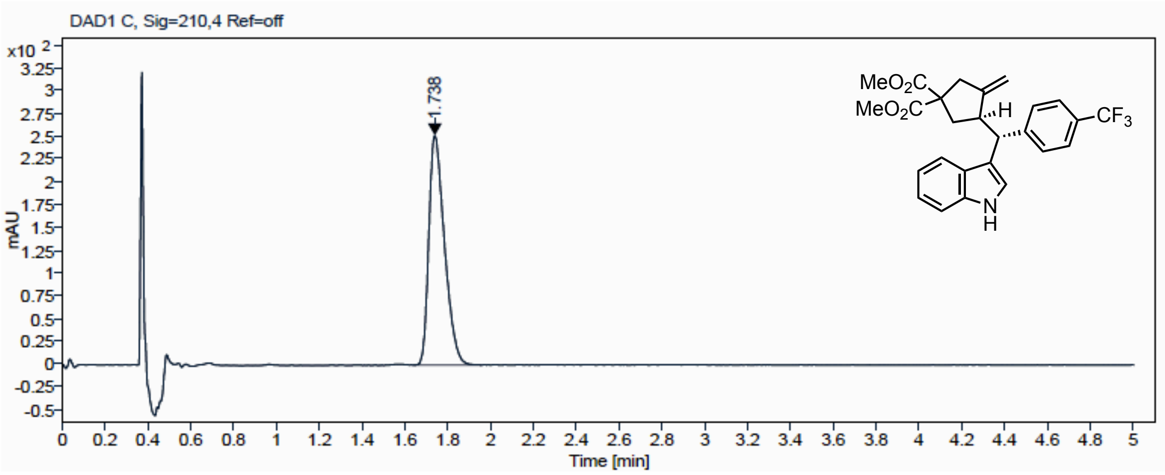

Signal: DAD1 C, Sig=210,4 Ref=off

RT [min]	Type	Width [min]	Area	Height	Area%	Name
1.738	BV R	0.0767	1278.8433	251.9598	100.0000	

Dimethyl (*R*)-3-((*R*)-(1H-indol-3-yl)(4-nitrophenyl)methyl)-4-methylenecyclopentane-1,1-dicarboxylate (2u)

SFC (IA (100 × 3 mm, 3 µm), 80:20 CO_2:*i*-PrOH, 1.2 mL/min, 35 ºC, BPR 150 bar, 210 nm): en2 (major, 100%) 3.08 min.

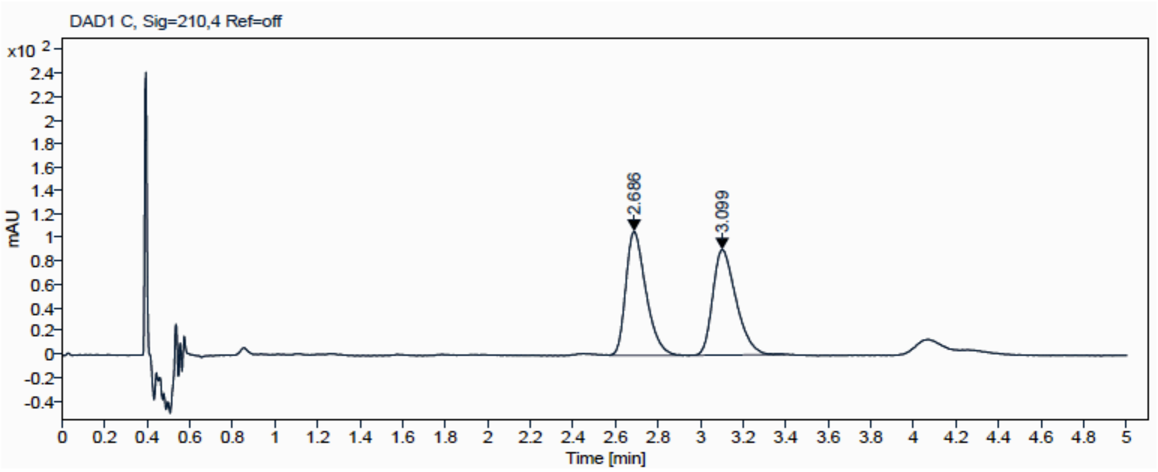

Signal: DAD1 C, Sig=210,4 Ref=off

RT [min]	Type	Width [min]	Area	Height	Area%	Name
2.686	BV R	0.1002	694.7028	105.6649	50.2708	
3.099	VV R	0.1117	687.2196	89.8917	49.7292	

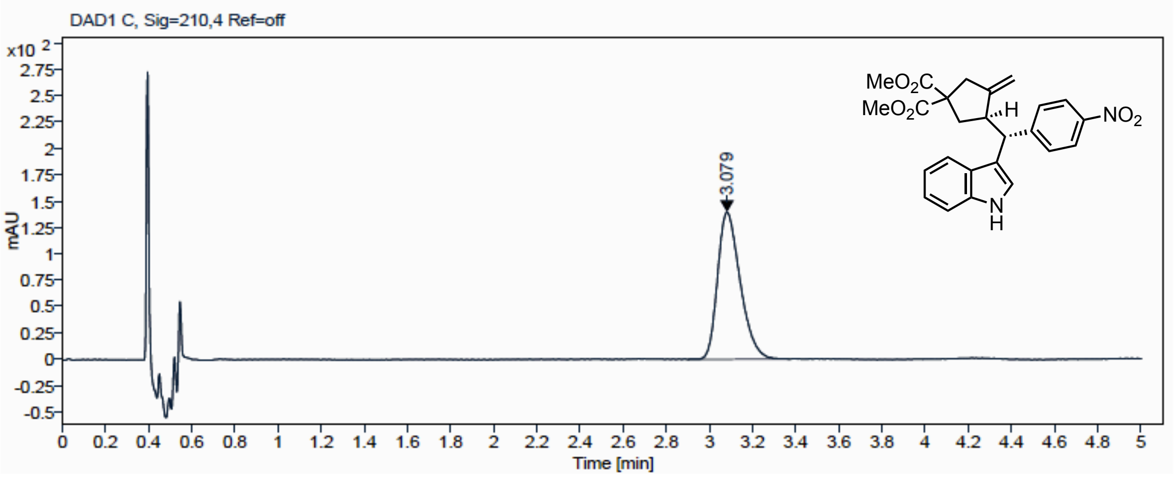

Signal: DAD1 C, Sig=210,4 Ref=off

RT [min]	Type	Width [min]	Area	Height	Area%	Name
3.079	VV R	0.1118	1019.0900	139.4089	100.0000	

Dimethyl (*R*)-3-((*R*)-(4-Chlorophenyl)(1H-indol-3-yl)methyl)-4-methylenecyclopentane-1,1-dicarboxylate (2v)

SFC (OJ (100 × 3 mm, 3 μm), 80:20 CO_2:EtOH, 1.2 mL/min, 35 ºC, BPR 150 bar, 210 nm): en1 (minor, 1%) 1.93 min, en2 (major, 99%) 2.43 min.

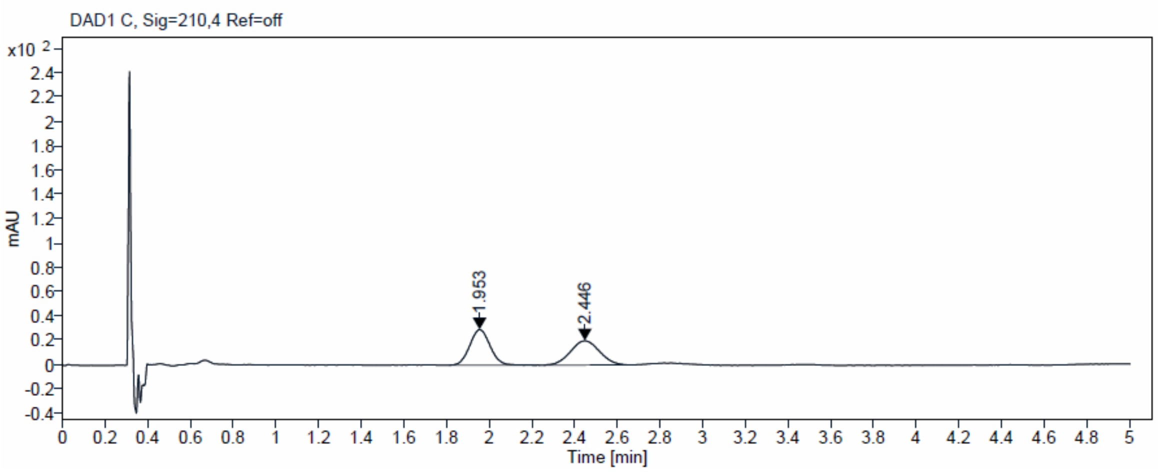

Signal: DAD1 C, Sig=210,4 Ref=off

RT [min]	Type	Width [min]	Area	Height	Area%	Name
1.953	BV R	0.1026	194.0784	29.1939	50.6672	
2.446	BB	0.1274	188.9670	19.9610	49.3328	

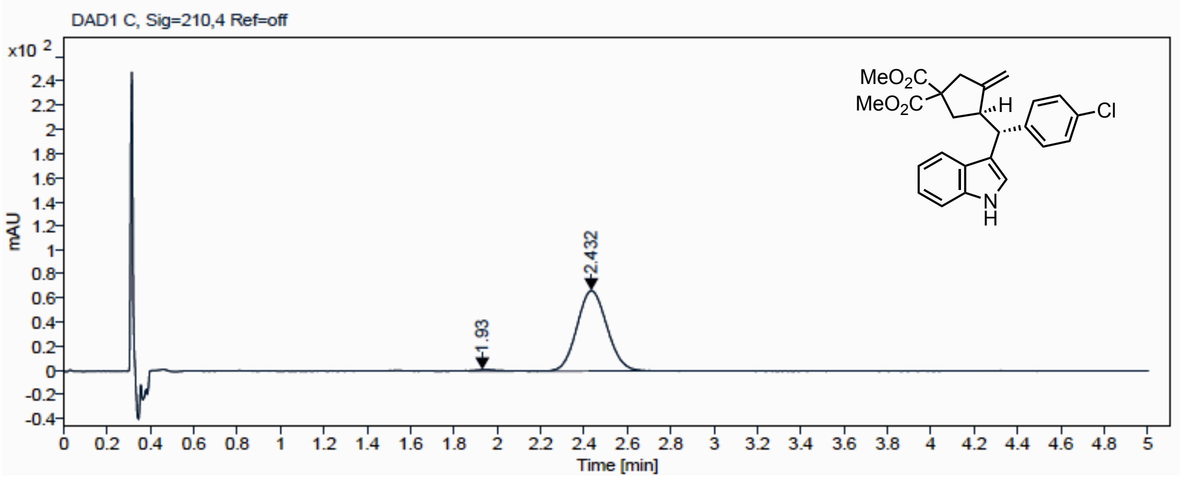

Signal: DAD1 C, Sig=210,4 Ref=off

RT [min]	Type	Width [min]	Area	Height	Area%	Name
1.930	VV	0.0874	7.8574	1.0860	1.2072	
2.432	VV R	0.1477	643.0286	66.8742	98.7928	

Dimethyl (*S*)-3-((*S*)-(1H-indol-3-yl)(4-methoxyphenyl)methyl)-4-methylenecyclopentane-1,1-dicarboxylate (2w)

SFC (IG (100 × 3 mm, 3 µm), 80:20 CO_2:*i*-PrOH, 1.2 mL/min, 35 ºC, BPR 150 bar, 210 nm): en1 (minor, 13%) 1.58 min, en2 (major, 87%) 2.04 min.

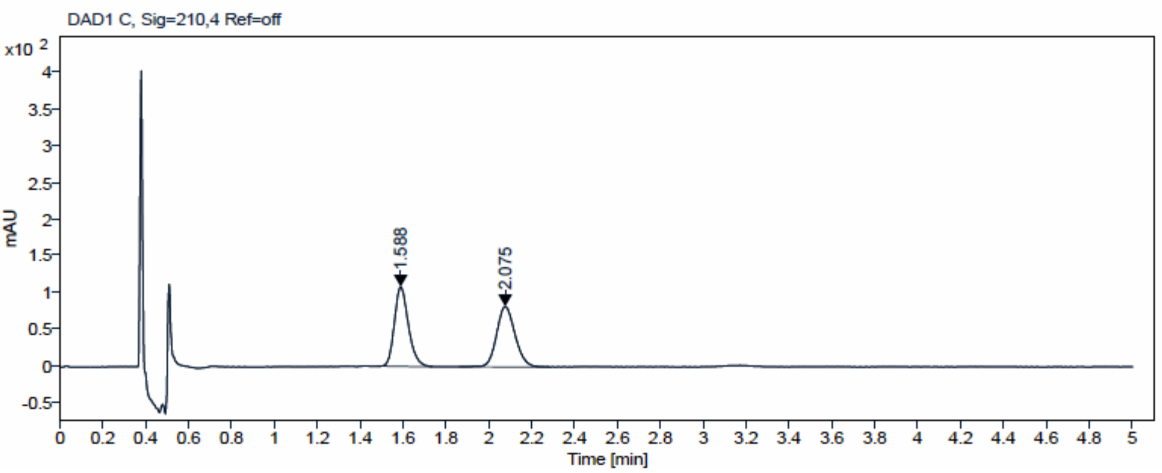

Signal: DAD1 C, Sig=210,4 Ref=off

RT [min]	Type	Width [min]	Area	Height	Area%	Name
1.588	MM	0.0758	490.0864	107.7261	50.0779	
2.075	VV R	0.0923	488.5614	81.7024	49.9221	

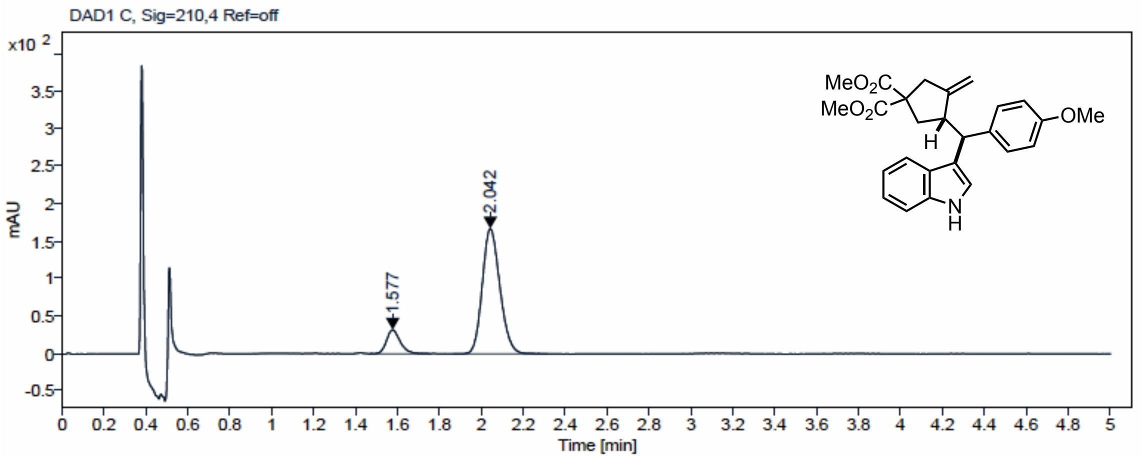

Signal: DAD1 C, Sig=210,4 Ref=off

RT [min]	Type	Width [min]	Area	Height	Area%	Name
1.577	VV R	0.0682	145.0145	31.8081	12.9016	
2.042	BV R	0.0894	978.9914	167.0685	87.0984	

Dimethyl (*R*)-3-((*R*)-(1H-indol-3-yl)(3-methoxyphenyl)methyl)-4-methylenecyclopentane-1,1-dicarboxylate (2x)

SFC (OD (100 × 3 mm, 3 µm), 85:15 CO_2:MeOH, 1.2 mL/min, 35 ºC, BPR 150 bar, 210 nm): en1 (major, 99.5%) 2.67 min, en2 (minor, 0.5%) 3.32 min.

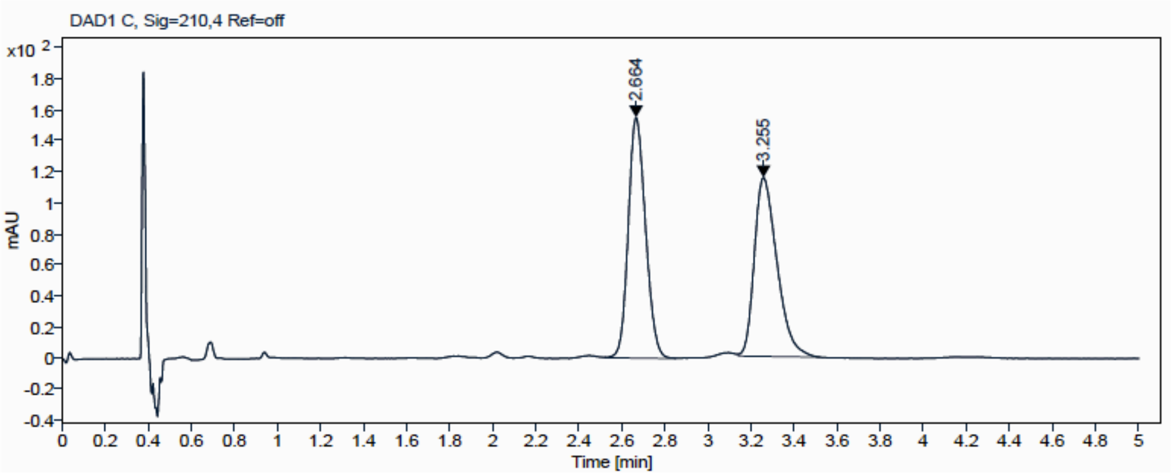

Signal: DAD1 C, Sig=210,4 Ref=off

RT [min]	Type	Width [min]	Area	Height	Area%	Name
2.664	MM	0.0930	863.1884	154.7510	50.6401	
3.255	MM	0.1220	841.3680	114.9783	49.3599	

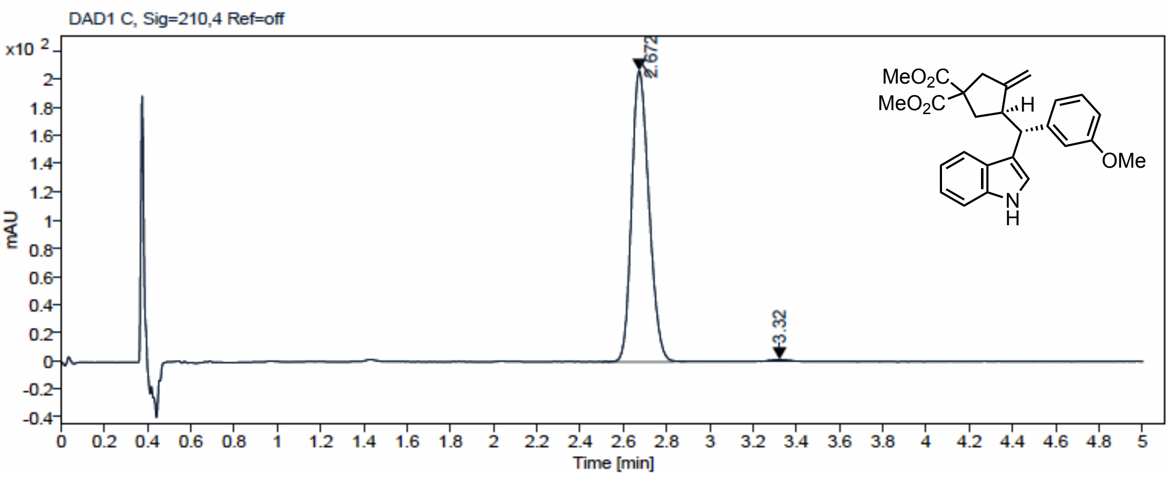

Signal: DAD1 C, Sig=210,4 Ref=off

RT [min]	Type	Width [min]	Area	Height	Area%	Name
2.672	VV R	0.0865	1149.3713	206.2618	99.5093	
3.320	MM	0.0870	5.6680	1.0857	0.4907	

Dimethyl (*R*)-3-((*R*)-(1H-indol-3-yl)(3-nitrophenyl)methyl)-4-methylenecyclopentane-1,1-dicarboxylate (2y)

SFC (IB-N (100 × 3 mm, 3 µm), 70:30 CO_2:*i*-PrOH, 1.2 mL/min, 35 ºC, BPR 150 bar, 210 nm): en1 (major, 99.5%) 1.43 min, en2 (minor, 0.5%) 1.95 min.

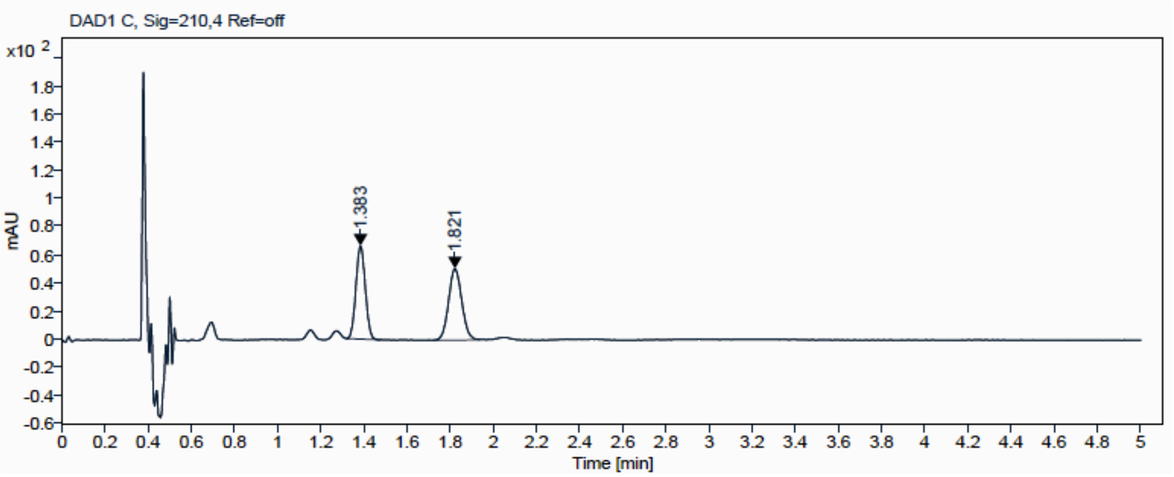

Signal: DAD1 C, Sig=210,4 Ref=off

RT [min]	Type	Width [min]	Area	Height	Area%	Name
1.383	MM	0.0505	200.1439	66.1173	48.1393	
1.821	MM	0.0709	215.6163	50.6798	51.8607	

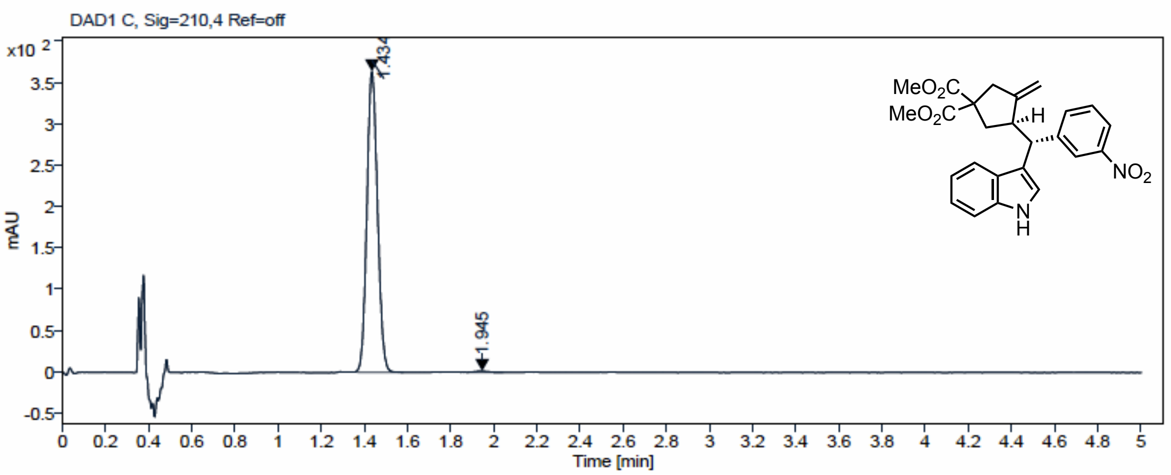

Signal: DAD1 C, Sig=210,4 Ref=off

RT [min]	Type	Width [min]	Area	Height	Area%	Name
1.434	BB	0.0529	1247.5052	364.7848	99.4696	
1.945	BB	0.0576	6.6525	1.4358	0.5304	

Dimethyl (*R*)-3-((*S*)-(1H-indol-3-yl)(2-methoxyphenyl)methyl)-4-methylenecyclopentane-1,1-dicarboxylate (2z)

SFC (IC (100 × 3 mm, 3 μm), 90:10 CO_2:EtOH, 1.2 mL/min, 35 ºC, BPR 150 bar, 210 nm): en1 (minor, 7%) 2.13 min, en2 (major, 93%) 2.55 min.

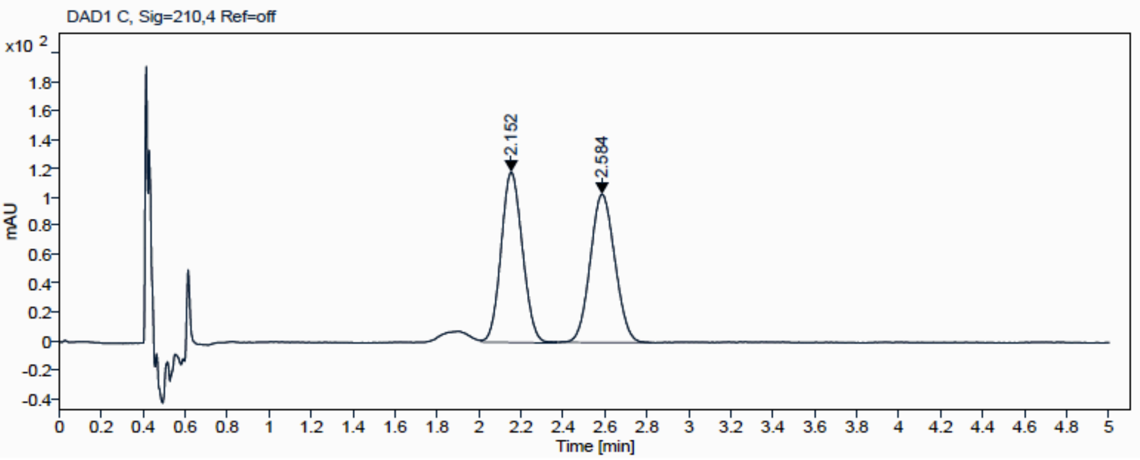

Signal: DAD1 C, Sig=210,4 Ref=off

RT [min]	Type	Width [min]	Area	Height	Area%	Name
2.152	MM	0.1181	833.1249	117.6206	49.9196	
2.584	VV R	0.1233	835.8081	102.3579	50.0804	

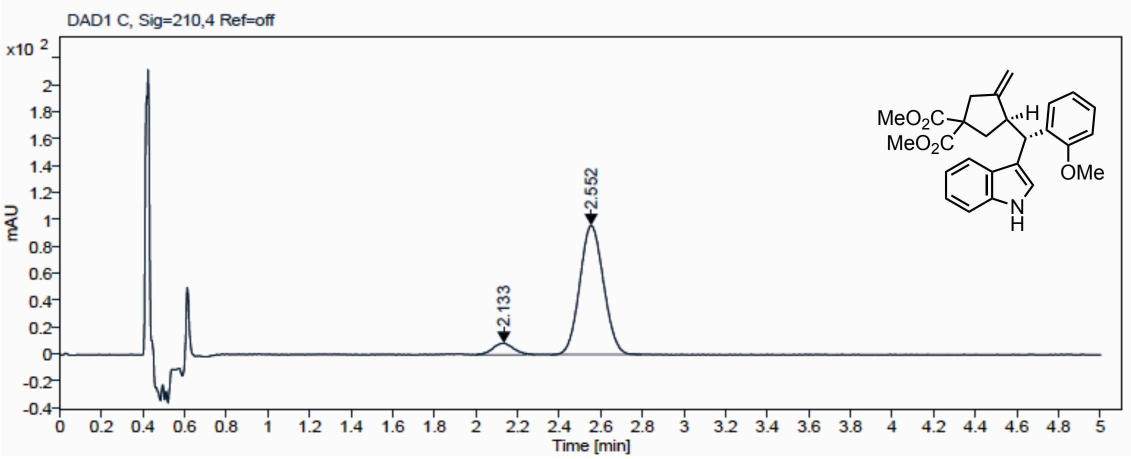

Signal: DAD1 C, Sig=210,4 Ref=off

RT [min]	Type	Width [min]	Area	Height	Area%	Name
2.133	BB	0.0887	59.2613	8.4083	7.2057	
2.552	BV R	0.1258	763.1668	95.9003	92.7943	

Dimethyl (*R*)-3-((*S*)-(2-Fluorophenyl)(1H-indol-3-yl)methyl)-4-methylenecyclopentane-1,1-dicarboxylate (2aa)

SFC (OJ (100 × 3 mm, 3 µm), 80:20 CO_2:*i*-PrOH, 1.2 mL/min, 35 ºC, BPR 150 bar, 210 nm): en1 (minor, 1.5%) 2.28 min, en2 (major, 98.5%) 3.03 min.

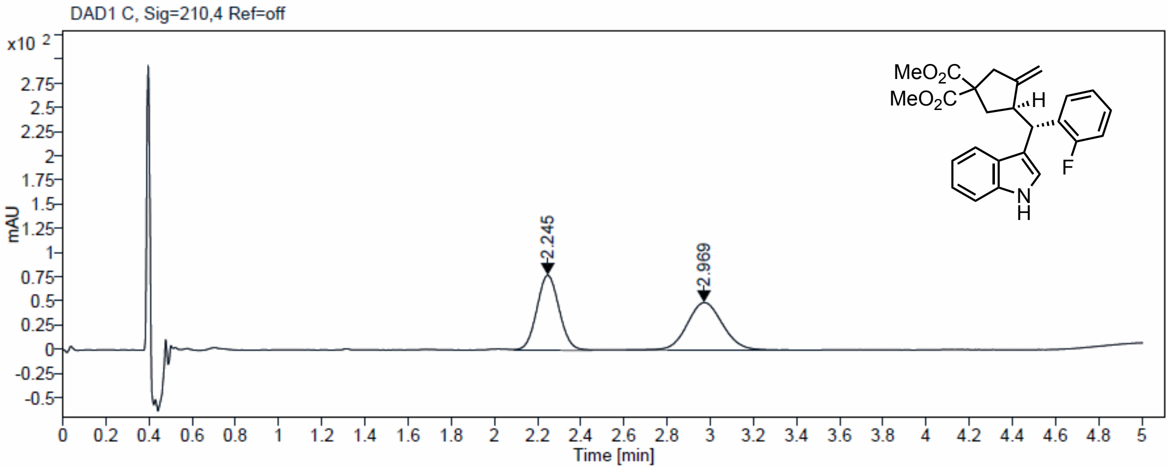

Signal: DAD1 C, Sig=210,4 Ref=off

RT [min]	Type	Width [min]	Area	Height	Area%	Name
2.245	MM	0.1200	559.6260	77.7333	49.9246	
2.969	VV R	0.1731	561.3170	49.1929	50.0754	

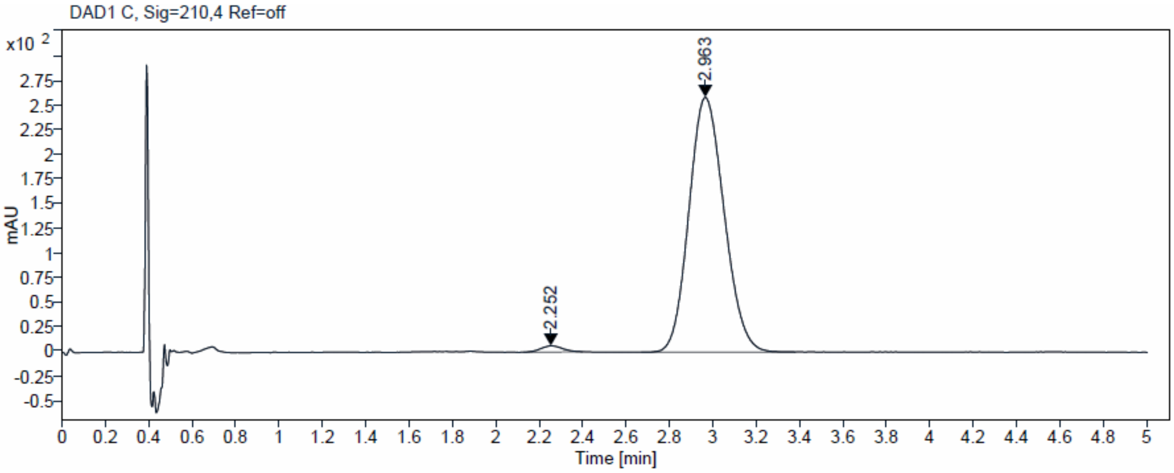

Signal: DAD1 C, Sig=210,4 Ref=off

RT [min]	Type	Width [min]	Area	Height	Area%	Name
2.252	MM	0.1186	44.2328	6.2135	1.4932	
2.963	BV R	0.1735	2917.9910	258.8774	98.5068	

Dimethyl (*R*)-3-((*R*)-(1H-indol-3-yl)(naphthalen-1-yl)methyl)-4-methylenecyclopentane-1,1-dicarboxylate (2ab)

SFC (IC (100 × 3 mm, 3 μm), 85:15 CO_2:*i*-PrOH, 1.2 mL/min, 35 ºC, BPR 150 bar, 210 nm): en1 (minor, 1.5%) 2.02 min, en2 (major, 98.5%) 2.55 min.

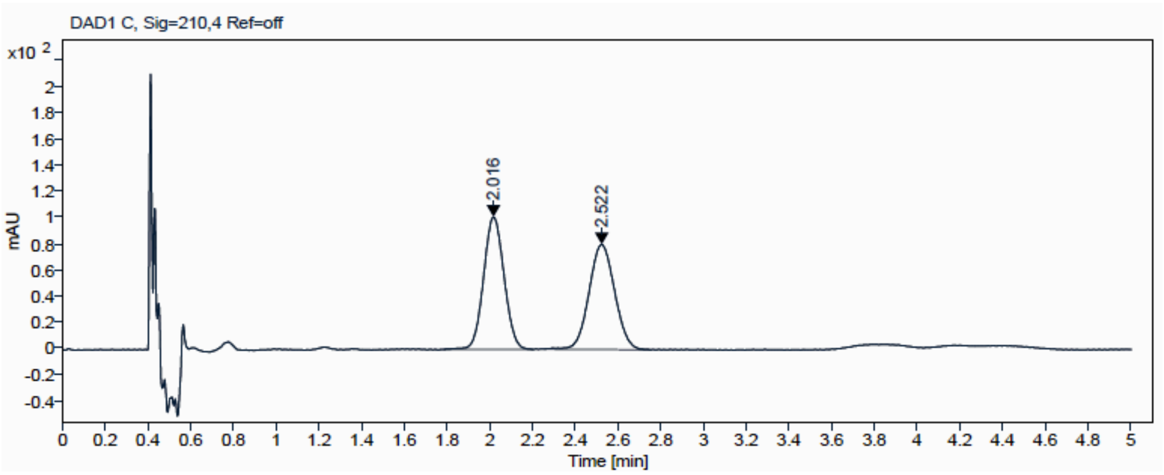

Signal: DAD1 C, Sig=210,4 Ref=off

RT [min]	Type	Width [min]	Area	Height	Area%	Name
2.016	VV R	0.1045	675.1539	100.9854	50.2886	
2.522	VV R	0.1263	667.4037	79.9897	49.7114	

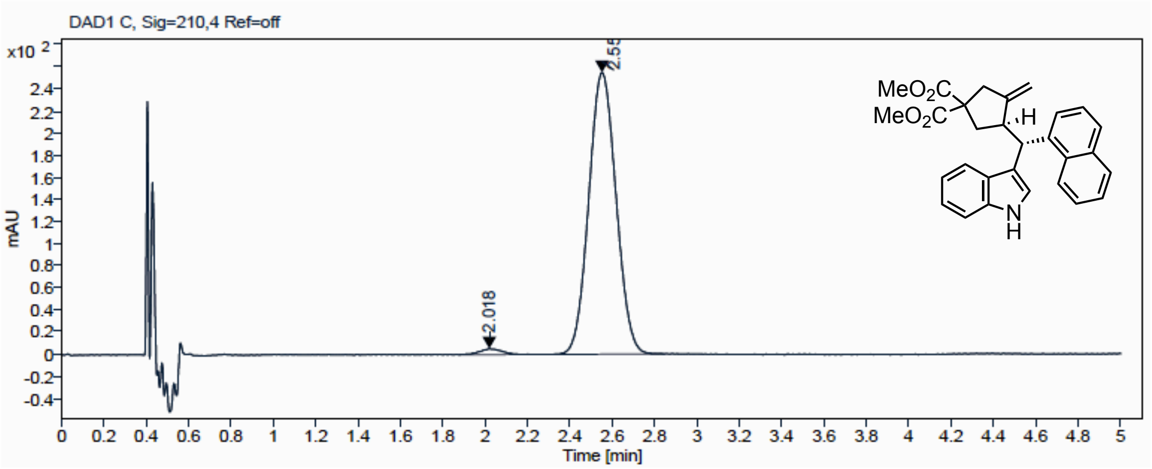

Signal: DAD1 C, Sig=210,4 Ref=off

RT [min]	Type	Width [min]	Area	Height	Area%	Name
2.018	VB R	0.0887	38.5353	5.3091	1.6618	
2.550	VV R	0.1404	2280.4165	254.9507	98.3382	

Dimethyl (*S*)-3-(2-(1H-indol-3-yl)propan-2-yl)-4-methylenecyclopentane-1,1-dicarboxylate (2ac)

SFC (OJ (100 × 3 mm, 3 µm), 80:20 CO_2:MeOH, 1.2 mL/min, 35 ºC, BPR 150 bar, 210 nm): en1 (minor, 1%) 2.35 min, en2 (minor, 99%) 3.11 min.

Note: as inseparable side product compound dimethyl 1-((1H-indol-3-yl)methyl)-6,6-dimethylbicyclo[3.1.0]hexane-3,3-dicarboxylate (2ac') was obtained, corresponding each enantiomer to peaks at 1.92 min and 3.55 min.

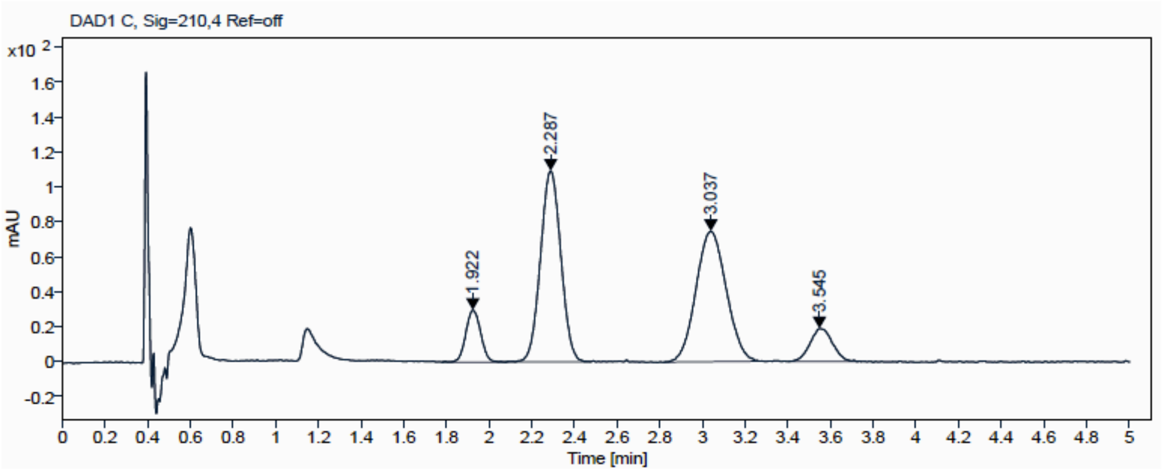

Signal: DAD1 C, Sig=210,4 Ref=off

RT [min]	Type	Width [min]	Area	Height	Area%	Name
1.922	VV R	0.0784	144.8091	29.7121	8.2780	
2.287	VV R	0.1041	727.6901	109.3770	41.5985	
3.037	VV R	0.1472	736.5493	74.6130	42.1050	
3.545	BV R	0.0917	140.2677	18.8784	8.0184	

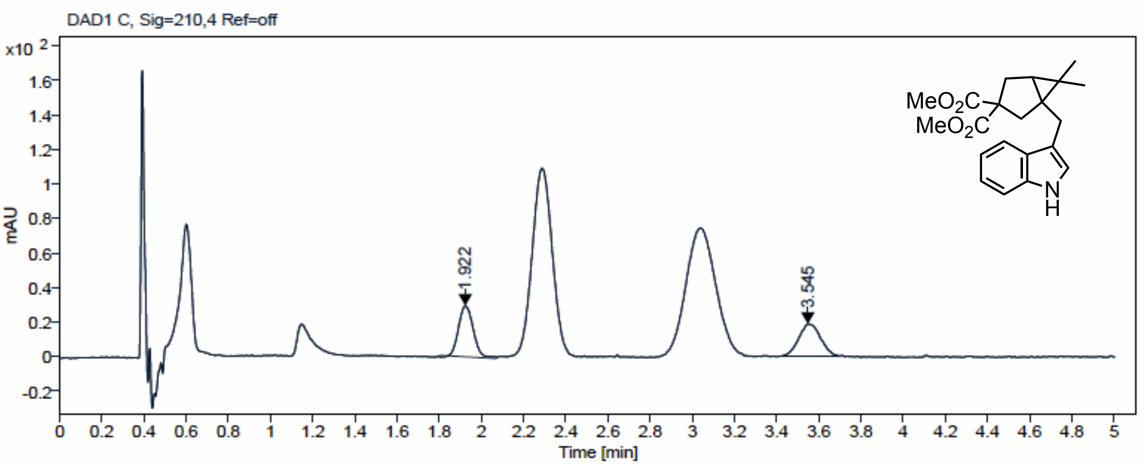

Signal: DAD1 C, Sig=210,4 Ref=off

RT [min]	Type	Width [min]	Area	Height	Area%	Name
1.922	MM	0.0812	143.5744	29.4746	51.5346	
3.545	MM	0.1208	135.0234	18.6347	48.4654	

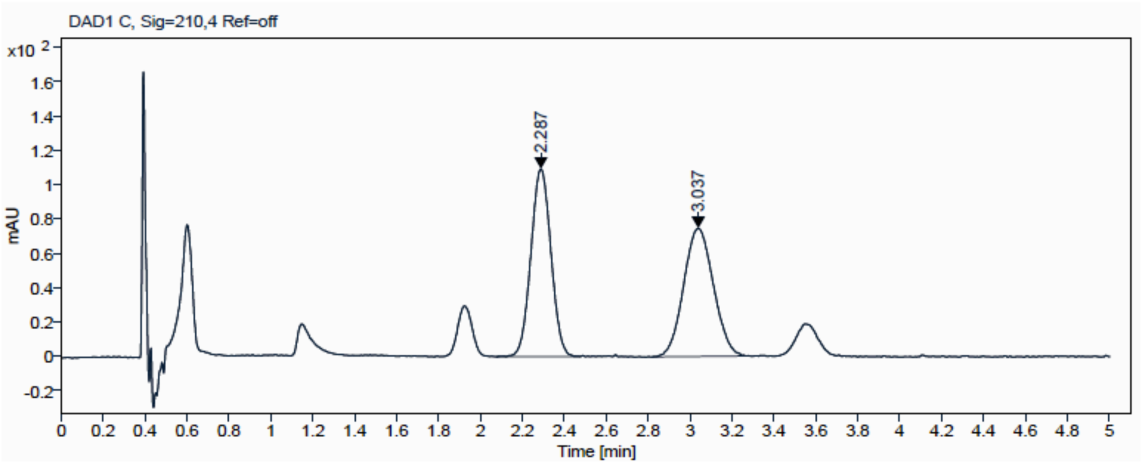

Signal: DAD1 C, Sig=210,4 Ref=off

RT [min]	Type	Width [min]	Area	Height	Area%	Name
2.287	VV R	0.1041	727.6901	109.3770	49.6975	
3.037	VV R	0.1472	736.5493	74.6130	50.3025	

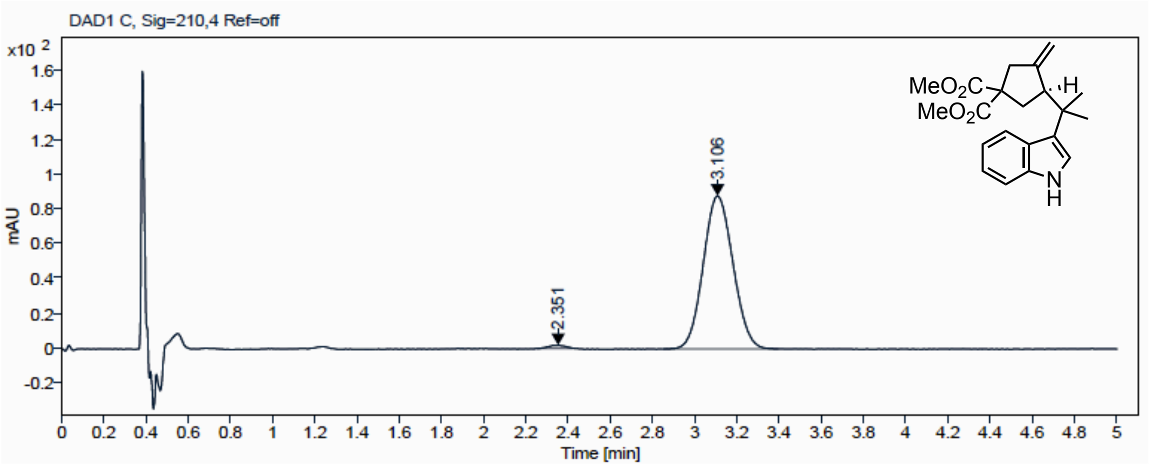

Signal: DAD1 C, Sig=210,4 Ref=off

RT [min]	Type	Width [min]	Area	Height	Area%	Name
2.351	MM	0.0967	10.7646	1.8555	1.2311	
3.106	VV R	0.1481	863.6091	87.9515	98.7689	

Dimethyl (*S*)-3-((*S*)-2-Ethoxy-6-methylhept-5-en-2-yl)-4-methylenecyclopentane-1,1-dicarboxylate (2ad)

SFC (IG-3 (150 × 4.6 mm, 3 μm), 95:5 CO_2:*i*-PrOH, 2.0 mL/min, 35 ºC, BPR 140 bar, 230 nm): en1 (major, 100%) 1.82 min.

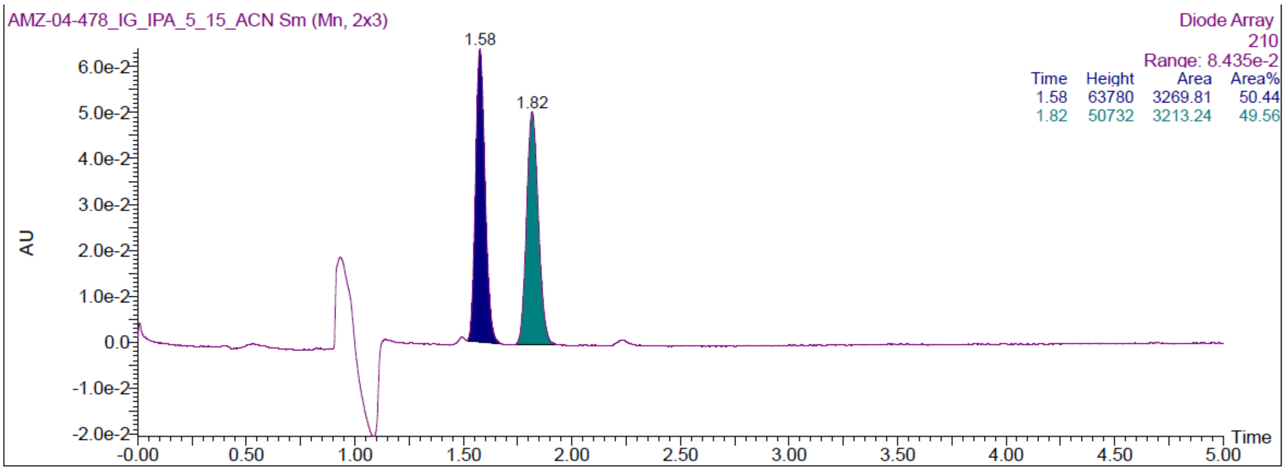

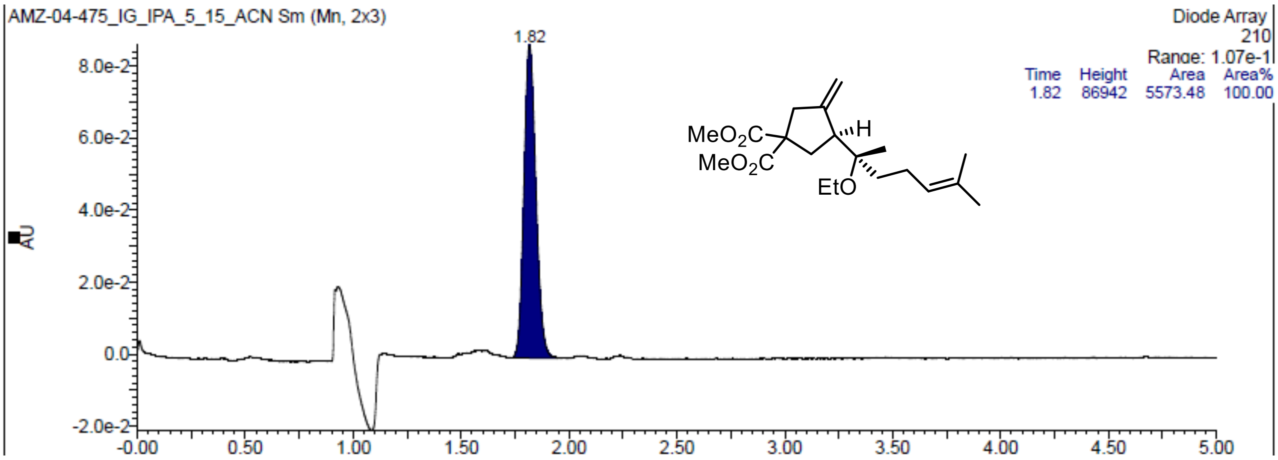

3-((*R*)-((*R*)-4,4-Bis(methoxymethyl)-2-methylenecyclopentyl)(phenyl)methyl)-1H-indole (2ae)

SFC (IB-N (100 × 3 mm, 3 µm), 80:20 CO_2:MeOH, 1.2 mL/min, 35 ºC, BPR 150 bar, 210 nm): en1 (major, 96%) 2.62 min, en2 (minor, 4%) 3.65 min.

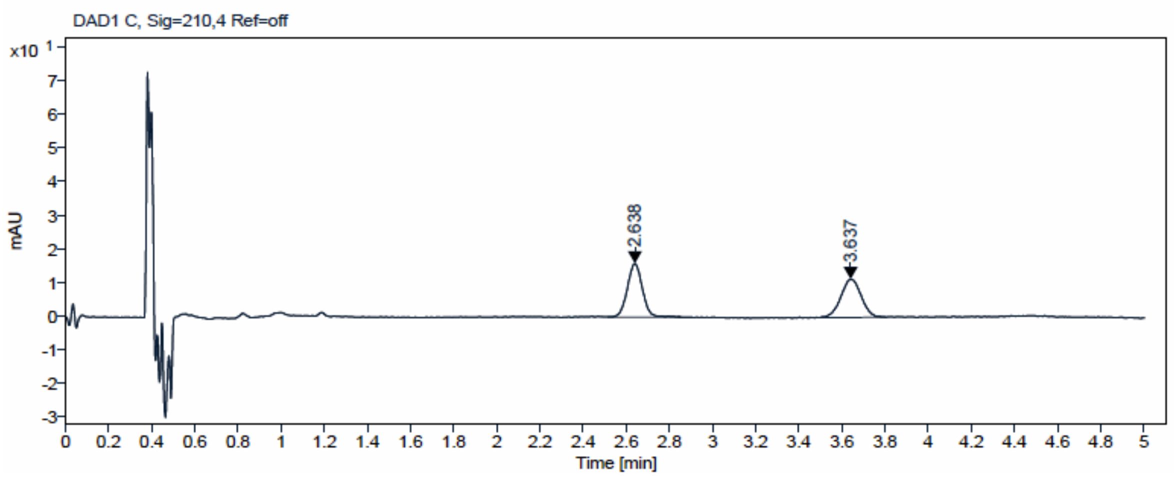

Signal: DAD1 C, Sig=210,4 Ref=off

RT [min]	Type	Width [min]	Area	Height	Area%	Name
2.638	BV R	0.0752	77.8630	15.8924	51.1341	
3.637	BV	0.0936	74.4092	11.3332	48.8659	

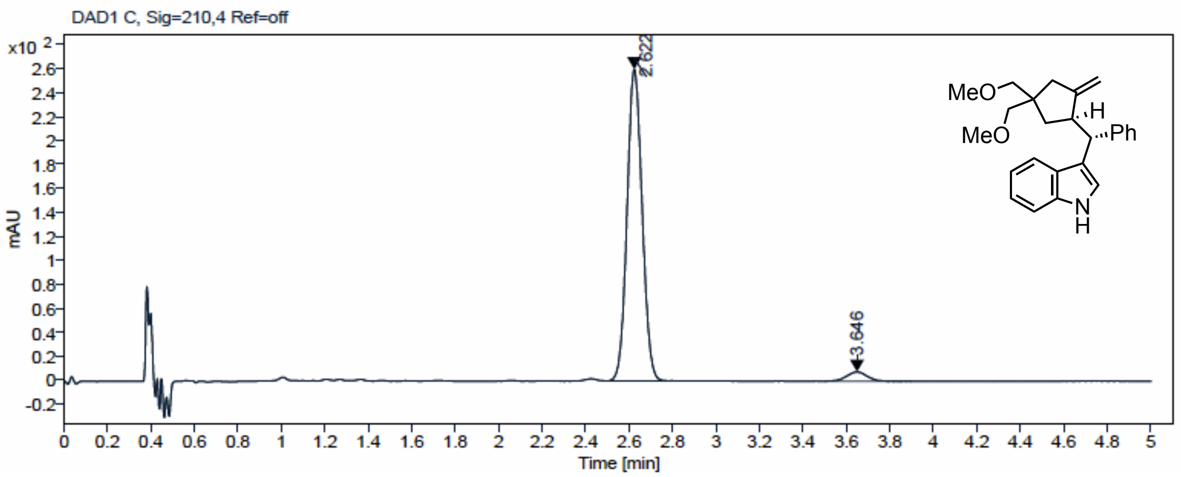

Signal: DAD1 C, Sig=210,4 Ref=off

RT [min]	Type	Width [min]	Area	Height	Area%	Name
2.622	MM	0.0805	1255.1051	259.9890	95.9814	
3.646	VV R	0.0861	52.5490	7.7955	4.0186	

Diisopropyl (*R*)-3-((*R*)-(1H-indol-3-yl)(phenyl)methyl)-4-methylenecyclopentane-1,1-dicarboxylate (2af)

SFC (IB-N (100 × 3 mm, 3 μm), 85:15 CO_2:MeOH, 1.2 mL/min, 35 ºC, BPR 150 bar, 210 nm): en1 (major, 93%) 2.14 min, en2 (minor, 7%) 2.70 min.

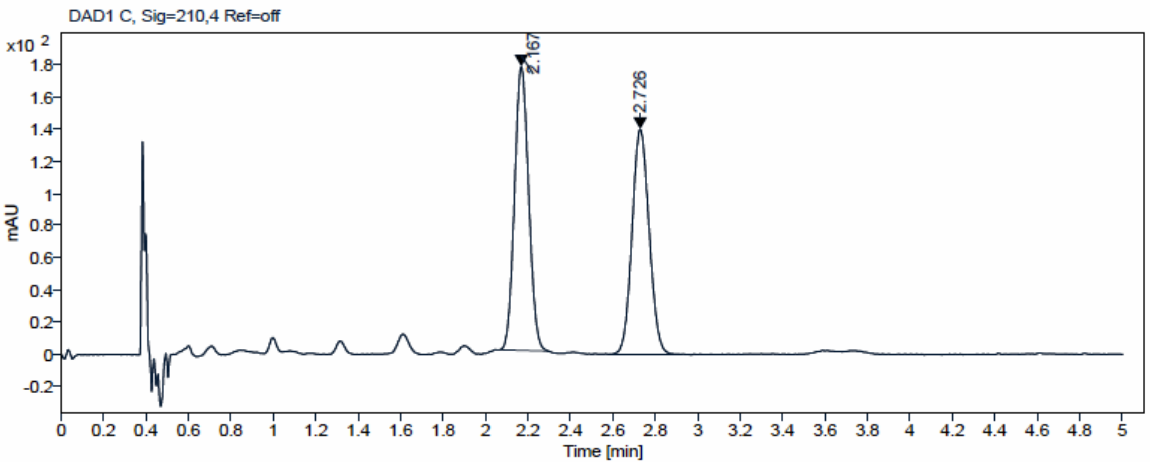

Signal: DAD1 C, Sig=210,4 Ref=off

RT [min]	Type	Width [min]	Area	Height	Area%	Name
2.167	MM	0.0772	814.2636	175.7873	51.0450	
2.726	BV R	0.0868	780.9253	139.4397	48.9550	

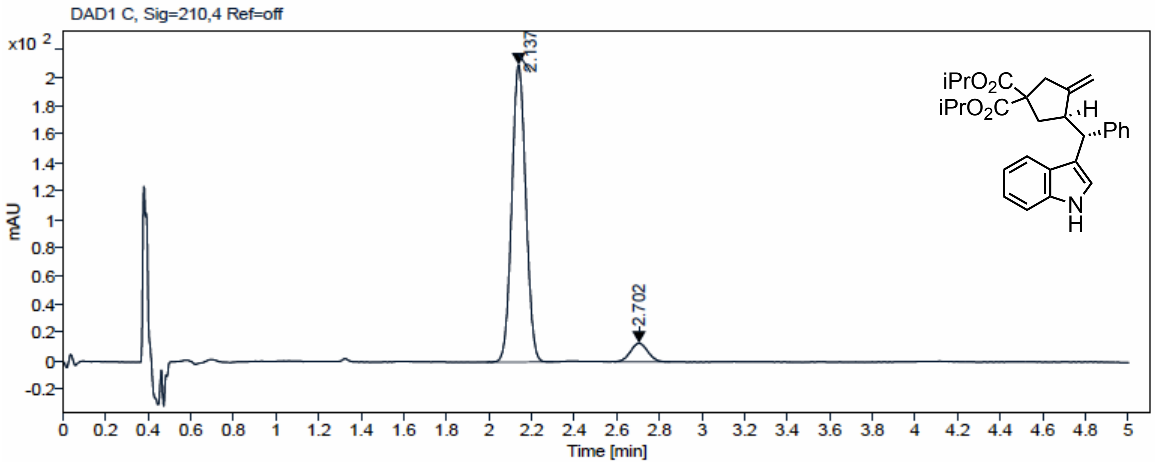

Signal: DAD1 C, Sig=210,4 Ref=off

RT [min]	Type	Width [min]	Area	Height	Area%	Name
2.137	VV R	0.0710	961.6310	209.5857	92.8125	
2.702	BV R	0.0866	74.4700	13.1452	7.1875	

3-((*R*)-((*R*)-4-Methylene-1-tosylpyrrolidin-3-yl)(phenyl)methyl)-1H-indole (2ag)

SFC (IC (100 × 3 mm, 3 μm), 80:20 CO_2:EtOH, 1.2 mL/min, 35 ºC, BPR 150 bar, 210 nm): en1 (minor, 1%) 4.66 min, en2 (major, 99%) 5.11 min.

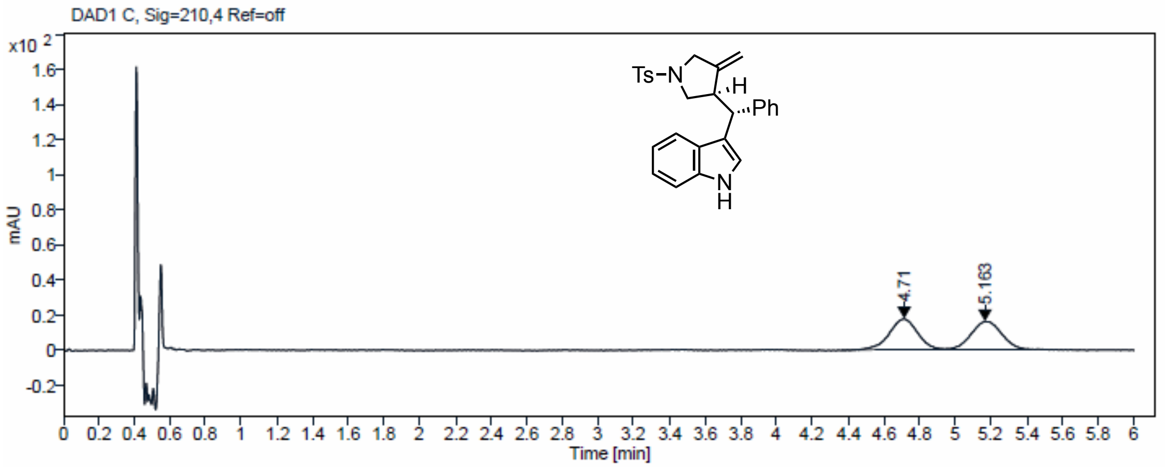

Signal: DAD1 C, Sig=210,4 Ref=off

RT [min]	Type	Width [min]	Area	Height	Area%	Name
4.710	MM	0.1826	193.2881	17.6428	50.6699	
5.163	MM	0.1964	188.1776	15.9651	49.3301	

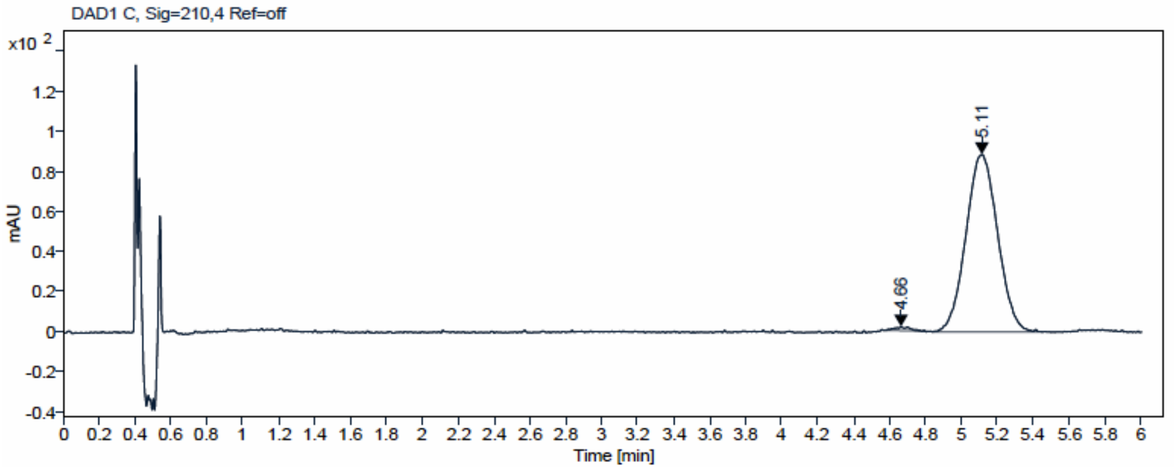

Signal: DAD1 C, Sig=210,4 Ref=off

RT [min]	Type	Width [min]	Area	Height	Area%	Name
4.660	MM	0.1027	11.9701	1.9426	1.0860	
5.110	VV R	0.1507	1090.2388	88.7993	98.9140	

(*R*)-3-((*R*)-Methoxy(phenyl)methyl)-4-methylene-1-tosylpyrrolidine (2ah)

SFC (IA (100 × 3 mm, 3 μm), 85:15 CO_2:MeOH, 1.2 mL/min, 35 ºC, BPR 150 bar, 210 nm): en1 (major, 98%) 1.27 min, en2 (minor, 2%) 2.31 min.

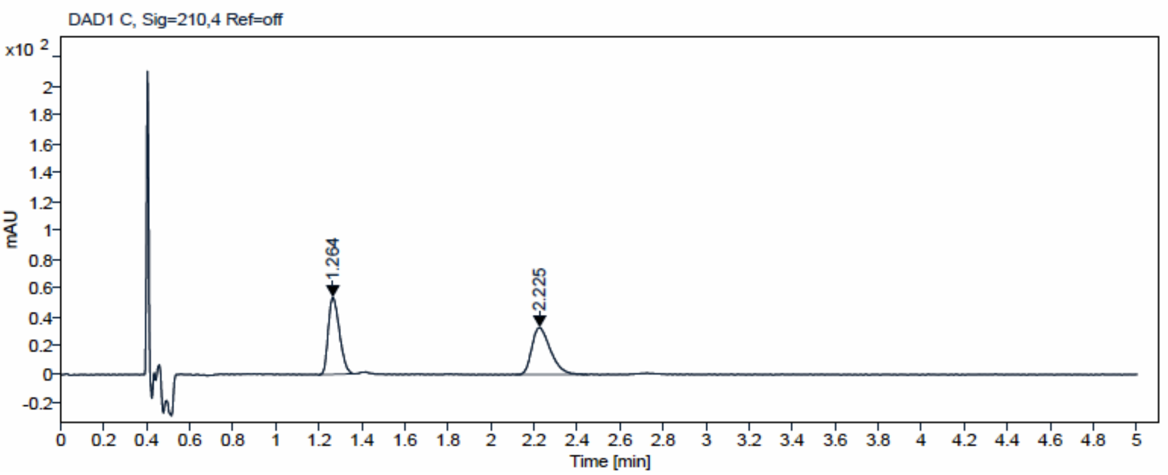

Signal: DAD1 C, Sig=210,4 Ref=off

RT [min]	Type	Width [min]	Area	Height	Area%	Name
1.264	BB	0.0577	193.2192	53.4670	49.4320	
2.225	VV R	0.0925	197.6598	32.7008	50.5680	

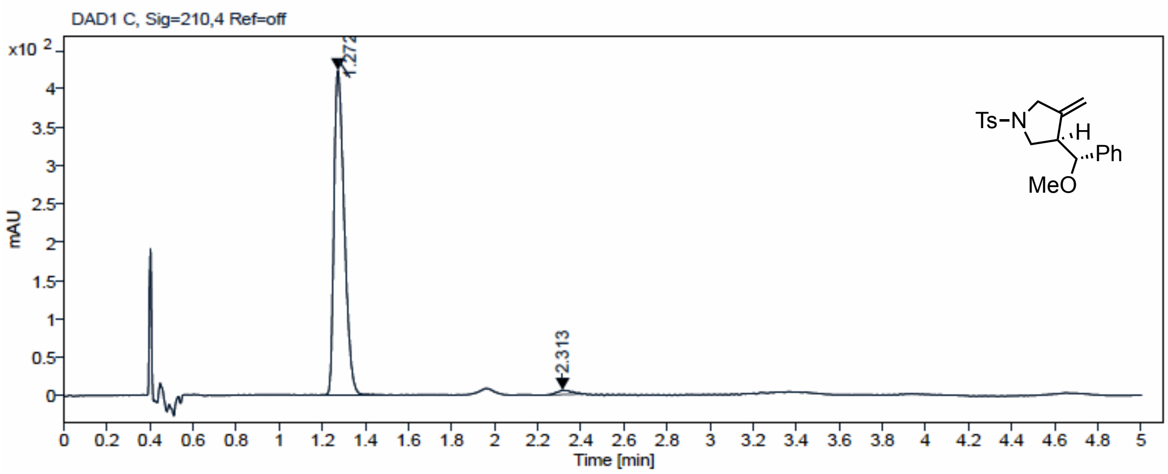

Signal: DAD1 C, Sig=210,4 Ref=off

RT [min]	Type	Width [min]	Area	Height	Area%	Name
1.272	VV R	0.0511	1402.1711	423.3980	98.1193	
2.313	MM	0.0780	26.8759	5.7420	1.8807	

(1R,5R,6S)-6-Phenyl-3-tosyl-3-azabicyclo[3.1.0]hexane-1-carbaldehyde (2ai)

SFC (OD (100 × 3 mm, 3 µm), 70:30 CO_2:EtOH, 1.2 mL/min, 35 ºC, BPR 150 bar, 210 nm): en1 (minor, 1%) 0.83 min, en2 (minor, 99%) 1.25 min.

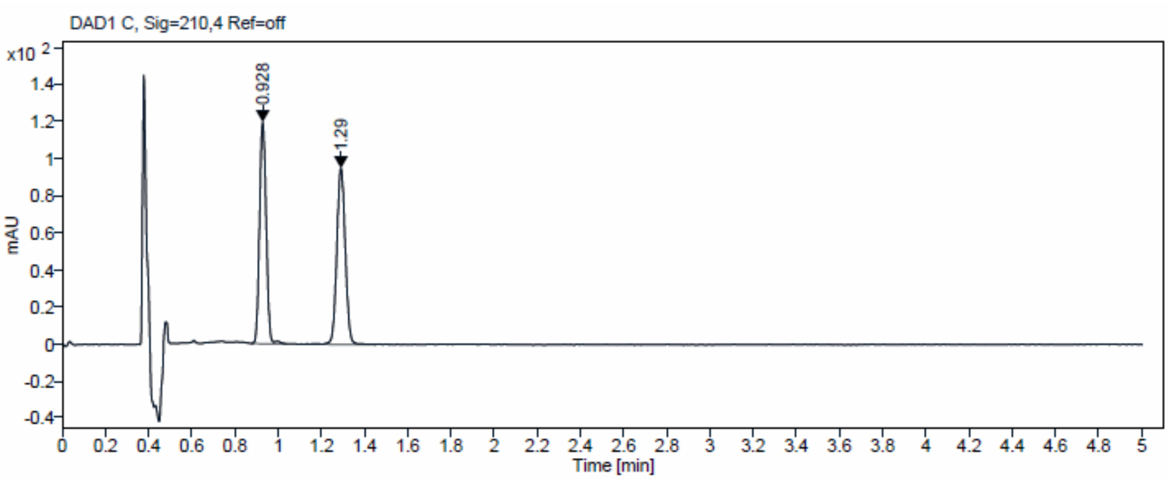

Signal: DAD1 C, Sig=210,4 Ref=off

RT [min]	Type	Width [min]	Area	Height	Area%	Name
0.928	BV R	0.0340	256.0844	119.5292	49.6198	
1.290	VV R	0.0429	260.0087	95.0093	50.3802	

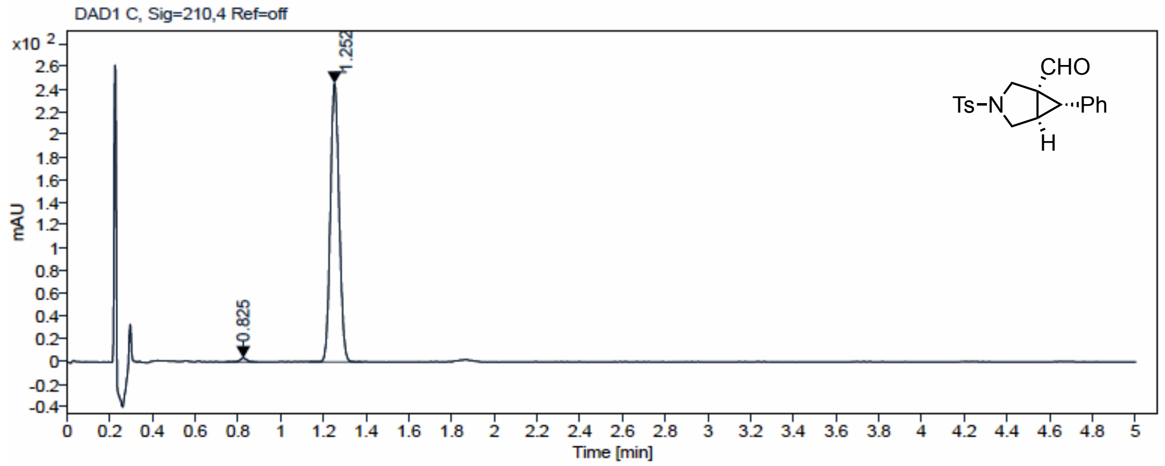

Signal: DAD1 C, Sig=210,4 Ref=off

RT [min]	Type	Width [min]	Area	Height	Area%	Name
0.825	VV R	0.0384	8.3311	3.3054	1.1735	
1.252	VV R	0.0448	701.6047	245.4536	98.8265	

Dimethyl (*1R,8aS*)-1-Phenyl-4,6,8,8a-tetrahydro-1H-cyclopenta[c]oxepine-7,7(3H)-dicarboxylate (3a)

SFC (IG (100 × 3 mm, 3 µm), 95:5 CO_2:MeOH, 1.2 mL/min, 35 ºC, BPR 150 bar, 210 nm): en1 (major, 99%) 2.60 min, en2 (minor, 1%) 3.795 min.

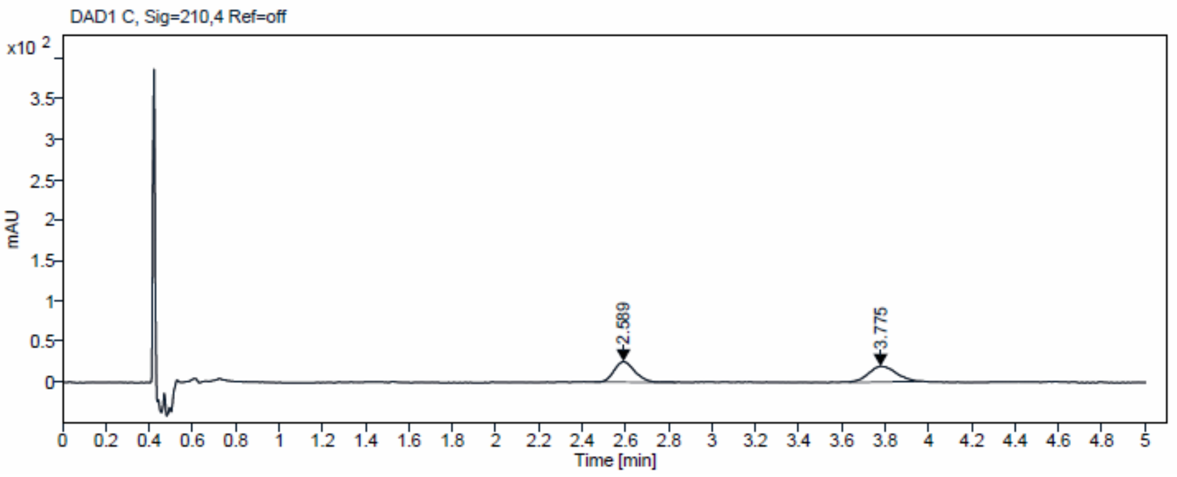

Signal: DAD1 C, Sig=210,4 Ref=off

RT [min]	Type	Width [min]	Area	Height	Area%	Name
2.589	VV R	0.0987	168.8584	25.3479	49.4895	
3.775	MM	0.1482	172.3420	19.3878	50.5105	

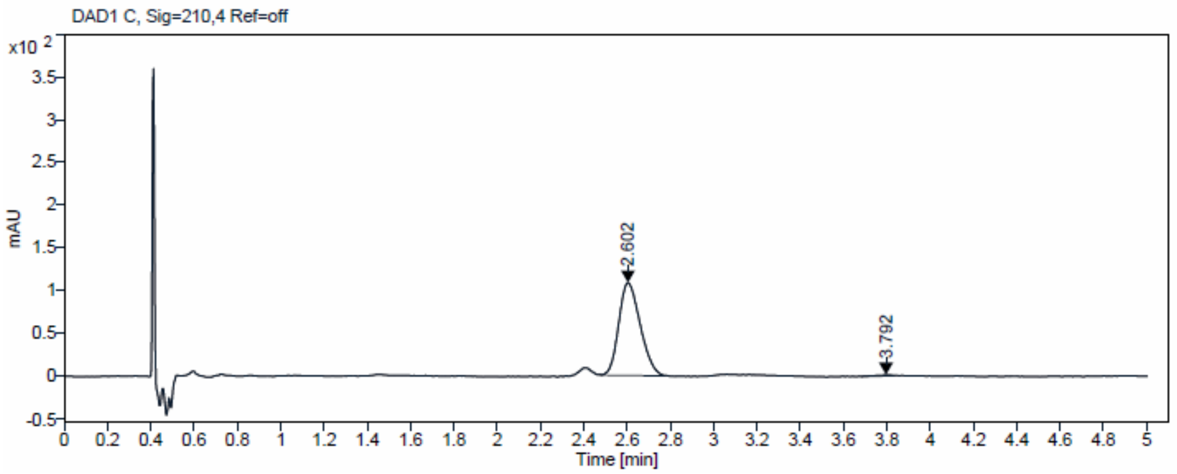

Signal: DAD1 C, Sig=210,4 Ref=off

RT [min]	Type	Width [min]	Area	Height	Area%	Name
2.602	MM	0.1146	747.2365	108.6290	99.2457	
3.792	VV R	0.0586	5.6791	1.2030	0.7543	

Dimethyl (3aS,9bR)-9b-Methyl-4-phenyl-1,3,3a,9b-tetrahydro-2H-cyclopenta[c]quinoline-2,2-dicarboxylate (3b)

SFC (IG (100 × 3 mm, 3 µm), 85:15 CO_2:EtOH, 1.2 mL/min, 35 ºC, BPR 150 bar, 210 nm): en1 (major, 98.5%) 1.05 min, en2 (minor, 1.5%) 1.30 min.

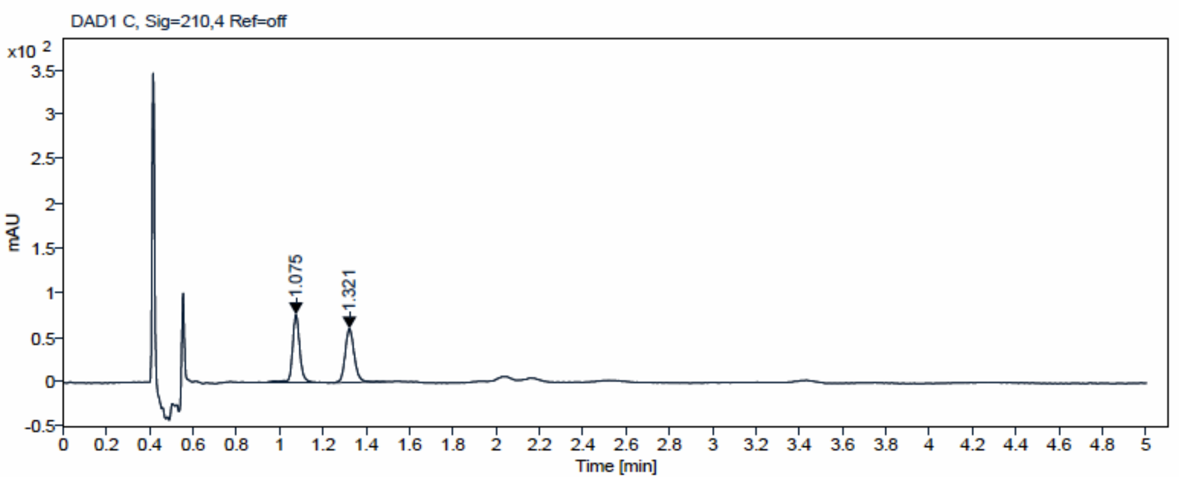

Signal: DAD1 C, Sig=210,4 Ref=off

RT [min]	Type	Width [min]	Area	Height	Area%	Name
1.075	VB R	0.0357	177.6379	76.0770	49.9686	
1.321	VV R	0.0450	177.8614	60.1610	50.0314	

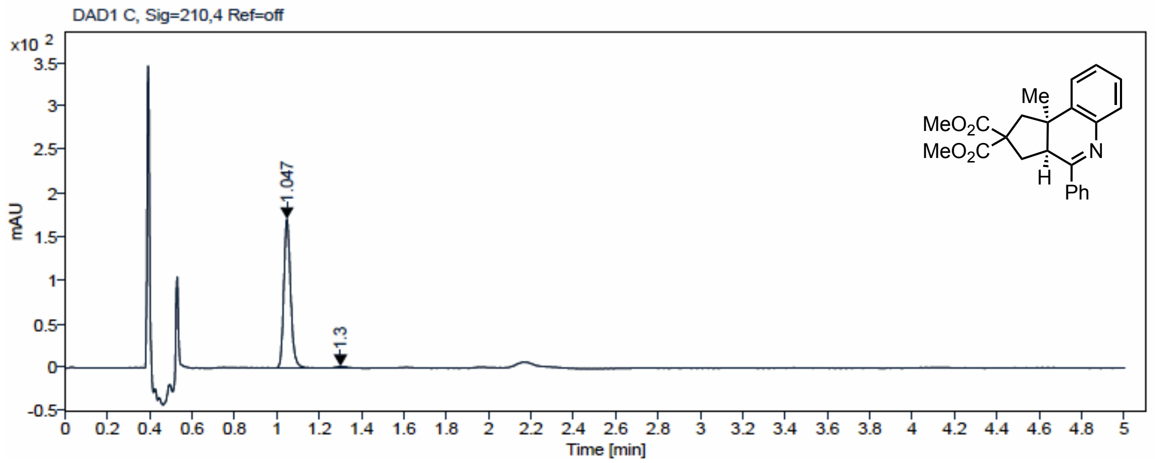

Signal: DAD1 C, Sig=210,4 Ref=off

RT [min]	Type	Width [min]	Area	Height	Area%	Name
1.047	VV R	0.0341	383.1263	171.1013	98.4035	
1.300	VV R	0.0439	6.2157	1.9115	1.5965	

(*R*)-4-((*R*)-Methoxy(phenyl)methyl)-3-methylene-1-tosylpyrrolidin-2-one (3c)

SFC (IC (100 × 3 mm, 3 μm), 80:20 CO_2:ACN, 1.2 mL/min, 35 ºC, BPR 150 bar, 210 nm): en1 (minor, 2%) 2.26 min, en2 (major, 98%) 3.30 min.

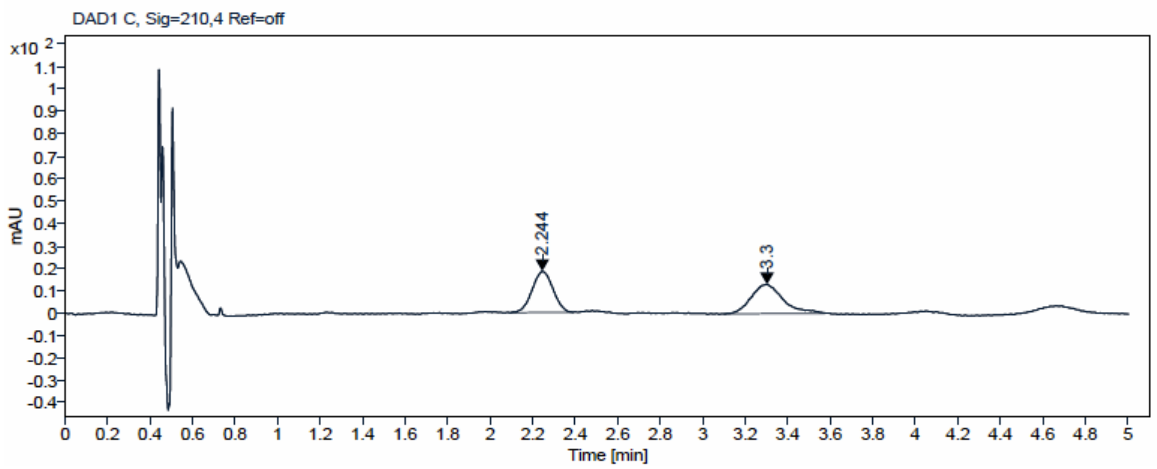

Signal: DAD1 C, Sig=210,4 Ref=off

RT [min]	Type	Width [min]	Area	Height	Area%	Name
2.244	VB R	0.0920	124.6645	18.3030	47.8694	
3.300	VV R	0.1287	135.7621	12.9069	52.1306	

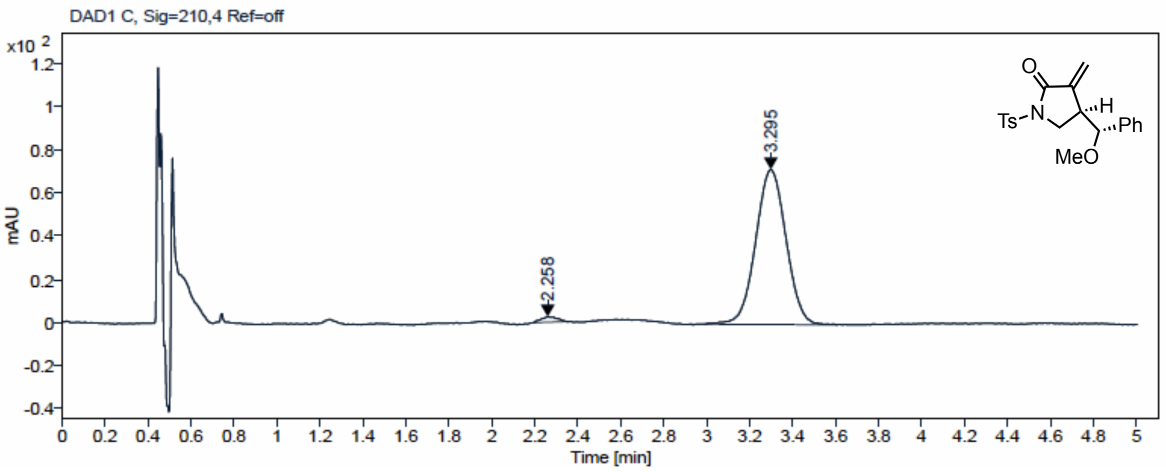

Signal: DAD1 C, Sig=210,4 Ref=off

RT [min]	Type	Width [min]	Area	Height	Area%	Name
2.258	MM	0.0873	12.7958	2.4429	1.7802	
3.295	VV R	0.1523	705.9994	71.7208	98.2198	

9. References

(1) Fulmer, G. R.; Miller, A. J. M.; Sherden, N. H.; Gottlieb, H. E.; Nudelman, A.; Stoltz, B. M.; Bercaw, J. E.; Goldberg, K. I. NMR chemical shifts of trace impurities: common laboratory solvents, organics, and gases in deuterated solvents relevant to the organometallic chemist. *Organometallics* **2010**, *29*, 2176–2179.

(2) (a) A. Franchino, À. Martí, A. M. Echavarren H-Bonded Counterion-Directed Enantioselective Au(I) Catalysi. *J. Am. Chem. Soc.* **2022**, *144*, 3497–3509. (b) A. Martí, M. Montesinos-Magraner, A. M. Echavarren, A. Franchino, European H-Bonded Counterion-Directed Catalysis: Enantioselective Gold(I)-Catalyzed Addition to 2-Alkynyl Enones as a Case Study. *J. Org. Chem.* **2022.** DOI: 10.1002/ejoc.202200518.

(3) Delpont, N.; Escofet, I.; Pérez-Galán, P.; Spiegl, D.; Raducan, M.; Bour, C.; Sinisi, R.; Echavarren, A. M. Modular chiral gold(i) phosphite complexes. *Catal. Sci. Technol.*, **2013**, *3*, 30073012.

(4) Velappan, A. B.; Charan Raja, M. R.; Datta, D.; Tsai, Y. T.; Halloum, I.; Wan, B.; Kremer, L.; Gramajo, H.; Franzblau, S. G.; Kar Mahapatra, S.; Debnath, J. Attenuation of Mycobacterium Species through Direct and Macrophage Mediated Pathway by Unsymmetrical Diaryl Urea. *Eur. J. Med. Chem.* **2017**, *125*, 825–841.

(5) Zou, H.; Supa, K.; Zhang, J. Synthesis method of urea compound, and its application in synthesizing pesticide methyldymron and thidiazuron and anticancer drug sorafenib. Patent CN109776244, May 21, 2019.

(6) Hungerbühler, E.; Naef, R.; Schnurrenberger, P.; Weidmann, B.; Züger, M.; Seebach, D. Titanate-Mediated Transesterifications with Functionalized Substrates. *Synthesis,* **1982**, *2*, 138–141

(7) Chao, C. M.; Vitale, M. R.; Toullec, P. Y.; Genêt, J. P.; Michelet, V. Asymmetric Gold-Catalyzed Hydroarylation/Cyclization Reactions. *Chem. - A Eur. J.* **2009**, *15* (6), 1319–1323.

8 Wang, G.; Wang, Y.; Li, Z.; Li, H.; Yu, M.; Pang, M.; Zhao, X. Gold-Catalyzed Cyclization/Hydroboration of 1,6-Enynes: Synthesis of Bicyclo[3.1.0]hexane Boranes *Org. Lett.* **2022**, *24* (51), 9425–9430.

(9) Zhao, S.; Zang, Z.-L.; Li, S.; Wen, X.; Wang, C.; Guo, J.; He, Y. Iridium-Catalyzed Cycloisomerization of *N*-Tethered 1,7-Enynes: Construction of an Azabicyclo[5.1.0]octene System *J. Org. Chem.* **2020**, *85*, 14, 9321–9330.

(10) Nieto-Oberhuber, C.; Muñoz, M. P.; Lopez, S.; Jimenez-Nuñez, E.; Nevado, C.; Herrero-Gomez, E.; Raducan, M.; Echavarren, A. M. Gold(I)-Catalyzed Cyclizations of 1,6-Enynes: Alkoxycyclizations and exo/endo Skeletal Rearrangements *Chem. Eur. J.* **2006**, *12*, 1677–1693.

(11) Toullec, P. Y.; Genin, E.; Leseurre, L.; Genêt, J.-P.; Michelet, V. Room-Temperature AuI-Catalyzed C-C Bond Formation through a Tandem Friedel–Crafts-Type Addition/Carbocyclization Reaction. *Angew. Chemie* **2006**, *118* (44), 7587–7590.

(12) Leseurre, L.; Chao, C. M.; Seki, T.; Genin, E.; Toullec, P. Y.; Genêt, J. P.; Michelet, V. Synthesis of Functionalized Carbo- and Heterocycles via Gold-Catalyzed Cycloisomerization Reactions of Enynes. *Tetrahedron* **2009**, *65* (9), 1911–1918.

(13) Miller, R.; Carreras, J.; Muratore, M. E.; Gaydou, M.; Camponovo, F.; Echavarren, A. M. Broad Scope Aminocyclization of Enynes with Cationic JohnPhos–Gold (I) Complex as the Catalyst. *J. Org. Chem.* **2016**, *81*, 1839–1849.

(14) Charruault, L.; Michelet, V.; Taras, R.; Gladiali, S.; Genêt, J. P. Functionalized carbo- and heterocycles via Pt-catalyzed asymmetric alkoxycyclization of 1,6-enynes. *Chem. Commun.* **2004**, *4*, 850–851.

(15) Méndez, M.; Muñoz, M. P.; Nevado, C.; Cárdenas, D. J.; Echavarren, A. M. Cyclizations of Enynes Catalyzed by PtCl2 or Other Transition Metal Chlorides: Divergent Reaction Pathways. *J. Am. Chem. Soc.* **2001**, *123* (43), 10511–10520.

(16) Genin, E.; Leseurre, L.; Toullec, P. Y.; Genêt, J. P.; Michelet, V. Gold-Catalyzed Hydroxy- and Alkoxycyclization of Functionalized Enynes. *Synlett* **2007**, *11*, 1780–1784.

(17) Amijs, C. H. M.; López-Carrillo, V.; Raducan, M.; Pérez-Galán, P.; Ferrer, C.; Echavarren, A. M. Gold(I)-Catalyzed Intermolecular Addition of Carbon Nucleophiles to 1,5- and 1,6-Enynes. *J. Org. Chem.* **2008**, *73* (19), 7721–7730.

(18) Amijs, C. H. M.; Ferrer, C.; Echavarren, A. M. Gold(I)-Catalysed Arylation of 1,6-Enynes: Different Site Reactivity of Cyclopropyl Gold Carbenes. *Chem. Commun.* **2007**, *7*, 698–700.

(19) Martín-Torres, I.; Ogalla, G.; Yang, J. M.; Rinaldi, A.; Echavarren, A. M. Enantioselective Alkoxycyclization of 1,6-Enynes with Gold(I)-Cavitands: Total Synthesis of Mafaicheenamine C. *Angew. Chemie. Int. Ed.* **2021**, *60* (17), 9339–9344.

(20) Witham, C. A.; Mauleo, P.; Shapiro, N. D.; Sherry, B. D.; Toste, F. D. Gold (I) -Catalyzed Oxidative Rearrangements. *J. Am. Chem. Soc.* **2007**, *1*, 5838–5839.

(21) Chao, C.M.; Toullec, P.Y.; Michelet, V. Synthesis of polycyclic heterocycles via sequential Au-catalyzed cycloisomerization and Ru-catalyzed metathesis reactions. *Tetrahedron Lett.*, **2009**, *50*, 3719–3722.

(22) Wu, B.; Mosher, H. S. Configuration of some para-substituted benzhydrols. *J. Org. Chem.*, **1986**, *51*, 1904–1906.

(23) Trost, B. M.; Belletire, J. L.; Godelski, S.; McDougal, P. G.; Balkovec, J. M. On the use of the O-methylmandelate ester for establishment of absolute configuration of secondary alcohols *J. Org. Chem.*, **1986**, *51*, 2370–2374.

(24) Ohtani, I.; Kusumi, T.; Kashman, Y.; Kakisawa, H. High-field FT NMR application of Mosher's method. The absolute configurations of marine terpenoids. *J. Am. Chem. Soc.*, **1991**, *113*, 4092–4096.

(25) a) Michelet, V.; Charruault, L.; Gladiali, S.; Genêt, J. P. Alkoxy- and Hydroxycyclization of Enynes Catalyzed by Pd(II) and Pt(II) Catalysts. *Pure Appl. Chem.* **2006**, *78*, 397–407. b) Chao, C. M.; Genin, E.; Toullec, P. Y.; Genêt, J. P.; Michelet, V. Towards Asymmetric Au-Catalyzed Hydroxy- and Alkoxycyclization of 1,6-Enynes. *J. Organomet. Chem.* **2009**, *694*, 538–545. c) Matsumoto, Y.; Selim, K. B.; Nakanishi, H.; Yamada, K. ichi; Yamamoto, Y.; Tomioka, K. Chiral Carbene Approach to Gold-Catalyzed Asymmetric Cyclization of 1,6-Enynes. *Tetrahedron Lett.* **2010**, *51*, 404–406. d) Pradal, A.; Chao, C. M.; Vitale, M. R.; Toullec, P. Y.; Michelet, V. Asymmetric Au-Catalyzed Domino Cyclization/nucleophile Addition Reactions of Enynes in the Presence of Water, Methanol and Electron-Rich Aromatic Derivatives. *Tetrahedron* **2011**, *67*, 4371–4377. e) Wang, W.; Yang, J.; Wang, F.; Shi, M. Axially Chiral N-Heterocyclic Carbene gold(I) Complex Catalyzed Asymmetric Cycloisomerization of 1,6-Enynes. *Organometallics* **2011**, *30*, 3859–3869. f) Tugny, C.; Del Rio, N.; Koohgard, M.; Vanthuyne, N.; Lesage, D.; Bijouard, K.; Zhang, P.; Meijide Suárez, J.; Roland, S.; Derat, E.; Bistri-Aslanoff,

O.; Sollogoub, M.; Fensterbank, L.; Mouriès-Mansuy, V. β-Cyclodextrin-NHC-Gold(I) Complex (β-ICyD)AuCl: A Chiral Nanoreactor for Enantioselective and Substrate-Selective Alkoxycyclization Reactions. ACS Catal. **2020**, *10*, 5964–5972.

(26) Gaussian 09, Revision D.01, M. J. Frisch, G. W. Trucks, H. B. Schlegel, G. E. Scuseria, M. A. Robb, J. R. Cheeseman, G. Scalmani, V. Barone, B. Mennucci, G. A. Petersson, H. Nakatsuji, M. Caricato, X. Li, H. P. Hratchian, A. F. Izmaylov, J. Bloino, G. Zheng, J. L. Sonnenberg, M. Hada, M. Ehara, K. Toyota, R. Fukuda, J. Hasegawa, M. Ishida, T. Nakajima, Y. Honda, O. Kitao, H. Nakai, T. Vreven, J. A. Montgomery, Jr., J. E. Peralta, F. Ogliaro, M. Bearpark, J. J. Heyd, E. Brothers, K. N. Kudin, V. N. Staroverov, T. Keith, R. Kobayashi, J. Normand, K. Raghavachari, A. Rendell, J. C. Burant, S. S. Iyengar, J. Tomasi, M. Cossi, N. Rega, J. M. Millam, M. Klene, J. E. Knox, J. B. Cross, V. Bakken, C. Adamo, J. Jaramillo, R. Gomperts, R. E. Stratmann, O. Yazyev, A. J. Austin, R. Cammi, C. Pomelli, J. W. Ochterski, R. L. Martin, K. Morokuma, V. G. Zakrzewski, G. A. Voth, P. Salvador, J. J. Dannenberg, S. Dapprich, A. D. Daniels, O. Farkas, J. B. Foresman, J. V. Ortiz, J. Cioslowski, D. J. Fox, Gaussian, Inc., Wallingford CT, 2013.

(27) (a) A. D. Becke, Density-functional thermochemistry. III. The role of exact exchange. *J. Chem. Phys.* **1993**, *98*, 5648–5652. (b) C. Lee, W. Yang, R. G. Parr, Development of the Colle-Salvetti correlation-energy formula into a functional of the electron density. *Phys. Rev. B.* **1988**, *37*, 785–789. (c) S. H. Vosko, L. Wilk, M. Nusair, Accurate spin-dependent electron liquid correlation energies for local spin density calculations: a critical analysis. *Can. J. Phys.* **1980**, *58*, 1200–1211. (d) P. J. Stephens, F. J. Devlin, C. F. Chabalowsky, M. J. Frisch, Ab Initio Calculation of Vibrational Absorption and Circular Dichroism Spectra Using Density Functional Force Fields. *J. Phys. Chem.* **1994**, *98*, 11623–11627.

(28) Grimme, S.; Antony, J.; Ehrlich, S.; Krieg, H. A consistent and accurate ab initio parametrization of density functional dispersion correction (DFT-D) for the 94 elements H-Pu. *J. Chem. Phys.* **2010**, *132*, 154104-1–19.

(29) Hehre, W. J.; Ditchfield, R.; Pople, Self-Consistent Molecular Orbital Methods. XII. Further Extensions of Gaussian—Type Basis Sets for Use in Molecular Orbital Studies of Organic Molecules. J. A. *J. Chem. Phys.* **1972**, *56*, 2257–2261.

(30) Andrae, D.; Häussermann, U.; Dolg, M.; Stoll, H.; Preuss, H. Energy-adjusted ab initio pseudopotentials for the second and third row transition elements. *Theor. Chim. Acta* **1990**, 77, 123–141.

(31) E. Cancès, B. Mennucci, J. Tomasi, *J. Chem. Phys.* **1997**, *107*, 3032−3041.

(32) Álvarez-Moreno, M.; De Graaf, C.; Lopez, N.; Maseras, F.; Poblet, J.M.; Bo, C. Managing the Computational Chemistry Big Data Problem: The ioChem-BD Platform. *J. Chem. Inf. Model.* **2015**, *55*, 95−103.

(33) Zuccarello, G.; Mayans, J. G.; Escofet, I.; Scharnagel, D.; Kirillova, M. S.; Pérez-Jimeno, A. H.; Calleja, P.; Boothe, J. R.; Echavarren, A. M. Enantioselective Folding of Enynes by Gold(I) Catalysts with a Remote C2-Chiral Element. *J. Am. Chem. Soc.* **2019**, *141* (30), 11858–11863.

(34) Escofet, I.; Armengol-Relats, H.; Bruss, H.; Besora, M.; Echavarren, A. M. On the Structure of Intermediates in Enyne Gold(I)-Catalyzed Cyclizations: Formation of Trans-Fused Bicyclo[5.1.0]Octanes as a Case Study. *Chem. - A Eur. J.* **2020**, *26* (67), 15738–15745.
